# Regioselective Hydroxylation of Unsymmetrical Ketones Using Cu, H_2_O_2_, and Imine Directing Groups via Formation of an Electrophilic Cupric Hydroperoxide Core

**DOI:** 10.1021/acs.joc.3c02647

**Published:** 2024-02-07

**Authors:** Shuming Zhang, Sunipa Goswami, Karl H. G. Schulz, Karan Gill, Xinyi Yin, Jimin Hwang, Jasmine Wiese, Isabel Jaffer, Roberto R. Gil, Isaac Garcia-Bosch

**Affiliations:** Department of Chemistry, Carnegie Mellon University, Pittsburgh, Pennsylvania 15213, United States

## Abstract

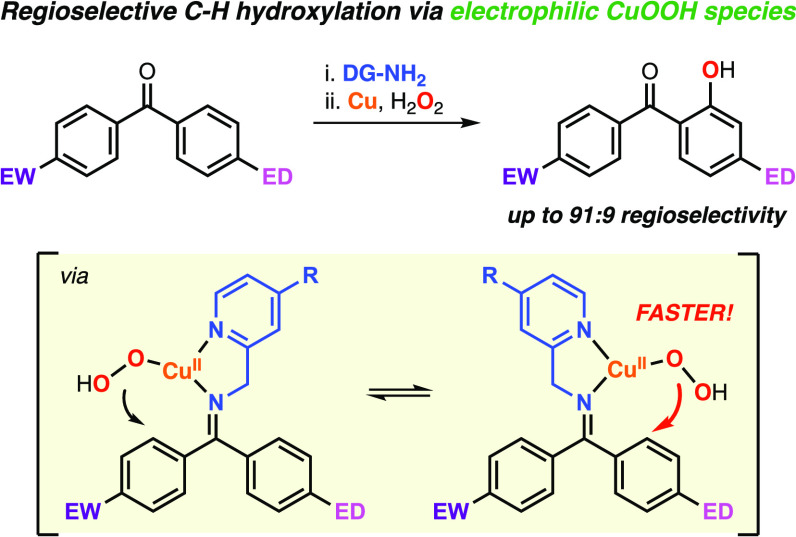

Herein, we describe the regioselective functionalization of unsymmetrical ketones using imine directing groups, Cu, and H_2_O_2_. The C–H hydroxylation of the substrate–ligands derived from 2-substituted benzophenones occurred exclusively at the γ-position of the unsubstituted ring due to the formation of only one imine stereoisomer. Conversely, the imines derived from 4-substituted benzophenones produced *E*/*Z* mixtures that upon reacting with Cu and H_2_O_2_ led to two γ-C–H hydroxylation products. Contrary to our initial hypothesis, the ratio of the hydroxylation products did not depend on the ratio of the *E*/*Z* isomers but on the electrophilicity of the reactive [LCuOOH]^1+^. A detailed mechanistic analysis suggests a fast isomerization of the imine substrate–ligand binding the CuOOH core before the rate-determining electrophilic aromatic hydroxylation. Varying the benzophenone substituents and/or introducing electron-donating and electron-withdrawing groups on the 4-position of pyridine of the directing group allowed for fine-tuning of the electrophilicity of the mononuclear [LCuOOH]^1+^ to reach remarkable regioselectivities (up to 91:9 favoring the hydroxylation of the electron-rich arene ring). Lastly, we performed the C–H hydroxylation of alkyl aryl ketones, and like in the unsymmetrical benzophenones, the regioselectivity of the transformations (sp^3^ vs sp^2^) could be controlled by varying the electronics of the substrate and/or the directing group.

## Introduction

**Figure 1:**
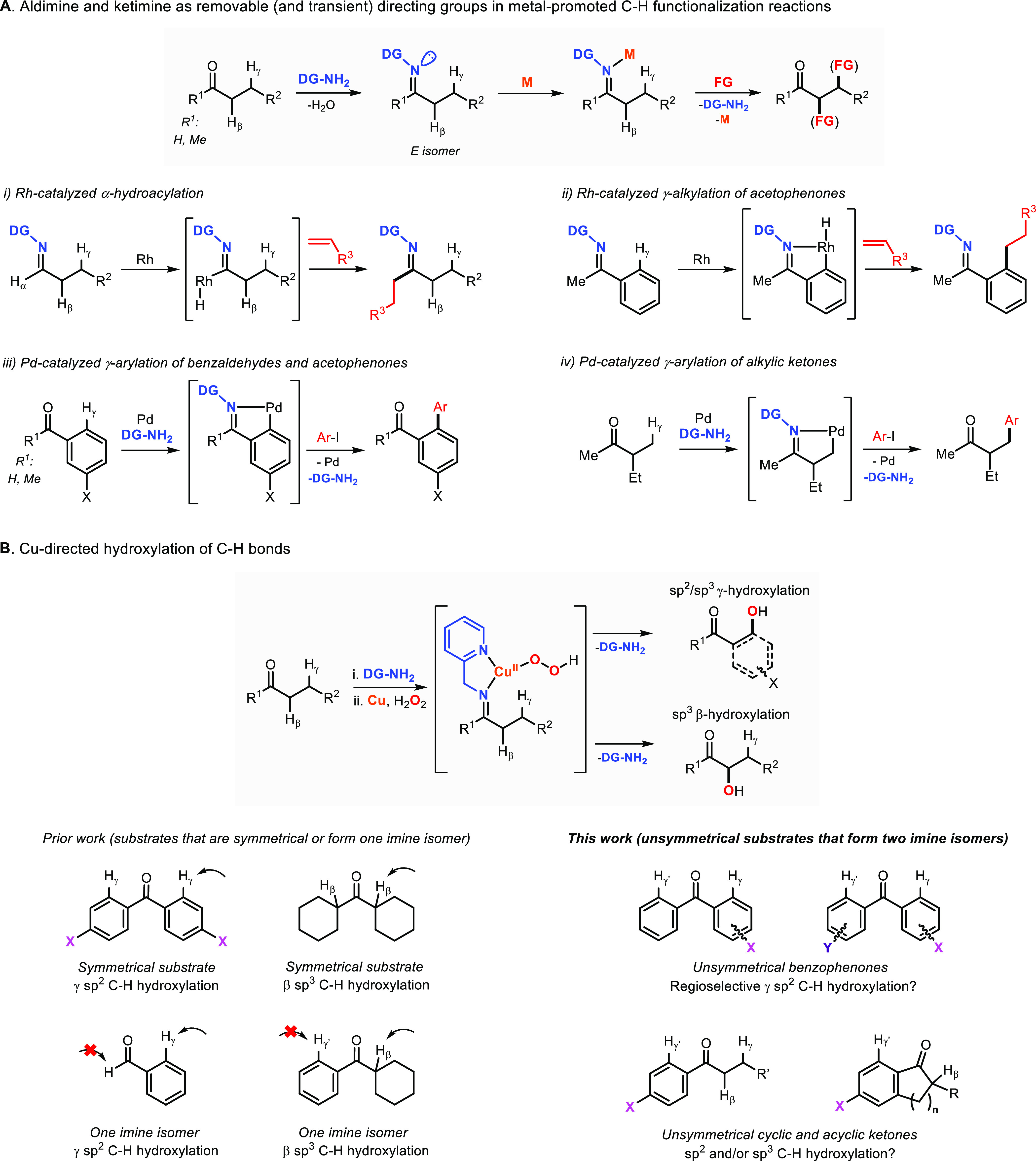
(A) Metal-promoted C–H functionalization of aldehydes and ketones using removable imine directing groups.^8−12^ (B) Cu-directed hydroxylations using imino-pyridine directing groups.^17−20^

The direct functionalization of C–H bonds is a powerful tool for organic synthesis.^1,2^ A vast array of metal-promoted C–H functionalization reactions utilize directing groups (DGs) to achieve selectivity.^3−5^ Imine directing groups are widely used due to their facile installation (i.e., condensation between amine and aldehyde or ketone) and removal, which, in selected examples, allows for their utilization as transient directing groups.^6,7^ The regioselectivity of these metal-promoted C–H functionalization reactions is usually based on the formation of one of the imine stereoisomers (*E*), which is usually accomplished by employing aldehydes and methyl ketones as substrates (Figure 1A). With the exception of the Rh-catalyzed α-C–H hydroacylation of aldimines,^8−10^ the metal is proposed to coordinate to the imine N donor, which promotes the selective activation of the β- and γ-C–H bonds of the most sterically hindered carbonyl substituent (i.e., functionalization occurs at substituent *trans* to the directing group N-substituent, see Figure 1A).^8,11,12^

Inspired by the reactivity of Cu-dependent monooxygenase/peroxygenase enzymes,^13−16^ our research laboratory has published a series of papers that describe the utilization of imine directing groups, Cu, and H_2_O_2_ to perform the γ hydroxylation of sp^2^ and sp^3^ C–H bonds and the β hydroxylation of sp^3^ C–H bonds (Figure 1B; Note: following literature precedents on Cu-directed hydroxylation reactions, we named the carbonyl of the ketone C_α_ and the adjacent position C_β_).^17−20^ The selectivity of these Cu-promoted oxidations, which were initially developed by Schöenecker and co-workers for the selective oxidation of steroids^21^ and have been applied to the total synthesis of complex molecules,^22−24^ also relies on the formation of only one of the imine isomers in unsymmetrical substrates (e.g., cyclohexyl phenyl ketone^18^), an issue that can be avoided in the hydroxylation of symmetrical substrates (e.g., symmetrical benzophenones^18^). In this paper, we studied the regioselectivity in the Cu-directed hydroxylation of unsymmetrical ketones (Figure 1B). We initially hypothesized that the regioselectivity of these Cu-promoted C–H hydroxylation reactions would be determined by the ratio of the imine *E*/*Z* isomers of the substrate–ligand. Contrary to our initial thoughts, the regioselectivity of these transformations is dictated by the electrophilicity of the hydroxylating Cu^II^OOH intermediate.

## Results and Discussion

### Synthesis of Imines Derived from 2-Picolylamine and Unsymmetrical Benzophenone, and Determination of Imine *E*/*Z* Isomer Ratio

**Figure 2:**
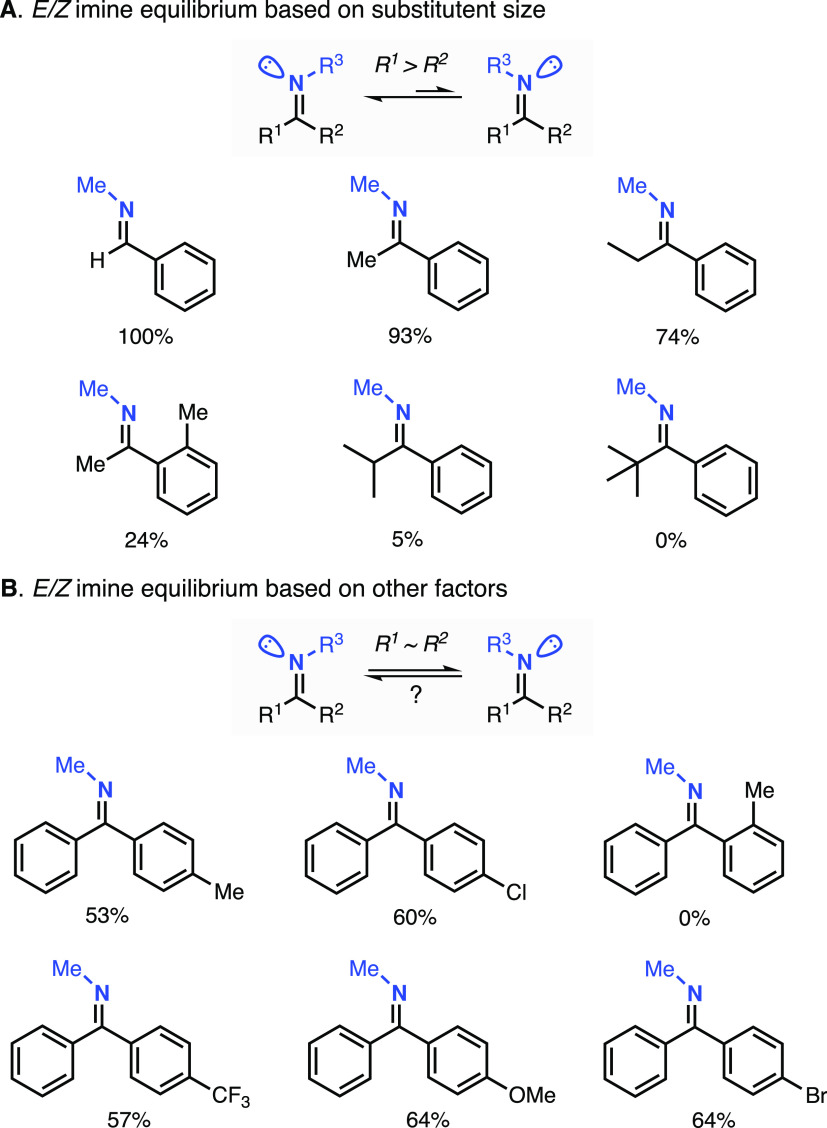
*E*/*Z* equilibrium for imines derived from benzaldehyde, alkyl aryl ketones, and substituted benzophenones.^25^

The formation of *E*/*Z* imine isomers derived from benzophenones and other aryl ketones (e.g., acetophenones) was studied in detail by Boyd and co-workers several decades ago (Figure 2).^25^ As expected, the *E*/*Z* ratios in imines are highly dependent on steric factors, in which the bulkier group on the carbon atom is positioned *trans* to the nitrogen substituent (e.g., imines derived from benzaldehyde only form the *E*-isomer in solution, see Figure 2A). In the case of ketones containing substituents with similar size (e.g., substituted benzophenones), it was found that the ratio of *E*/*Z* isomers is highly dependent on the position of the substituents. Imines derived from 2-substituted benzophenones only produced *Z* isomers while 4-substituted benzophenones produced mixtures (ca. *E/Z*: 60/40). Boyd and co-workers suggested that two competing effects determined the *E*/Z ratio of the mixtures, a stabilizing planar resonance effect between the aromatic substituent and the imine double bond and a destabilizing steric/electronic repulsion between the aromatic substituents. For 2-substituted benzophenones, it was proposed that the aromatic ring containing the substituent twists to avoid steric repulsion with the unsubstituted ring, which adopts a planar conformation with the imine double bond. For all of the imines analyzed, minor changes in the *E*/*Z* ratios were found upon changing the imine substituent or when different solvents were utilized.

**Figure 3:**
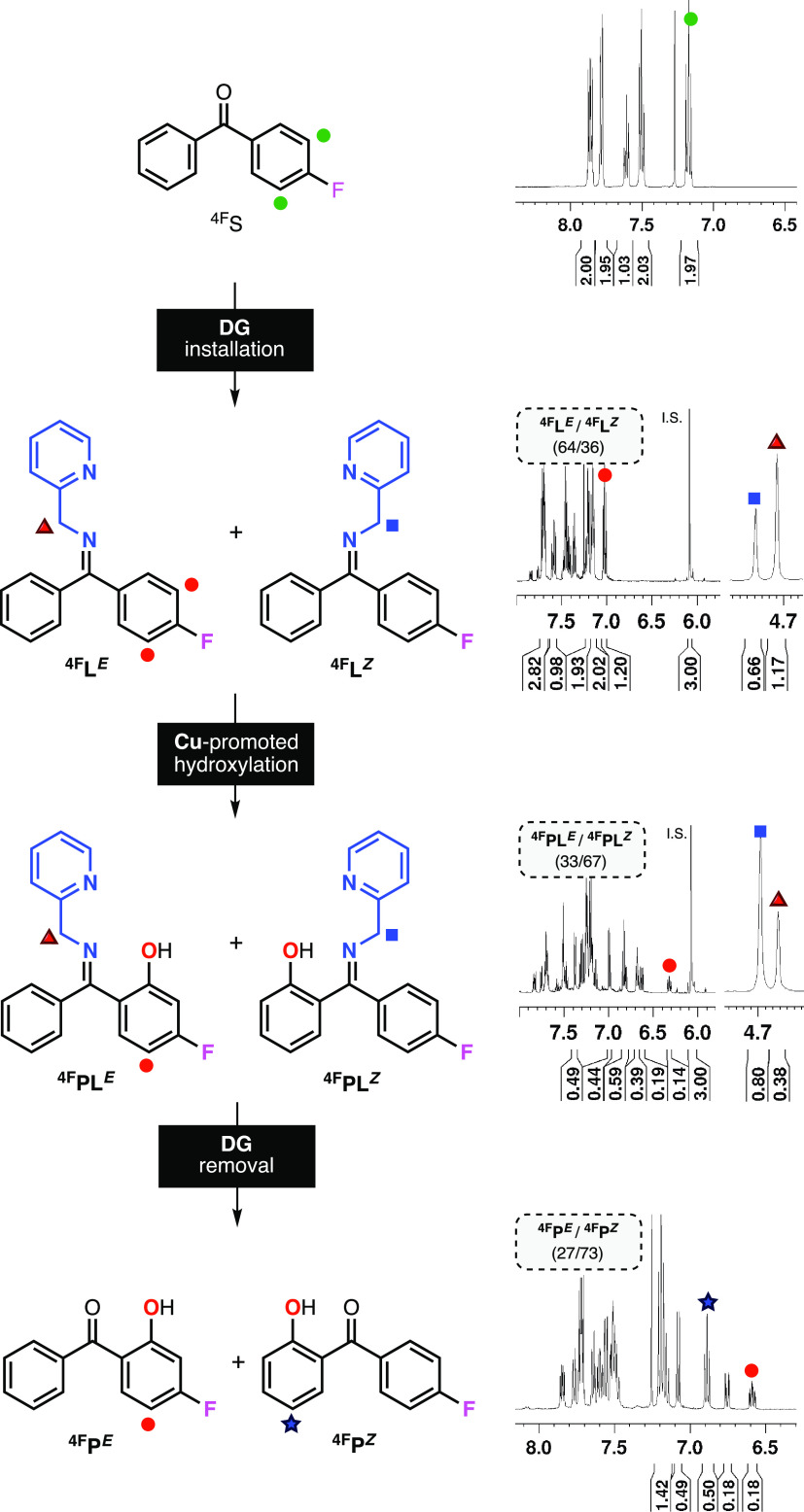
NMR analysis of the Cu-directed hydroxylation of unsymmetrical benzophenones (Note: as an example, we included the data for 4-fluorobenzophenone). Characterization and quantification of the reaction products were carried out in CDCl_3_ using 2,4,6-trimethoxybenzene as an internal standard (I.S.). See the SI for further details.

In this work, the synthesis of the imine substrate–ligands derived from substituted benzophenones and 2-picolylamine was carried out in a Dean–Stark apparatus using toluene as the solvent and catalytic amounts of *p*-toluenesulfonic acid monohydrate (see example in Figure 3; see also the Supporting Information, SI, for details on the synthetic procedure and characterization of the imine substrate–ligands). After isolation of the imine substrates, we determined the ratio of *E*/*Z* isomers by NMR (^1^H NMR, ^13^C NMR, COSY, and NOESY, see the SI). The imine systems derived from the 2-substituted benzophenones (2-MeO, 2-Me, 2-F, 2-Cl, and 2-Br) produced only the *Z* isomer, in agreement with the report by Boyd and co-workers.^25^ For imines derived from 4-substituted benzophenones (4-MeO, 4-Me, 4-F, 4-Cl 4-Br, and 4-CF_3_), we observed mixtures of *E*/*Z* isomers in which the *E* isomers were slightly more favored than the *Z* (ca. *E*/Z: 60/40), also in agreement with literature precedents.^25^

### Hydroxylation of Imine Substrate–Ligands

An example of the protocol we followed to analyze the Cu-promoted hydroxylation of unsymmetrical benzophenones is included in Figure 3 (hydroxylation of 4-fluorobenzophenone using 2-picolylamine as a directing group). After synthesizing the imine substrate–ligand and determining the purity and *E*/*Z* ratio by NMR (see ^**4F**^**L**^***E***^ and ^**4F**^**L**^**Z**^ in Figure 3), we carried out the hydroxylation in acetone at room temperature using 1 equiv of [Cu^I^(CH_3_CN)_4_](PF_6_) and 5 equiv of 30% aqueous H_2_O_2_. NMR analysis allowed for determining the hydroxylation yield, the mass balance of the reaction, and the ratio of hydroxylation products derived from the imine substrate–ligand *E*/*Z* isomers (see ^**4F**^**PL**^***E***^ and ^**4F**^**PL**^**Z**^ in Figure 3). Removal of the directing group was accomplished using aqueous 1 M HCl, and the ratio of the resulting hydroxy benzophenone products agreed with the *E*/*Z* ratio calculated before imine cleavage (e.g., ^**4F**^**PL**^***E***^/^**4F**^**PL**^**Z**^ ∼ ^**4F**^**L**^***E***^/^**4F**^**L**^**Z**^, see Figure 3 and SI for further details).

**Figure 4:**
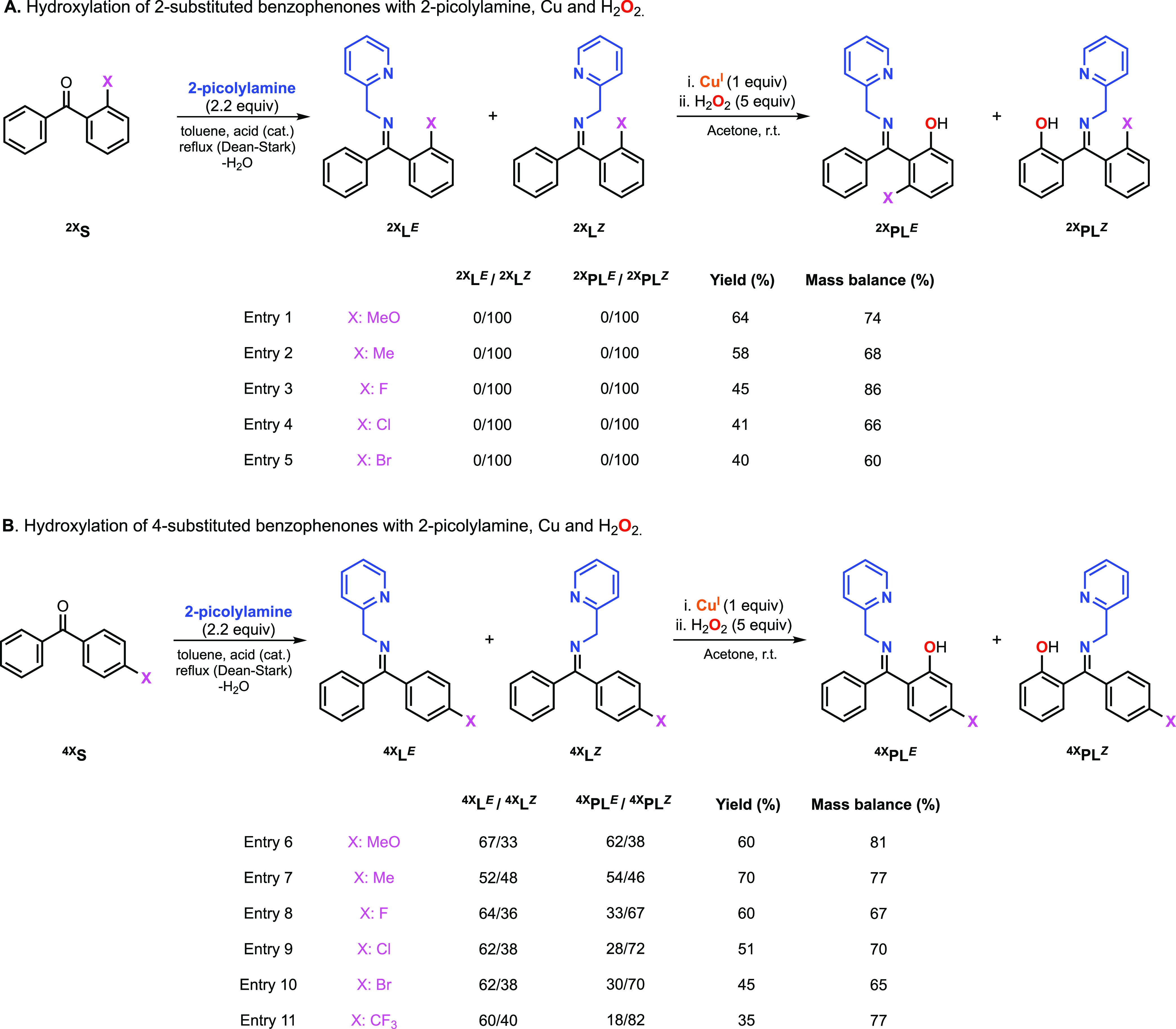
Cu-promoted hydroxylation of imine substrate–ligands derived from 2-substituted benzophenones (A) and 4-substituted benzophenones (B) using 2-picolylamine as a directing group and H_2_O_2_ as an oxidant (see the SI for experimental details on the synthesis of substrate–ligands, hydroxylation reactions, and directing group removal).

In the hydroxylation of the substrate–ligands derived from 2-substituted benzophenones, we observed selective γ sp^2^ C–H hydroxylation of the unsubstituted phenyl ring (^**2X**^**PL**^***E***^/^**2X**^**PL**^***Z***^, 0/100), which agreed with the presence of only one of the imine isomers in the starting substrate–ligands (^**2X**^**L**^***E***^/^**2**^**L**^***Z***^, 0/100, see Figure 4A). The hydroxylation yields (from 40 to 64%) and the mass balance of the reactions (from 60 to 84%) were comparable to the results obtained in our previous publications.

The Cu-promoted hydroxylation of the imine systems derived from the 4-substituted benzophenones also led to hydroxylation products with good yields (35–70%) and good mass balance (65–81%, see Figure 4B). Unexpectedly, the ratio of the two hydroxylation products, namely, ^**4X**^**PL**^***E***^ and ^**4X**^**PL**^***Z***^, did not match the *E*/*Z* ratio of the initial imine substrate–ligands. While the Cu-promoted oxidation of ^**4MeO**^**L** (*E*/*Z*: 67/33) and ^**4Me**^**L** (*E*/*Z*: 52/48) produced hydroxylation products with *E*/*Z* ratios similar to the imine substrate–ligands (e.g., ^**4MeO**^**PL**^***E***^/^**4MeO**^**PL**^***Z***^: 62/38), we observed that ^**4F**^**L** (*E*/*Z*: 64/36), ^**4Cl**^**L** (*E*/*Z*: 62/38), ^**4Br**^**L** (*E*/*Z*: 62/38), and ^**4CF3**^**L** (*E*/*Z*: 60/40) favored the formation of the *Z* hydroxylation product (e.g., ^**4CF3**^**PL**^***E***^/^**4CF3**^**PL**^***Z***^: 18/82). Thus, it appeared that the regioselectivity for the hydroxylation of the 4-substituted systems was determined by the electronics of the substituent, with the electron-donating substituents favoring the functionalization of the substituted phenyl ring and the electron-withdrawing substituents favoring the functionalization of the unsubstituted phenyl ring.

### Mechanistic Studies on Regioselectivity

**Figure 5:**
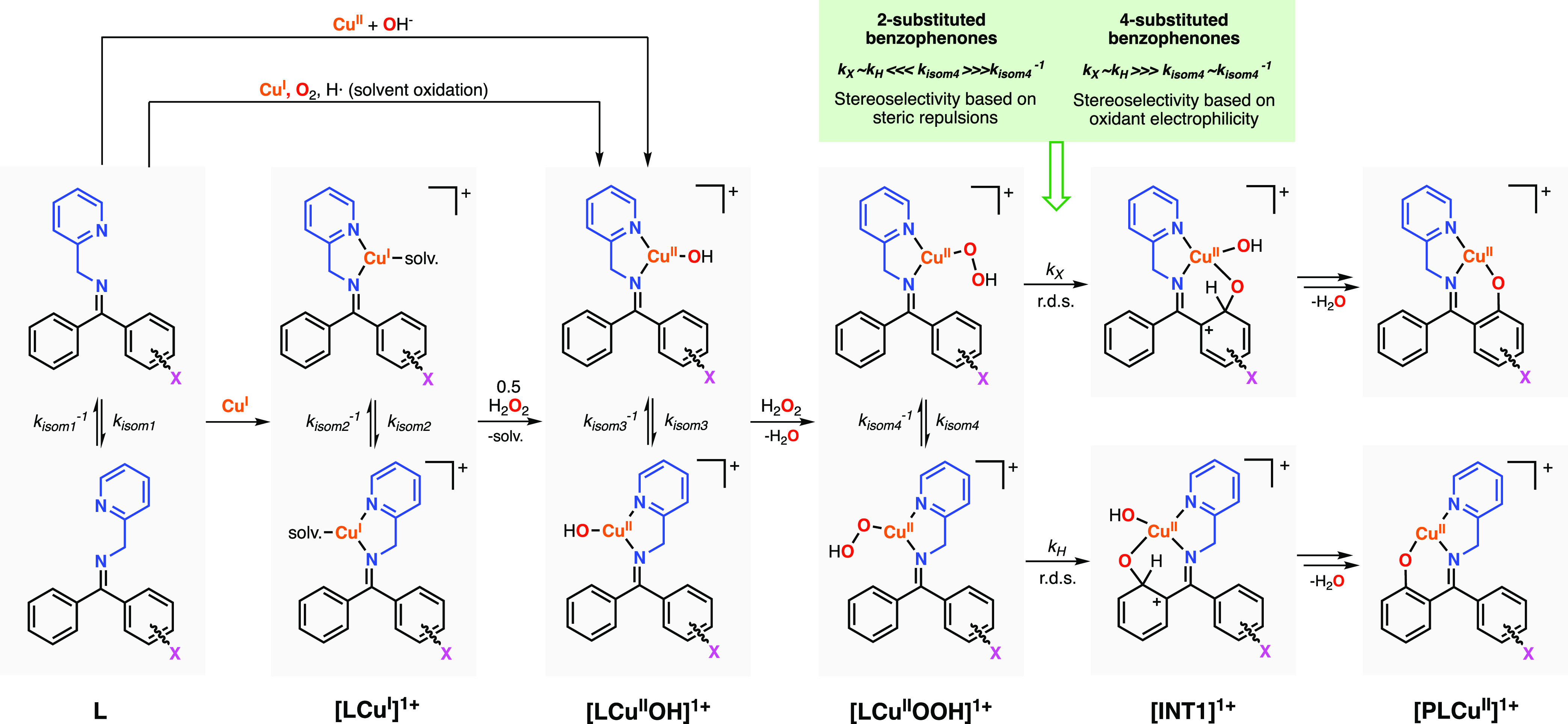
Mechanistic proposal for the Cu-directed hydroxylation of substrate–ligands derived from unsymmetrical benzophenones.

The proposed mechanism for the regioselective Cu-directed hydroxylation of sp^2^ C–H bonds is depicted in Figure 5. In our previous publications, we have proposed that the hydroxylation of aryl substrates (e.g., imine substrate–ligands derived from symmetrical benzophenones) occurred via mononuclear Cu/O_2_ species.^18^ Our experimental and theoretical data supported the formation of a Cu^II^-hydroperoxo intermediate before the rate-determining step of the reaction. The [LCu^II^OOH]^1+^ species can be formed by adding H_2_O_2_ to copper(I) or copper(II) sources or by oxidation of [LCu^I^]^1+^ with O_2_, in which copper(II) and H_2_O_2_ (a real oxidant) are formed via superoxide disproportionation and solvent oxidation.^17^ For the unsymmetrical benzophenones described herein, we hypothesized that for each of the mononuclear species formed before the r.d.s., an equilibrium between the *E/Z* imine isomers exists but the ratio of the hydroxylation products is solely determined during the rate-determining step. We have previously shown that during the r.d.s., the [LCu^II^OOH]^1+^ species oxidizes the aromatic sp^2^ C–H bonds via a concerted heterolytic O–O bond cleavage with concomitant electrophilic attack on the arene system.^18^

**Figure 6:**
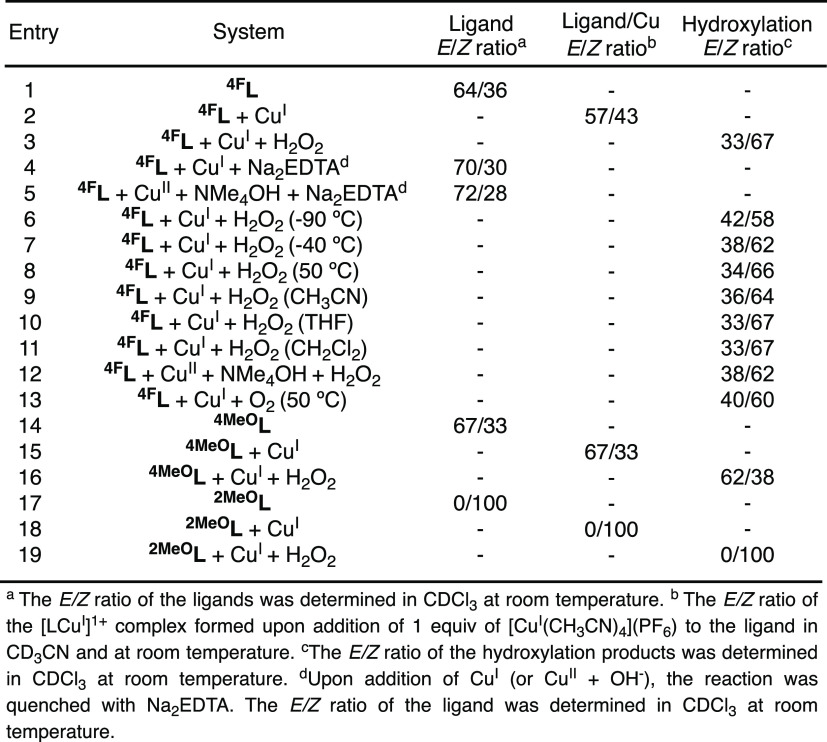
Determination of the *E/Z* ratio of ligands, cuprous complexes, and hydroxylation products under different reaction conditions.

For some of the imine substrate–ligands analyzed, we observed that the addition of Cu^I^ induced a change in the *E*/*Z* ratios (Figure 6). The change in the *E/Z* ratio could be analyzed by ^1^H NMR due to the formation of diamagnetic [LCu^I^]^1+^ species in solution (see the SI for details on NMR analysis). Addition of 1 equiv of Cu^I^ to ^**4F**^**L** (*E/Z*: 70/30) shifted the equilibrium to **[**^**4F**^**LCu**^**I**^**]**^**1+**^ (*E/Z*: 57/43, entry 2 in Figure 6). However, the change in the ratio upon addition of Cu^I^ could not account for the *E/Z* ratio of the hydroxylation product, ^**4F**^**PL** (*E/Z*: 38/62, entry 3 in Figure 6). We also analyzed the ratio of the ^**4F**^**L** imine isomers upon addition of copper (Cu^I^ or Cu^II^) and removal of Cu with Na_2_EDTA (entries 4 and 5 in Figure 6), and we observed no major changes in the imine substrate–ligand *E*/*Z* ratios, suggesting that the coordination of Cu did not lead to irreversible isomerization of the imine substrate–ligands. Hydroxylation of ^**4F**^**L** with Cu^I^ and H_2_O_2_ at different temperatures (entries 6 to 8 in Figure 5) and with different solvents (entries 9 to 11 in Figure 6) did not substantially change the *E*/*Z* hydroxylation ratios. Like in the hydroxylation of ^**4F**^**L** with Cu^I^ and H_2_O_2_, the oxidations using Cu^II^, NMe_4_OH, and H_2_O_2_ led to an *E*/*Z* ratio of 38/62 (entry 12 in Figure 6). Likewise, the hydroxylation of ^**4F**^**L** with Cu^I^ and O_2_ at 50 °C led to similar *E*/*Z* ratios (entry 13 in Figure 6).

For ^**4MeO**^**L** (*E*/*Z*: 67/33), no change in the *E*/*Z* ratio was observed upon Cu^I^ addition **[**^**4MeO**^**LCu**^**I**^**]**^**1+**^ (*E*/*Z*: 67/33, see entries 14 and 15 in Figure 6), while slight changes in the E/Z ratios were observed after hydroxylation (^**4MeO**^**PL**^***E***^/^**4MeO**^**PL**^***Z***^: 62/38, see entry 16 in Figure 6). The addition of Cu^I^ to ^**2MeO**^**L** (*E*/*Z*: 0/100) did not generate the E-isomer for **[**^**4MeO**^**LCu**^**I**^**]**^**1+**^ (*E*/*Z*: 0/100), in agreement with the formation of one hydroxylation product upon addition of Cu/H_2_O_2_ (^**2MeO**^**PL**^***E***^/^**2MeO**^**PL**^***Z***^: 0/100, see entries 17–19 in Figure 6).

Overall, coordination of Cu^I^ to the 4-substituted substrate–ligands caused a change in the *E/Z* ratio, while no change in the *E/Z* ratio was observed in the cuprous complexes of the 2-substituted analogues. However, these changes in the *E/Z* ratio of the imine substrate–ligands upon addition of Cu^I^ did not match the ratios of the hydroxylation products, suggesting that the addition of H_2_O_2_ triggers the formation of the electrophilic [LCu^II^OOH]^1+^ intermediate, which will determine the regioselectivity of these reactions during the rate-determining step (see Figure 5).

### Redox Potentials and Electrophilicity

**Figure 7:**
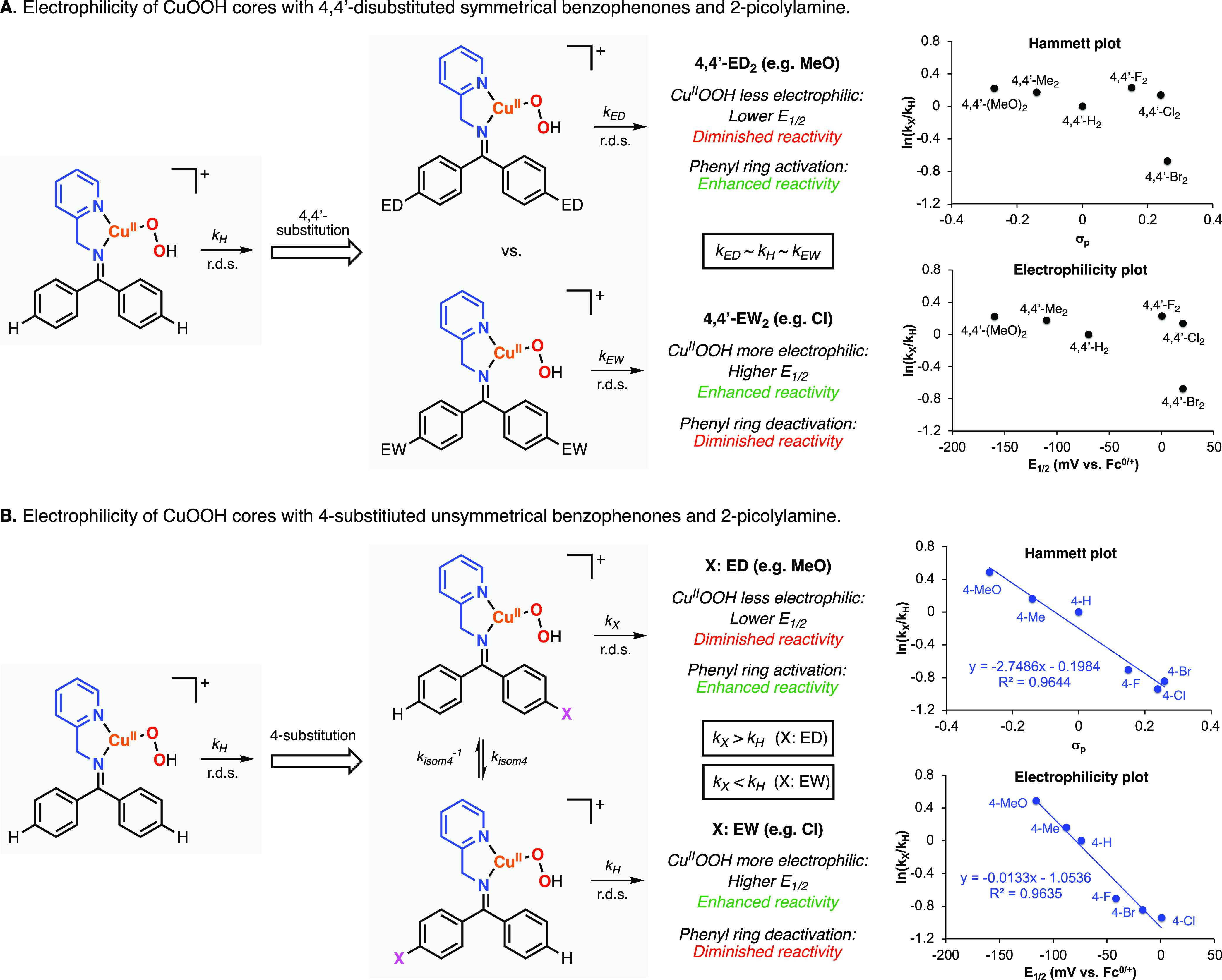
Electrophilicity of the CuOOH cores derived from 4,4′-disubstituted benzophenones^18^ (A) and 4-substituted benzophenones (B) with 2-picolylamine as a directing group. Note: the Hammett plot included in panel B was constructed by plotting the relative reaction rates for the hydroxylation of the substituted (*k*_X_) and unsubstituted (*k*_H_) rings (*k*_X_/*k*_H_ obtained from the ^**4X**^**PL**^***E***^**/**^**4X**^**PL**^**Z**^ ratios) vs the σ_p_ values. The electrophilicity plot included in panel B was constructed by plotting *k*_X_/*k*_H_ vs the *E*_1/2_ values of the Cu^I^ complexes derived from 2-picolylamine and 4-substituted benzophenones.

In our previous publication on the Cu-directed hydroxylation of imine substrates derived from symmetrical 4,4′-disubstituted benzophenones and 2-picolylamine, we observed that the decay of the reactive [LCuOOH]^1+^ species (rate-determining step) was not influenced by the substituents on the benzophenone.^18^ We rationalized those findings based on the electrophilicity of the reactive CuOOH core and the relative reactivity of the substituted phenyl rings (Figure 7A). When compared to benzophenone, the system derived from 4,4′-dimethoxybenzophenone produced a CuOOH core with diminished electrophilicity, which was compensated for by the higher reactivity of the arene substrate. Conversely, the system derived from 4,4′-dichlorobenzophenone produced a CuOOH core with enhanced electrophilicity, which was compensated for by the lower reactivity of the arene substrate. To provide experimental evidence for the electrophilicity of the CuOOH cores, we measured the reduction potentials of the LCu^I^ complexes,^26−29^ and we observed remarkable variations of *E*_1/2_ (*E*_1/2_: −160 mV vs Fc^0/+^ for 4,4′-dimethoxybenzophenone; *E*_1/2_: 20 mV vs Fc^0/+^ for 4,4′-dichlorobenzophenone).

Based on the proposed mechanism depicted in Figure 5, the *E*/*Z* isomers of the reactive [LCu^II^OOH]^1+^ are in fast equilibrium and the isomer that directs the electrophilic CuOOH core toward the more electron-rich ring will react faster than the isomer that directs the CuOOH core toward the electron-poor ring (Figure 7B). Hence, the hydroxylation of the imines derived from 4-substituted benzophenones with electron-donating substituents (4-MeO and 4-Me) favors the hydroxylation of the substituted ring (electron-rich arene), and the hydroxylation of the imines derived from 4-substituted benzophenones with electron-withdrawing substituents (4-F, 4-Cl, 4-Br, and 4-CF_3_) favors the hydroxylation of the unsubstituted ring (which is also the electron-rich arene). The relative reaction rates for the hydroxylation of substituted (*k*_X_) and unsubstituted (*k*_H_) rings can be obtained from the ^**4X**^**PL**^***E***^**/**^**4X**^**PL**^**Z**^ ratios (^**4X**^**PL**^***E***^**/**^**4X**^**PL**^**Z**^**=***k*_X_/*k*_H_), which allowed constructing a Hammett plot (Figure 7B). The slope of the Hammett plot (ρ = −2.6) is consistent with the involvement of an electrophilic oxidant during the r.d.s.^30−32^

Like we did for the systems derived from symmetrical 4,4′-disubstituted benzophenones, we measured the *E*_1/2_ for the 1-electron oxidation of the Cu^I^ complexes derived from 2-picolylamine and unsymmetrical 4-substituted benzophenones (Figure 7B, see also SI for experimental details). When the *E*_1/2_ values were plotted against the relative reaction rates, we obtained a linear correlation (slope: −0.013), suggesting that the electrophilicity of the CuOOH core could be benchmarked by measuring the *E*_1/2_ values of the Cu complexes. Moreover, we envisioned that the regioselectivity of the Cu-directed hydroxylation reactions could be potentially controlled by changing the electrophilicity of the CuOOH core via substrate and/or directing group modification (see sections below).

### Hydroxylation of 4,4′-Disubstituted Benzophenones

**Figure 8:**
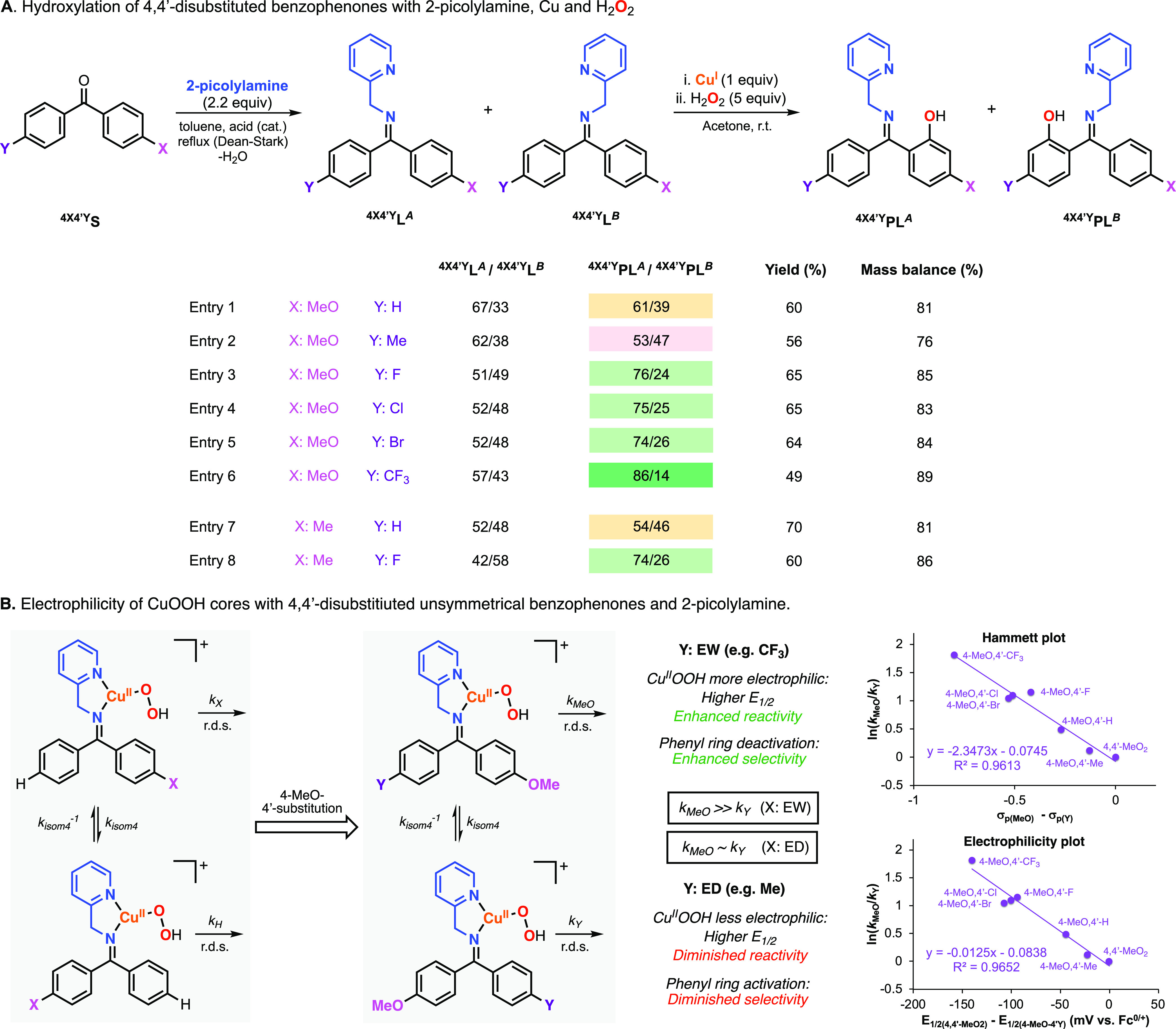
(A) Cu-promoted hydroxylation of systems derived from 4,4′-disubstituted benzophenones and 2-picolylamine using H_2_O_2_ as an oxidant (see the SI for experimental details on the synthesis of substrate–ligands and hydroxylation reactions). Note: the regioselectivity results (^**4×4**^′^**Y**^**PL**^***A***^/^**4×4**^′^**Y**^**PL**^***B***^ column) are color-coded according to orange (results obtained for 4-substituted benzophenones), red (worsened regioselectivity for 4,4′-disubstituted benzophenones when compared to 4-substituted benzophenone analogue), or green (improved regioselectivity for 4,4′-disubstituted benzophenones when compared to 4-substituted benzophenone analogue). (B) Electrophilicity of the CuOOH cores involved in the hydroxylation of systems derived from unsymmetrical 4,4′-disubstituted benzophenones, 2-picolylamine, and H_2_O_2_. Note: the Hammet plot included in (B) was constructed by plotting the relative reaction rates between the hydroxylation of the arene ring containing the MeO substituent (*k*_MeO_) and the hydroxylation of the other arene ring (*k*_Y_, Y: Me, H, F, Cl, CF_3_); (*k*_MeO_/*k*_Y_, calculated from the ^**4MeO4**^′^**Y**^**PL**^***A***^**/**^**4MeO4**^′^**Y**^**PL***^**B**^* ratios) vs the difference between the σ_p_ value of MeO and the σ_p_ value of other substituents. The electrophilicity plot was constructed by plotting *k*_MeO_/*k*_Y_ vs the difference in the *E*_1/2_ values of the Cu^I^ complexes derived from 2-picolylamine and 4,4′-dimethoxybenzophenone or unsymmetrical 4,4′-disubstituted benzophenone.

Based on the mechanistic findings described above, we hypothesized that the utilization of 4,4′-disubstituted benzophenones could enhance the regioselectivity of the Cu-directed hydroxylation reactions (Figure 8). Imine systems derived from 4,4′-disubstituted benzophenones containing electron-rich substituents on one of the arene rings (e.g., X: MeO) and electron-poor substituents on the other arene ring (e.g., Y: F, Cl, Br, CF_3_) were synthesized, and the *A*/*B* isomers ratios were determined by NMR (Note: instead of *E*/*Z* isomers, these are denoted as *A*/*B* to avoid misunderstanding).

As we expected, the electrophilic character of the putative CuOOH intermediate favored the oxidation of the electron-rich phenyl ring. For example, ^**4MeO4**^′^**F**^**L** (*A*/*B*: 51/49, see Figure 8A) was oxidized with Cu^I^ and H_2_O_2_ to produce hydroxylation products with remarkable regioselectivity (^**4MeO4**^′^**F**^**PL**^***A***^/^**4MeO4**^′^**F**^**PL**^***B***^: 76/24). Similar regioselectivities were observed in the hydroxylation of ^**4MeO4**^′^**Cl**^**L** (^**4MeO4**^′^**Cl**^**PL**^***A***^/^**4MeO4**^′^**Cl**^**PL**^***B***^: 75/25) and ^**4Me4**^′^**F**^**L** (^**4Me4**^′^**F**^**PL**^***A***^/^**4Me4**^′^**F**^**PL**^***B***^: 74/26). Like we observed in the 4-substituted benzophenones, the introduction of the electron-withdrawing CF_3_ substituent in ^**4MeO4**^′^**CF3**^**L** enhanced significantly the selectivity for the hydroxylation of the electron-rich arene ring (^**4MeO4**^′^**CF3**^**PL**^***A***^/^**4MeO4**^′^**CF3**^**PL**^***B***^: 86/14). Conversely, the intramolecular competition between the 4-MeO-substituted and 4-Me-substituted arene rings in ^**4MeO4**^′^**Me**^**L** led to a slight excess of one of the hydroxylation products (^**4MeO4**^′^**Me**^**PL**^***A***^/^**4MeO4**^′^**Me**^**PL**^***B***^: 53/47).

The relative reaction rates between the hydroxylation of the arene ring containing the MeO substituent (*k*_MeO_) and the hydroxylation of the other arene ring (*k*_Y_, Y: Me, H, F, Cl, CF_3_) can be calculated from the ^**4MeO4**^′^**Y**^**PL**^***A***^**/**^**4MeO4**^′^**Y**^**PL**^**B**^ ratios (^**4MeO4**^′^**Y**^**PL**^***A***^**/**^**4MeO4**^′^**Y**^**PL**^**B**^**=***k*_MeO_/*k*_Y_), which allowed constructing a Hammett plot (Figure 8B; Note: for the *x* axis, we used the difference between σ_p(MeO)_ and σ_p(Y)_). The slope obtained (ρ = −2.38) is similar to the value obtained in the Hammett plot constructed with the systems derived from 4-substituted benzophenones (see Figure 7B), suggesting the involvement of an electrophilic oxidant (CuOOH core) during the rate-determining step. Further support for this proposal was obtained when we plotted the relative hydroxylation rates (*k*_MeO_/*k*_Y_) against the difference between the *E*_1/2_ values of cuprous systems derived from the 4,4′-(MeO)_2_ and 4-MeO-4′-Y systems (electrophilicity plot in Figure 8B). The slope obtained (−0.0125) is analogous to the one found in the electrophilicity plot of the 4-substituted benzophenones systems (slope = −0.013, see Figure 7B), suggesting the formation of the same electrophilic species (CuOOH core) in both systems.

### Hydroxylation of Unsymmetrical 4-Substituted Benzophenones with Varying Directing Groups

**Figure 9:**
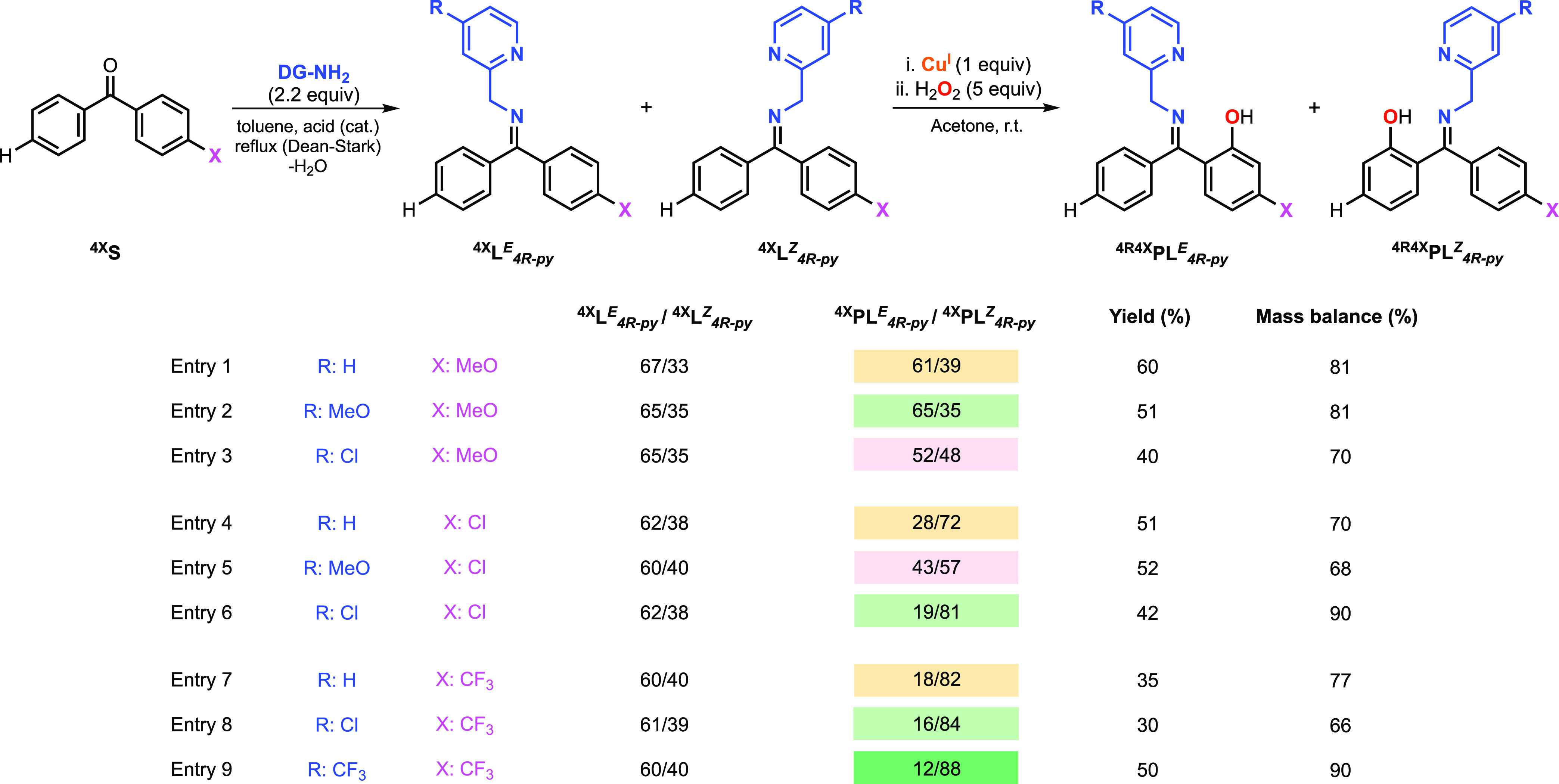
Cu-promoted hydroxylation of systems derived from unsymmetrical 4-substituted benzophenones and varying directing groups using H_2_O_2_ as an oxidant (see the SI for experimental details on the synthesis of substrate–ligands and hydroxylation reactions). Note: the regioselectivity results (^**4X**^**PL**_***4R-py***_^***E***^/^**4X**^**PL**_***4R-py***_^***Z***^ column) are color-coded according to orange (results obtained with 2-picolylamine), red (worsened regioselectivity for other DGs when compared to 2-picolylamine), or green (improved regioselectivity for other DGs when compared to 2-picolylamine).

The Cu-directed hydroxylation of 4-MeO-, 4-Cl-, and 4-CF_3_-benzophenone using different directing groups was also analyzed (Figure 9). Based on our mechanistic hypothesis, the utilization of directing groups with different electronics should alter the electrophilicity of the reactive CuOOH core, which should lead to changes in the regioselectivity. When 2-(aminomethyl)-4-methoxypyridine was utilized as DG instead of 2-picolylamine in the hydroxylation of 4-MeO-benzophenone, the regioselectivity improved (^**4MeO**^**PL**_***4MeO-py***_^***E***^/^**4MeO**^**PL**_***4MeO-py***_^***Z***^: 65/35) but worsened dramatically with 2-(aminomethyl)-4-chloropyridine (^**4MeO**^**PL**_***4Cl-py***_^***E***^/^**4MeO**^**PL**_***4Cl-py***_^***Z***^: 52/48). An opposite trend was observed in the hydroxylation of 4-Cl-benzophenone, in which the regioselectivity decreased when 2-(aminomethyl)-4-methoxypyridine was used (^**4Cl**^**PL**_***4MeO-py***_^***E***^/^**4Cl**^**PL**_***4MeO-py***_^***Z***^: 43/57) but increased substantially with 2-(aminomethyl)-4-chloropyridine (^**4Cl**^**PL**_***4Cl-py***_^***E***^/^**4Cl**^**PL**_***4Cl-py***_^***Z***^: 19/81).

These results seem to indicate a “substituent matching” effect in which enhanced selectivity is observed when substituents with similar donating ability are included in the substrate and the directing group (i.e., enhanced regioselectivity when both 4-substituted benzophenone and pyridine of the directing group contained electron-withdrawing Cl). In agreement with this trend, the regioselectivity for the hydroxylation of 4-CF_3_-benzophenone (CF_3_: electron-withdrawing) was enhanced when 2-aminomethyl-4-chloropyridine (^**4Cl**^**PL**_***4Cl-py***_^***E***^/^**4Cl**^**PL**_***4Cl-py***_^***Z***^: 16/84) and 2-aminomethyl-4-trifluoromethylpyridine (^**4Cl**^**PL**_***4CF3-py***_^***E***^/^**4Cl**^**PL**_***4CF3-py***_^***Z***^: 12/88) were utilized.

### Hydroxylation of Unsymmetrical 4,4′-Disubstituted Benzophenones with Varying Directing Groups

**Figure 10:**
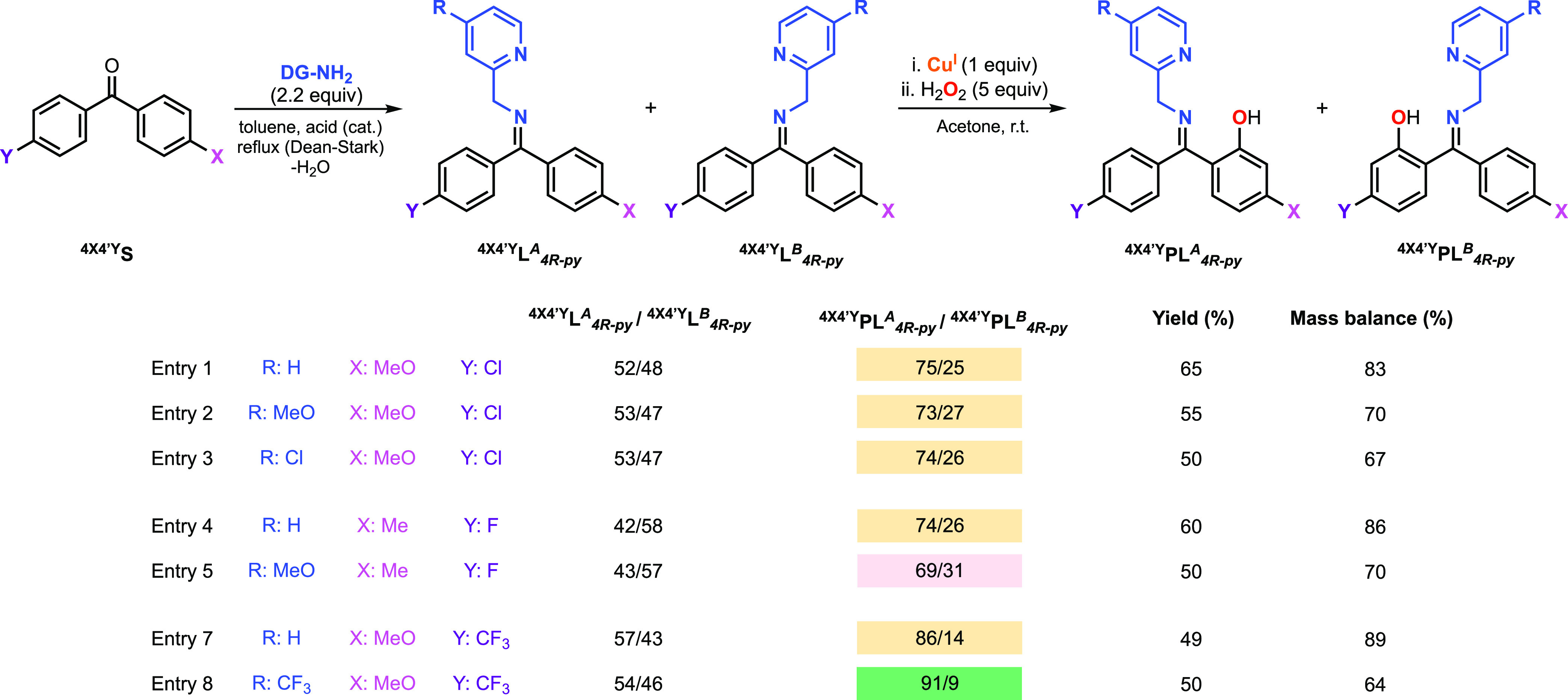
Cu-promoted hydroxylation of systems derived from unsymmetrical 4,4′-disubstituted benzophenones and varying directing groups using H_2_O_2_ as an oxidant (see the SI for experimental details on the synthesis of substrate–ligands and hydroxylation reactions). Note: the regioselectivity results (^**4×4**^′^**Y**^**PL**_***4R-py***_^***A***^/^**4×4**^′^**Y**^**PL**_***4R-py***_^***Z***^ column) are color-coded according to orange (results obtained with 2-picolylamine and results with other DGs that did not improve the regioselectivity when compared to 2-picolylamine), red (worsened regioselectivity for other DGs when compared to 2-picolylamine), or green (improved regioselectivity for other DGs when compared to 2-picolylamine).

The Cu-promoted hydroxylation of the imine substrate–ligands derived from unsymmetrical 4,4′-disubstituted benzophenones with varying directing groups was also explored (Figure 10). Small variations of the regioselectivity for 4-methoxy-4′-chlorobenzophenone were observed (entries 1 to 3) with 2-(aminomethyl)-4-methoxypyridine (^**4MeO4**^′^**Cl**^**PL**_***4MeO-py***_^***A***^/^**4MeO4**^′^**Cl**^**PL**_***4MeO-py***_^***B***^: 73/27) or 2-(aminomethyl)-4-chloropyridine (^**4MeO4**^′^**Cl**^**PL**_***4Cl-py***_^***A***^/^**4MeO4**^′^**Cl**^**PL**_***4Cl-py***_^***B***^: 74/26). A slight decrease in the regioselectivity was found in the hydroxylation of 4-methyl-4′-fluorobenzophenone when 2-(aminomethyl)-4-methoxypyridine was used when compared with 2-picolylamine (entries 4 and 5 in Figure 10). Conversely, the use of 2-(aminomethyl)-4-(trifluoro)methylpyridine in the hydroxylation of 4-methoxy-4′-(trifluoro)benzophenone led to the highest regioselectivity results in this article (^**4MeO4**^′^**CF3**^**PL**_***4CF3-py***_^***A***^/^**4MeO4**^′^**CF3**^**PL**_***4CF3-py***_^***B***^: 91/9).

## Hydroxylation of Acyclic Alkyl Aryl Ketones

**Figure 11:**
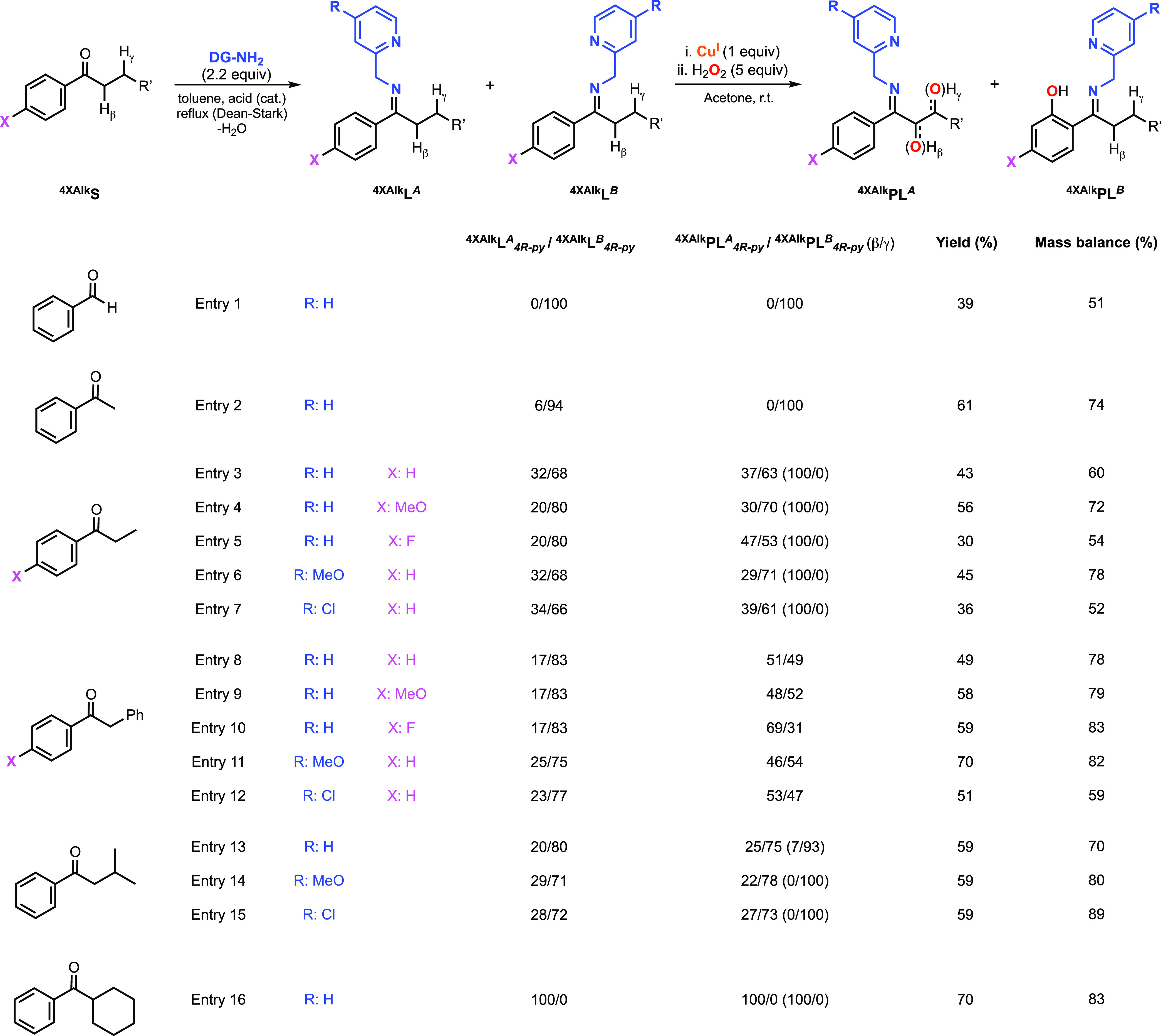
Cu-promoted hydroxylation of systems derived from acyclic alkyl aryl ketones and varying directing groups using H_2_O_2_ as an oxidant (see the SI for experimental details on the synthesis of substrate–ligands and hydroxylation reactions). Note: see the SI for details on the characterization and quantification of the products derived from β sp^3^ C–H oxidation, including hydroxylation and overoxidation products.

Encouraged by the results obtained in the regioselective hydroxylation of substituted benzophenones described above, we decided to explore the hydroxylation of a series of alkyl aryl ketones (Figure 11; Note: to avoid confusion, the imine isomer in which the directing group is facing the alkylic substituent is defined as the A isomer and the imine isomer in which the directing group is facing the aromatic ring is defined as the B isomer). In one of our previous reports, we described that the Cu-directed hydroxylation of benzaldehyde using 2-picolylamine and H_2_O_2_ led to selective γ sp^2^ C–H hydroxylation (see entries 1 in Figure 11).^18^ Conversely, the substrate–ligand derived from 2-picolylamine and cyclohexyl phenyl ketone underwent selective β sp^3^ C–H hydroxylation (entry 16, Figure 11). The regioselectivity of these transformations was attributed to the formation of one of the imine isomers (*B* isomer for benzaldehyde and *A* isomer for cyclohexyl phenyl ketone, see Figure 11).

For this article, we synthesized substrate–ligands derived from acetophenone, aryl-substituted propiophenones, aryl-substituted 2-phenylacetophenones, isovalerophenone, and 2,2-dimethylpropiophenone (see Figure 11). Variations in the size of the alkyl substituent led to changes in the substrate–ligand imine A/B ratios, in agreement with the findings described by Boyd and co-workers (see Figure 2A). For example, acetophenone produced the imine B isomer almost selectively (^**4XAlk**^**L**_**4R-py**_^***A***^/^**4XAlk**^**L**_**4R-py**_^***B***^: 6/94, entry 2 in Figure 11), while propiophenone produced substantial amounts of the imine isomer A (^**4XAlk**^**L**_**4R-py**_^***A***^/^**4XAlk**^**L**_**4R-py**_^***B***^: 32/68, entry 3 in Figure 11). The oxidation of the acetophenone substrate–ligand led to the selective formation of the sp^2^ hydroxylation product (entry 2 in Figure 11), while the oxidation of the propiophenone substrate–ligand formed products derived from the hydroxylation of the β sp^3^ C–H and γ sp^2^ C–H bonds (entry 3 in Figure 11; Note: see the SI for details on the characterization and quantification of the products derived from β sp^3^ C–H hydroxylation, which included diketone and benzoic acid as overoxidation products). Substitution of the para position of the aromatic ring of the propiophenone substrate led to substantial changes in the ^**4XAlk**^**PL**_**4R-py**_^***A***^/^**4XAlk**^**PL**_**4R-py**_^***B***^ ratios, with the MeO substituent favoring aromatic sp^2^ hydroxylation (^**4XAlk**^**PL**_**4R-py**_^***A***^/^**4XAlk**^**PL**_**4R-py**_^***B***^: 30/70, entry 4 in Figure 11) when compared with the unsubstituted (^**4XAlk**^**PL**_**4R-py**_^***A***^/^**4XAlk**^**PL**_**4R-py**_^***B***^: 37/63, entry 3 in Figure 11) and the 4-F substituted systems (^**4XAlk**^**PL**_**4-py**_^***A***^/^**4XAlk**^**PL**_**4-py**_^***B***^: 47/53, entry 5 in Figure 11). Interestingly, variations on the directing group also led to changes in the ^**4XAlk**^**PL**_**4R-py**_^***A***^/^**4XAlk**^**PL**_**4R-py**_^***B***^ ratio for the propiophenone substrate, with 2-(aminomethyl)-4-methoxypyridine enhancing sp^2^ hydroxylation (^**4XAlk**^**PL**_**4R-py**_^***A***^/^**4XAlk**^**PL**_**4R-py**_^***B***^: 29/71, entry 6 in Figure 11) when compared with 2-picolylamine (^**4XAlk**^**PL**_**4R-py**_^***A***^/^**4XAlk**^**PL**_**4R-py**_^***B***^: 37/63, entry 3 in Figure 11) and 2-(aminomethyl)-4-chloropyridine diminishing sp^3^ hydroxylation (^**4XAlk**^**PL**_**4R-py**_^***A***^/^**4XAlk**^**PL**_**4R-py**_^***B***^: 39/61, entry 7 in Figure 11).

The Cu-directed hydroxylation of the substrate–ligands derived from 2-phenylacetophenones was also analyzed (entries 8–12 in Figure 11). When compared to the propiophenone systems, we observed a higher tendency for sp^3^ hydroxylation (i.e., ^**4XAlk**^**PL**_**4R-py**_^***A***^/^**4XAlk**^**PL**_**4R-py**_^***B***^: 51/49 for 2-phenylacetophenone vs ^**4XAlk**^**PL**_**4R-py**_^***A***^/^**4XAlk**^**PL**_**4R-py**_^***B***^: 37/63 for propiophenone), which was attributed to the weakness of the β sp^3^ C–H bond in 2-phenylacetophenone (benzylic C–H bond) when compared to propiophenone (see entries 3 and 8 in Figure 11). While the introduction of a methoxy substituent in the para position of the 2-phenylacetopheone aromatic substrate led only to a modest enhancement of the sp^2^ hydroxylation (^**4XAlk**^**PL**_**4R-py**_^***A***^/^**4XAlk**^**PL**_**4R-py**_^***B***^: 48/52 vs ^**4XAlk**^**PL**_**4R-py**_^***A***^/^**4XAlk**^**PL**_**4R-py**_^***B***^: 51/49 for unsubstituted, see entries 8 and 9 in Figure 11), the use of an F substituent led to a remarkable enhancement of the sp^3^ hydroxylation (^**4XAlk**^**PL**_**4R-py**_^***A***^/^**4XAlk**^**PL**_**4R-py**_^***B***^: 69/31 vs ^**4XAlk**^**PL**_**4R-py**_^***A***^/^**4XAlk**^**PL**_**4R-py**_^***B***^: 51/49 for unsubstituted, see entries 8 and 10 in Figure 11). Variations on the electronics of the directing group had a small impact on the sp^3^/sp^2^ hydroxylation ratios, with 2-(aminomethyl)-4-methoxypyridine favoring sp^2^ hydroxylation (^**4XAlk**^**PL**_**4R-py**_^***A***^/^**4XAlk**^**PL**_**4R-py**_^***B***^: 46/54 vs ^**4XAlk**^**PL**_**4R-py**_^***A***^/^**4XAlk**^**PL**_**4R-py**_^***B***^: 51/49 for unsubstituted DG, see entries 8 and 11 in Figure 11) and 2-(aminomethyl)-4-chlorpyridine favoring sp^3^ hydroxylation (^**4XAlk**^**PL**_**4R-py**_^***A***^/^**4XAlk**^**PL**_**4R-py**_^***B***^: 53/47 vs ^**4XAlk**^**PL**_**4R-py**_^***A***^/^**4XAlk**^**PL**_**4R-py**_^***B***^: 51/49 for unsubstituted DG, see entries 8 and 12 in Figure 11).

The hydroxylation of isovalerophenone allowed us to study the competition between the sp^3^ and sp^2^ C–H bonds (i.e., hydroxylation of isomer A or isomer B) and the competition between β and γ hydroxylation within the sp^3^ C–H bonds of the isomer A (see entries 13–15 in Figure 11). When compared with propiophenone, we observed that the hydroxylation of the isovalerophenone system led to similar sp^3^/sp^2^ ratios (^**4XAlk**^**PL**_**4R-py**_^***A***^/^**4XAlk**^**PL**_**4R-py**_^***B***^: 25/75, ^**4XAlk**^**PL**_**4R-py**_^***A***^/^**4XAlk**^**PL**_**4R-py**_^***B***^: 25/75 vs ^**4XAlk**^**PL**_**4R-py**_^***A***^/^**4XAlk**^**PL**_**4R-py**_^***B***^: 37/63 for propiophenone, see entries 3 and 13 in Figure 11). However, we observed significant differences in the ratio of β and γ hydroxylation within isomer A, with propiophenone being oxidized exclusively at the β position (β/γ: 100/0) and isovalerophenone favoring γ oxidation (β/γ: 7/93). By changing the directing group, we observed modest variations on the sp^3^/sp^2^ and β/γ ratios, with 2-(aminomethyl)-4-methoxypyridine enhancing sp^2^ hydroxylation and improving slightly the γ sp^3^ selectivity (^**4XAlk**^**PL**_**4R-py**_^***A***^/^**4XAlk**^**PL**_**4R-py**_^***B***^: 22/78, β/γ: 0/100) and 2-(aminomethyl)-4-chlorpyridine enhancing sp^3^ hydroxylation (^**4XAlk**^**PL**_**4R-py**_^***A***^/^**4XAlk**^**PL**_**4R-py**_^***B***^: 27/73, β/γ: 0/100).

## Hydroxylation of Cyclic Alkyl Aryl Ketones

**Figure 12:**
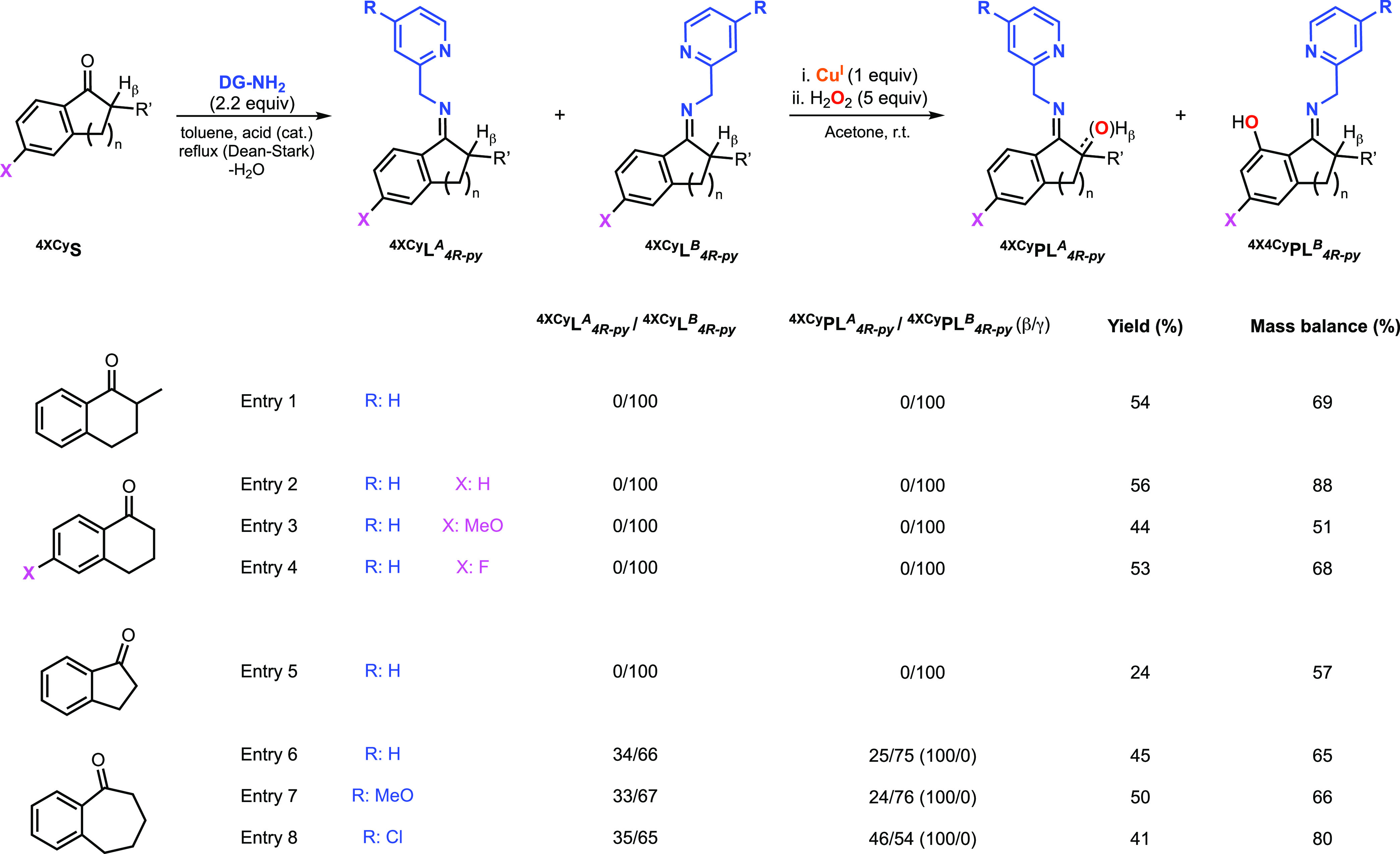
Cu-promoted hydroxylation of systems derived from cyclic alkyl aryl ketones and 2-picolylamine using H_2_O_2_ as an oxidant (see the SI for experimental details on the synthesis of substrate–ligands and hydroxylation reactions). Note: see the SI for details on the characterization and quantification of the products derived from b sp^3^ C–H hydroxylation.

In one of our previous reports, we showed that the hydroxylation of the substrate ligand derived from 2-picolylamine and 2-methyl-1-tetralone occurred selectively at the γ sp^2^ C–H bond (entry 1, Figure 12). We were puzzled by this result since we expected the formation of the imine isomer A (DG facing toward the sp^3^ C–H bond, see ^**4XCy**^**L**_**4R-py**_^***A***^ in Figure 12), which would undergo selective β sp^3^ hydroxylation like the cyclohexyl phenyl ketone system (see entry 16 in Figure 11). For this paper, we analyzed the 2-methyl-1-tetralone system again, and we observed that the installation of the 2-picolylamine led to the selective formation of the imine isomer B, which explained our prior hydroxylation results (see NMR analysis in the SI). We hypothesized that the rigidity of the cyclic substrate might preclude the formation of the imine isomer A. To test our hypothesis, we carried out the hydroxylation of a series of cyclic alkyl aryl ketones (Figure 12). In fact, the imine substrate–ligands derived from substituted tetralones (entries 1–4 in Figure 12) and 1-indanone (entry 5 in Figure 12) were all found to produce only the B isomer in solution. Consequently, the hydroxylation of these substrate–ligands selectively produced the oxidation products derived from γ sp^2^ C–H functionalization.

Conversely, the substrate–ligands derived from 1-benzosuberone produce two imine isomers (entries 6 to 8 in Figure 12). For all of the DGs tested, we observed similar imine isomer ratios (^**4XCy**^**L**_**4R-py**_^***A***^/^**4XCy**^**L**_**4R-py**_^***B***^ ∼ 35:65), but the ration of sp^3^/sp^2^ varied. While 2-picolylamine and 2-(aminomethyl)-4-methoxypyridine favored sp^2^ hydroxylation (^**4XCy**^**PL**_**4R-py**_^***A***^/^**4XCy**^**PL**_**4R-py**_^***B***^: 25/75 and 24/76, respectively), the use of 2-(aminomethyl)-4-chloropyridine allowed for reaching higher sp^3^ hydroxylation yields (^**4XCy**^**PL**_**4R-py**_^***A***^/^**4XCy**^**PL**_**4R-py**_^***B***^: 46/54). These findings are aligned with the selectivity results obtained for all of the acyclic alkyl aryl ketones analyzed (propiophenones, aryl-substituted 2-phenylacetophenones, and isovalerophenone), in which the utilization of 2-(aminomethyl)-4-methoxypyridine enhanced sp^2^ hydroxylation, while 2-(aminomethyl)-4-methoxypyridine enhanced sp^2^ hydroxylation, suggesting that electron-rich CuOOH cores are prone to oxidize sp^2^ C–H bonds and electron-poor ones prefer sp^3^ C–H bonds.

## Conclusions

To the best of our knowledge, this is the first report in which the regioselectivity of metal-promoted functionalization of C–H bonds using directing groups can be controlled by tuning the electrophilicity of the metal species involved in substrate oxidation. As we have shown, the ability of the imine substrate–ligands derived from unsymmetrical benzophenones to isomerize before the rate-determining step of the reaction allowed for the formation of an electrophilic oxidant (CuOOH core), which can selectively carry out the hydroxylation of the electron-rich ring. The electrophilicity of the reactive copper(II)-hydroperoxo intermediate (which can be benchmarked by measuring the redox potential of the cuprous complexes) can be further modulated by introducing electron-donating (MeO) or electron-withdrawing (Cl, CF_3_) substituents at the 4-position of the pyridine of the directing group, leading to remarkable regioselectivities (up to 91:9). These findings challenge our initial hypothesis (and the current paradigm in metal-promoted C–H functionalization reactions with imine directing groups), in which the regioselectivity is exclusively determined by the substrate/directing group composition (the *E*/*Z* ratio of the imine substrate–ligand) and not by the reactivity of the metal-based intermediates.

Based on the findings on Cu-directed hydroxylation of unsymmetrical benzophenones, we expanded this approach to substrate–ligands derived from unsymmetrical alkyl aryl ketones, and we observed that, like in unsymmetrical benzophenones, the regioselectivity (sp^3^ vs sp^2^) did not only depend on the ratio of the imine substrate–ligands but also on the electronics of the substrate (e.g., inclusion of MeO groups on the aryl substituent enhanced sp^2^ hydroxylation) and the electronics of the directing group (e.g., use of electron-poor directing groups enhanced sp^3^ hydroxylation). We believe that the results presented in this article will not only provide novel synthetic tools for the selective functionalization of complex molecules using cheap reagents under mild conditions (Cu, H_2_O_2_ at room temperature) but also a better understanding of the reaction mechanisms by which Cu-dependent monooxygenase and peroxygenase metalloenzymes (e.g., lytic polysaccharide monooxygenases^16,33−35^) and mononuclear Cu/O_2_ synthetic inorganic complexes^36−38^ perform selective C–H oxidations.

## Experimental Section

### Materials

All reagents and solvents were purchased at the highest level of purity and used as received except as noted. Solvents were purified and dried by passing through an activated alumina purification system (mBRAUN SPS) or by conventional distillation techniques.

### Physical Methods

The synthesis of copper complexes and preparation of some NMR samples were carried out under anaerobic conditions in an mBRAUN MB-Unilab Pro SP Glovebox system. All NMR experiments were collected at 300 K on either a two-channel Bruker Avance III NMR instrument equipped with a Broad Band Inverse (BBI) probe or a Bruker NEO 500 NMR spectrometer equipped with a multinuclear BBO Prodigy cryoprobe. Both instruments operate at 500 MHz for ^1^H (125.7 MHz for ^13^C{ ^1^H}). The ^1^H NMR spectra are referenced to residual protio solvents (7.26 ppm for CDCl_3_ and 1.94 ppm for CD_3_CN), and the ^13^C{^1^H} NMR spectra are referenced to CDCl_3_ (77.2 ppm). Structural assignments were made with additional information from COSY, NOESY, HMBC, and HSQC experiments. ESI-MS high-resolution mass spectrometry was performed on a Thermo Scientific Exactive Plus EMR Orbitrap Mass Spectrometer in the Department of Chemistry at Carnegie Mellon University. Electrochemical measurements were carried out on a model 620E Electrochemical Workstation (CH Instruments) using a glassy carbon working electrode (4 mm diameter), a Pt wire as the counter electrode, and Ag/AgNO_3_ (0.01 M in CH_3_CN) as the reference electrode. The working electrode was polished before each measurement with alumina polishing powder onto a wet polishing cloth. All measurements were made in CH_3_CN with 1 mM Cu complex and NBu_4_PF_6_ as the electrolyte (100 mM) at room temperature.

### General Procedure for the Synthesis of Imine Substrate–Ligands

In an oven-dried flask, 2-picolylamine (2.2 equiv) was added to the benzophenone substrate (9.85 mmol) and *p*-toluenesulfonic acid monohydrate (cat. 20 mg) in toluene (50 mL). The reaction mixture was refluxed under argon with a Dean–Stark apparatus until imine formation was completed. The reaction was cooled to room temperature and diluted with diethyl ether (30 mL). The organic layer was washed with saturated ammonia chloride (20 mL × 2), saturated aqueous sodium bicarbonate (20 mL), and brine (20 mL) and dried with sodium sulfate. The final product was isolated under vacuum. The purity of the resulting imine substrate–ligands was analyzed by ^1^H NMR by adding a known amount of an internal standard (1,3,5-trimethoxybenzene or 1,2-dichloroethane).

### Standard Procedure for the Hydroxylation of the Imine Substrate–Ligands

In the glovebox, 4 mL of acetone was added to a 20 mL vial containing 0.159 mmol of the imine substrate–ligand equipped with a stir bar. To the solution, 0.159 mmol of [Cu^I^(CH_3_CN)_4_](PF_6_) was added and allowed to react. The solution mixture was taken out of the glovebox, and 5 equiv of 30% H_2_O_2_ were added. After 30 min, the reaction was quenched using Na_2_EDTA (50 mL, pH = 4). The resulting mixture was extracted with EtOAc (50 mL × 3). The organic phases were separated, combined, dried over MgSO_4_, filtered, and dried under vacuum. The reaction products were dissolved in 1.4 mL of CDCl_3_ solution containing 27.1 mg of 1,3,5-trimethoxybenzene (internal standard). The reaction products were quantified by ^1^H NMR using integration signals that correspond to the starting material and products with the integration signal of the internal standard.

### Standard Procedure for the Removal of the Directing Group

After hydroxylation, the products were dissolved in a round-bottom flask with 50 mL of EtOAc, and 100 mL of 1 M HCl was added. The resulting mixture was stirred at room temperature for 30 min and was extracted with EtOAc (50 mL × 2). The organic phases were separated, combined, dried over MgSO_4_, filtered, and dried under vacuum. The reaction products were dissolved in 1.4 mL of CDCl_3_ solution containing 27.1 mg of 1,3,5-trimethoxybenzene (internal standard). The reaction products were quantified by ^1^H NMR using integration signals that correspond to the starting material and products with the integration signal of the internal standard.

### Calculation of the Ratio of Isomers for the Imine Substrate–Ligands, Hydroxylation Products, and Cleaved Hydroxylation Products

^1^H NMR, ^13^C NMR, COSY, and NOESY measurements were carried out to characterize the two isomers formed in the synthesis of the imine substrate–ligands. The ratio of the isomers was calculated using the average of the integration of CH_2_ peaks (between 4.5 and 5.0 ppm) and CH peaks (between 7.0 and 7.5 ppm). ^1^H NMR measurements were carried out to characterize the products derived from the hydroxylation of the imine substrate–ligands. The ratio of the products is calculated using the average of the integration of CH_2_ peaks (between 4.5 and 5.0 ppm) and CH peaks (between 6.2 and 7.0 ppm). For each of the systems, at least two hydroxylation reactions were analyzed, and only slight variations in the reaction yield, mass balance (Note: the mass balance includes the hydroxylation yields and starting material unreacted), and product ratio were observed. ^1^H NMR measurements were carried out to characterize the products derived from the cleavage of the products derived from the hydroxylation of the imine substrate–ligands. The ratio of the cleaved products was calculated using the average of the integration of CH peaks (between 6.2 and 7.0 ppm). We noticed that these ratios slightly differ from the ratios obtained in the noncleaved hydroxylation products (5–10% difference). We believe that this is due to the loss of some of the hydroxylation products during the cleavage step (Note: the highest variations are observed for the systems containing a MeO substituent).

### Electrochemical Measurements

Three milliliters of a CH_3_CN solution containing 1 mM Cu complex (the cuprous complexes were generated in situ via addition of 1 equiv of [Cu^I^(CH_3_CN)_4_](PF_6_) to the corresponding imine substrate–ligand) and NBu_4_PF_6_ (100 mM) were prepared in the glovebox and were transferred to an electrochemical cell outside the glovebox, which was purged with Ar for 10 min (Note: a conventional three-electrode cell was used with a glassy carbon working electrode, an Ag/AgNO_3_ (0.01 M), and a platinum wire as the counter electrode). The potentials were measured with respect to the Ag/AgNO_3_ reference electrode and converted to Fc^0/+^ (Fc^0/+^ potential measured under the same experimental conditions). Cyclic voltammograms were obtained at a scan rate of 0.1 V/s.

## Data Availability

The data underlying this study are available in the published article and its Supporting Information

## Supporting Information Available

Experimental details, including characterization data, spectra, and electrochemical measurements (PDF)
