# Supplementary material for: Regioselective Hydroxylation of Unsymmetrical Ketones Using Cu, H2O2, and Imine Directing Groups via Formation of an Electrophilic Cupric Hydroperoxide Core
Source: J Org Chem. 2024 Feb 7;89(4):2622–36. doi: 10.1021/acs.joc.3c02647 (PMC10877615; doi:10.1021/acs.joc.3c02647)
Supplement: Supplementary file 1 — jo3c02647_si_001.pdf [file jo3c02647_si_001.pdf]

Supporting Information

for

Regioselective hydroxylation of unsymmetrical ketones using Cu, H_2O_2 and imine directing groups via formation of an electrophilic cupric hydroperoxide core

Shuming Zhang, Sunipa Goswami, Karl H. G. Schulz, Karan Gill, Xinyi Yin, Jimin Hwang, Jasmine Wiese, Isabel Jaffer, Roberto R. Gil, and Isaac Garcia-Bosch*

[†]Department of Chemistry, Carnegie Mellon University, Pittsburgh, Pennsylvania 15213, United States

E-mail corresponding author: igarciab@andrew.cmu.edu

Contents

1. Experimental section S3

2. Description of the general protocol for the synthesis and hydroxylation of the imine substrate-ligands. S4

3. Synthesis and hydroxylation of the imine substrate-ligands S6

4. ^{1}H-NMR experiments included in Figure 6 ..... S187

5. Mass balance experiments S202

6. Electrochemistry S203

7. ^{13}C-NMR data for imine substrate-ligands S205

8. References S227

1. Experimental section

Physical methods and materials reagents: All reagents and solvents were purchased at the highest level of purity and used as received except as noted. All substrate-containing ligands were synthesized as previously reported.[1] Solvents were purified and dried by passing through an activated alumina purification system (mBRAUN SPS) or by conventional distillation techniques.

Glovebox: The synthesis of copper complexes and preparation of some NMR samples was carried out under anaerobic conditions in an mBRAUN MB-Unilab Pro SP Glovebox system.

All NMR experiments were collected at 300 K on either a two-channel Bruker Avance III NMR instrument equipped with a Broad Band Inverse (BBI) probe, or a Bruker NEO 500 NMR spectrometer equipped with the multinuclear BBO Prodigy cryoprobe. Both instruments operate at 500 MHz for ^{1}H (125.7 MHz for $^{13}C\{^{1}H\}$). The 1H NMR spectra are referenced to residual protio solvents (7.26 ppm for $CDCl_3$ and 1.94 ppm for CD_3CN) and the $^{13}C\{^{1}H\}$ NMR spectra are referenced to $CDCl_3$ (77.2 ppm).

ESI-MS: High resolution mass spectrometry was performed on Thermo Scientific Exactive Plus EMR Orbitrap Mass Spectrometer in the Department of Chemistry at Carnegie Mellon University.

Electrochemical measurements were carried out on a model 620E Electrochemical Workstation (CH Instruments) using a glassy carbon working electrode (4 mm diameter), Pt wire as counter electrode, and $Ag/AgNO_3$ (0.01 M in CH_3CN) as reference electrode The surface area and geometry of working electrode. The working electrode was polished with alumina polishing powder onto a wet polishing cloth. All measurements were made in CH_3CN with 1 mM Cu complex and NBu_4PF_6 as electrolyte (100 mM) and at room temperature.

2. Description of the general protocol for the synthesis and hydroxylation of the imine substrate-ligands.

2.1 General Procedure for imine substrate-ligand synthesis

In an oven dried flask, 2-picolylamine (2.2 equiv.) was added to the benzophenone substrate (9.85 mmol) and p-toluenesulfonic acid monohydrate (cat. 20 mg) in toluene (50 mL). The reaction mixture was refluxed under argon with a Dean-Stark apparatus until imine formation was completed. The reaction was cooled to room temperature and diluted with diethyl ether (30 mL). The organic layer was washed with saturated ammonia chloride (20 mL x 2), saturated aqueous sodium bicarbonate (20 mL), brine (20 mL), and dried with sodium sulfate. The final product was isolated under vacuum. The purity of the resulting imine substrate-ligands was analyzed by ^{1}H-NMR by adding a known amount of internal standard (1,3,5-trimethoxybenzene or 1,2-dichloroethane).

2.2 Standard procedure for imine substrate-ligand hydroxylation

In the glovebox, 4 mL of acetone were added to a 20-mL vial containing 0.159 mmols of the imine substrate-ligand equipped with a stir bar. To the solution, 0.159 mmol of $[Cu^I(CH_3CN)_4](PF_6)$ was added and allowed to react. The solution mixture was taken out of the glovebox and 5 equiv of 30% H_2O_2 were added. After 30 min, the reaction was quenched using Na_2EDTA (50 mL, pH = 4). The resulting mixture was extracted with EtOAc (50 mL X 3). The organic phases were separated, combined, dried over $MgSO_4$, filtered, and dried under vacuum. The reaction products were dissolved in 1.4 mL of $CDCl_3$ solution containing 27.1 mg of 1,3,5-trimethoxybenzene (internal standard). The reaction products were quantified by ^{1}H-NMR using integration signals that correspond to the starting material and products with the integration signal of the internal standard.

2.3 Standard procedure for cleavage of directing group:

After hydroxylation, the products were dissolved in a round bottom flask with 50 mL EtOAc and 100 mL 1M HCl were added. The resulting mixture was stirred for 30 min at room temperature. The resulting mixture was extracted with EtOAc (50 mL X 2). The organic phases were separated, combined, dried over $MgSO_4$, filtered, and dried under vacuum. The reaction products were dissolved in 1.4 mL of $CDCl_3$ solution containing 27.1 mg of 1,3,5-trimethoxybenzene (internal standard). The reaction products were quantified by ^{1}H-NMR using integration signals that correspond to the starting material and products with the integration signal of the internal standard.

2.4 Calculation of the ratio of isomers for the imine substrate-ligands, hydroxylation products and cleaved hydroxylation products.

^{1}H-NMR, ^{13}C-NMR, COSY and NOESY measurements were carried out to characterize the two isomers formed in the synthesis of the imine substrate-ligands. The ratio of the isomers is calculated using the average of the integration of CH_2 peaks (between 4.5 and 5.0 ppm) and CH peaks (between 7.0 and 7.5 ppm).

^{1}H-NMR measurements were carried out to characterize the products derived from the hydroxylation of the imine substrate-ligands. The ratio of the products is calculated using the average of the integration of CH_2 peaks (between 4.5 and 5.0 ppm) and CH peaks (between 6.2 and 7.0 ppm). For each of the systems, at least two hydroxylation reactions were analyzed and only slight variations in the reaction yield, mass balance (note: the mass balance includes the hydroxylation yields and starting material unreacted) and products ratio were observed.

^{1}H-NMR measurements were carried out to characterize the products derived from the cleavage of the products derived from the hydroxylation of the imine substrate-ligands. The ratio of the cleaved products is calculated using the average of the integration of CH peaks (between 6.2 and 7.0 ppm). We noticed that these ratios slightly differ from the ratios obtained in the non-cleaved hydroxylation products (5-10% difference). We believe that this is due to the loss of some of the hydroxylation products during the cleavage step (note: the highest variations are observed for the systems containing a MeO substituent).

3. Synthesis and hydroxylation of the imine substrate-ligands

3.1 ^{2MeO}S and 2-picolyamine

Synthesis of ^{2MeO}L

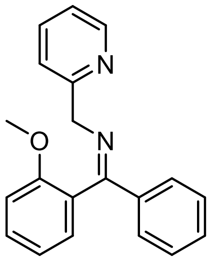

^{2MeO}L^{Z}

In an oven dried flask, 2-picolylamine (2.2 equiv. 0.33 mL) was added to 2-methoxybenzophenone (300 mg, 1.40 mmol) and p- toluenesulfonic acid monohydrate (cat. 10 mg, 4 mol%) in toluene (50 mL). The reaction mixture was refluxed under argon with a Dean-Stark apparatus until imine formation was complete (2 days). The reaction was cooled to room temperature and diluted with diethyl ether (30 mL). The organic layer was washed with saturated ammonia chloride (20 mL x 2), saturated aqueous sodium bicarbonate (20 mL), brine (20 mL), and dried with magnesium sulfate. The final product was isolated as a brown solid (94% yield, 397.4 mg, 95% pure). ^{1}H-NMR (500 MHz, $CDCl_3$): δ 8.48 (d, 1H), 7.74 (d, 2H), 7.68 (m, 2H), 7.47-7.34 (m, 4H), 7.25 (m, 1H), 7.03 (m, 3H), 4.73 (q, 2H), 3.79 (s, 3H). HRMS (ESI) m/z: [M + Na]+ Calcd for C20H18N2ONa 302.3770, found 303.1482.

Hydroxylation of ^{2MeO}L

The reaction was carried out on 0.159 mmol scale using 50.5 mg of the imine according to the Standard Procedure. The reaction products were quantified using 0.159 mmol of 1,3,5-trimethoxybenzene (int. std.). (64% yield). The identity of the hydroxylation products was confirmed by ^{1}H-NMR.

^{1}H-NMR spectra of [2MeO]L

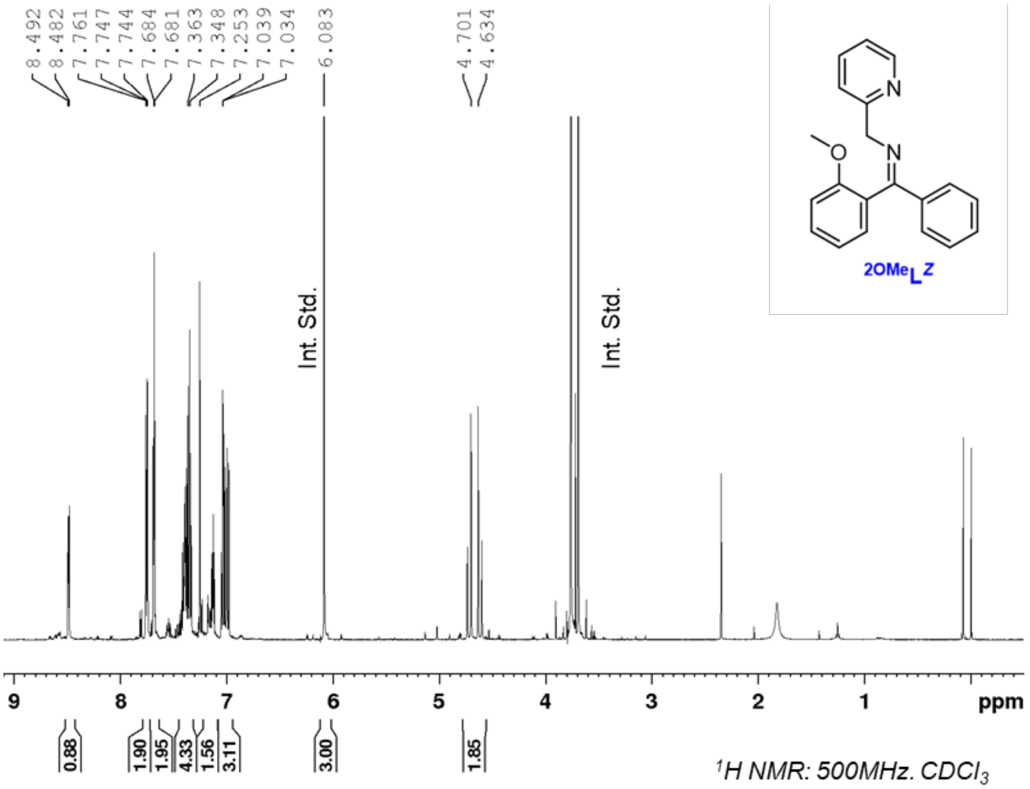

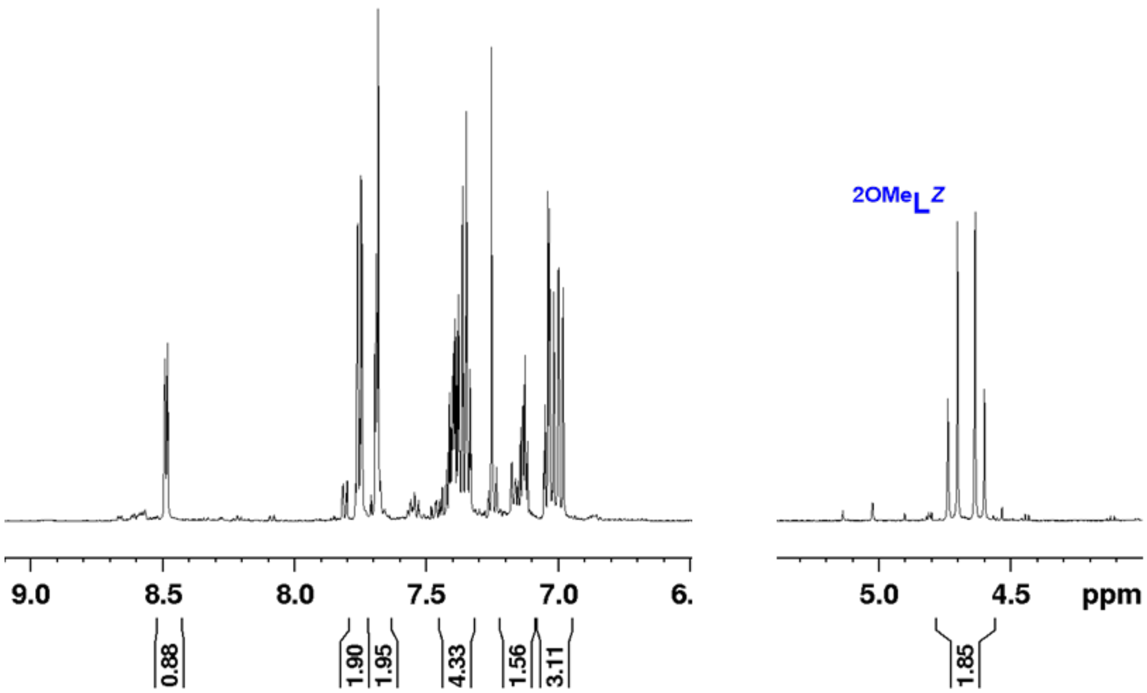

Figure S1. ^{1}H-NMR spectra of **[2MeO]L**. Note: Only one of the imine isomers is formed.

^{1}H-NMR spectra for the hydroxylation of ^{2MeO}L

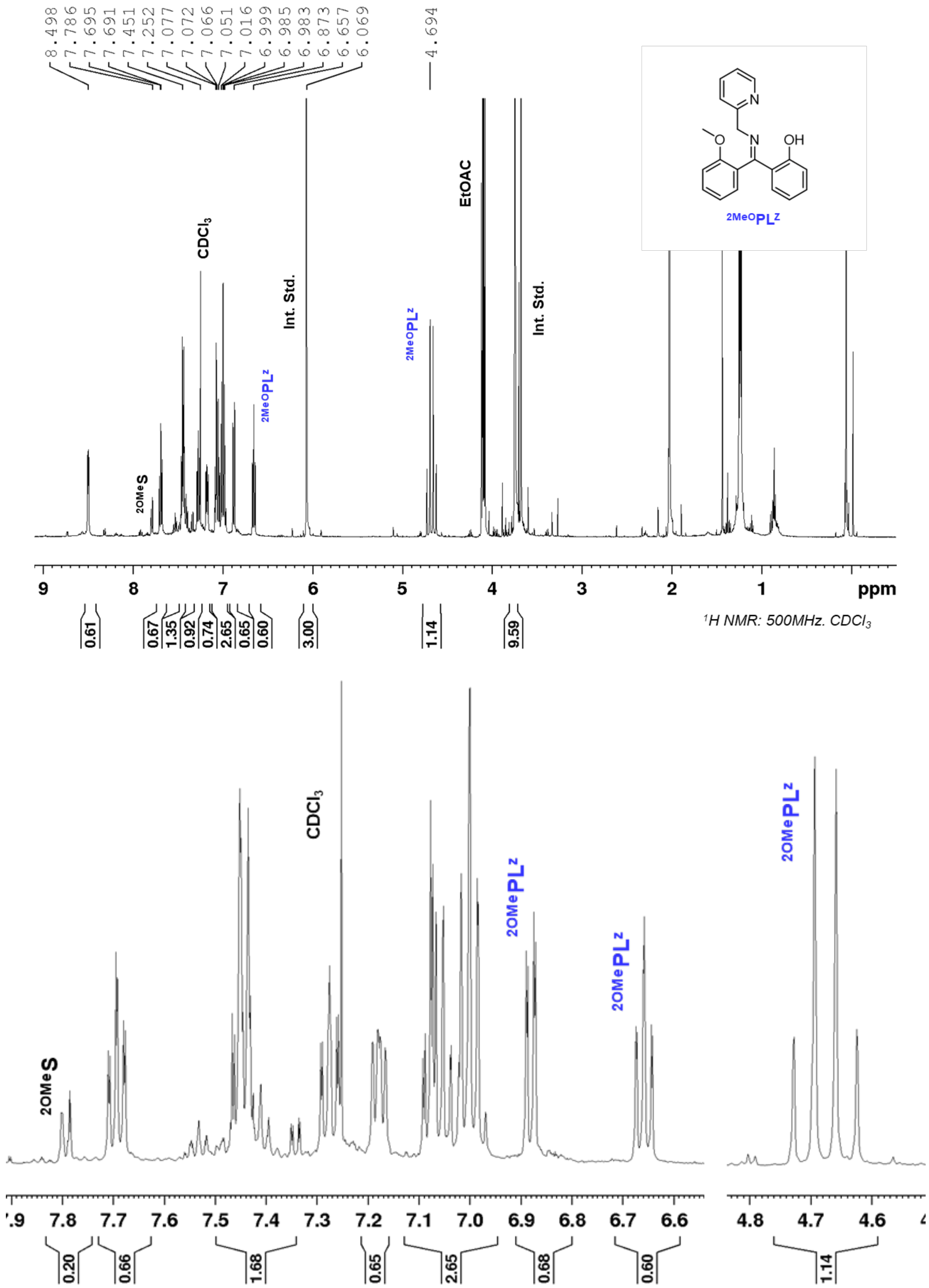

Figure S2. ^{1}H NMR spectra for the hydroxylation of ^{2MeO}**L**. Note: Only one hydroxylation product is formed.

3.2 ^{2Me}S and 2-picolyamine

Synthesis of ^{2Me}L

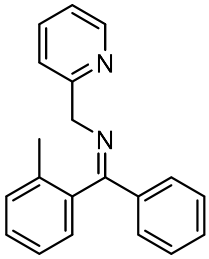

^{2Me}L *Z*

In an oven dried flask, 2-picolylamine (2.2 equiv., 0.90 mL) was added to 2-methybenzophenone (0.75 g, 3.80 mmol) and p- toluenesulfonic acid monohydrate (cat. 10 mg, 1.5 mol%) in toluene (50 mL). The reaction mixture was refluxed under argon with a Dean-Stark apparatus until imine formation was complete (6 days). The reaction was cooled to room temperature and diluted with diethyl ether (30 mL). The organic layer was washed with saturated ammonia chloride (20 mL x 2), saturated aqueous sodium bicarbonate (20 mL), brine (20 mL), and dried with magnesium sulfate. The final product was isolated as a brown solid (94% yield, 1.02 g, 80% pure). ^{1}H-NMR (500 MHz, $CDCl_3$): δ 8.43 (d, 1H), 7.71 (td, 1H), 7.63 (m, 2H), 7.51-7.52 (d, 1H), 7.37-7.43 (m, 1H), 7.34-7.38 (m, 4H), 7.25-7.31 (td, 1H), 7.16-7.18 (m, 1H), 7.07-7.08 (d, 1H), 4.42-4.47 (d, 2H), 2.03 (s, 3H). 13C{1H} NMR (500 MHz, CD_3CN): δ 170.1, 161.2, 149.9, 139.9, 137.5, 135.9, 131.26, 129.6, 129.3, 128.1, 127.1, 122.9, 122.8, 60.1, 19.5. HRMS (ESI) m/z: [M + Na]+ Calcd for C20H18N2Na 286.3780, found 287.1540.Note: this imine substrate-ligand was previously synthesized by our group,[2] but the NMR analysis of the imine substrate-ligands and oxidation was carried out in CD_3CN.

Hydroxylation of ^{2Me}L

The reaction was carried out on 0.159 mmol scale using 56.8 mg of the imine according to the Standard Procedure. The reaction products were quantified using 0.159 mmol of 1,3,5-trimethoxybenzene (int. std.). (58% yield). The identity of the hydroxylation products was confirmed by ^{1}H-NMR.

^{1}H-NMR spectra of ***2MeL***

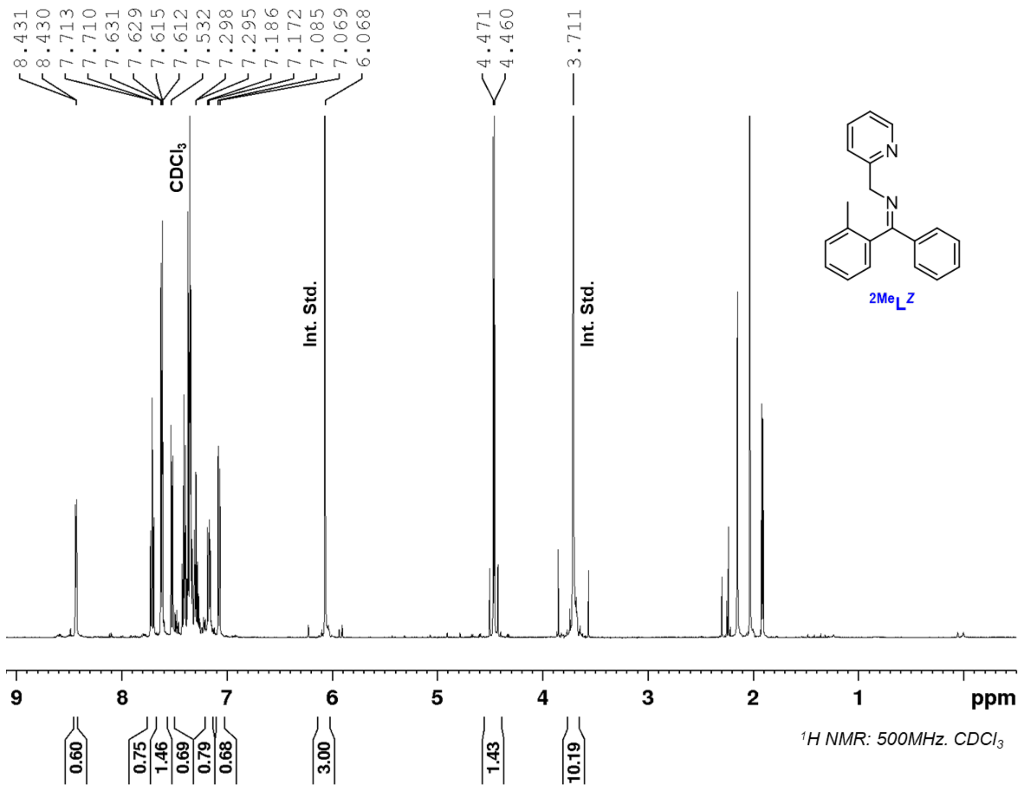

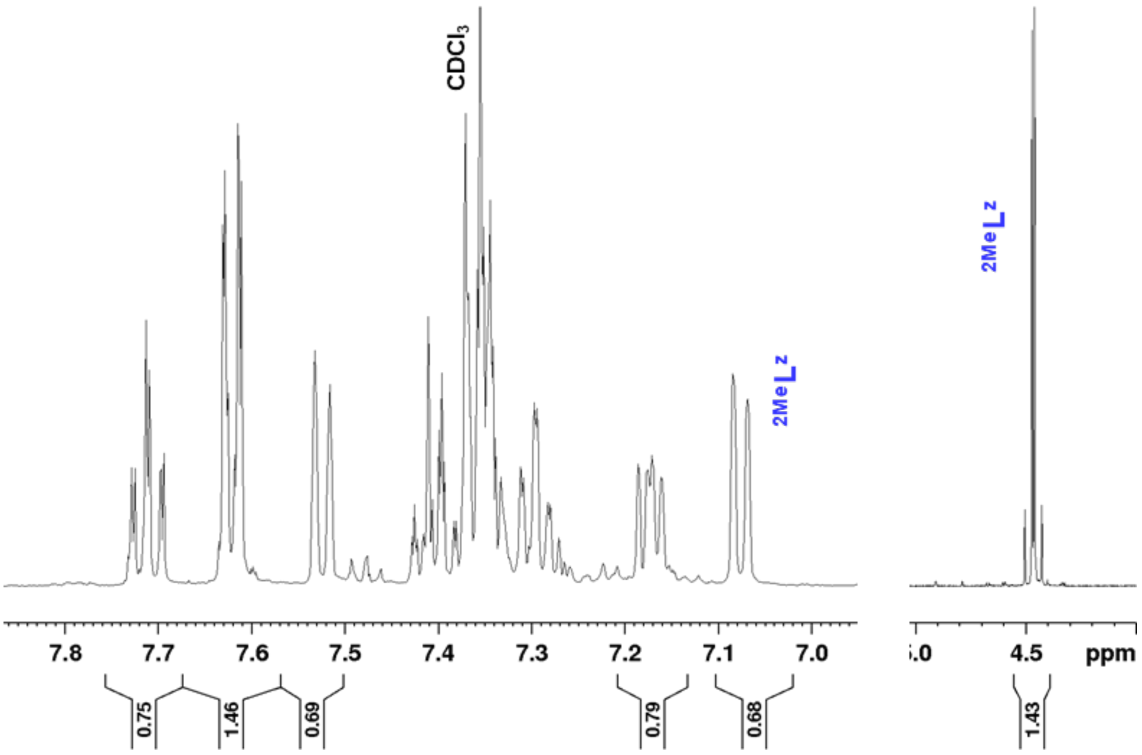

Figure S3. ^{1}H-NMR spectra of **2MeL**. Note: Only one of the imine isomers is formed.

^{1}H-NMR spectra for the hydroxylation of ^{2Me}L

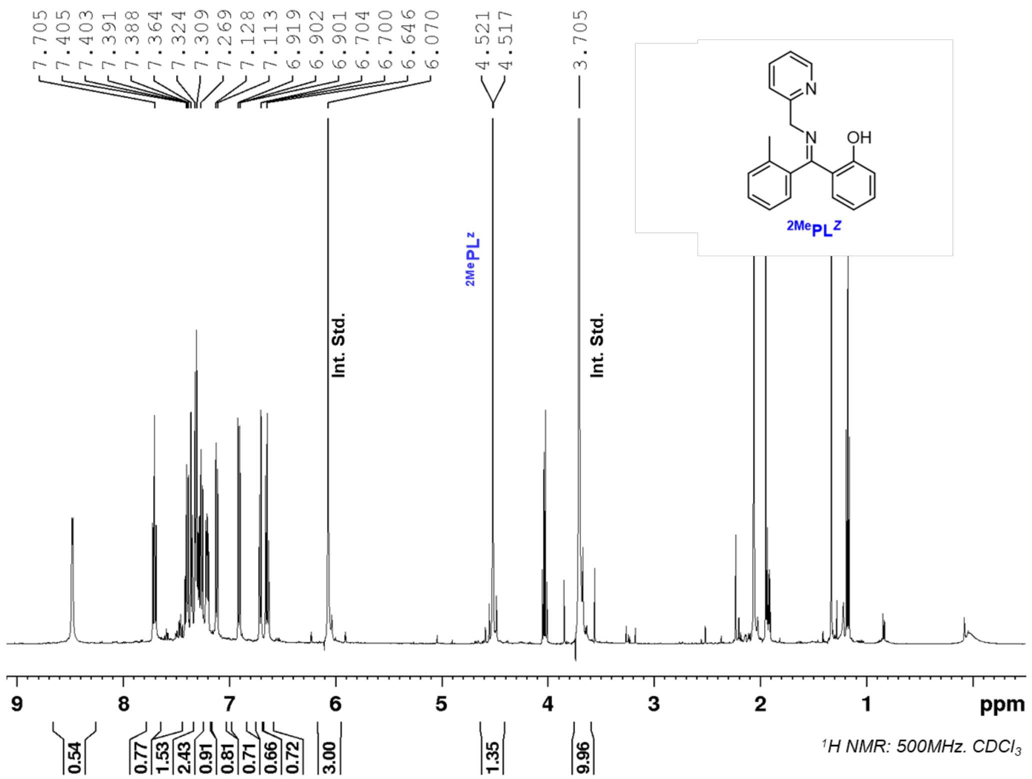

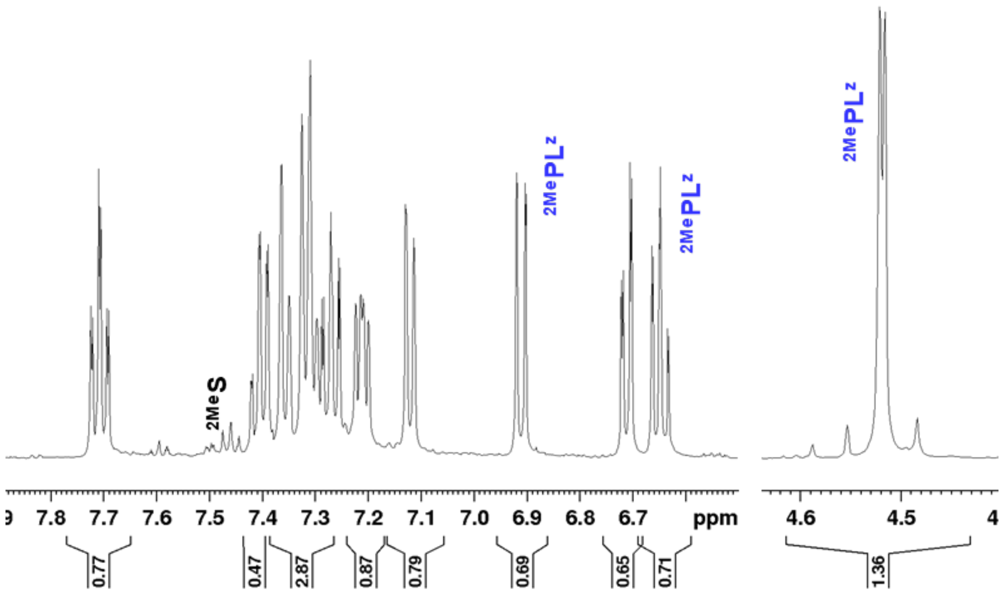

Figure S4. ^{1}H NMR spectra for the hydroxylation of ^{2Me}**L**. Note: Only one hydroxylation product is formed.

3.3 ^{2F}S and 2-picolyamine

Synthesis of ^{2F}L

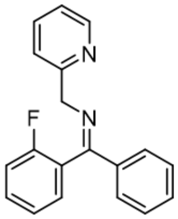

^{2F}**L**^{Z}

In an oven dried flask, 2-picolylamine (2.2 equiv., 1.16 mL) was added to 2-fluorobenzophenone (0.90 g, 4.90 mmol) and p- toluenesulfonic acid monohydrate (cat. 10 mg, 1.2 mol%) in toluene (50 mL). The reaction mixture was refluxed under argon with a Dean-Stark apparatus until imine formation was complete (7 days). The reaction was cooled to room temperature and diluted with diethyl ether (30 mL). The organic layer was washed with saturated ammonia chloride (20 mL x 2), saturated aqueous sodium bicarbonate (20 mL), brine (20 mL), and dried with magnesium sulfate. The final product was isolated as a brown solid (94% yield, 1.3 g, 95% pure). ^{1}H-NMR (500 MHz, $CDCl_3$): δ 8.45 (d, 1H), 7.71 (td, 1H), 7.66 (m, 2H), 7.53 (m, 2H), 7.43 (m, 1H), 7.39 (m, 2H), 7.33-7.22 (m, 3H), 7.17 (m, 1H), 4.58 (s, 2H). 13C{1H} NMR (500 MHz, CD_3CN): δ 164.7, 160.8, 149.9, 139.8, 137.5, 132.2, 131.5, 130.6, 129.3, 128.6, 127.7, 125.7, 122.8, 117.0, 116.5, 60.5. HRMS (ESI) m/z: [M + Na]+ Calcd for C19H15FN2Na 290.3414, found 291.4051. Note: this imine substrate-ligand was previously synthesized by our group,[2] but the NMR analysis of the imine substrate-ligands and oxidation was carried out in CD_3CN.

Hydroxylation of ^{2F}L

The reaction was carried out on 0.159 mmol scale using 48.5 mg of the imine according to the Standard Procedure. The reaction products were quantified using 0.159 mmol of 1,3,5-trimethoxybenzene (int. std.). (45% yield). The identity of the hydroxylation products was confirmed by ^{1}H-NMR.

^{1}H-NMR spectra of [2F]L

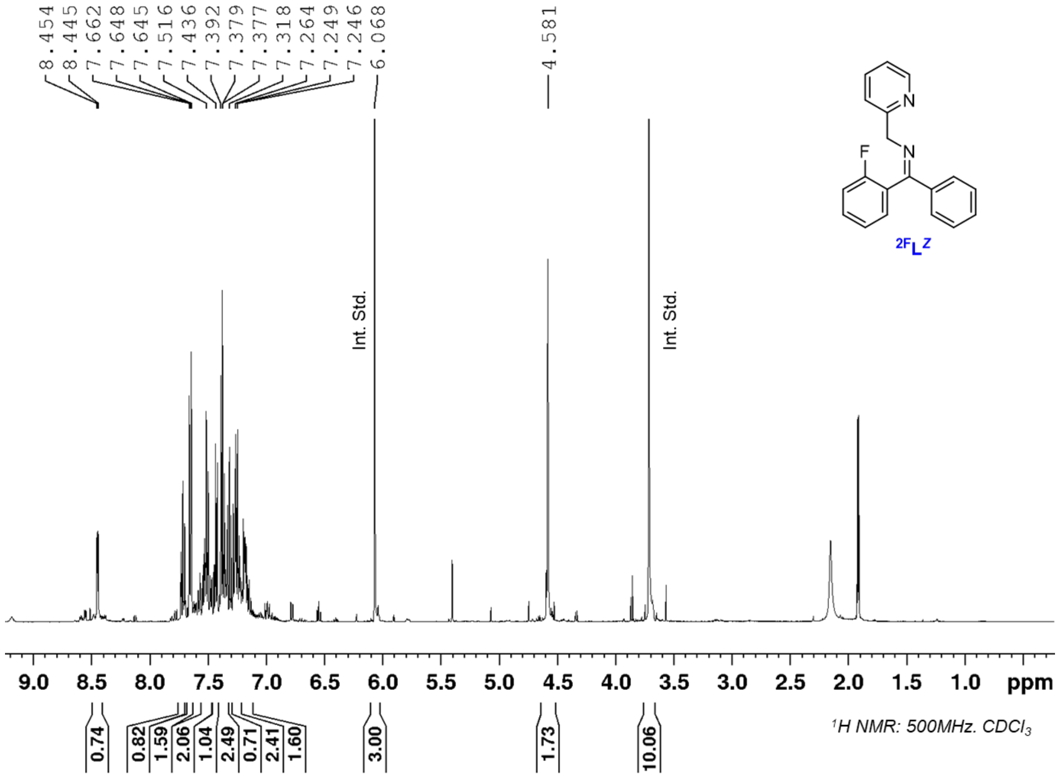

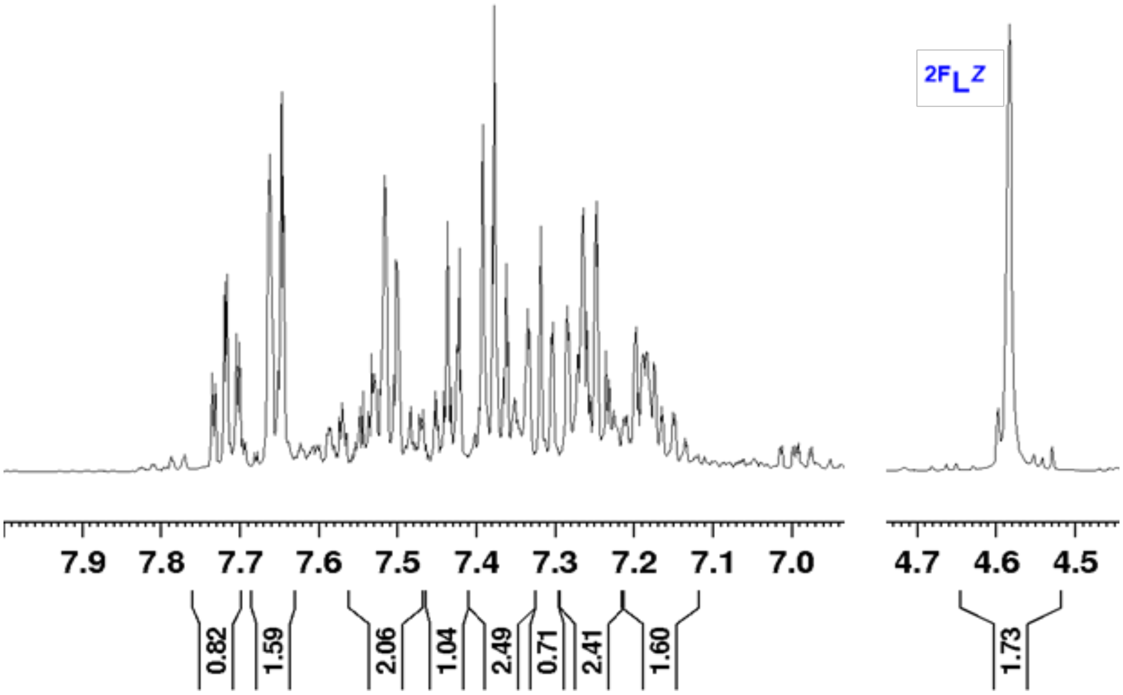

Figure S5. ^{1}H-NMR spectra of **[2F]L**. Note: Only one of the imine isomers is formed.

^{1}H-NMR spectra for the hydroxylation of ^{2F}L

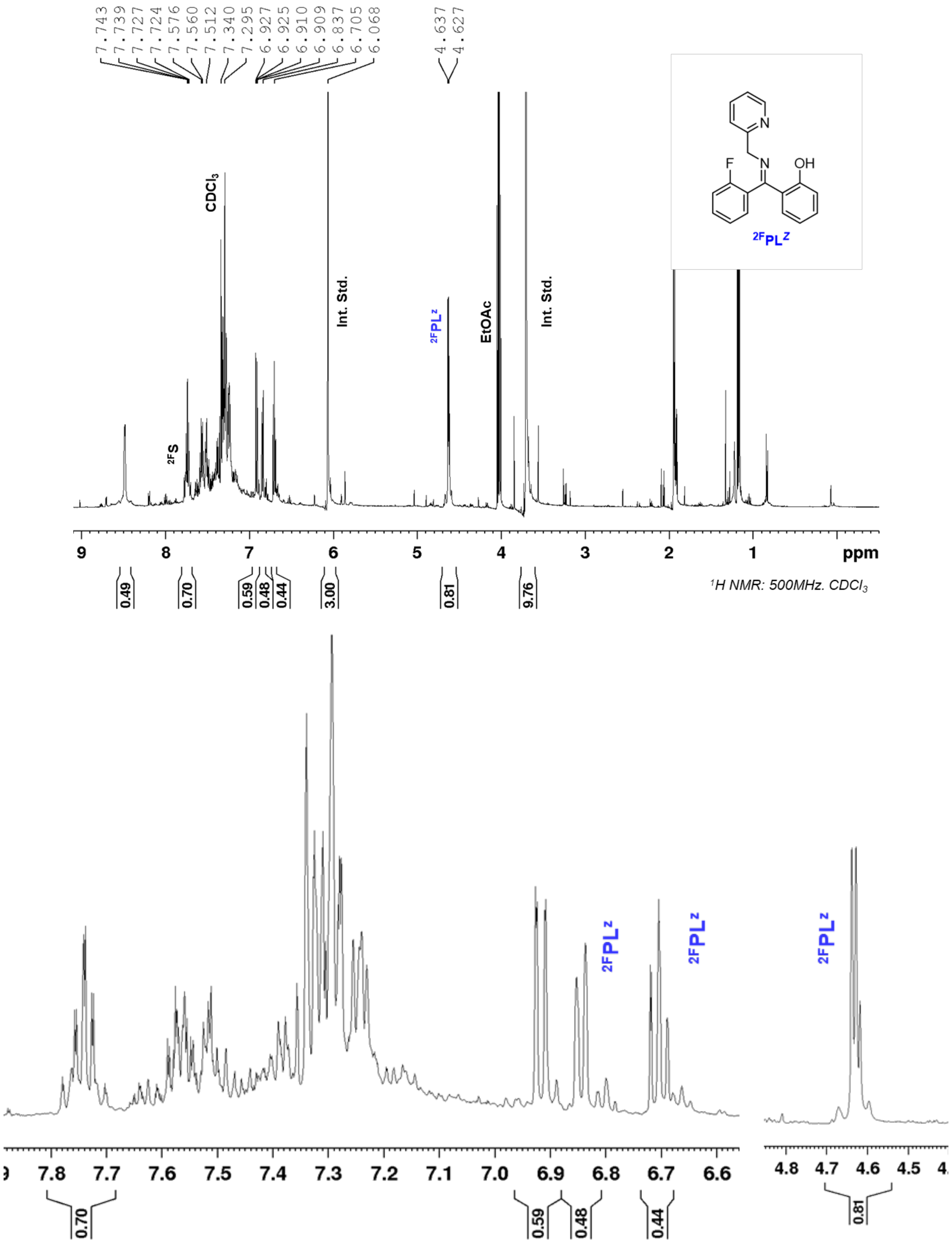

Figure S6. ^{1}H-NMR spectra for the hydroxylation of ^{2F}L. Note: only one hydroxylation product is formed.

3.4 ^{2Cl}S and 2-picolyamine

Synthesis of ^{2Cl}L

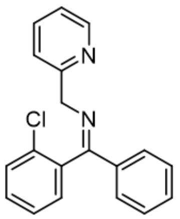

^{2Cl}L *Z*

In an oven dried flask, 2-picolylamine (2.2 equiv., 2.2 mL) was added to 2-chlorobenzophenone (2.13 g, 9.85 mmol) and p- toluenesulfonic acid monohydrate (cat. 20 mg, 1.2 mol%) in toluene (50 mL). The reaction mixture was refluxed under argon with a Dean-Stark apparatus until imine formation was complete (7 days). The reaction was cooled to room temperature and diluted with diethyl ether (30 mL). The organic layer was washed with saturated ammonia chloride (20 mL x 2), saturated aqueous sodium bicarbonate (20 mL), brine (20 mL), and dried with magnesium sulfate. The final product was isolated as a brown solid (94% yield, 2.84 g, 95% pure). ^{1}H-NMR (500 MHz, $CDCl_3$): δ 8.50 (d, 1H), 7.69 (m, 4H), 7.50 (d, 1H), 7.37 (m, 5H), 7.15 (m, 2H), 4.55 (s, 2H). 13C{1H} NMR (500 MHz, CD_3CN): δ 167.0, 160.9, 149.9, 137.5, 130.6, 130.2, 129.4, 128.6, 123.0, 122.8, 60.1. HRMS (ESI) m/z: [M + Na]+ Calcd for C19H15ClN2Na 306.7930, found 307.1031. Note: this imine substrate-ligand was previously synthesized by our group,[2] but the NMR analysis of the imine substrate-ligands and oxidation was carried out in CD_3CN.

Hydroxylation of ^{2Cl}L

The reaction was carried out on 0.159 mmol scale using 51.2 mg of the imine according to the Standard Procedure. The reaction products were quantified using 0.159 mmol of 1,3,5-trimethoxybenzene (int. std.). (41% yield). The identity of the hydroxylation products was confirmed by ^{1}H-NMR.

^{1}H-NMR spectra of ^{2Cl}L

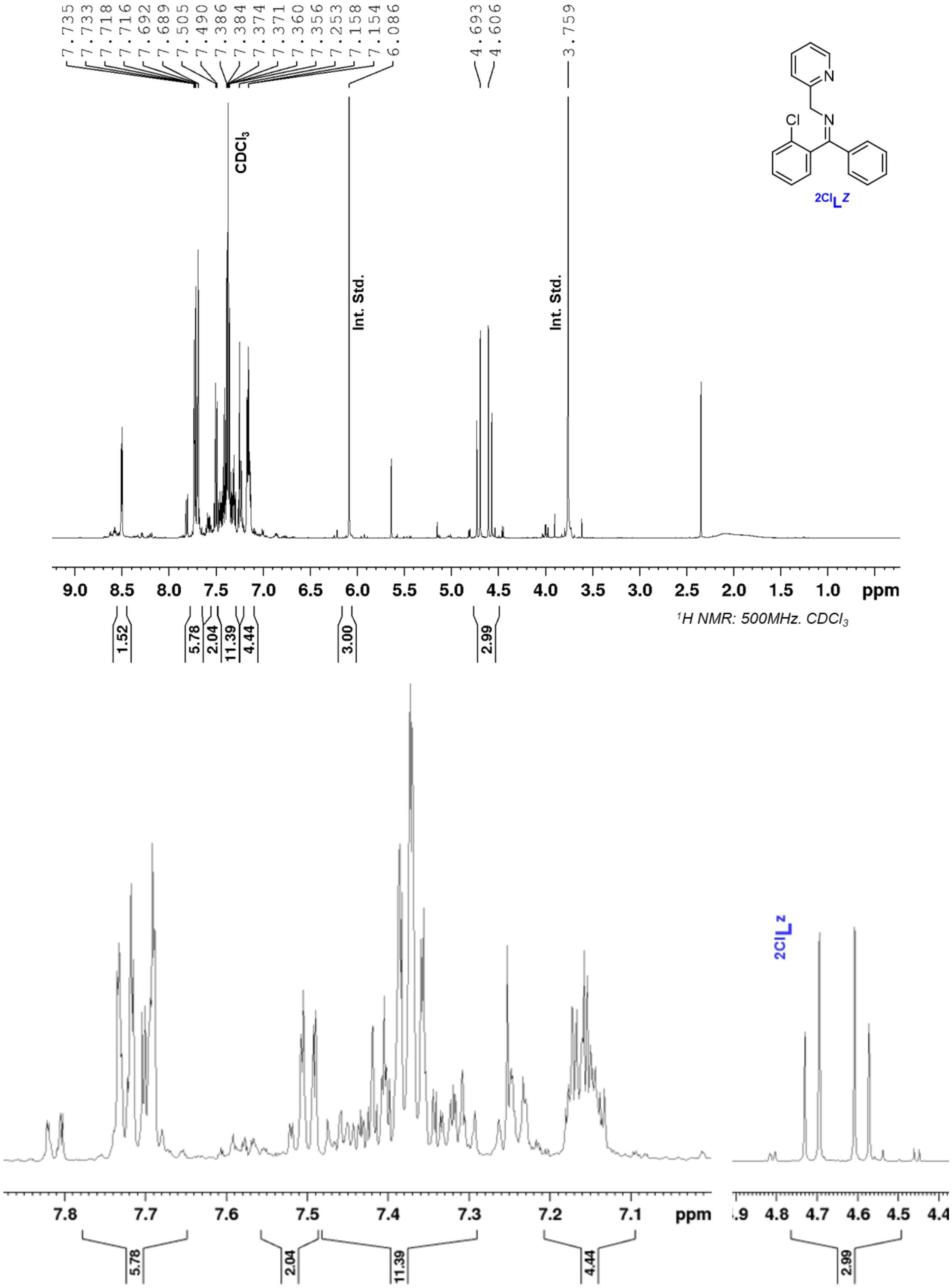

Figure S7. ^{1}H-NMR spectra of ^{2Cl}L. Note: Only one of the imine isomers is formed.

^{1}H-NMR spectra for the hydroxylation of ^{2Cl}L

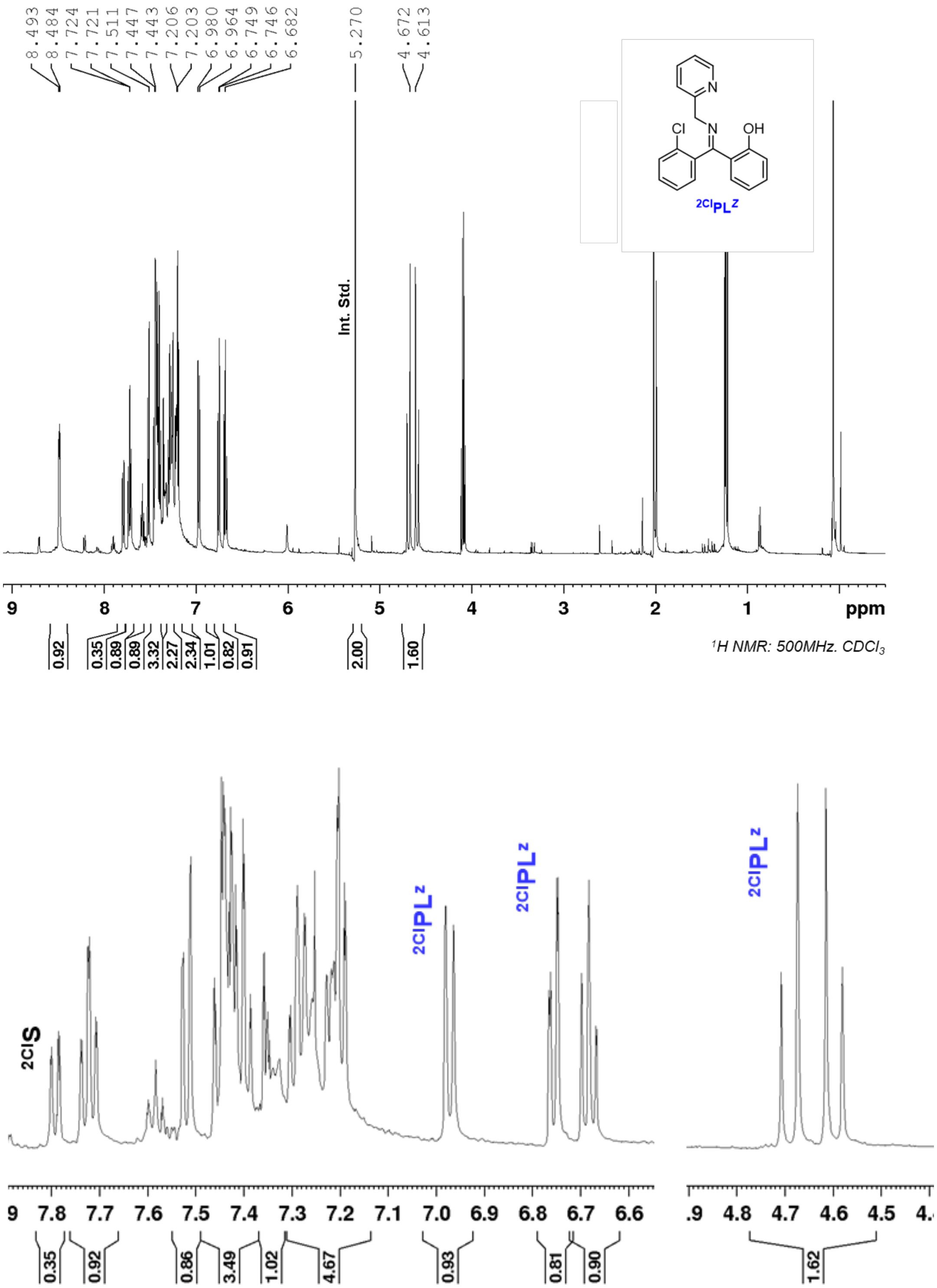

Figure S8. ^{1}H-NMR spectra for the hydroxylation of ^{2Cl}L. Note: only one hydroxylation product is formed.

3.5 ^{2Br}S and 2-picolyamine

Synthesis of ^{2Br}L

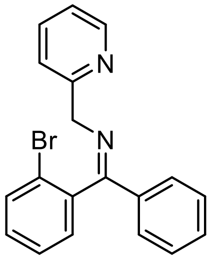

^{2Br}L Z

In an oven dried flask, 2-picolylamine (2.2 equiv., 0.90 mL) was added to 2-bromobenzophenone (750 mg, 3.80 mmol) and p- toluenesulfonic acid monohydrate (cat. 20 mg, 3mol%) in toluene (50 mL). The reaction mixture was refluxed under argon with a Dean-Stark apparatus until imine formation was complete (6 days). The reaction was cooled to room temperature and diluted with diethyl ether (30 mL). The organic layer was washed with saturated ammonia chloride (20 mL x 2), saturated aqueous sodium bicarbonate (20 mL), brine (20 mL), and dried with magnesium sulfate. The final product was isolated as a brown solid (90% yield, 1.20 g, 85% pure). ^{1}H-NMR (500 MHz, $CDCl_3$): δ 8.49 (d, 1H), 7.73 (m, 4H), 7.45-7.35 (m, 4H), 7.35-7.27 (td, 1H), 7.23 (t, 1H), 7.17-7.12 (m, 2H), 4.74-4.54 (q, 2H). 13C{1H} NMR (500 MHz, CD_3CN): δ 168.0, 160.9, 149.9, 139.0, 138.7, 137.5, 133.8, 131.5, 130.3, 129.4, 129.0, 128.7, 123.0, 122.8, 121.4, 60.1. HRMS (ESI) m/z: [M + Na]+ Calcd for C19H15BrN2Na 351.2470, found 352.0712. Note: this imine substrate-ligand was previously synthesized by our group,[2] but the NMR analysis of the imine substrate-ligands and oxidation was carried out in CD_3CN.

Hydroxylation of ^{2Br}L

The reaction was carried out on 0.159 mmol scale using 65.6 mg of the imine according to the Standard Procedure. The reaction products were quantified using 0.159 mmol of 1,3,5-trimethoxybenzene (int. std.). (40% yield). The identity of the hydroxylation products was confirmed by ^{1}H-NMR.

^{1}H-NMR spectra of ^{2Br}L

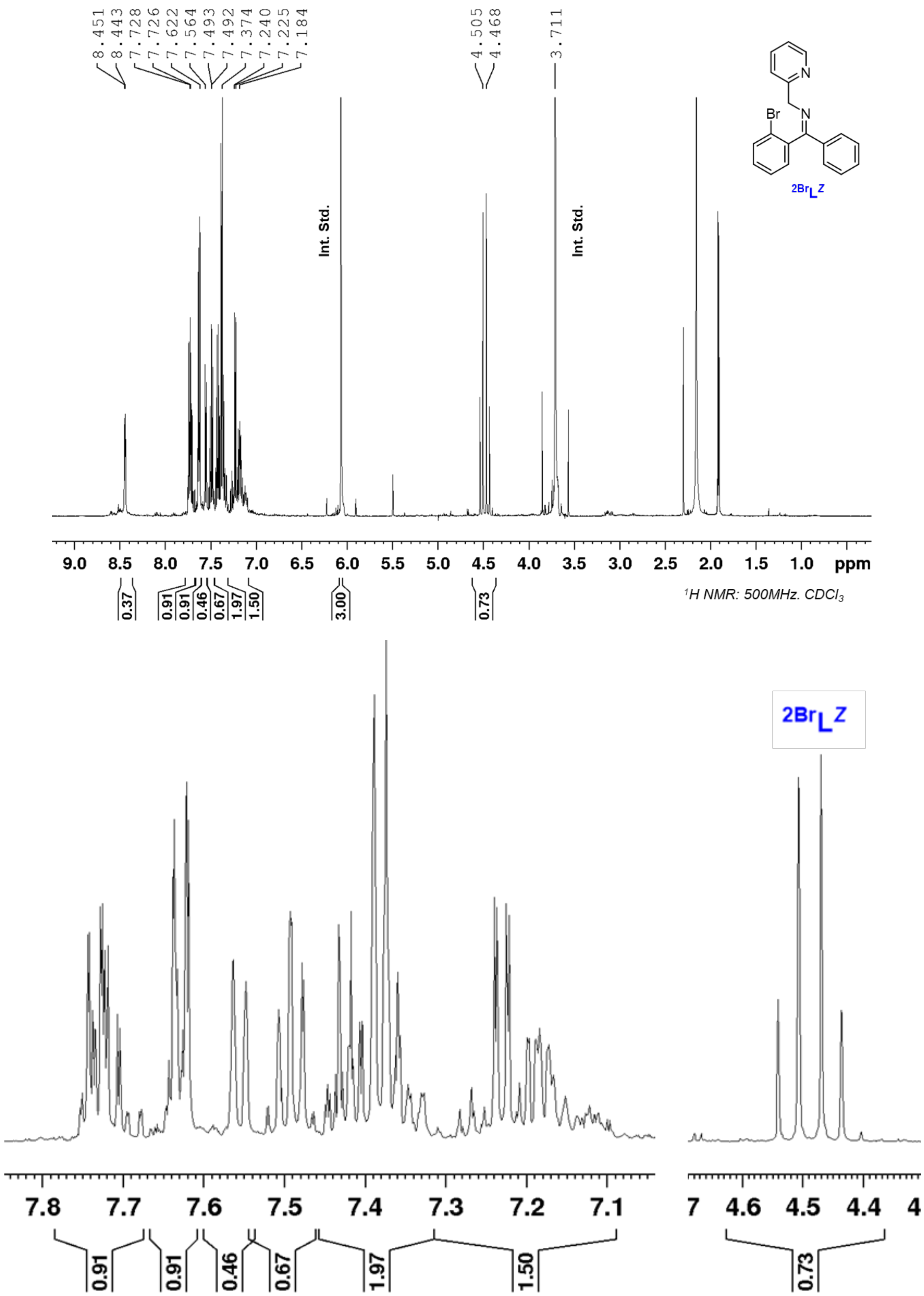

Figure S9. ^{1}H NMR spectra of ^{2Br}L. Note: only one imine isomer is formed.

^{1}H-NMR spectra for the hydroxylation of [2Br]L

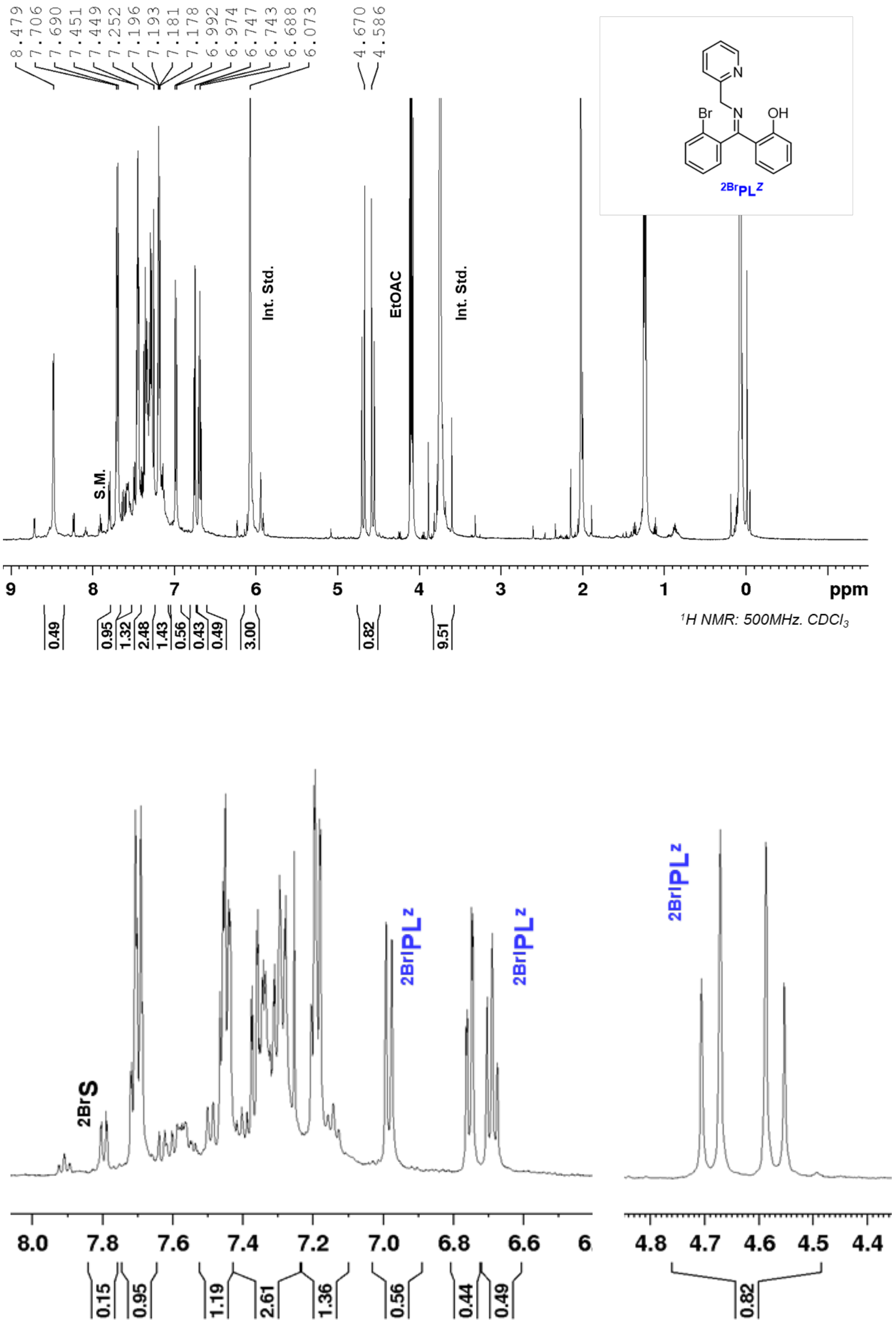

Figure 10. ^{1}H-NMR spectra for the hydroxylation of [2Br]L. Note: only one hydroxylation product is formed.

3.6 ^{4MeO}S and 2-picolyamine

Synthesis of ^{4MeO}L

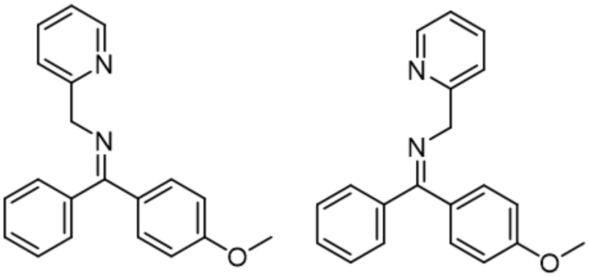

In an oven dried flask, 2-picolylamine (2.2 equiv., 2.34 mL) was added to 4-Methoxybenzophenone (2.08 g, 9.85 mmol) and p- toluenesulfonic acid monohydrate (cat. 20 mg, 1.2 mol%) in toluene (50 mL). The reaction mixture was refluxed under argon with a Dean-Stark apparatus until imine formation was complete (2 days). The reaction was cooled to room temperature and diluted with diethyl ether (30 mL). The organic layer was washed with saturated ammonia chloride (20 mL x 2), saturated aqueous sodium bicarbonate (20 mL), brine (20 mL), and dried with magnesium sulfate. The final product was isolated as a brown solid (90% yield, 2.70 g, 93% pure). ^{1}H-NMR (500 MHz, $CDCl_3$): δ 8.50 (d, L^E+L^Z, 2H), 7.60 (m, L^E+L^Z, 2H), 7.50-7.17 (m, L^E+L^Z, 18H), 6.97 (d, L^Z, 2H), 6.85 (d, L^E, 2H), 4.80 (s, L^Z, 2H), 4.69 (s, L^E, 2H), 3.67 (s, L^E+L^Z, 6H). HRMS (ESI) m/z: [M + Na]+ Calcd for C20H18N2ONa 302.1419, found 303.1486.

Hydroxylation of ^{4MeO}L

The reaction was carried out on 0.159 mmol scale using 51.6 mg of the imine according to the Standard Procedure. The reaction products were quantified using 0.083 mmol of 1,3,5-trimethoxybenzene (int. std.). (60% yield). The identity of the hydroxylation products was confirmed by ^{1}H-NMR.

Cleavage of ^{4MeO}PL

Dissolving ^{4MeO}PL in round bottom flask with 50 mL EtOAc, then adding 100 mL 1M HCl. Reaction was going for 30 min. The resulting mixture was extracted with EtOAc (50 mL X 2). The organic phases were separated, combined, dried over $MgSO_4$, filtered, and dried under vacuum. The reaction products were dissolved in 1.4 mL of $CDCl_3$ solution containing 15 mg of 1,3,5-trimethoxybenzene (internal standard). The reaction products were quantified by ^{1}H-NMR using integration signals that correspond to the starting material and products with the integration signal of the internal standard.

^{1}H-NMR spectra of ^{4MeO}L

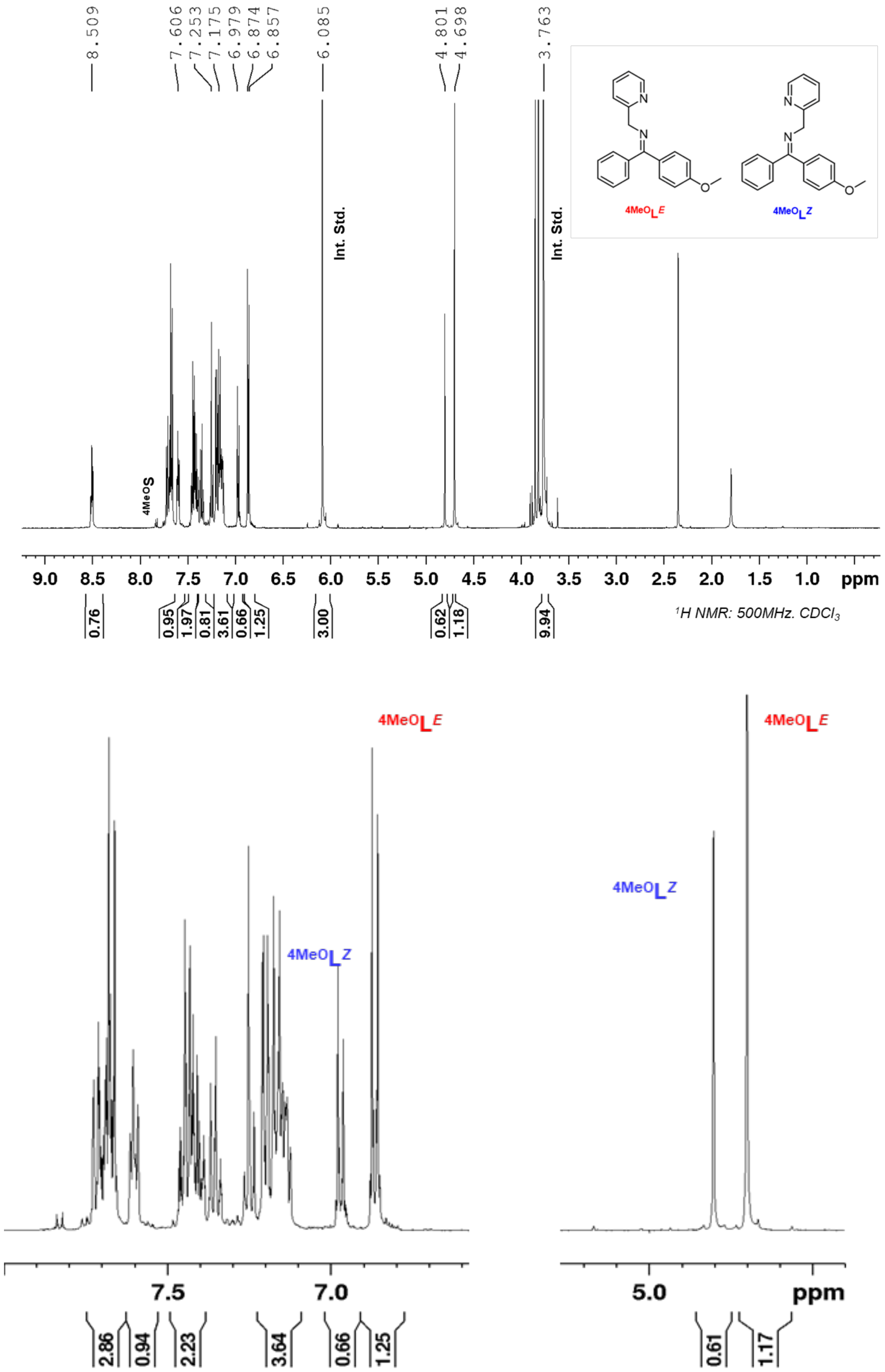

Figure S11. ^{1}H-NMR spectra of **^{4MeO}L**. Note: There are two imine isomers. The ratio of $^{4MeO}L^{E}$ and $^{4MeO}L^{Z}$ (67/33) is calculated using the average of the integration of CH_2 peaks and CH peaks.

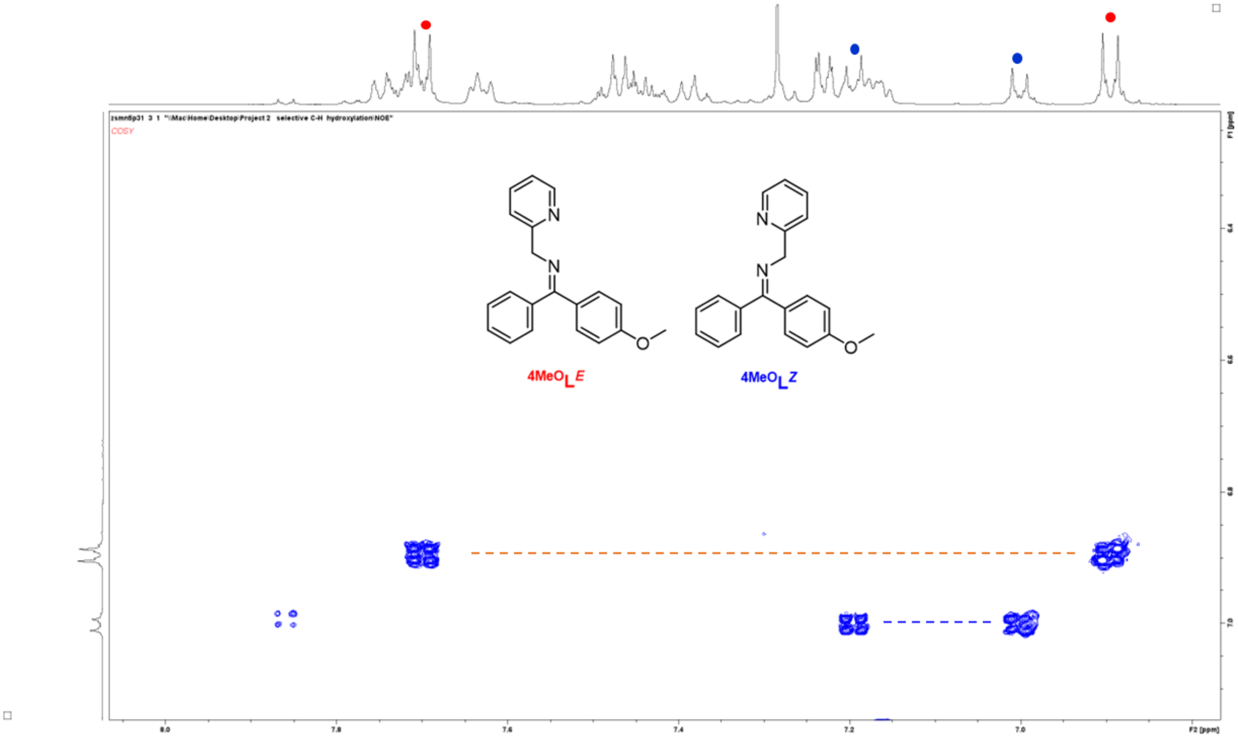

Figure S12. Homonuclear correlation spectroscopy (COSY) spectra for ^{4MeO}L.

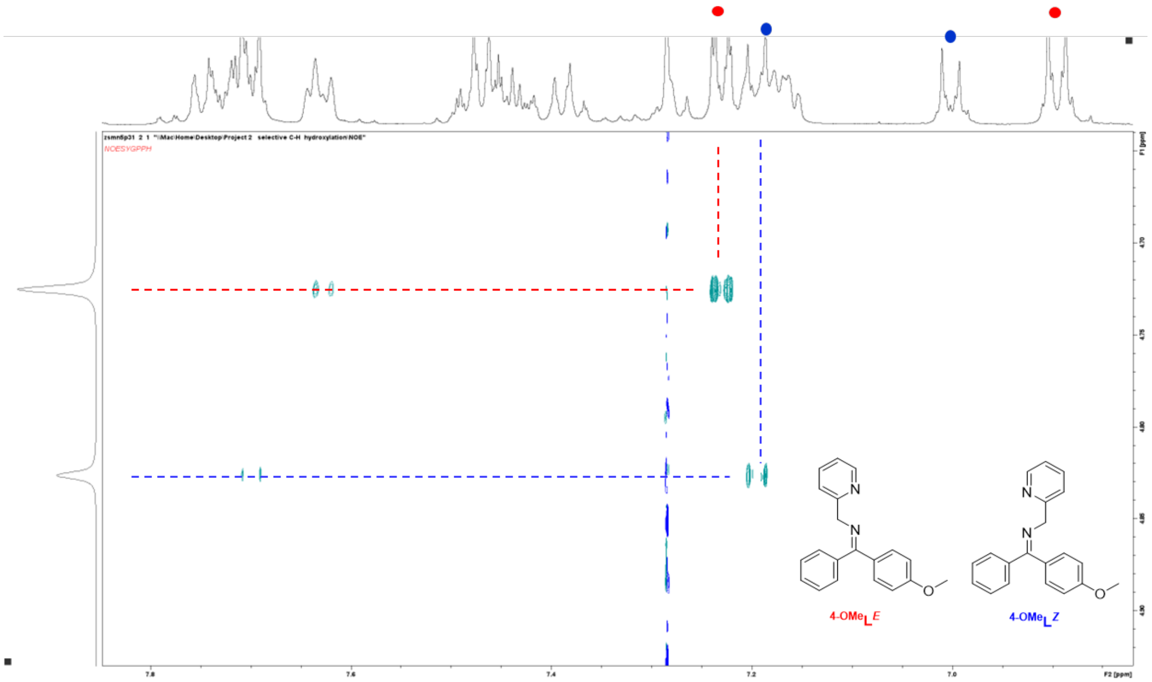

Figure S13. Nuclear Overhauser Effect Spectroscopy (NOESY) spectra for ^{4MeO}L.

^{1}H-NMR spectra for the hydroxylation of [4MeO]L

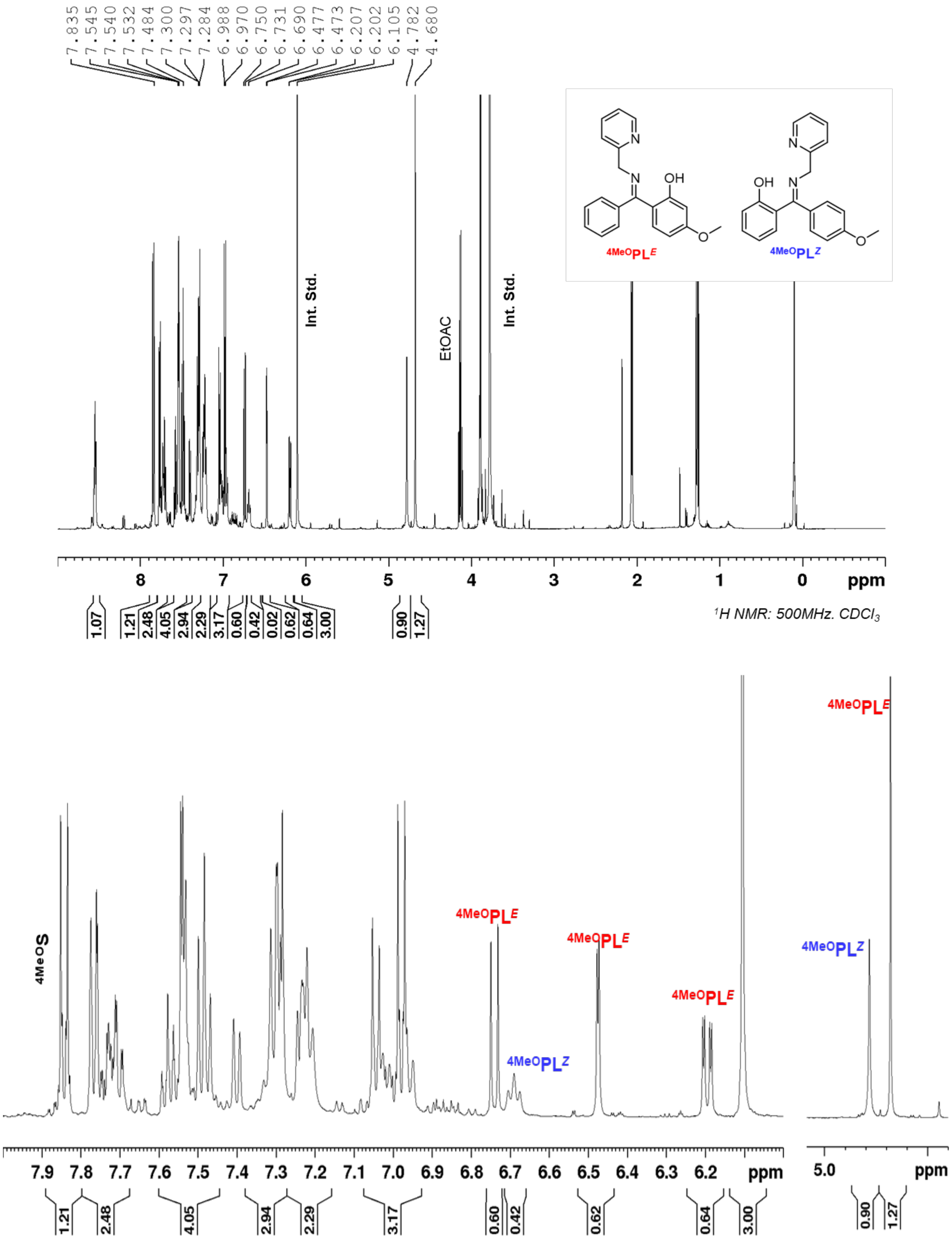

Figure 14. ^{1}H-NMR spectra for the hydroxylation of [4MeO]L. Note: The ratio of [4MeO]PL[E] and [4MeO]PL[Z] (62/38) is calculated using the average of the integration of CH_2 peaks and CH peaks.

^{1}H-NMR spectra for the cleavage of ^{4MeO}PL

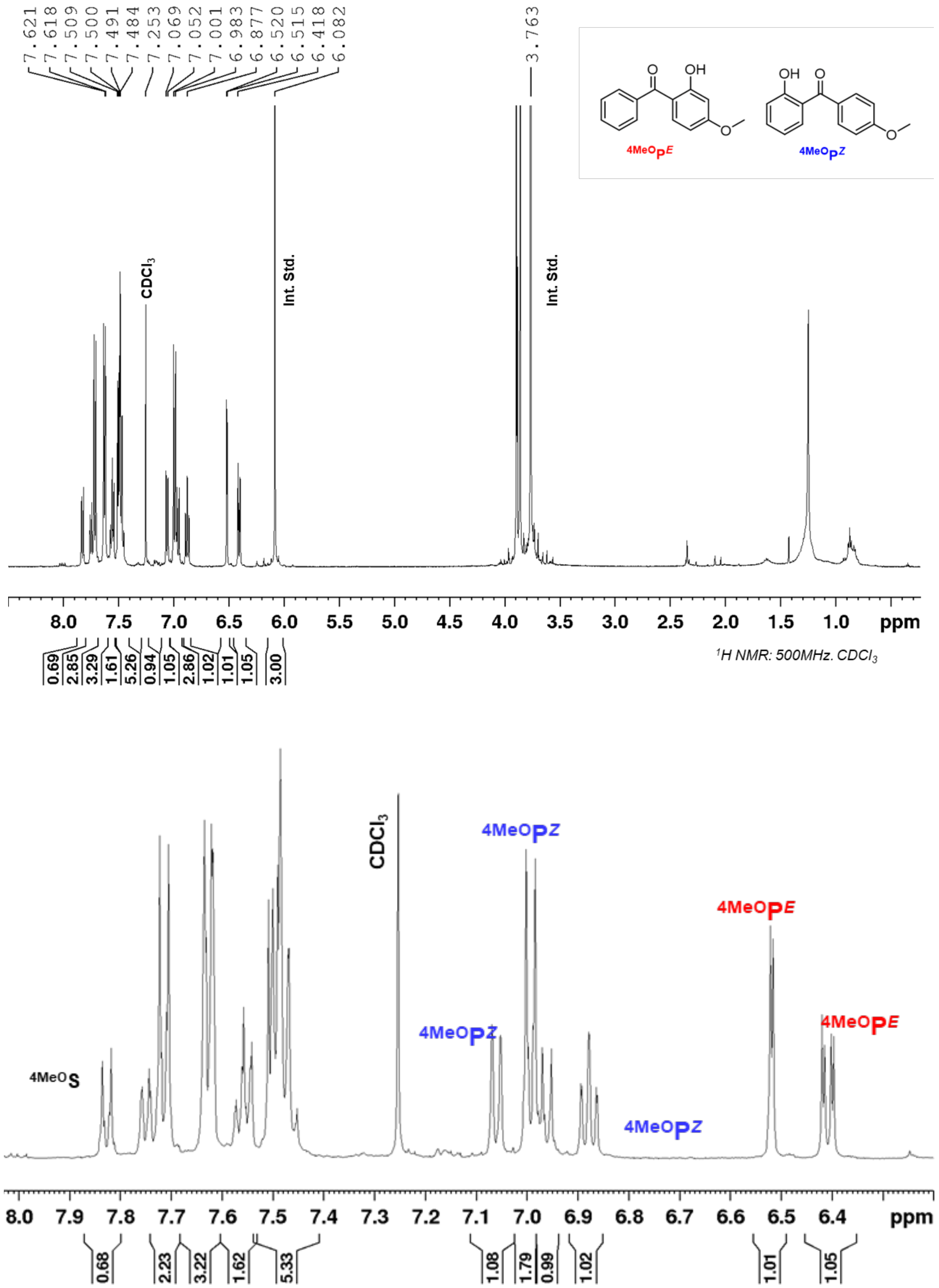

Figure S15. ^{1}H-NMR spectra for the cleavage of ^{4MeO}PL. Note: The ratio of $^{4MeO}P^{E}$ and $^{4MeO}P^{Z}$ (51/49) is calculated using the average of the integration of CH peaks.

3.7 ^{4Me}S and 2-picolyamine

Synthesis of ^{4Me}L

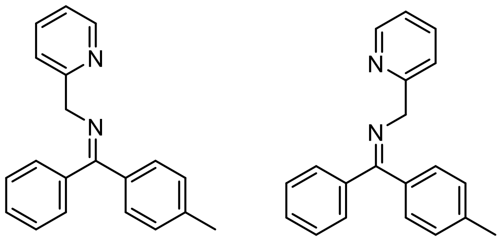

^{4Me}L *E* ^{4Me}L *Z*

In an oven dried flask, 2-picolylamine (2.2 equiv., 2.2 mL) was added to 4-Methoxybenzophenone (1.92 g, 9.85 mmol) and p- toluenesulfonic acid monohydrate (cat. 20 mg, 1.2 mol%) in toluene (50 mL). The reaction mixture was refluxed under argon with a Dean-Stark apparatus until imine formation was complete (2 days). The reaction was cooled to room temperature and diluted with diethyl ether (30 mL). The organic layer was washed with saturated ammonia chloride (20 mL x 2), saturated aqueous sodium bicarbonate (20 mL), brine (20 mL), and dried with magnesium sulfate. The final product was isolated as a brown solid (80% yield, 2.25 g, 99% pure). ^{1}H-NMR (500 MHz, $CDCl_3$): δ 8.50 (d, L^E+L^Z, 2H), 7.60 (m, L^E+L^Z, 4H), 7.50 (dd, L^E+L^Z , 4H) 7.50-7.29 (m, L^E+L^Z, 6H), 7.23-7.08 (m, L^E+L^Z, 8H), 6.85 (d, L^E, 2H), 4.76 (s, L^Z, 2H), 4.72 (s, L^E, 2H), 2.36 (s, L^E+L^Z, 6H), HRMS (ESI) m/z: [M + Na]+ Calcd for C20H18N2Na 286.3780, found 287.1543.

Hydroxylation of ^{4Me}L

The reaction was carried out on 0.159 mmol scale using 46.0 mg of the imine according to the Standard Procedure. The reaction products were quantified using 0.159 mmol of 1,3,5-trimethoxybenzene (int. std.). (70% yield). The identity of the hydroxylation products was confirmed by ^{1}H-NMR.

Cleavage of ^{4Me}PL

Dissolving ^{4Me}PL in round bottom flask with 50 mL EtOAc, then adding 100 mL 1M HCl. Reaction was going for 30 min. The resulting mixture was extracted with EtOAc (50 mL X 2). The organic phases were separated, combined, dried over $MgSO_4$, filtered, and dried under vacuum. The reaction products were dissolved in 1.4 mL of $CDCl_3$ solution containing 27.1 mg of 1,3,5-trimethoxybenzene (internal standard). The reaction products were quantified by ^{1}H-NMR using integration signals that correspond to the starting material and products with the integration signal of the internal standard.

^{1}H-NMR spectra of [4Me]L

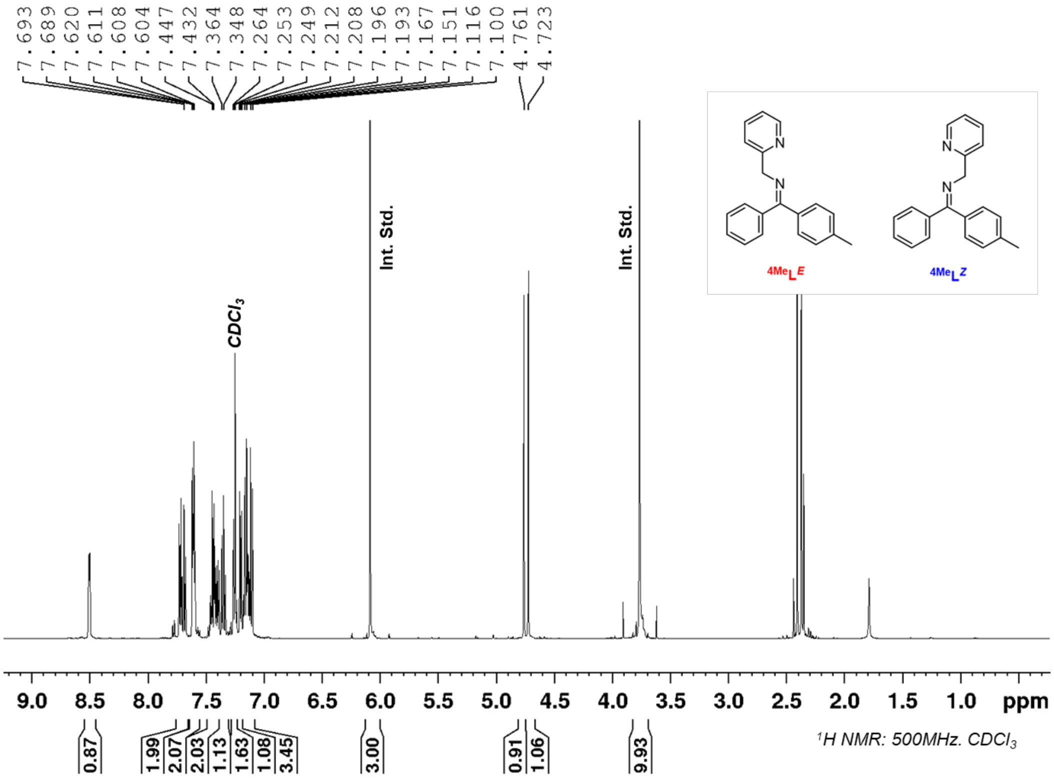

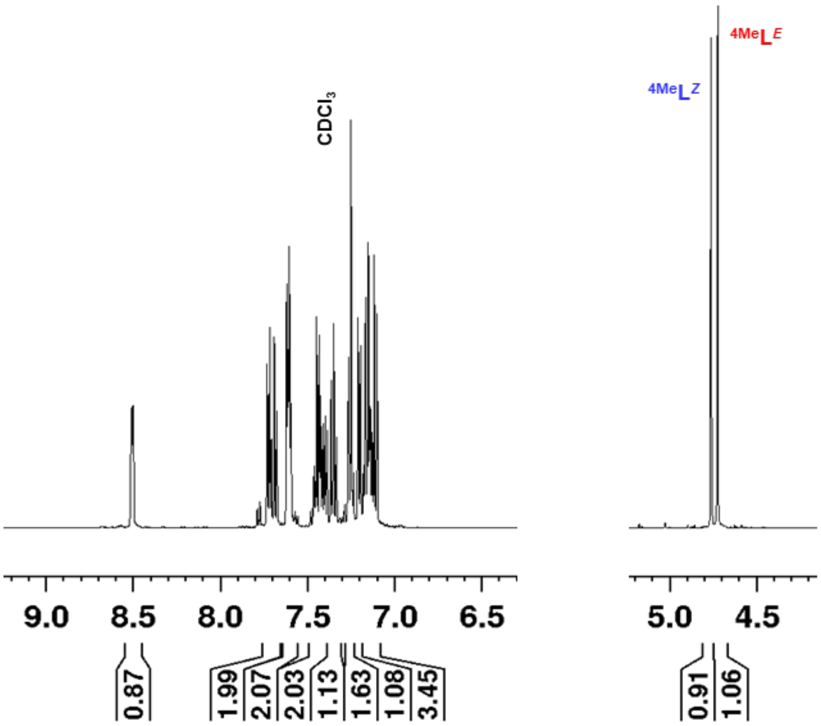

Figure S16. ^{1}H-NMR spectra of ^{4Me}L. Note: The ratio of $^{4Me}L^{E}$ and $^{4Me}L^{Z}$ is calculated using the average of the integration of CH_2 peaks and CH peaks (52:48)

^{1}H-NMR spectra for the hydroxylation of [4Me]L

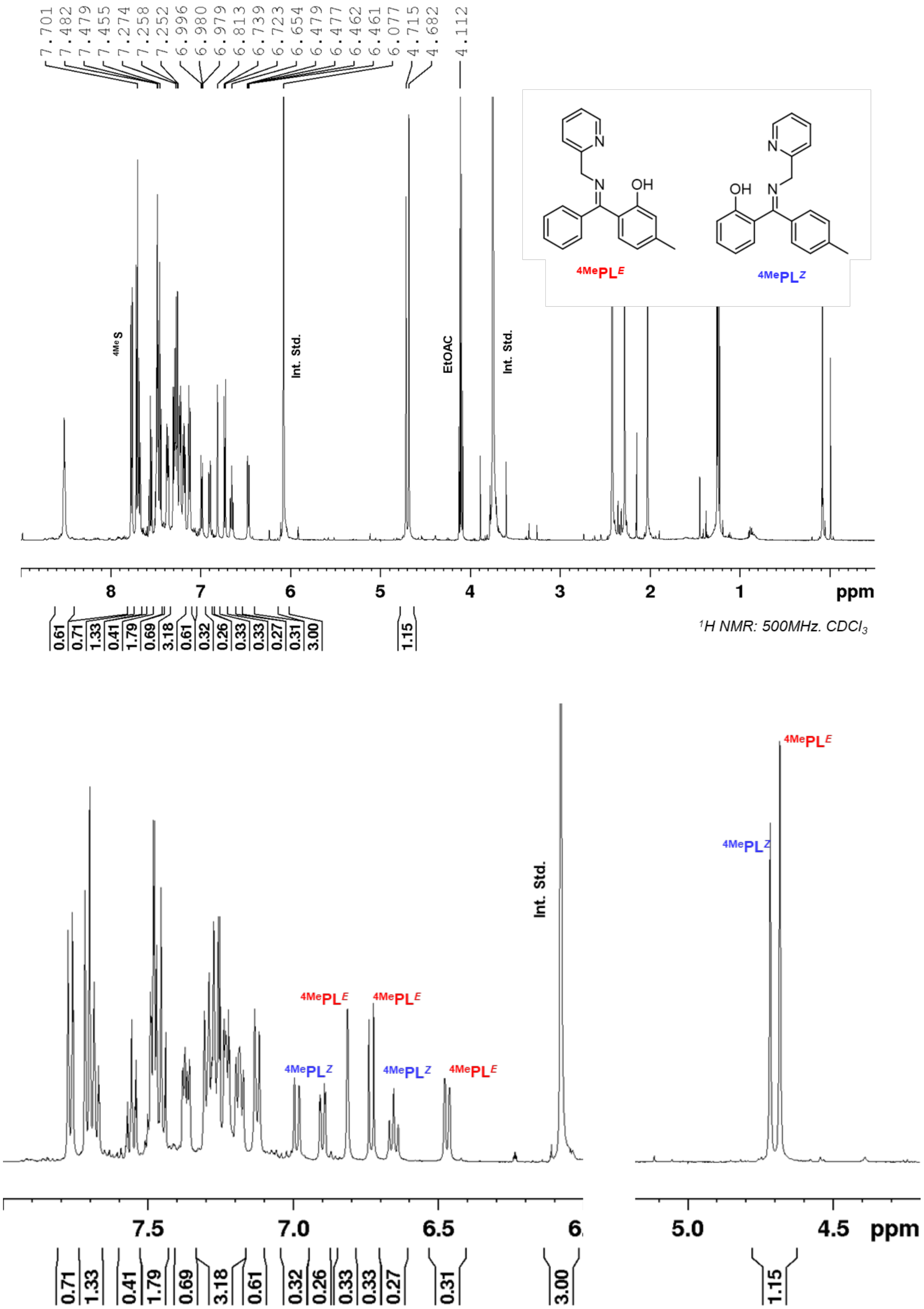

Figure S17. ^{1}H-NMR spectra for the hydroxylation of ^{4Me}L. Note: The ratio of ^{4Me}PL^E and ^{4Me}PL^Z (54/46) is calculated using the average of the integration of CH_2 peaks and CH peaks.

^{1}H-NMR spectra for the cleavage of ^{4Me}PL

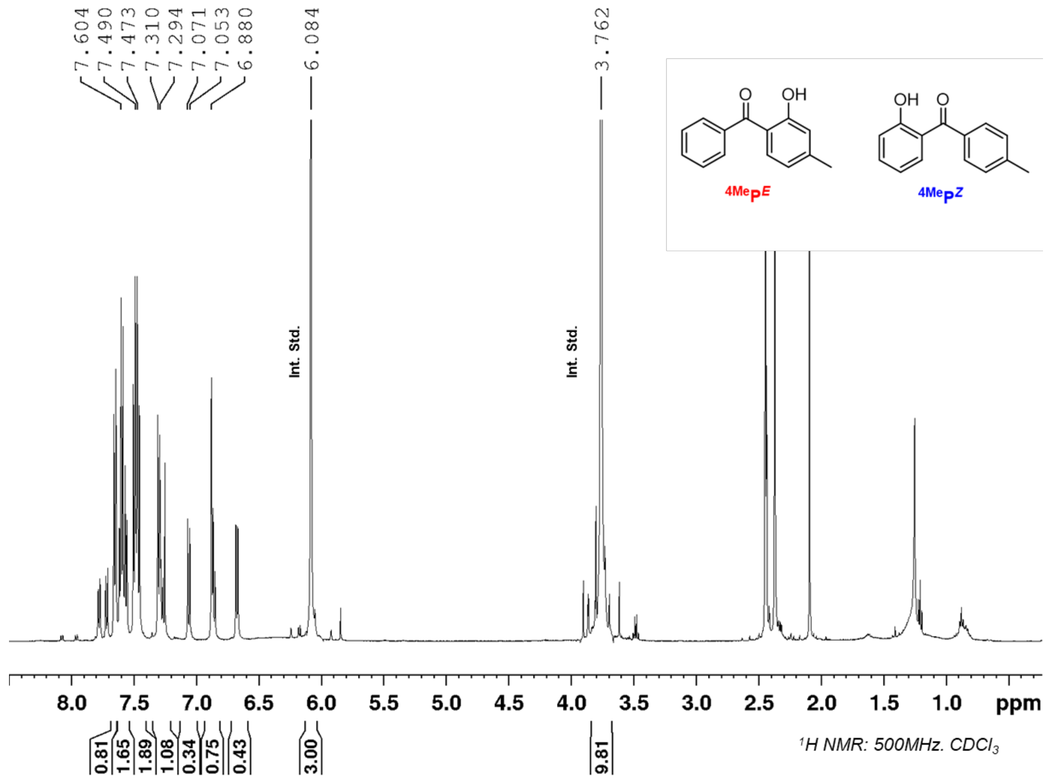

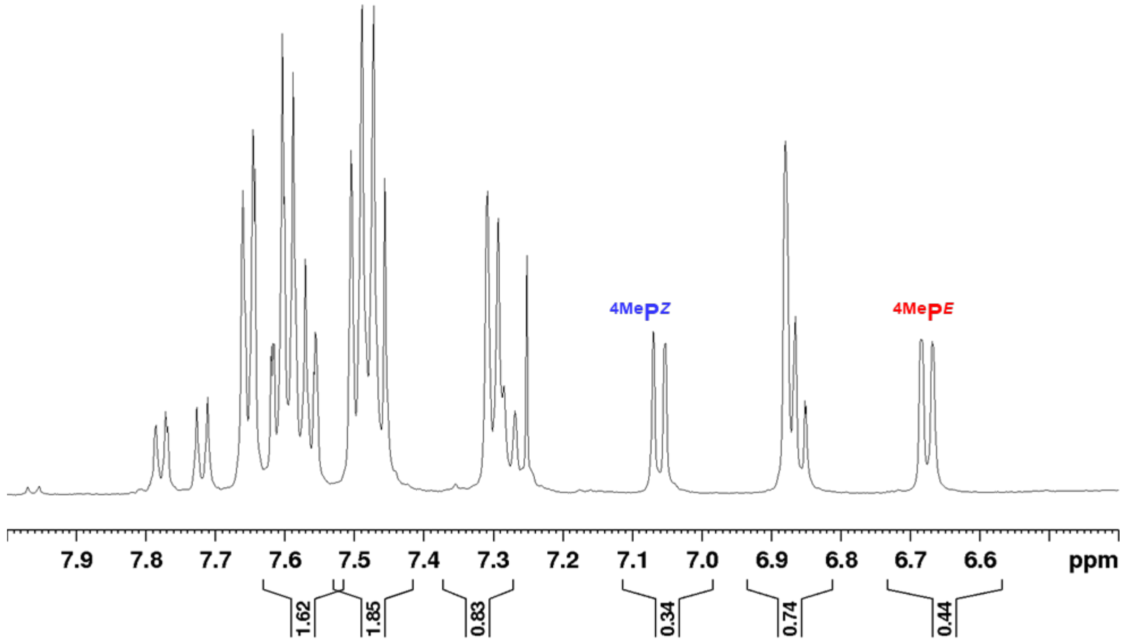

Figure S18. ^{1}H-NMR spectra for the cleavage of ^{4Me}PL. Note: The ratio of $^{4Me}P^{E}$ and $^{4Me}P^{Z}$ (56/44) is calculated using the average of the integration of CH peaks.

3.8 ^{4F}S and 2-picolyamine

Synthesis of ^{4F}L

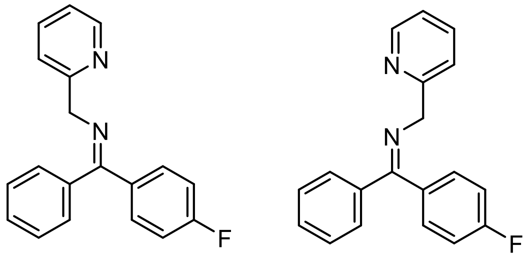

^{4F}L^{E} ^{4F}L^{Z}

In an oven dried flask, 2-picolylamine (2.2 equiv., 2.2 mL) was added to 4-flourobenzophenone (1.97 g, 9.85 mmol) and p- toluenesulfonic acid monohydrate (cat. 20 mg, 1.2 mol%) in toluene (50 mL). The reaction mixture was refluxed under argon with a Dean-Stark apparatus until imine formation was complete (3 days). The reaction was cooled to room temperature and diluted with diethyl ether (30 mL). The organic layer was washed with saturated ammonia chloride (20 mL x 2), saturated aqueous sodium bicarbonate (20 mL), brine (20 mL), and dried with magnesium sulfate. The final product was isolated as a brown solid (94% yield, 2.69 g, 95% pure). ^{1}H-NMR (500 MHz, $CDCl_3$): δ 8.51 (d, L^E+L^Z, 2H), 7.71 (m, L^E+L^Z, 6H), 7.60 (dd, L^E+L^Z, 2H) 7.46-7.25 (m, L^E+L^Z, 4H), 7.15-7.25 (m, L^E+L^Z, 8H), 7.15 (m, L^E+L^Z, 2H), 7.02 (t, L^E, 2H), 4.73 (s, L^Z, 2H), 4.71 (s, L^E, 2H). HRMS (ESI) m/z: [M + Na]+ Calcd for C19H15FN2Na 290.3414, found 291.1289.

Hydroxylation of ^{4F}L

The reaction was carried out on 0.159 mmol scale using 48.5 mg of the imine according to the Standard Procedure. The reaction products were quantified using 0.159 mmol of 1,3,5-trimethoxybenzene (int. std.). (60% yield). The identity of the hydroxylation products was confirmed by ^{1}H-NMR.

Cleavage of ^{4F}PL

Dissolving ^{4F}PL in round bottom flask with 50 mL EtOAc, then adding 100 mL 1M HCl. Reaction was going for 30 min. The resulting mixture was extracted with EtOAc (50 mL X 2). The organic phases were separated, combined, dried over $MgSO_4$, filtered, and dried under vacuum. The reaction products were dissolved in 1.4 mL of $CDCl_3$ solution containing 27.1 mg of 1,3,5-trimethoxybenzene (internal standard). The reaction products were quantified by ^{1}H-NMR using integration signals that correspond to the starting material and products with the integration signal of the internal standard.

^{1}H-NMR spectra of ^{4F}L

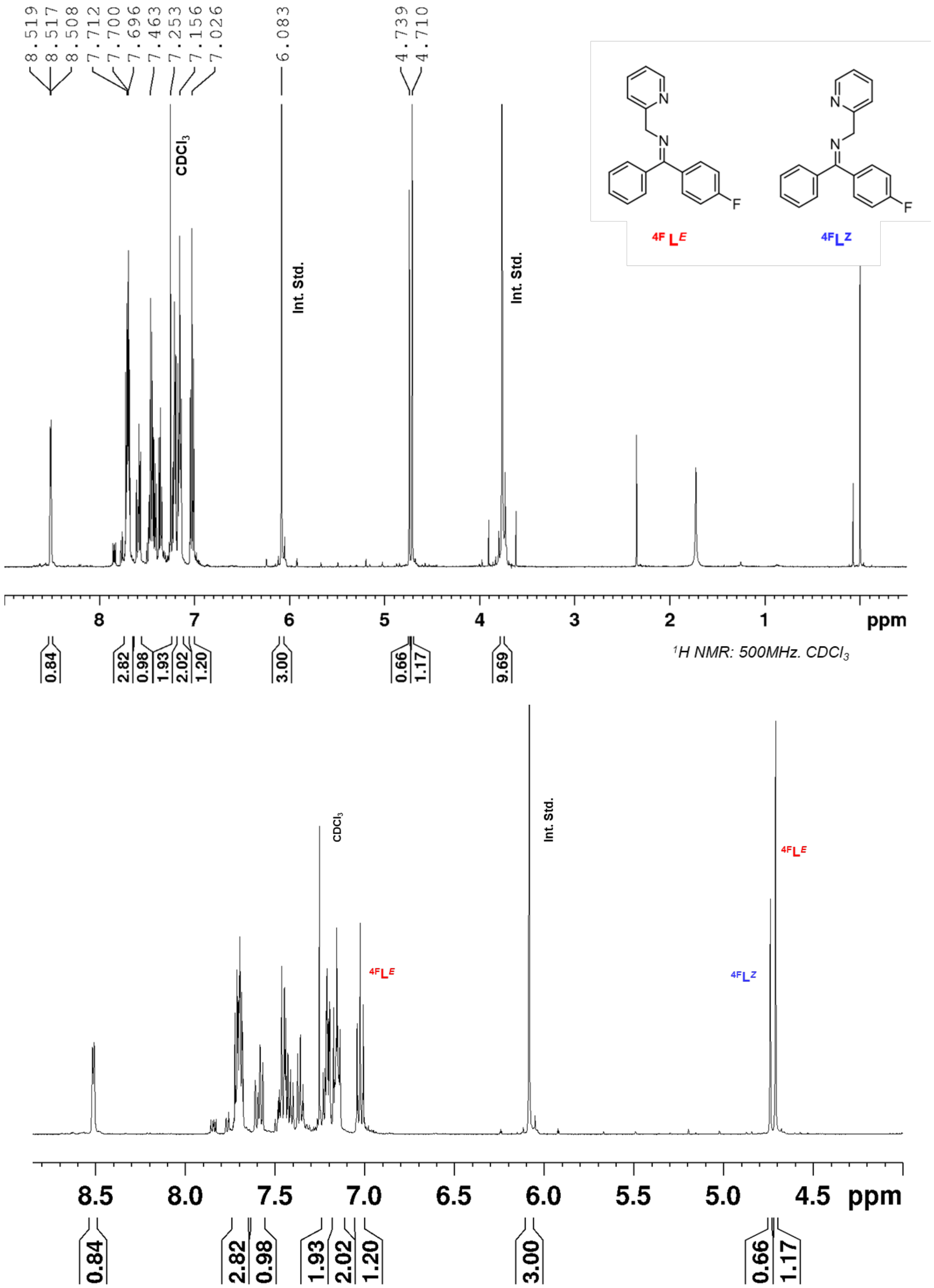

Figure S19. ^{1}H-NMR spectra of ^{4F}L. Note: There are two imine isomers. The ratio of $^{F}L^{E}$ and $^{F}L^{Z}$ (64/36) is calculated using the average of the integration of CH_2 peaks and CH peaks.

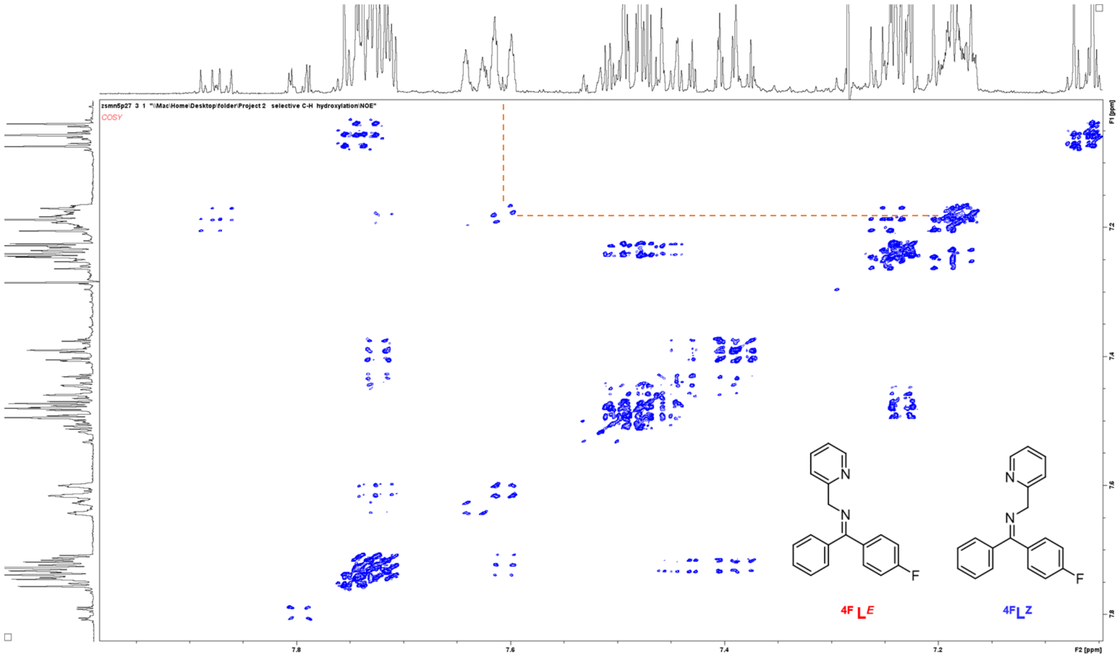

Figure S20. Homonuclear correlation spectroscopy (COSY) spectra of ^{4F}L

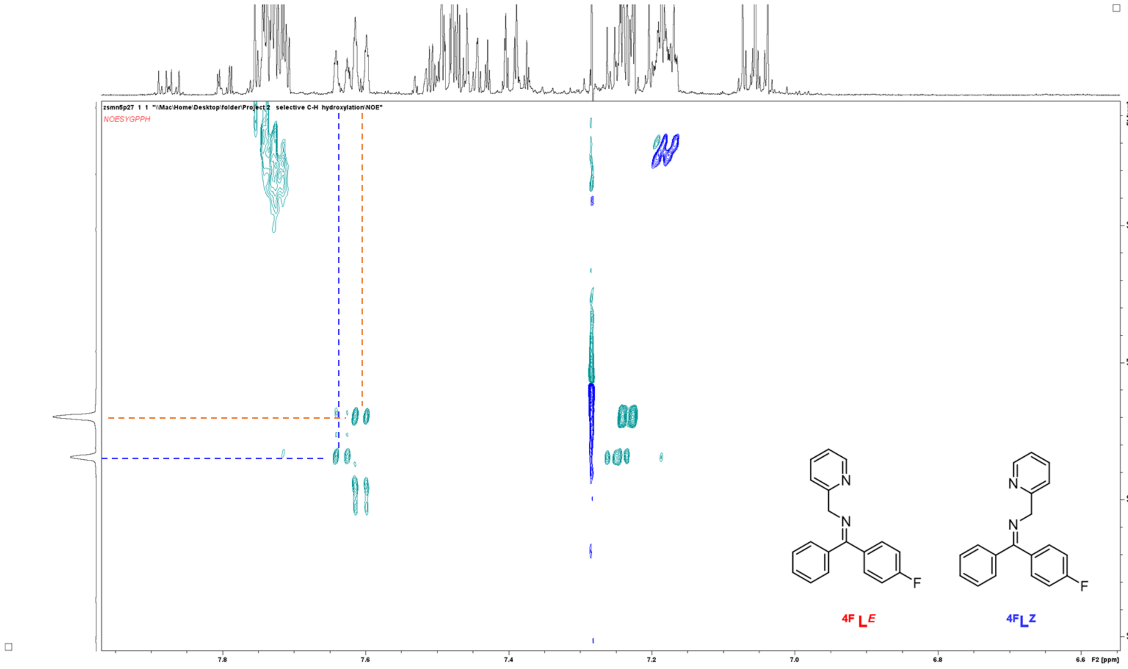

Figure S21. Nuclear Overhauser Effect Spectroscopy (NOESY) spectra of ^{4F}L.

¹H-NMR spectra for the hydroxylation of ^{4F}L

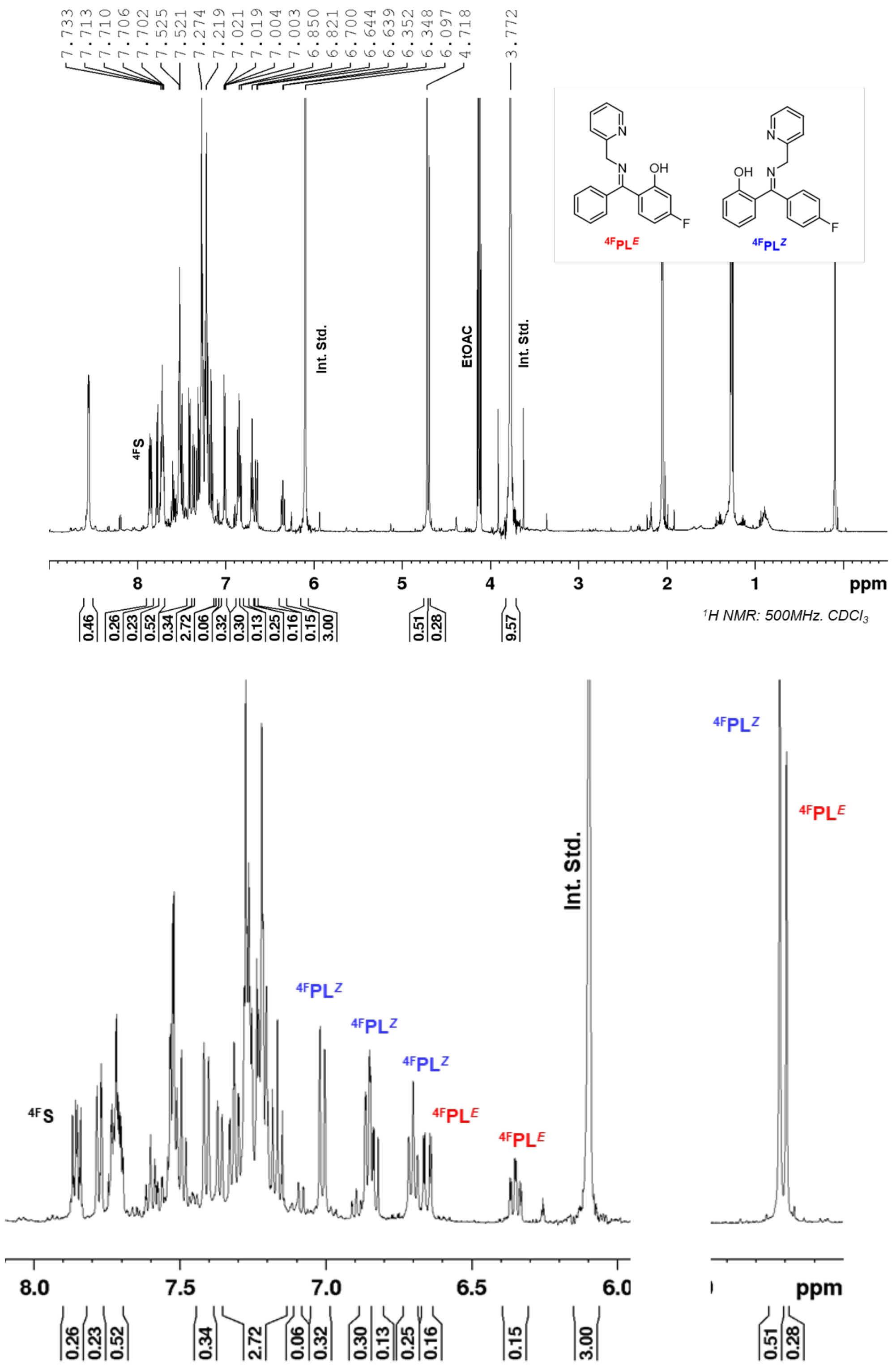

Figure S22. ¹H-NMR spectra for the hydroxylation of ^{4F}L. Note: The ratio of ^{4F}PL^{E} and ^{4F}PL^{Z} (33/67) is calculated using the average of the integration of the CH_2 peaks and CH peaks.

^{1}H-NMR spectra for the cleavage of ^{4F}PL

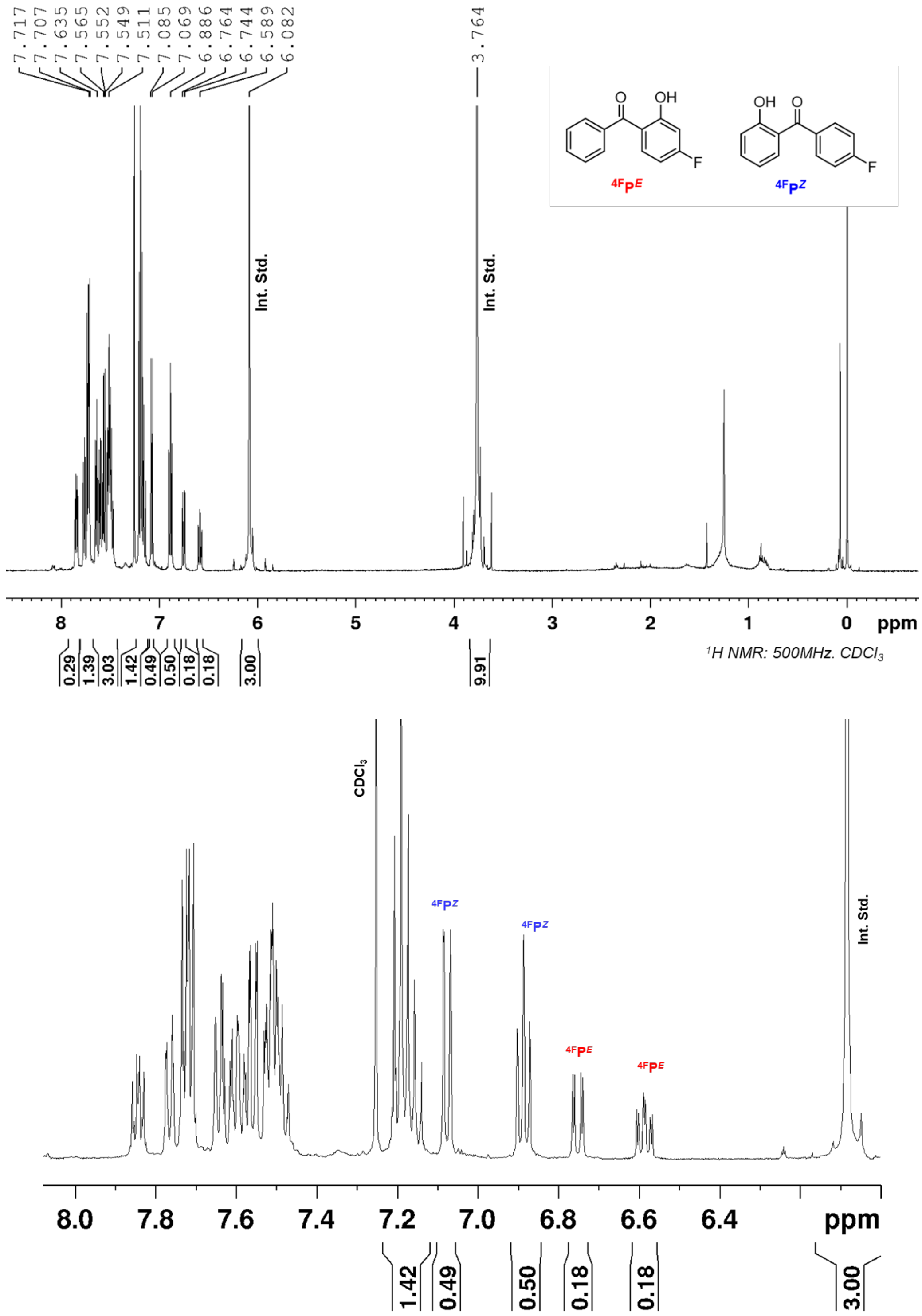

Figure S23. ^{1}H-NMR spectra for the cleavage ^{4F}PL. Note: There are two imine isomers. The ratio of $^{4F}P^{E}$ and $^{4F}P^{Z}$ (27/73) is calculated using the average of the integration of CH_2 peaks and CH peaks.

3.9 ^{4Cl}S and 2-picolyamine

Synthesis of ^{4Cl}L

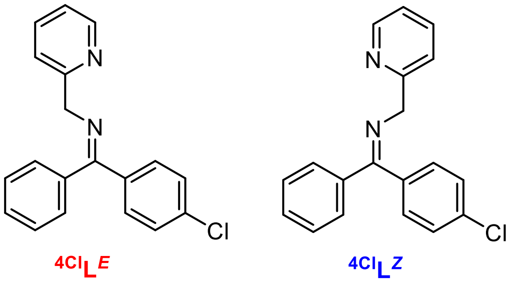

In an oven dried flask, 2-picolylamine (2.2 equiv., 0.63 mL) was added to 4-Methoxybenzophenone (0.75 g, 3.8 mmol) and p- toluenesulfonic acid monohydrate (cat. 10 mg, 1.5 mol%) in toluene (50 mL). The reaction mixture was refluxed under argon with a Dean-Stark apparatus until imine formation was complete (6 days). The reaction was cooled to room temperature and diluted with diethyl ether (30 mL). The organic layer was washed with saturated ammonia chloride (20 mL x 2), saturated aqueous sodium bicarbonate (20 mL), brine (20 mL), and dried with magnesium sulfate. The final product was isolated as a brown solid (80% yield, 0.93 g, 95% pure). ^{1}H-NMR (500 MHz, $CDCl_3$): δ 8.51 (d, L^E+L^Z, 2H), 7.8-7.60 (m, L^E+L^Z, 6H), 7.59-7.41 (m, L^E+L^Z , 4H) 7.39-7.32 (m, L^E+L^Z, 4H), 7.29-7.15 (m, L^E+L^Z, 8H), 4.72 (s, L^Z, 2H), 4.71 (s, L^E, 2H). HRMS (ESI) m/z: [M + Na]+ Calcd for C19H15ClN2Na 306.7930, found 307.1023.

Hydroxylation of ^{4Cl}L

The reaction was carried out on 0.159 mmol scale using 51.2 mg of the imine according to the Standard Procedure. The reaction products were quantified using 0.159 mmol of 1,3,5-trimethoxybenzene (int. std.). (51% yield). The identity of the hydroxylation products was confirmed by ^{1}H-NMR.

Cleavage of ^{4Cl}PL

Dissolving ^{4Cl}PL in round bottom flask with 50 mL EtOAc, then adding 100 mL 1M HCl. Reaction was going for 30 min. The resulting mixture was extracted with EtOAc (50 mL X 2). The organic phases were separated, combined, dried over $MgSO_4$, filtered, and dried under vacuum. The reaction products were dissolved in 1.4 mL of $CDCl_3$ solution containing 27.1 mg of 1,3,5-trimethoxybenzene (internal standard). The reaction products were quantified by ^{1}H-NMR using integration signals that correspond to the starting material and products with the integration signal of the internal standard.

^{1}H-NMR spectra of ^{4Cl}L

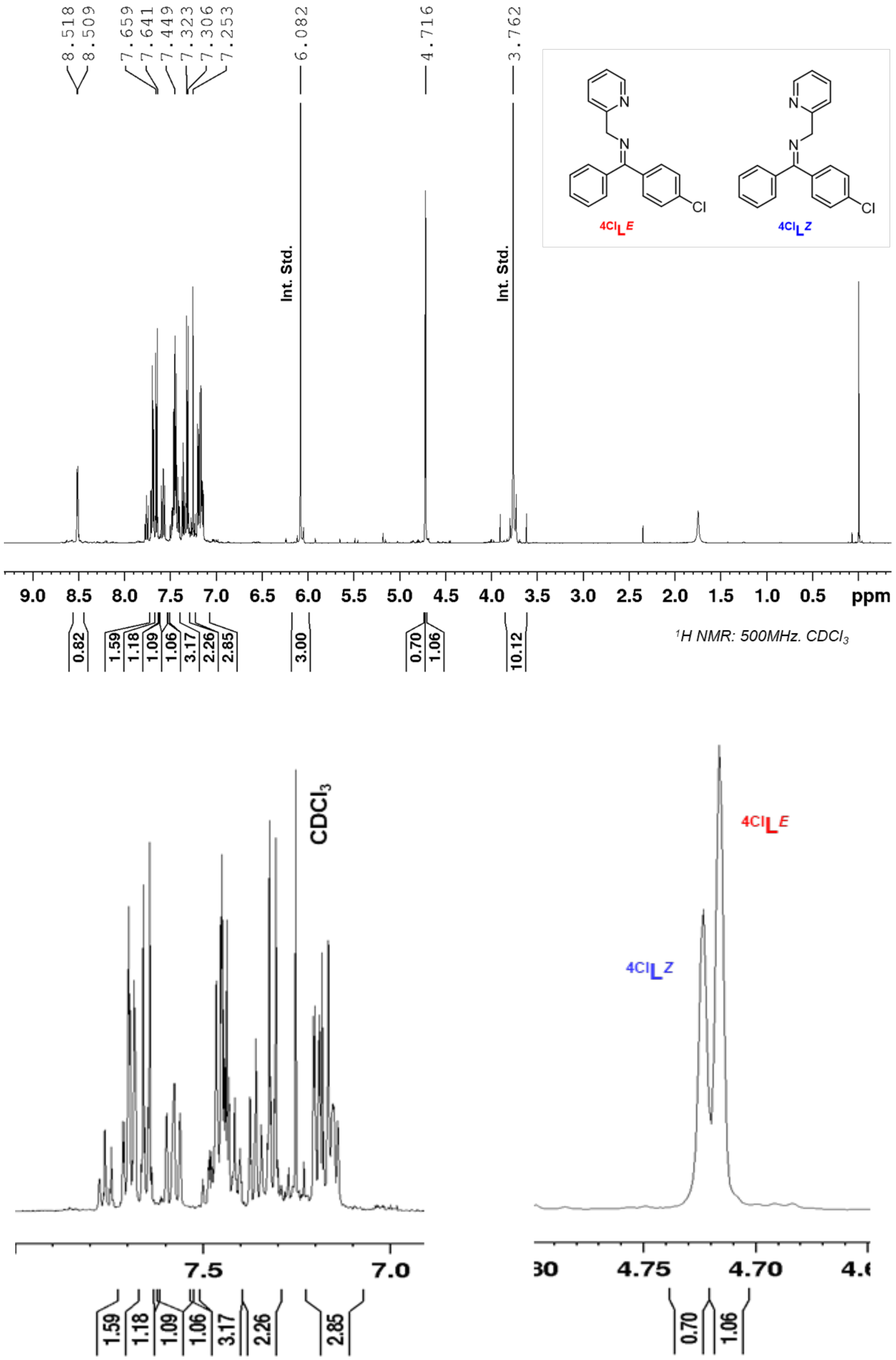

Figure S24. ^{1}H-NMR spectra of ^{4Cl}L. Note: There are two imine isomers. The ratio of $^{4Cl}L^{E}$ and $^{4Cl}L^{Z}$ (62/38) is calculated using the average of the integration of CH_2 peaks and CH peaks.

^{1}H-NMR spectra for the hydroxylation of ^{4Cl}L

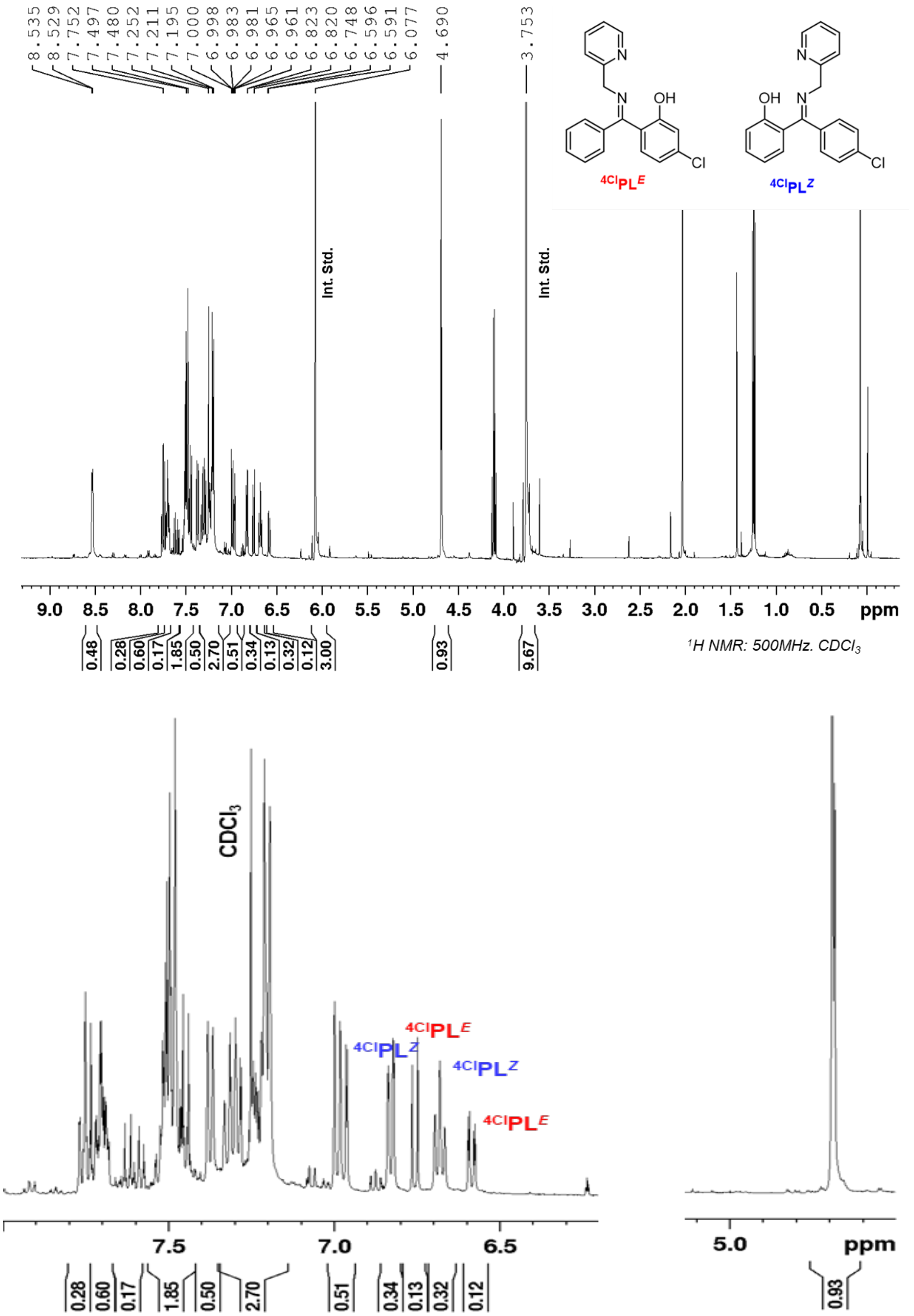

Figure S25. ^{1}H-NMR spectra for the hydroxylation of ^{4Cl}L. Note: The ratio of $^{4Cl}PL^{E}$ and $^{4Cl}PL^{Z}$ (28/72) is calculated using the average of the integration of CH_2 peaks and CH peaks.

^{1}H-NMR spectra for the cleavage of ^{4Cl}PL

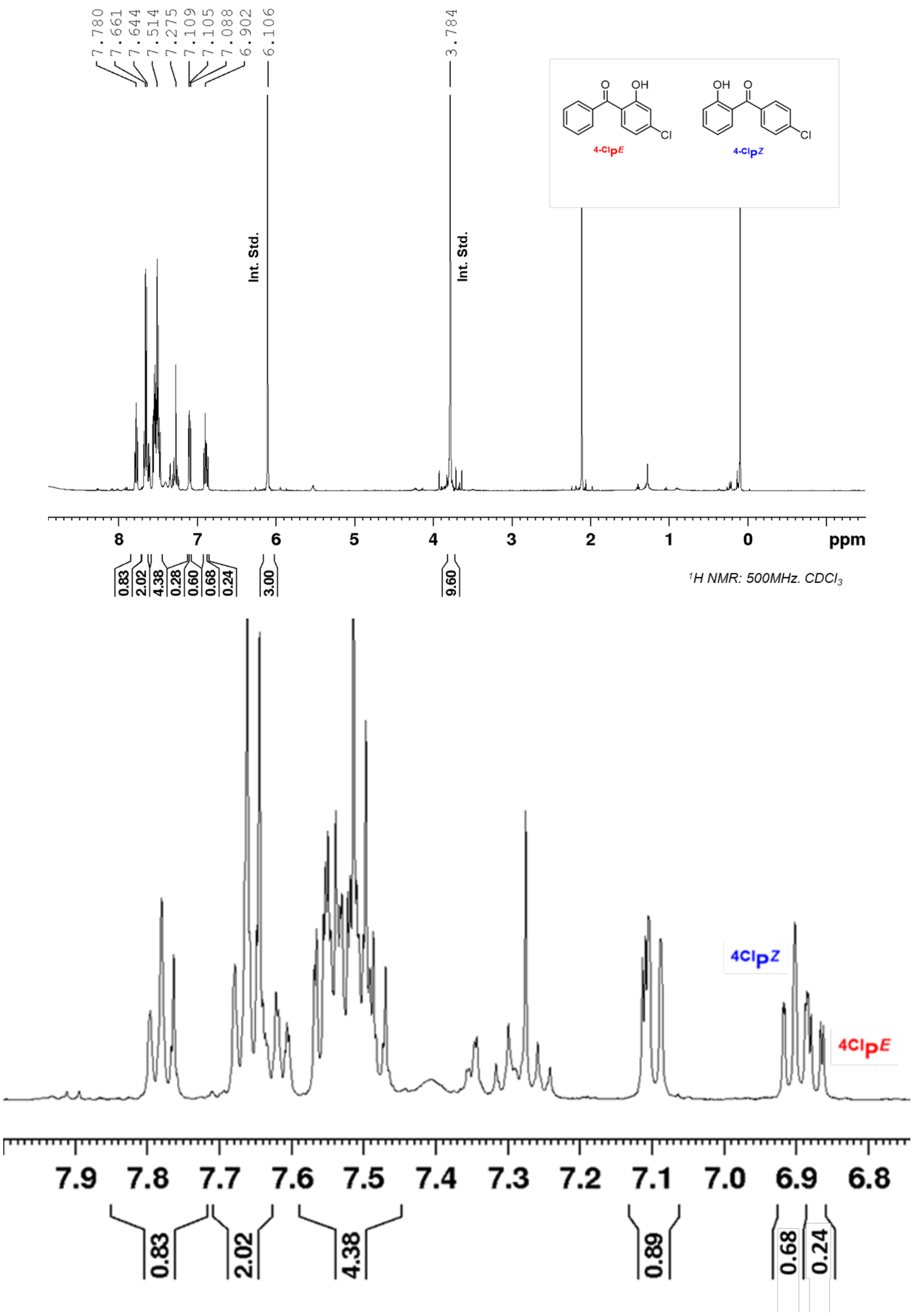

Figure S26. ^{1}H-NMR spectra for the cleavage of ^{4Cl}PL. Note: The ratio of $^{4Cl}P^{E}$ and $^{4Cl}P^{Z}$ (40/60) is calculated using the average of the integration of CH peaks (Note: the overlap between two products precludes establishing an accurate ratio of $^{4Cl}P^{E}$ and $^{4Cl}P^{Z}$).

3.10 ^{4Br}S and 2-picolyamine

Synthesis of ^{4Br}L

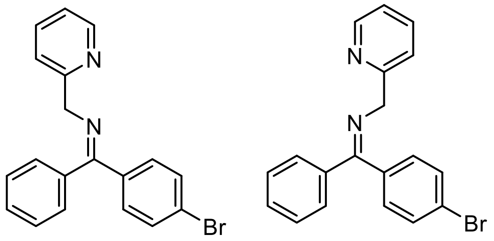

^{4Br}L*E* ^{4Br}L*Z*

In an oven dried flask, 2-picolylamine (2.2 equiv., 0.63 mL) was added to 4-Methoxybenzophenone (0.75 g, 3.8 mmol) and p- toluenesulfonic acid monohydrate (cat. 10 mg, 1.5 mol%) in toluene (50 mL). The reaction mixture was refluxed under argon with a Dean-Stark apparatus until imine formation was complete (6 days). The reaction was cooled to room temperature and diluted with diethyl ether (30 mL). The organic layer was washed with saturated ammonia chloride (20 mL x 2), saturated aqueous sodium bicarbonate (20 mL), brine (20 mL), and dried with magnesium sulfate. The final product was isolated as a brown solid (90% yield, 1.2 g, 85% pure). ^{1}H-NMR (500 MHz, $CDCl_3$): δ 8.54 (d, L^E+L^Z, 2H), 7.72 (m, L^E+L^Z, 4H), 7.68-7.60 (m, L^E+L^Z , 4H) 7.59-7.48 (m, L^E+L^Z, 4H), 7.21-7.12 (m, L^E+L^Z, 12H), 4.74 (s, L^Z, 2H), 4.73 (s, L^E, 2H). HRMS (ESI) m/z: [M + Na]+ Calcd for C19H15BrN2 calculated 351.2470, found 353.1069.

Hydroxylation of ^{4Br}L

The reaction was carried out on 0.159 mmol scale using 65.6 mg of the imine according to the Standard Procedure. The reaction products were quantified using 0.159 mmol of 1,3,5-trimethoxybenzene (int. std.). (45% yield). The identity of the hydroxylation products was confirmed by ^{1}H-NMR.

Cleavage of ^{4Br}PL

Dissolving ^{4Br}PL in round bottom flask with 50 mL EtOAc, then adding 100 mL 1M HCl. Reaction was going for 30 min. The resulting mixture was extracted with EtOAc (50 mL X 2). The organic phases were separated, combined, dried over $MgSO_4$, filtered, and dried under vacuum. The reaction products were dissolved in 1.4 mL of $CDCl_3$ solution containing 27.1 mg of 1,3,5-trimethoxybenzene (internal standard). The reaction products were quantified by ^{1}H-NMR using integration signals that correspond to the starting material and products with the integration signal of the internal standard.

^{1}H-NMR spectra of ^{4Br}L

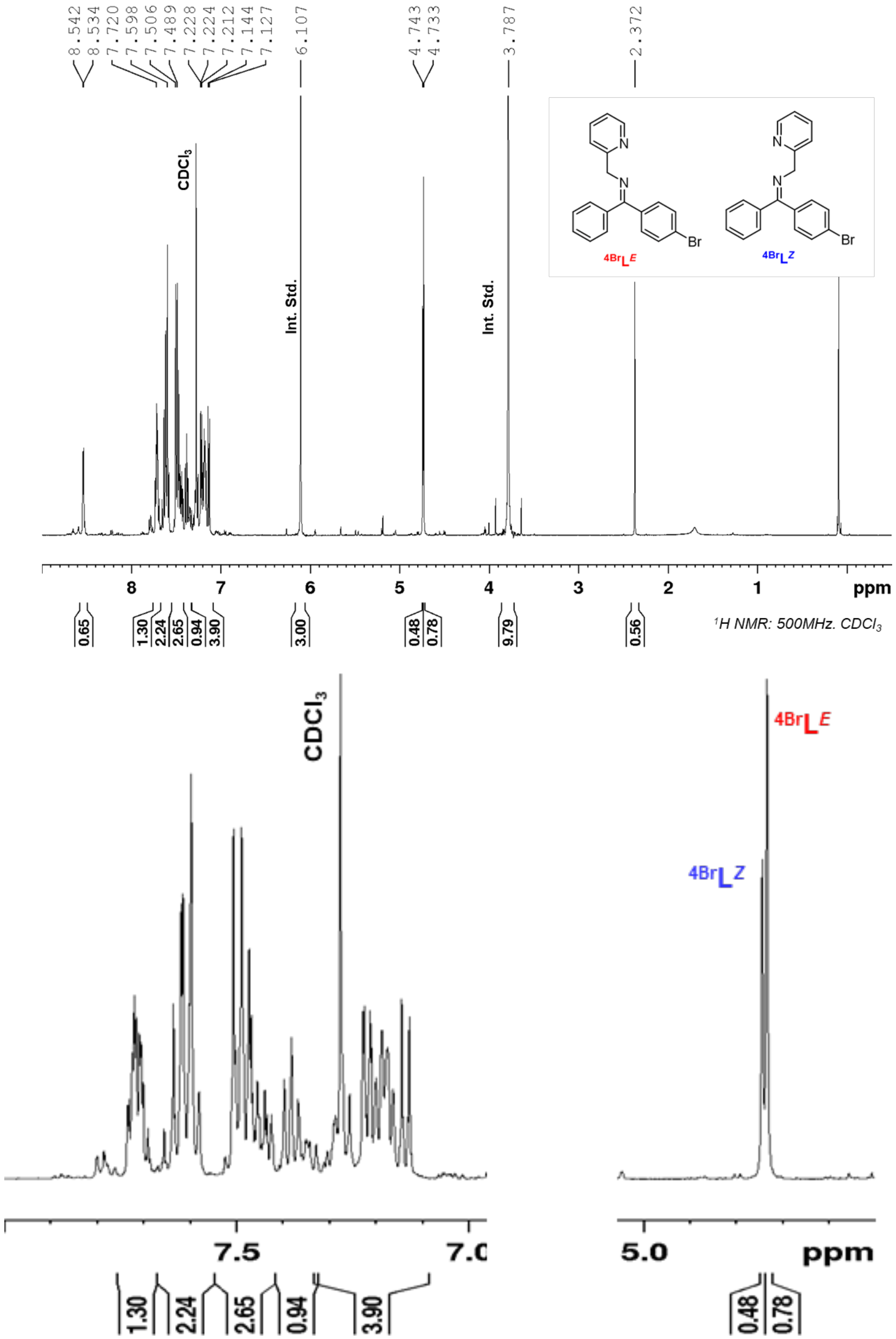

Figure S27. ^{1}H-NMR spectra of ^{4Br}L. Note: There are two imine isomers. The ratio of ^{4Br}L^E and ^{4Br}L^Z (62:38) is calculated using the average of the integration of CH_2 peaks and CH peaks.

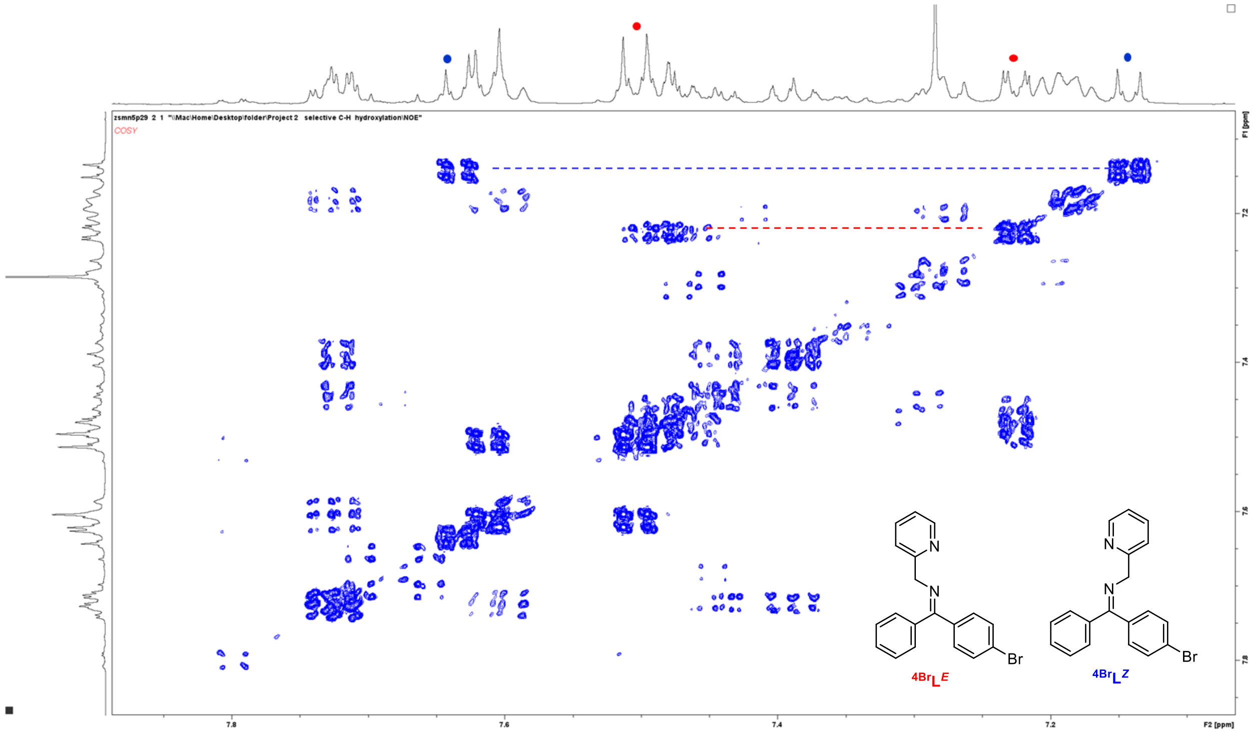

Figure S28. Homonuclear correlation spectroscopy (COSY) spectra for ^{4Br}L

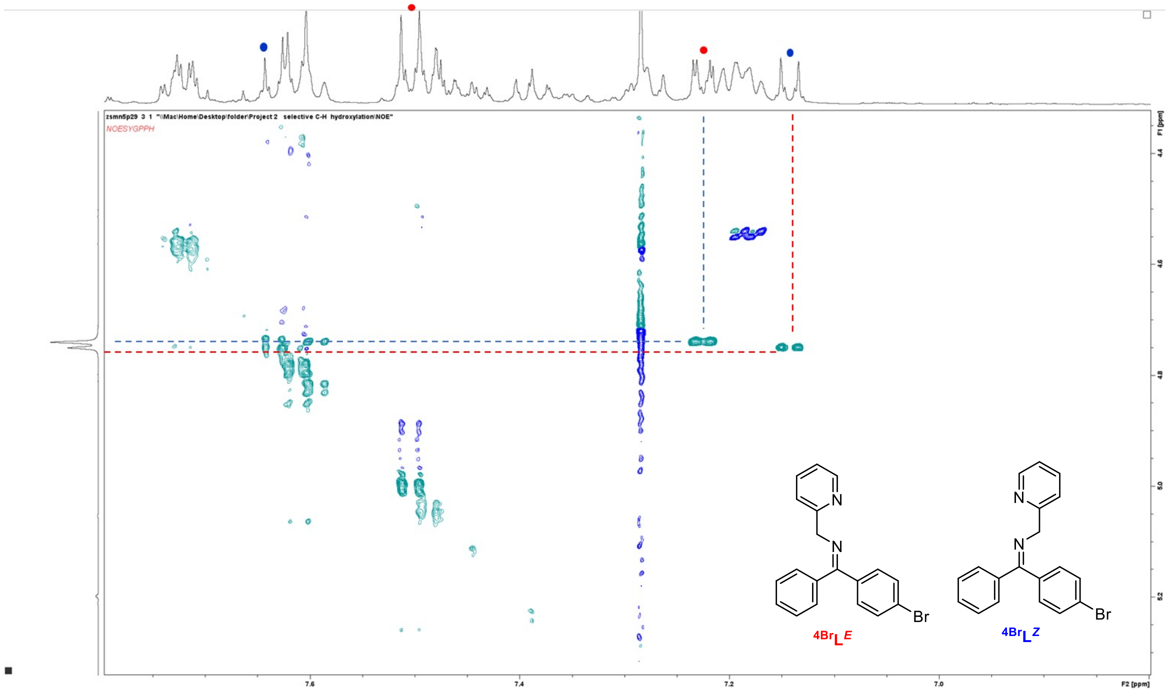

Figure S29. Nuclear Overhauser Effect Spectroscopy (NOESY) spectra for ^{4Br}L

^{1}*H-NMR spectra for the hydroxylation of* ^{4Br}*L*

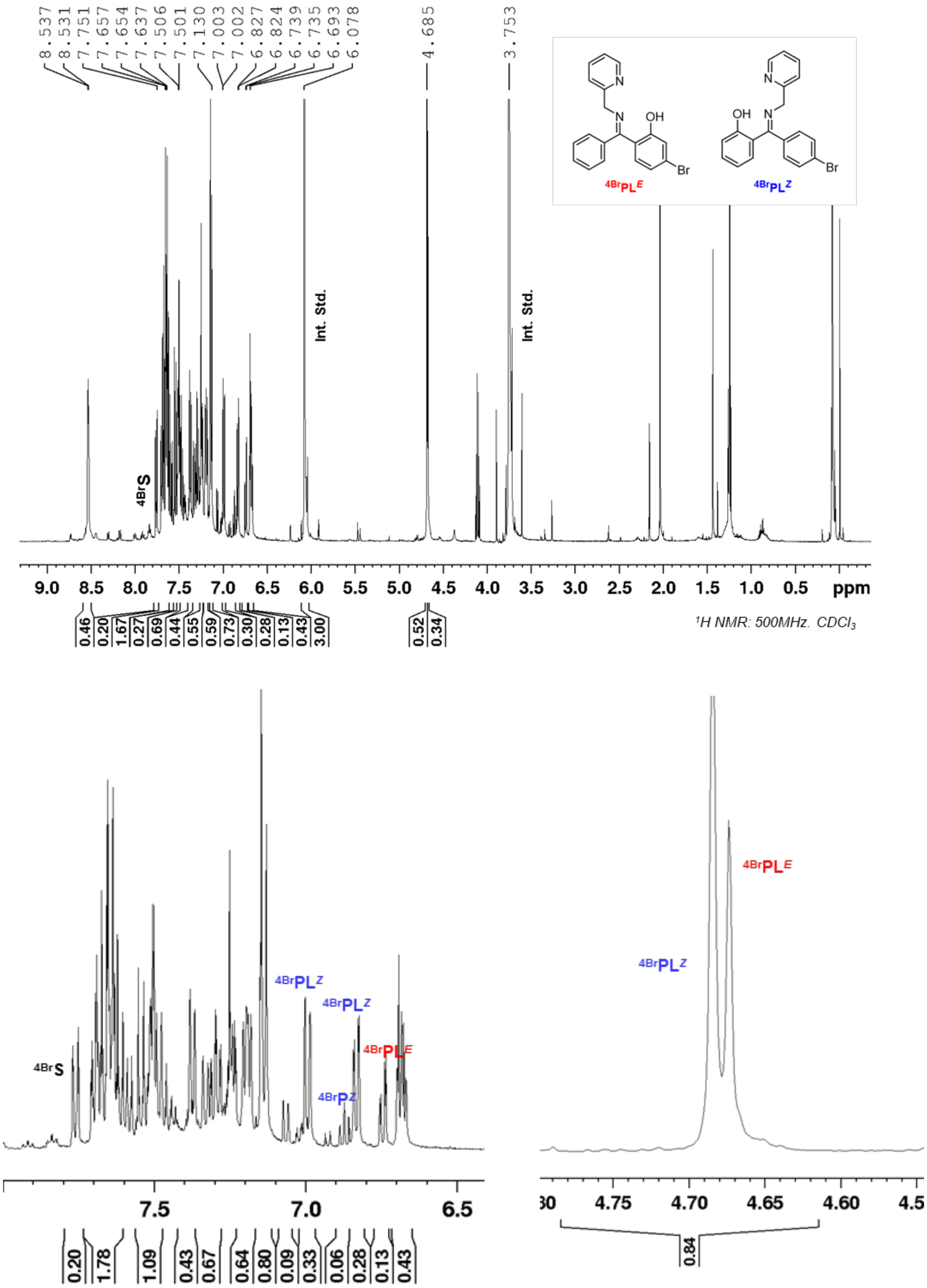

Figure S30. ^{1}H-NMR spectra for the hydroxylation of ^{4Br}L. Note: The ratio of $^{4Br}PL^{E}$ and $^{4Br}PL^{Z}$ (30/70) is calculated using the average of the integration of CH_2 peaks and CH peaks.

^{1}H-NMR spectra for the cleavage of [4Br]PL

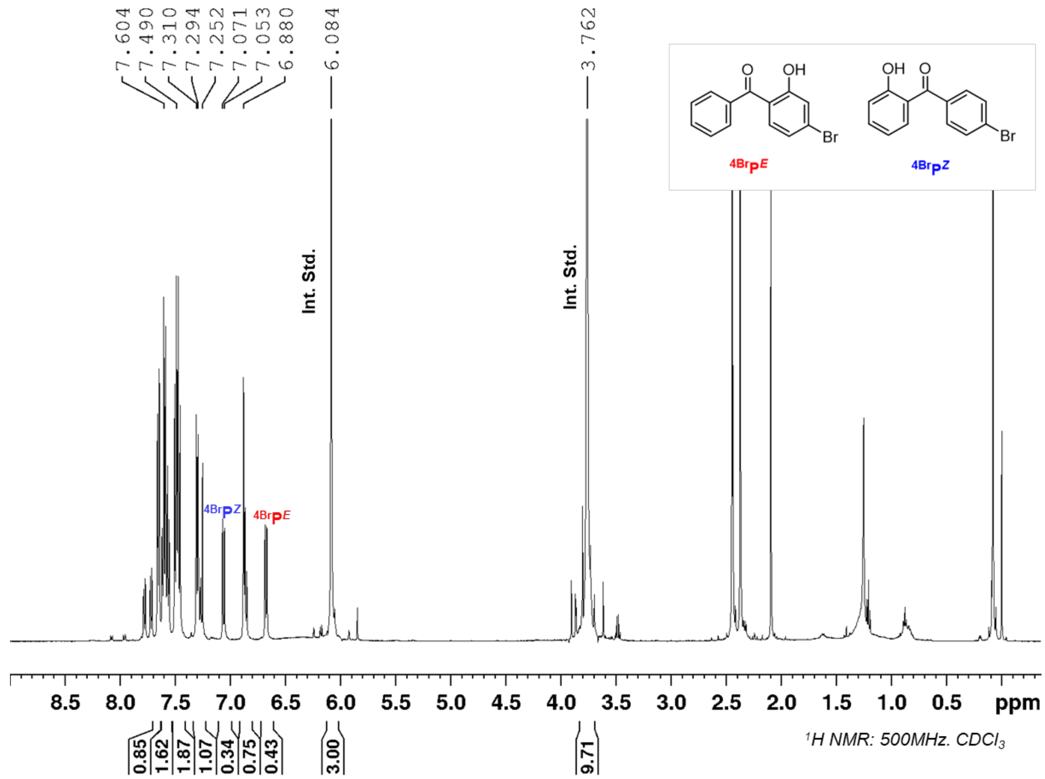

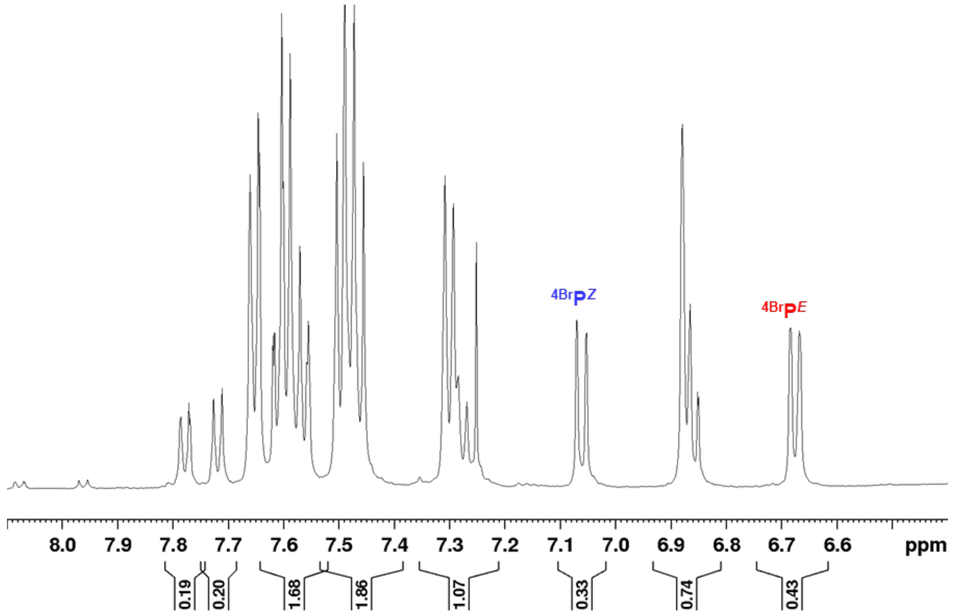

Figure S31. ^{1}H-NMR spectra for the cleavage of ^{4Br}PL. Note: The ratio of $^{4Br}P^E$ and $^{4Br}P^Z$ (56/44) is calculated using the average of the integration of CH peaks. (Note: the ratio calculated differs from the one obtained before DG cleavage. We believe this might be due to the decomposition of the hydroxylation products during the cleavage work-up or the poor mass balance of the DG removal).

3.11 ^{4CF3}S and 2-picolyamine

Synthesis of ^{4CF3}L

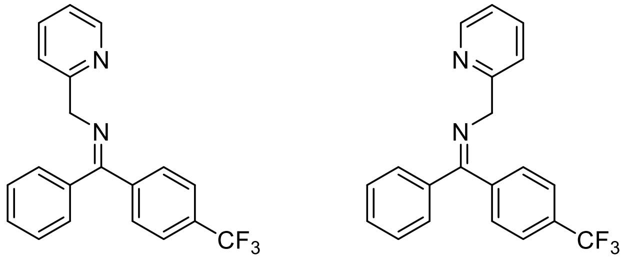

^{4CF3}L *E* ^{4CF3}L *Z*

In an oven dried flask, 2-picolylamine (2.2 equiv., 0.50 mL) was added to 4-Methoxybenzophenone (0.75 g, 2.46 mmol) and p- toluenesulfonic acid monohydrate (cat. 20 mg, 4.7 mol%) in toluene (50 mL). The reaction mixture was refluxed under argon with a Dean-Stark apparatus until imine formation was complete (4 days). The reaction was cooled to room temperature and diluted with diethyl ether (30 mL). The organic layer was washed with saturated ammonia chloride (20 mL x 2), saturated aqueous sodium bicarbonate (20 mL), brine (20 mL), and dried with magnesium sulfate. The final product was isolated as a brown solid (91% yield, 0.75 g, 85% pure). ^{1}H-NMR (500 MHz, $CDCl_3$): δ 8.54 (d, L^E+L^Z, 2H), 7.85 (d, L^E+L^Z, 4H), 7.75-7.65 (m, L^E+L^Z , 4H) 7.59-7.42 (m, L^E+L^Z, 8H), 7.40-7.20 (m, L^E+L^Z, 8H), 4.69 (s, L^Z, 2H), 4.61 (s, L^E, 2H). HRMS (ESI) m/z: [M + Na]+ Calcd for C20H15F3N2Na 340.3492, found 341.2475.

Hydroxylation of ^{4CF3}L

The reaction was carried out on 0.159 mmol scale using 63.6 mg of the imine according to the Standard Procedure. The reaction products were quantified using 0.159 mmol of 1,3,5-trimethoxybenzene (int. std.). (35% yield). The identity of the hydroxylation products was confirmed by ^{1}H-NMR.

Cleavage of ^{4CF3}PL

Dissolving ^{4CF3}PL in round bottom flask with 50 mL EtOAc, then adding 100 mL 1M HCl. Reaction was going for 30 min. The resulting mixture was extracted with EtOAc (50 mL X 2). The organic phases were separated, combined, dried over $MgSO_4$, filtered, and dried under vacuum. The reaction products were dissolved in 1.4 mL of $CDCl_3$ solution containing 27.1 mg of 1,3,5-trimethoxybenzene (internal standard). The reaction products were quantified by ^{1}H-NMR using integration signals that correspond to the starting material and products with the integration signal of the internal standard.

^{1}H-NMR spectra of ^{4CF3}L

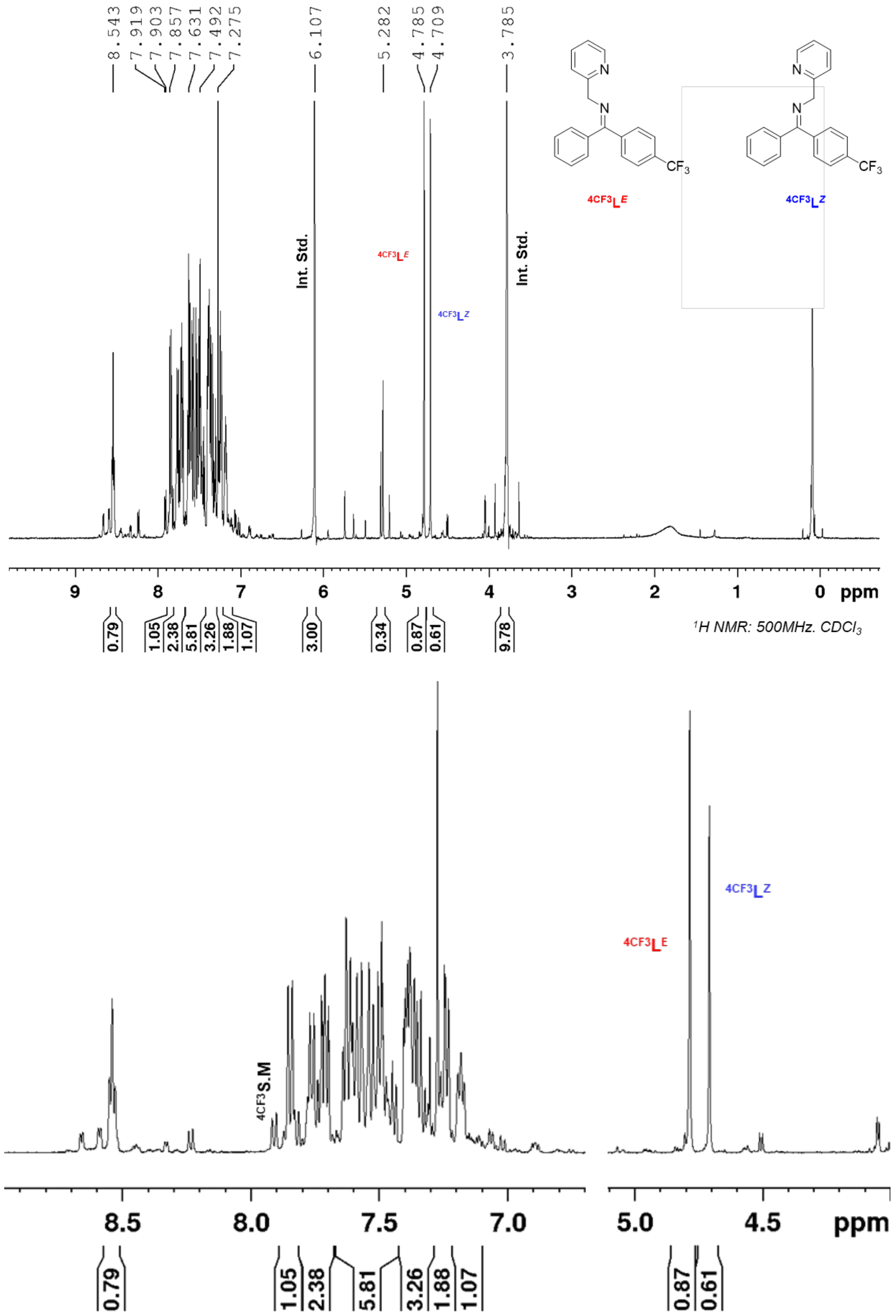

Figure S32. ^{1}H-NMR spectra of ^{4CF3}L. Note: There are two imine isomers. The ratio of $^{4CF3}L^{E}$ and $^{4CF3}L^{Z}$ (60:40) is calculated using the average of the integration of CH_2 peaks and CH peaks.

^{1}H-NMR spectra for the hydroxylation of ^{4CF3}L

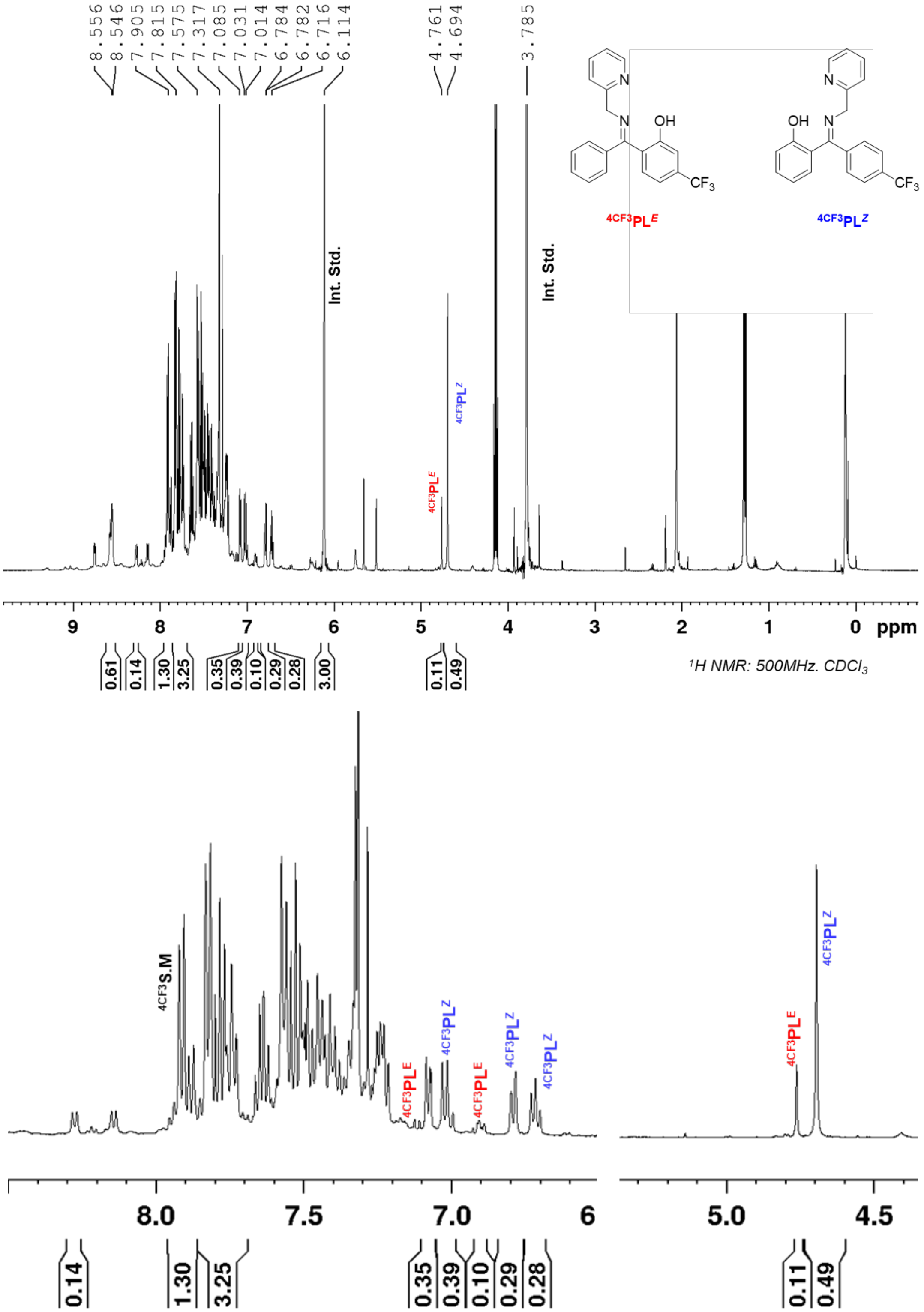

Figure S33. ^{1}H-NMR spectra for the hydroxylation of ^{4CF3}L. Note: The ratio of ${}^{4CF3}PL^{E}$ and ${}^{4CF3}PL^{Z}$ (18/82) is calculated using the average of the integration of CH_2 peaks and CH peaks.

^{1}H-NMR spectra for the cleavage ^{4CF3}PL

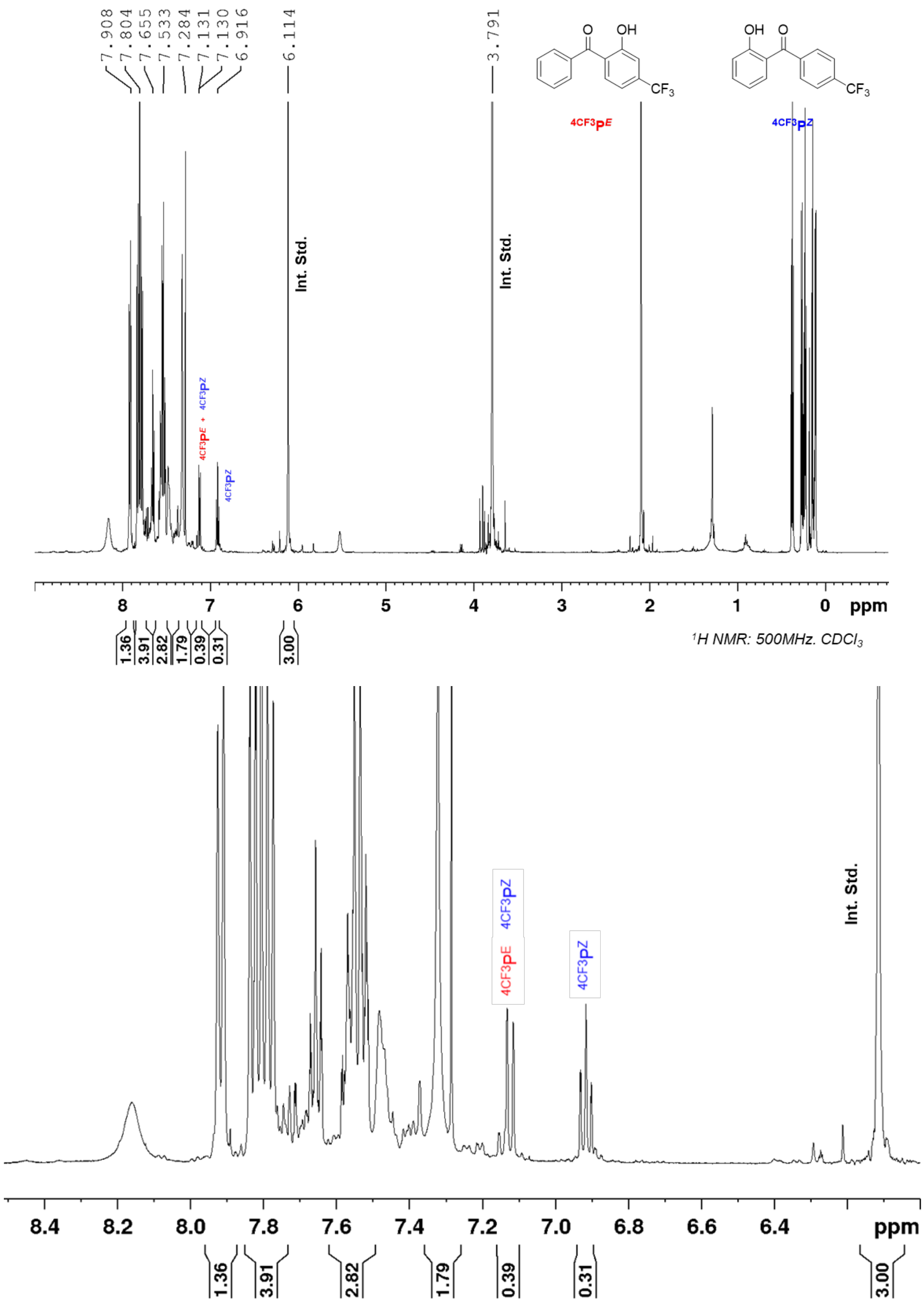

Figure S34. ^{1}H-NMR spectra for the cleavage of ^{4CF3}PL. Note: The ratio of $^{4CF3}P^{E}$ and $^{4CF3}P^{Z}$ (21/79) is calculated using the average of the integration of CH peaks.

3.12 $^{4MeO4'Me}$S and 2-picolyamine

Synthesis of $^{4MeO4'Me}$L

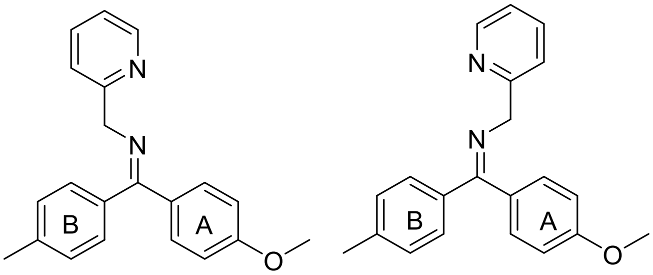

$^{4MeO4'Me}$L *A* $^{4MeO4'Me}$L *B*

In an oven dried flask, 2-picolylamine (2.2 equiv., 2.2 mL) was added to 4-Methyl-4'-methoxybenzophenone (1.97 g, 9.85 mmol) and p- toluenesulfonic acid monohydrate (cat. 20 mg, 1.2 mol%) in toluene (50 mL). The reaction mixture was refluxed under argon with a Dean-Stark apparatus until imine formation was complete (3 days). The reaction was cooled to room temperature and diluted with diethyl ether (30 mL). The organic layer was washed with saturated ammonia chloride (20 mL x 2), saturated aqueous sodium bicarbonate (20 mL), brine (20 mL), and dried with magnesium sulfate. The final product was isolated as a brown solid (85% yield, 2.65 g, 85% pure). ^{1}H-NMR (500 MHz, $CDCl_3$): δ 8.52 (s, L^A+L^B, 2H), 7.70 (d, L^A+L^B, 4H), 7.68(td, L^A+L^B, 4H), 7.26 (d, L^A+L^B,4H), 7.20-7.17(m, L^A+L^B,4H), 7.11(d, 2H), 6.99(d, L^A ,2H), 6.87(d, L^B, 2H), 4.79(s, L^B ,2H), 4.73(s, L^A ,2H), 3.87(s, L^B, 3H), 3.84(s, L^A,3H), 2.39(s, L^B, 3H), 2.37(s, L^A,3H). HRMS (ESI) m/z: [M + Na]+ Calcd for C21H20N2ONa 316.4040, found 317.2375.

Hydroxylation of $^{4MeO4'Me}$L

The reaction was carried out on 0.159 mmol scale using 59.6 mg of the imine according to the Standard Procedure. The reaction products were quantified using 0.159 mmol of 1,3,5-trimethoxybenzene (int. std.). (56% yield). The identity of the hydroxylation products was confirmed by ^{1}H-NMR.

Cleavage of $^{4MeO4'Me}$PL

Dissolving $^{4MeO4'Me}$PL in round bottom flask with 50 mL EtOAc, then adding 100 mL 1M HCl. Reaction was going for 30 min. The resulting mixture was extracted with EtOAc (50 mL X 2). The organic phases were separated, combined, dried over $MgSO_4$, filtered, and dried under vacuum. The reaction products were dissolved in 1.4 mL of $CDCl_3$ solution containing 27.1 mg of 1,3,5-trimethoxybenzene (internal standard). The reaction products were quantified by ^{1}H-NMR using integration signals that correspond to the starting material and products with the integration signal of the internal standard.

^{1}H NMR spectra of $^{4MeO4'Me}$L

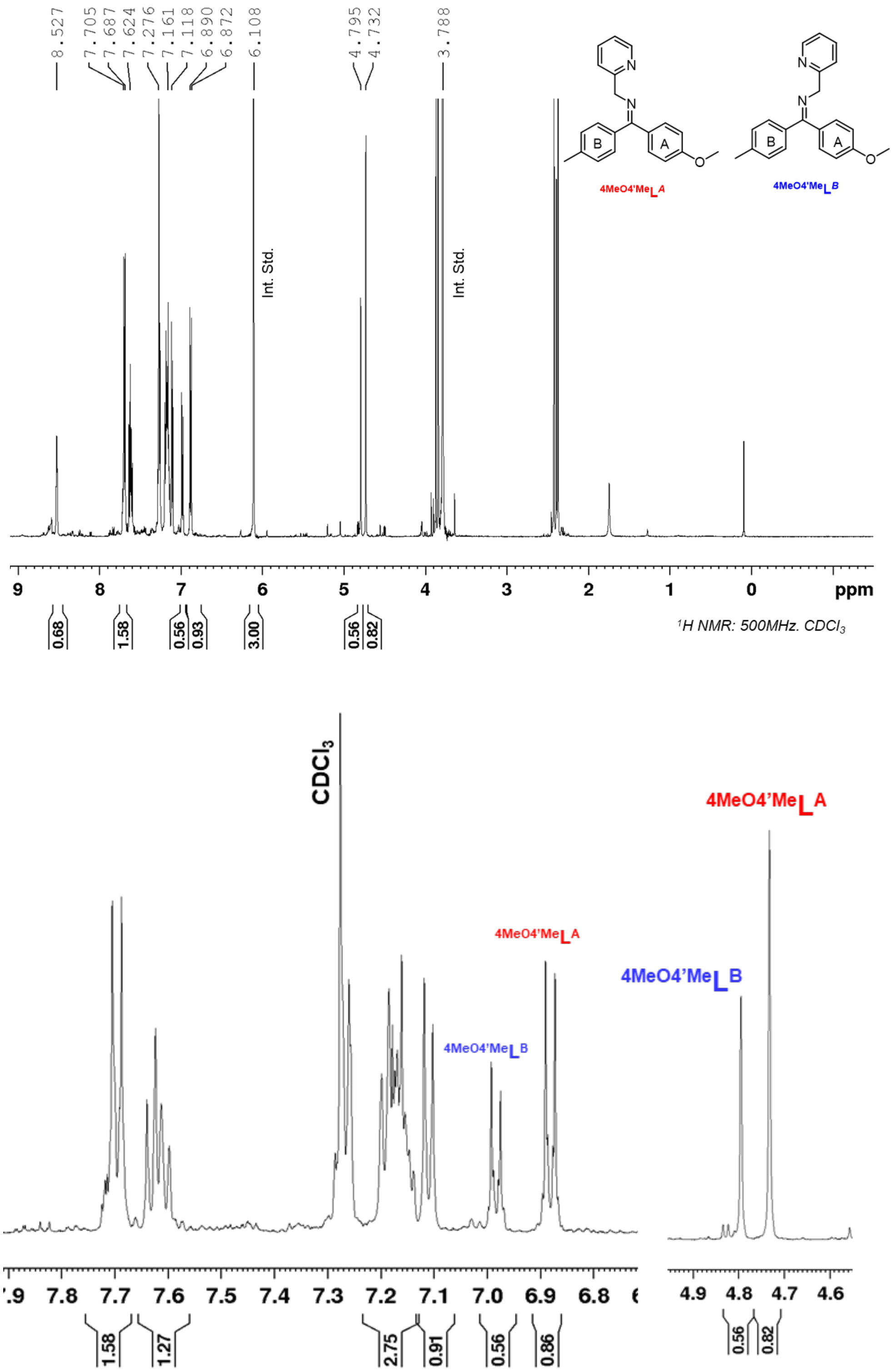

Figure S35. ^{1}H-NMR spectra of $^{4MeO4'Me}$L. Note: There are two imine isomers. The ratio of $^{4MeO4'Me}L^{A}$ and $^{4MeO4'Me}L^{B}$ (62/38) is calculated using the average of the integration of CH_2 peaks and CH peaks.

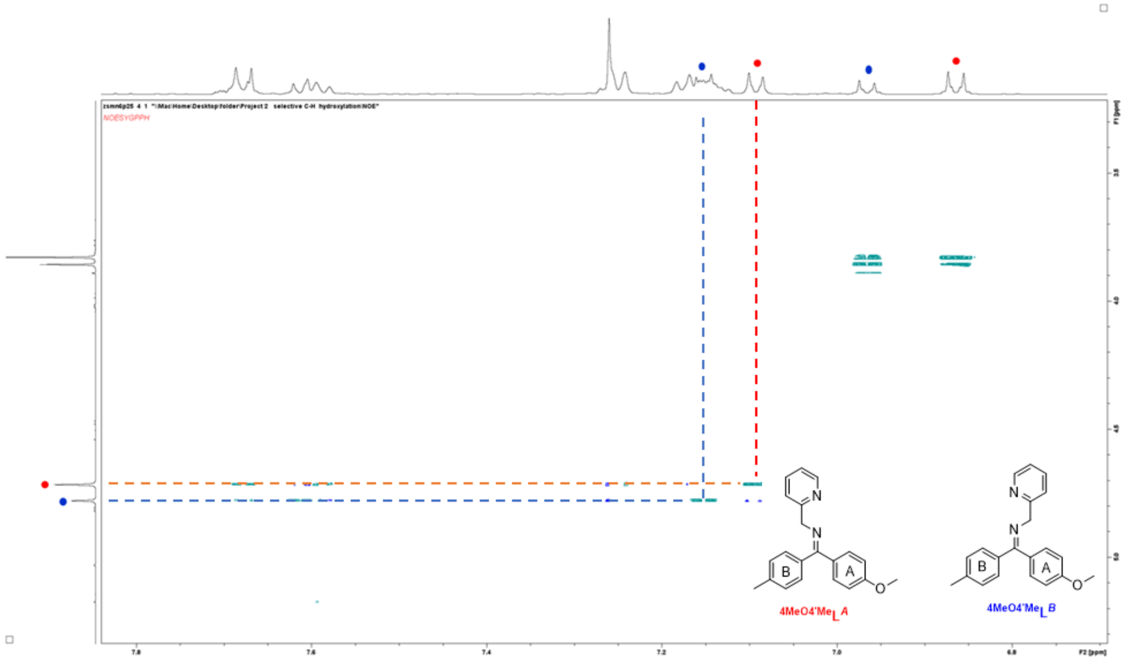

Figure S36. Nuclear Overhauser Effect Spectroscopy (NOESY) spectra for $^{4MeO4'Me}L$

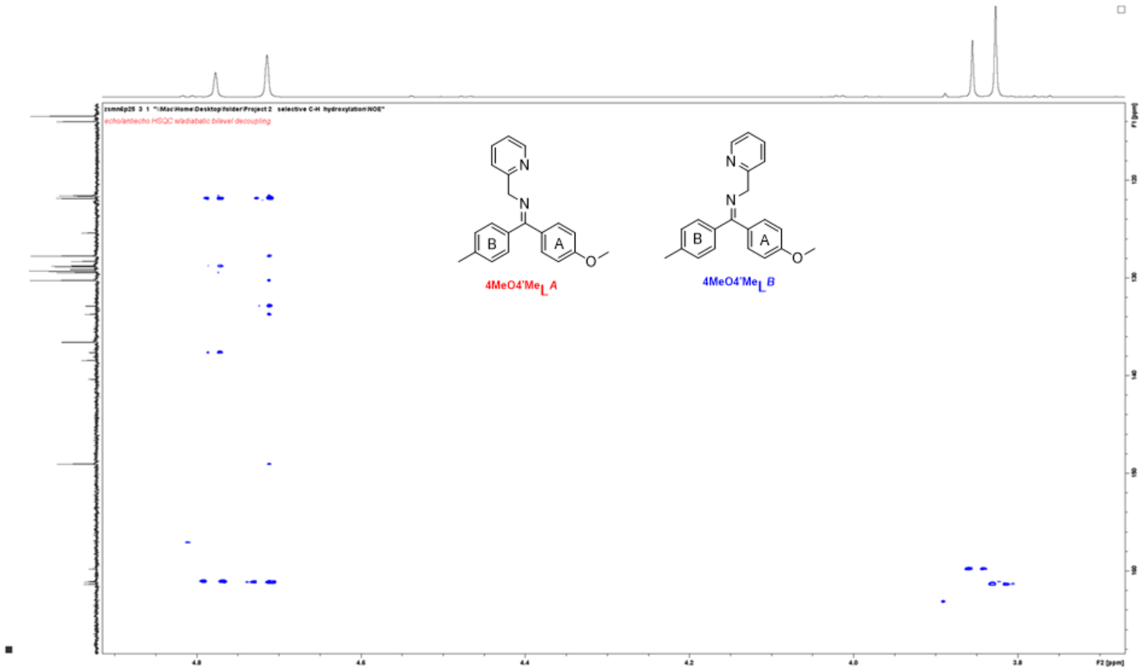

Figure S37. Heteronuclear Multiple Bond Correlation (HMBC) spectra for $^{4MeO4'Me}L$

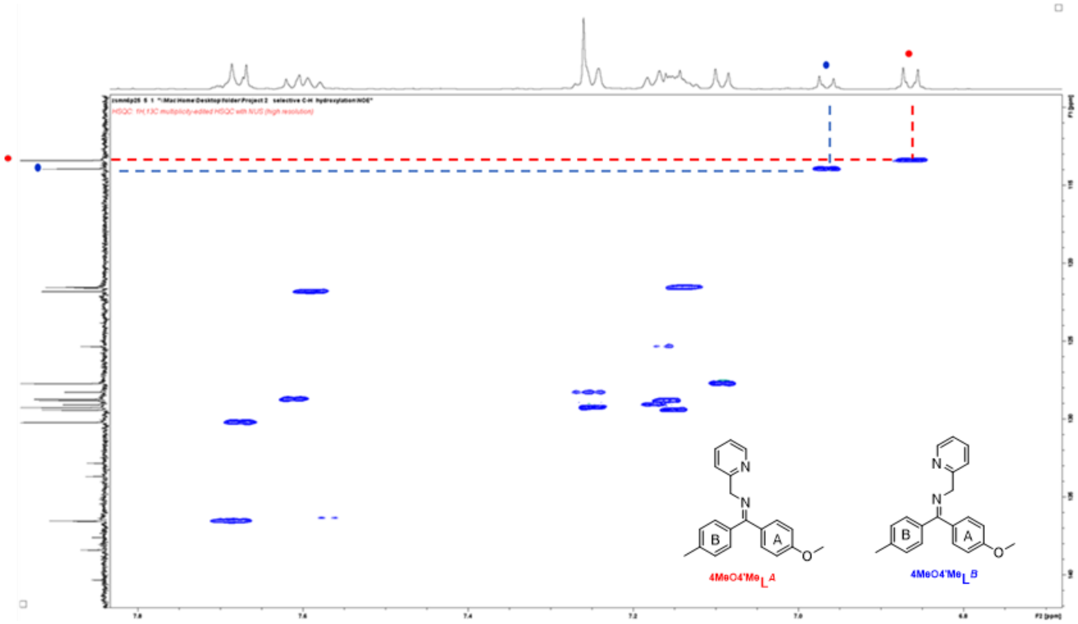

Figure S38. Heteronuclear Single-Quantum Correlation Spectroscopy (HSQC) spectra for $^{4MeO4'Me}L$

^{1}H-NMR spectra for the hydroxylation of $^{4MeO4'Me}$L

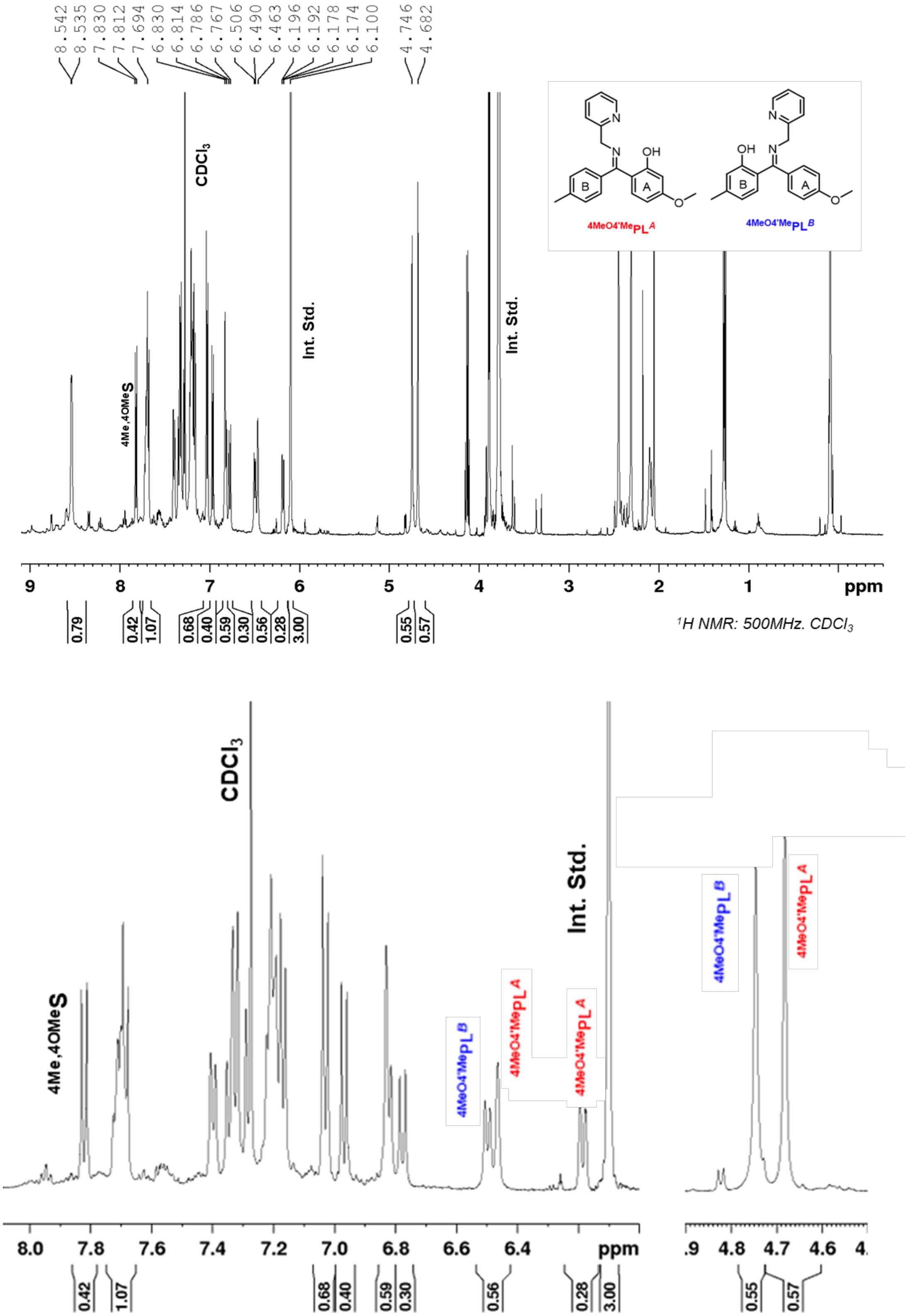

Figure 39. ^{1}H-NMR spectra for the hydroxylation of $^{4MeO4'Me}$L. Note: The ratio of $^{4MeO4'Me}$PL^{A} and $^{4MeO4'Me}$PL^{B} (53/47) is calculated using the average of the integration of CH_2 peaks and CH peaks.

^{1}H-NMR spectra for the cleavage of ${}^{4MeO4'Me}$PL

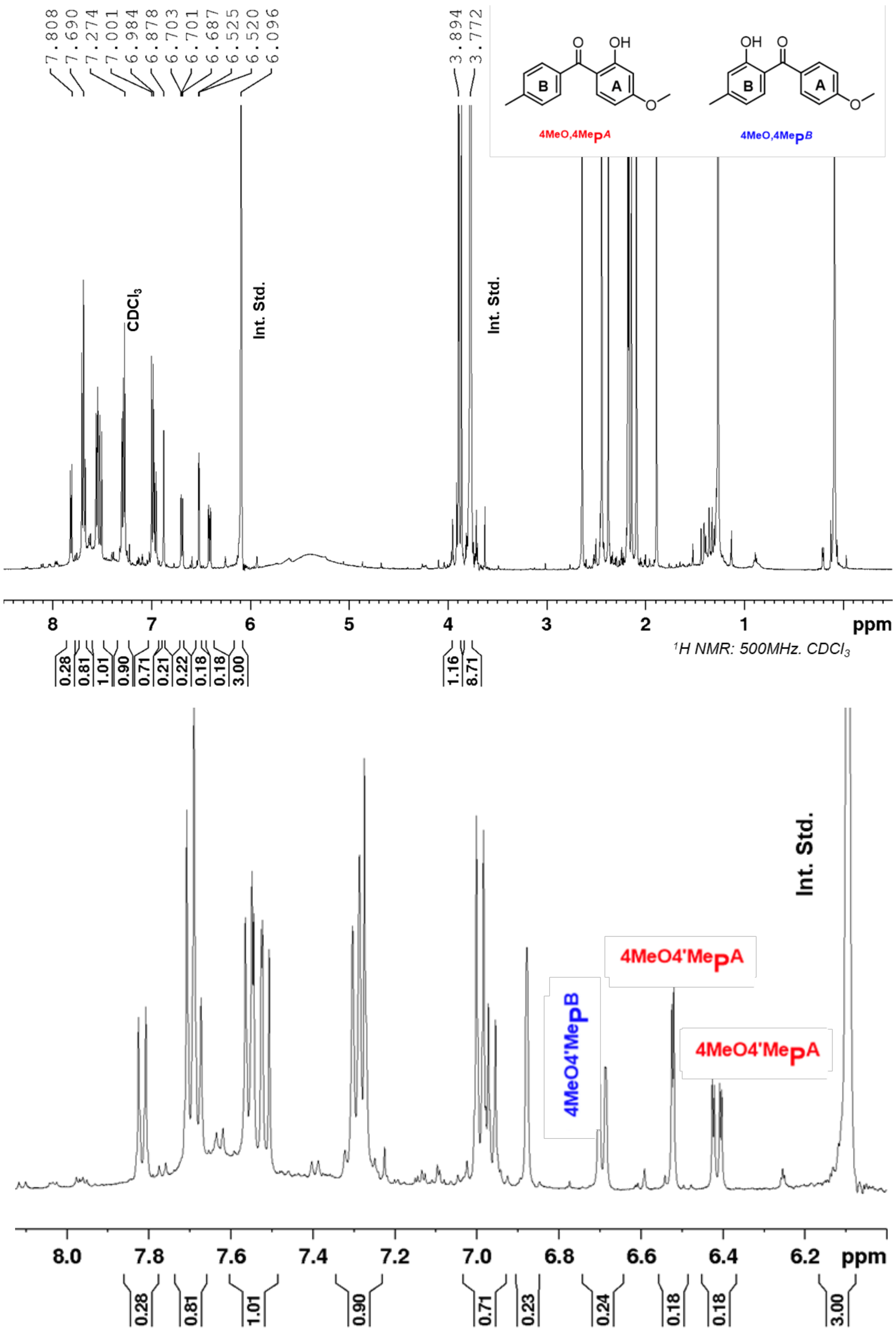

Figure S40. ^{1}H-NMR spectra for the cleavage of ${}^{4MeO4'Me}$PL. Note: The ratio of ${}^{4MeO4'Me}$P^{A} and ${}^{4MeO4'Me}$P^{B} (44/56) is calculated using the average of the integration of CH peaks.

3.13 $^{4MeO4'F}$S and 2-picolyamine

Synthesis of $^{4MeO4'F}$L

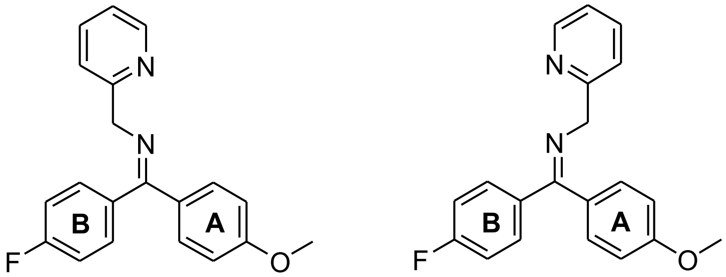

$^{4MeO4'F}$L *A* $^{4MeO4'F}$L *B*

In an oven dried flask, 2-picolylamine (2.2 equiv., 2.2 mL) was added to 4-Fluoro-4'-methoxybenzophenone (2.30 g, 9.85 mmol) and p- toluenesulfonic acid monohydrate (cat. 20 mg, 1.2 mol%) in toluene (50 mL). The reaction mixture was refluxed under argon with a Dean-Stark apparatus until imine formation was complete (3 days). The reaction was cooled to room temperature and diluted with diethyl ether (30 mL). The organic layer was washed with saturated ammonia chloride (20 mL x 2), saturated aqueous sodium bicarbonate (20 mL), brine (20 mL), and dried with magnesium sulfate. The final product was isolated as a brown solid (87% yield, 2.74 g, 93% pure). ^{1}H-NMR (500 MHz, $CDCl_3$): δ 8.53 (s, L^A+L^B, 2H), 7.70 (m, L^A+L^B, 4H), 7.67(d, 2H), 7.60 (t , 2H), 7.25-7.15(m, L^A+L^B,8H), 7.05(t, 2H), 7.00(d, L^B ,2H), 6.87(d, L^A, 2H), 4.78(s, L^B ,2H), 4.71(s, L^A ,2H), 3.93(s, L^B, 3H), 3.91(s, L^A,3H). HRMS (ESI) m/z: [M + Na]+ Calcd for C20H17FN2ONa 320.3674, found 321.1573.

Hydroxylation of $^{4MeO4'F}$L

The reaction was carried out on 0.159 mmol scale using 54.7 mg of the imine according to the Standard Procedure. The reaction products were quantified using 0.159 mmol of 1,3,5-trimethoxybenzene (int. std.). (65% yield). The identity of the hydroxylation products was confirmed by ^{1}H-NMR.

Cleavage of $^{4MeO4'F}$PL

Dissolving ^{4F,4MeO}PL in round bottom flask with 50 mL EtOAc, then adding 100 mL 1M HCl. Reaction was going for 30 min. The resulting mixture was extracted with EtOAc (50 mL X 2). The organic phases were separated, combined, dried over $MgSO_4$, filtered, and dried under vacuum. The reaction products were dissolved in 1.4 mL of $CDCl_3$ solution containing 27.1 mg of 1,3,5-trimethoxybenzene (internal standard). The reaction products were quantified by ^{1}H-NMR using integration signals that correspond to the starting material and products with the integration signal of the internal standard.

1H-NMR spectra of 4MeO4'FL

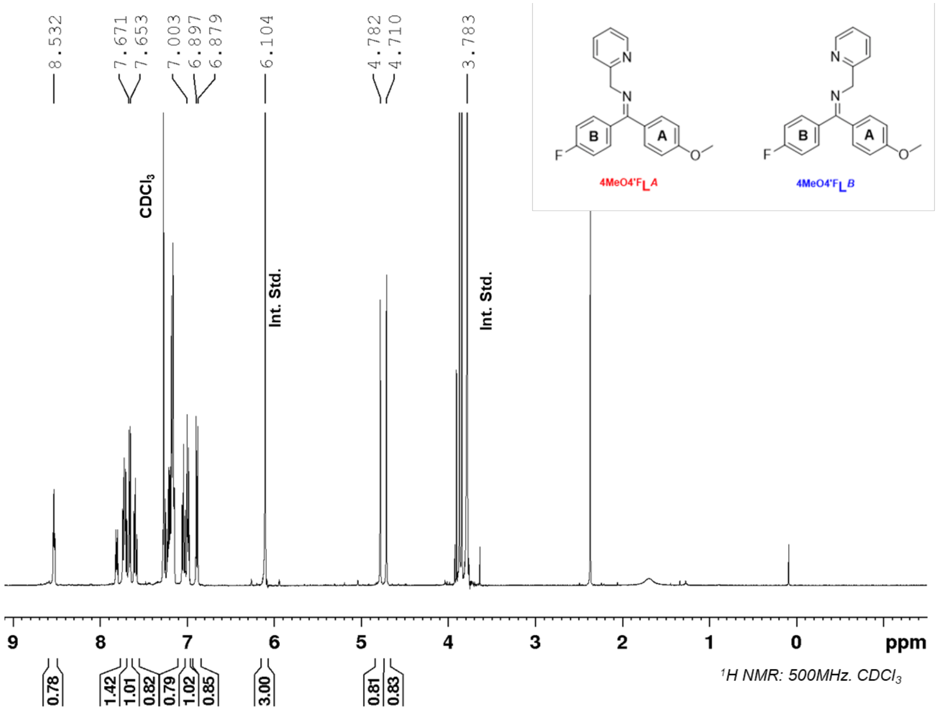

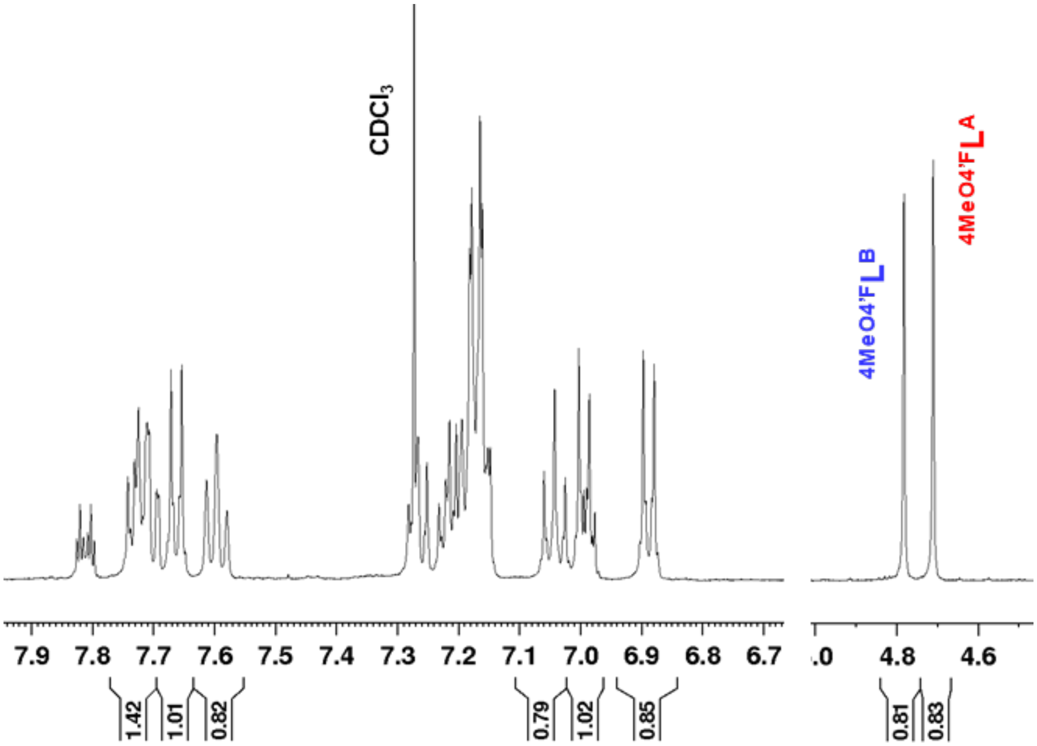

Figure S41. ^{1}H-NMR spectra of $^{4MeO4'F}$L. Note: There are two imine isomers. The ratio of $^{4MeO4'F}$L^{A} and $^{4MeO4"F}$L^{B} (51/49) is calculated using the average of the integration of CH_2 peaks and CH peaks.

^{1}H-NMR spectra for the hydroxylation of $^{4MeO,4'F}$L

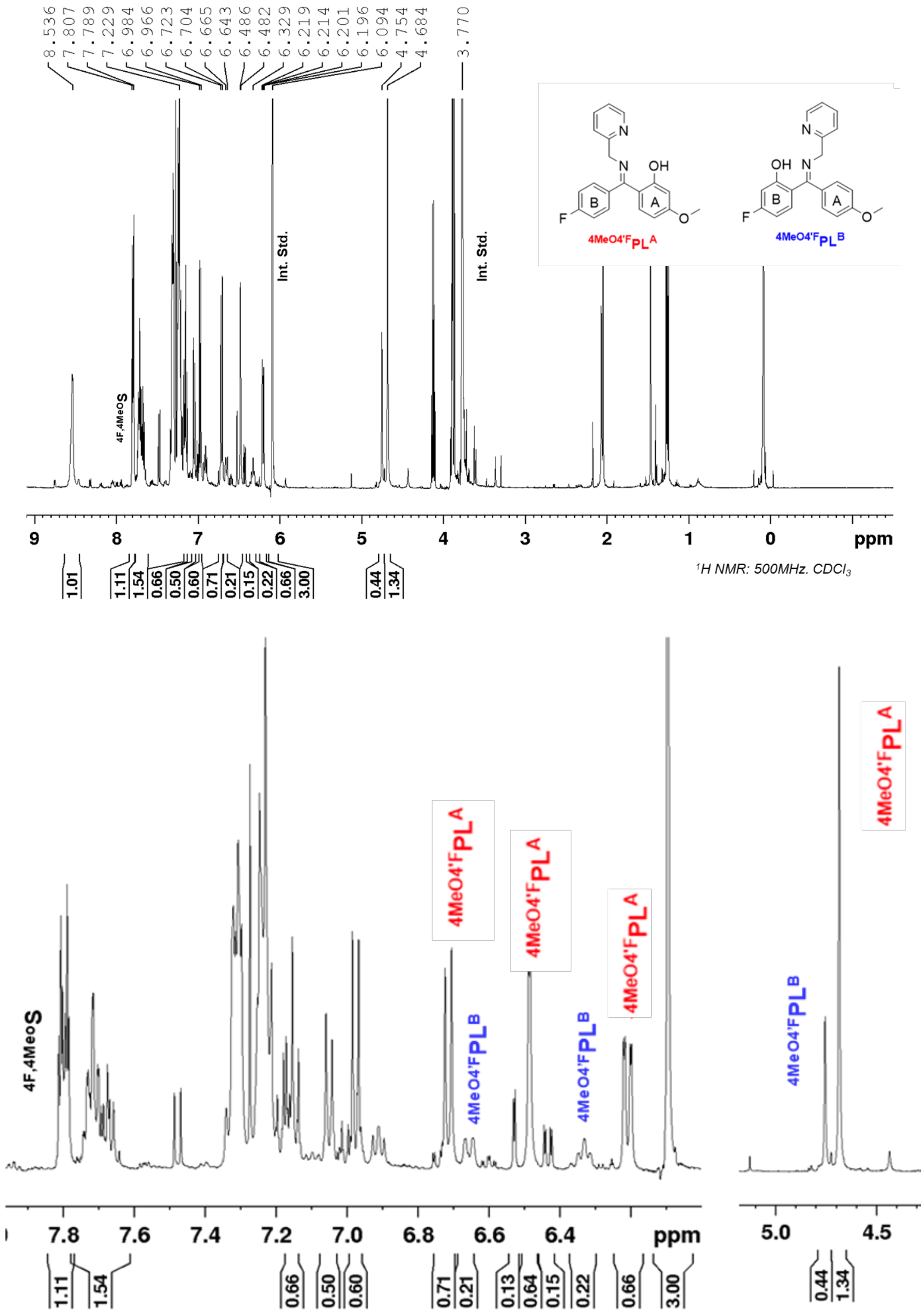

Figure S42. ^{1}H-NMR spectra for the hydroxylation of $^{4MeO4'F}$L. Note: The ratio of $^{4MeO4'F}$PL^A and $^{4MeO4'F}$PL^B (76/24) is calculated using the average of the integration of CH_2 peaks and CH peaks.

^{1}H-NMR spectra for the cleavage of $^{4MeO4'F}$PL

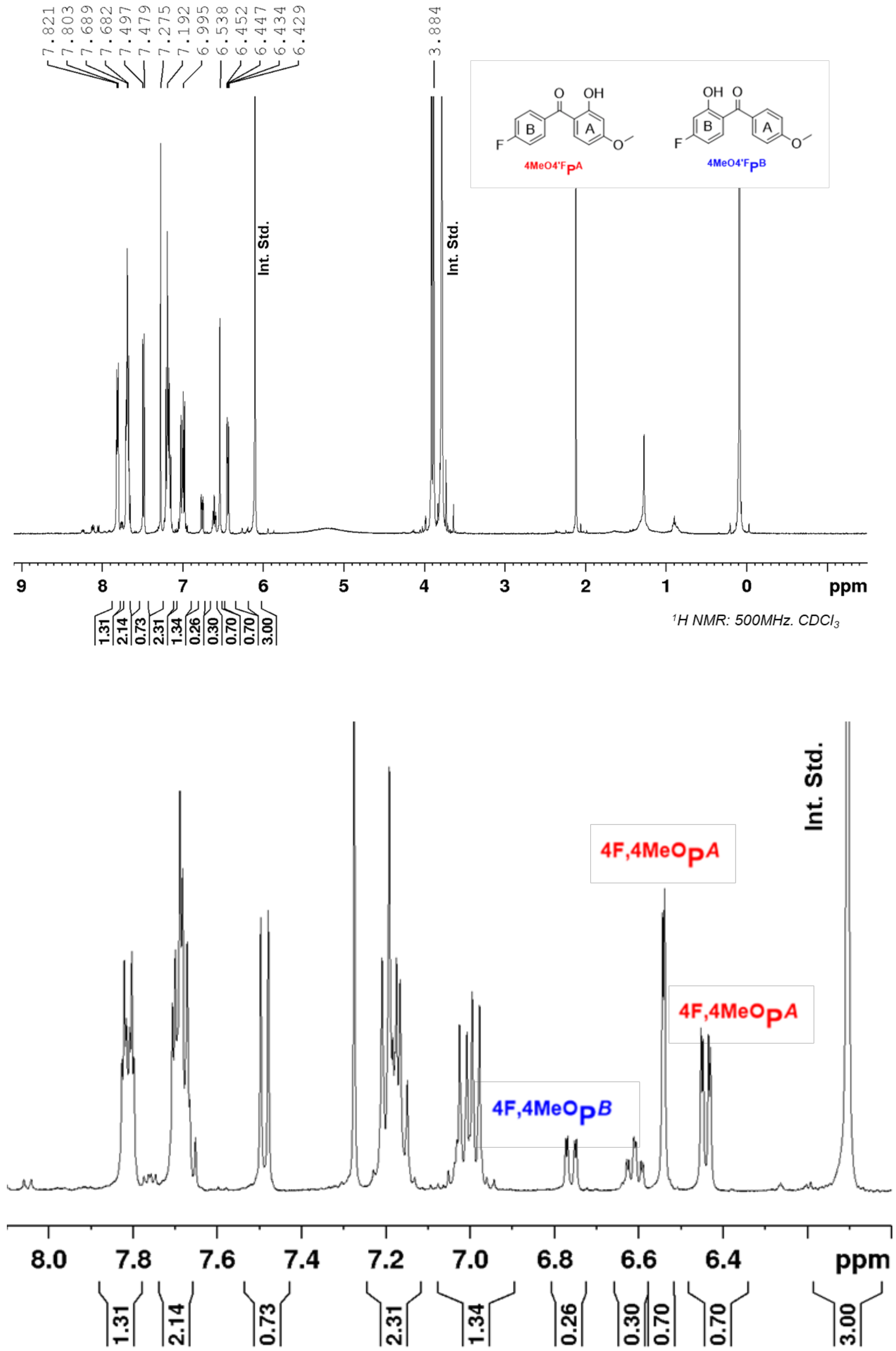

Figure S43. ^{1}H NMR spectra for the cleavage of $^{4MeO4'F}$PL. Note: The ratio of $^{4MeO4'F}$P^{A} and $^{4MeO4'F}$P^{B} (67/33) is calculated using the average of the integration of CH peaks.

3.14 $^{4MeO4'Cl}$S and 2-picolyamine

Synthesis of $^{4MeO4'Cl}$L

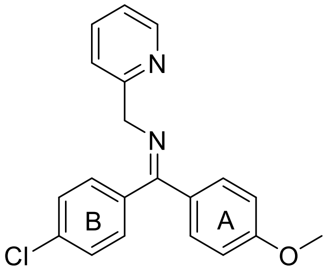

$^{4MeO4'Cl}L^{A}$

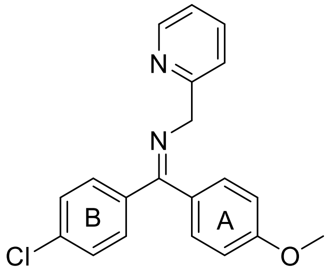

$^{4MeO4'Cl}L^{B}$

In an oven dried flask, 2-picolylamine (2.2 equiv., 0.90 mL) was added to 4-Chloro-4'-methoxybenzophenone (1.00 g, 4.06 mmol) and p- toluenesulfonic acid monohydrate (cat. 10 mg, 4.5 mol%) in toluene (50 mL). The reaction mixture was refluxed under argon with a Dean-Stark apparatus until imine formation was complete (3 days). The reaction was cooled to room temperature and diluted with diethyl ether (30 mL). The organic layer was washed with saturated ammonia chloride (20 mL x 2), saturated aqueous sodium bicarbonate (20 mL), brine (20 mL), and dried with magnesium sulfate. The final product was isolated as a brown solid (95% yield, 1.29 g, 90% pure). ^{1}H-NMR (500 MHz, $CDCl_3$): δ 8.54 (t, L^{A}+L^{B}, 2H), 7.78-7.65 (m, L^{A}+L^{B}, 5H), 7.60(t, 2H), 7.46 (d , 2H), 7.34(d,2H), 7.19(m, 7H), 7.00(d, L^{B} ,2H), 6.87(d, L^{A}, 2H), 4.79(s, L^{B} ,2H), 4.69(s, L^{A} ,2H), 3.87(s, L^{B}, 3H), 3.84(s, L^{A},3H). HRMS (ESI) m/z: [M + Na]+ Calcd for C20H17ClN2ONa 336.8190, found 337.1136.

Hydroxylation of $^{4MeO4'Cl}$L

The reaction was carried out on 0.159 mmol scale using 59.36 mg of the imine according to the Standard Procedure. The reaction products were quantified using 0.159 mmol of 1,3,5-trimethoxybenzene (int. std.). (65% yield). The identity of the hydroxylation products was confirmed by ^{1}H-NMR.

Cleavage of $^{4MeO4'Cl}$PL

Dissolving $^{4MeO4'Cl}$PL in round bottom flask with 50 mL EtOAc, then adding 100 mL 1M HCl. Reaction was going for 30 min. The resulting mixture was extracted with EtOAc (50 mL X 2). The organic phases were separated, combined, dried over $MgSO_4$, filtered, and dried under vacuum. The reaction products were dissolved in 1.4 mL of $CDCl_3$ solution containing 27.1 mg of 1,3,5-trimethoxybenzene (internal standard). The reaction products were quantified by ^{1}H-NMR using integration signals that correspond to the starting material and products with the integration signal of the internal standard.

^{1}H-NMR spectra of ${}^{4MeO4'Cl}$L

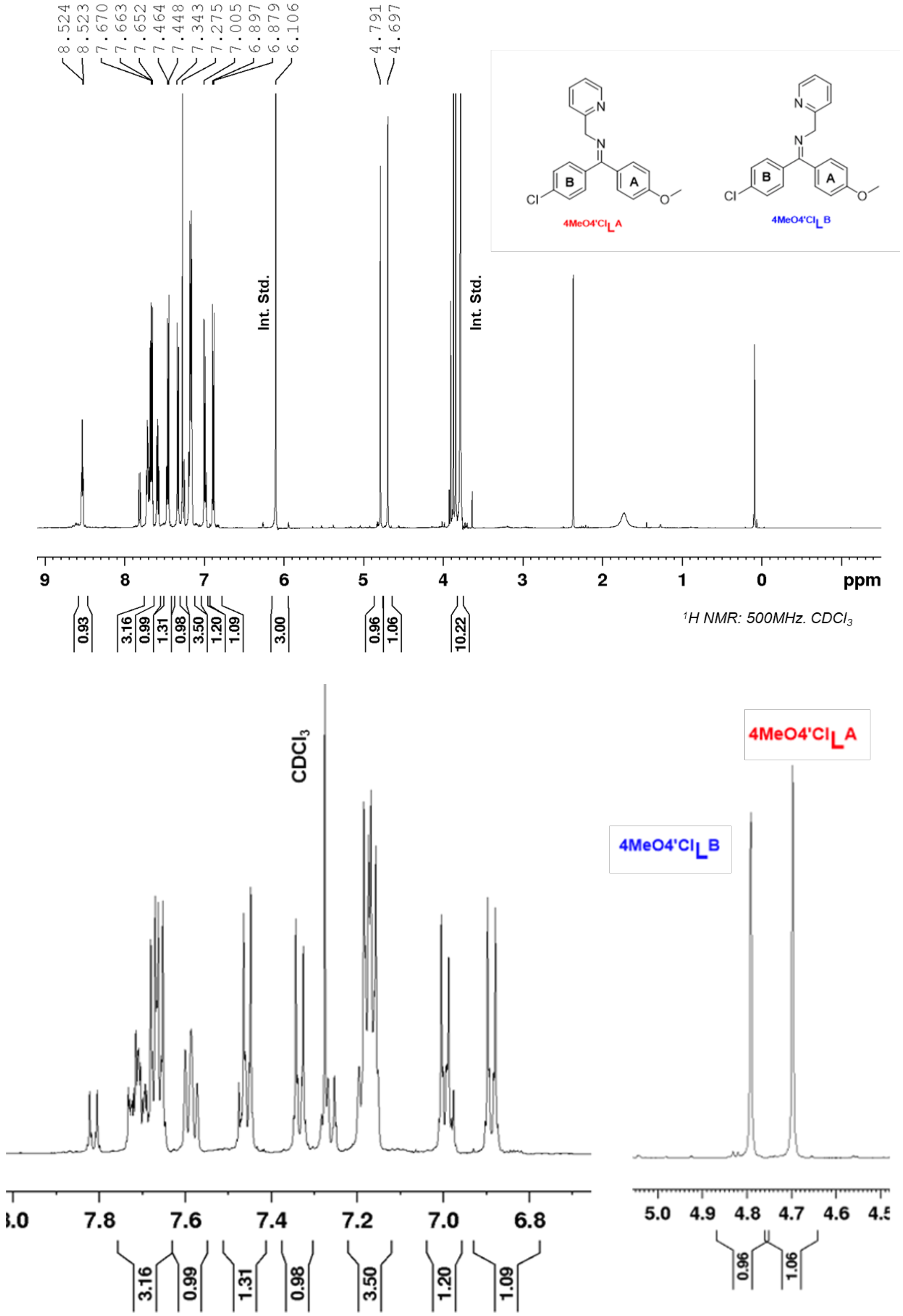

Figure S44. ^{1}H-NMR spectra of ${}^{4MeO4'Cl}$L. Note: There are two imine isomers. The ratio of ${}^{4MeO4'Cl}L^{A}$ and ${}^{4MeO4'Cl}L^{B}$ (52/48) is calculated using the average of the integration of CH_2 peaks and CH peaks.

^{1}H-NMR spectra for the hydroxylation of $^{4MeO4'Cl}$L

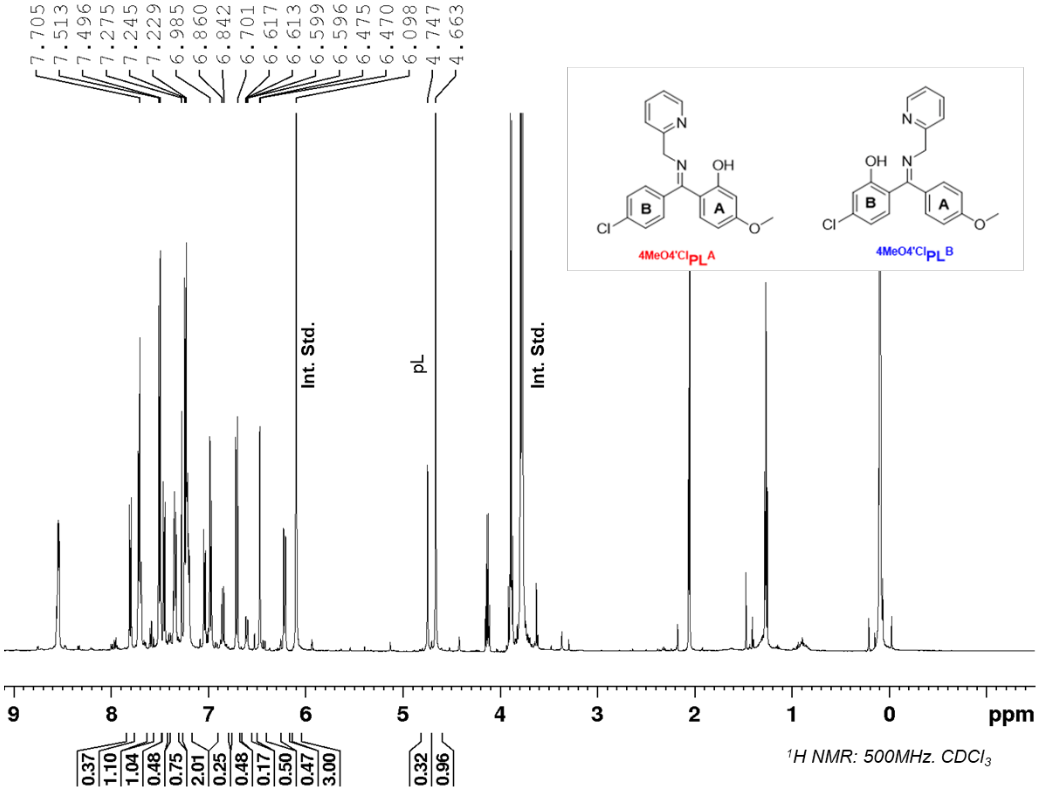

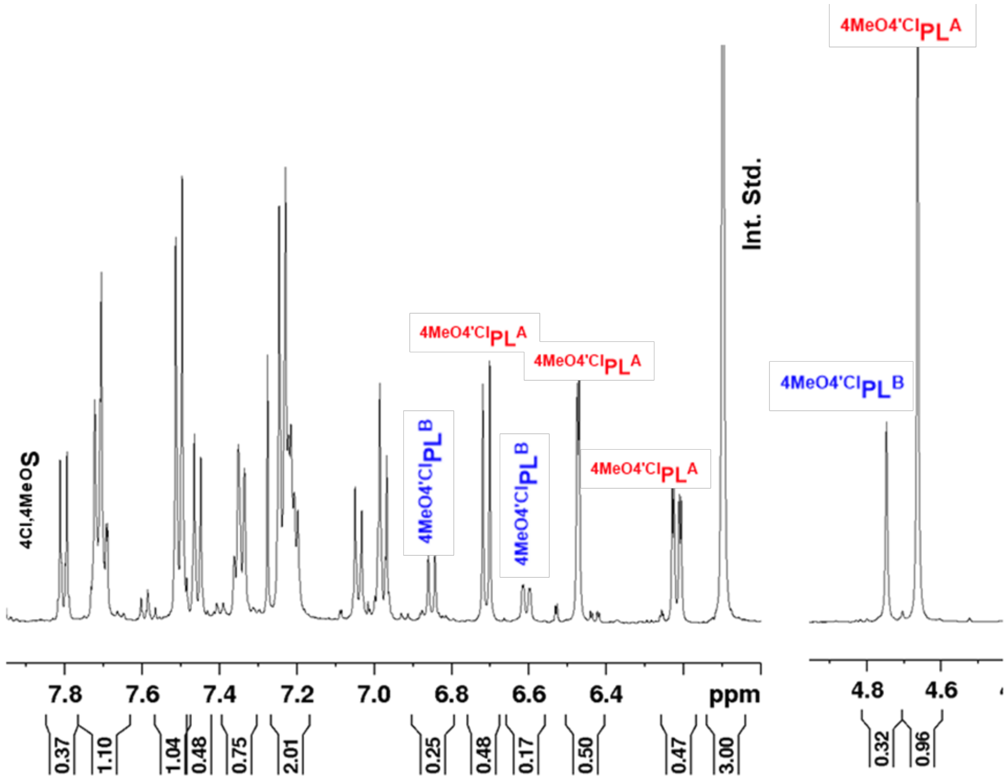

Figure S45. ^{1}H-NMR spectra for the hydroxylation of $^{4MeO4'Cl}$L. Note: The ratio of $^{4MeO4'Cl}$PL^{A} and $^{4MeO4'Cl}$PL^{B} (75/25) is calculated using the average of the integration of CH_2 peaks and CH peaks.

^{1}H-NMR spectra for the cleavage of [4MeO4'Cl]PL

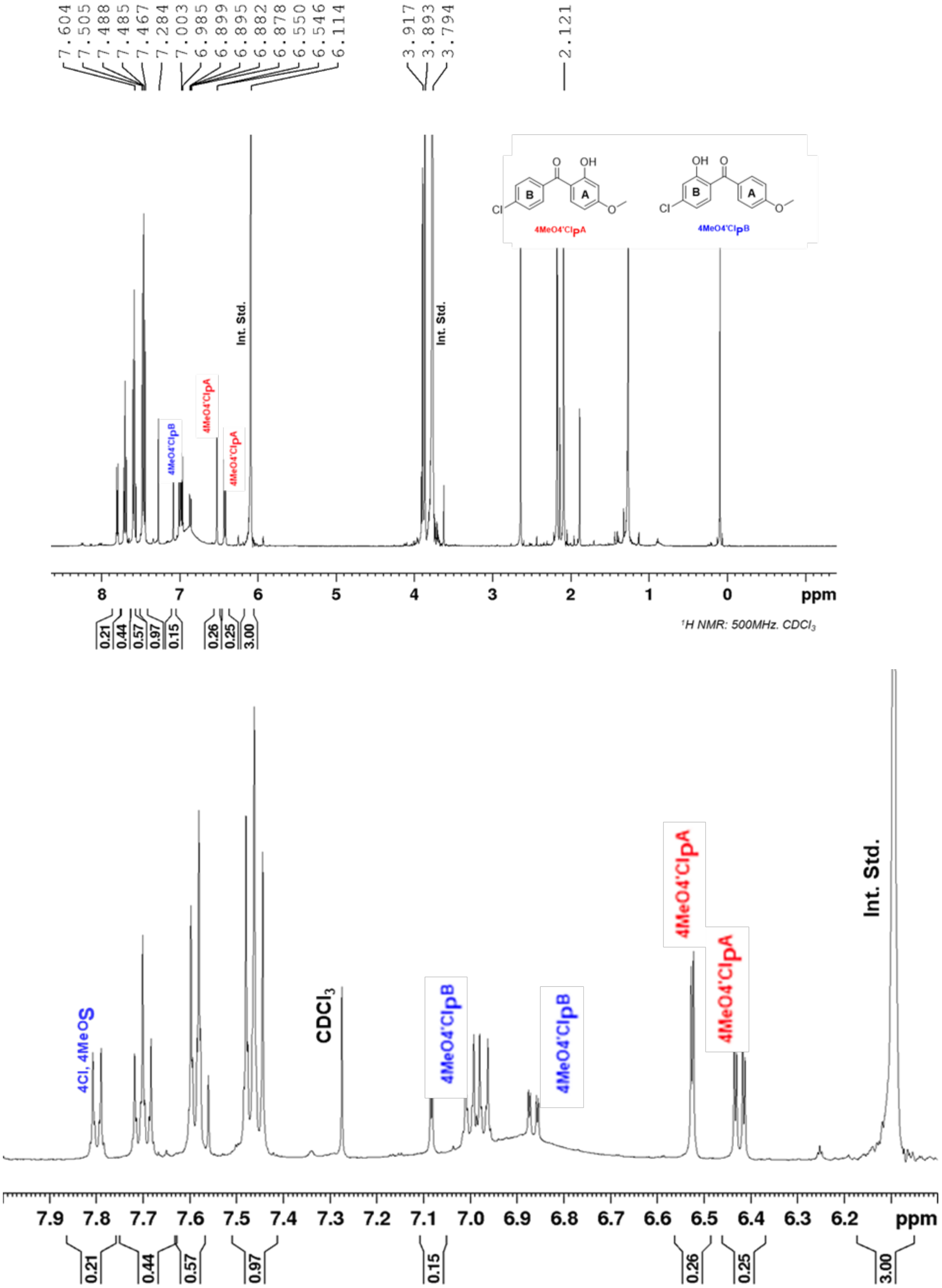

Figure S46. ^{1}H-NMR spectra for the cleavage of [4MeO4'Cl]PL. Note: The ratio of [4MeO4'Cl]P[A] and [4MeO4'Cl]P[B] (63/37) is calculated using the average of the integration of CH peaks.

3.15 $^{4MeO4'Br}$S and 2-picolyamine

Synthesis of $^{4MeO4'Br}$L

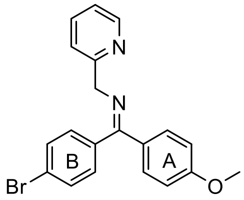

$^{4MeO4'Br}$L *A*

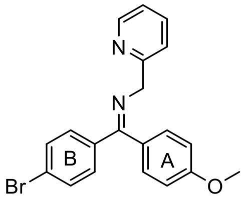

$^{4MeO4'Br}$L *B*

In an oven dried flask, 2-picolylamine (2.2 equiv., 0.76 mL) was added to 4-Bromo-4'-methoxybenzophenone (1.00 g, 3.4 mmol) and p- toluenesulfonic acid monohydrate (cat. 10 mg, 1.7 mol%) in toluene (50 mL). The reaction mixture was refluxed under argon with a Dean-Stark apparatus until imine formation was complete (3 days). The reaction was cooled to room temperature and diluted with diethyl ether (30 mL). The organic layer was washed with saturated ammonia chloride (20 mL x 2), saturated aqueous sodium bicarbonate (20 mL), brine (20 mL), and dried with magnesium sulfate. The final product was isolated as a brown solid (94% yield, 1.22 g, 97% pure). ^{1}H-NMR (500 MHz, $CDCl_3$): δ 8.53 (t, L^A+L^B, 2H), 7.72 (m, L^A+L^B, 2H), 7.66-7.58(m, L^A+L^B, 9H), 7.50 (d , 2H), 7.18(m, L^A+L^B, 3H), 7.12(d, 2H), 7.00(d, L^B ,2H), 6.87(d, L^A, 2H), 4.78(s, L^B ,2H), 4.69(s, L^A ,2H), 3.87(s, L^B, 3H), 3.84(s, L^A,3H). HRMS (ESI) m/z: [M + Na]+ Calcd for C20H17BrN2ONa 381.2730, found 381.0595.

Hydroxylation of $^{4MeO4'Br}$L

The reaction was carried out on 0.159 mmol scale using 62.4 mg of the imine according to the Standard Procedure. The reaction products were quantified using 0.159 mmol of 1,3,5-trimethoxybenzene (int. std.). (64% yield). The identity of the hydroxylation products was confirmed by ^{1}H-NMR.

¹H-NMR spectra of $^{4MeO4'Br}L$

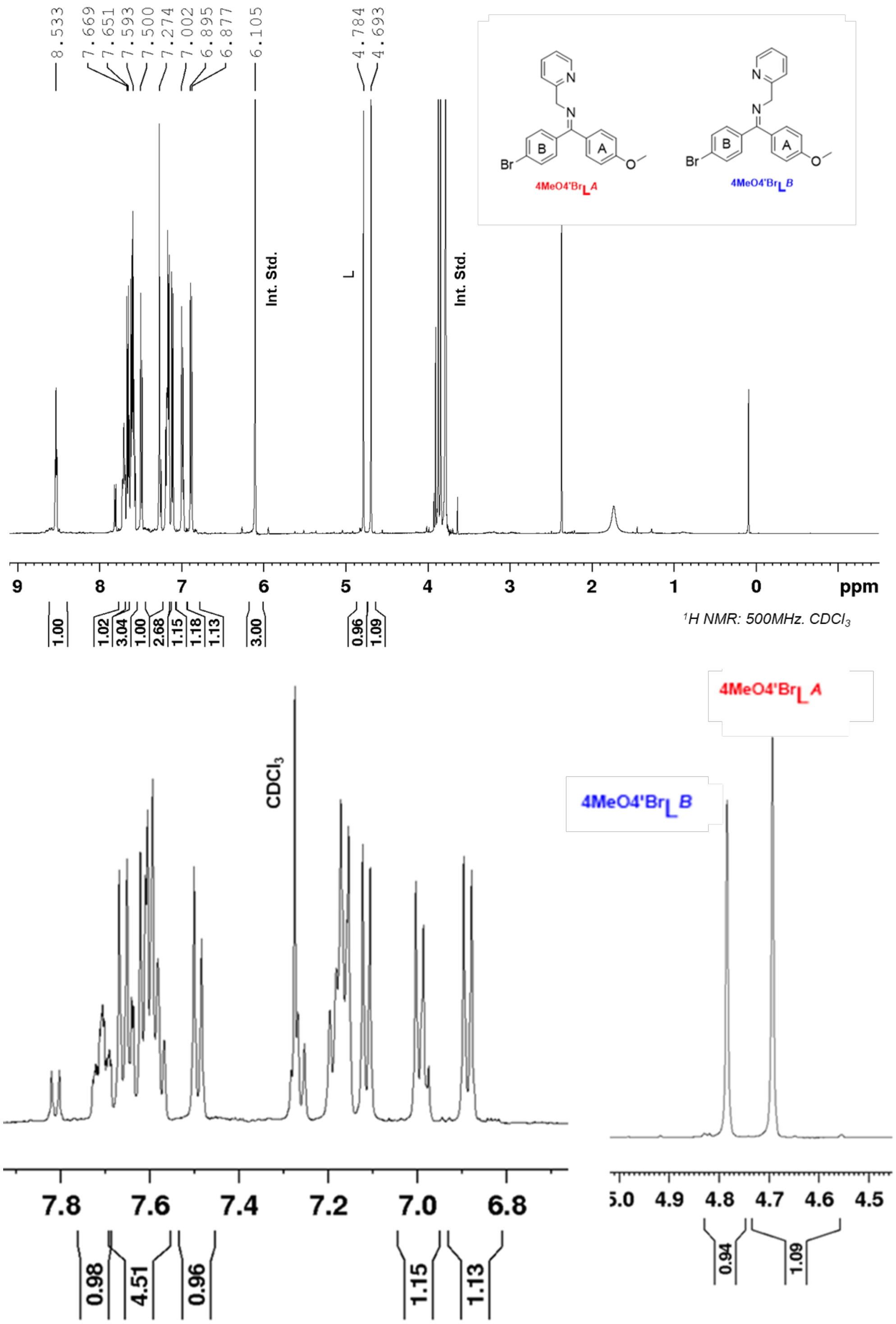

Figure S47. ¹H-NMR spectra of $^{4MeO4'Br}L$. Note: There are two imine isomers. The ratio of $^{4MeO4'Br}L^{A}$ and $^{4MeO4'Br}L^{B}$ (52/48) is calculated using the average of the integration of CH_2 peaks and CH peaks.

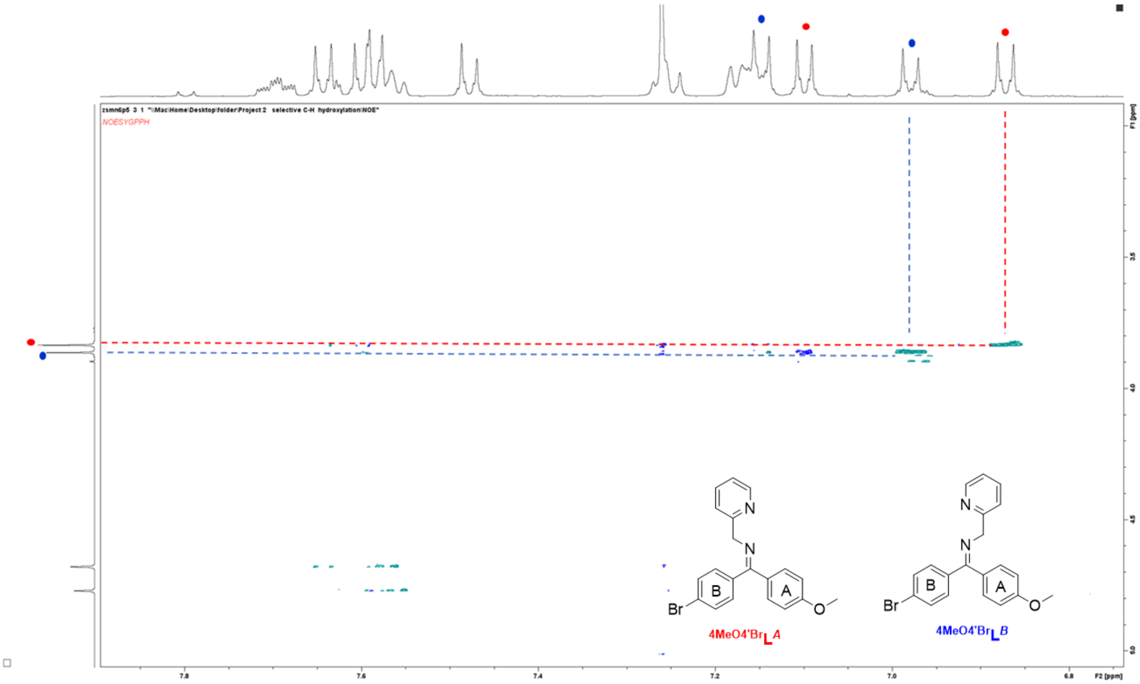

Figure S48. Nuclear Overhauser Effect Spectroscopy (NOESY) spectra for $^{4MeO4'Br}L$

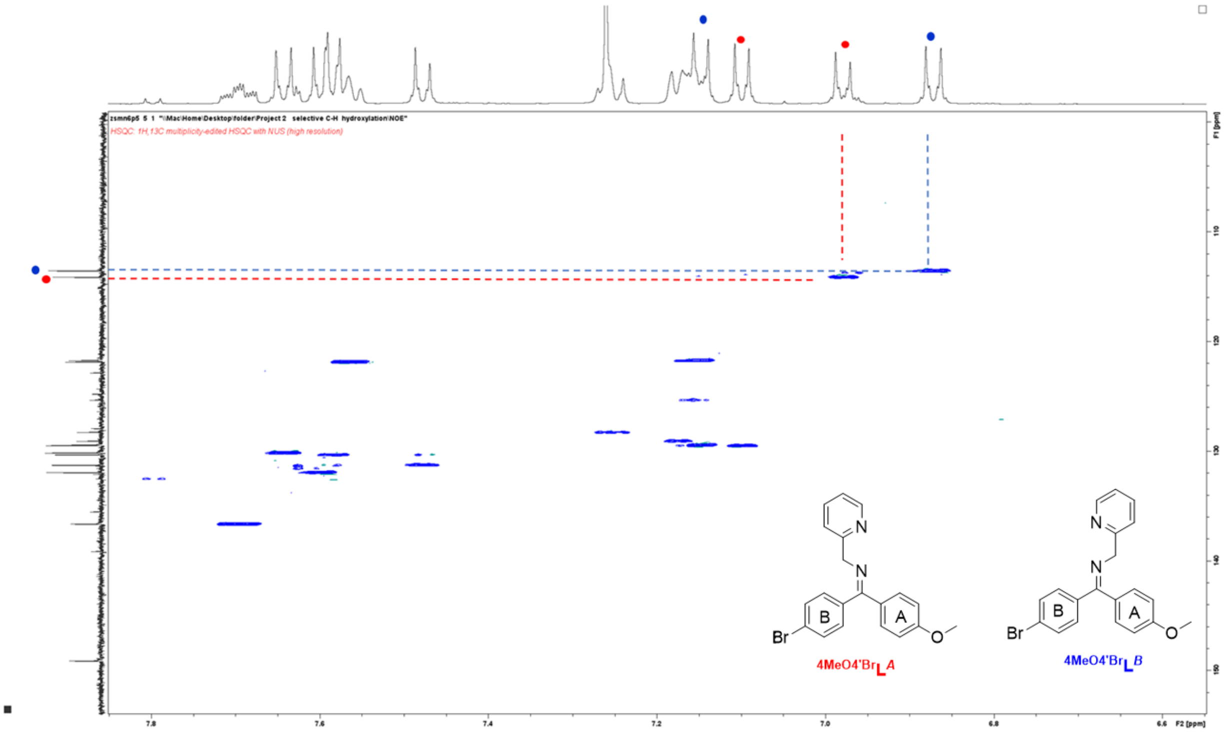

Figure S49. Heteronuclear Single-Quantum Correlation Spectroscopy (HSQC) spectra for $^{4MeO4'Br}L^{A}$

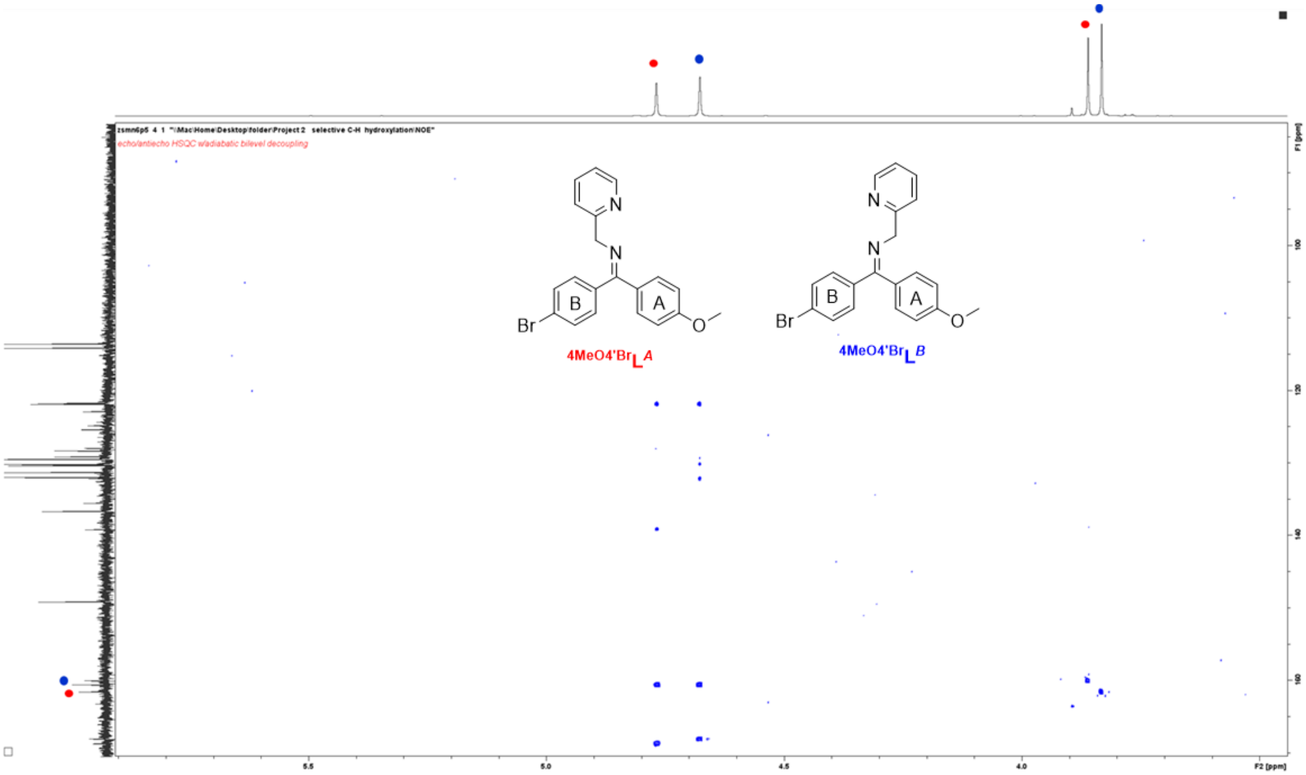

Figure S50. Heteronuclear Multiple Bond Correlation (HMBC) spectra for $^{4MeO4'Br}L$

^{1}H-NMR spectra for the hydroxylation of $^{4MeO4'Br}$L

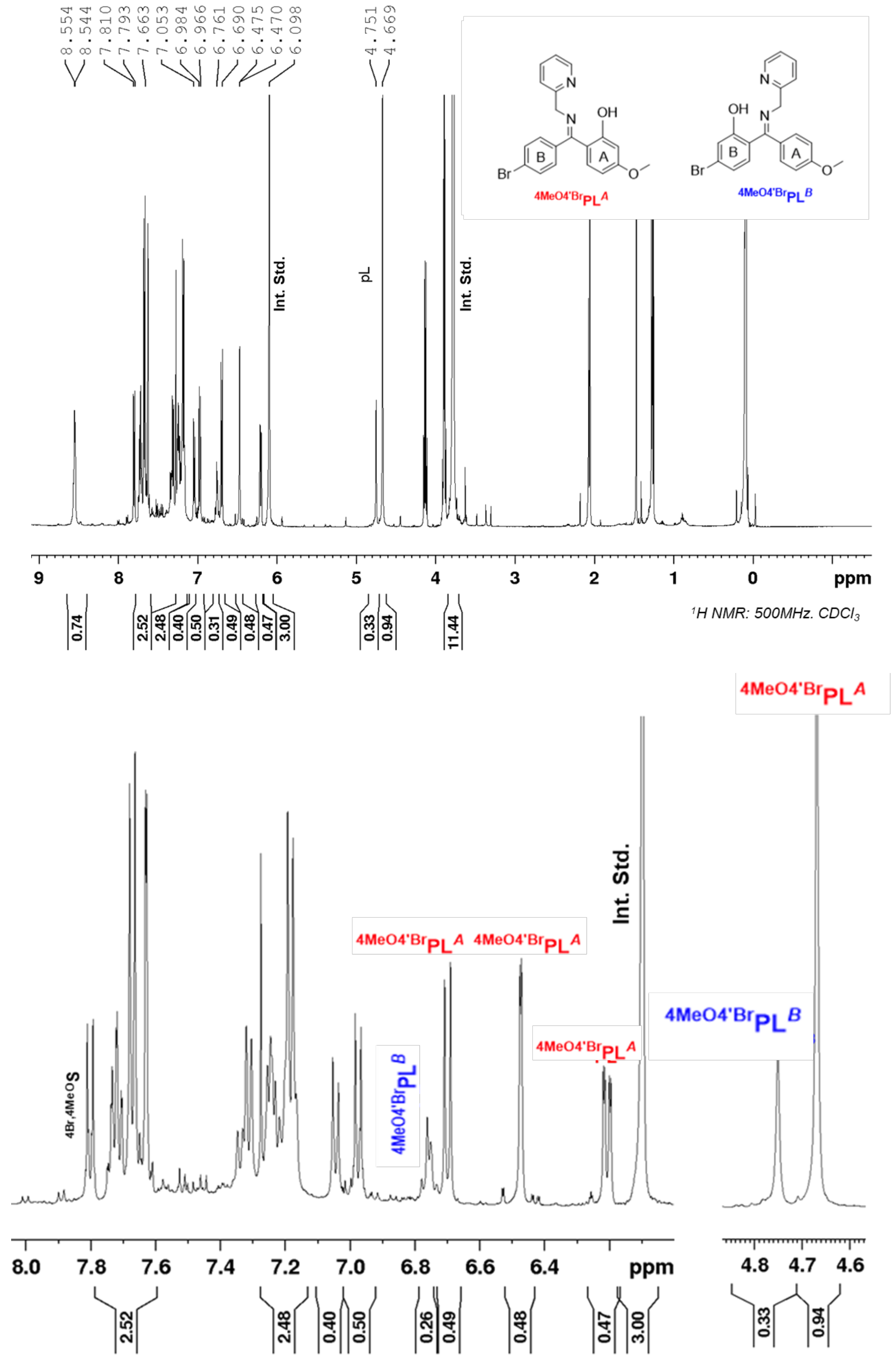

Figure S51. ^{1}H NMR spectra for the hydroxylation of $^{4MeO4'Br}$L. Note: The ratio of $^{4MeO4'Br}$PL^{A} and $^{4MeO4'Br}$PL^{B} (74/26) is calculated using the average of the integration of CH_2 peaks and CH peaks.

3.16 $^{4MeO4'CF3}$S and 2-picolyamine

Synthesis of $^{4MeO4'CF3}$L

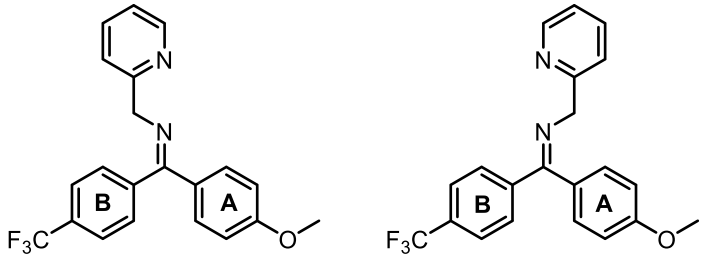

$^{4MeO4'CF3}$L *A* $^{4MeO4'CF3}$L *B*

In an oven dried flask, 2-picolylamine (2.2 equiv., 0.17 mL) was added to 4-(Trifluoromethyl)-4'-methoxybenzophenone (200 mg, 0.71 mmol) and p- toluenesulfonic acid monohydrate (cat. 10 mg, 2 mol%) in toluene (25 mL). The reaction mixture was refluxed under argon with a Dean-Stark apparatus until imine formation was complete (3 days). The reaction was cooled to room temperature and diluted with diethyl ether (30 mL). The organic layer was washed with saturated ammonia chloride (20 mL x 2), saturated aqueous sodium bicarbonate (20 mL), brine (20 mL), and dried with magnesium sulfate. The final product was isolated as a brown solid (80% yield, 210 mg, 90% pure). ^{1}H-NMR (500 MHz, $CDCl_3$): δ 8.53 (d, L^B, 2H), 8.52 (d, L^A, 2H), 7.84 (d, 2H), 7.77(d, L^A, 2H), 7.65-7.50 (m , L^A+L^B , 8H), 7.38(d, L^A, 4H), 7.18(d, L^A+L^B ,4H), 7.03 (d, L^B ,2H), 6.91(d, L^A, 2H), 4.84(s, L^B ,2H), 4.67(s, L^A ,2H), 3.89(s, L^B, 3H), 3.85(s, L^A,3H). HRMS (ESI) m/z: [M + Na]+ Calcd for C21H17F3N2ONa 370.1293, found 371.6245.

Hydroxylation of $^{4MeO4'CF3}$L

The reaction was carried out on 0.159 mmol scale using 65.3 mg of the imine according to the Standard Procedure. The reaction products were quantified using 0.159 mmol of 1,3,5-trimethoxybenzene (int. std.). (49% yield). The identity of the hydroxylation products was confirmed by ^{1}H-NMR.

Cleavage of $^{4MeO4'CF3}$PL

Dissolving $^{4MeO4'CF3}$PL in round bottom flask with 50 mL EtOAc, then adding 100 mL 1M HCl. Reaction was going for 30 min. The resulting mixture was extracted with EtOAc (50 mL X 2). The organic phases were separated, combined, dried over $MgSO_4$, filtered, and dried under vacuum. The reaction products were dissolved in 1.4 mL of $CDCl_3$ solution containing 27.1 mg of 1,3,5-trimethoxybenzene (internal standard). The reaction products were quantified by ^{1}H-NMR using integration signals that correspond to the starting material and products with the integration signal of the internal standard.

^{1}H-NMR spectra of $^{4MeO4'CF3}$L

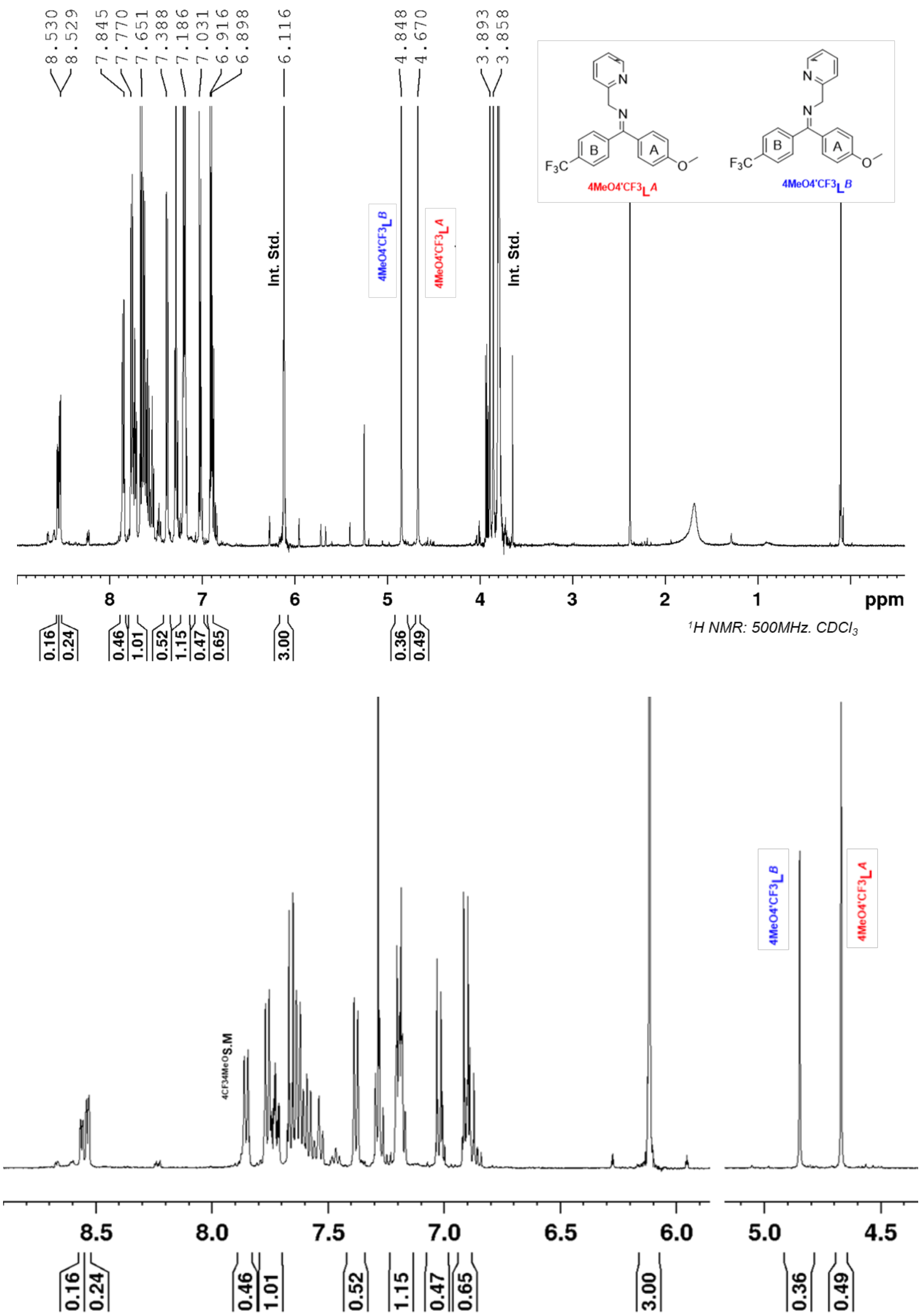

Figure S52. ^{1}H-NMR spectra of $^{4MeO4'CF3}$L. Note: There are two imine isomers. The ratio of $^{4MeO4'CF3}$L^A and $^{4MeO4'CF3}$L^B (57/43) is calculated using the average of the integration of CH_2 peaks and CH peaks.

^{1}H-NMR spectra for the hydroxylation of $^{4MeO4'CF3}$L

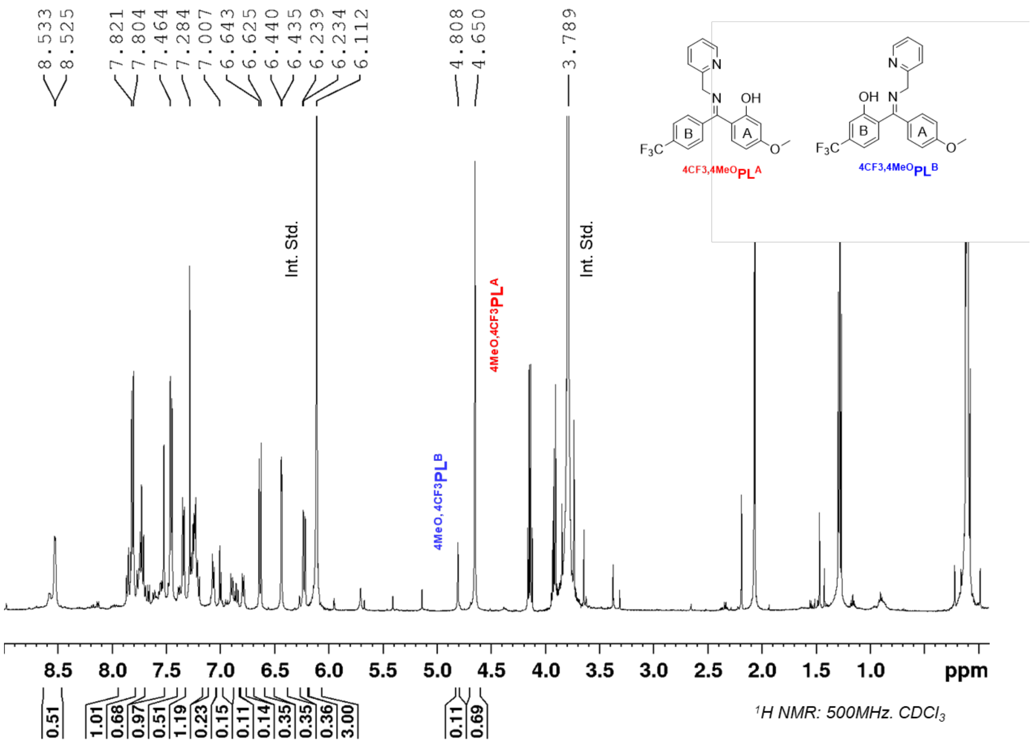

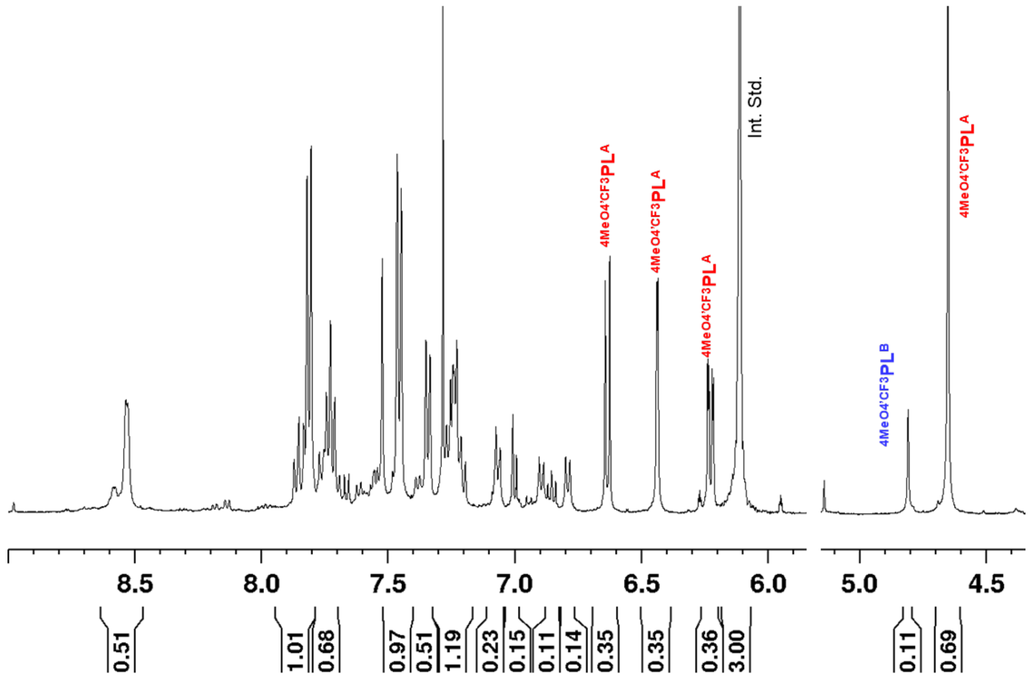

Figure S53. ^{1}H-NMR spectra for hydroxylation of $^{4MeO4'CF3}$L. Note: The ratio of $^{4MeO4'CF3}$PL^{A} and $^{4MeO4'CF3}$PL^{B} (86/14) is calculated using the average of the integration of CH_2 peaks and CH peaks.

^{1}H-NMR spectra for the cleavage of $^{4MeO4'CF3}$PL

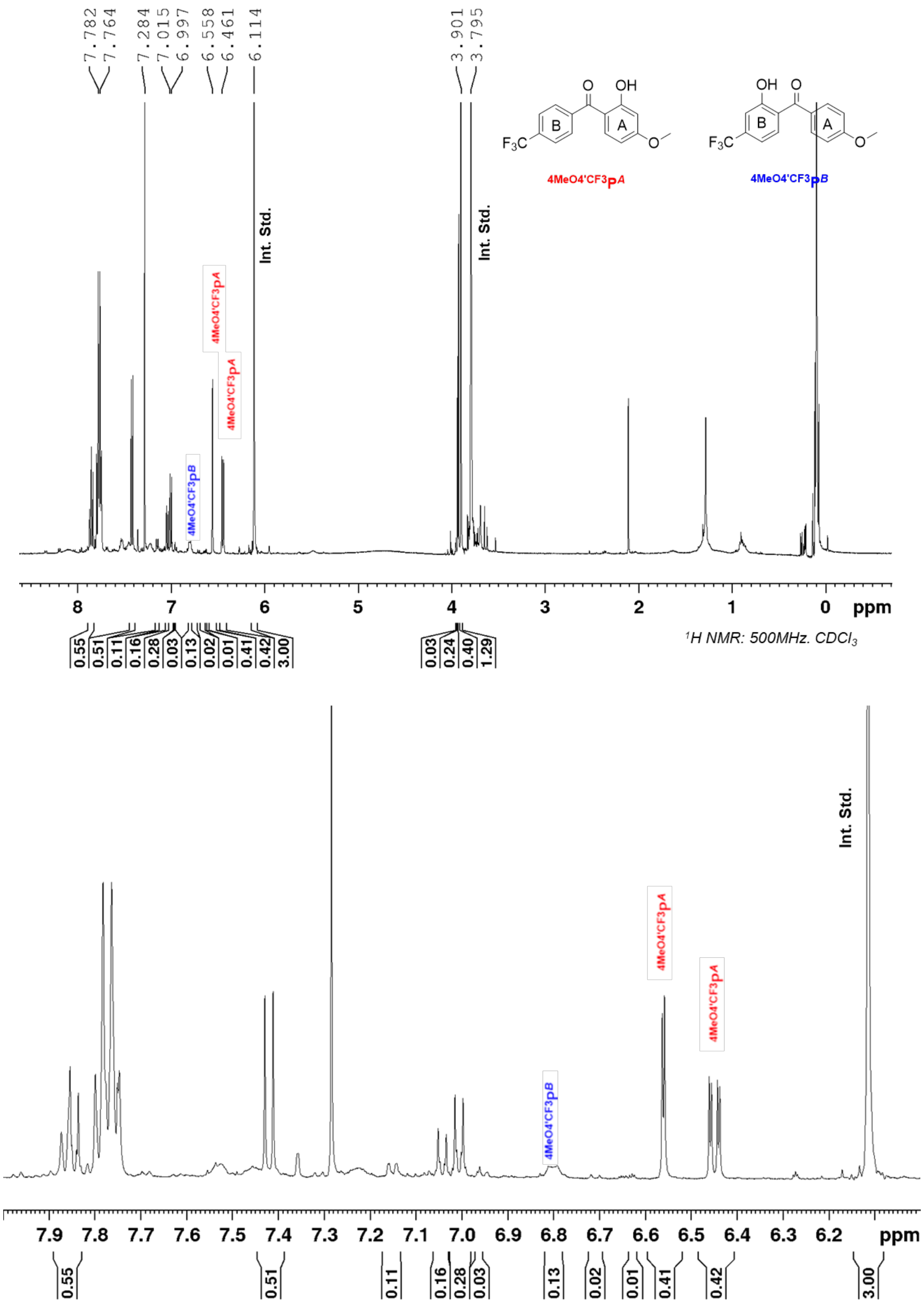

Figure S54. ^{1}H-NMR spectra for the cleavage of $^{4MeO4'CF3}$PL. Note: The ratio of $^{4MeO4'CF3}$P^{A} and $^{4MeO4'CF3}$P^{B} (79/21) is calculated using the average of the integration of CH peaks.

3.17 $^{4Me4'F}$S and 2-picolyamine

Synthesis of $^{4Me4'F}$L

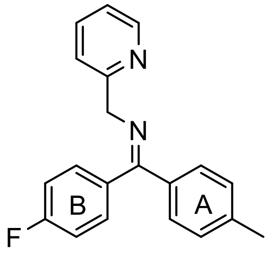

$^{4Me4'F}$L *A*

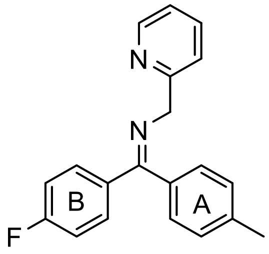

$^{4Me4'F}$L *B*

In an oven dried flask, 2-picolylamine (2.2 equiv, 2.2 mL.) was added to 4-Fluoro-4'-methybenzophenone (2.17 g, 9.85 mmol) and p- toluenesulfonic acid monohydrate (cat. 20 mg, 1.2 mol%) in toluene (50 mL). The reaction mixture was refluxed under argon with a Dean-Stark apparatus until imine formation was complete (2 days). The reaction was cooled to room temperature and diluted with diethyl ether (30 mL). The organic layer was washed with saturated ammonia chloride (20 mL x 2), saturated aqueous sodium bicarbonate (20 mL), brine (20 mL), and dried with magnesium sulfate. The final product was isolated as a brown solid (80% yield, 2.40 g, 86% pure). ^{1}H-NMR (500 MHz, $CDCl_3$): δ 8.54 (d, L^A+L^B, 2H), 7.70 (m, L^A+L^B, 4H), 7.60(t, L^A+L^B ,4H), 7.30- 7.20(d, L^A+L^B ,10H), 7.12(d, 2H), 7.02(t, L^B, 2H), 4.76(s, L^B ,2H), 4.73(s, L^A ,2H), 2.43(s, L^A ,3H), 2.41(s, L^B ,3H). HRMS (ESI) m/z: [M + Na]+ Calcd for C20H17FN2Na 304.1376, found 305.6431.

Hydroxylation of $^{4Me4'F}$L

The reaction was carried out on 0.159 mmol scale using 56.2 mg of the imine according to the Standard Procedure. The reaction products were quantified using 0.159 mmol of 1,3,5-trimethoxybenzene (int. std.). (60% yield). The identity of the hydroxylation products was confirmed by ^{1}H-NMR.

Cleavage of $^{4Me4'F}$PL

Dissolving $^{4Me4'F}$PL in round bottom flask with 50 mL EtOAc, then adding 100 mL 1M HCl. Reaction was going for 30 min. The resulting mixture was extracted with EtOAc (50 mL X 2). The organic phases were separated, combined, dried over $MgSO_4$, filtered, and dried under vacuum. The reaction products were dissolved in 1.4 mL of $CDCl_3$ solution containing 27.1 mg of 1,3,5-trimethoxybenzene (internal standard). The reaction products were quantified by ^{1}H-NMR using integration signals that correspond to the starting material and products with the integration signal of the internal standard.

^{1}H NMR spectra of [4Me4’F]L

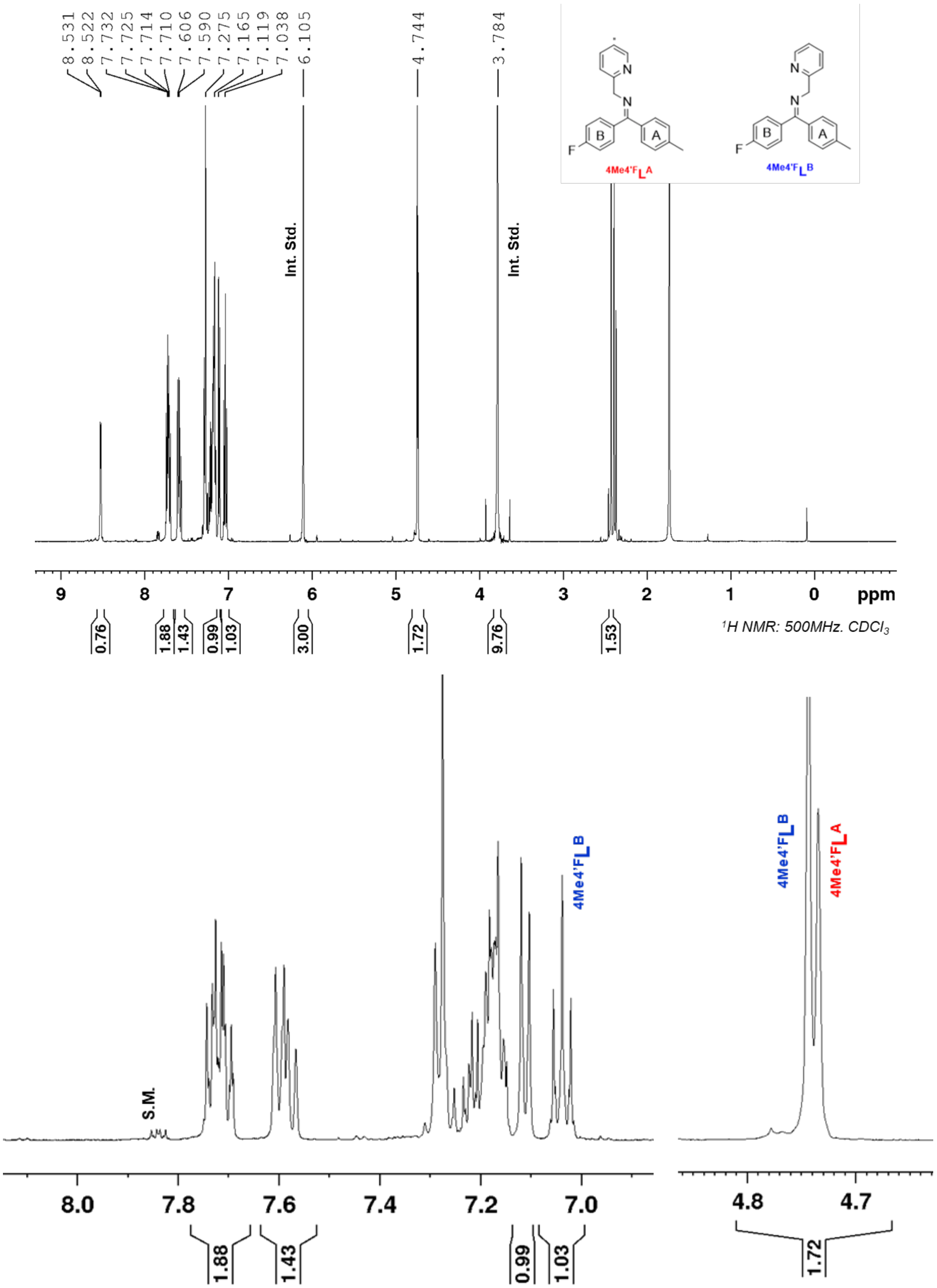

Figure S55. ^{1}H-NMR spectra of $^{4Me4'F}L$. Note: There are two imine isomers. The ratio of $^{4Me4'F}L^A$ and $^{4Me4'F}L^B$ (42/58) is calculated using the average of the integration of CH_2 peaks and CH peaks.

^{1}H-NMR spectra for the hydroxylation of $^{4Me4'F}L$

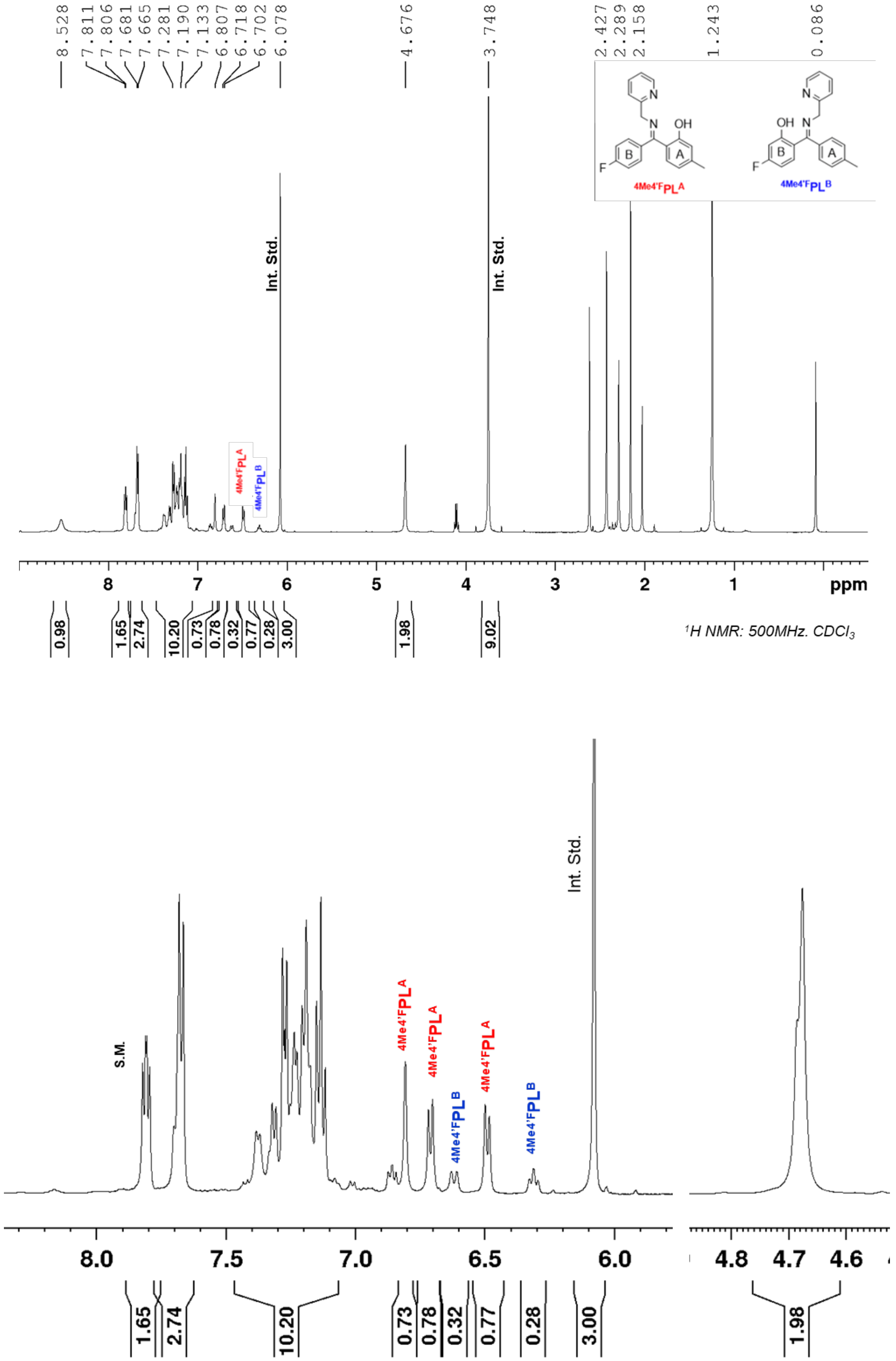

Figure S56. ^{1}H-NMR spectra for the hydroxylation of $^{4Me4'F}L$. Note: The ratio of $^{4Me4'F}PL^A$ and $^{4Me4'F}PL^B$ (74/26) is calculated using the average of the integration of CH_2 peaks and CH peaks.

^{1}H-NMR spectra for the cleavage of $^{4Me4'F}$PL

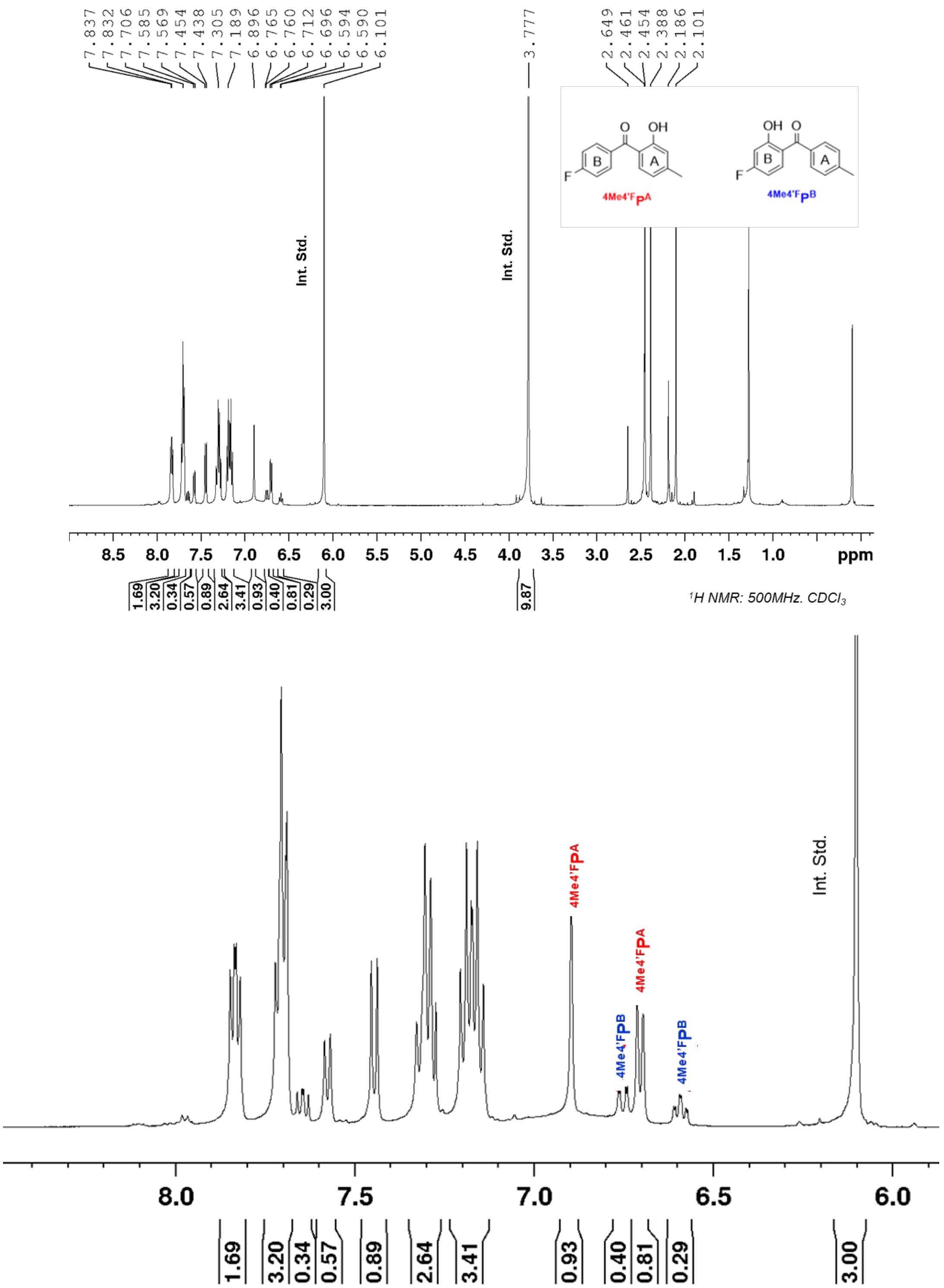

Figure S57. ^{1}H-NMR spectra for the cleavage of $^{4Me4'F}$PL. Note: The ratio of $^{4Me4'F}P^{A}$ and $^{4Me4'F}P^{B}$ (74/26) is calculated using the average of the integration of CH peaks.X

3.18 ^{4MeO}S and 2-(aminomethyl)-4-methoxypyridine

Synthesis of $^{4MeO}L_{4MeO\text{-}py}$

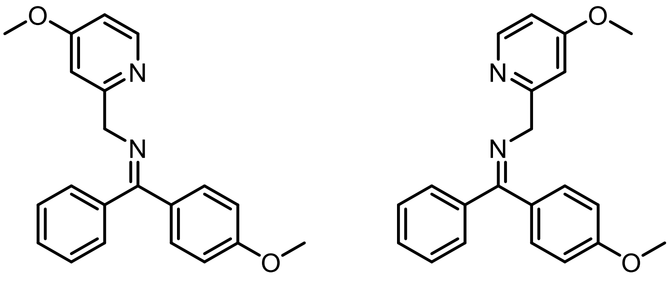

In an oven dried flask, 2-(aminomethyl)-4-methoxypyridine (2.2 equiv. 0.31 mL) was added to 4-Methoxybenzophenone (600 mg, 1.38 mmol) and p-toluenesulfonic acid monohydrate (cat. 10 mg, 1.5 mol%) in toluene (30 mL). The reaction mixture was refluxed under argon with a Dean-Stark apparatus until imine formation was complete (2 days). The reaction was cooled to room temperature and diluted with diethyl ether (30 mL). The organic layer was washed with saturated ammonia chloride (20 mL x 2), saturated aqueous sodium bicarbonate (20 mL), brine (20 mL), and dried with magnesium sulfate. The final product was isolated as a brown solid (80% yield, 366 mg, 96% pure). ^{1}H-NMR (500 MHz, $CDCl_3$): δ 8.34 (d, 2H), 7.70 - 7.66 (m, L^E +L^Z , 4H), 7.48-7.38 (m, L^E +L^Z ,6H) 7.27 – 7.16 (m, L^E +L^Z , 10H), 6.99 (d, L^E +L^Z ,2H), 6.89 (d, L^E +L^Z ,2H), 6.69 (d, 2H), 4.77 (s, L^Z, 2H), 4.67 (s, L^E , 2H), 3.91(s, 6H), 3.86 (s , L^E +L^Z , 6H). HRMS (ESI) m/z: [M + Na]+ Calcd for C21H20N2O2Na 332.1525, found 333.1558.

Hydroxylation of $^{4MeO}L_{4MeO\text{-}py}$

The reaction was carried out on 0.079 mmol scale using 27.3 mg of the imine according to the Standard Procedure. The reaction products were quantified using 0.079 mmol of 1,3,5-trimethoxybenzene (int. std.). (51% yield). The identity of the hydroxylation products was confirmed by ^{1}H-NMR.

Cleavage of $^{4MeO}PL_{4MeO\text{-}py}$

Dissolving $^{4MeO}PL_{4MeO\text{-}py}$ in round bottom flask with 50 mL EtOAc, then adding 100 mL 1M HCl. Reaction was going for 30 min. The resulting mixture was extracted with EtOAc (50 mL X 2). The organic phases were separated, combined, dried over $MgSO_4$, filtered, and dried under vacuum. The reaction products were dissolved in 1.4 mL of $CDCl_3$ solution containing 13.5 mg of 1,3,5-trimethoxybenzene (internal standard). The reaction products were quantified by ^{1}H-NMR using integration signals that correspond to the starting material and products with the integration signal of the internal standard.

^{1}H-NMR spectra of $^{4MeO}L_{4MeO\text{-}py}$

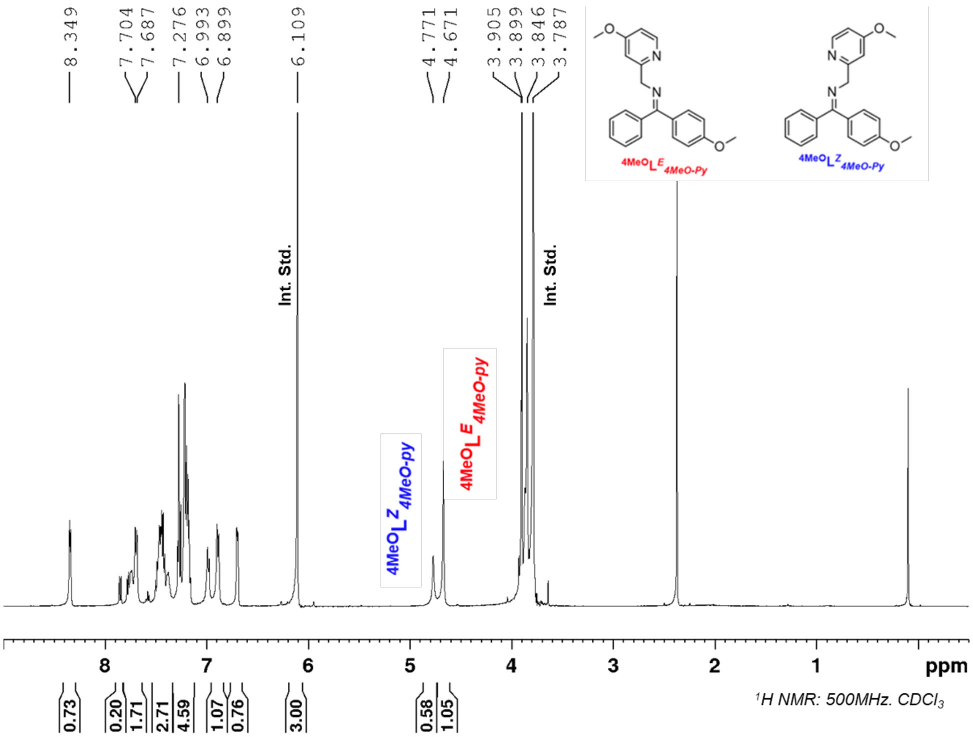

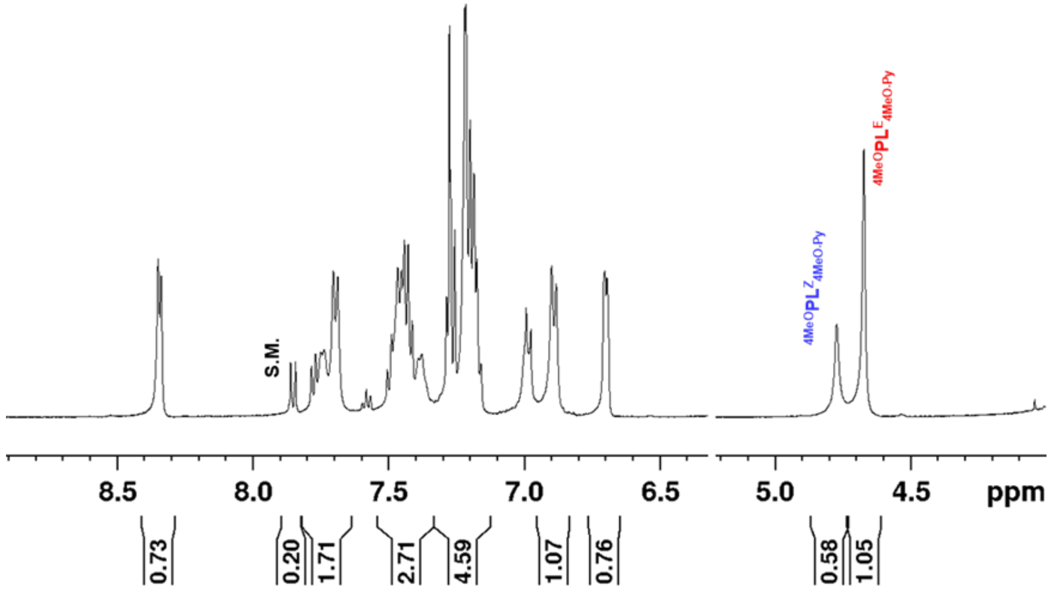

Figure S58. ^{1}H-NMR spectra of $^{4MeO}L_{4MeO\text{-}py}$. Note: There are two imine isomers. The ratio of $^{4MeO}L^{E}_{4MeO\text{-}py}$ and $^{4MeO}L^{Z}_{4MeO\text{-}py}$ (65/35) is calculated using the average of the integration of CH_2 peaks and CH peaks.

^{1}H-NMR spectra for the hydroxylation of $^{4MeO}L_{4MeO\text{-}py}$

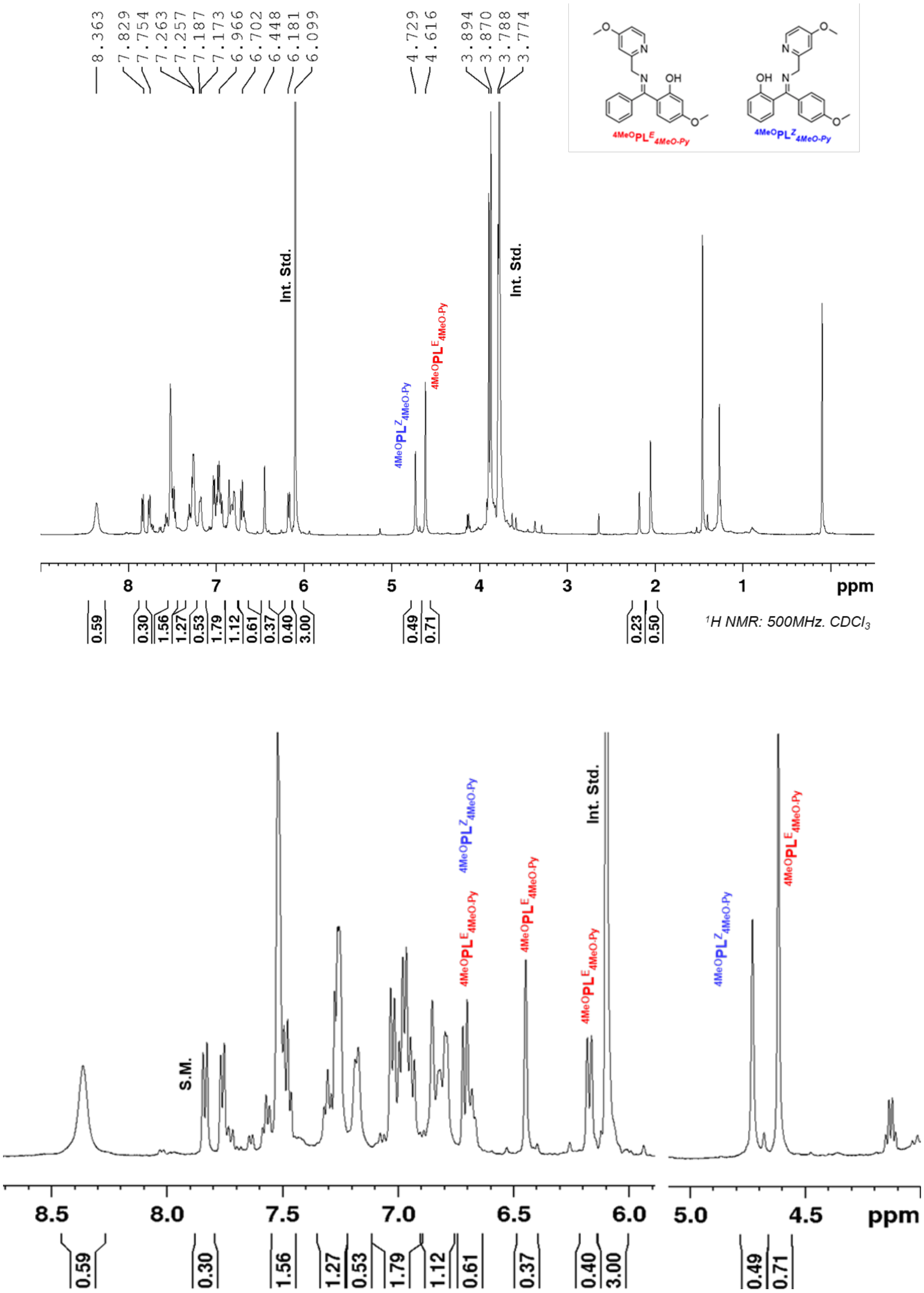

Figure S59. ^{1}H-NMR spectra of hydroxylation products of $^{4MeO}L_{4MeO\text{-}py}$. Note: The ratio of $^{4MeO}PL^{E}_{4MeO\text{-}py}$ and $^{4MeO}PL^{Z}_{4MeO\text{-}py}$ (65/35) is calculated using the average of the integration of CH_2 peaks and CH peaks.

^{1}H-NMR spectra for the cleavage of [4MeO]PL[4MeO-py]

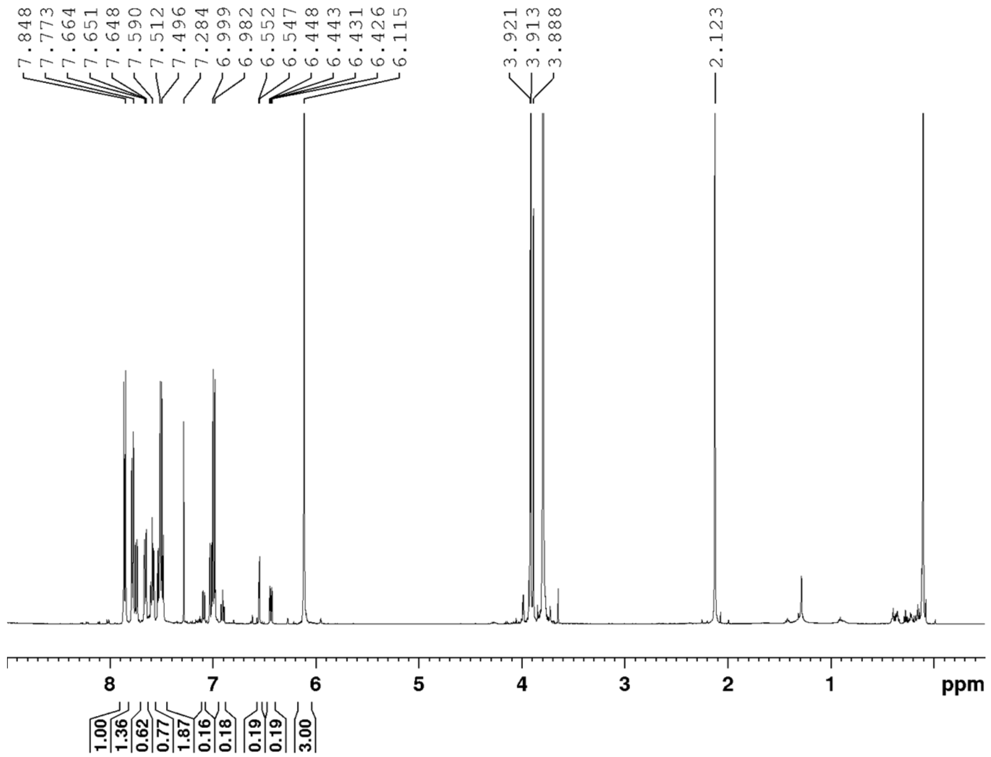

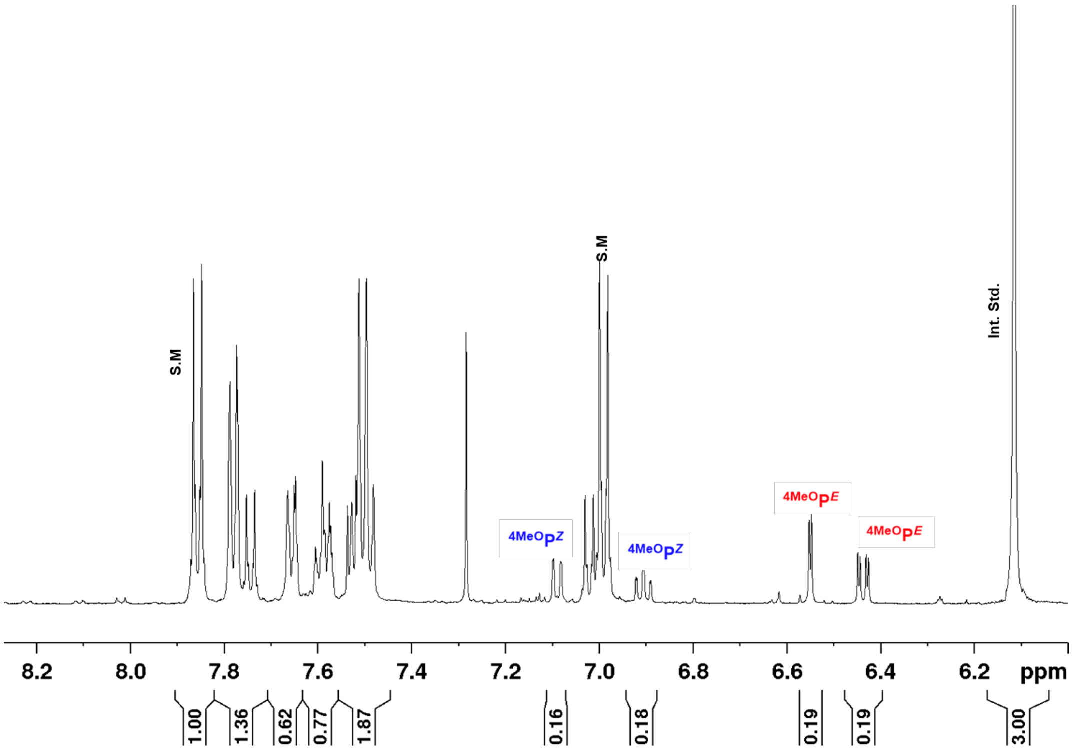

Figure S60. ^{1}H-NMR spectra for the cleavage of $^{4MeO}PL_{4MeO\text{-}py}$**.** Note: The ratio of $^{4MeO}P^{E}$ and $^{4MeO}P^{Z}$ (52/48) is calculated using the average of the integration of CH peaks.

3.19 ^{4Cl}S and 2-(aminomethyl)-4-methoxypyridine

Synthesis of $^{4Cl}L_{4MeO\text{-}py}$

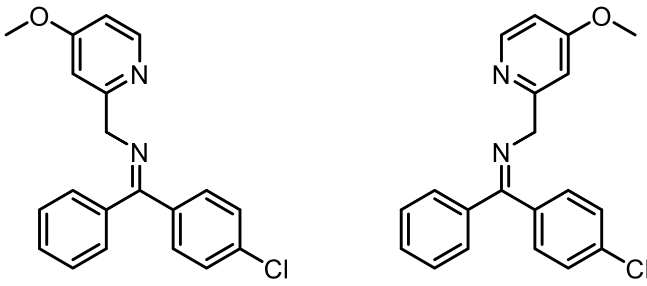

$^{4Cl}L^{E}_{4MeO\text{-}py}$ $^{4Cl}L^{Z}_{4MeO\text{-}py}$

In an oven dried flask, 2-(aminomethyl)-4-methoxypyridine (2.2 equiv., 0.31 mL) was added to 4-chlorobenzophenone (600 mg, 1.38 mmol) and p-toluenesulfonic acid monohydrate (cat. 10 mg, 4 mol%) in toluene (30 mL). The reaction mixture was refluxed under argon with a Dean-Stark apparatus until imine formation was complete (2 days). The reaction was cooled to room temperature and diluted with diethyl ether (30 mL). The organic layer was washed with saturated ammonia chloride (20 mL x 2), saturated aqueous sodium bicarbonate (20 mL), brine (20 mL), and dried with magnesium sulfate. The final product was isolated as a brown solid (86% yield, 399 mg, 90% pure). ^{1}H-NMR (500 MHz, $CDCl_3$): δ 8.35 (d, L^E + L^Z ,2H), 7.70 (d , 2H), 7.66 (d, L^E +L^Z , 4H), 7.46 (m, L^E +L^Z , 6H), 7.34 (d , L^E +L^Z , 4H), 7.20 (m, L^E +L^Z ,4H), 6.71 (d ,2H), 4.69 (s, L^Z , 2H), 4.68 (s, L^E , 2H). HRMS (ESI) m/z: [M + Na]+ Calcd for C20H17ClN2ONa 336.1029, found 337.1143.

Hydroxylation of $^{4Cl}L_{4MeO\text{-}py}$

The reaction was carried out on 0.079 mmol scale using 29.5 mg of the imine according to the Standard Procedure. The reaction products were quantified using 0.079 mmol of 1,3,5-trimethoxybenzene (int. std.). (52% yield). The identity of the hydroxylation products was confirmed by ^{1}H-NMR.

Cleavage of $^{4Cl}PL_{4MeO\text{-}py}$

Dissolving $^{4Cl}PL_{4MeO\text{-}py}$ in round bottom flask with 50 mL EtOAc, then adding 100 mL 1M HCl. Reaction was going for 30 min. The resulting mixture was extracted with EtOAc (50 mL X 2). The organic phases were separated, combined, dried over $MgSO_4$, filtered, and dried under vacuum. The reaction products were dissolved in 1.4 mL of $CDCl_3$ solution containing 13.5 mg of 1,3,5-trimethoxybenzene (internal standard). The reaction products were quantified by ^{1}H-NMR using integration signals that correspond to the starting material and products with the integration signal of the internal standard.

^{1}H-NMR spectra of $^{4Cl}L_{4MeO\text{-}py}$

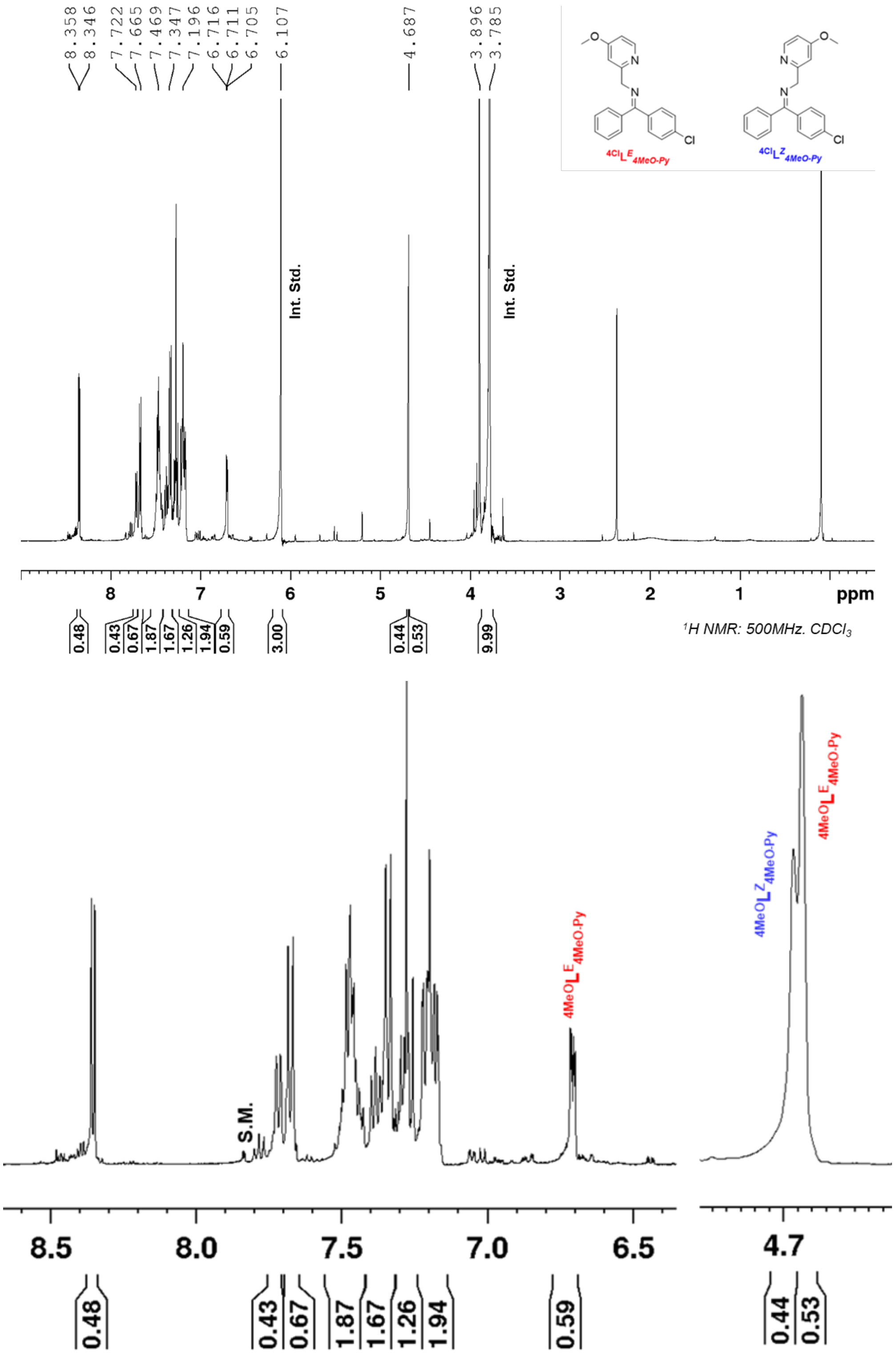

Figure S61. ^{1}H-NMR spectra of $^{4Cl}L_{4MeO\text{-}py}$. Note: There are two imine isomers. The ratio of $^{4Cl}L^{E}_{4MeO\text{-}py}$ and $^{4Cl}L^{Z}_{4MeO\text{-}py}$ (60/40) is calculated using the average of the integration of CH_2 peaks and CH peaks.

^{1}H-NMR spectra for the hydroxylation of $^{4Cl}L_{4MeO\text{-}py}$

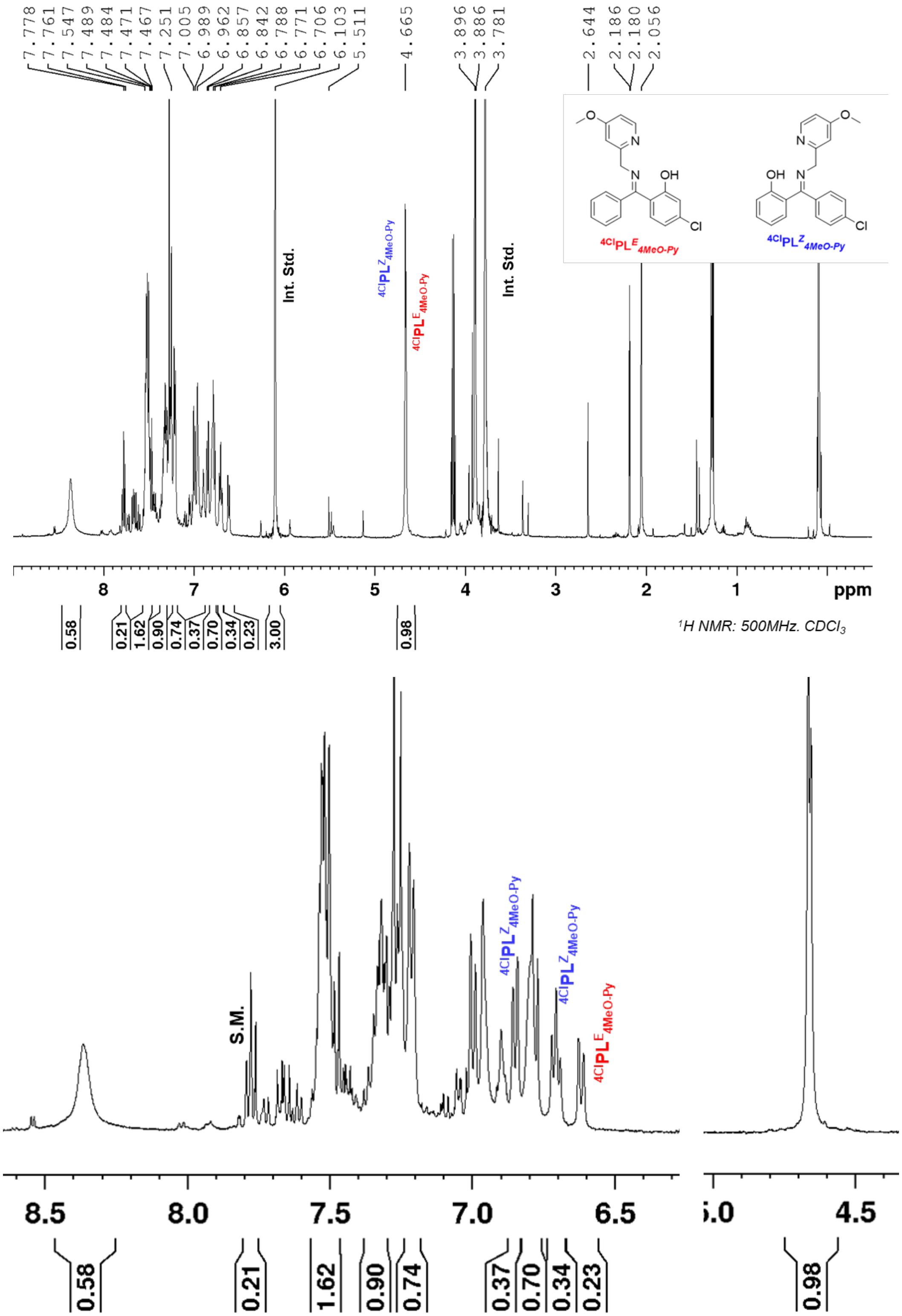

Figure S62. ^{1}H-NMR spectra for the hydroxylation of $^{4Cl}L_{4MeO\text{-}py}$. Note: The ratio of $^{4Cl}PL^{E}_{4MeO\text{-}py}$ and $^{4Cl}PL^{Z}_{4MeO\text{-}py}$ (43/57) is calculated using the average of the integration of CH_2 peaks and CH peaks.

^{1}H-NMR spectra for the cleavage of $^{4Cl}PL_{4MeO\text{-}py}$

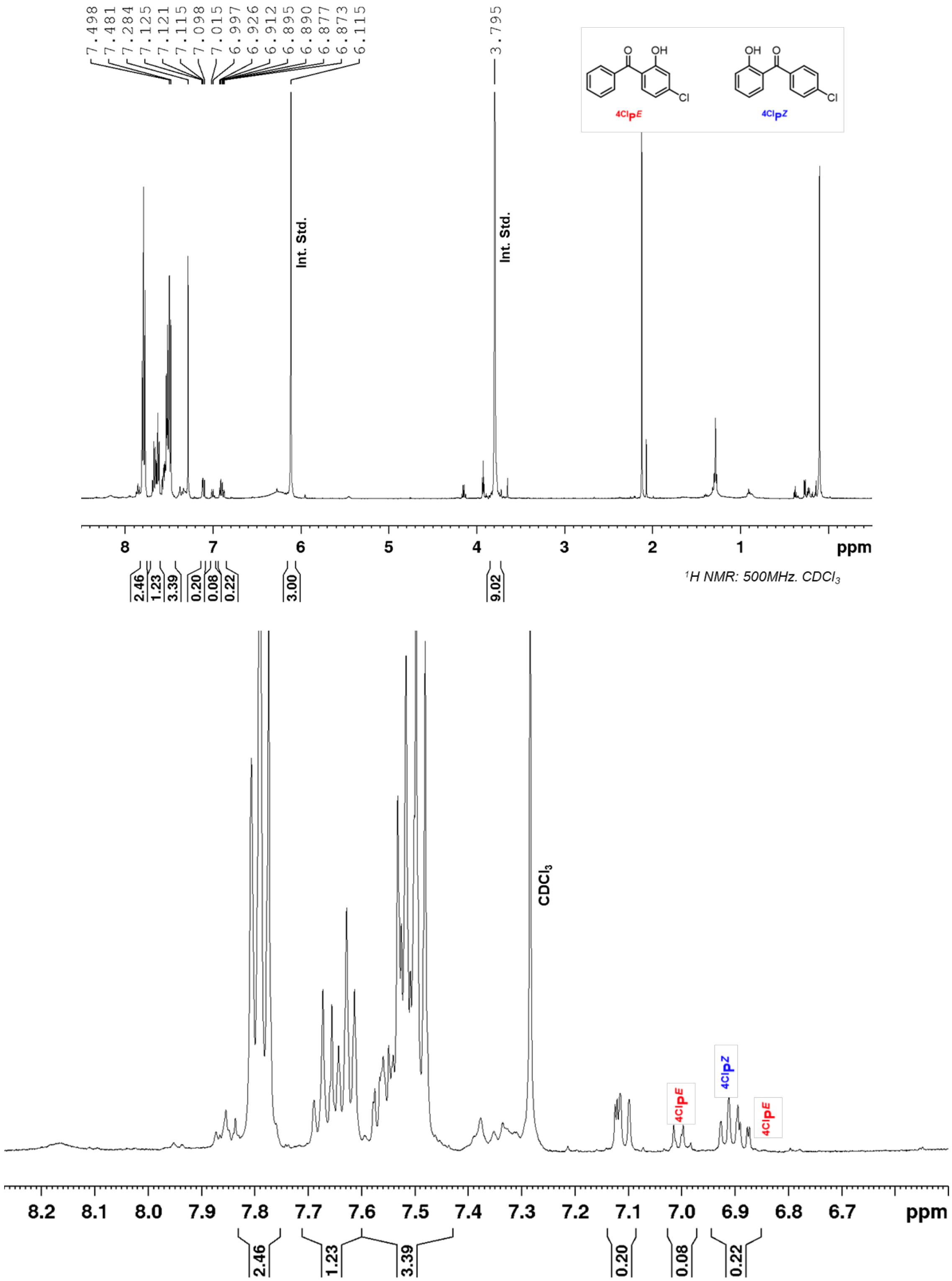

Figure S63. ^{1}H-NMR spectra for the cleavage of $^{4Cl}PL_{4MeO\text{-}py}$**.** Note: The ratio of $^{4Cl}P^{E}$ and $^{4Cl}P^{Z}$ (36/64) is calculated using the average of the integration of CH peaks.

3.20 $^{4MeO4'Cl}$S and 2-(aminomethyl)-4-methoxypyridine

Synthesis of $^{4MeO4'Cl}L_{4MeO\text{-}py}$

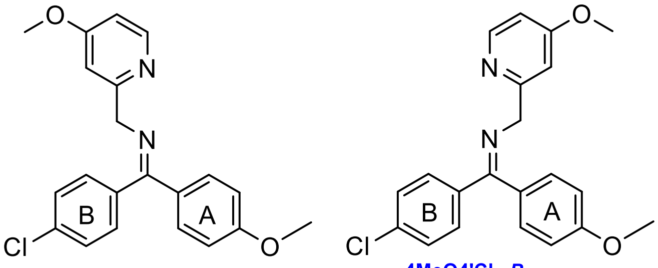

$^{4MeO4'Cl}L^{A}_{4MeO\text{-}py}$ $^{4MeO4'Cl}L^{B}_{4MeO\text{-}py}$

In an oven dried flask, (4-Methoxypyridin-2-yl)methanamine (2.2 equiv., 0.36 mL) was added to 4-Chloro, 4-methoxybenzophenone (400 mg, 1.62 mmol) and p-toluenesulfonic acid monohydrate (cat. 10 mg, 3.5 mol%) in toluene (30 mL). The reaction mixture was refluxed under argon with a Dean-Stark apparatus until imine formation was complete (3 days). The reaction was cooled to room temperature and diluted with diethyl ether (30 mL). The organic layer was washed with saturated ammonia chloride (20 mL x 2), saturated aqueous sodium bicarbonate (20 mL), brine (20 mL), and dried with magnesium sulfate. The final product was isolated as a brown solid (90% yield, 540 mg, 90% pure). ^{1}H-NMR (500 MHz, $CDCl_3$): δ 8.34 (d, L^B +L^A 2H), 7.65 (q, L^B +L^A , 4H), 7.46 (d, L^B +L^A , 2H), 7.34 (d, L^B +L^A , 2H), 7.27 (d, L^B +L^A, 2H), 7.15(m , L^B +L^A, 4H), 7.0 (d,L^B +L^A , 2H), 6.90 (d, ,2H), 6.70(dd, L^B +L^A, 2H), 4.73 (s, L^B , 2H), 4.64 (s, L^A , 2H), 3.89 (s , L^B +L^A ,6H), 3.87 (s, L^B , 3H). 3.84 (s, L^A, 3H). HRMS (ESI) m/z: [M + Na]+ Calcd for C20H16Cl2N2ONa 371.2610, found 373.1375.

Hydroxylation of $^{4MeO4'Cl}L_{4MeO\text{-}py}$

The reaction was carried out on 0.079 mmol scale using 32.6 mg of the imine according to the Standard Procedure. The reaction products were quantified using 0.079 mmol of 1,3,5-trimethoxybenzene (int. std.). (55% yield). The identity of the hydroxylation products was confirmed by ^{1}H-NMR.

Cleavage of $^{4MeO4'Cl}PL_{4MeO\text{-}py}$

Dissolving $^{4MeO,\ 4Cl}PL_{4MeO\text{-}Py}$ in round bottom flask with 50 mL EtOAc, then adding 100 mL 1M HCl. Reaction was going for 30 min. The resulting mixture was extracted with EtOAc (50 mL X 2). The organic phases were separated, combined, dried over $MgSO_4$, filtered, and dried under vacuum. The reaction products were dissolved in 1.4 mL of $CDCl_3$ solution containing 13.5 mg of 1,3,5-trimethoxybenzene (internal standard). The reaction products were quantified by ^{1}H-NMR using integration signals that correspond to the starting material and products with the integration signal of the internal standard.

^{1}H-NMR spectra of $^{4MeO4'Cl}L_{4MeO\text{-}py}$

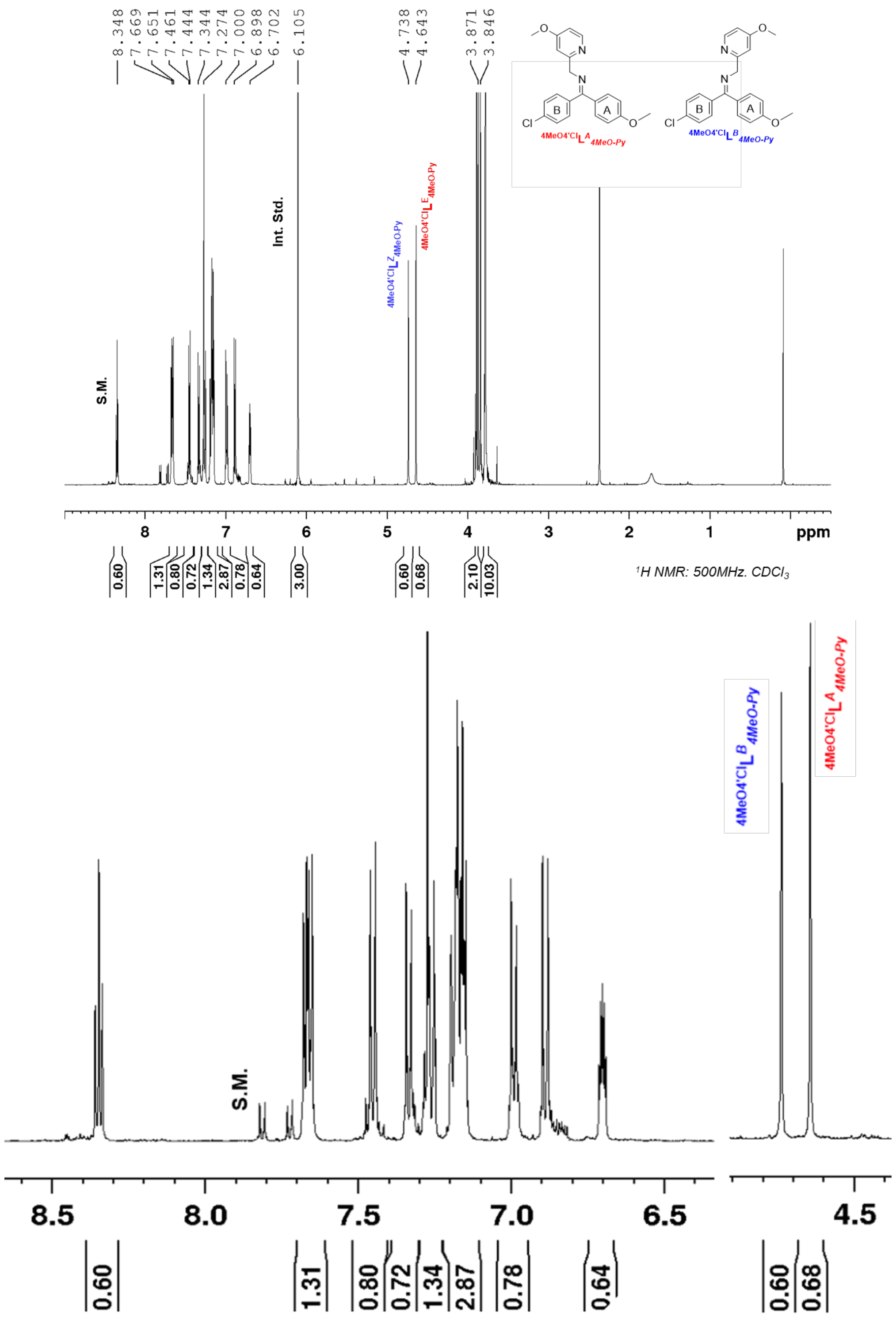

Figure S64. ^{1}H-NMR spectra of $^{4MeO4'Cl}L_{4MeO\text{-}py}$. Note: There are two imine isomers. The ratio of $^{4MeO4'Cl}L^{A}_{4MeO\text{-}py}$ and $^{4MeO4'Cl}L^{B}_{4MeO\text{-}py}$ (53/47) is calculated using the average of the integration of CH_2 peaks and CH peaks.

^{1}H-NMR spectra for the hydroxylation of $^{4MeO4'Cl}L_{4MeO\text{-}py}$

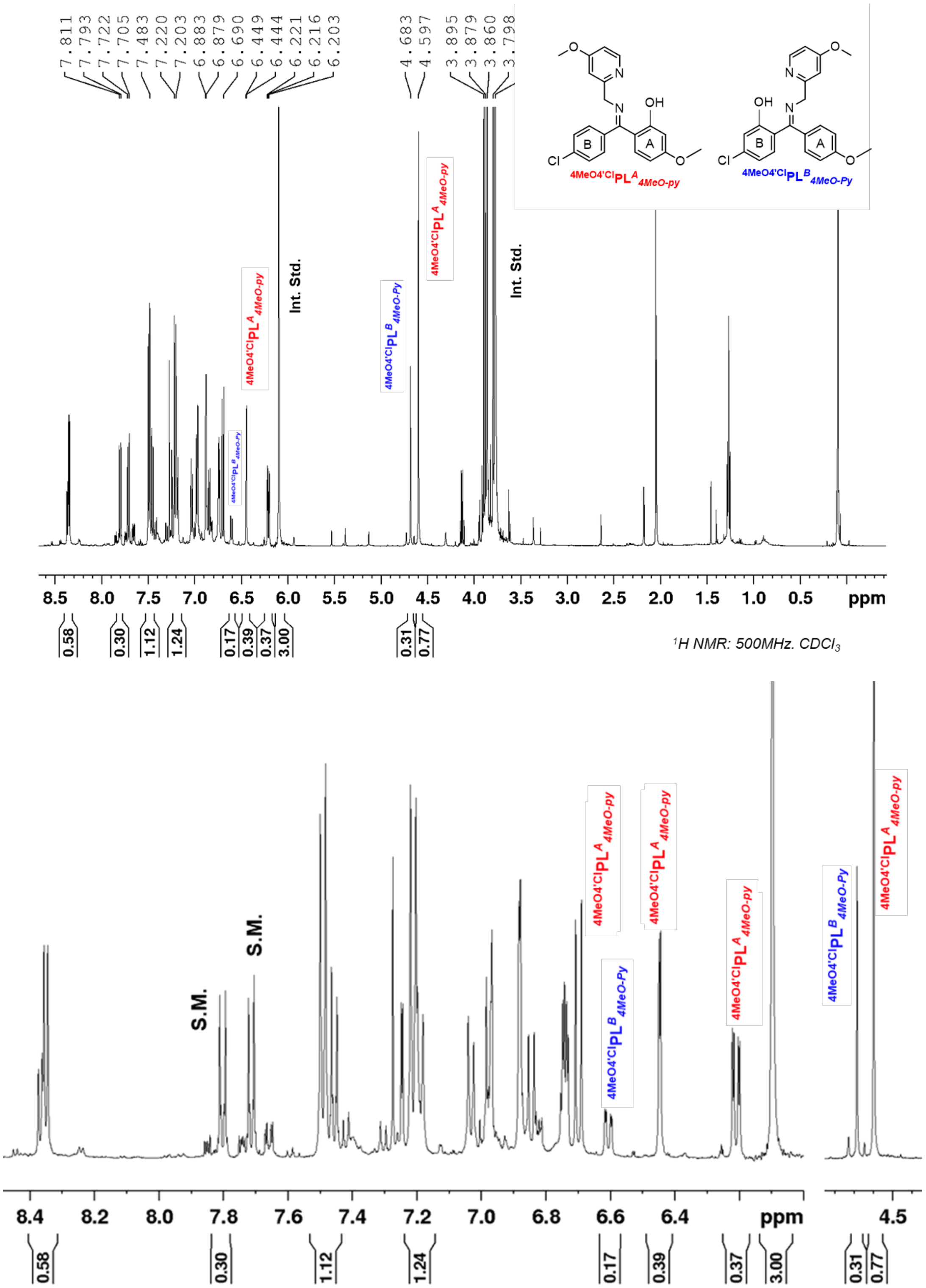

Figure S65. ^{1}H-NMR spectra for the hydroxylation of $^{4MeO4'Cl}L_{4MeO\text{-}py}$. Note: The ratio of $^{4MeO4'Cl}PL^{A}_{4MeO\text{-}py}$ and $^{4MeO4'Cl}PL^{B}_{4MeO\text{-}py}$ (74/26) is calculated using the average of the integration of CH_2 peaks and CH peaks.

^{1}H-NMR spectra for the cleavage of $^{4MeO4'Cl}PL_{4MeO\text{-}py}$

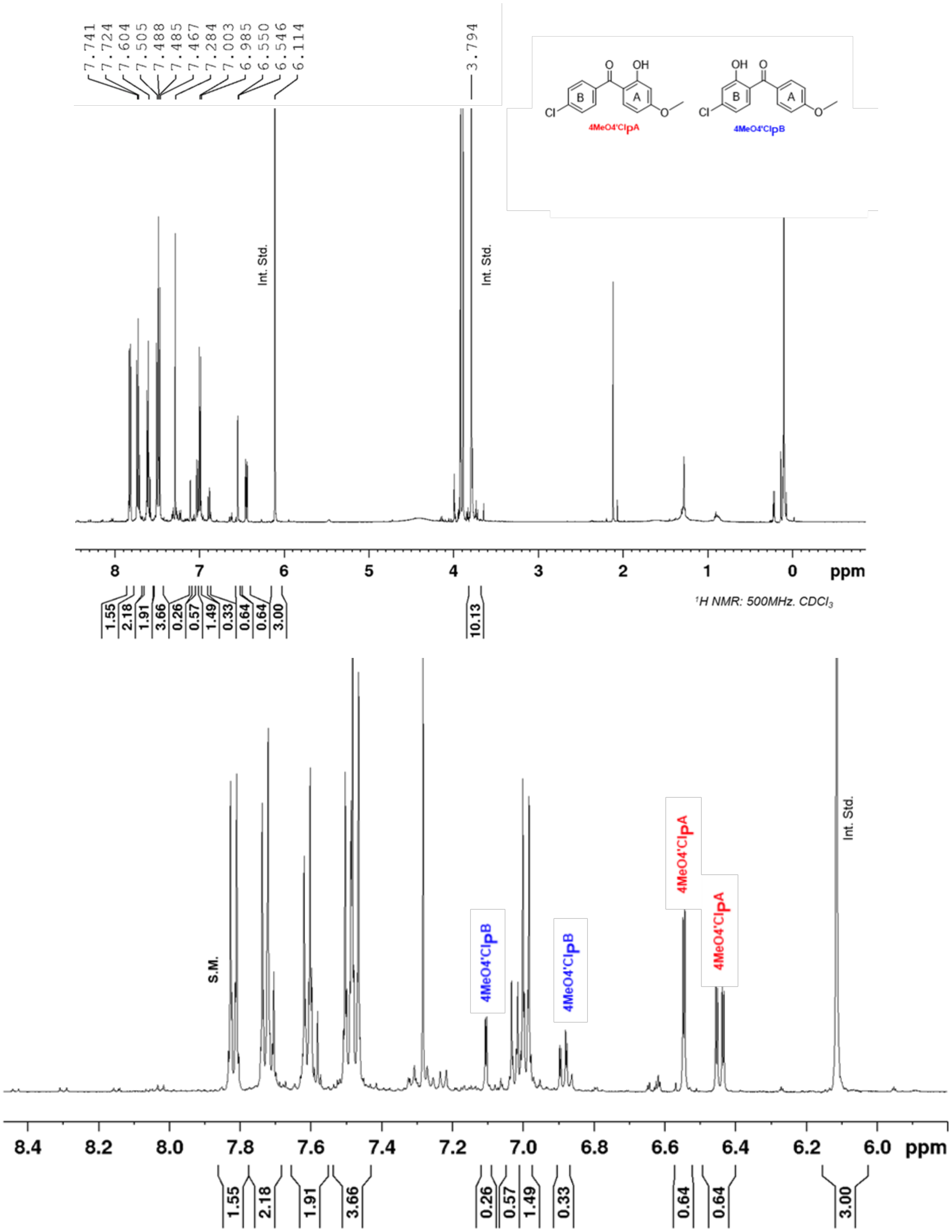

Figure S66. ^{1}H NMR spectra for the cleavage of $^{4MeO4'Cl}PL_{4MeO\text{-}py}$**.** Note: The ratio of $^{4MeO4'Cl}P^{A}$ and $^{4MeO4'Cl}P^{B}$ (71/29) is calculated using the average of the integration of CH peaks.

3.21 $^{4Me4'F}$S and 2-(aminomethyl)-4-methoxypyridine

Synthesis of $^{4Me4'F}L_{4MeO\text{-}py}$

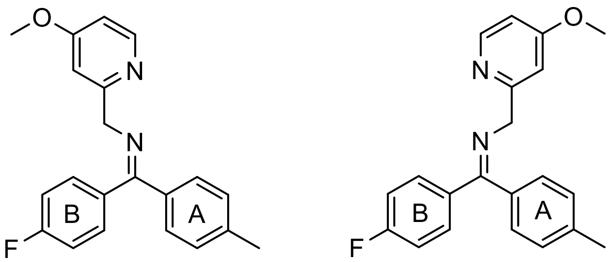

$^{4Me4'F}L^{A}_{4MeO\text{-}py}$ $^{4Me4'F}L^{B}_{4MeO\text{-}py}$

In an oven dried flask, 2-(aminomethyl)-4-methoxypyridine (2.2 equiv., 2.2 mL) was added to 4-methyl,4'fluoro-benzophenone (2.15 g, 9.85 mmol) and p- toluenesulfonic acid monohydrate (cat. 20 mg, 1.2%) in toluene (50 mL). The reaction mixture was refluxed under argon with a Dean-Stark apparatus until imine formation was complete (3 days). The reaction was cooled to room temperature and diluted with diethyl ether (30 mL). The organic layer was washed with saturated ammonia chloride (20 mL x 2), saturated aqueous sodium bicarbonate (20 mL), brine (20 mL), and dried with magnesium sulfate. The final product was isolated as a brown solid (80% yield, 2.60 g, 95% pure). ^{1}H-NMR (500 MHz, $CDCl_3$): δ 8.35 (d, 2H), 7.73 (td, 2H), 7.59 (d, 2H), 7.27 (m, L^B +L^A , 4H) 7.19 (m, L^B +L^A , 6H), 7.09 (d, 2H), 7.04 (t, L^A ,2H), 6.86 (d, 2H), 4.69 (d, L^B +L^A, 4H), 3.89 (s, L^B +L^A ,6H). 2.39 (s, L^B, 3H), 2.37 (s, L^A, 3H). HRMS (ESI) m/z: [M + Na]+ Calcd for C21H19FN2ONa 334.3944, found 335.1521.

Hydroxylation of $^{4Me4'F}L_{4MeO\text{-}py}$

The reaction was carried out on 0.079 mmol scale using 27.8 mg of the imine according to the Standard Procedure. The reaction products were quantified using 0.079 mmol of 1,3,5-trimethoxybenzene (int. std.). (50% yield). The identity of the hydroxylation products was confirmed by ^{1}H-NMR.

^{1}H-NMR spectra of $^{4Me4'F}L_{4MeO\text{-}py}$

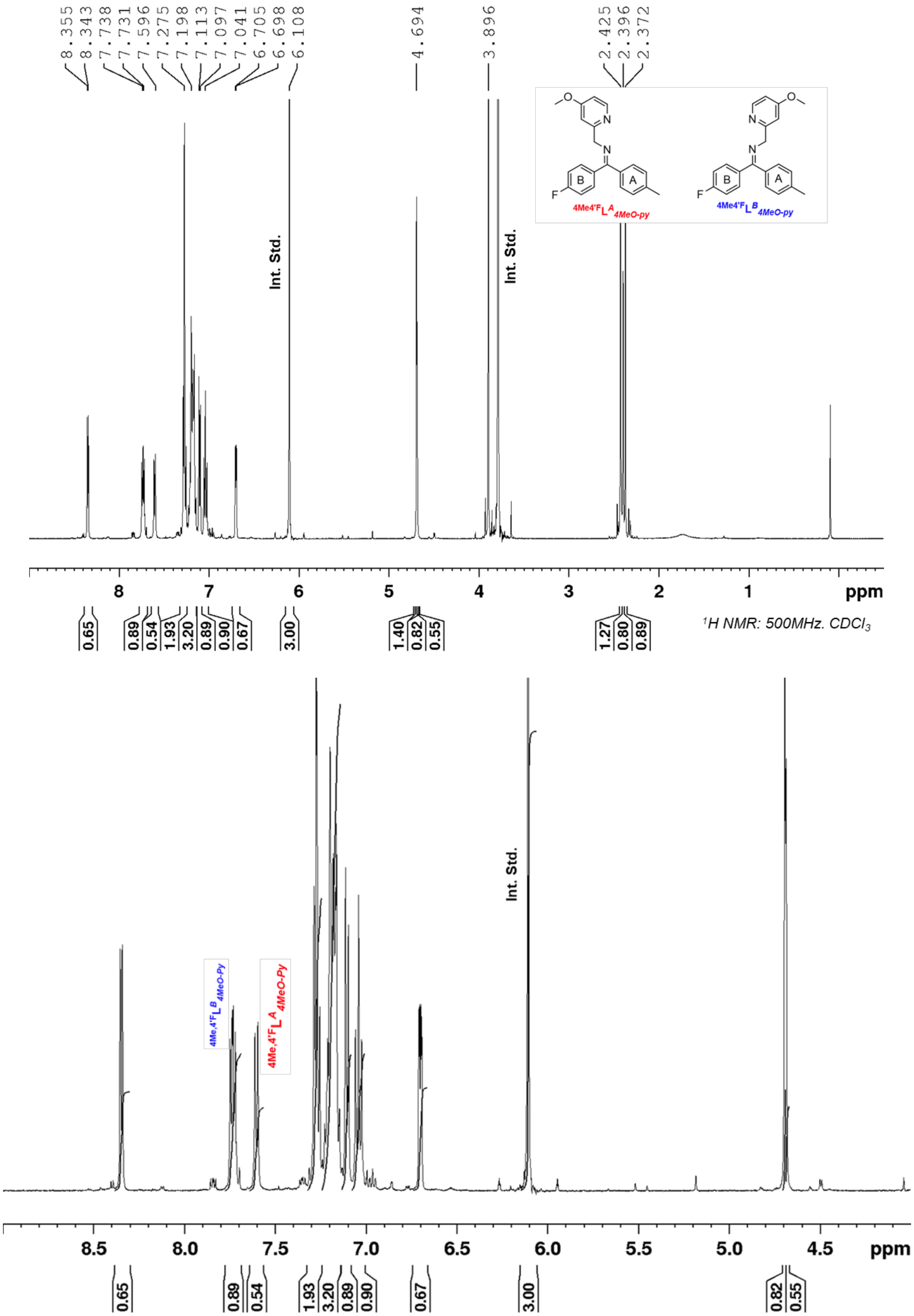

Figure S67. ^{1}H-NMR spectra of $^{4Me4'F}L_{4MeO\text{-}py}$ Note: There are two imine isomers. The ratio of $^{4Me4'F}L^{A}_{4MeO\text{-}py}$ and $^{4Me4'F}L^{B}_{4MeO\text{-}py}$ (43/57) is calculated using the average of the integration of CH_2 peaks and CH peaks.

^{1}H-NMR spectra for the hydroxylation of $^{4Me4'F}L_{4MeO\text{-}py}$

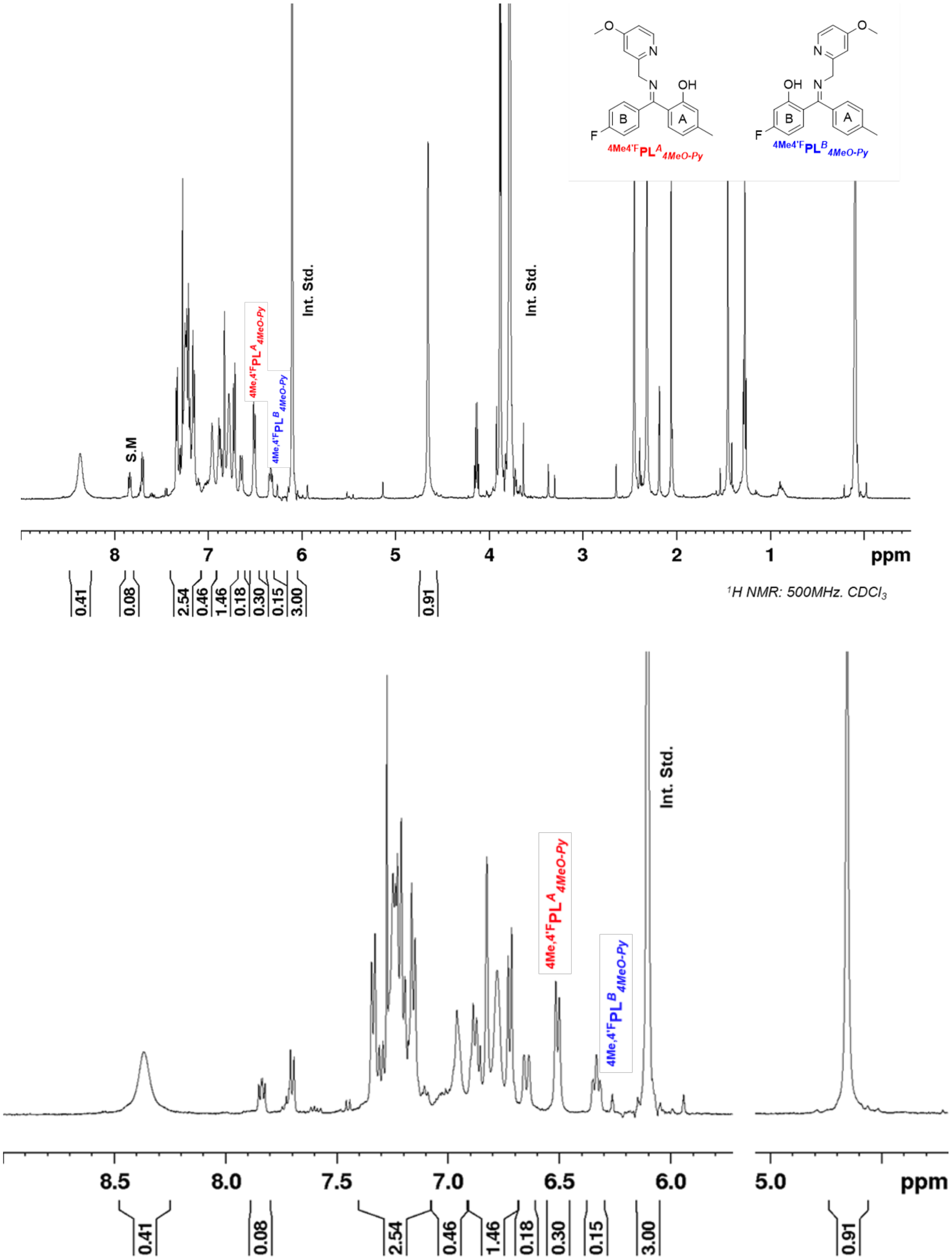

Figure S68. ^{1}H-NMR spectra for the hydroxylation of $^{4Me4'F}L_{4MeO\text{-}py}$. Note: The ratio of $^{4Me4'F}PL^{A}_{4MeO\text{-}py}$ and $^{4Me4'F}PL^{B}_{4MeO\text{-}py}$ (69/31) is calculated using the average of the integration of CH_2 peaks and CH peaks.

3.22 ^{4Cl}S and 2-(aminomethyl)-4-chloropyridine

Synthesis of $^{4Cl}L_{4Cl\text{-}py}$

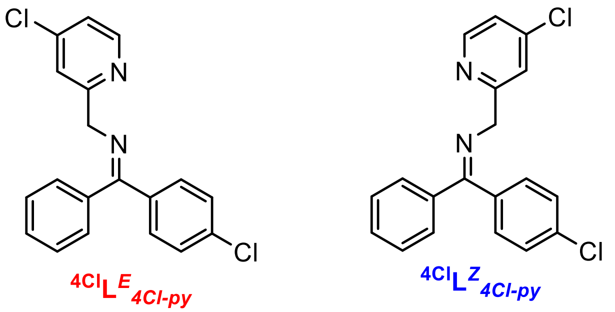

$^{4Cl}L^{E}_{4Cl\text{-}py}$ $^{4Cl}L^{Z}_{4Cl\text{-}py}$

In an oven dried flask, 2-(aminomethyl)-4-chloropyridine (2.2 equiv., 0.31 mL) was added to 4-chlorobenzophenone (300 mg, 1.38 mmol) and p-toluenesulfonic acid monohydrate (cat. 10 mg, 4.2 mol%) in toluene (30 mL). The reaction mixture was refluxed under argon with a Dean-Stark apparatus until imine formation was complete (5 days). The reaction was cooled to room temperature and diluted with diethyl ether (30 mL). The organic layer was washed with saturated ammonia chloride (20 mL x 2), saturated aqueous sodium bicarbonate (20 mL), brine (20 mL), and dried with magnesium sulfate. The final product was isolated as a brown solid (91% yield, 428 mg, 90% pure). ^{1}H-NMR (500 MHz, $CDCl_3$): δ 8.42 (d, 2H), 7.68 (t, 6H), 7.55-7.45 (m, L^B +L^A, 6H), 7.38 (m, L^B +L^A, 4H), 7.2 (m, L^B +L^A, 6H), 4.69 (d, L^B +L^A, 4H). HRMS (ESI) m/z: [M + Na]+ Calcd for C19H14Cl2N2Na 341.2350, found 343.1058.

Hydroxylation of $^{4Cl}L_{4Cl\text{-}py}$

The reaction was carried out on 0.079 mmol scale using 30.0 mg of the imine according to the Standard Procedure. The reaction products were quantified using 0.079 mmol of 1,3,5-trimethoxybenzene (int. std.). (42% yield). The identity of the hydroxylation products was confirmed by ^{1}H-NMR.

Cleavage of $^{4Cl}PL_{4Cl\text{-}py}$

Dissolving $^{4Cl}PL_{4Cl\text{-}Py}$ in round bottom flask with 50 mL EtOAc, then adding 100 mL 1M HCl. Reaction was going for 30 min. The resulting mixture was extracted with EtOAc (50 mL X 2). The organic phases were separated, combined, dried over $MgSO_4$, filtered, and dried under vacuum. The reaction products were dissolved in 1.4 mL of $CDCl_3$ solution containing 13.5 mg of 1,3,5-trimethoxybenzene (internal standard). The reaction products were quantified by ^{1}H-NMR using integration signals that correspond to the starting material and products with the integration signal of the internal standard.

^{1}H-NMR spectra of $^{4Cl}L_{4Cl\text{-}py}$

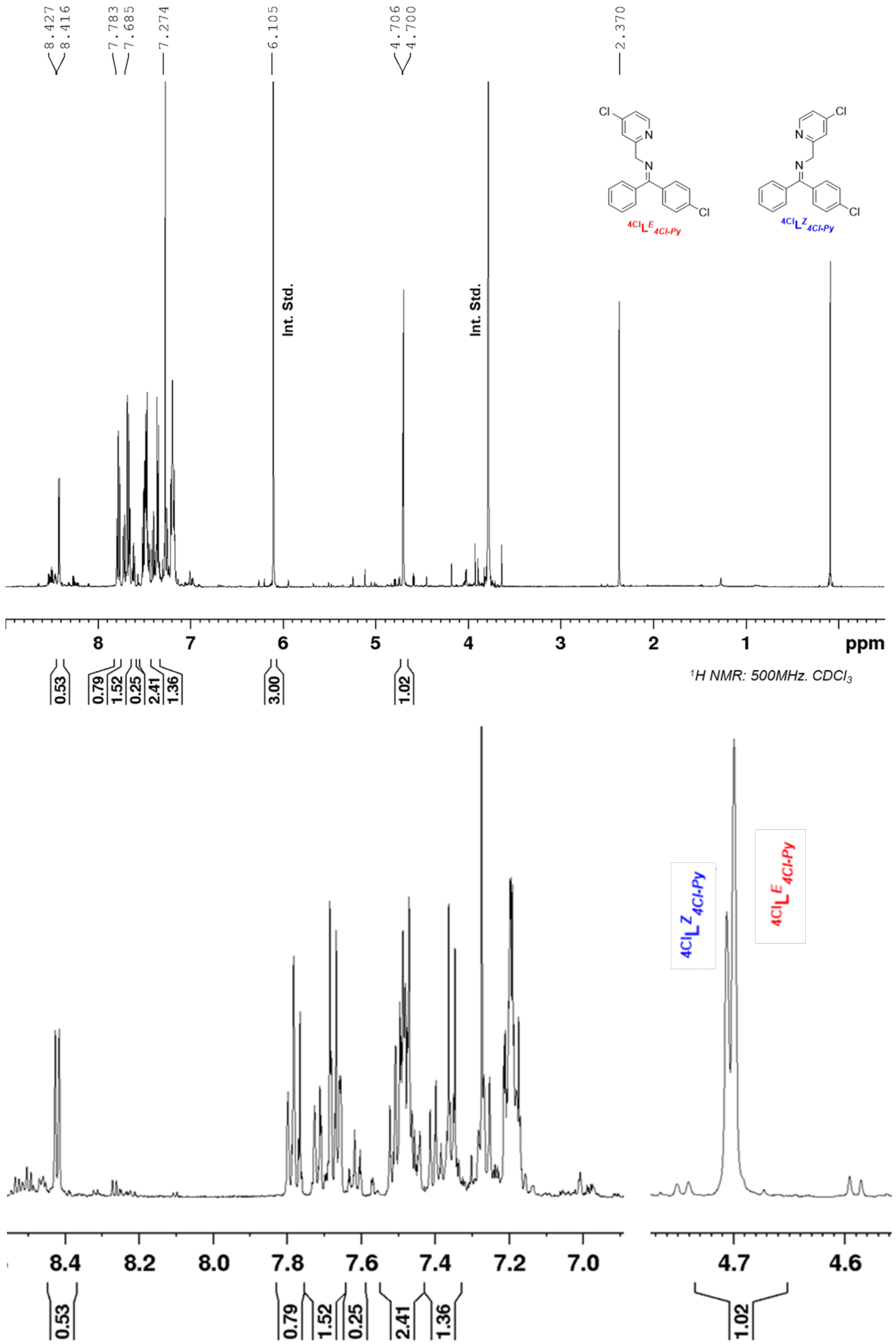

Figure S69. ^{1}H-NMR spectra of $^{4Cl}L_{4Cl\text{-}py}$ Note: There are two imine isomers. The ratio of $^{4Cl}L^{E}_{4Cl\text{-}py}$ and $^{4MeO}L^{Z}_{4Cl\text{-}py}$ (62/38) is calculated using the average of the integration of CH_2 peaks and CH peaks.

^{1}H-NMR spectra for the hydroxylation of $^{4Cl}L_{4Cl-py}$

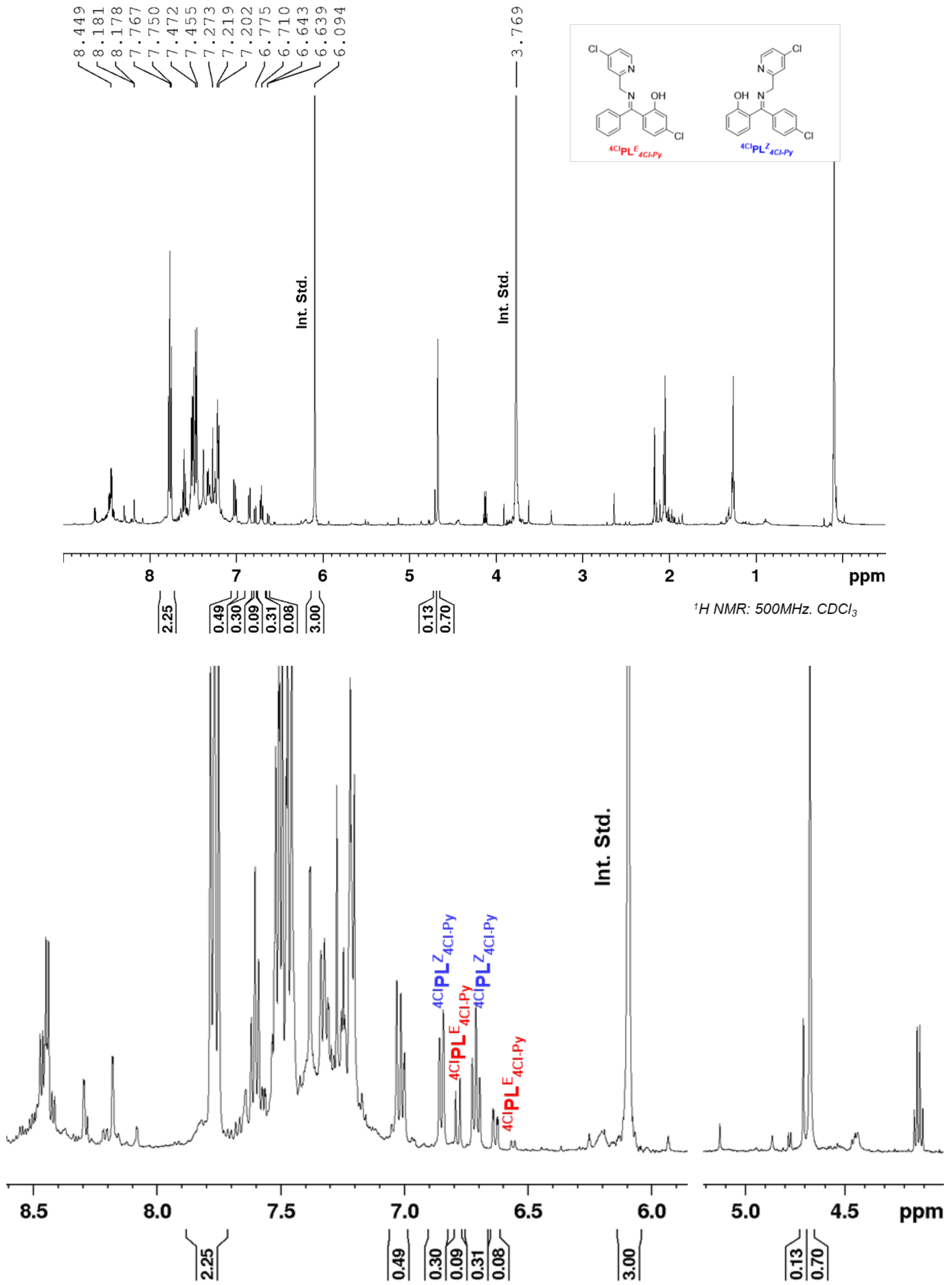

Figure S70. ^{1}H-NMR spectra for the hydroxylation of $^{4Cl}L_{4Cl-py}$. Note: The ratio of $^{4Cl}PL^{E}_{4Cl-py}$ and $^{4MeO}PL^{Z}_{4MeO-py}$ (19/81) is calculated using the average of the integration of CH_2 peaks and CH peaks.

^{1}H-NMR spectra for the cleavage of ^{4Cl}PL$_{4Cl\text{-}py}$

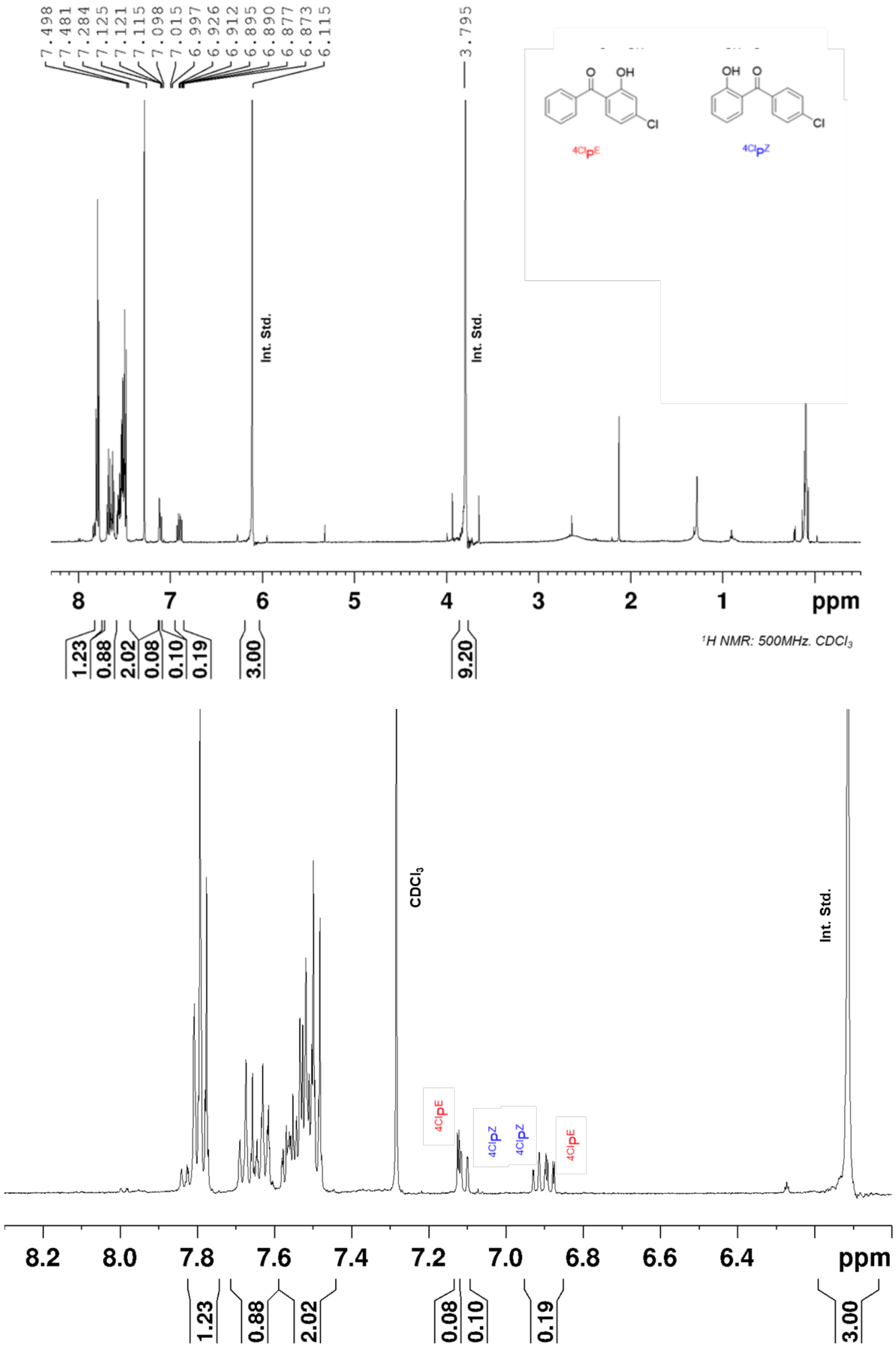

Figure S71. ^{1}H-NMR spectra for the cleavage of ^{4Cl}PL$_{4Cl\text{-}py}$. Note: The ratio of ^{4Cl}P^{E} and ^{4Cl}P^{Z} could not be calculated due to the overlap between the product peaks.

3.23 ^{4MeO}S and 2-(aminomethyl)-4-chloropyridine

Synthesis of $^{4MeO4'Cl}L_{4Cl-py}$

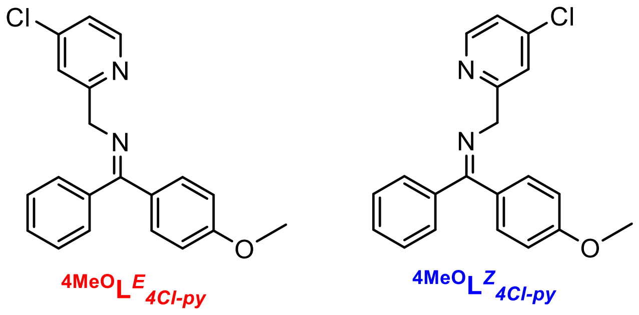

In an oven dried flask, 2-(aminomethyl)-4-chloropyridine (2.2 equiv., 0.31 mL) was added to 4-Methoxybenzophenone (300 mg, 1.38 mmol) and p-toluenesulfonic acid monohydrate (cat. 10 mg, 4.2 mol%) in toluene (30 mL). The reaction mixture was refluxed under argon with a Dean-Stark apparatus until imine formation was complete (5 days). The reaction was cooled to room temperature and diluted with diethyl ether (30 mL). The organic layer was washed with saturated ammonia chloride (20 mL x 2), saturated aqueous sodium bicarbonate (20 mL), brine (20 mL), and dried with magnesium sulfate. The final product was isolated as a brown solid (85% yield, 394 mg, 86% pure). ^{1}H-NMR (500 MHz, $CDCl_3$): δ 8.41 (d, 2H), 7.80-7.60 (m, L^E +L^Z , 6H) 7.50-7.40 (m, L^E +L^Z , 4H), 7.30-7.15 (m, 8H), 6.99 (t, L^Z ,2H), 6.89 (d, , L^E ,2H), 4.78 (s, , L^E, 2H), 4.67 (s, L^Z, 2H). 3.87 (s, L^Z , 3H), 3.84 (s, L^E, 3H). HRMS (ESI) m/z: [M + Na]+ Calcd for C20H17ClN2ONa 336.8190, found 337.1143.

Hydroxylation of $^{4MeO}L_{4Cl-py}$

The reaction was carried out on 0.079 mmol scale using 30.9 mg of the imine according to the Standard Procedure. The reaction products were quantified using 0.079 mmol of 1,3,5-trimethoxybenzene (int. std.). (40% yield). The identity of the hydroxylation products was confirmed by ^{1}H-NMR.

Cleavage of $^{4MeO}PL_{4Cl-py}$

Dissolving $^{4MeO}PL_{4Cl\text{-}Py}$ in round bottom flask with 50 mL EtOAc, then adding 100 mL 1M HCl. Reaction was going for 30 min. The resulting mixture was extracted with EtOAc (50 mL X 2). The organic phases were separated, combined, dried over $MgSO_4$, filtered, and dried under vacuum. The reaction products were dissolved in 1.4 mL of $CDCl_3$ solution containing 13.5 mg of 1,3,5-trimethoxybenzene (internal standard). The reaction products were quantified by ^{1}H-NMR using integration signals that correspond to the starting material and products with the integration signal of the internal standard.

^{1}H-NMR spectra of $^{4MeO}L_{4Cl\text{-}py}$

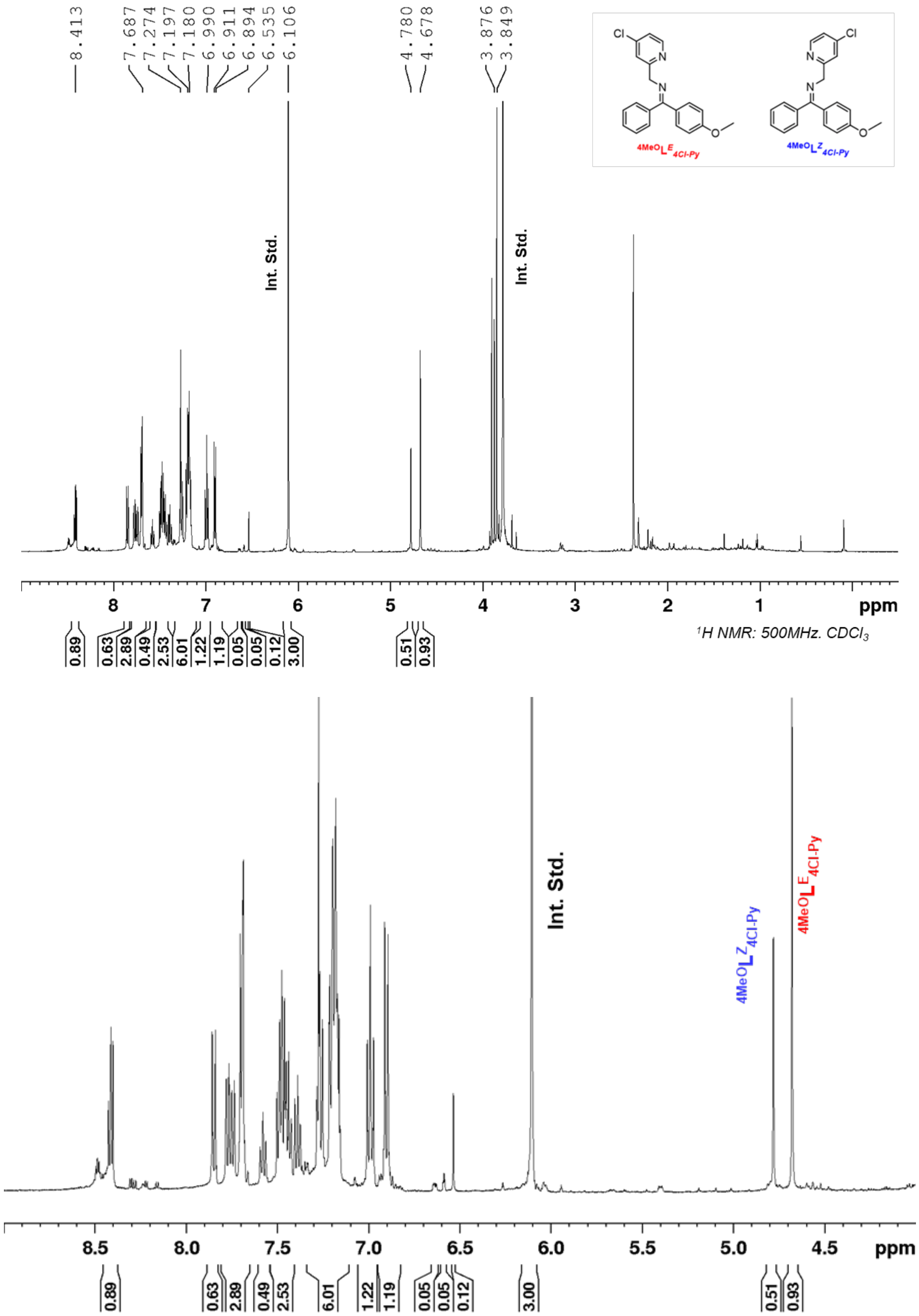

Figure S72. ^{1}H NMR spectra of $^{4MeO}L_{4Cl\text{-}py}$. Note: Two imine isomers were formed. The ratio of $^{4MeO}L^{E}_{4Cl\text{-}py}$ and $^{4MeO}L^{Z}_{4Cl\text{-}py}$ (65/35) is calculated using the average of the integration of CH_2 peaks and CH peaks.

^{1}*H-NMR spectra for the hydroxylation* $^{4MeO}L_{4Cl\text{-}py}$

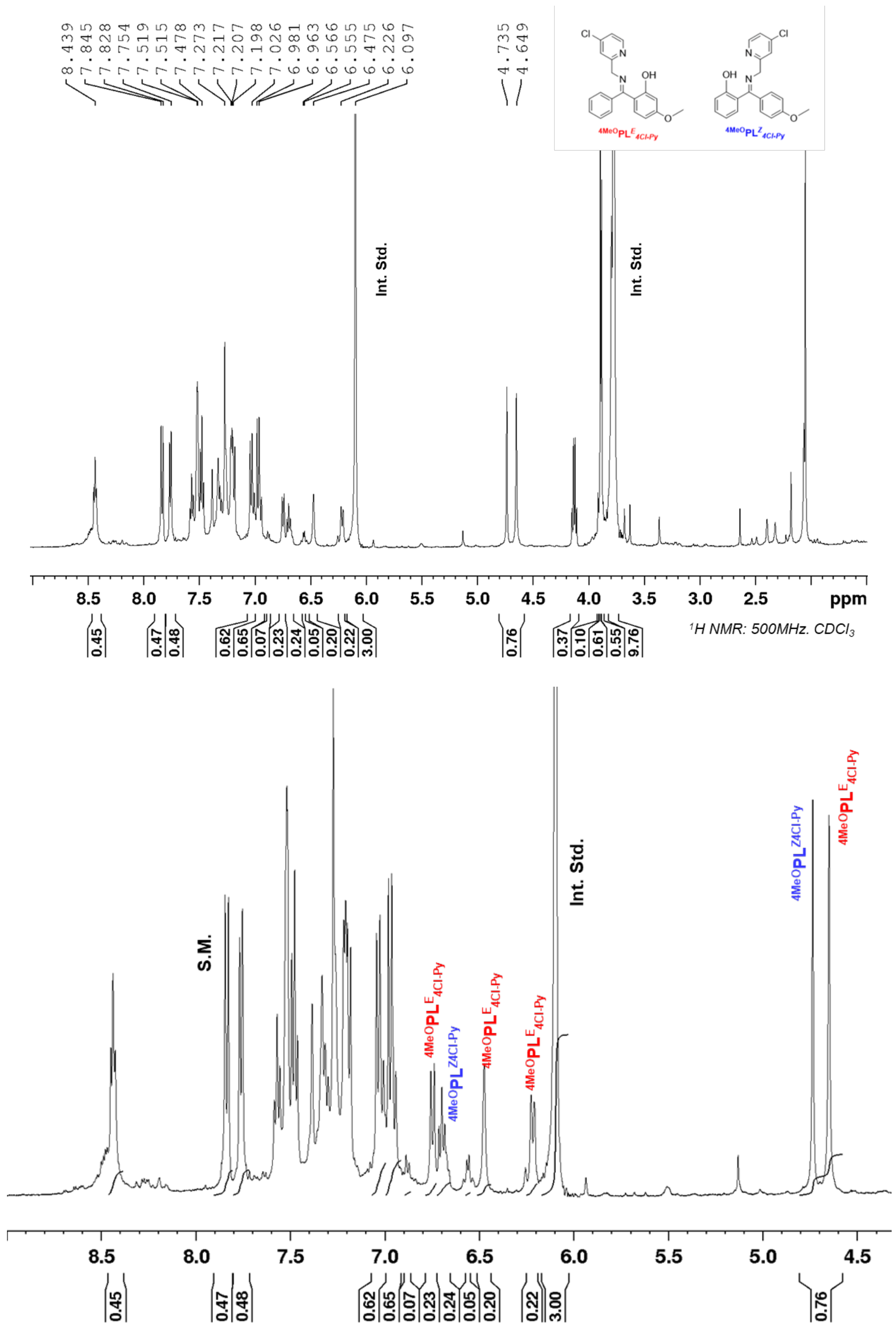

Figure S73. ^{1}H-NMR spectra for the hydroxylation of $^{4MeO}L_{4Cl\text{-}py}$. Note: The ratio of $^{4MeO}PL^{E}_{4Cl\text{-}py}$ and $^{4MeO}PL^{Z}_{4MeO\text{-}py}$ (52/48) is calculated using the average of the integration of CH_2 peaks and CH peaks.

^{1}H-NMR spectra for the cleavage of ${}^{4MeO}PL_{4Cl\text{-}py}$

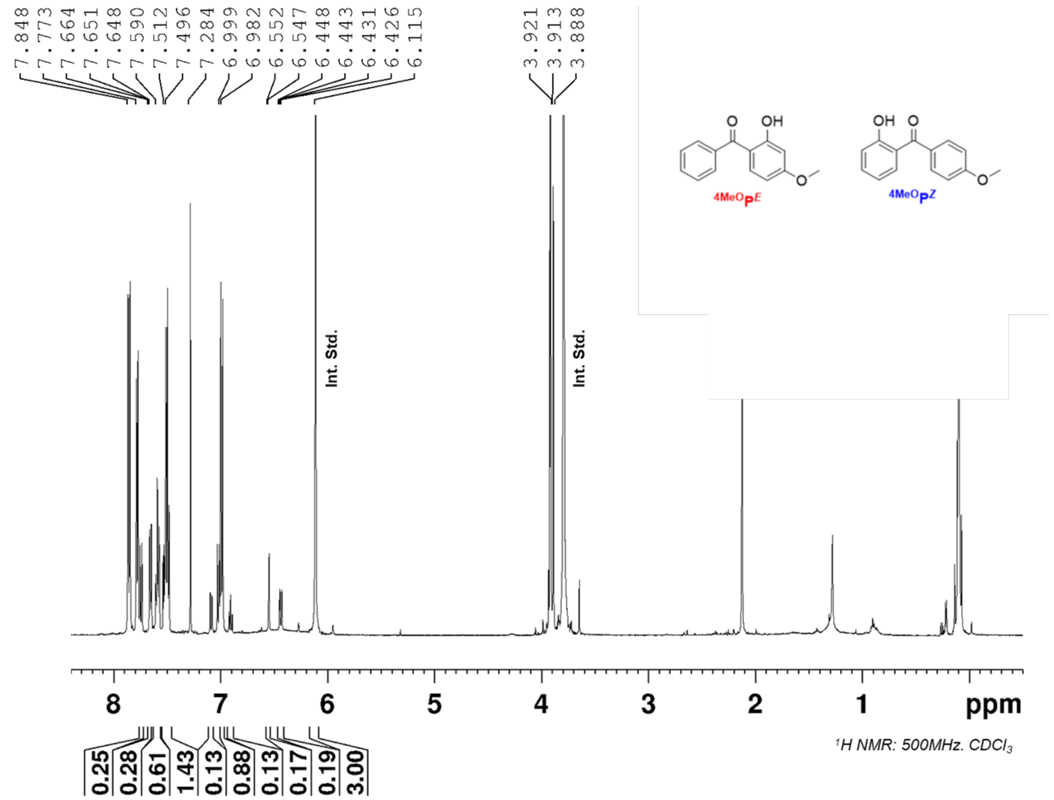

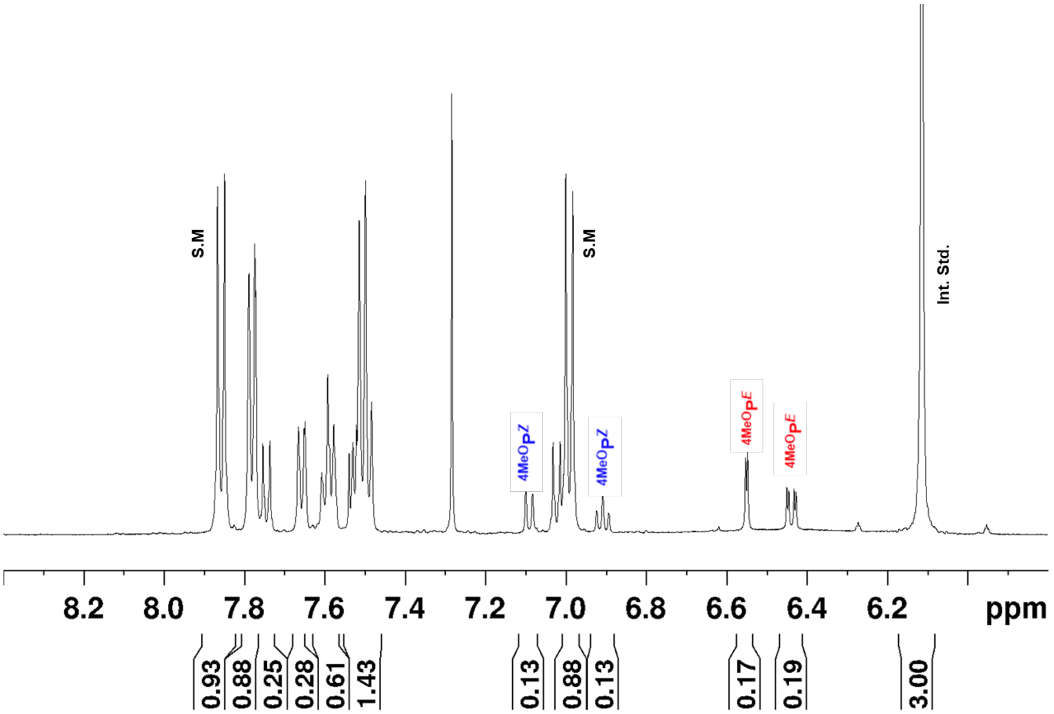

Figure S74. ^{1}H-NMR spectra for the cleavage of ${}^{4MeO}PL_{4Cl\text{-}py}$**.** Note: The ratio of ${}^{4MeO}P^{E}$ and ${}^{4MeO}P^{Z}$ (58/42) is calculated using the average of the integration of CH peaks.

3.24 ^{4CF3}S and 2-(aminomethyl)-4-chloropyridine

Synthesis of $^{4CF3}L_{4Cl\text{-}py}$

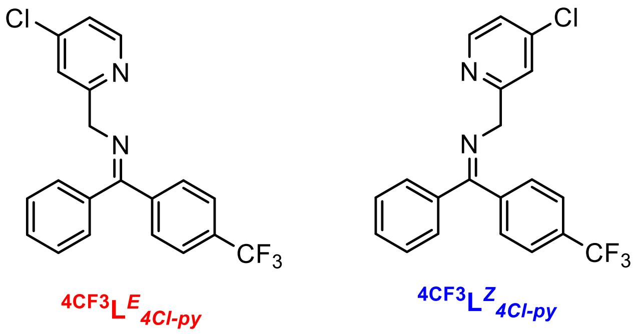

In an oven dried flask, 2-(aminomethyl)-4-chloropyridine (2.2 equiv., 0.18 mL) was added to 4-Methoxybenzophenone (200 mg, 0.80 mmol) and p-toluenesulfonic acid monohydrate (cat. 10 mg, 7 mol%) in toluene (30 mL). The reaction mixture was refluxed under argon with a Dean-Stark apparatus until imine formation was complete (7 days). The reaction was cooled to room temperature and diluted with diethyl ether (30 mL). The organic layer was washed with saturated ammonia chloride (20 mL x 2), saturated aqueous sodium bicarbonate (20 mL), brine (20 mL), and dried with magnesium sulfate. The final product was isolated as a brown solid (75% yield, 224 mg, 82% pure). ^{1}H-NMR (500 MHz, $CDCl_3$): δ 8.45 (d, 1H),8.43 (d, 1H) 7.86 (d, 2H), 7.83 (d, 2H), 7.79(d, L^E +L^Z ,2H), 7.71 (d, 2H), 7.65(t, 2H), 7.60-7.46 (m, L^E +L^Z ,4H), 7.40 -7.30(m, 4H), 7.24-7.20(m, L^E +L^Z ,4H), 4.75 (s, , L^E, 2H), 4.67 (s, L^Z, 2H). HRMS (ESI) m/z: [M + Na]+ Calcd for C20H14ClF3N2Na 374.7912, found 376.5468.

Hydroxylation of $^{4CF3}L_{4Cl\text{-}py}$

The reaction was carried out on 0.079 mmol scale using 36.0 mg of the imine according to the Standard Procedure. The reaction products were quantified using 0.079 mmol of 1,3,5-trimethoxybenzene (int. std.). (30% yield). The identity of the hydroxylation products was confirmed by ^{1}H-NMR.

^{1}H-NMR spectra of $^{4CF3}L_{4Cl\text{-}py}$

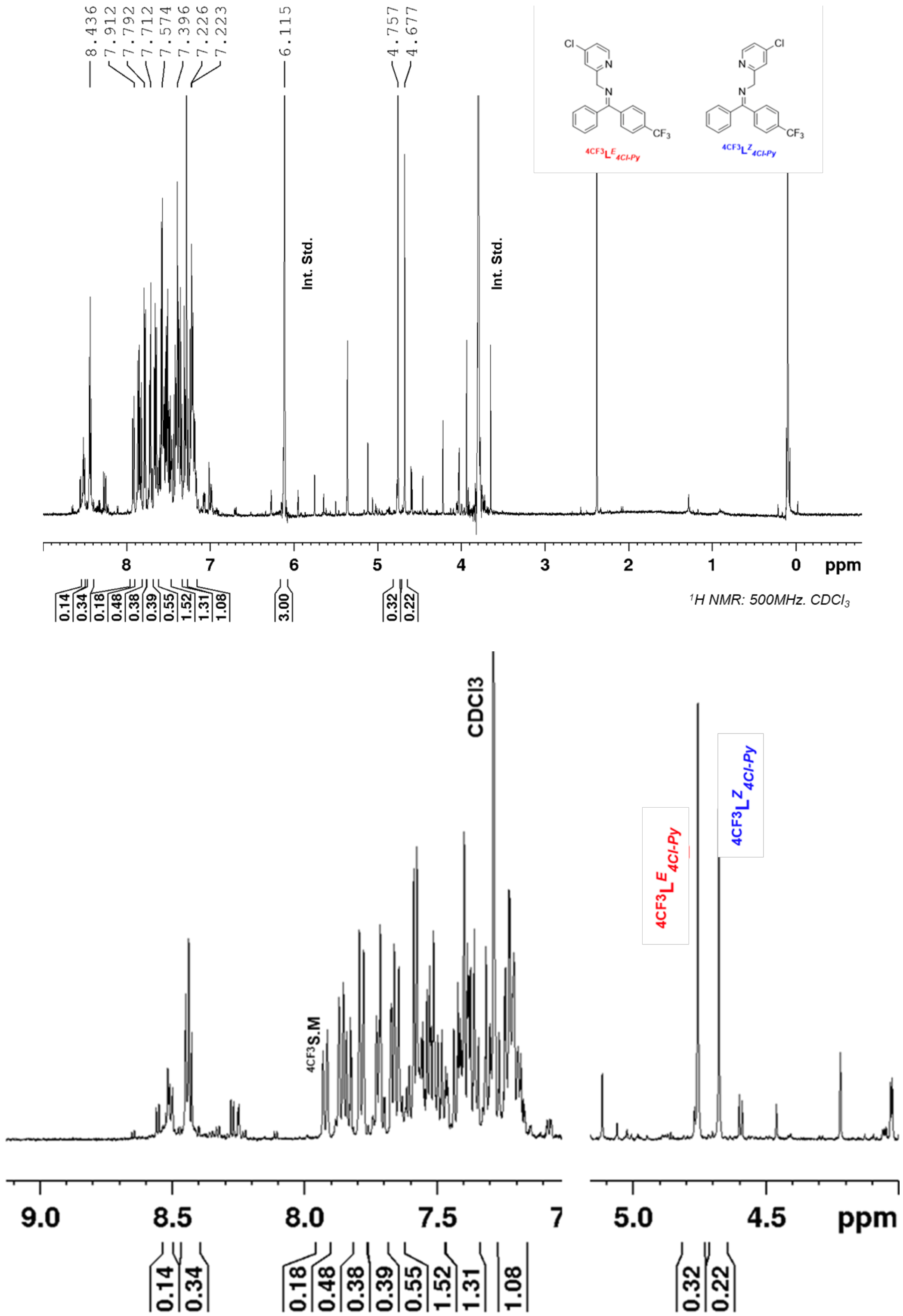

Figure S75. ^{1}H-NMR of $^{4CF3}L_{4Cl\text{-}py}$ Note: two imine isomers were formed. The ratio of $^{4CF3}L^E_{4Cl\text{-}py}$ and $^{4CF3}L^Z_{4Cl\text{-}py}$ (61/39) is calculated using the average of the integration of CH_2 peaks and CH peaks.

^{1}H-NMR spectra for the hydroxylation of $^{4CF3}L_{4Cl-py}$

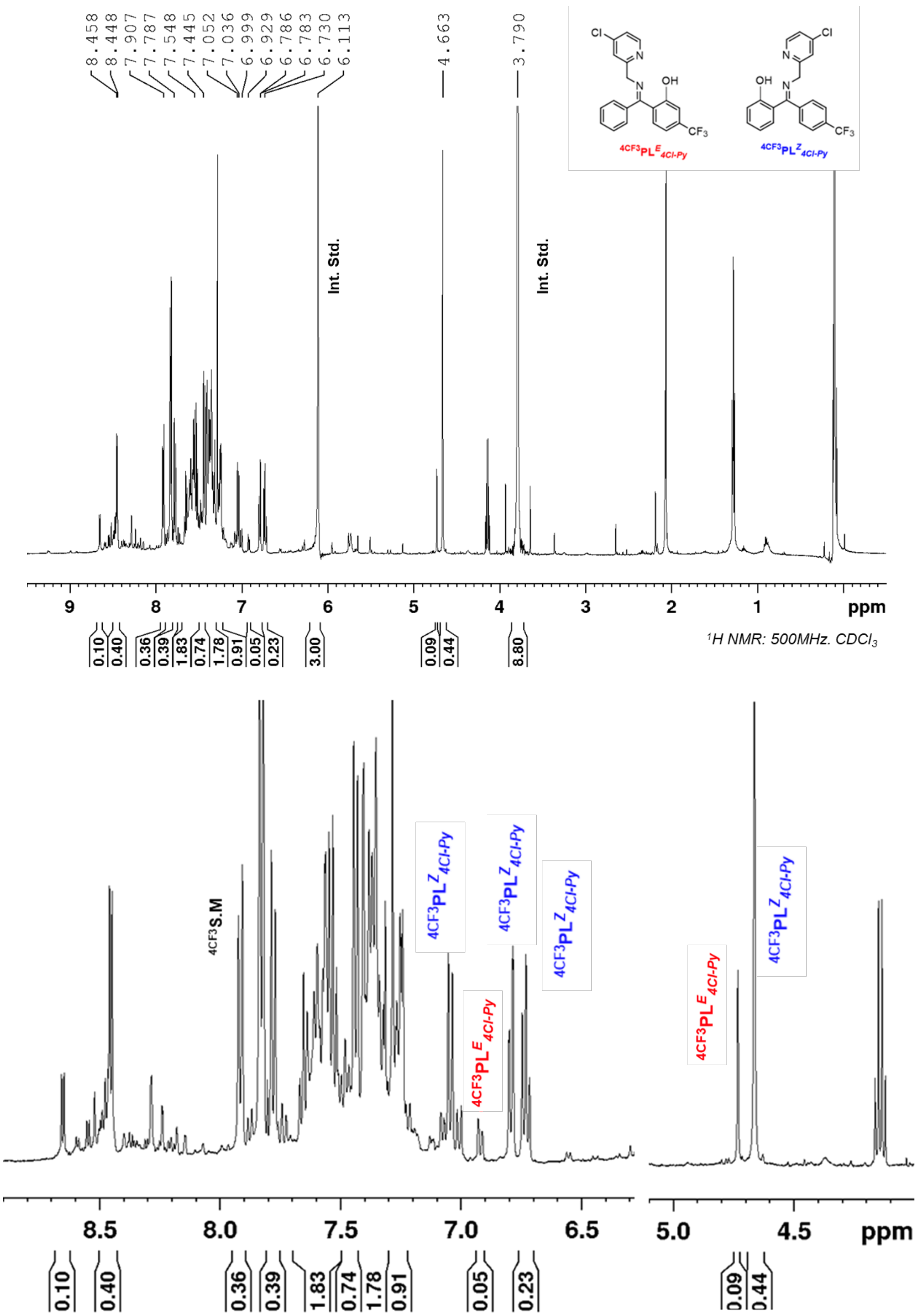

Figure S76. ^{1}H-NMR spectra for the hydroxylation of $^{4CF3}L_{4Cl\text{-}py}$. Note: The ratio of $^{4CF3}PL^{E}_{4Cl\text{-}py}$ and $^{4CF3}PL^{Z}_{4MeO\text{-}py}$ (16/84) is calculated using the average of the integration of CH_2 peaks and CH peaks.

3.25 ^{4CF3}S and 2-(aminomethyl)-4-chloropyridine

Synthesis of ^{4CF3}L$_{4Cl\text{-}py}$

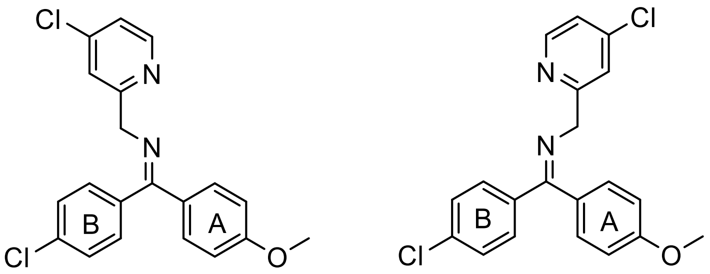

$^{4MeO4'Cl}$L$^{A}_{4Cl\text{-}py}$ $^{4MeO4'Cl}$L$^{B}_{4Cl\text{-}py}$

In an oven dried flask, 2-(aminomethyl)-4-chloropyridine (2.2 equiv., 0.31 mL) was added to 4-Methoxybenzophenone (300 mg, 1.38 mmol) and p-toluenesulfonic acid monohydrate (cat. 10 mg, 4.2 mol%) in toluene (30 mL). The reaction mixture was refluxed under argon with a Dean-Stark apparatus until imine formation was complete (5 days). The reaction was cooled to room temperature and diluted with diethyl ether (30 mL). The organic layer was washed with saturated ammonia chloride (20 mL x 2), saturated aqueous sodium bicarbonate (20 mL), brine (20 mL), and dried with magnesium sulfate. The final product was isolated as a brown solid (86% yield, 444 mg, 85% pure). ^{1}H-NMR (500 MHz, $CDCl_3$): δ 8.42 (d, 2H), 7.66 (td, 4H), 7.40-7.09 (m, L^B +L^A , 12H) 7.0 (d, L^B +L^A , 2H), 6.90 (d, ,2H), 4.75 (s, L^B , 2H), 4.66 (s, L^A , 2H), 3.85-3.78 (m, L^B +L^A, 6H). HRMS (ESI) m/z: [M + Na]+ Calcd for C20H16Cl2N2ONa 371.2670, found 373.1067

Hydroxylation of $^{4MeO4'Cl}$L$_{4Cl\text{-}py}$

The reaction was carried out on 0.079 mmol scale using 34.5 mg of the imine according to the Standard Procedure. The reaction products were quantified using 0.079 mmol of 1,3,5-trimethoxybenzene (int. std.). (50% yield). The identity of the hydroxylation products was confirmed by ^{1}H-NMR.

Cleavage of $^{4MeO4'Cl}$PL$_{4Cl\text{-}py}$

Dissolving $^{4MeO4'Cl}$PL$_{4Cl\text{-}py}$ in round bottom flask with 50 mL EtOAc, then adding 100 mL 1M HCl. Reaction was going for 30 min. The resulting mixture was extracted with EtOAc (50 mL X 2). The organic phases were separated, combined, dried over $MgSO_4$, filtered, and dried under vacuum. The reaction products were dissolved in 1.4 mL of $CDCl_3$ solution containing 13.5 mg of 1,3,5-trimethoxybenzene (internal standard). The reaction products were quantified by ^{1}H-NMR using integration signals that correspond to the starting material and products with the integration signal of the internal standard.

^{1}*H-NMR spectra of* $^{4MeO4'Cl}L_{4Cl\text{-}py}$

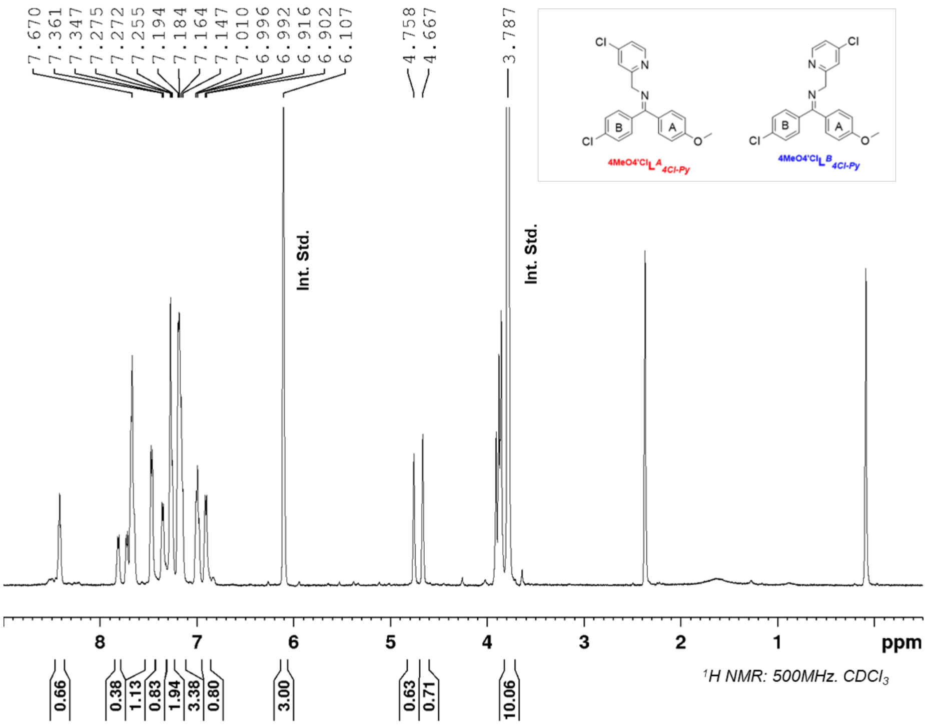

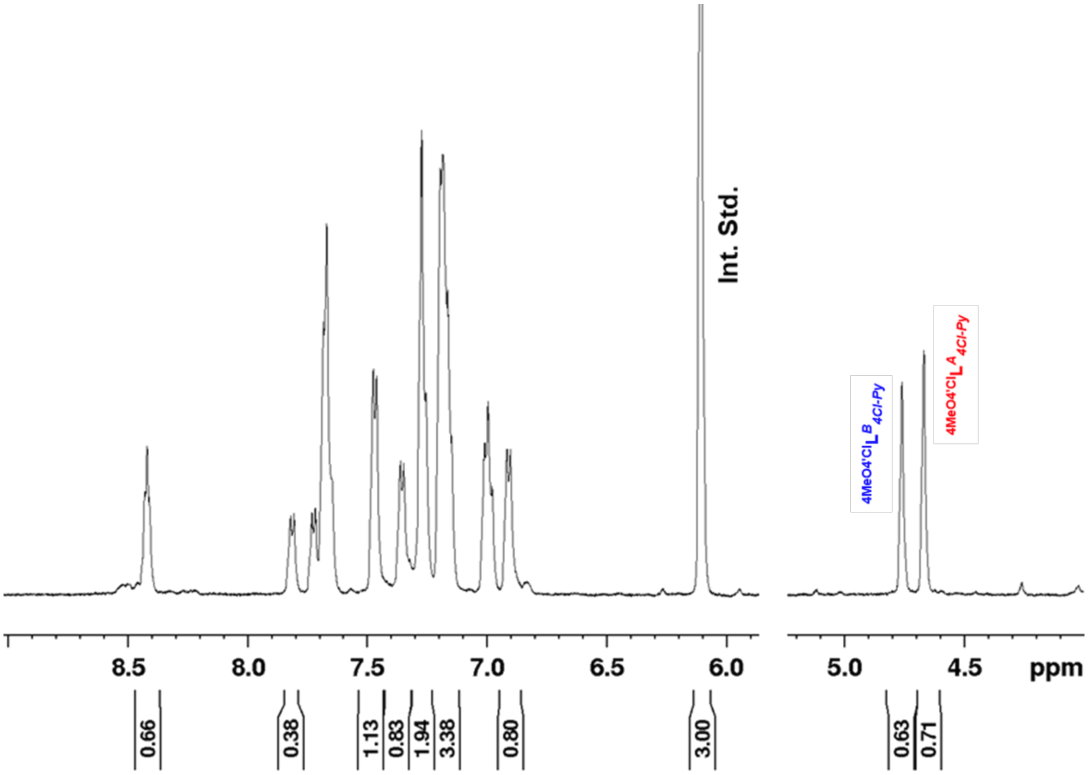

Figure S77. ^{1}H-NMR spectra of $^{4MeO4'Cl}L_{4Cl\text{-}py}$. Note: There are two imine isomers. The ratio of $^{4MeO4'Cl}L^{A}_{4Cl\text{-}py}$ and $^{4MeO4'Cl}L^{B}_{4Cl\text{-}py}$ (53/47) is calculated using the average of the integration of CH_2 peaks and CH peaks.

^{1}*H-NMR spectra for the hydroxylation* $^{4MeO4'Cl}L_{4Cl\text{-}py}$

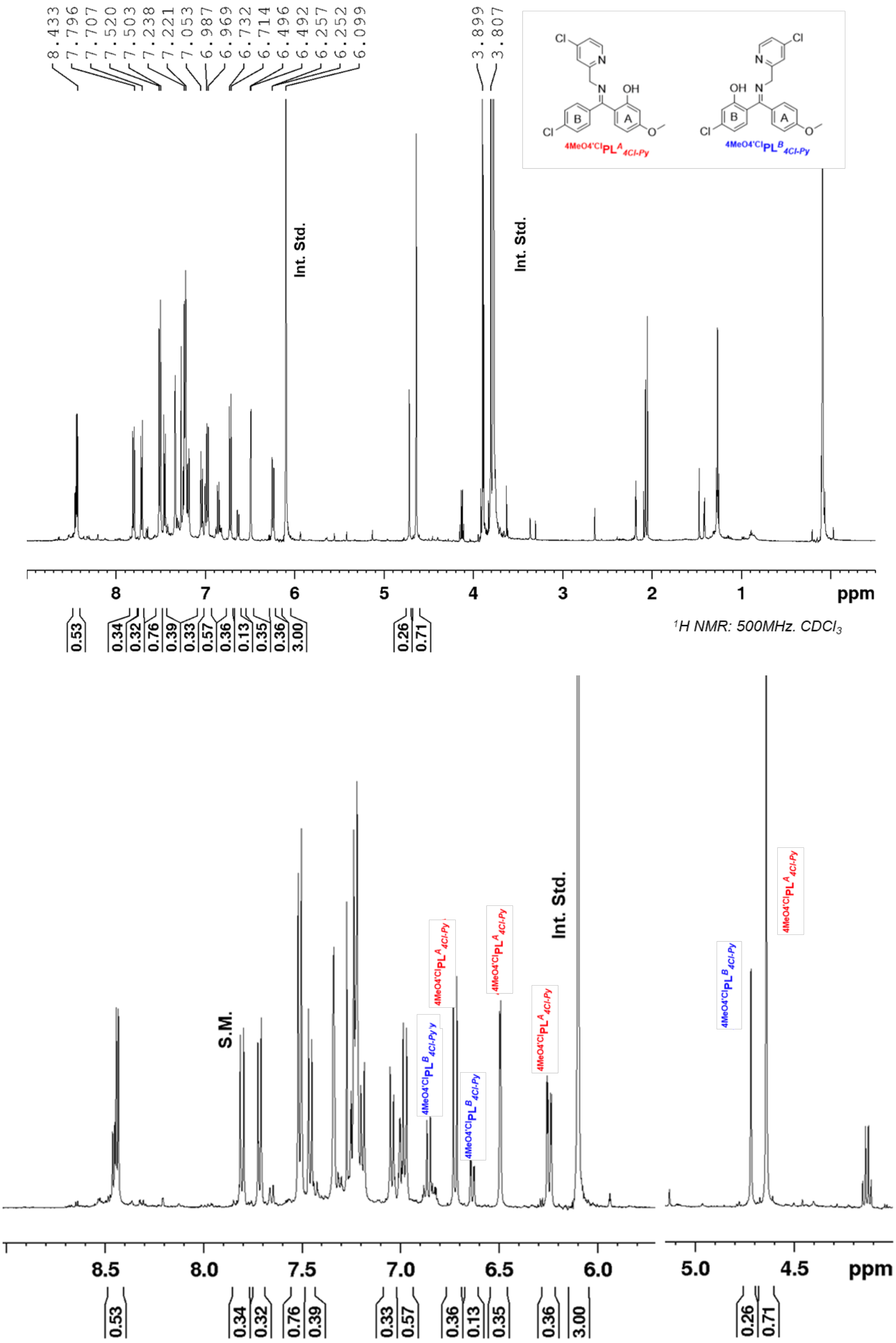

Figure S78. ^{1}H-NMR spectra for the hydroxylation of $^{4MeO4'Cl}L_{4Cl\text{-}py}$. Note: The ratio of $^{4MeO4'Cl}PL^{A}_{4Cl\text{-}py}$ and $^{4MeO4'Cl}PL^{B}_{4MeO\text{-}py}$ (74/26) is calculated using the average of the integration of CH_2 peaks and CH peaks.).

^{1}H-NMR spectra for the cleavage of $^{4MeO4'Cl}$PL$_{4Cl\text{-}py}$

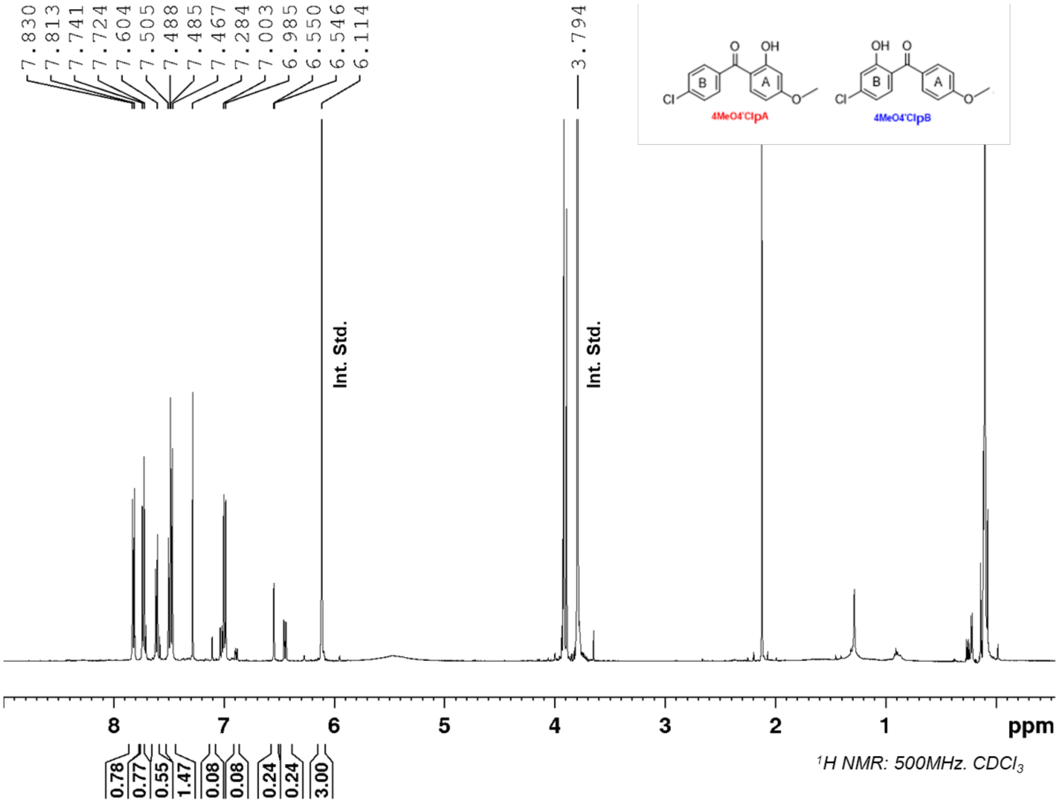

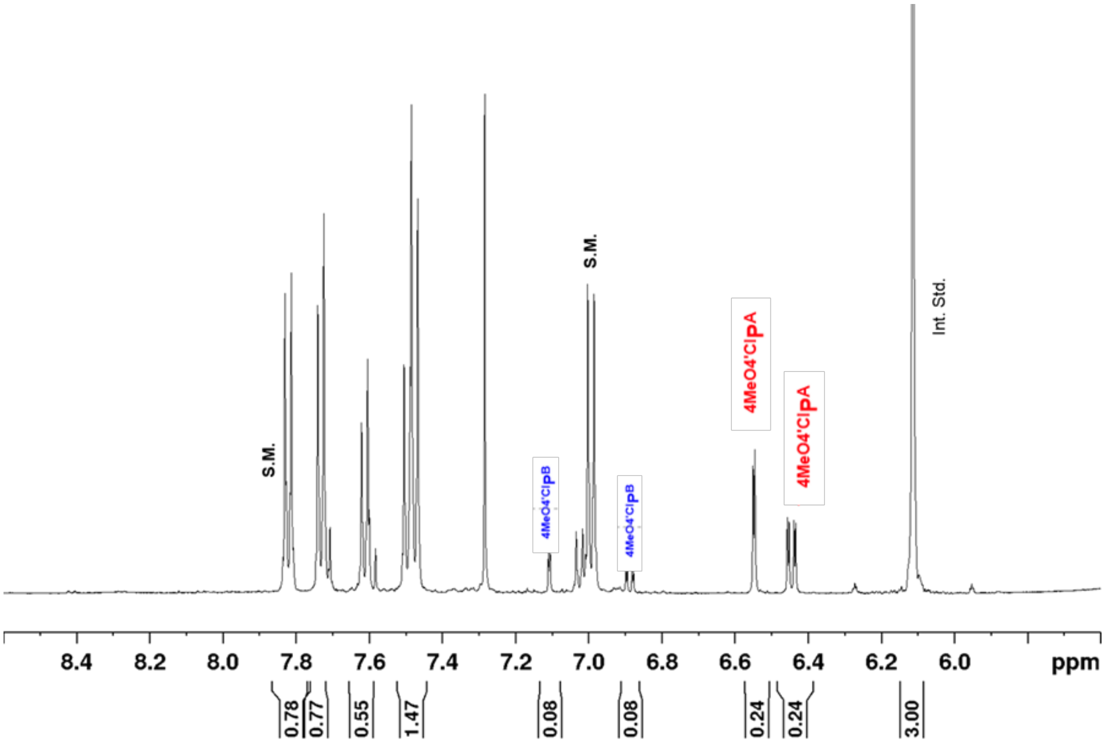

Figure S79. ^{1}H-NMR spectra for the cleavage of $^{4MeO4'Cl}$PL$_{4Cl\text{-}py}$**.** Note: The ratio of $^{4MeO4'Cl}$P^{A} and $^{4MeO4'Cl}$P^{B} (75/25) is calculated using the average of the integration of CH peaks.

3.26 ^{4CF3}S and 2-(aminomethyl)-4-(trifluoromethyl)pyridine

Synthesis of $^{4CF3}L_{4CF3\text{-}py}$

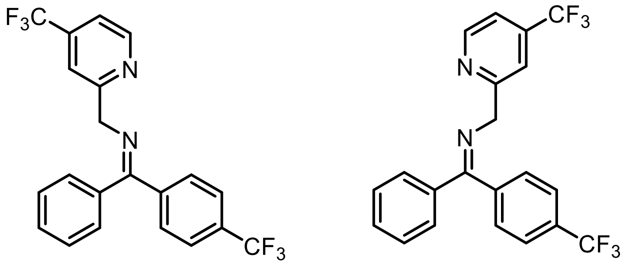

$^{4CF3}L^{E}_{4CF3\text{-}py}$ $^{4CF3}L^{Z}_{4CF3\text{-}py}$

In an oven dried flask, 2-(aminomethyl)-4-(trifluoromethyl)pyridine (2.2 equiv., 0.18 mL) was added to 4-Trifluoromethylbenzophenone (200 mg, 0.8 mmol) and p-toluenesulfonic acid monohydrate (cat. 10 mg, 7.2 mol%) in toluene (30 mL). The reaction mixture was refluxed under argon with a Dean-Stark apparatus until imine formation was complete (2 days). The reaction was cooled to room temperature and diluted with diethyl ether (30 mL). The organic layer was washed with saturated ammonia chloride (20 mL x 2), saturated aqueous sodium bicarbonate (20 mL), brine (20 mL), and dried with magnesium sulfate. The final product was isolated as a brown solid (75% yield, 244 mg, 80% pure). ^{1}H-NMR (500 MHz, $CDCl_3$): δ 8.72 (t, 2H), 7.94 (d, 2H), 7.85 (d, 2H), 7.78 (d, 2H), 7.66 (d, 2H), 7.52 (m, L^E + L^Z, 6H), 7.33 (m , L^E + L^Z , 6H), 7.25 (d, 2H), 4.83 (s, L^E, 2H), 4.75 (s, L^Z, 2H). HRMS (ESI) m/z: [M + Na]+ Calcd for C21H14F6N2Na 408.3474, found 409.1092.

Hydroxylation of $^{4CF3}L_{4CF3\text{-}py}$

The reaction was carried out on 0.079 mmol scale using 40.3 mg of the imine according to the Standard Procedure. The reaction products were quantified using 0.079 mmol of 1,3,5-trimethoxybenzene (int. std.). (50% yield). The identity of the hydroxylation products was confirmed by ^{1}H-NMR.

^{1}H-NMR spectra of $^{4CF3}L_{4CF3\text{-}py}$

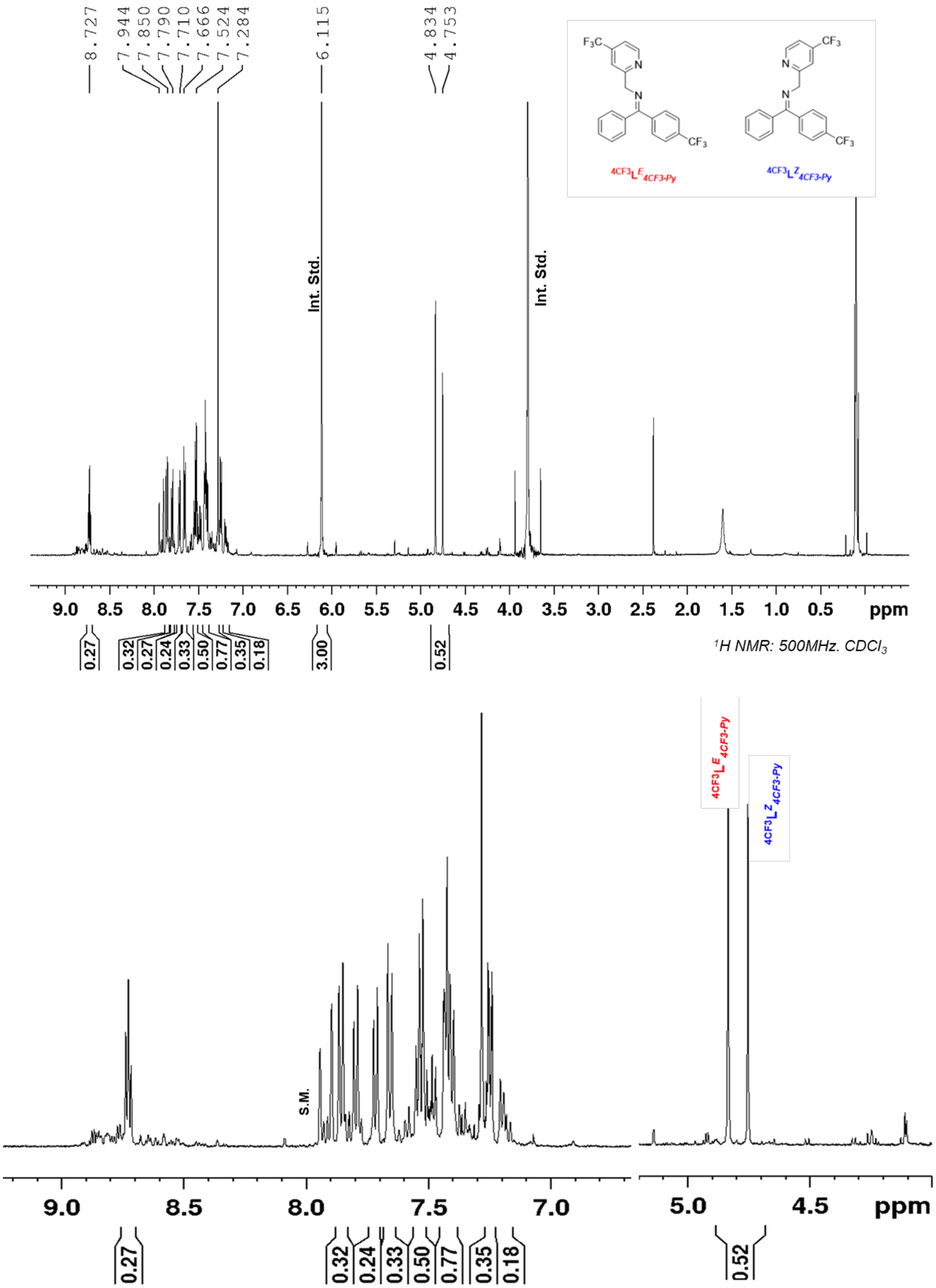

Figure S80. ^{1}H-NMR spectra of $^{4CF3}L_{4CF3\text{-}py}$. Note: two imine isomers were formed. The ratio of $^{4CF3}L^{E}_{4CF3\text{-}py}$ and $^{4CF3}L^{Z}_{4CF3\text{-}py}$ (60/40) is calculated using the average of the integration of CH_2 peaks and CH peaks.

^{1}H-NMR spectra for the hydroxylation of $^{4CF3}L_{4CF3-py}$

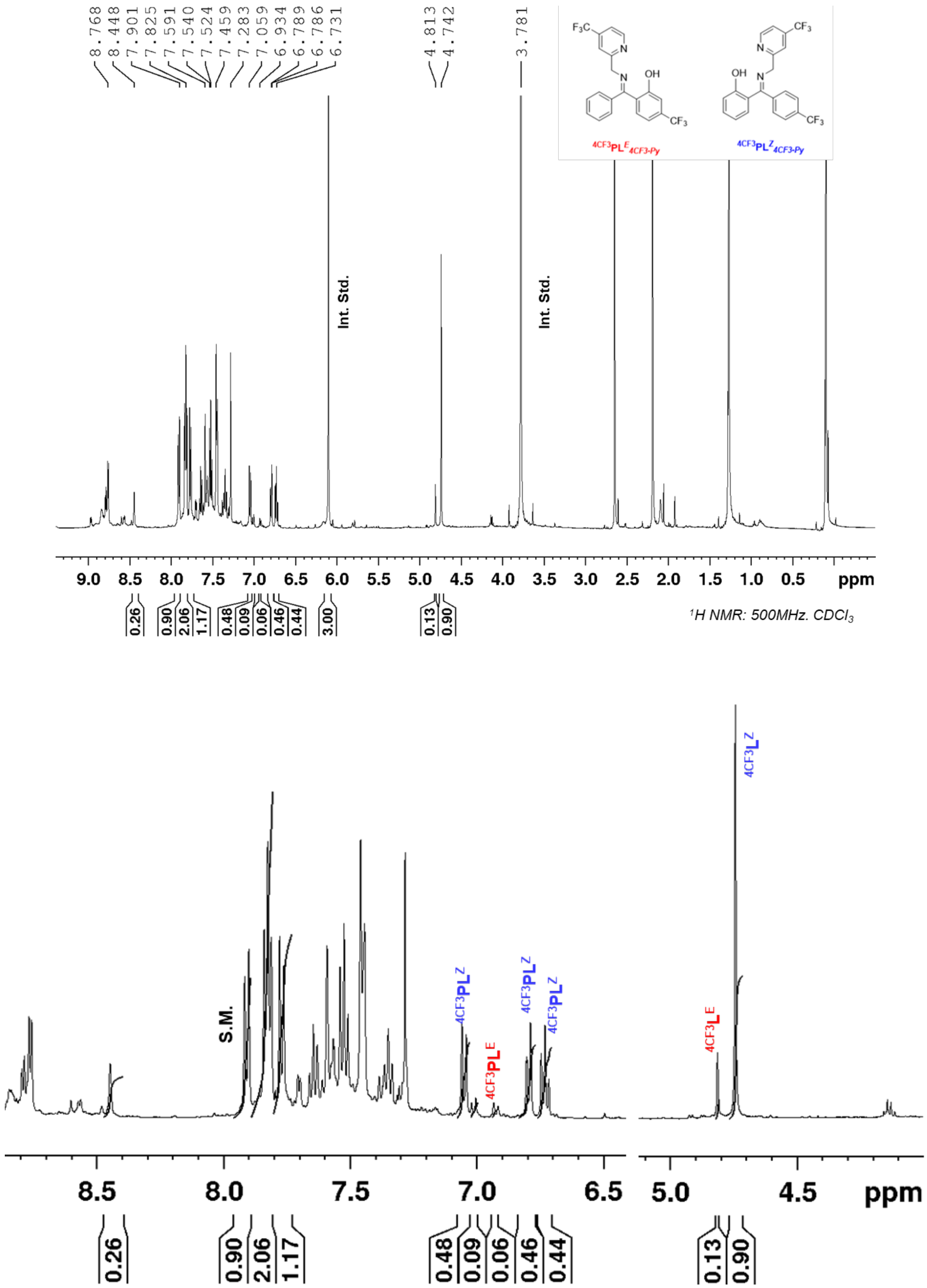

Figure S81. ^{1}H-NMR spectra for the hydroxylation of $^{4CF3}L_{4CF3-py}$. Note: The ratio of $^{4CF3}PL^E{}_{4CF3-py}$ and $^{4CF3}PL^Z{}_{4CF3-py}$ (12/88) is calculated using the average of the integration of CH_2 peaks and CH peaks.

3.27 $^{4MeO4'CF3}$S and 2-(aminomethyl)-4-(trifluoromethyl)pyridine

Synthesis of $^{4MeO4'CF3}L_{4CF3-py}$

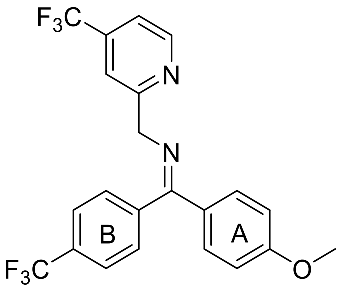

$^{4MeO4'CF3}L^{A}_{4CF3-py}$

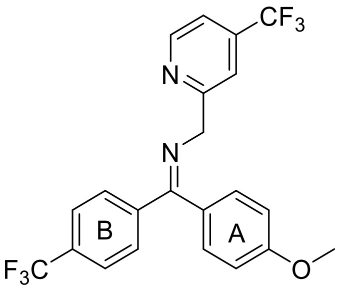

$^{4MeO4'CF3}L^{B}_{4CF3-py}$

In an oven dried flask, 2-(aminomethyl)-4-(trifluoromethyl)pyridine (2.2 equiv., 0.16 mL) was added to 4-Methoxy, 4-Trifluoromethylbenzophenone (200 mg, 0.71 mmol) and p-toluenesulfonic acid monohydrate (cat. 10 mg, 8 mol%) in toluene (30 mL). The reaction mixture was refluxed under argon with a Dean-Stark apparatus until imine formation was complete (5 days). The reaction was cooled to room temperature and diluted with diethyl ether (30 mL). The organic layer was washed with saturated ammonia chloride (20 mL x 2), saturated aqueous sodium bicarbonate (20 mL), brine (20 mL), and dried with magnesium sulfate. The final product was isolated as a brown solid (86% yield, 267 mg, 95% pure). ^{1}H-NMR (500 MHz, $CDCl_3$): δ 8.74 (d, 2H) , 8.71(d, 2H), 7.94 (d, 2H), 7.86 (d, 2H), 7.78 (d, 2H), 7.66 (d, L^A + L^B , 4H), 7.29 (m, L^A + L^B, 4H), 7.20 (m , L^E + L^Z, 4H), 7.04 (d, 2H), 6.93 (d, 2H), 4.88 (s, L^B, 2H), 4.70 (s, L^A, 2H), 3.90 (s, L^B ,3H), 3.86 (s, L^A , 3H). HRMS (ESI) m/z: [M + Na]+ Calcd for C22H16F6N2ONa 438.3734, found 439.1310.

Hydroxylation of $^{4MeO4'CF3}L_{4CF3-py}$

The reaction was carried out on 0.079 mmol scale using 36.4 mg of the imine according to the Standard Procedure. The reaction products were quantified using 0.079 mmol of 1,3,5-trimethoxybenzene (int. std.). (50% yield). The identity of the hydroxylation products was confirmed by ^{1}H-NMR.

Cleavage of $^{4MeO,4CF3}PL_{4CF3-py}$

Dissolving $^{4MeO,4CF3}PL_{4CF3-py}$ in round bottom flask with 50 mL EtOAc, then adding 100 mL 1M HCl. Reaction was going for 30 min. The resulting mixture was extracted with EtOAc (50 mL X 2). The organic phases were separated, combined, dried over $MgSO_4$, filtered, and dried under vacuum. The reaction products were dissolved in 1.4 mL of $CDCl_3$ solution containing 13.5 mg of 1,3,5-trimethoxybenzene (internal standard). The reaction products were quantified by ^{1}H-NMR using integration signals that correspond to the starting material and products with the integration signal of the internal standard.

^{1}H-NMR spectra of $^{4MeO4'CF3}L_{4CF3\text{-}py}$

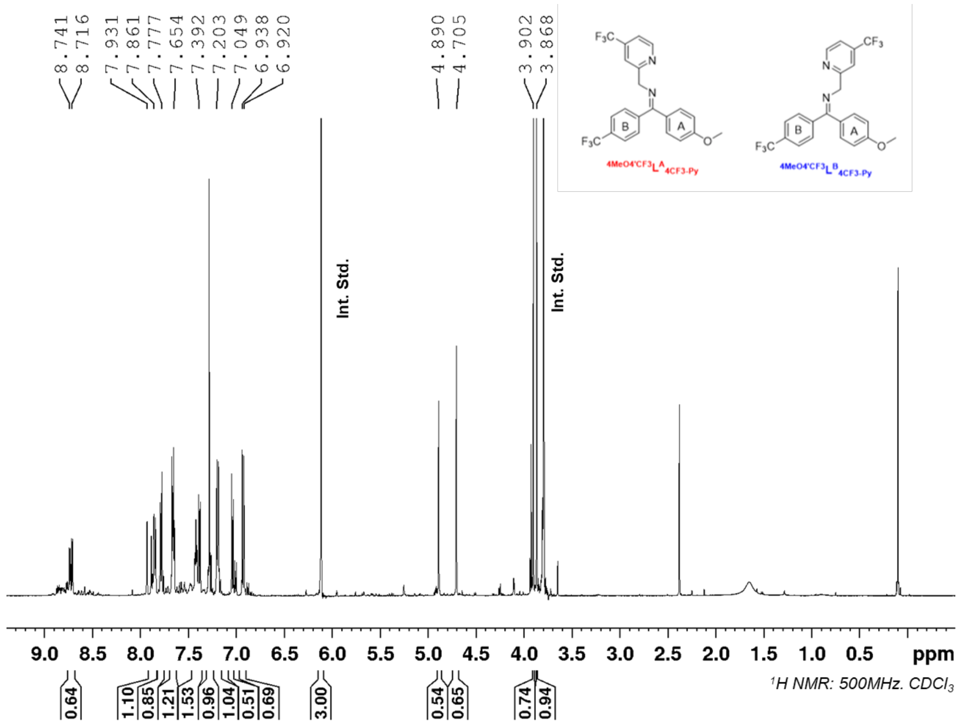

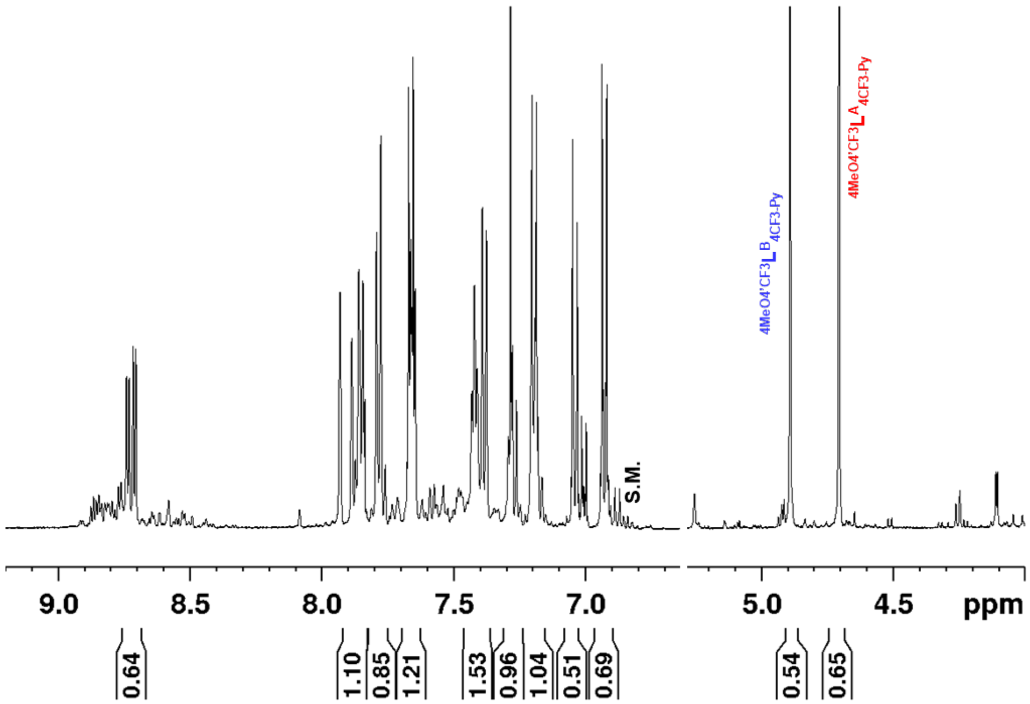

Figure S82. ^{1}H-NMR spectra for the $^{4MeO4'CF3}L_{4CF3\text{-}py}$. Note: two imine isomers were formed. The ratio of $^{4MeO4'CF3}L^{E}_{4CF3\text{-}py}$ and $^{4MeO4'CF3}L^{Z}_{4CF3\text{-}py}$ (54/46) is calculated using the average of the integration of CH_2 peaks and CH peaks.

^{1}H-NMR spectra for the hydroxylation of $^{4MeO4'CF3}L_{4CF3-py}$

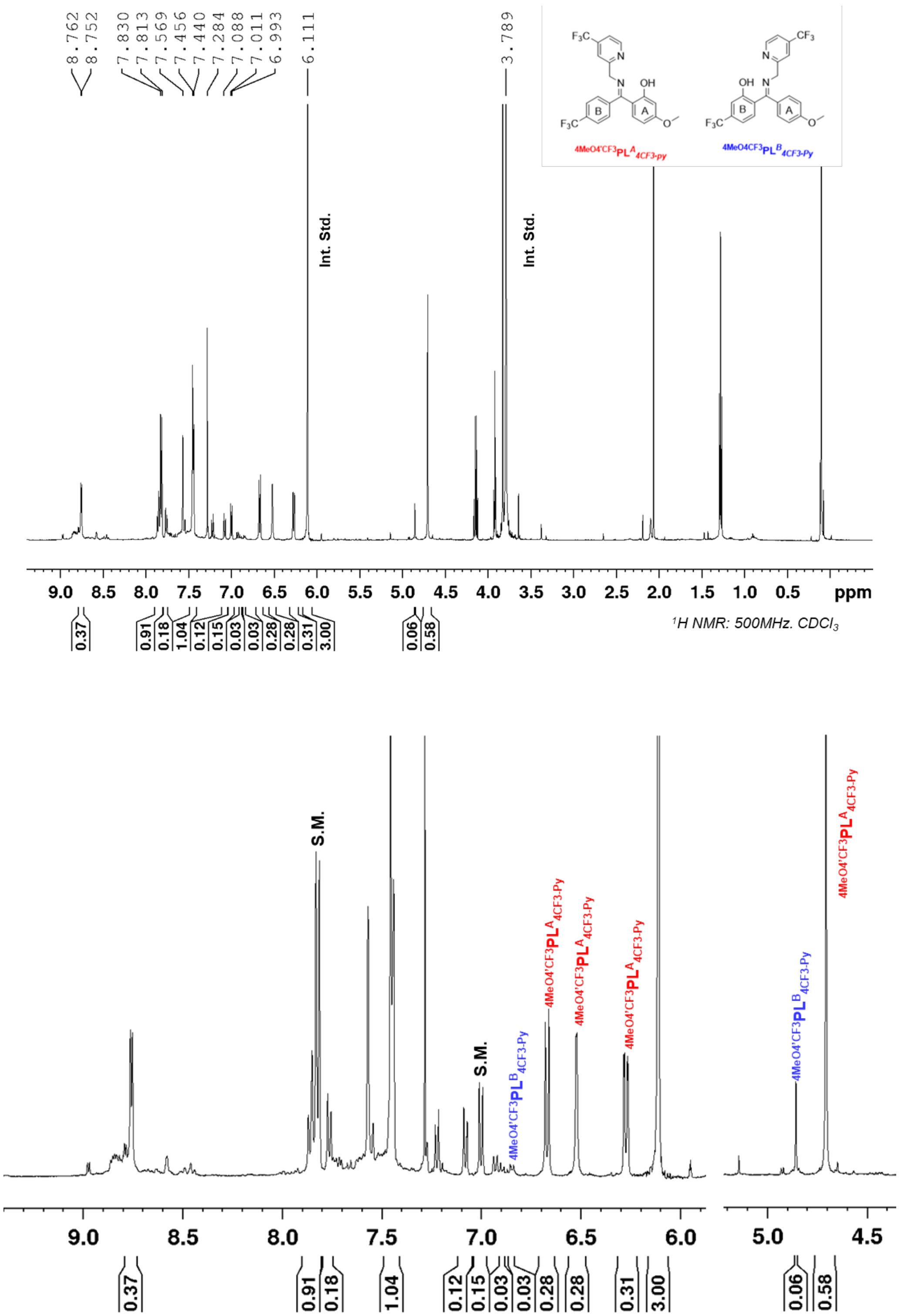

Figure S83. ^{1}H-NMR spectra for the hydroxylation of $^{4MeO4'CF3}L_{4CF3-py}$. Note: The ratio of $^{4MeO4'CF3}PL^{E}_{4CF3-py}$ and $^{4MeO4'CF3}PL^{Z}_{4CF3-py}$ (91/9) is calculated using the average of the integration of CH_2 peaks and CH peaks.

^{1}H-NMR spectra for the cleavage of $^{4MeO4'CF3}PL_{4CF3\text{-}py}$

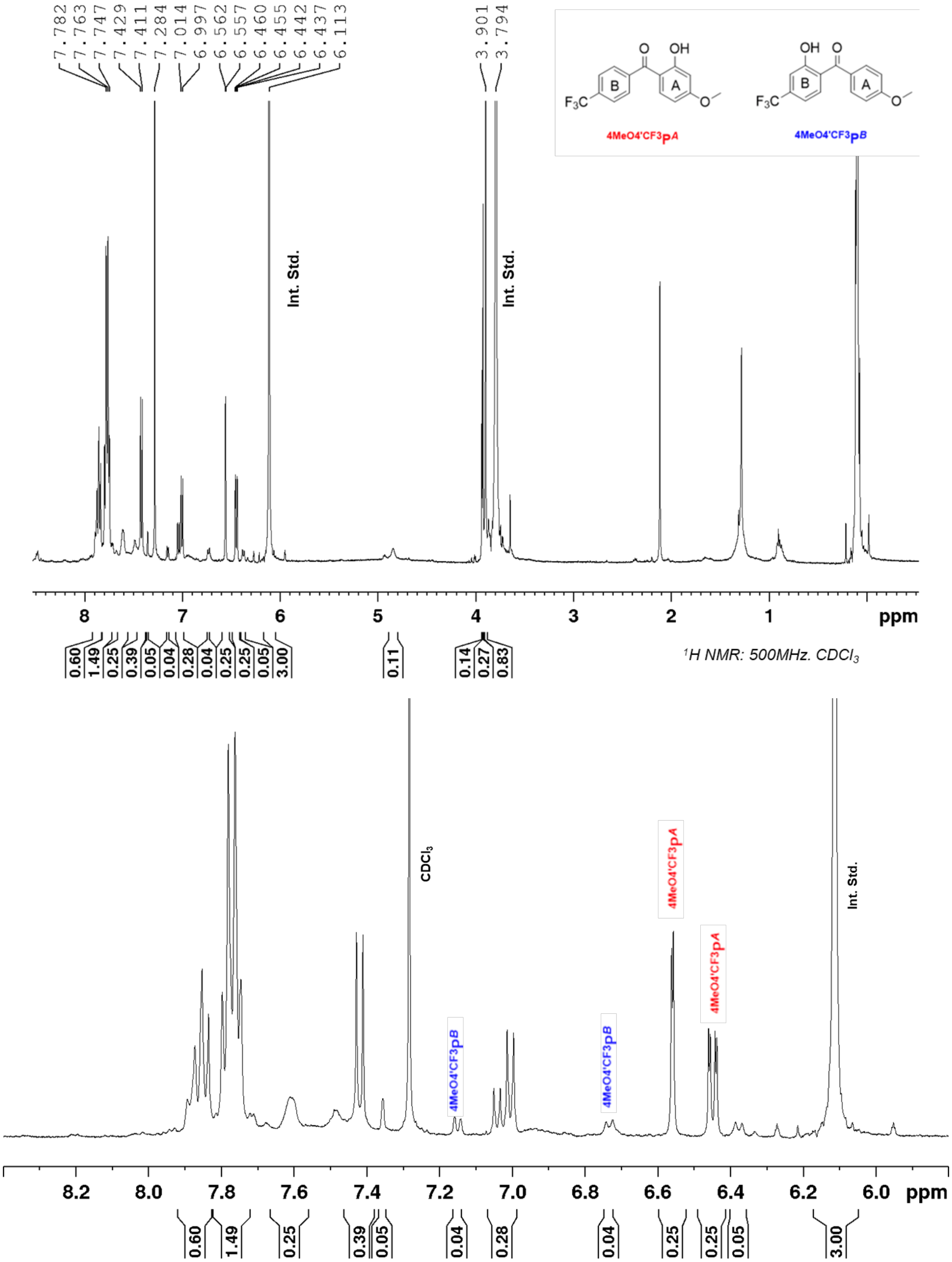

Figure S84. ^{1}H-NMR spectra for the cleavage of $^{4MeO4'CF3}PL_{4CF3\text{-}py}$. Note: The ratio of $^{4MeO4'CF3}P^{E}$ and $^{4MeO4'CF3}P^{Z}$ (86/14) is calculated using the average of the integration of CH peaks.

3.28 ^{4HMe}S and 2-picolylamine

Synthesis of ^{4HMe}L

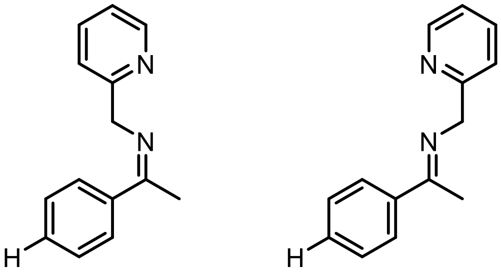

^{4HMe}L *A* ^{4HMe}L *B*

In an oven dried flask, 2-picolylamine (2.2 equiv., 2.2 mL) was added to acetophenone (1.17 g, 9.85 mmol) and p- toluenesulfonic acid monohydrate (cat. 20 mg, 1.2 mol%) in toluene (50 mL). The reaction mixture was refluxed under argon with a Dean-Stark apparatus until imine formation was complete (24 hours). The reaction was cooled to room temperature and diluted with diethyl ether (30 mL). The organic layer was washed with saturated ammonia chloride (20 mL x 2), saturated aqueous sodium bicarbonate (20 mL), brine (20 mL), and dried with magnesium sulfate. The final product was isolated as a brown solid (86% yield, 1.70 g, 90% pure). ^{1}H-NMR (500 MHz, $CDCl_3$): δ 8.58 (d, 2H), 7.92 (d, 4H), 7.70 (d, 4H), 7.43 (m, 6H), 7.19 (t, 2H), 4.88 (s, 2H), 4.61 (s, 2H), 2.64 (s, 3H), 2.41 (s, 3H).). HRMS (ESI) m/z: [M + Na]+ Calcd for C14H14N2Na 210.2800, found 211.1304. Note: this imine substrate-ligand was previously synthesized by our group.

Hydroxylation of ^{4HMe}L

The reaction was carried out on 0.159 mmol scale using 37.0 mg of the imine according to the Standard Procedure. The reaction products were quantified using 0.159 mmol of 1,3,5-trimethoxybenzene (int. std.). (60% yield). The identity of the hydroxylation products was confirmed by ^{1}H-NMR.

^{1}H-NMR spectra of ^{4HMe}L

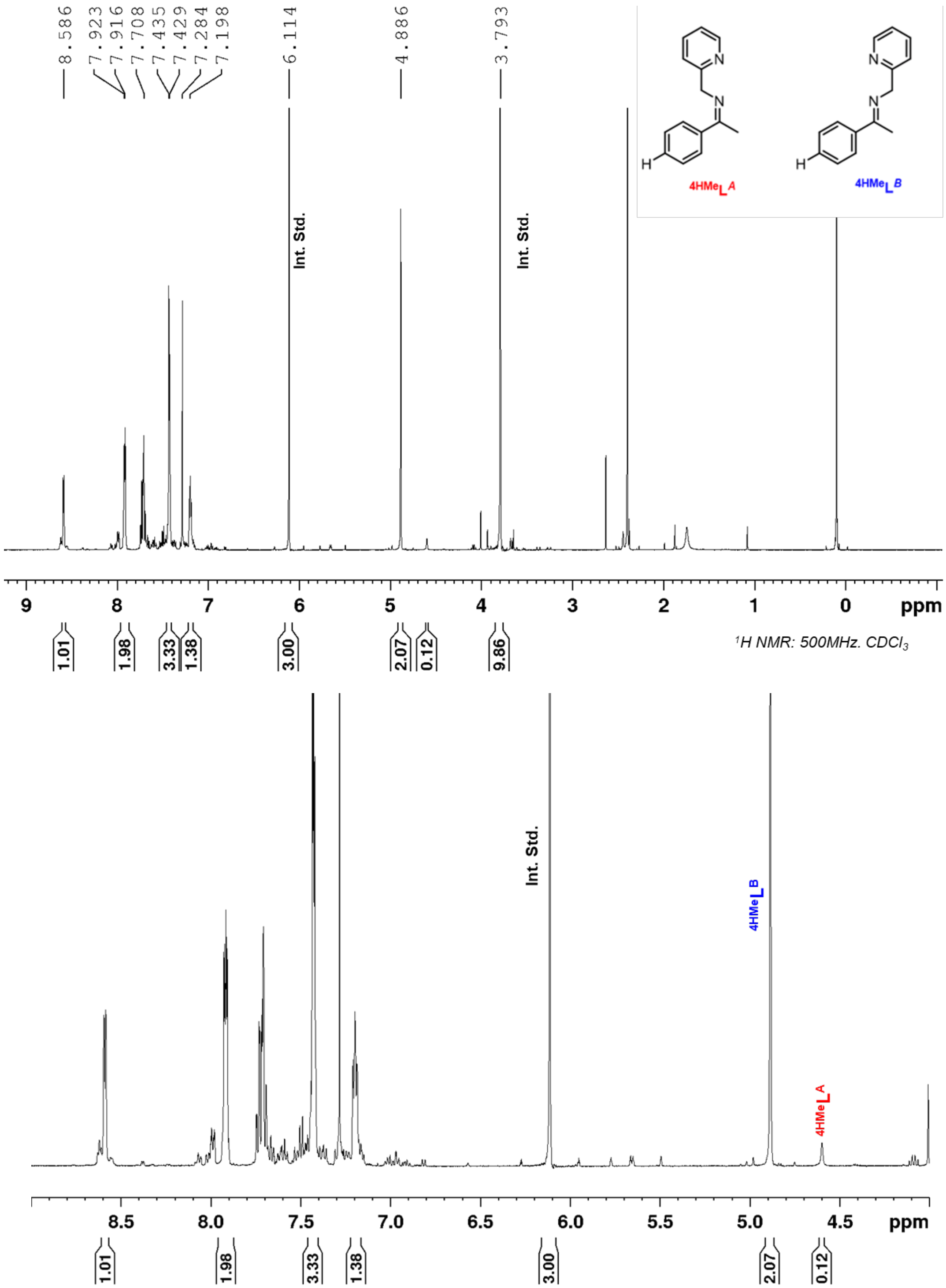

Figure S85. ^{1}H-NMR spectra of ^{4HMe}L. Note: two imine isomers were formed. The ratio of $^{4HMe}L^{A}$ and $^{4Hme}L^{B}$ (5/95) is calculated using the average of the integration of CH_2 peaks and CH peaks.

^{1}H-NMR spectra for the hydroxylation of ^{4HMe}L

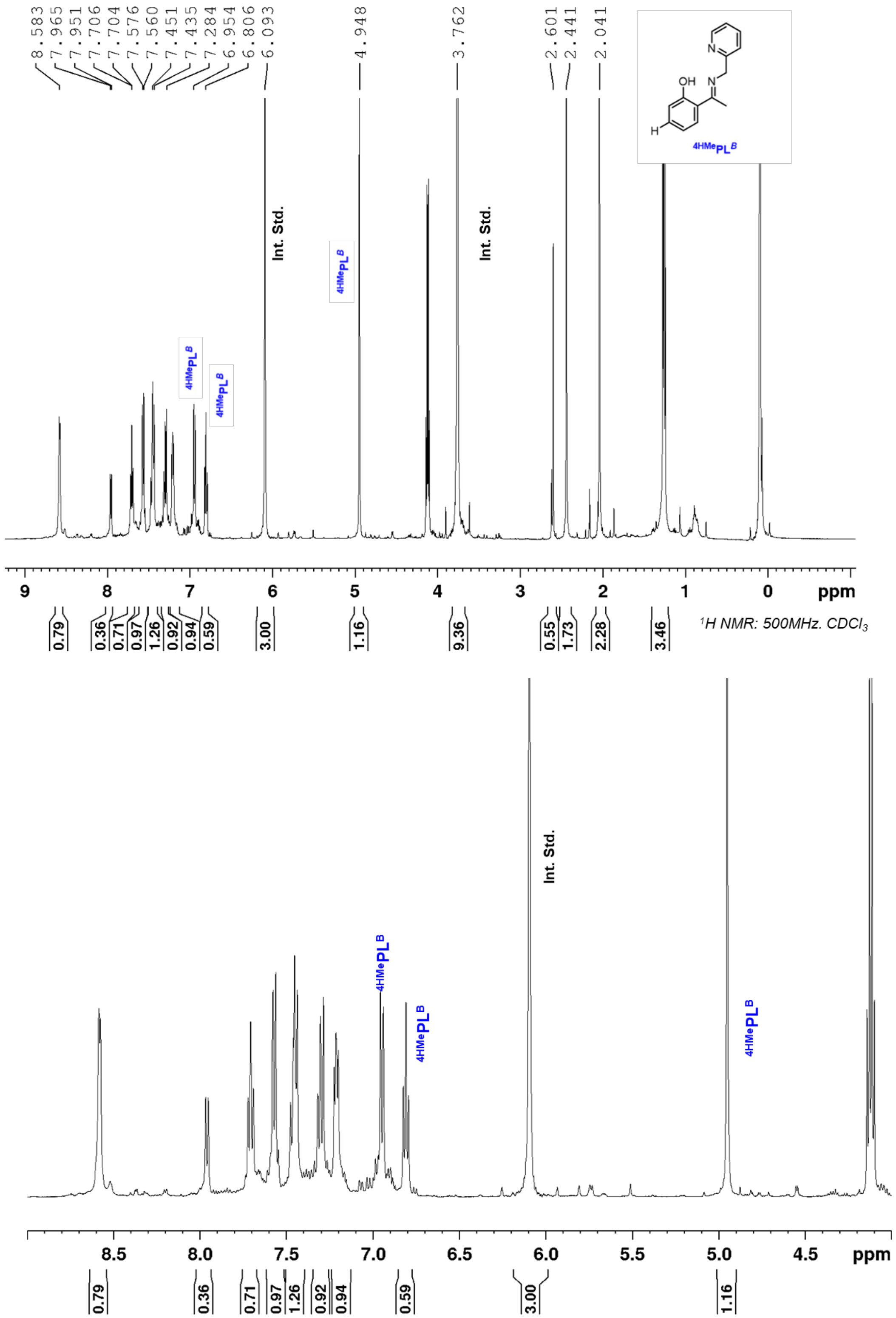

Figure S86. ^{1}H-NMR spectra for the hydroxylation of ^{4HMe}L. Note: The ratio of ^{4HMe}P^A and ^{4HMe}P^B (0/100) is calculated using the average of the integration of CH_2 peaks and CH peaks.

3.29 ^{4HEt}S and 2-picolylamine

Synthesis of ^{4HEt}L

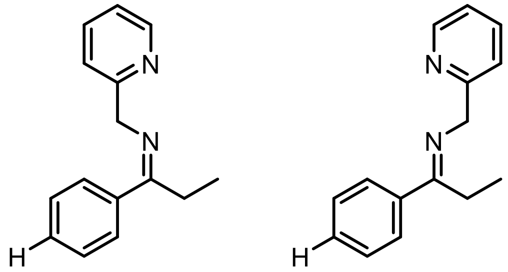

^{4HEt}L *A* ^{4HEt}L *B*

In an oven dried flask, 2-picolylamine (2.2 equiv., 2.2 mL) was added to propiophenone (1.3 g, 9.85 mmol) and p- toluenesulfonic acid monohydrate (cat. 20 mg, 1.2 mol%) in toluene (50 mL). The reaction mixture was refluxed under argon with a Dean-Stark apparatus until imine formation was complete (12 hours). The reaction was cooled to room temperature and diluted with diethyl ether (30 mL). The organic layer was washed with saturated ammonia chloride (20 mL x 2), saturated aqueous sodium bicarbonate (20 mL), brine (20 mL), and dried with magnesium sulfate. The final product was isolated as a brown solid (94% yield, 2.07 g, 95% pure). ^{1}H-NMR (500 MHz, $CDCl_3$): δ 8.56 (d, 2H), 7.92 (d, 2H), 7.75 (d, 4H), 7.37 (m, 6H), 7.25 (m, 4H), 4.95 (s, 2H), 4.56 (s, 2H), 2.88 (t, 2H), 2.72 (t, 2H), 1.20(s, 6H). 13C{1H} NMR (500 MHz, CD_3CN): δ 171.5, 160.8, 149.1, 149.0, 136.6, 130.0, 129.0, 128.3, 127.0, 126.3, 122.0, 121.1, 58.7, 56.9, 35.1, 22.4. HRMS (ESI) m/z: [M + Na]+ Calcd for C15H16N2Na 224.3070, found 225.3674.

Hydroxylation of ^{4HEt}L

The reaction was carried out on 0.159 mmol scale using 38.0 mg of the imine according to the Standard Procedure. The reaction products were quantified using 0.159 mmol of 1,3,5-trimethoxybenzene (int. std.). (43% yield). The identity of the hydroxylation products was confirmed by ^{1}H-NMR.

^{1}H-NMR spectra of ^{4HEt}L

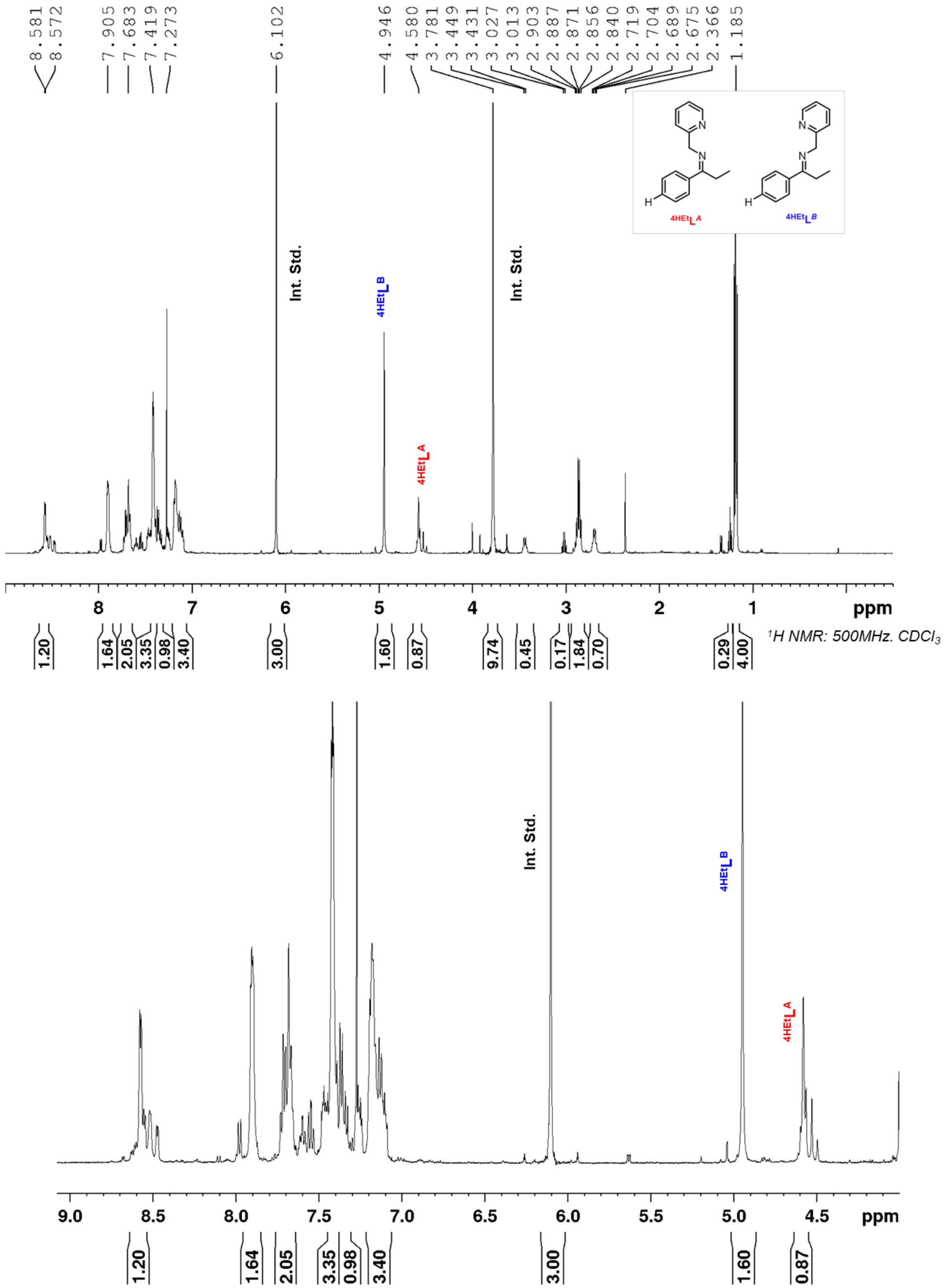

Figure S87. ^{1}H-NMR spectra of ^{4HEt}L. Note: two imine isomers were formed. The ratio of ^{4HEt}**L**^{A} and ^{4HEt}**L**^{B} (32/68) is calculated using the average of the integration of CH_2 peaks and CH peaks.

^{1}H-NMR spectra for the hydroxylation of ^{4HEt}L

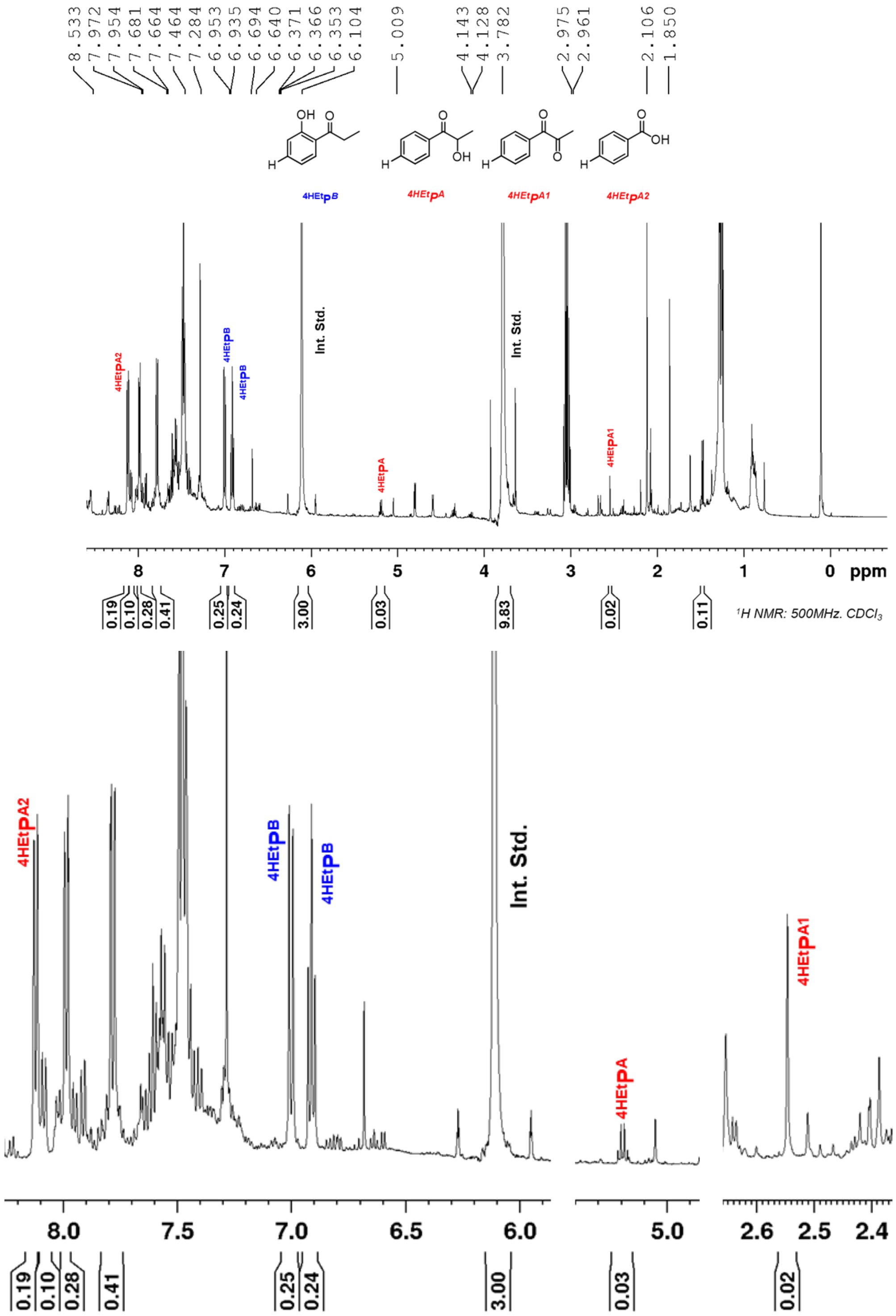

Figure S88. ^{1}H-NMR spectra for the hydroxylation of ^{4HEt}L. Note: The ratio of $^{4HEt}P^A$ and $^{4HEt}P^B$ (37/63) is calculated using the average of the integration of CH_2 peaks and CH peaks.

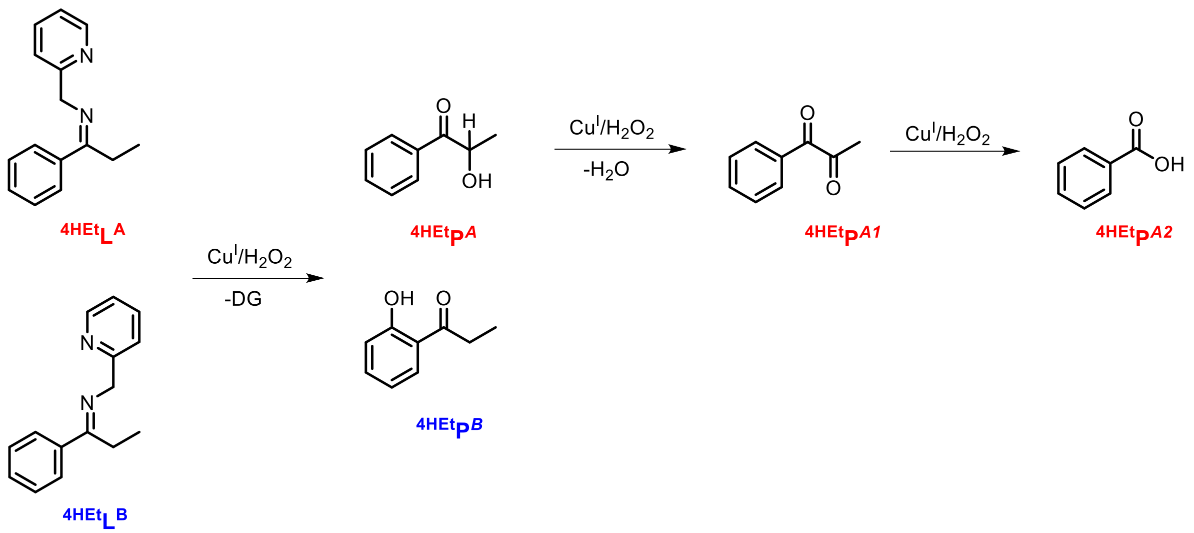

Scheme 1. Oxidation of *$^{4HEt}L^{A}$ and $^{4HEt}L^{B}$ to all oxidation products*

3.30 ^{4MeOEt}S and 2-picolylamine

Synthesis of ^{4MeOEt}L

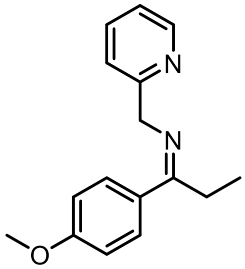

^{4MeOEt}L *A*

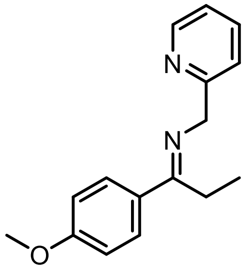

^{4MeOEt}L *B*

In an oven dried flask, 2-picolylamine (2.2 equiv., 2.2 mL) was added to 4-methoxypropiophenone (1.63 g, 9.85 mmol) and p- toluenesulfonic acid monohydrate (cat. 20 mg, 1.2 mol%) in toluene (50 mL). The reaction mixture was refluxed under argon with a Dean-Stark apparatus until imine formation was complete (12 hours). The reaction was cooled to room temperature and diluted with diethyl ether (30 mL). The organic layer was washed with saturated ammonia chloride (20 mL x 2), saturated aqueous sodium bicarbonate (20 mL), brine (20 mL), and dried with magnesium sulfate. The final product was isolated as a brown solid (85% yield, 2.12 g, 90% pure). ^{1}H-NMR (500 MHz, $CDCl_3$): δ 8.58 (d, 1H), 8.57 (d, 1H), 7.90 (d, 2H), 7.73 (t, 2H), 7.67 (t, 2H), 7.19 (t, 2H), 7.13 (t, 2H), 6.94 (m, 4H), 4.92 (s, 2H), 4.63 (s, 2H), 3.83 (s, 6H), 2.84 (q, 2H), 2.70 (q, 2H), 1.20 (s, 6H). 13C{1H} NMR (500 MHz, CD_3CN): δ 170.6, 161.1, 149.1, 136.6, 136.5, 132.1, 128.5, 127.9, 121.9, 121.6, 113.6, 56.7, 55.3, 35.2, 22.1, 11.5. HRMS (ESI) m/z: [M + Na]+ Calcd for C16H18N2ONa 254.3330, found 255.1486.

Hydroxylation of ^{4MeOEt}L

The reaction was carried out on 0.159 mmol scale using 45.0 mg of the imine according to the Standard Procedure. The reaction products were quantified using 0.159 mmol of 1,3,5-trimethoxybenzene (int. std.). (56% yield). The identity of the hydroxylation products was confirmed by ^{1}H-NMR.

¹H-NMR spectra of ^4MeOEt^L

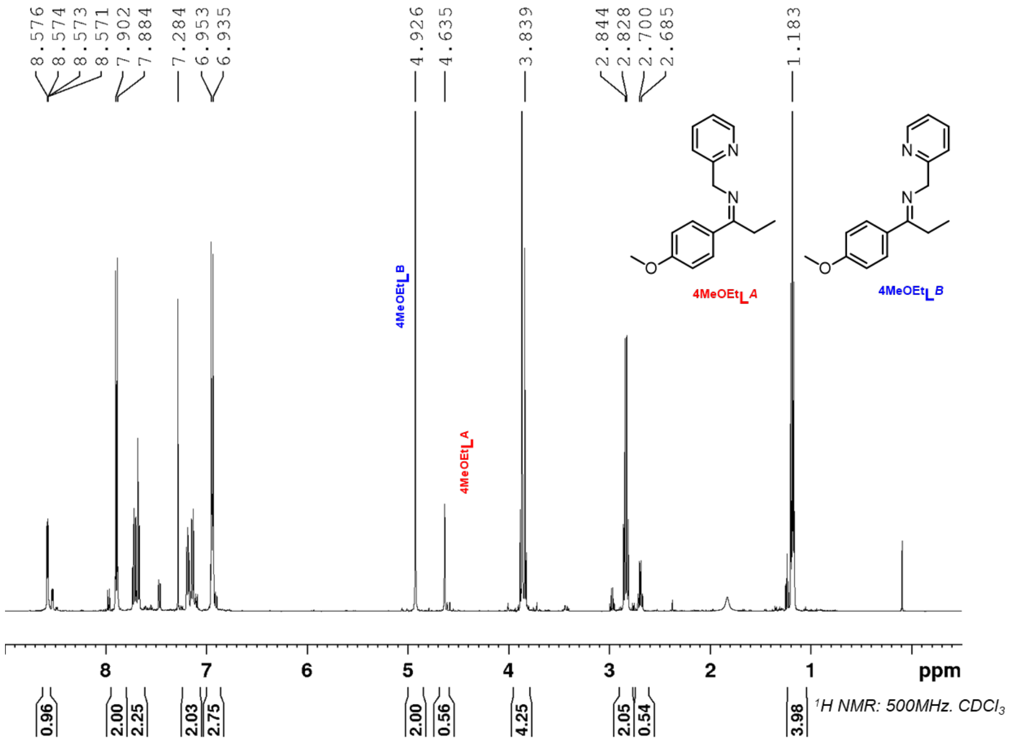

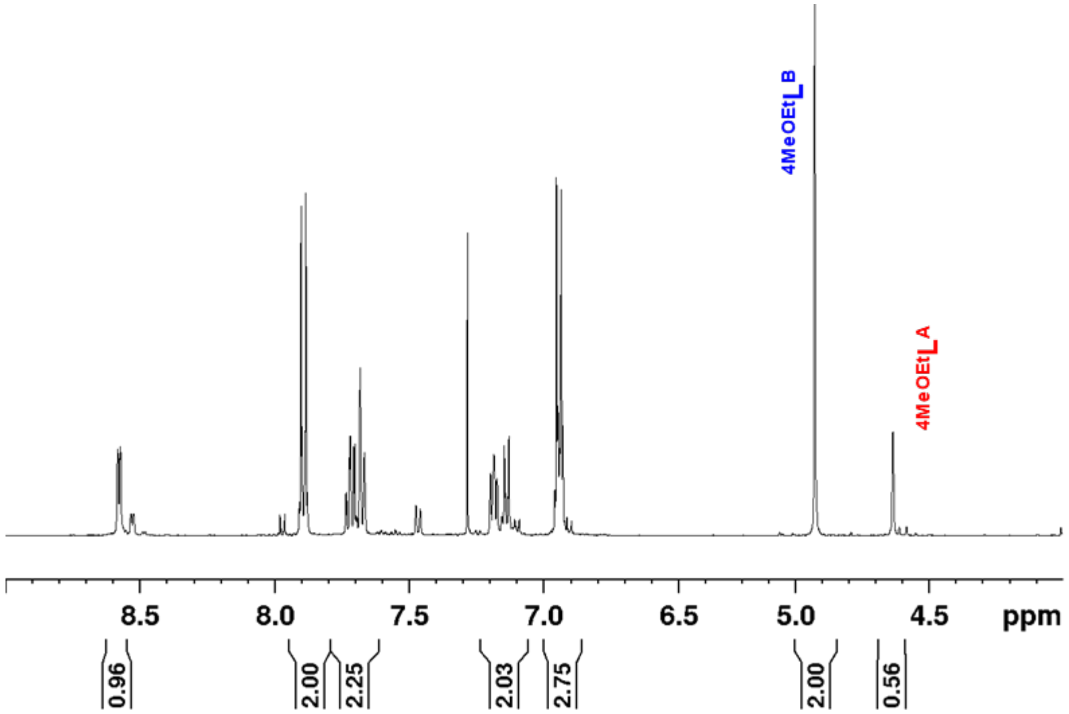

Figure S89. ^{1}H-NMR spectra of ^{4MeOEt}L. Note: two imine isomers were formed. The ratio of $^{4MeOEt}L^{A}$ and $^{4MeOEt}L^{B}$ (20/80) is calculated using the average of the integration of CH_2 peaks and CH peaks.

1H-NMR spectra for the hydroxylation of ^{4MeOEt}L

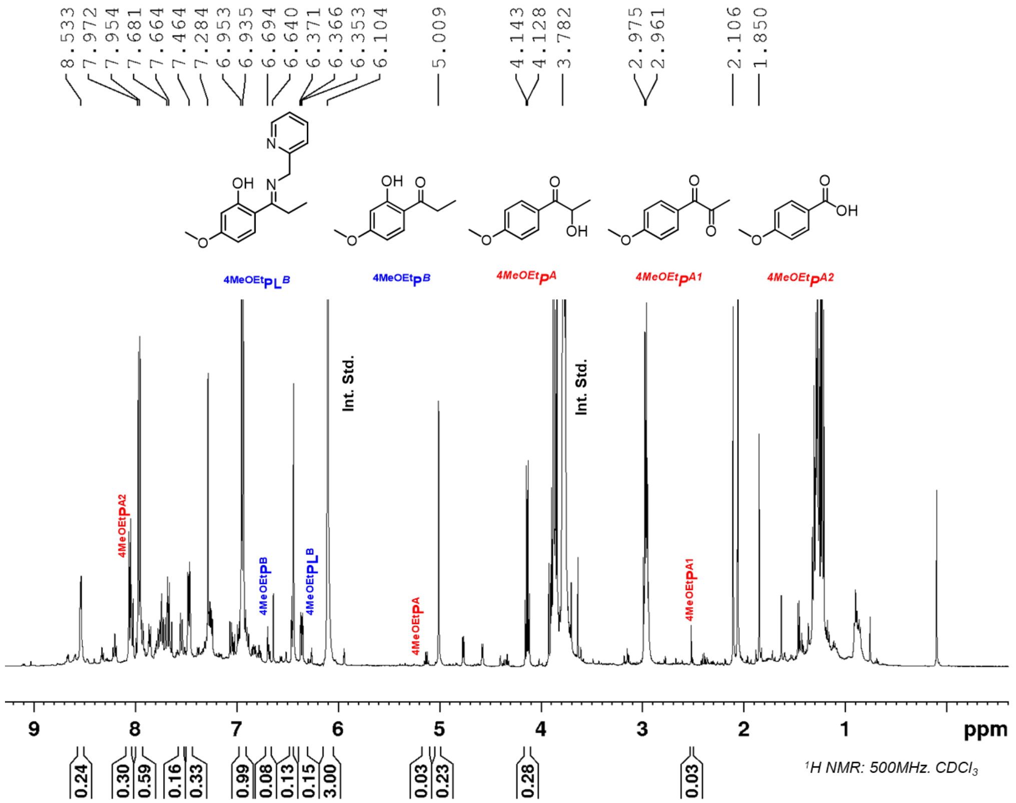

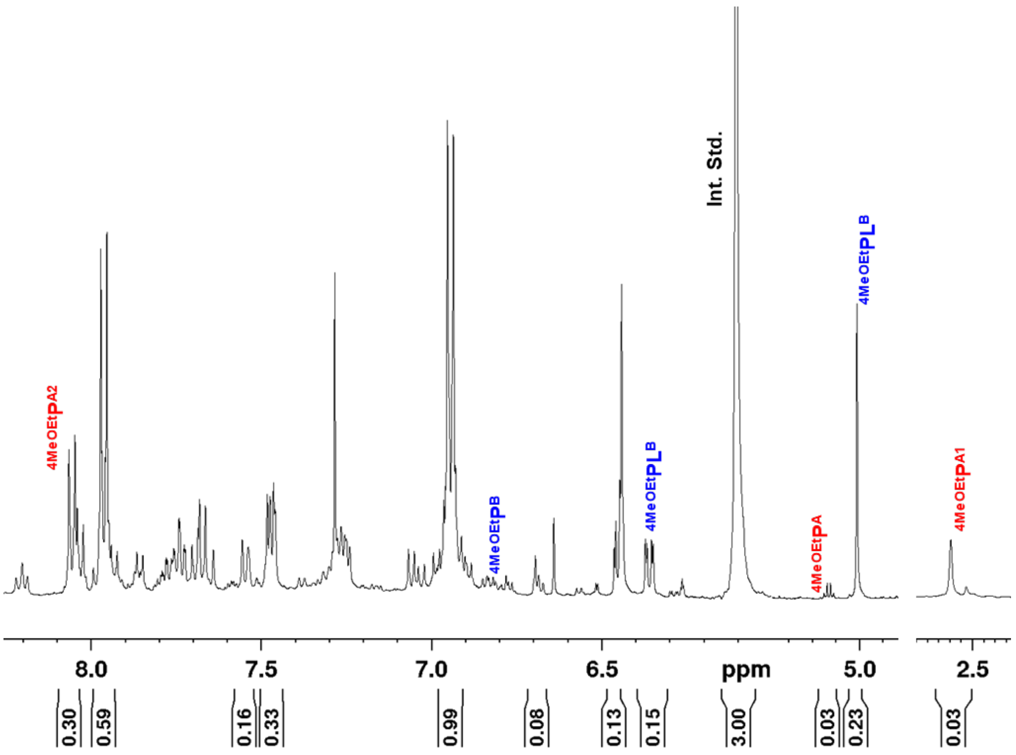

Figure S90. ^{1}H-NMR spectra for the hydroxylation of ^{4MeOEt}L. Note: The ratio of ^{4MeOEt}P^{A} and ^{4MeOEt}P^{B} (30/70) is calculated using the average of the integration of CH_2 peaks and CH peaks.

3.31 ^{4FEt}S and 2-picolylamine

Synthesis of ^{4FEt}L

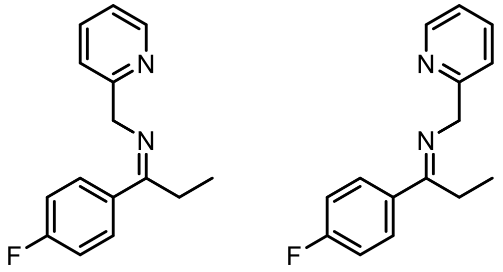

4FEtL *A*   4FEtL *B*

In an oven dried flask, 2-picolylamine (2.2 equiv., 2.2 mL) was added to 4-Fluoropropiophenone (1.39 g, 9.85 mmol) and p- toluenesulfonic acid monohydrate (cat. 20 mg, 1.2 mol%) in toluene (50 mL). The reaction mixture was refluxed under argon with a Dean-Stark apparatus until imine formation was complete (12 hours). The reaction was cooled to room temperature and diluted with diethyl ether (30 mL). The organic layer was washed with saturated ammonia chloride (20 mL x 2), saturated aqueous sodium bicarbonate (20 mL), brine (20 mL), and dried with magnesium sulfate. The final product was isolated as a brown solid (87% yield, 2.07 g, 92% pure). ^{1}H-NMR (500 MHz, $CDCl_3$): δ 8.59 (d, 1H), 8.54 (d, 1H), 7.91 (d, 2H), 7.74 (t, 4H), 7.22- 7.05 (m, 8H), 4.92 (s, 2H), 4.57 (s, 2H). 2.85 (q, 2H), 2.68 (q, 2H), 1.19 (t, 6H). 13C{1H} NMR (500 MHz, CD_3CN): δ 170.2, 164.9, 149.1, 136.7, 128.9, 121.8, 115.6, 58.7, 35.3, 22.4, 11.4. HRMS (ESI) m/z: [M + Na]+ Calcd for C15H15FN2Na calculated 242.2971, found 243.0914.

***Hydroxylation of ^{4FEt}*L**

The reaction was carried out on 0.159 mmol scale using 42.0 mg of the imine according to the Standard Procedure. The reaction products were quantified using 0.159 mmol of 1,3,5-trimethoxybenzene (int. std.). (30% yield). The identity of the hydroxylation products was confirmed by ^{1}H-NMR.

^{1}H-NMR spectra of ^{4FEt}L

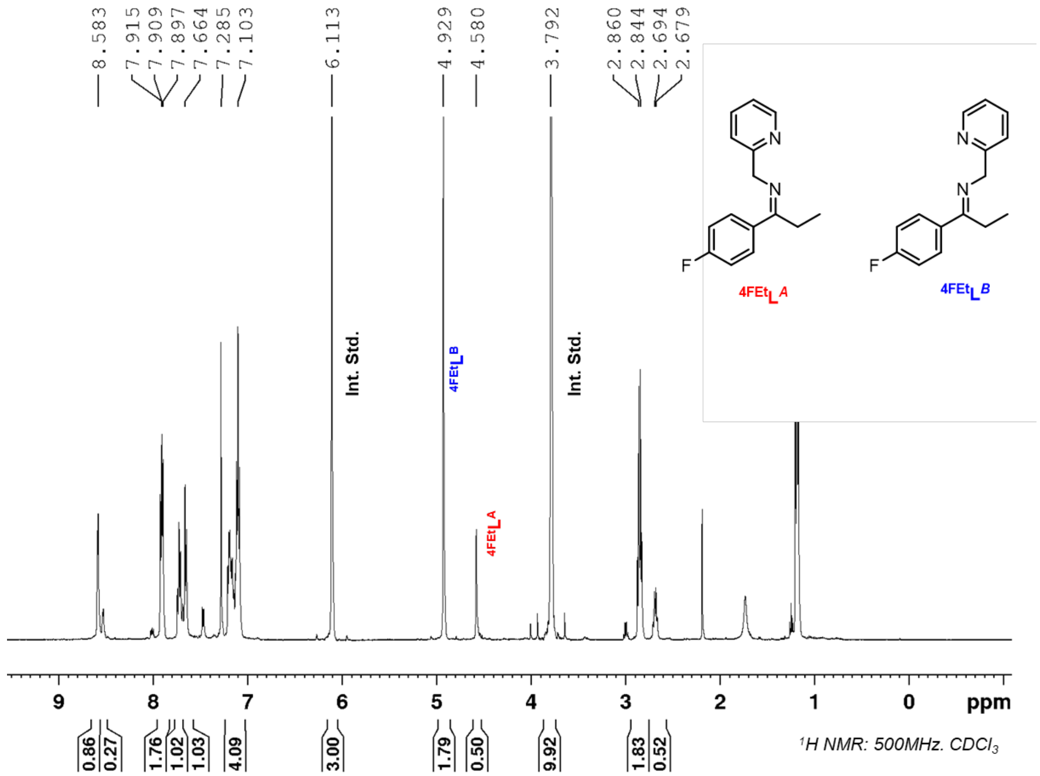

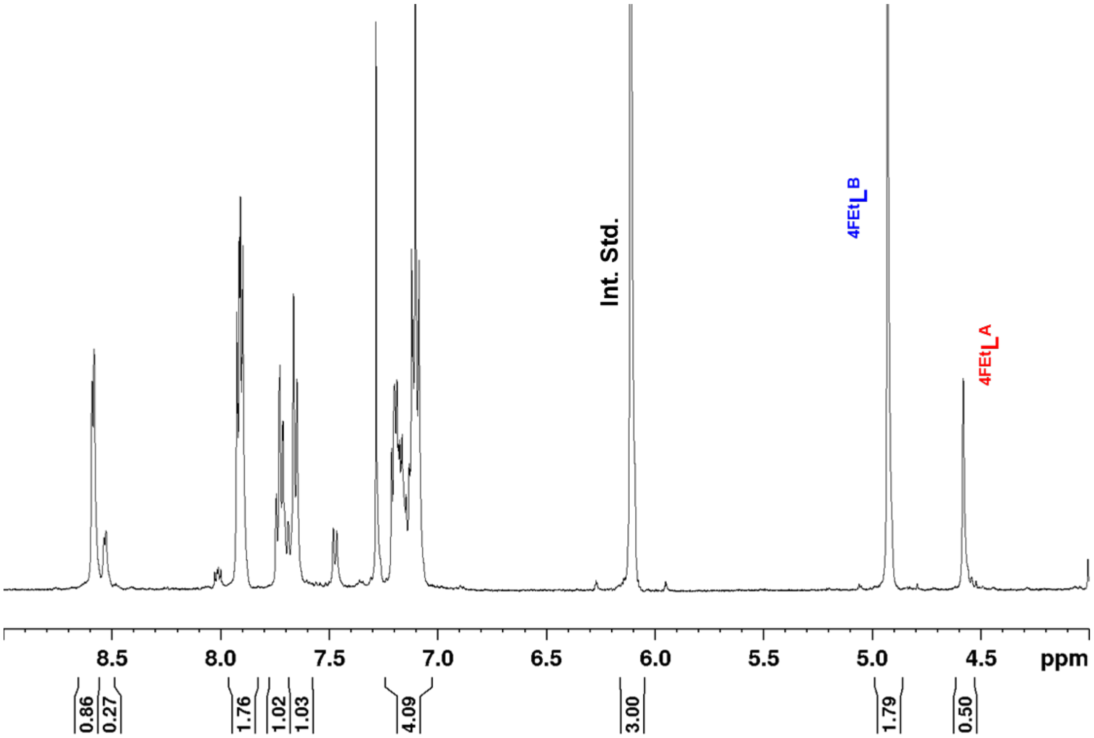

Figure S91. ^{1}H-NMR spectra of ^{4FEt}L. Note: two imine isomers were formed. The ratio of $^{4FEt}L^{A}$ and $^{4FEt}L^{B}$ (20/80) is calculated using the average of the integration of CH_2 peaks and CH peaks.

^{1}H-NMR spectra for the hydroxylation of ^{4FEt}L

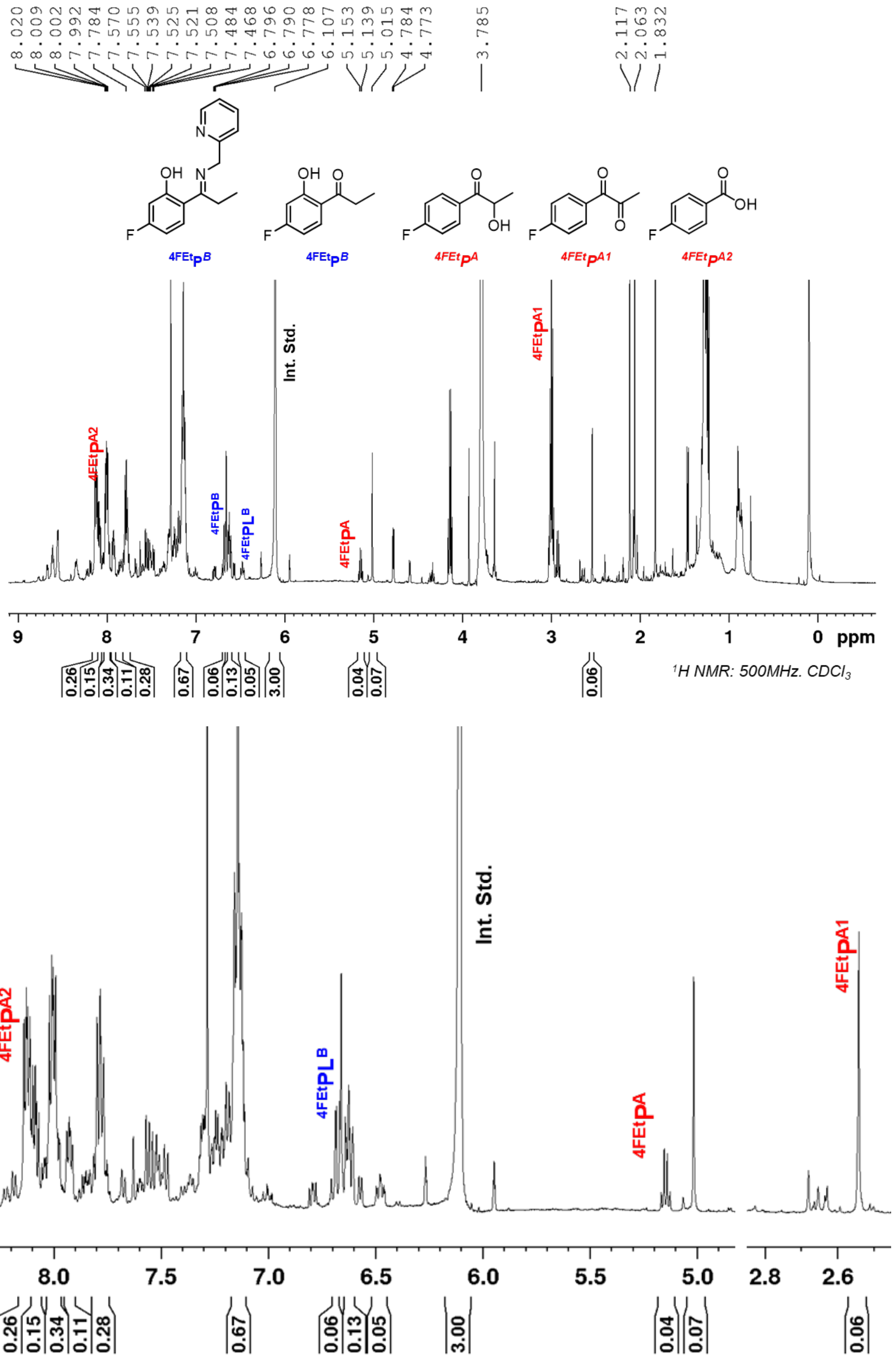

Figure S92. ^{1}H-NMR spectra for the hydroxylation of ^{4FEt}L. Note: The ratio of $^{4FEt}P^{A}$ and $^{4FEt}P^{B}$ (47/53) is calculated using the average of the integration of CH_2 peaks and CH peaks.

3.32 ^{4HEt}S and 2-(aminomethyl)-4-methoxypyridine

Synthesis of $^{4HEt}L_{4MeO\text{-}Py}$

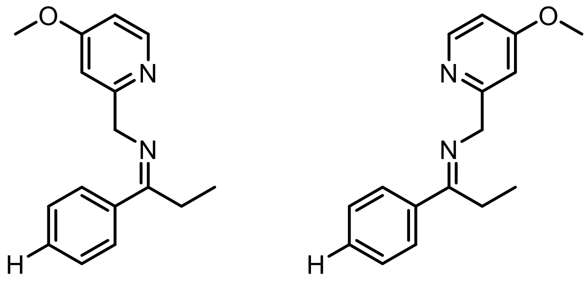

$^{4HEt}L^{A}_{4MeO\text{-}Py}$ $^{4HEt}L^{B}_{4MeO\text{-}Py}$

In an oven dried flask, 2-(aminomethyl)-4-methoxypyridine (2.2 equiv., 0.67 mL) was added to propiophenone (400 mg, 2.98 mmol) and p- toluenesulfonic acid monohydrate (cat. 10 mg, 1.9 mol%) in toluene (50 mL). The reaction mixture was refluxed under argon with a Dean-Stark apparatus until imine formation was complete (12 hours). The reaction was cooled to room temperature and diluted with diethyl ether (30 mL). The organic layer was washed with saturated ammonia chloride (20 mL x 2), saturated aqueous sodium bicarbonate (20 mL), brine (20 mL), and dried with magnesium sulfate. The final product was isolated as a brown solid (89% yield, 673 mg, 85% pure). ^{1}H-NMR (500 MHz, $CDCl_3$): δ 8.41 (d, 1H), 8.34 (d, 1H), 7.92 (d, 2H), 7.44 (m, 4H), 7.27 (m, 4H), 7.18 (m, 2H), 6.74 (d, 2H), 4.91 (s, 2H), 4.55 (s, 2H), 3.90 (s,6H), 2.87 (q, 2H), 2.72(q, 2H), 1.27 (t, 3H), 1.18 (t, 3H). 13C{1H} NMR (500 MHz, CD_3CN): δ 200.8, 175.1, 171.5, 166.5, 162.7, 150.2, 139.6, 136.9, 128.6, 128.3, 127.9, 126.9, 108.1, 58.5, 55.1, 35.15, 31.8, 29.7, 22.5, 11.4, 10.9, 8.2. HRMS (ESI) m/z: [M + Na]+ Calcd for C16H18N2ONa 254.3330, found 255.1287.

Hydroxylation of $^{4HEt}L_{4MeO\text{-}Py}$

The reaction was carried out on 0.159 mmol scale using 48.0 mg of the imine according to the Standard Procedure. The reaction products were quantified using 0.159 mmol of 1,3,5-trimethoxybenzene (int. std.). (45% yield). The identity of the hydroxylation products was confirmed by ^{1}H-NMR.

^{1}H-NMR spectra of $^{4HEt}L_{4MeO-Py}$

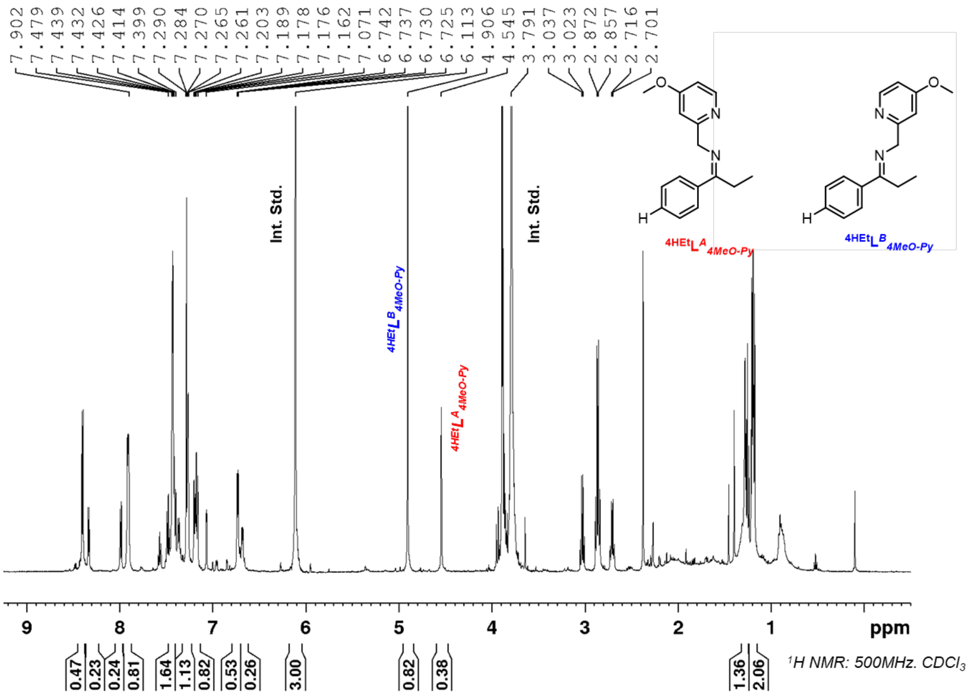

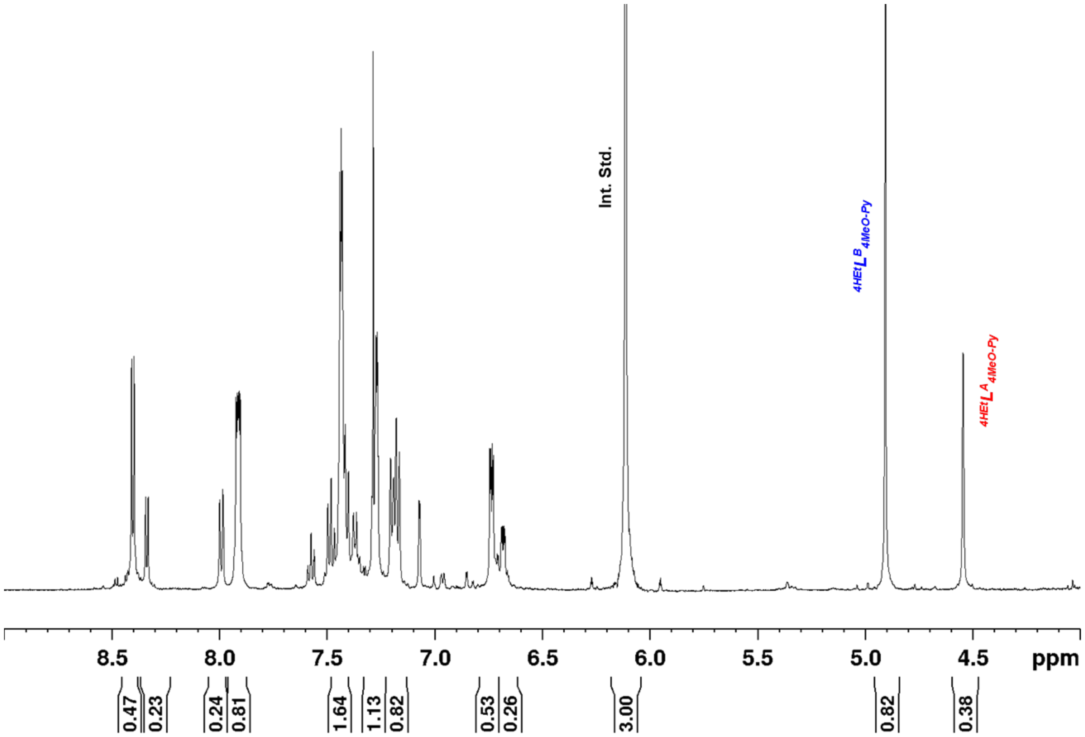

Figure S93. ^{1}H-NMR spectra of $^{4HEt}L_{4MeO-Py}$. Note: two imine isomers were formed. The ratio of $^{4HEt}L_{4MeO-Py}$ and $^{4HEt}L_{4MeO-Py}$ (32/68) is calculated using the average of the integration of CH_2 peaks and CH peaks.

^{1}H-NMR spectra for the Hydroxylation of $^{4HEt}L_{4MeO-Py}$

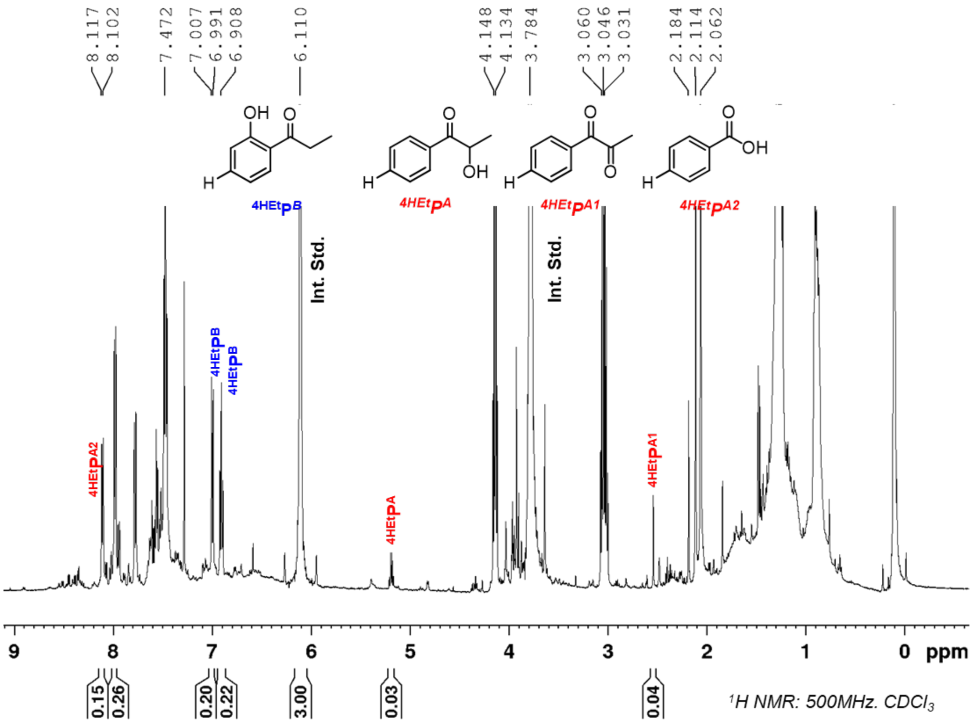

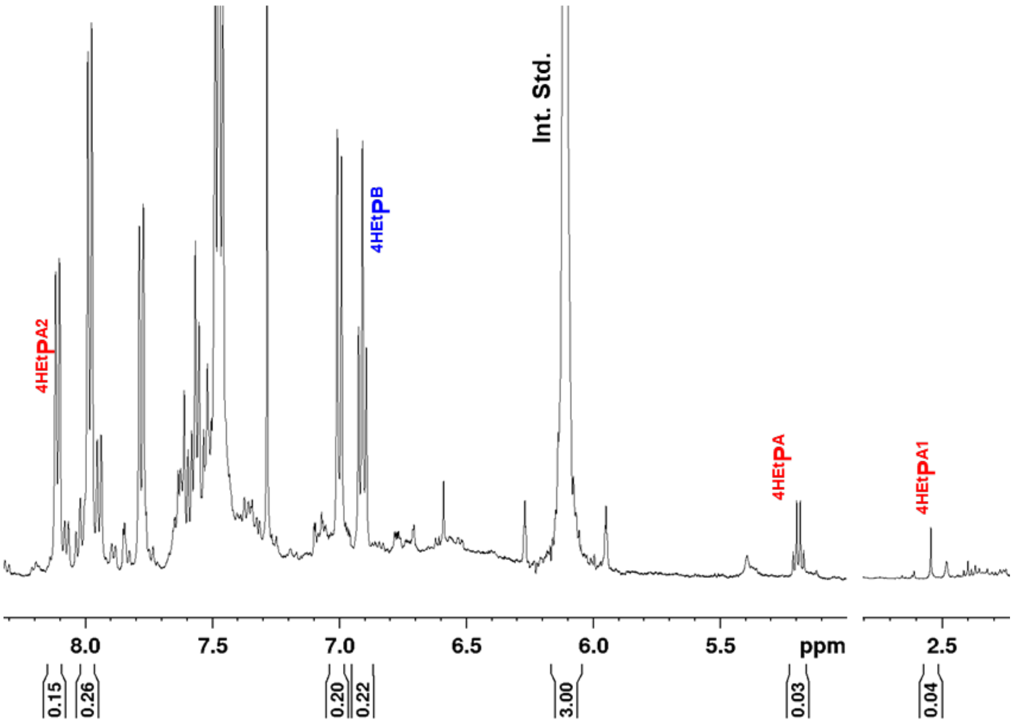

Figure S94. ^{1}H-NMR spectra for the hydroxylation of $^{4HEt}L_{4MeO\text{-}Py}$. Note: The ratio of $^{4HEt}P^{A}_{4MeO\text{-}Py}$ and $^{4HEt}P^{B}_{4MeO\text{-}Py}$ (29/71) is calculated using the average of the integration of CH_2 peaks and CH peaks.

3.33 ^{4HEt}S and 2-(aminomethyl)-4-chloropyridine

Synthesis of ^{4HEt}L$_{4Cl-Py}$

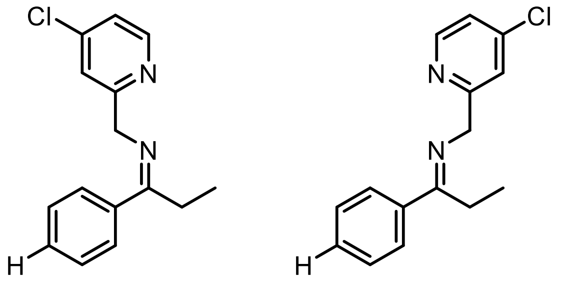

$^{4HEt}L^{A}_{4Cl-Py}$ $^{4HEt}L^{B}_{4Cl-Py}$

In an oven dried flask, 2-(aminomethyl)-4-chloropyridine (2.2 equiv., 0.50 mL) was added to propiophenone (300 mg, 2.23 mmol) and p- toluenesulfonic acid monohydrate (cat. 10 mg, 2.5 mol%) in toluene (50 mL). The reaction mixture was refluxed under argon with a Dean-Stark apparatus until imine formation was complete (12 hours). The reaction was cooled to room temperature and diluted with diethyl ether (30 mL). The organic layer was washed with saturated ammonia chloride (20 mL x 2), saturated aqueous sodium bicarbonate (20 mL), brine (20 mL), and dried with magnesium sulfate. The final product was isolated as a brown solid (96% yield, 552 mg, 93% pure). ^{1}H-NMR (500 MHz, $CDCl_3$): δ 8.48 (d, 1H), 8.40 (d, 1H), 7.92 (d, 2H), 7.76 (s, 2H), 7.45 (m, 6H), 7.28 (d, 2H), 7.17 (d, 2H), 4.72-4.55 (s, 2H). 13C{1H} NMR (500 MHz, CD_3CN): δ 171.8, 162.7, 149.7, 129.8, 128.3, 126.1, 122.1, 58.1, 35.1, 22.5, 11.3, 10.7, 8.2. HRMS (ESI) m/z: [M + Na]+ Calcd for C15H15ClN2Na 258.7490, found 258.1036.

Hydroxylation of ^{4HEt}L$_{4Cl-Py}$

The reaction was carried out on 0.159 mmol scale using 44.0 mg of the imine according to the Standard Procedure. The reaction products were quantified using 0.159 mmol of 1,3,5-trimethoxybenzene (int. std.). (36% yield). The identity of the hydroxylation products was confirmed by ^{1}H-NMR.

^{1}H-NMR spectra of ^{4HEt}L$_{4Cl\text{-}Py}$

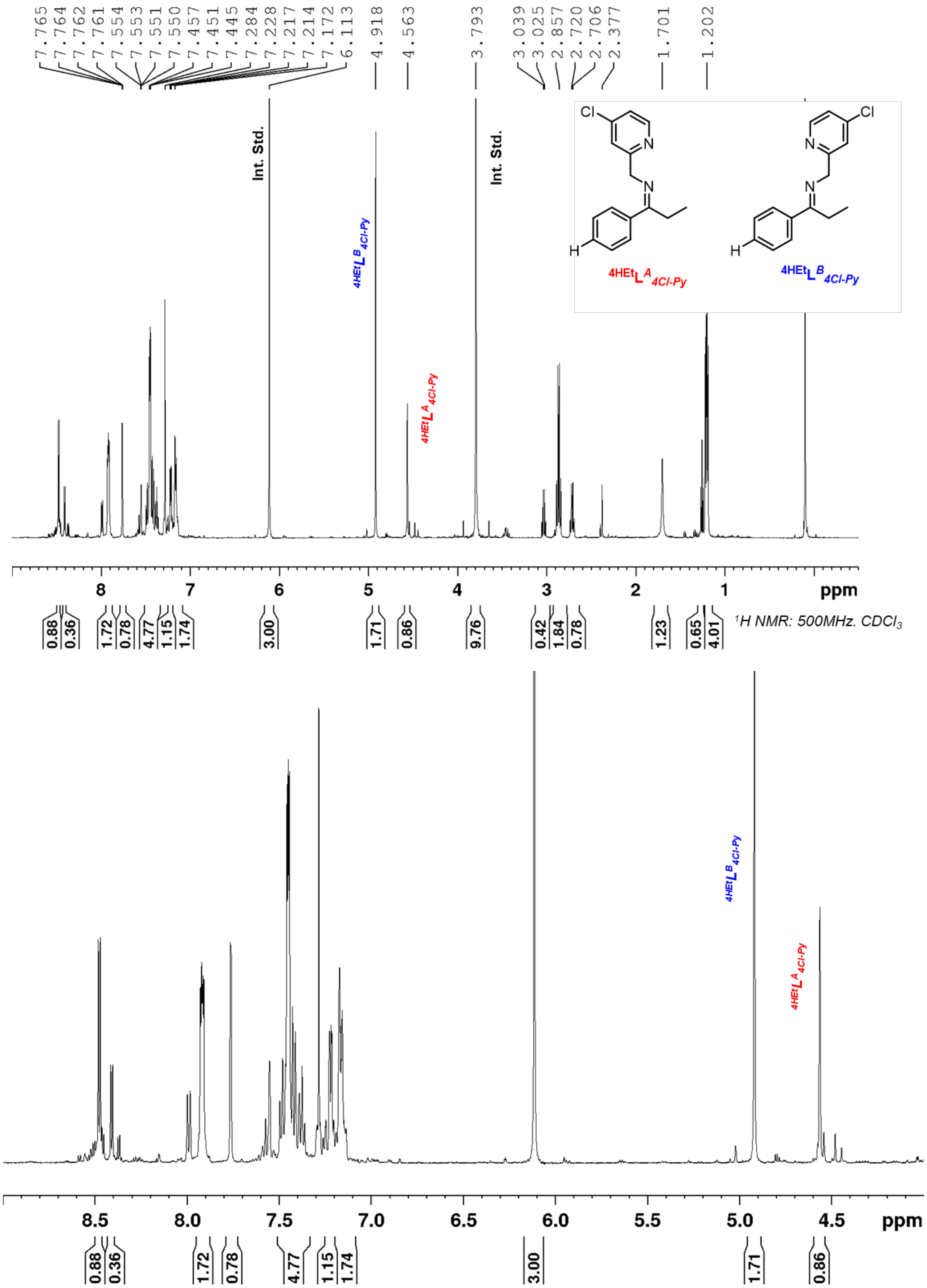

Figure S95. ^{1}H-NMR spectra of ^{4HEt}L$_{4Cl\text{-}Py}$. Note: two imine isomers were formed. The ratio of ^{4HEt}L$^{A}_{4Cl\text{-}Py}$ and ^{4HEt}L$^{B}_{4Cl\text{-}Py}$ (34/66) is calculated using the average of the integration of CH_2 peaks and CH peaks.

^{1}H-NMR spectra for the Hydroxylation of $^{4HEt}L_{4Cl\text{-}Py}$

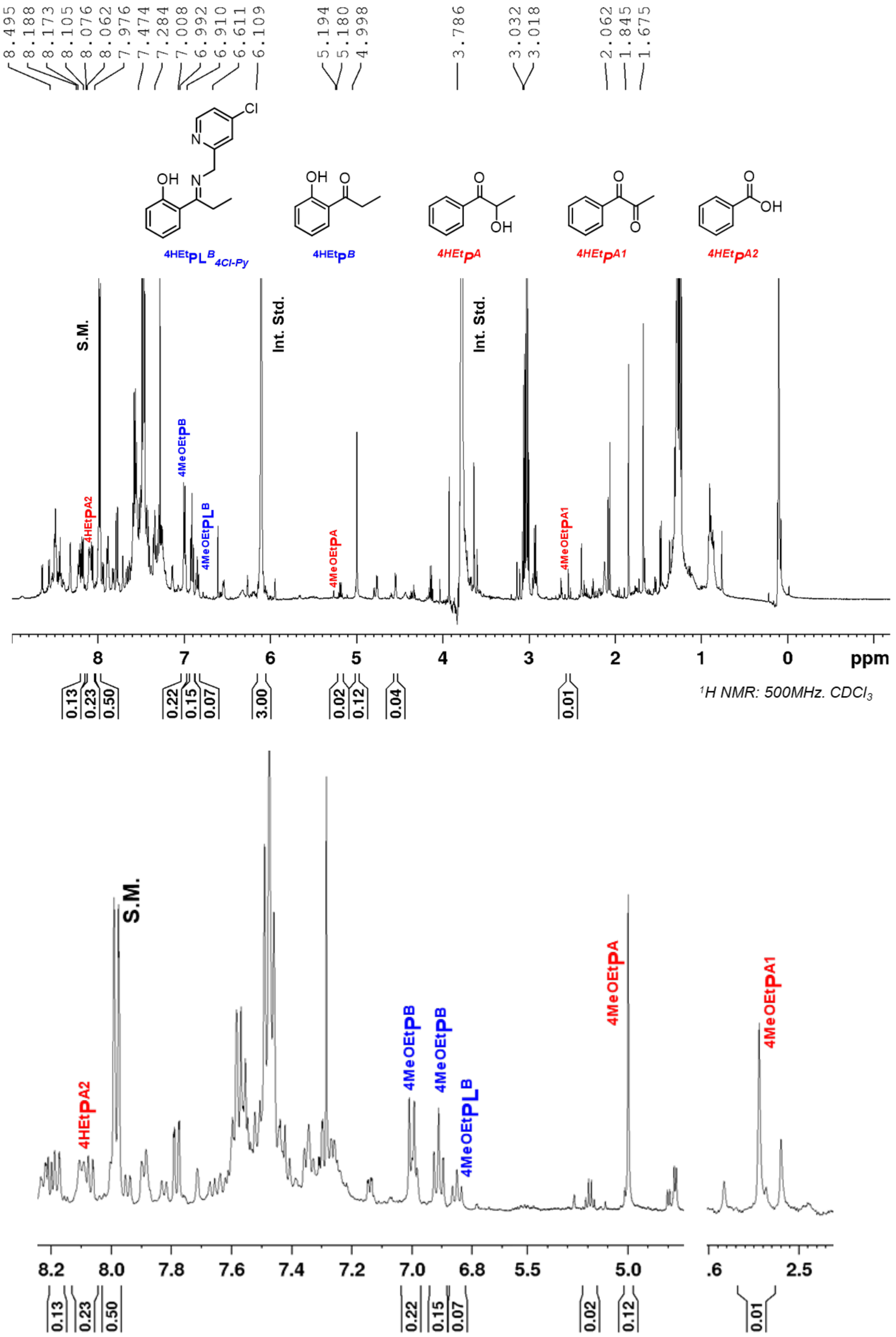

Figure S96. ^{1}H-NMR spectra for the hydroxylation of $^{4MeOEt}L$. Note: The ratio of $^{4HEt}PL^{A}_{4Cl\text{-}Py}$ and $^{4HEt}PL^{B}_{4Cl\text{-}Py}$ (39/61) is calculated using the average of the integration of CH_2 peaks and CH peaks.

3.34 ^{4HBn}S and 2-picolylamine

Synthesis of ^{4HBn}L

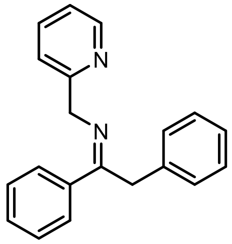

^{4HBn}L *A*

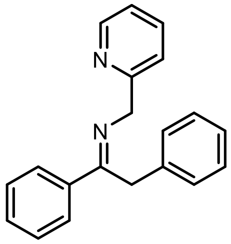

^{4HBn}L *B*

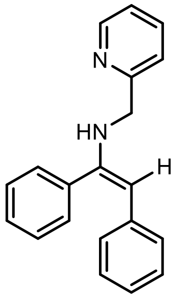

^{4HBn}L *C*

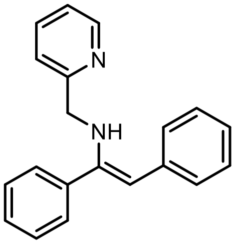

^{4HBn}L *D*

In an oven dried flask, 2-picolylamine (2.2 equiv., 2.2 mL) was added to 2-Phenylacetophenone (1.93 g, 9.85 mmol) and p- toluenesulfonic acid monohydrate (cat. 20 mg, 1.2 mol%) in toluene (50 mL). The reaction mixture was refluxed under argon with a Dean-Stark apparatus until imine formation was complete (12 hours). The reaction was cooled to room temperature and diluted with diethyl ether (30 mL). The organic layer was washed with saturated ammonia chloride (20 mL x 2), saturated aqueous sodium bicarbonate (20 mL), brine (20 mL), and dried with magnesium sulfate. The final product was isolated as a brown solid (94% yield, 2.64 g, 85% pure). ^{1}H-NMR (500 MHz, $CDCl_3$): δ 8.56 (d, 2H), 7.94 (d, 2H), 7.71 (d, 2H), 7.41 ((m, 6H), 7.37 (m, 6H), 7.29 (m, 4H), 7.21 (m, 6H), 4.72-4.55 (s, 2H). 13C{1H} NMR (500 MHz, CD_3CN): δ 167.6, 160.5, 149.0. 136.6, 128.9, 128.4, 127.3, 122.1, 121.8, 106.7, 98.3, 59.1, 49.4, 35.6. HRMS (ESI) m/z: [M + Na]+ Calcd for C20H18N2Na 286.3780, found 287.1513.

Hydroxylation of ^{4HBn}L

The reaction was carried out on 0.159 mmol scale using 53.6 mg of the imine according to the Standard Procedure. The reaction products were quantified using 0.159 mmol of 1,3,5-trimethoxybenzene (int. std.). (49% yield). The identity of the hydroxylation products was confirmed by ^{1}H-NMR.

Cleavage of ^{4HBn}PL

Dissolving ^{4HBn}*PL* in round bottom flask with 50 mL EtOAc, then adding 100 mL 1M HCl. Reaction was going for 30 min. The resulting mixture was extracted with EtOAc (50 mL X 2). The organic phases were separated, combined, dried over $MgSO_4$, filtered, and dried under vacuum. The reaction products were dissolved in 1.4 mL of $CDCl_3$ solution containing 13.5 mg of 1,3,5-trimethoxybenzene (internal standard). The reaction products were quantified by ^{1}H-NMR using integration signals that correspond to the starting material and products with the integration signal of the internal standard.

^{1}H NMR spectra of ^{4HBn}L

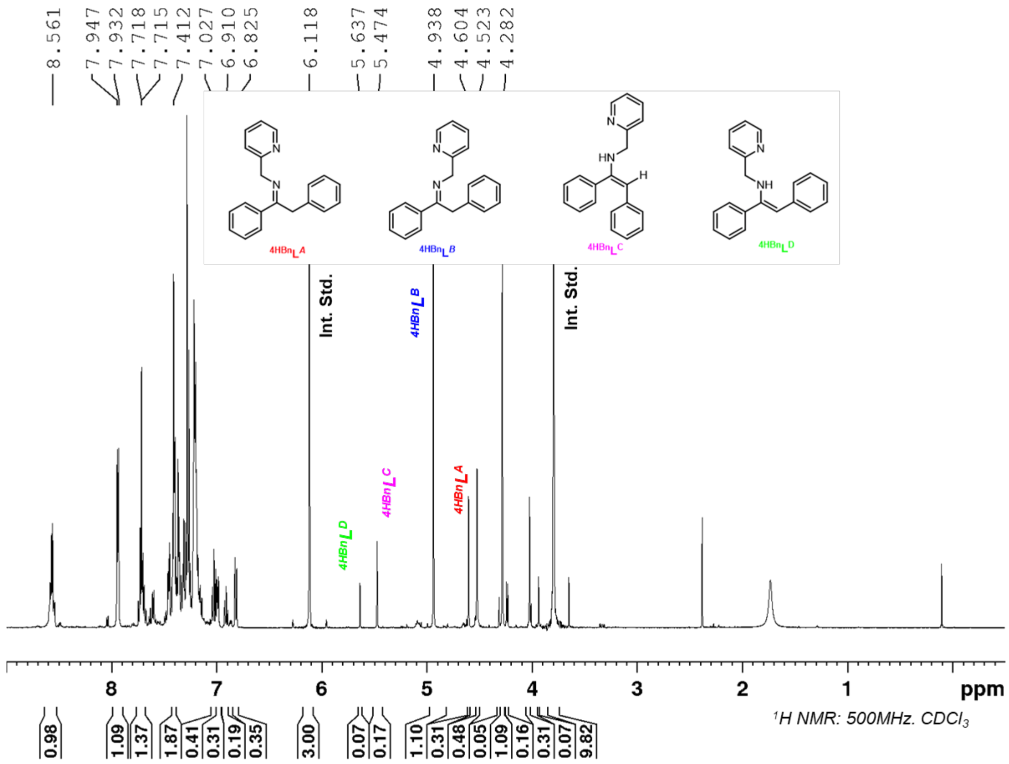

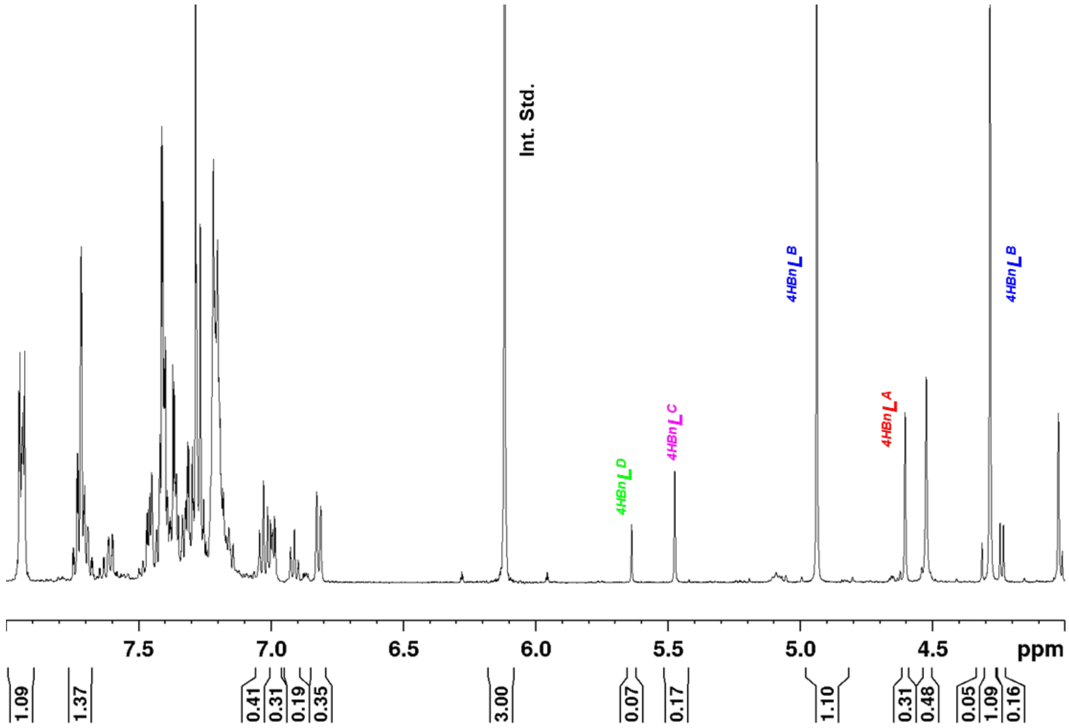

Figure S97. ^{1}H-NMR spectra of ^{4HBn}L. Note: two imine isomers were formed. The ratio of $^{4HBn}L^{A}$ and $^{4HBn}L^{B}$ (17/83) is calculated using the average of the integration of CH_2 peaks and CH peaks.

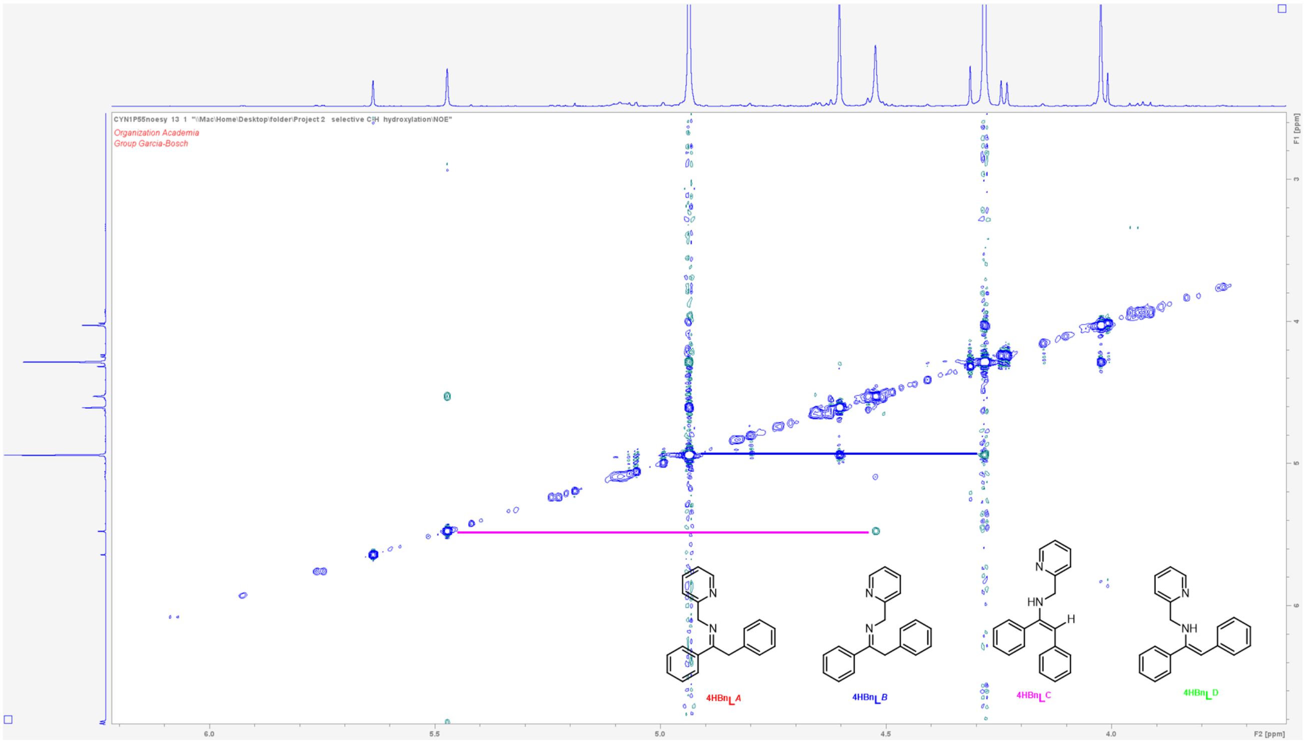

Figure S98. Nuclear Overhauser Effect Spectroscopy (NOESY) spectra for ^{4HBn}L.

^{1}H NMR spectra for the hydroxylation of ^{4HBn}L

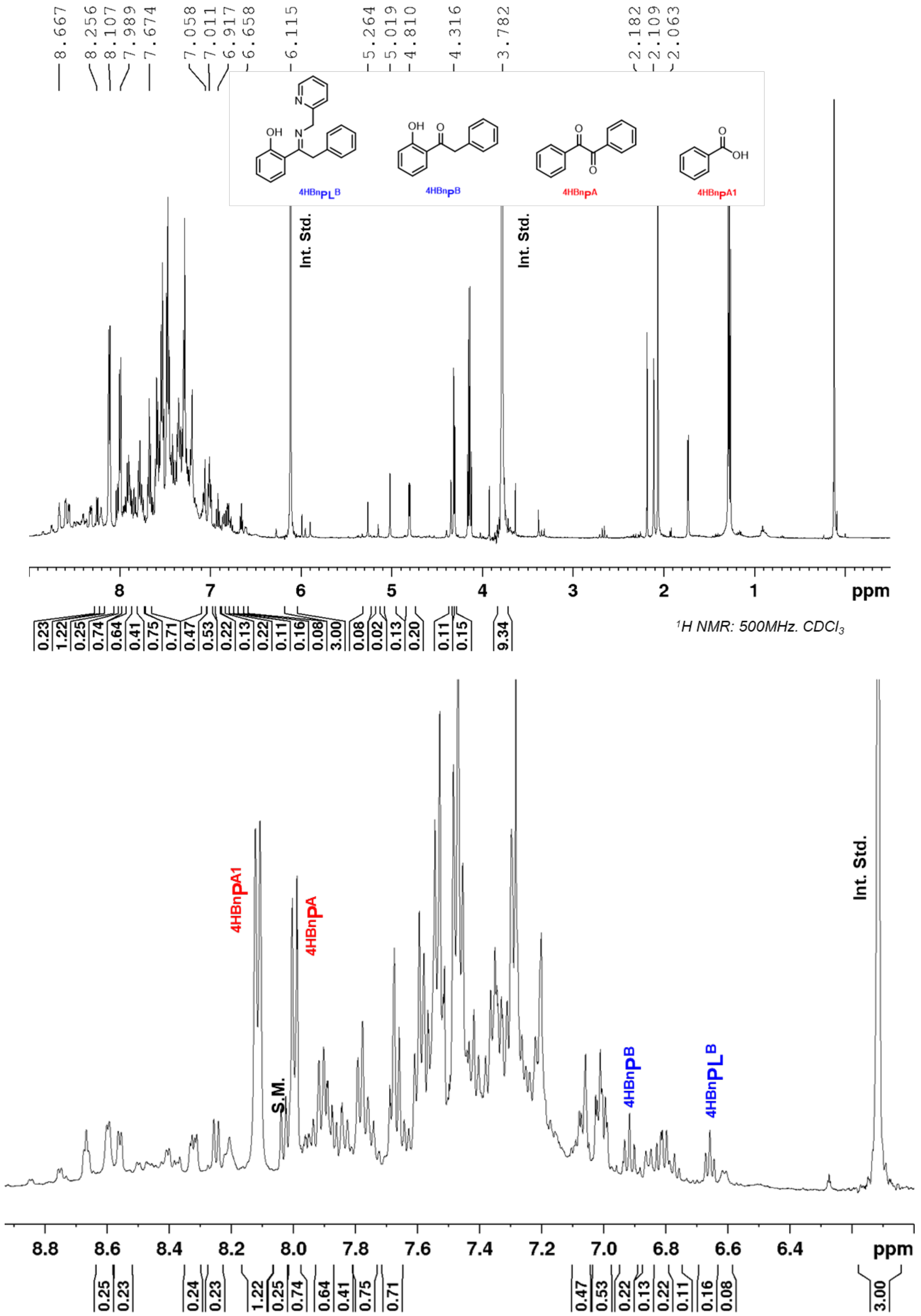

Figure S99. ^{1}H-NMR spectra for the hydroxylation of ^{4HBn}L. Note: The ratio of ^{4HBn}PL^A and ^{4HBn}PL^B (51/49) is calculated using the average of the integration of CH_2 peaks and CH peaks.

^{1}H NMR spectra for the Cleavage of ^{4HBn}PL

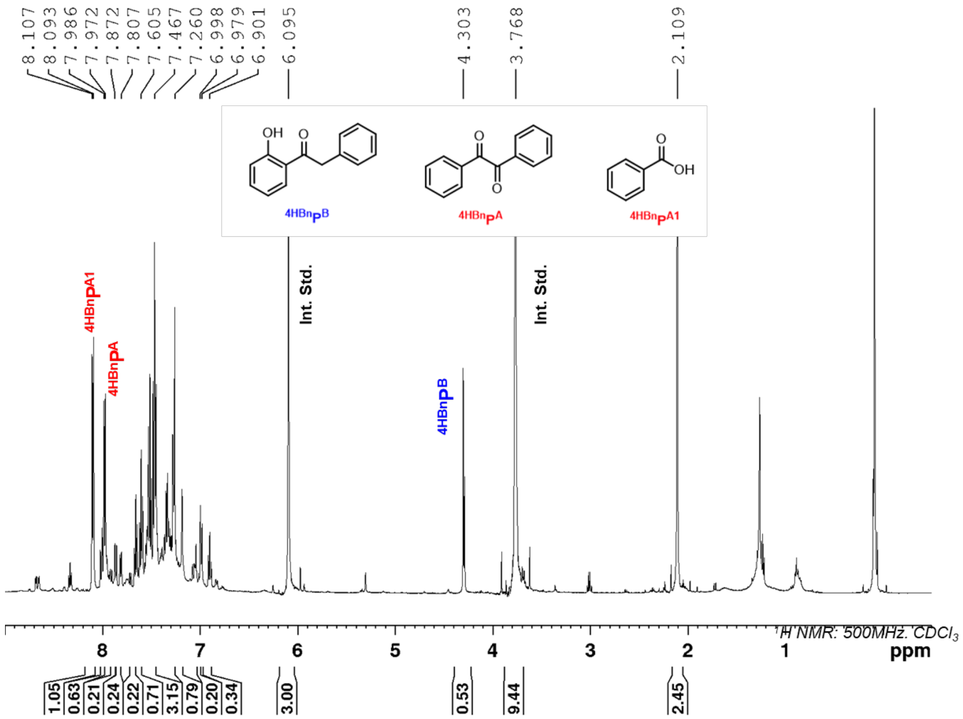

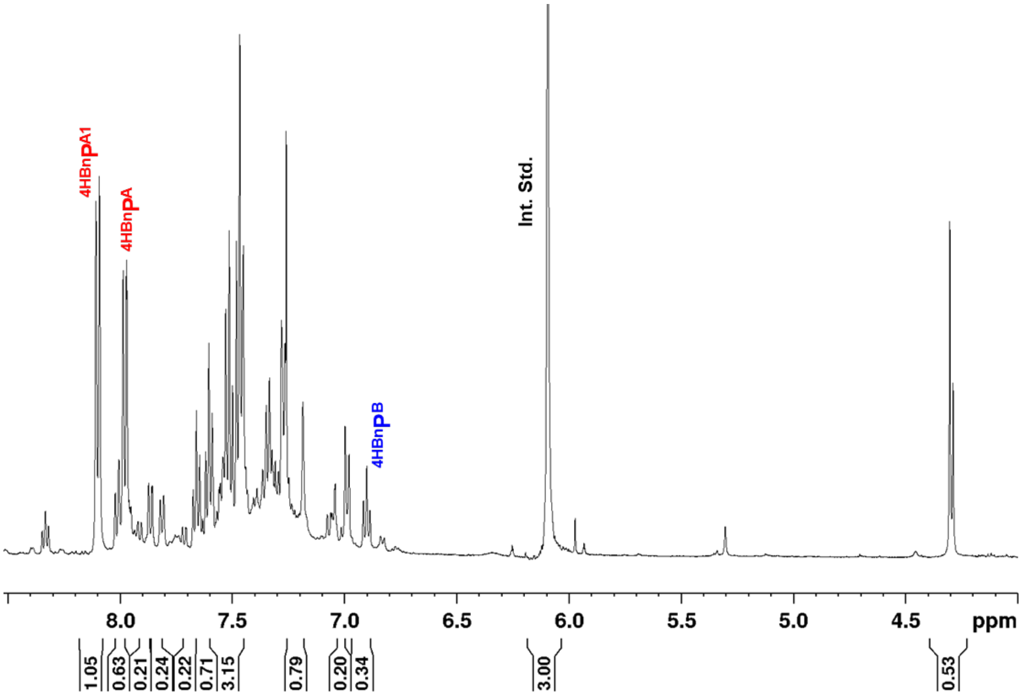

Figure S100. ^{1}H-NMR spectra for the cleavage of ^{4HBn}PL.

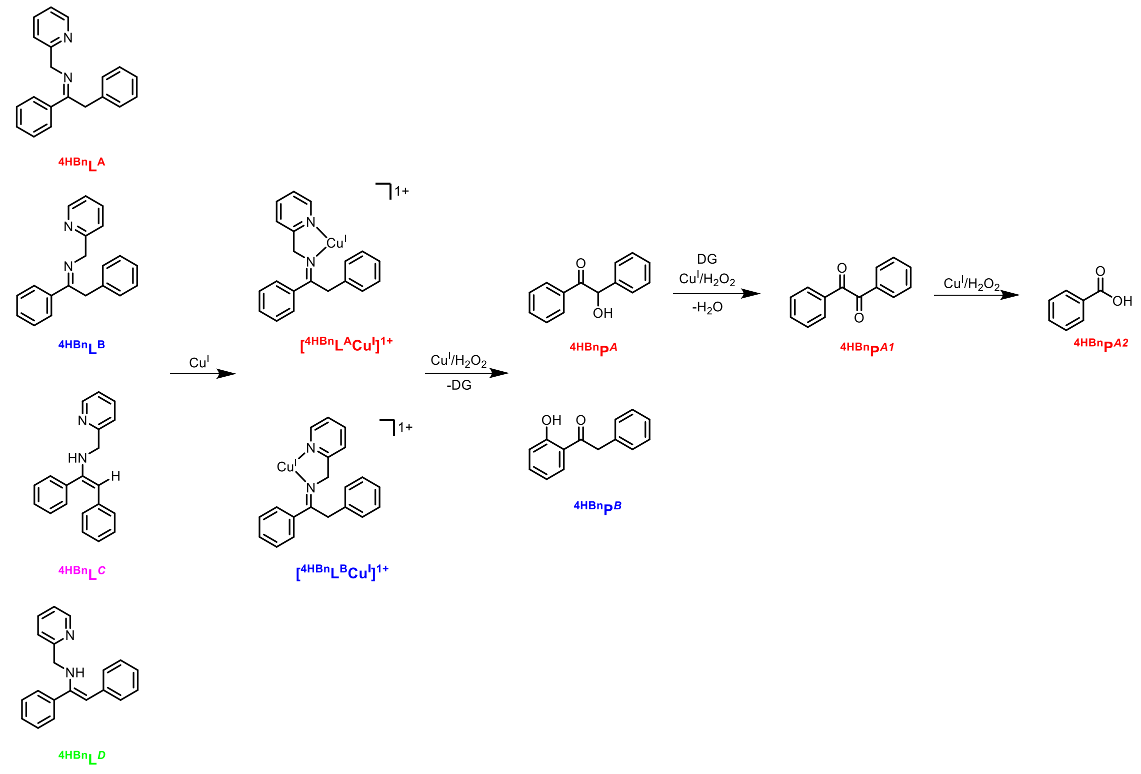

Scheme 2. Oxidation of ^{4HBn}L to all products

^{1}H-NMR spectra of $^{4HBn}L^{B}$ after addition of Cu^{I} in CD_3CN.

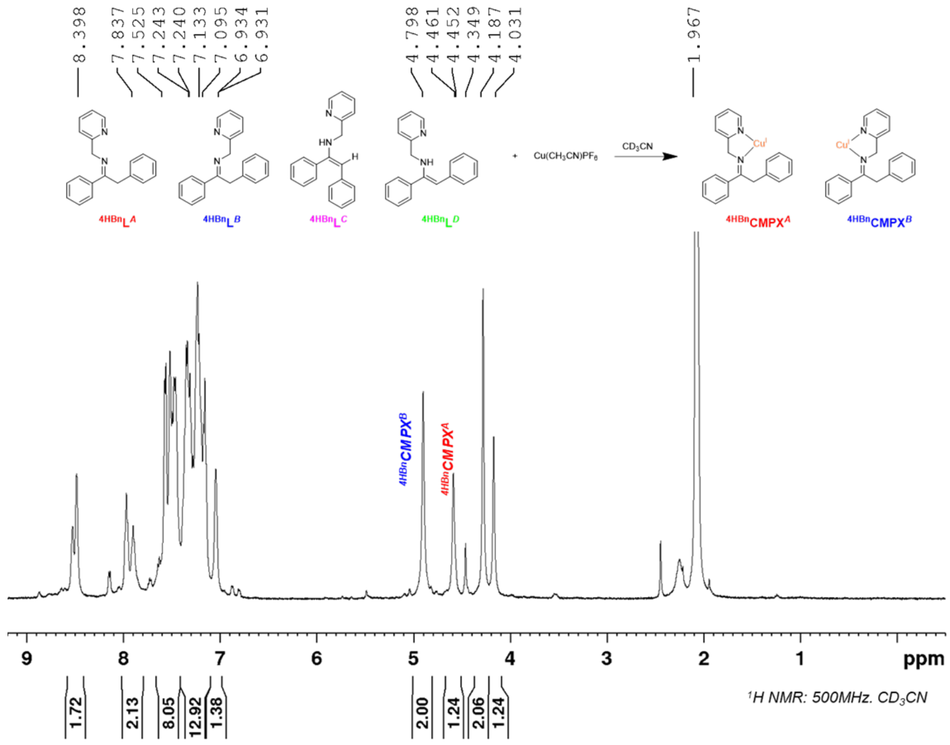

Figure S101. ^{1}H-NMR spectra of $^{4HBn}L^{B}$ after addition of Cu^{I} in CD_3CN.

^{1}H NMR spectra of the formation of benzil

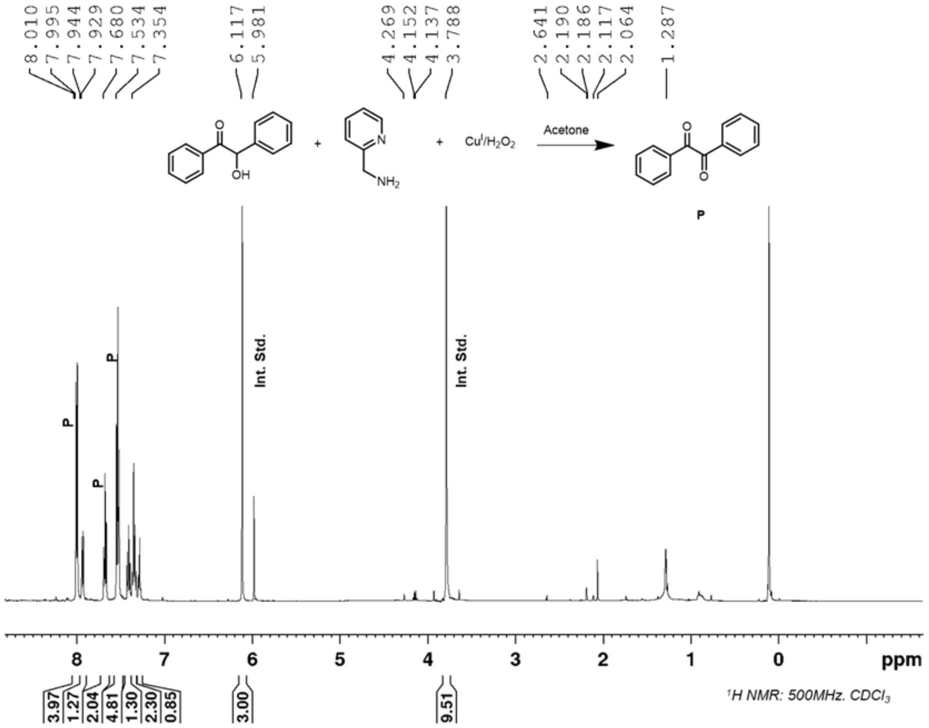

Figure S102. Evidence of formation of diketone from sp^3 hydroxylation product

^{1}H NMR spectra of the formation of benzoic acid

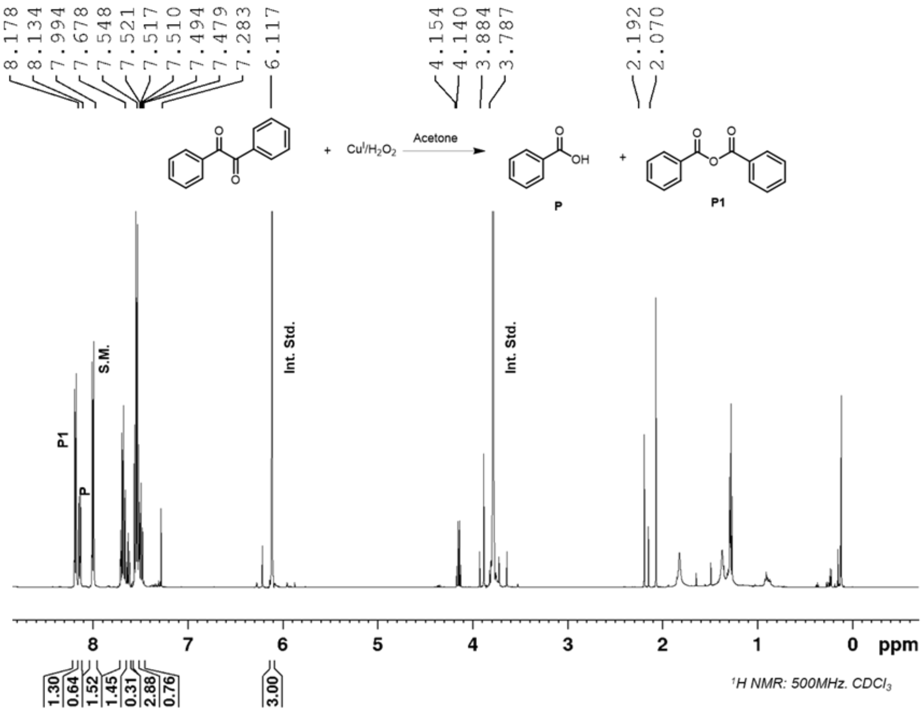

Figure S103. Evidence of formation of benzoic acid from benzil

3.35 ^{4MeOBn}S and 2-picolylamine

Synthesis of ^{4MeOBn}L

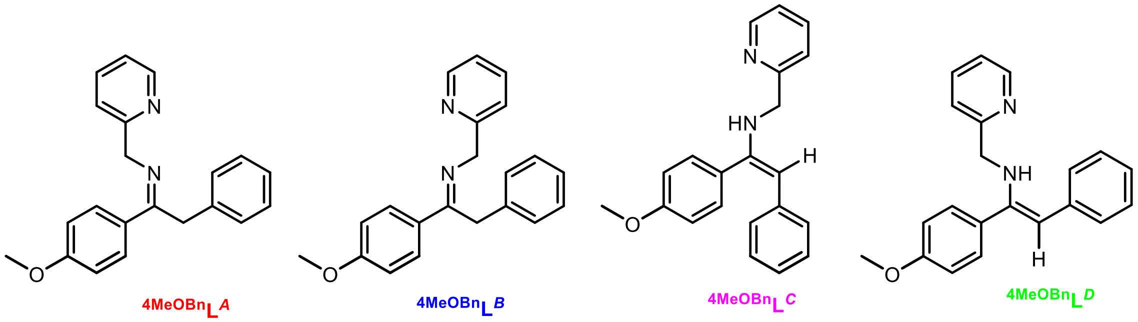

In an oven dried flask, 2-picolylamine (2.2 equiv., 2.2 mL) was added to 4-Methoxydeoxybenzoin (2.27 g, 9.85 mmol) and p- toluenesulfonic acid monohydrate (cat. 20 mg, 1.2 mol%) in toluene (50 mL). The reaction mixture was refluxed under argon with a Dean-Stark apparatus until imine formation was complete (12 hours). The reaction was cooled to room temperature and diluted with diethyl ether (30 mL). The organic layer was washed with saturated ammonia chloride (20 mL x 2), saturated aqueous sodium bicarbonate (20 mL), brine (20 mL), and dried with magnesium sulfate. The final product was isolated as a brown solid (95% yield, 2.96 g, 99% pure). ^{1}H-NMR (500 MHz, $CDCl_3$): δ 8.55 (d, 2H), 7.92 (d, 4H), 7.70 (d, 4H), 7.28 (d, 6H), 7.19 (d, 6H), 6.92 (d, 4H), 4.90 (s, 2H), 4.65 (s, 2H), 4.24 (s, 2H), 4.03 (s, 2H), 3.84 (s, 6H). 13C{1H} NMR (500 MHz, CD_3CN): δ 166.7, 161.0, 148.9, 136.6, 130.9, 129.3, 128.8, 122.0, 113.6, 57.5, 55.4, 45.2, 35.2. HRMS (ESI) m/z: [M + Na]+ Calcd for C21H20N2ONa 316.4040, found 317.1643.

Hydroxylation of ^{4MeOBn}L

The reaction was carried out on 0.159 mmol scale using 50.8 mg of the imine according to the Standard Procedure. The reaction products were quantified using 0.159 mmol of 1,3,5-trimethoxybenzene (int. std.). (58% yield). The identity of the hydroxylation products was confirmed by ^{1}H-NMR.

^{1}H NMR spectra of $^{4MeOBn}L$

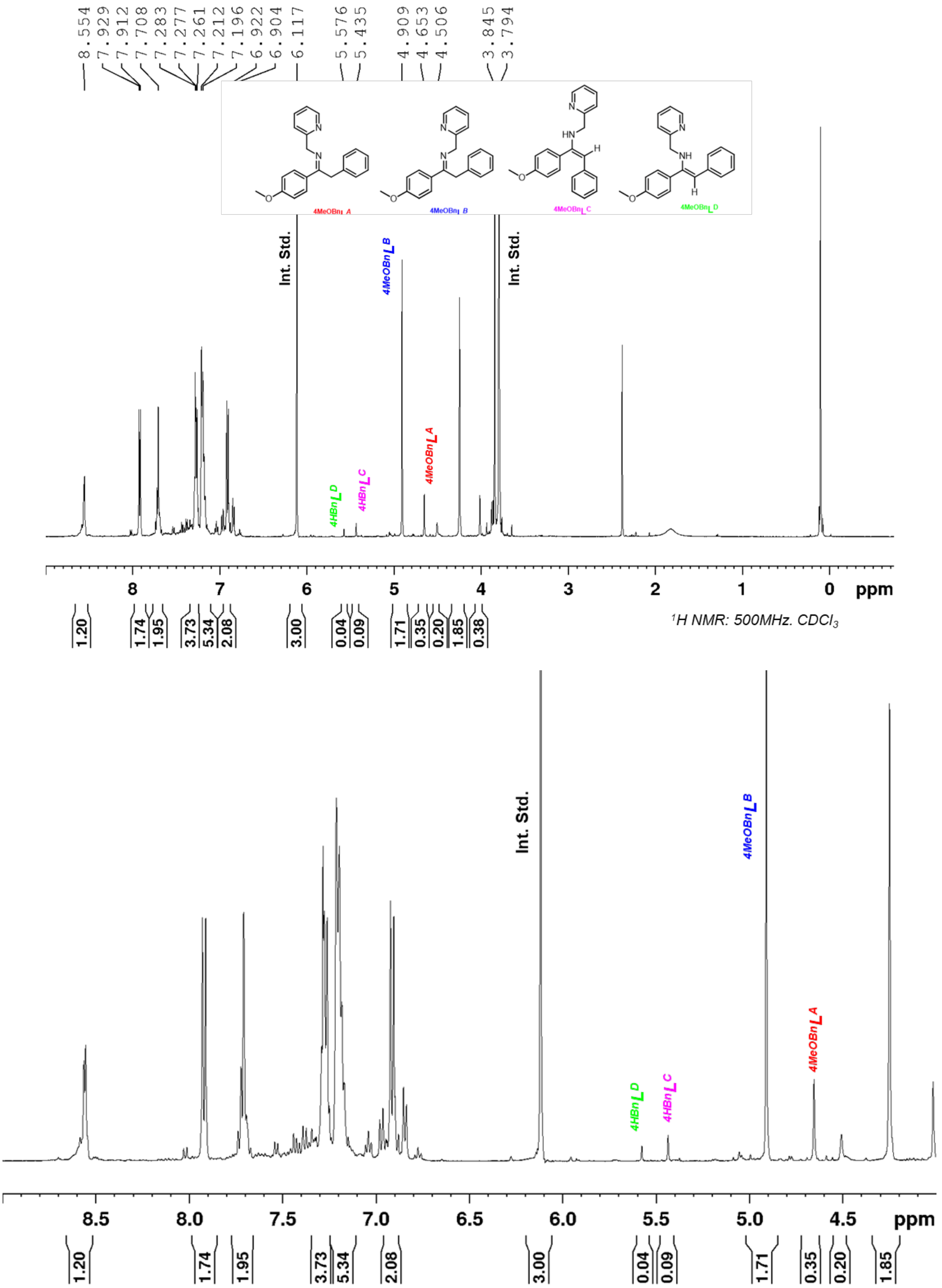

Figure S104. ^{1}H-NMR spectra of $^{4MeOBn}L$. Note: two imine isomers were formed. The ratio of $^{4MeOBn}L^{A}$ and $^{4MeOBn}L^{B}$ (17/83) is calculated using the average of the integration of CH_2 peaks and CH peaks.

^{1}H NMR spectra for the hydroxylation of ^{4MeOBn}L

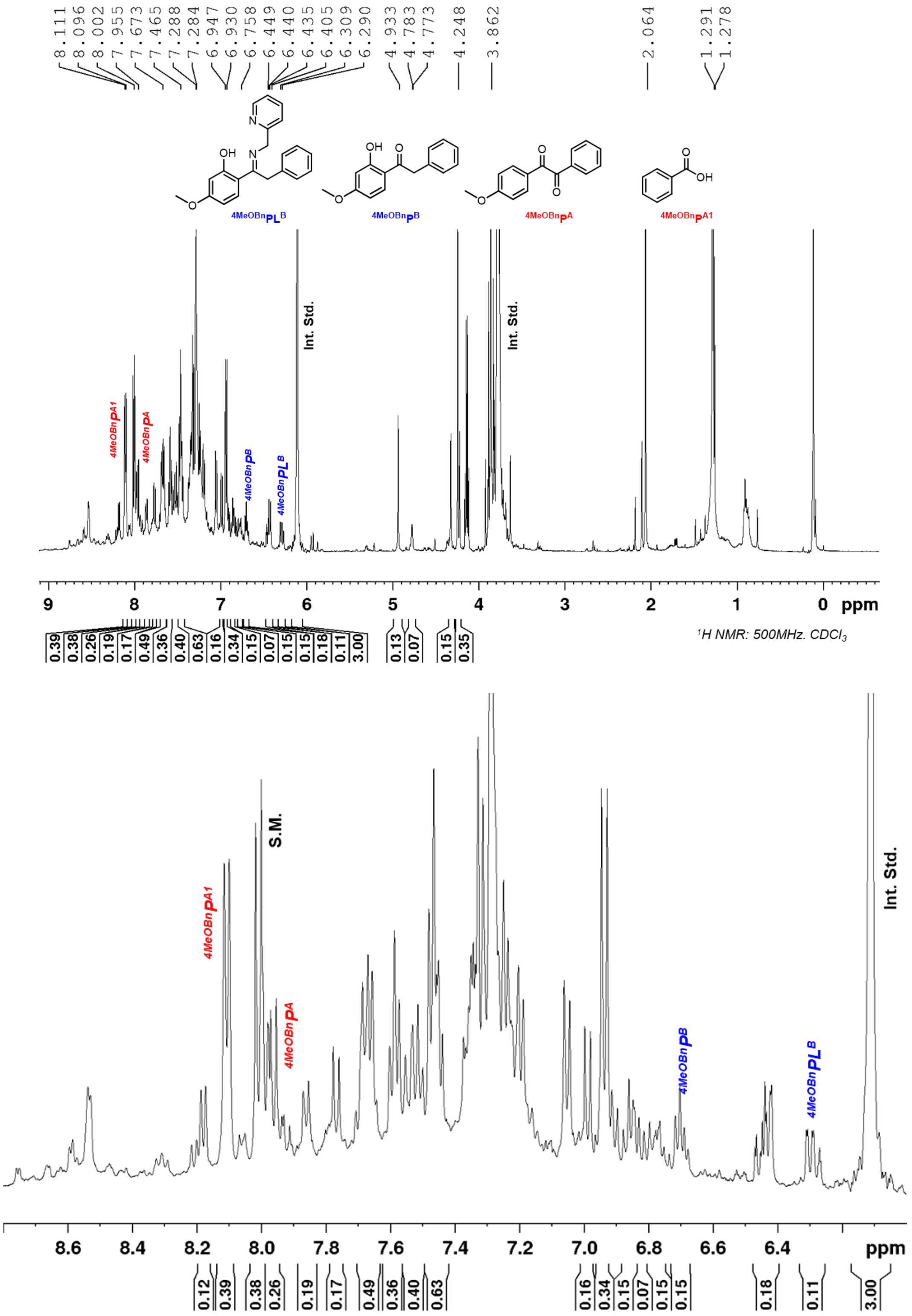

Figure S105. ^{1}H-NMR spectra for the hydroxylation of ^{4MeOBn}**L**. Note: The ratio of $^{4MeOBn}L^{A}$ and $^{4MeOBn}L^{B}$ (48/52) is calculated using the average of the integration of CH_2 peaks and CH peaks.

3.36 ^{4FBn}S and 2-picolylamine

Synthesis of ^{4FBn}L

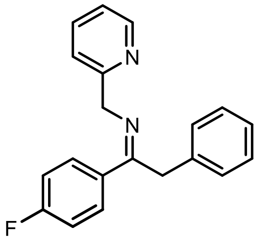

^{4FBn}L *A*

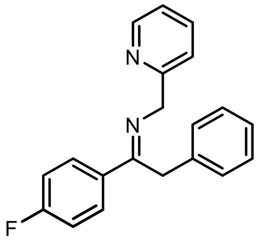

^{4FBn}L *B*

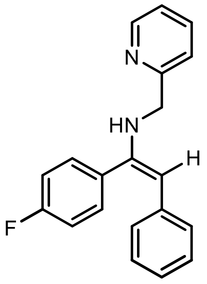

^{4FBn}L C

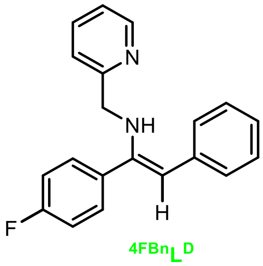

^{4FBn}L D

In an oven dried flask, 2-picolylamine (2.2 equiv., 2.2 mL) was added to Benzyl 4-fluorophenyl ketone (2.15 g, 9.85 mmol) and p- toluenesulfonic acid monohydrate (cat. 20 mg, 1.2 mol%) in toluene (50 mL). The reaction mixture was refluxed under argon with a Dean-Stark apparatus until imine formation was complete (12 hours). The reaction was cooled to room temperature and diluted with diethyl ether (30 mL). The organic layer was washed with saturated ammonia chloride (20 mL x 2), saturated aqueous sodium bicarbonate (20 mL), brine (20 mL), and dried with magnesium sulfate. The final product was isolated as a brown solid (94% yield, 2.81 g, 94% pure). ^{1}H-NMR (500 MHz, $CDCl_3$): δ 8.57 (d, 2H), 7.93 (m, 2H), 7.73 (t, 2H), 7.68 (d, 2H), 7.28 (t, 4H), 7.19 (m, 10H), 7.06 (t, 4H), 4.92 (s, 2H), 4.59 (s, 2H), 4.25 (s, 2H), 4.0 (s, 2H). 13C{1H} NMR (500 MHz, CD_3CN): δ 196.0, 166.8, 166.4, 149.0, 136.7, 129.4, 128.8, 126.6, 122.1, 121.8, 115.9, 115.7, 57.8, 45.5, 35.5. HRMS (ESI) m/z: [M + Na]+ Calcd for C20H17FN2Na 304.3684, found 305.1442.

Hydroxylation of ^{4FBn}L

The reaction was carried out on 0.159 mmol scale using 51.5 mg of the imine according to the Standard Procedure. The reaction products were quantified using 0.159 mmol of 1,3,5-trimethoxybenzene (int. std.). (59% yield). The identity of the hydroxylation products was confirmed by ^{1}H-NMR.

^{1}H NMR spectra of ^{4FBn}L

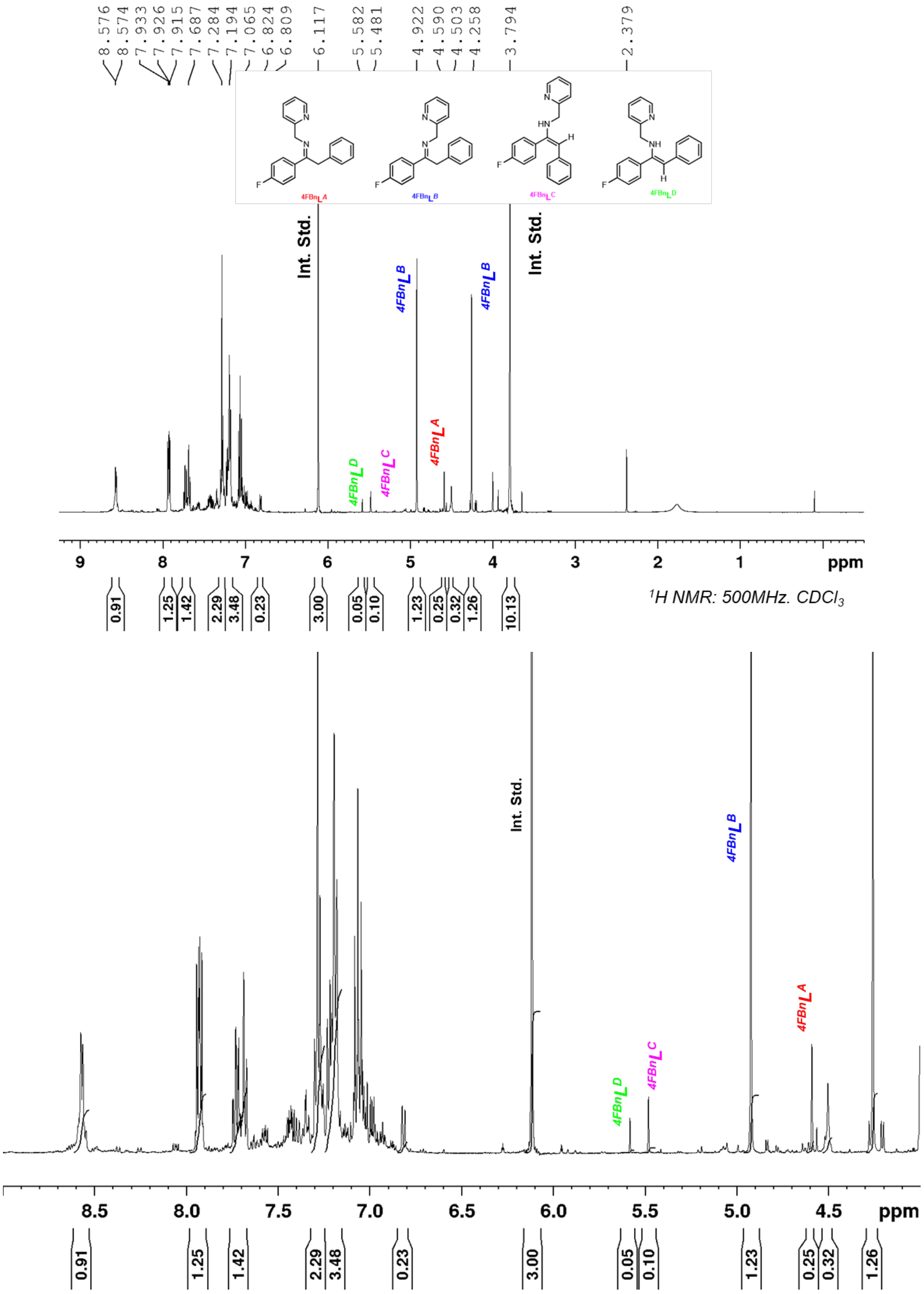

Figure S106. ^{1}H-NMR spectra of ^{4FBn}L. Note: two imine isomers were formed. The ratio of $^{4FBn}L^{A}$ and $^{4FBn}L^{B}$ (17/83) is calculated using the average of the integration of CH_2 peaks and CH peaks.

^{1}H NMR spectra for the hydroxylation of ^{4FBn}L

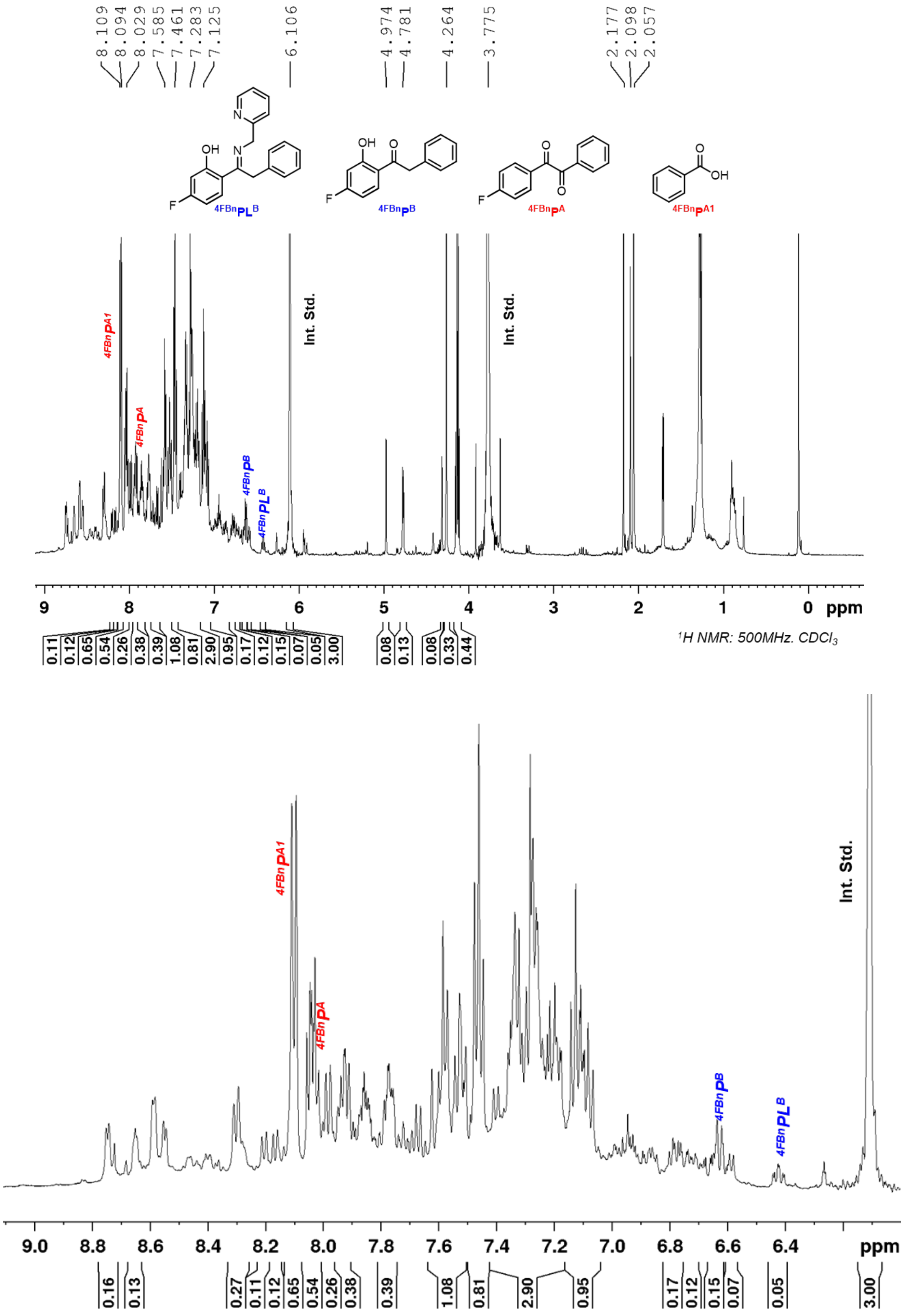

Figure S107. ^{1}H-NMR spectra for the hydroxylation of ^{4FBn}L. Note: The ratio of $^{4FBn}P^{A}$ and $^{4FBn}P^{B}$ (48/52) is calculated using the average of the integration of CH_2 peaks and CH peaks.

3.37 ^{4Hbn}S and 2-(aminomethyl)-4-methoxypyridine

Synthesis of $^{4HBn}L_{4MeO\text{-}Py}$

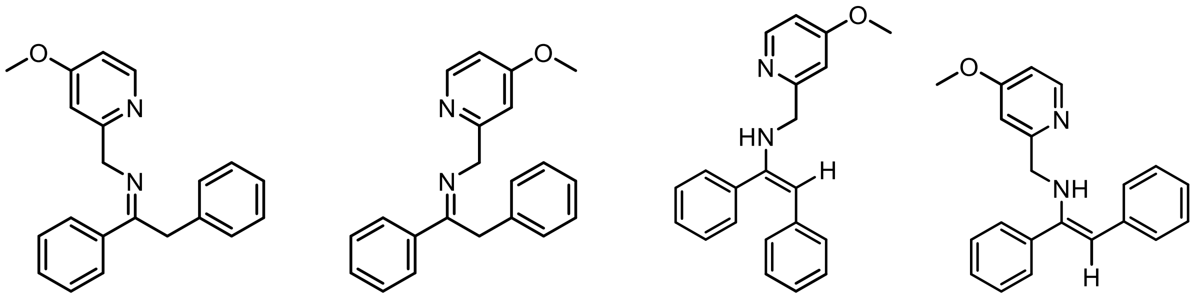

$^{4HBn}L^{A}_{4MeO\text{-}Py}$ $^{4HBn}L^{B}_{4MeO\text{-}Py}$ $^{4HBn}L^{C}_{4MeO\text{-}Py}$ $^{4HBn}L^{D}_{4MeO\text{-}Py}$

In an oven dried flask, 2-(aminomethyl)-4-methoxypyridine (2.2 equiv., 0.33 mL) was added to 2-Phenylacetophenone (360 mg, 1.50 mmol) and p- toluenesulfonic acid monohydrate (cat. 10 mg, 3.9 mol%) in toluene (50 mL). The reaction mixture was refluxed under argon with a Dean-Stark apparatus until imine formation was complete (12 hours). The reaction was cooled to room temperature and diluted with diethyl ether (30 mL). The organic layer was washed with saturated ammonia chloride (20 mL x 2), saturated aqueous sodium bicarbonate (20 mL), brine (20 mL), and dried with magnesium sulfate. The final product was isolated as a brown solid (94% yield, 446 mg, 78% pure). ^{1}H-NMR (500 MHz, $CDCl_3$): δ 8.38 (d, 1H), 8.37 (d, 1H), 7.94 (d, 2H), 7.41 (d, 4H), 7.28 (d, 4H), 7.20 (t, 6H), 7.00 (m, 6H), 6.72 (m, 2H), 6.71 (d, 2H). 4.88 (s, 2H), 4.56 (s, 2H), 4.27 (s, 2H), 4.02 (s, 2H), 3.91 (s, 3H), 3.85 (s, 3H). 13C{1H} NMR (500 MHz, CD_3CN): δ 197.6, 167.7, 162.3, 150.4, 140.2, 136.6, 134.5, 129.4, 127.6, 127.3, 126.8, 108.1, 57.5, 55.1, 45.5, 35.6. HRMS (ESI) m/z: [M + Na]+ Calcd for C21H20N2ONa 316.4040, found 317.1642.

Hydroxylation of $^{4HBn}L_{4MeO\text{-}Py}$

The reaction was carried out on 0.159 mmol scale using 74 mg of the imine according to the Standard Procedure. The reaction products were quantified using 0.159 mmol of 1,3,5-trimethoxybenzene (int. std.). (70% yield). The identity of the hydroxylation products was confirmed by ^{1}H-NMR.

¹H spectra of ⁴ᴴᴮⁿL₄ₘₑₒ₋ₚᵧ

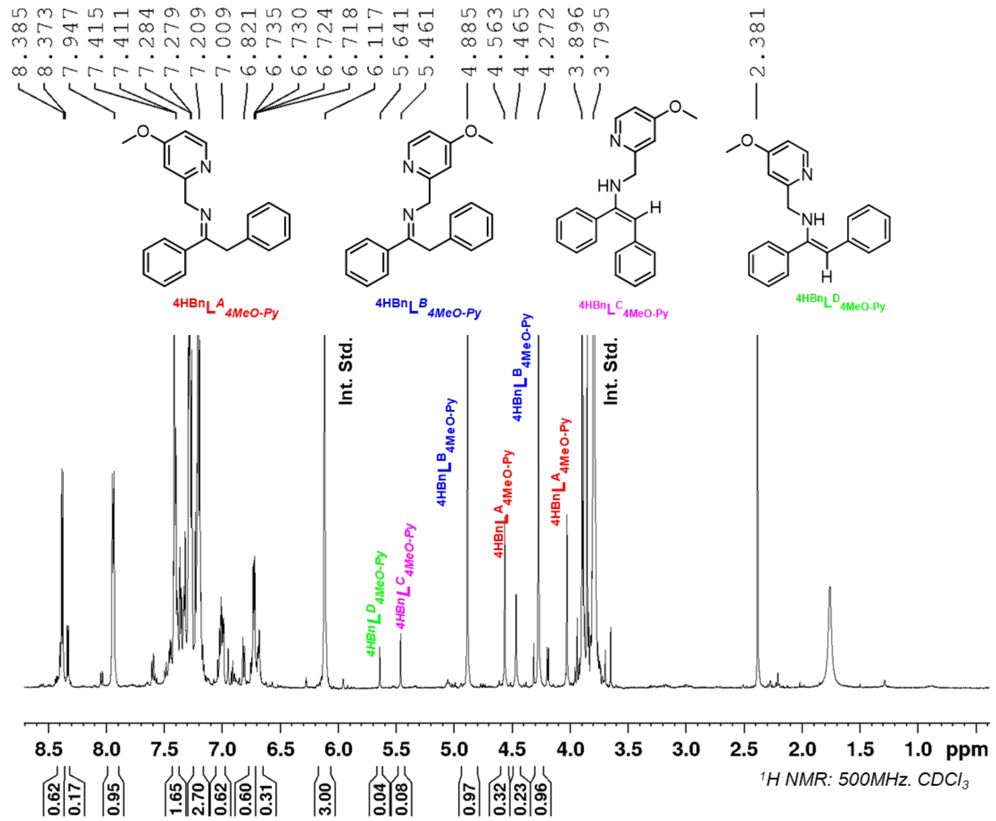

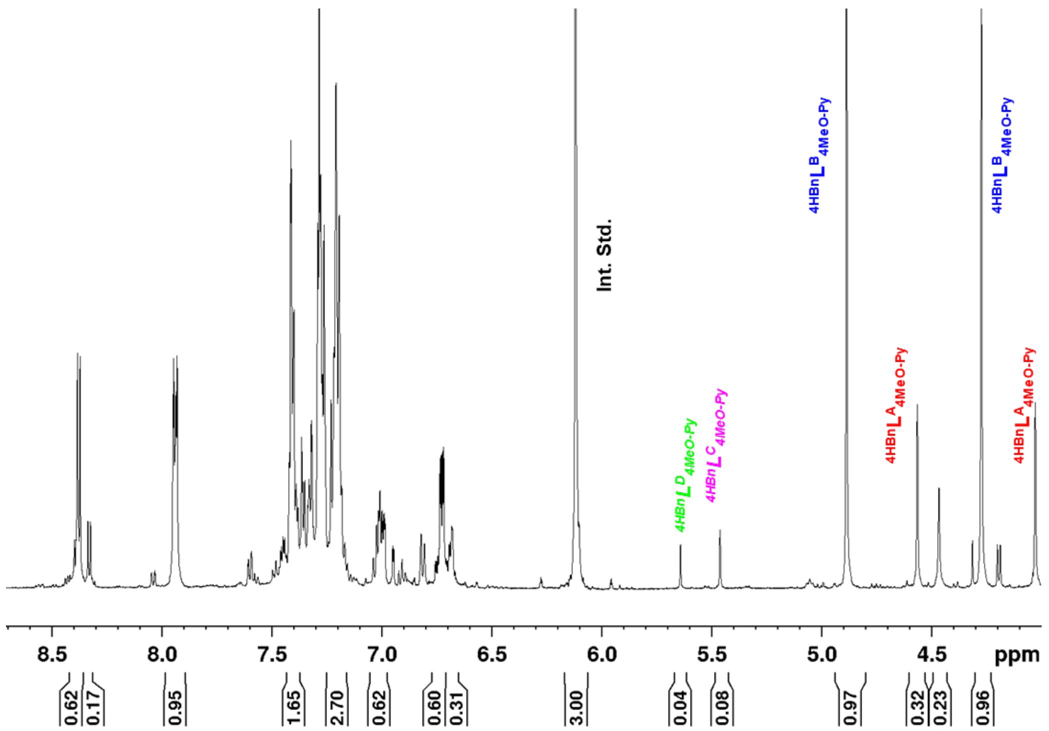

Figure S108. ¹H-NMR spectra of *4HBn*L*4MeO-Py*. Note: two imine isomers were formed. The ratio of 4HBnL*A*4MeO-Py and 4HBnL*B*4MeO-Py (17/83) is calculated using the average of the integration of CH_2 peaks and CH peaks.

^{1}H spectra for the hydroxylation of $^{4HBn}L_{4MeO\text{-}Py}$

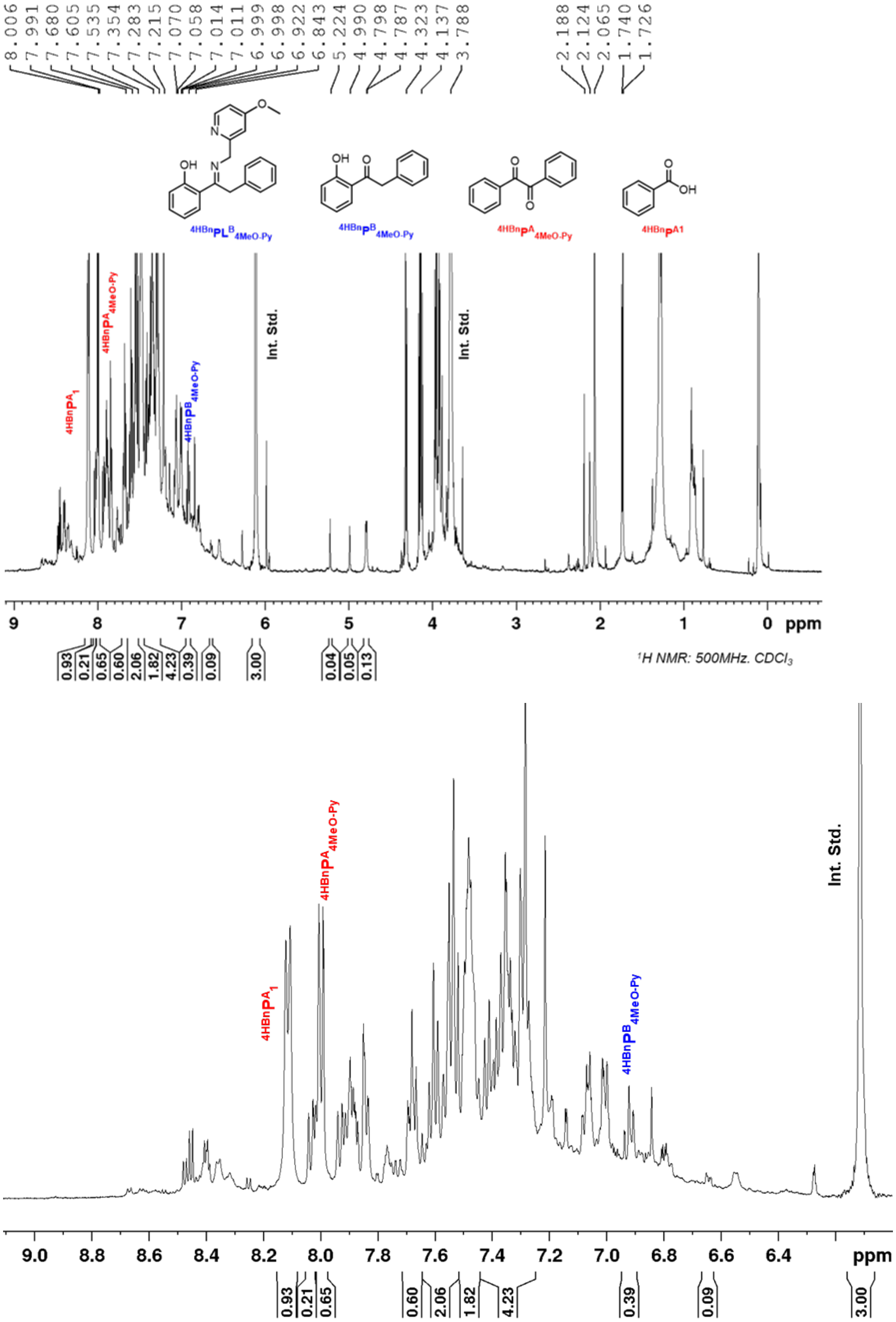

Figure S109. ^{1}H-NMR spectra for the hydroxylation of $^{4HBn}L_{4MeO\text{-}Py}$. Note: The ratio of $^{4HBn}P^{A}_{4MeO\text{-}Py}$ and $^{4HBn}P^{B}_{4MeO\text{-}Py}$ (46/54) is calculated using the average of the integration of CH_2 peaks and CH peaks.

3.38 ^{4HBn}S and 2-(aminomethyl)-4-chloropyridine

Synthesis of $^{4HBn}L_{4Cl\text{-}Py}$

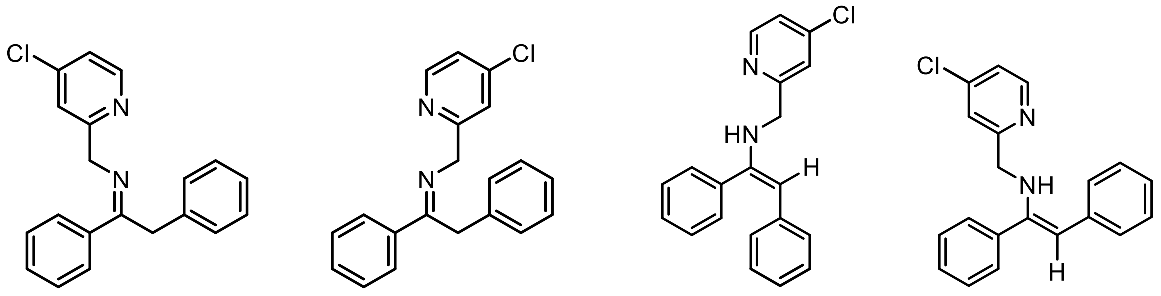

$^{4HBn}L^{A}_{4Cl\text{-}Py}$ $^{4HBn}L^{B}_{4Cl\text{-}Py}$ $^{4FBn}L^{C}_{4Cl\text{-}Py}$ $^{4FBn}L^{D}_{4Cl\text{-}Py}$

In an oven dried flask, 2-(aminomethyl)-4-chloropyridine (2.2 equiv., 0.46 mL) was added to 2-Phenylacetophenone (400 mg, 2.04 mmol) and p- toluenesulfonic acid monohydrate (cat. 10 mg, 2.8 mol%) in toluene (50 mL). The reaction mixture was refluxed under argon with a Dean-Stark apparatus until imine formation was complete (12 hours). The reaction was cooled to room temperature and diluted with diethyl ether (30 mL). The organic layer was washed with saturated ammonia chloride (20 mL x 2), saturated aqueous sodium bicarbonate (20 mL), brine (20 mL), and dried with magnesium sulfate. The final product was isolated as a brown solid (88% yield, 574 mg, 90% pure). ^{1}H-NMR (500 MHz, $CDCl_3$): δ 8.47 (m, 2H), 7.95 (m, 4H), 7.77 (s, 2H), 7.42 (d, 4H), 7.37 (m, 4H), 7.27 (m, 6H), 7.19 (m, 6H), 4.89 (s, 2H), 4.57 (s, 2H), 4.26 (s, 2H), 4.40 (s, 2H). 13C{1H} NMR (500 MHz, CD_3CN): δ 197.6, 168.2, 149.8, 133.2, 129.5, 128.9, 128.7, 128.2, 127.3, 57.1, 47.2, 45.5, 35.7. HRMS (ESI) m/z: [M + Na]+ Calcd for C20H17ClN2Na 320.8200, found 321.1147.

Hydroxylation of $^{4HBn}L_{4Cl\text{-}Py}$

The reaction was carried out on 0.159 mmol scale using 56.0 mg of the imine according to the Standard Procedure. The reaction products were quantified using 0.159 mmol of 1,3,5-trimethoxybenzene (int. std.). (51% yield). The identity of the hydroxylation products was confirmed by ^{1}H-NMR.

^{1}H NMR spectra of $^{4HBn}L_{4Cl\text{-}Py}$

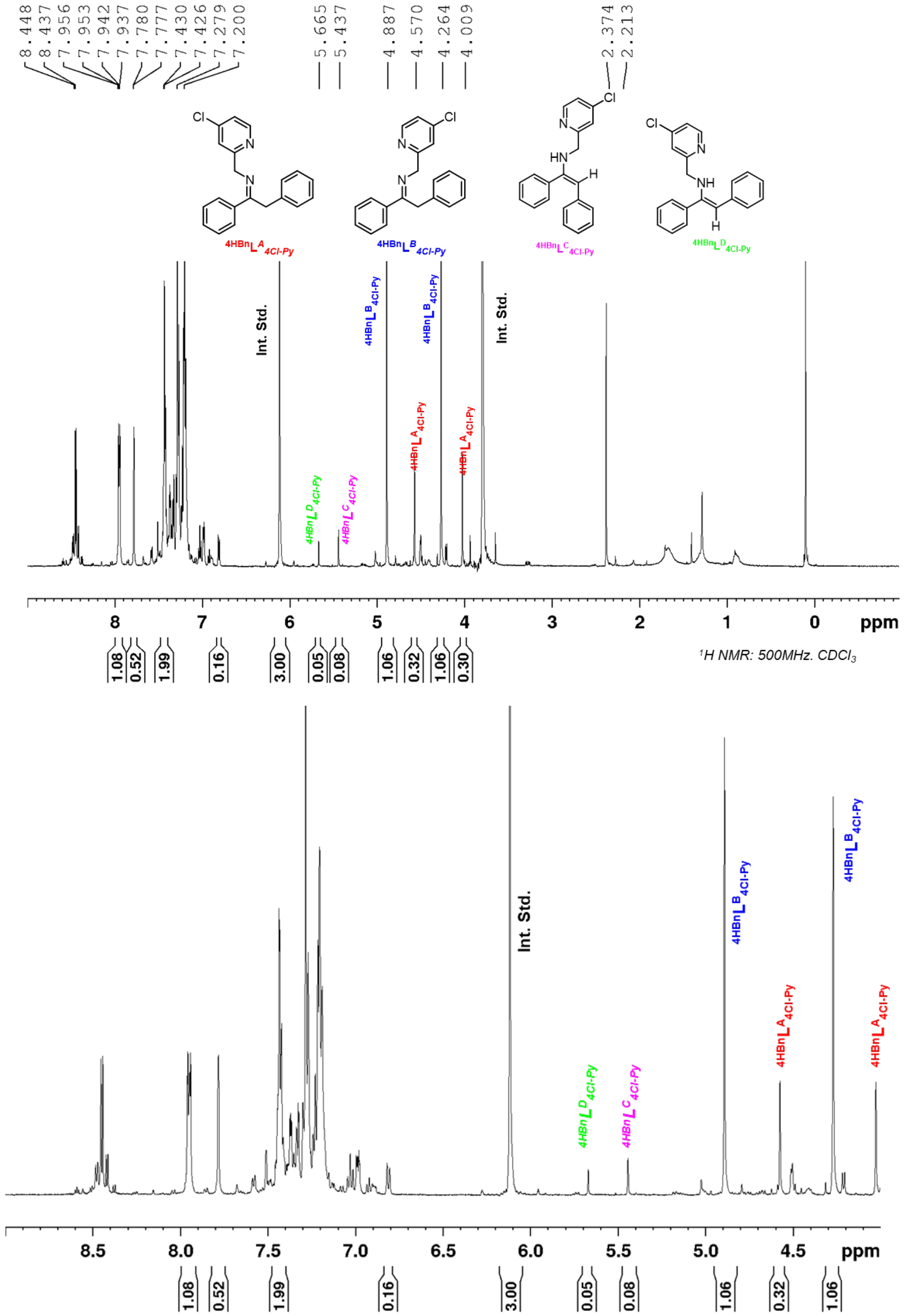

Figure S110. ^{1}H-NMR spectra of $^{4HBn}L_{4Cl\text{-}Py}$. Note: Note: two imine isomers were formed. The ratio of $^{4HBn}L^{A}_{4Cl\text{-}Py}$ and $^{4HBn}L^{B}_{4Cl\text{-}Py}$ (23/77) is calculated using the average of the integration of CH_2 peaks and CH peaks.

^{1}H NMR spectra for the Hydroxylation of $^{4HBn}L_{4Cl-Py}$

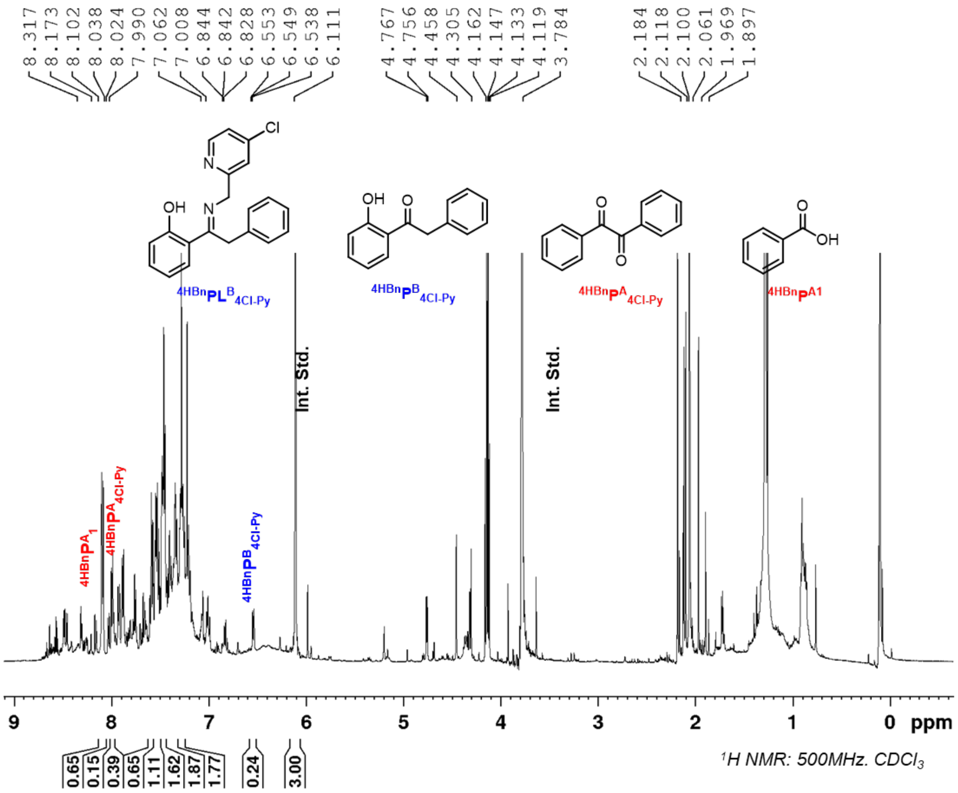

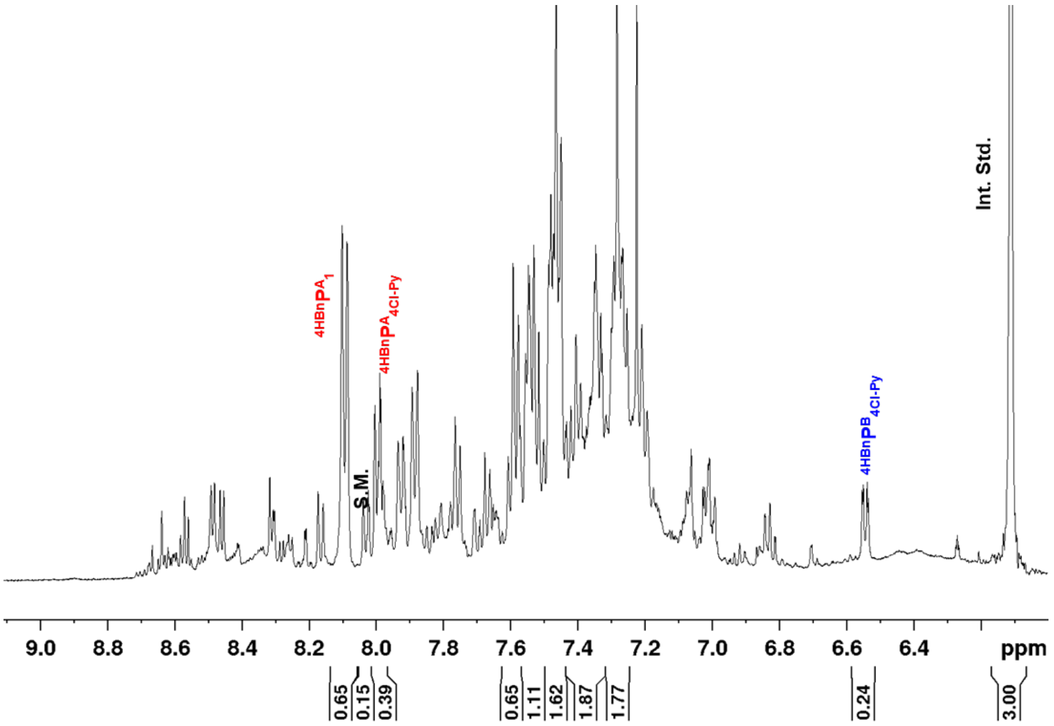

Figure S111. ^{1}H-NMR spectra for the hydroxylation of $^{4HBn}L_{4Cl\text{-}Py}$. Note: The ratio of $^{4HBn}L^{A}_{4Cl\text{-}Py}$ and $^{4HBn}L^{B}_{4Cl\text{-}Py}$ (53/47) is calculated using the average of the integration of CH_2 peaks and CH peaks.

3.39 ^{4HiBu}S and 2-picolylamine

Synthesis of ^{4HiBu}L

Molecular Weight: 252.3610

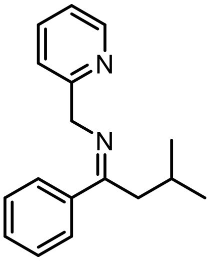

^{4HiBu}L *A*

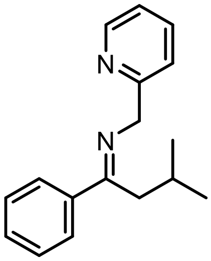

^{4HiBu}L *B*

In an oven dried flask, 2-picolylamine (2.2 equiv., 2.2 mL) was added to Isobutyl phenyl ketone (1.66 g, 9.85 mmol) and p- toluenesulfonic acid monohydrate (cat. 20 mg, 1.2 mol%) in toluene (50 mL). The reaction mixture was refluxed under argon with a Dean-Stark apparatus until imine formation was complete (12 hours). The reaction was cooled to room temperature and diluted with diethyl ether (30 mL). The organic layer was washed with saturated ammonia chloride (20 mL x 2), saturated aqueous sodium bicarbonate (20 mL), brine (20 mL), and dried with magnesium sulfate. The final product was isolated as a brown solid (92% yield, 2.29 g, 90% pure). ^{1}H-NMR (500 MHz, $CDCl_3$): δ 8.57 (d, 1H), 8.53 (d, 1H), 7.86 (d, 2H), 7.70 (m, 2H), 7.41 (m, 10H), 7.19 (d, 2H), 4.98 (s, 2H), 4.60 (s, 2H), 2.80 (d, 2H), 2.61 (d, 2H), 1.97 (m, 2H), 1.00 (d, 6H), 0.95 (d, 6H). 13C{1H} NMR (500 MHz, CD_3CN): δ 148.8, 136.5, 127.6, 121.9, 112.9, 56.4, 55.2, 30.1, 28.2, 22.6. HRMS (ESI) m/z: [M + Na]+ Calcd for C17H20N2Na 252.3610, found 253.0715.

Hydroxylation of ^{4HiBu}L

The reaction was carried out on 0.159 mmol scale using 40.0 mg of the imine according to the Standard Procedure. The reaction products were quantified using 0.159 mmol of 1,3,5-trimethoxybenzene (int. std.). (59% yield). The identity of the hydroxylation products was confirmed by ^{1}H-NMR.

¹H NMR spectra of ⁴ᴴⁱᴮᵘL

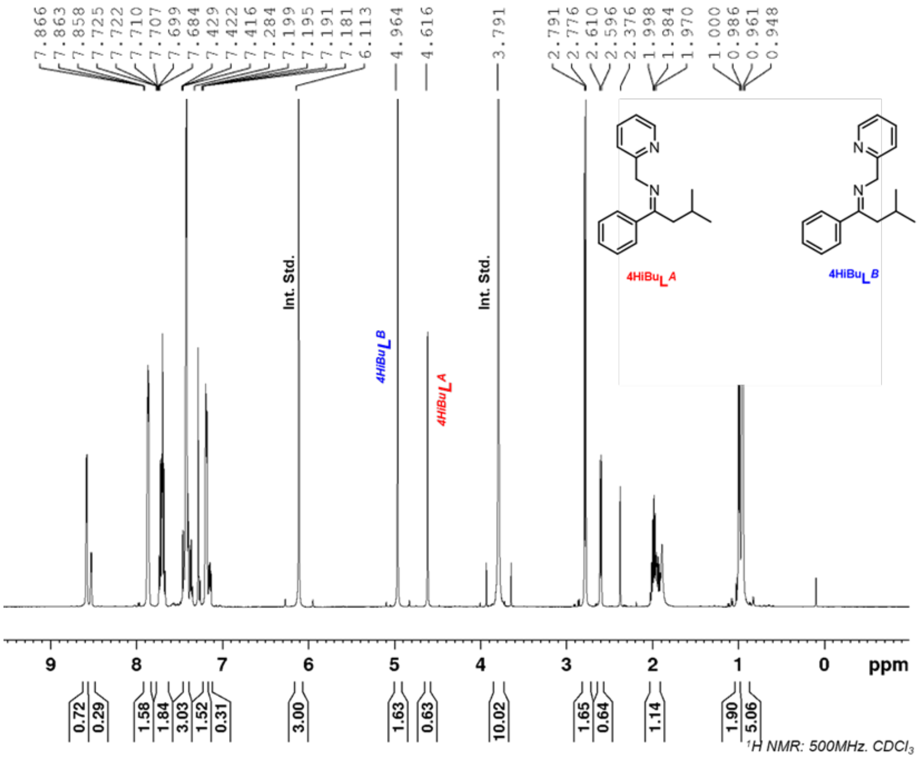

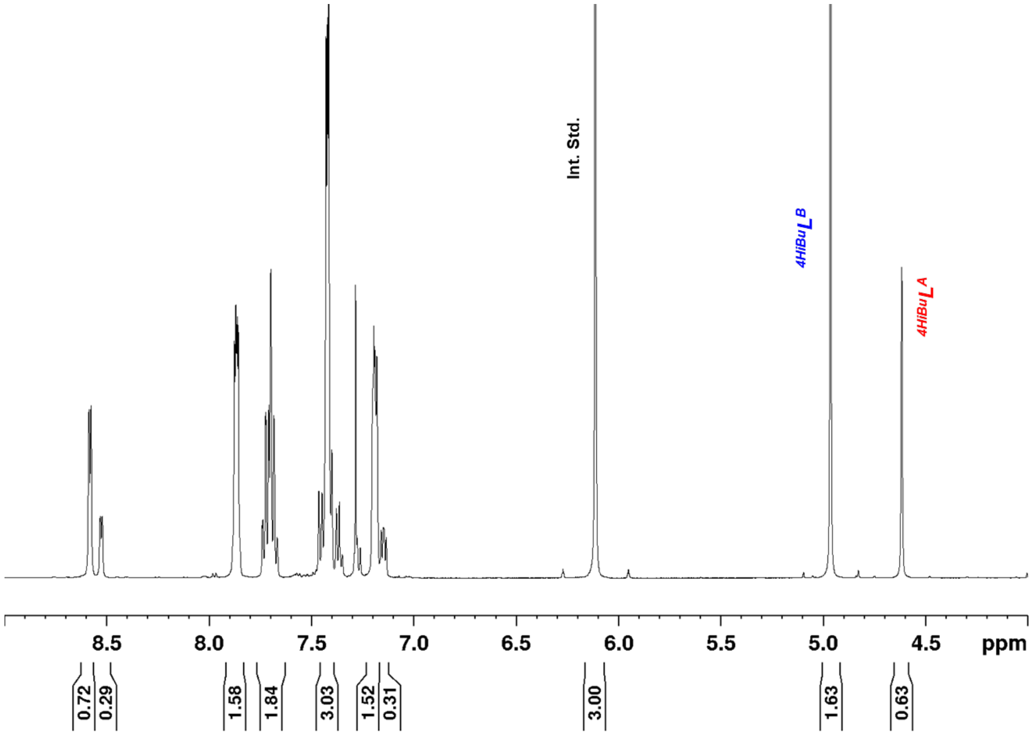

Figure S112. ¹H-NMR spectra of ^{4HiBut}L. Note: two imine isomers were formed. The ratio of ^{4HiBu}L^{A} *and* ^{4HiBu}L^{B} (20/80) is calculated using the average of the integration of CH_2 peaks and CH peaks.

^{1}H NMR spectra for the Hydroxylation of ^{4HiBu}L

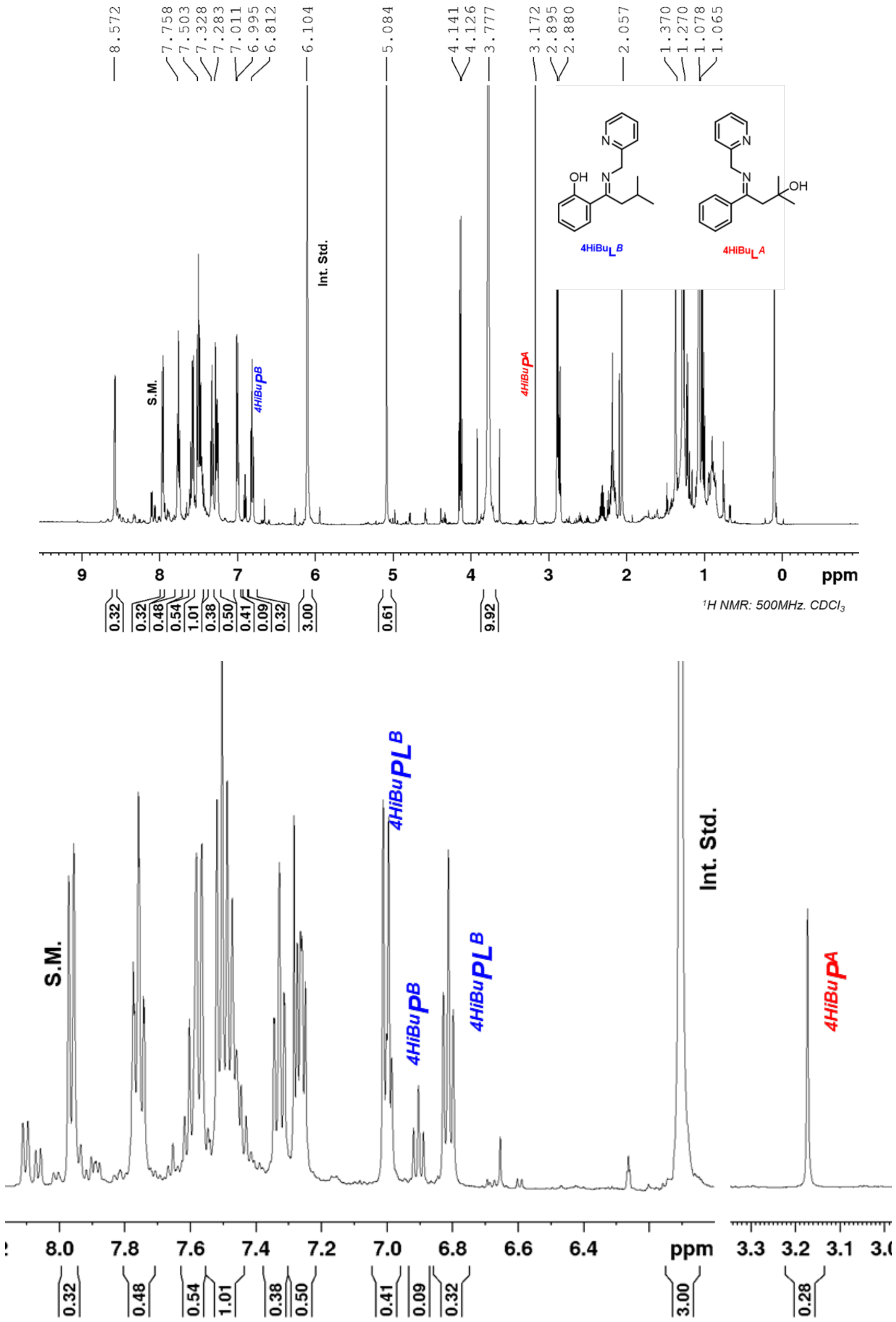

Figure S113. ^{1}H-NMR spectra for the hydroxylation of ^{4HiBu}L. Note: The ratio of ^{4HiBu}PL^{A} *and* ^{4HiBu}PL^{B} (25/75) is calculated using the average of the integration of CH_2 peaks and CH peaks.

3.40 ^{4HiBu}S and 2-(aminomethyl)-4-methoxypyridine

Synthesis of $^{4HiBu}L_{4MeO-Py}$

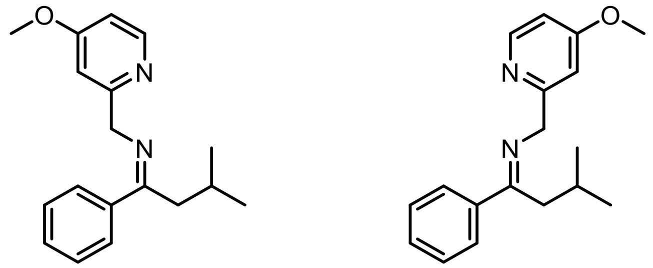

$^{4HiBu}L^{A}_{4MeO-Py}$ $^{4HiBu}L^{B}_{4MeO-Py}$

In an oven dried flask, 2-(aminomethyl)-4-methoxypyridine (2.2 equiv., 700 mg) was added to Isobutyl phenyl ketone (400 mg, 2.46 mmol) and p- toluenesulfonic acid monohydrate (cat. 20 mg, 4.7 mol%) in toluene (50 mL). The reaction mixture was refluxed under argon with a Dean-Stark apparatus until imine formation was complete (12 hours). The reaction was cooled to room temperature and diluted with diethyl ether (30 mL). The organic layer was washed with saturated ammonia chloride (20 mL x 2), saturated aqueous sodium bicarbonate (20 mL), brine (20 mL), and dried with magnesium sulfate. The final product was isolated as a brown solid (83% yield, 578 mg, 85% pure). ^{1}H-NMR (500 MHz, $CDCl_3$): δ 8.40 (d, 1H), 8.39 (d, 1H), 7.86 (m, 2H), 7.42 (d, 6H), 7.28 (d, 2H), 7.17 (d, 2H), 6.73 (d, 1H), 6.72 (d, 1H). 4.91 (s, 2H), 4.57 (s, 2H), 3.91 (s, 6H), 2.77 (d, 2H), 2.61 (d, 2H), 1.98 (m, 2H), 1.00 (d, 6H), 0.95 (d, 6H). 13C{1H} NMR (500 MHz, CD_3CN): δ 173.7, 170.3, 166.4, 162.8, 150.1, 140.5, 132.8, 108.1, 58.7, 57.4, 55.1, 51.3, 37.8, 27.2, 25.6, 22.8, 22.6. MS (ESI) m/z [M + H]+ calculated 282.3870, found 283.1765.

Hydroxylation of $^{4HiBu}L_{4MeO-Py}$

The reaction was carried out on 0.159 mmol scale using 53.0 mg of the imine according to the Standard Procedure. The reaction products were quantified using 0.159 mmol of 1,3,5-trimethoxybenzene (int. std.). (59% yield). The identity of the hydroxylation products was confirmed by ^{1}H-NMR.

^{1}H spectra of $^{4HiBu}L_{4MeO\text{-}Py}$

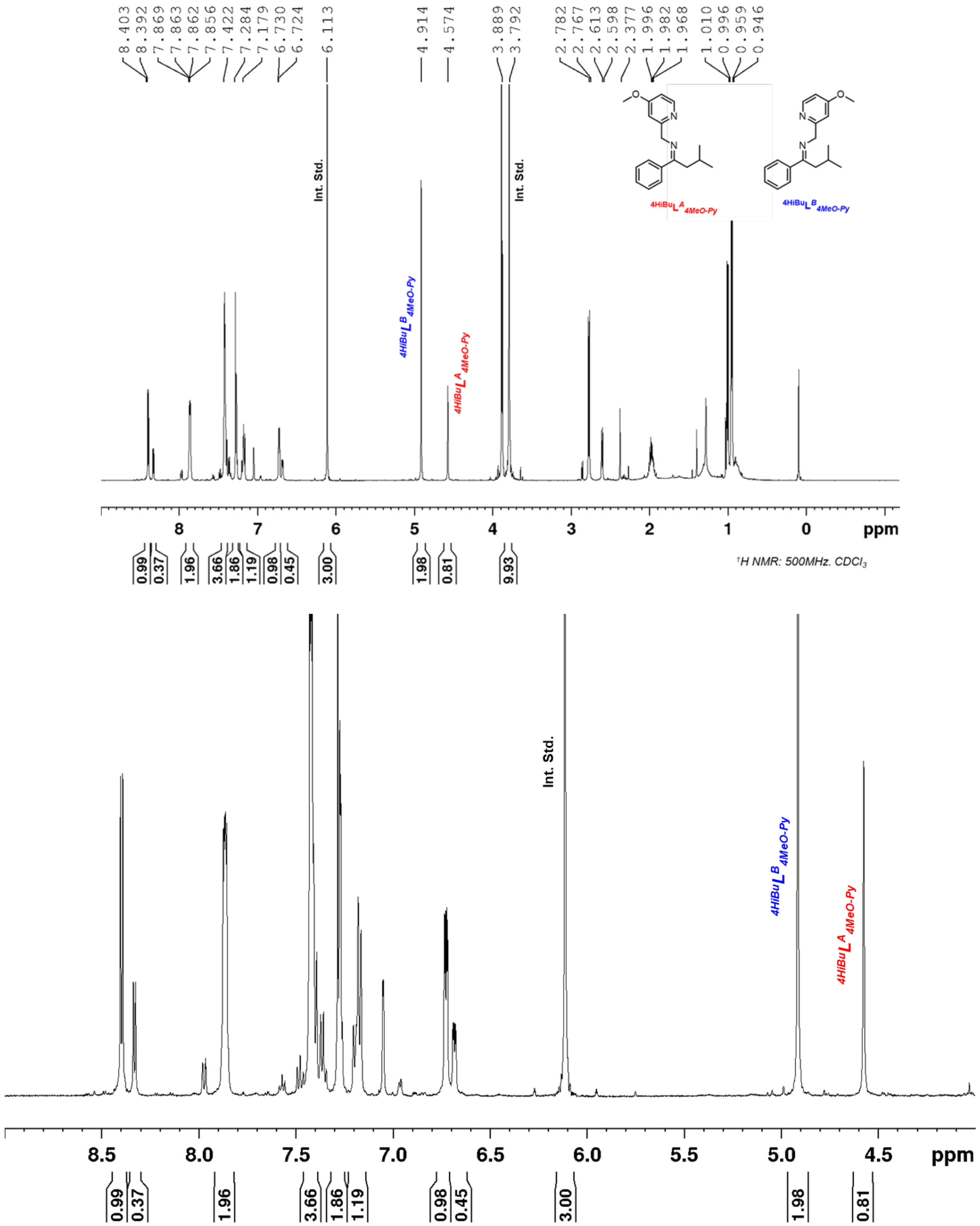

Figure S114. ^{1}H-NMR spectra of $^{4HiBu}L_{4MeO\text{-}Py}$. Note: two imine isomers were formed. The ratio of $^{4HiBu}L^{A}_{4MeO\text{-}Py}$ and $^{4HiBu}L^{B}_{4MeO\text{-}Py}$ (29/71) is calculated using the average of the integration of CH_2 peaks and CH peaks.

¹H spectra for the hydroxylation of $^{4HiBu}L_{4MeO\text{-}Py}$

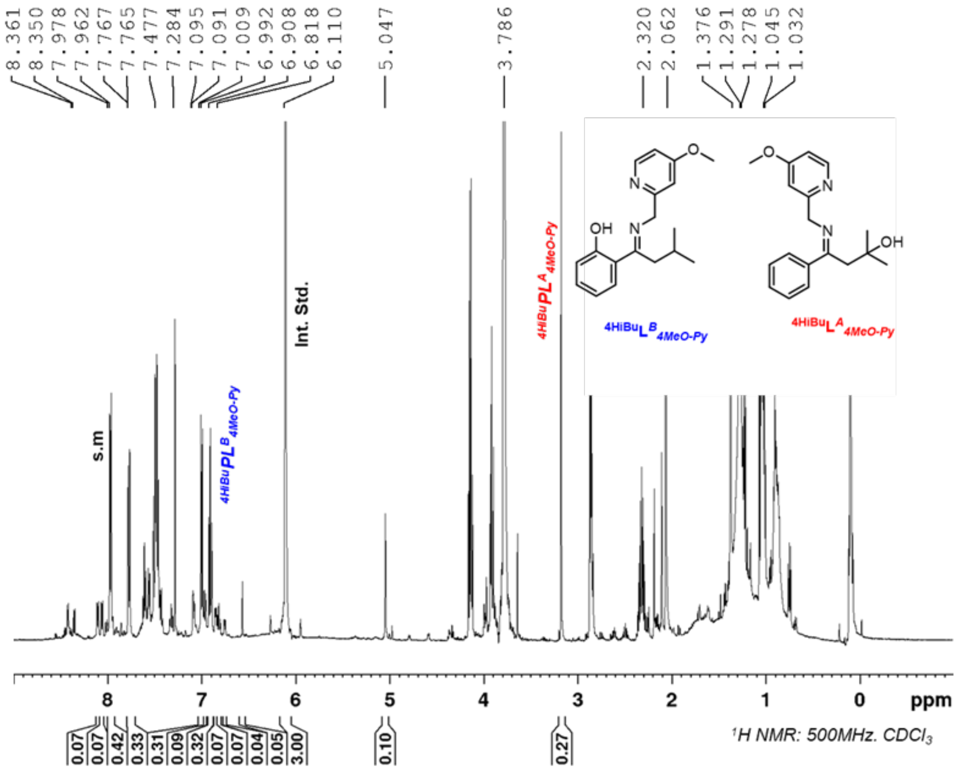

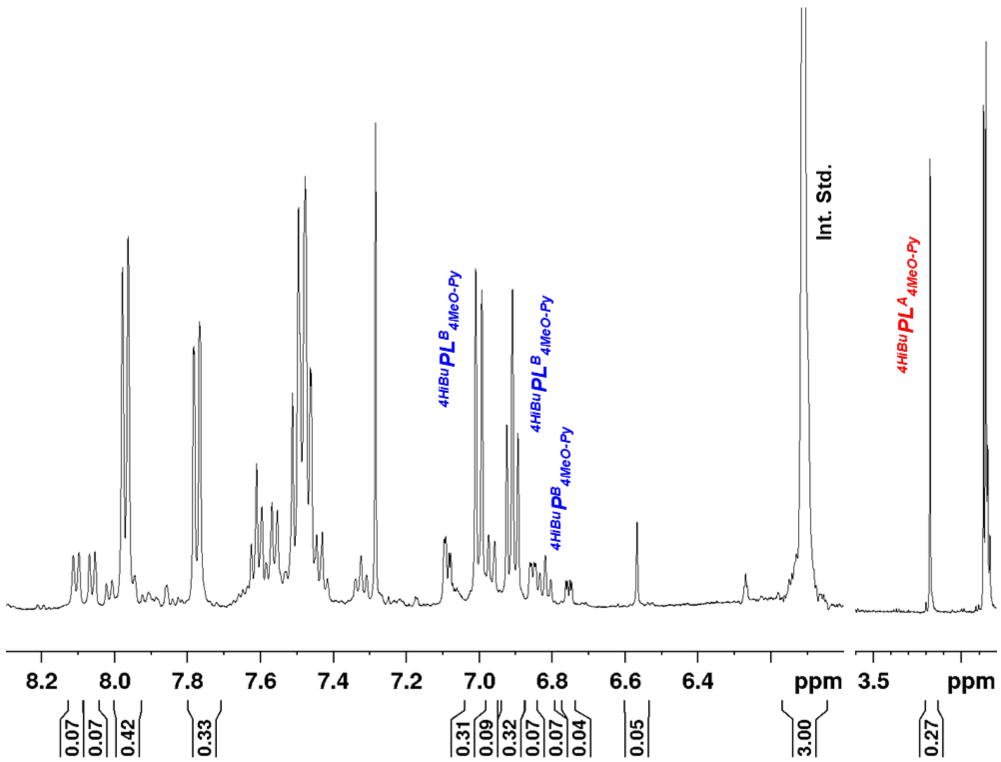

Figure S115. ^{1}H-NMR spectra for the hydroxylation of $^{4HiBu}L_{4MeO\text{-}Py}$. Note: The ratio of $^{4HiBu}PL^{A}_{4MeO\text{-}Py}$ and $^{4HiBu}PL^{B}_{4MeO\text{-}Py}$ (22/78) is calculated using the average of the integration of CH_2 peaks and CH peaks.

3.41 ^{4HiBu}S and 2-(aminomethyl)-4-chloropyridine

Synthesis of $^{4HiBu}\mathbf{L}_{4Cl\text{-}Py}$

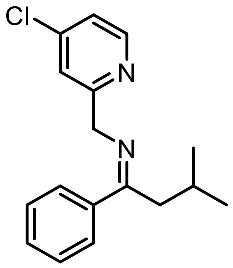

$^{4HiBu}\mathbf{L}^{A}_{4Cl\text{-}Py}$

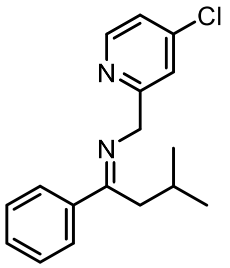

$^{4HiBu}\mathbf{L}^{B}_{4Cl\text{-}Py}$

In an oven dried flask, 2-picolylamine (2.2 equiv., 2.2 mL) was added to Isobutyl phenyl ketone (1.3 g, 9.85 mmol) and p- toluenesulfonic acid monohydrate (cat. 20 mg, 1.2 mol%) in toluene (50 mL). The reaction mixture was refluxed under argon with a Dean-Stark apparatus until imine formation was complete (12 hours). The reaction was cooled to room temperature and diluted with diethyl ether (30 mL). The organic layer was washed with saturated ammonia chloride (20 mL x 2), saturated aqueous sodium bicarbonate (20 mL), brine (20 mL), and dried with magnesium sulfate. The final product was isolated as a brown solid (89% yield, 2.5 g, 90% pure). ^{1}H-NMR (500 MHz, $CDCl_3$): δ 8.47 (d, 1H), 8.41(d, 1H), 7.87 (m, 3H), 7.76 (d, 1H), 7.44 (m, 6H), 7.20 (d, 2H), 7.15 (d, 2H), 4.92(s, 2H), 4.58 (s, 2H), 2.76(d, 2H), 2.59 (d, 2H), 1.98 (m, 2H), 0.96 (m, 12H) 13C{1H} NMR (500 MHz, CD_3CN): δ 170.7, 162.8, 149.8, 144.9, 140.2, 129.7, 128.3, 127.1, 122.4, 57.0, 51.5, 37.9, 27.2, 22.5. MS (ESI) m/z [M + H]+ calculated 286.8030, found 287.2311.

Hydroxylation of $^{4HiBu}\mathbf{L}_{4Cl\text{-}Py}$

The reaction was carried out on 0.159 mmol scale using 50.0 mg of the imine according to the Standard Procedure. The reaction products were quantified using 0.159 mmol of 1,3,5-trimethoxybenzene (int. std.). (59% yield). The identity of the hydroxylation products was confirmed by ^{1}H-NMR.

^{1}H NMR of $^{4HiBu}L_{4Cl-Py}$

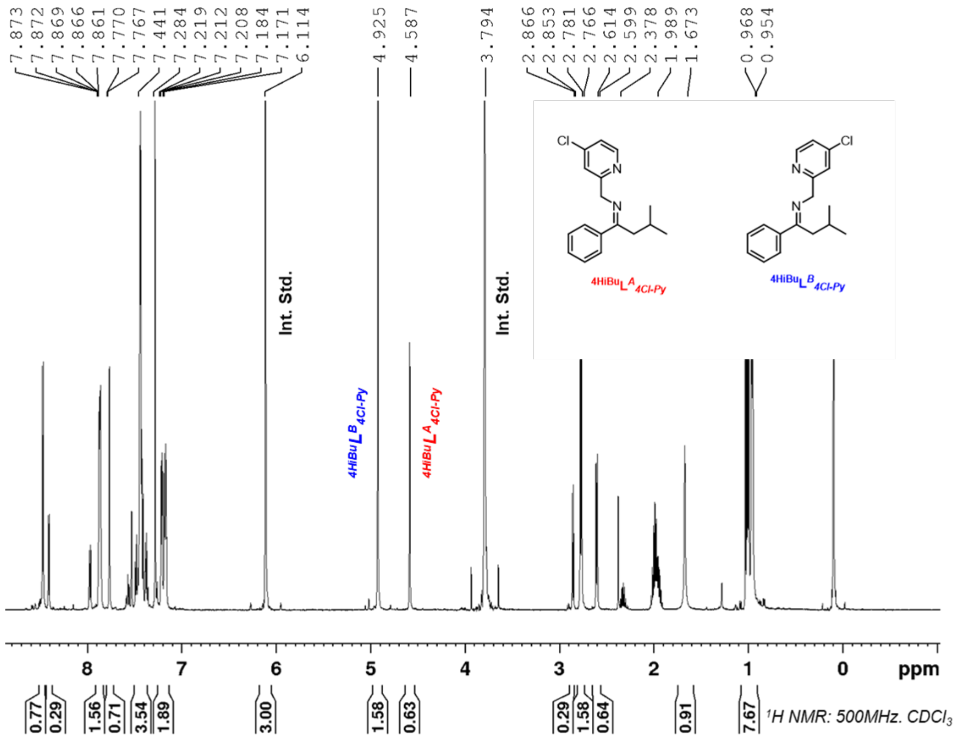

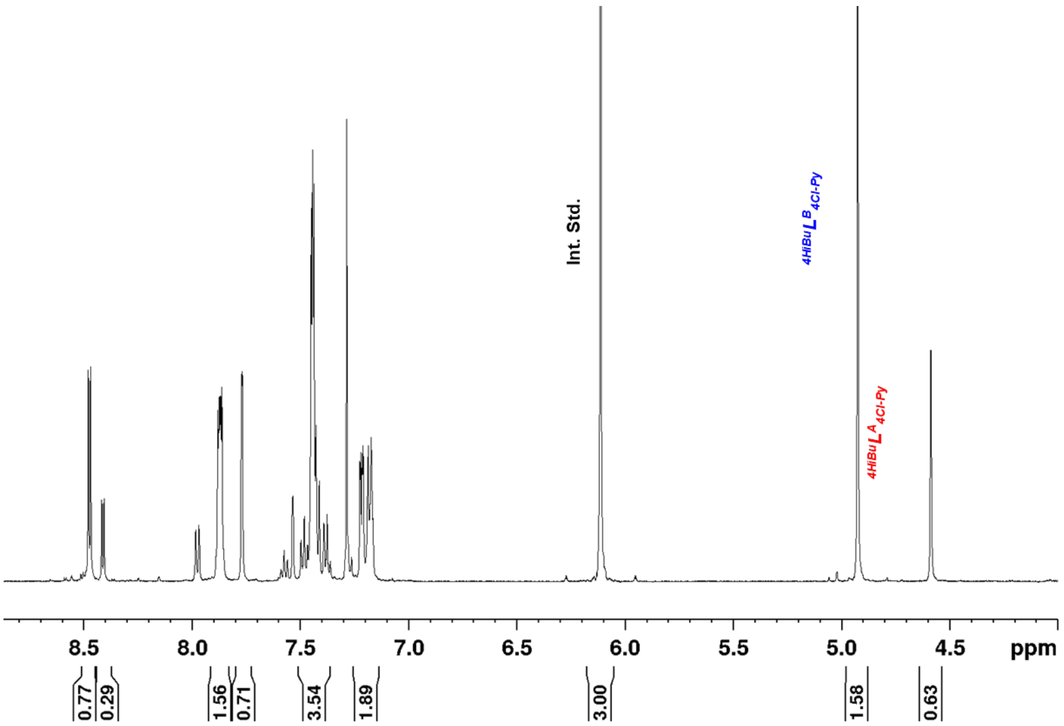

Figure S116. ^{1}H-NMR spectra of ^{4MeOEt}L. Note: two imine isomers were formed. The ratio of $^{4HiBu}L^{A}_{4Cl-Py}$ and $^{4HiBu}L^{B}_{4Cl-Py}$ (28/72) is calculated using the average of the integration of CH_2 peaks and CH peaks.

^{1}*H NMR for the hydroxylation of* ^{4HiBu}*L*$_{4Cl\text{-}Py}$

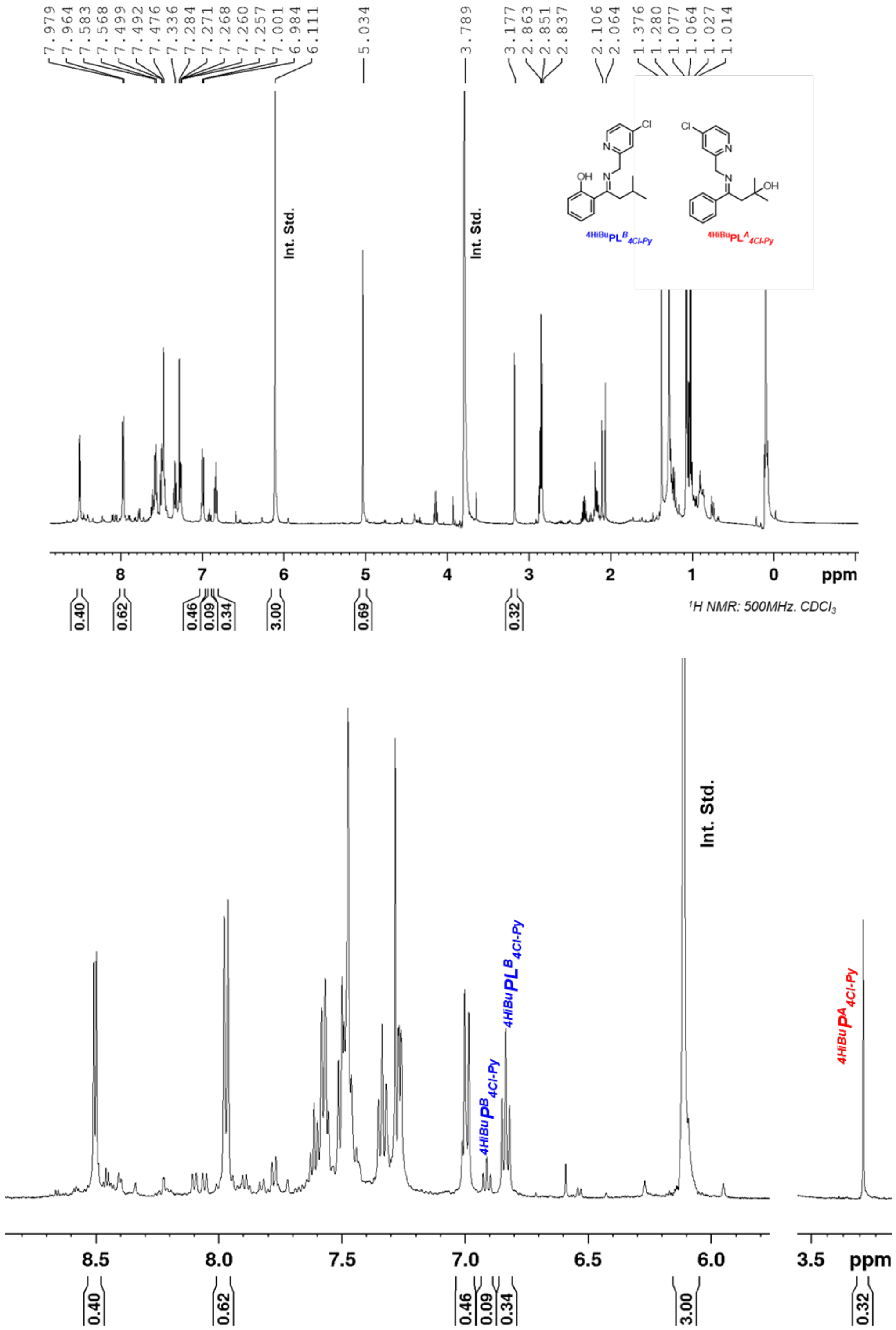

Figure S117. ^{1}H-NMR spectra for the hydroxylation of ^{4HiBu}L$_{4Cl\text{-}Py}$. Note: The ratio of ^{4HiBu}PL$^{A}_{4Cl\text{-}Py}$ and ^{4HiBu}P$L^{B}_{4Cl\text{-}Py}$ (27/73) is calculated using the average of the integration of CH_2 peaks and CH peaks.

3.42 $^{4HCy(n)}$S and 2-picolylamine

Synthesis of $^{4HCy(6, Me)}$L

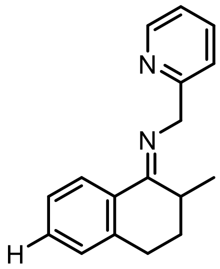

4HCy(6,Me)L *B*

In an oven dried flask, 2-picolylamine (2.2 equiv., 2.2 mL) was added to propiophenone (1.3 g, 9.85 mmol) and p- toluenesulfonic acid monohydrate (cat. 20 mg, 1.2 mol%) in toluene (50 mL). The reaction mixture was refluxed under argon with a Dean-Stark apparatus until imine formation was complete (12 hours). The reaction was cooled to room temperature and diluted with diethyl ether (30 mL). The organic layer was washed with saturated ammonia chloride (20 mL x 2), saturated aqueous sodium bicarbonate (20 mL), brine (20 mL), and dried with magnesium sulfate. The final product was isolated as a brown solid (90% yield, 2.22 g, 97% pure). ^{1}H-NMR (500 MHz, $CDCl_3$): δ 8.57 (d, 1H), 8.38 (d, 1H), 7.71 (d, 2H), 7.35 (t, 1H), 7.28 (t, 1H), 7.19 (t, 2H). 5.00 (q, 2H), 3.39 (m, 1H), 2.77 (d, 1H), 2.10 (m, 1H), 1.77 (m, 1H), 1.30 (d, 1H), 1.18 (d, 3H). 13C{1H} NMR (500 MHz, CD_3CN): δ 169.4, 161.1, 148.9, 136.6, 129.8, 128.6, 126.4, 121.9, 121.6, 55.8, 29,8, 28.7, 24.4, 14.8.

Note: the synthesis of this substrate-ligand was carried out to confirm the selective formation of the isomer B. The hydroxylation of this system was previously reported by our group.[1]

^{1}H-NMR spectra of [4HCy(6, Me)]L

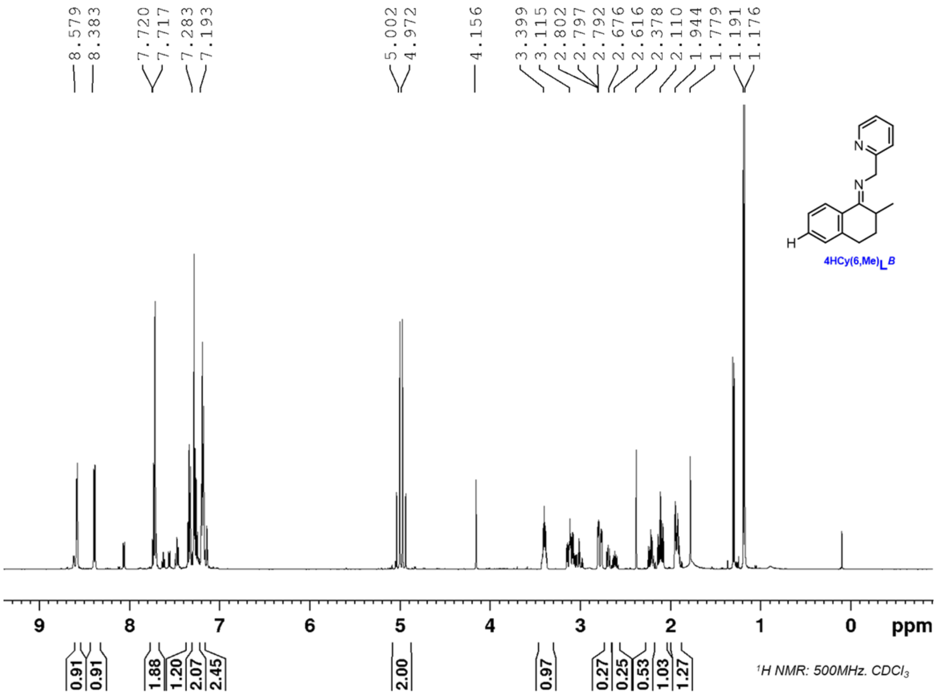

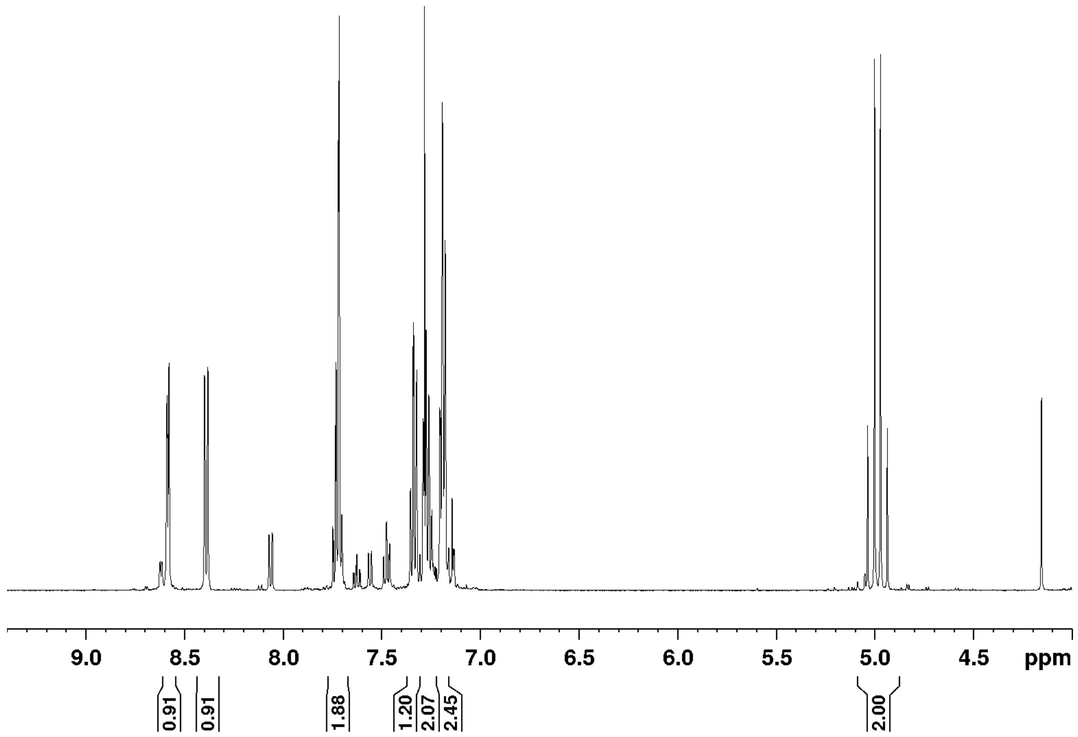

Figure S118. ^{1}H-NMR spectra of $^{4HCy(6,\ Me)}$L. Note: Only one of the imine isomers is formed.

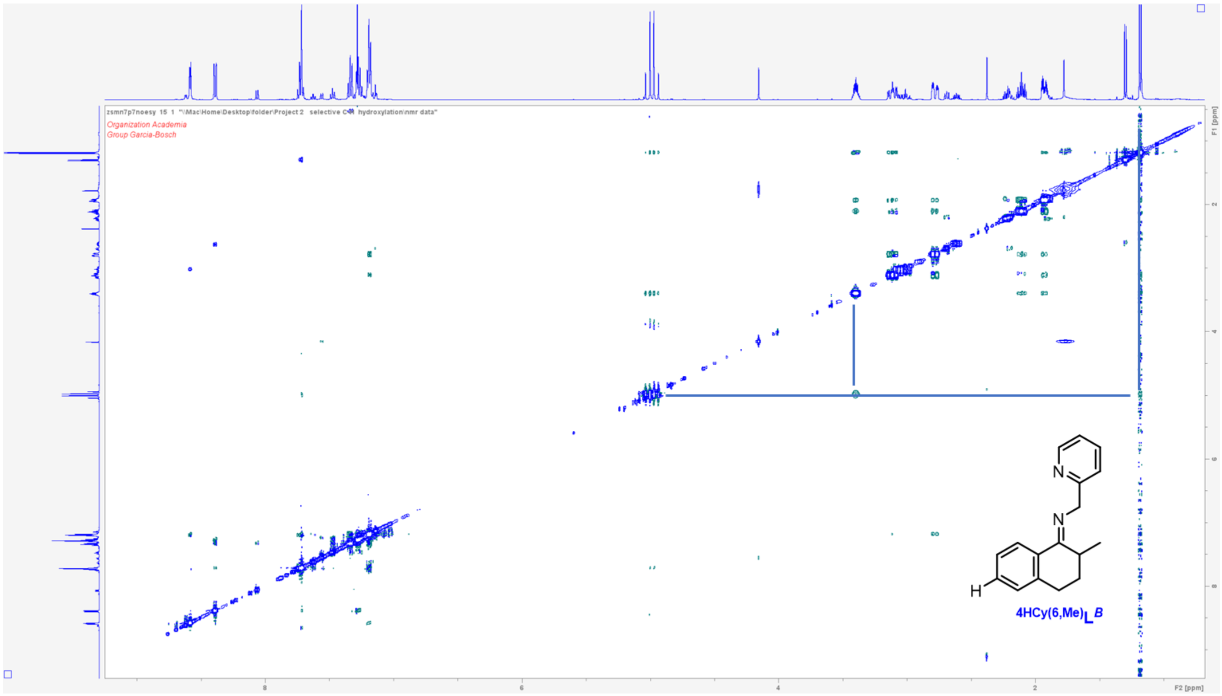

Figure S119. Nuclear Overhauser Effect Spectroscopy (NOESY) spectra for $^{4HCy(6,\ Me)}$L

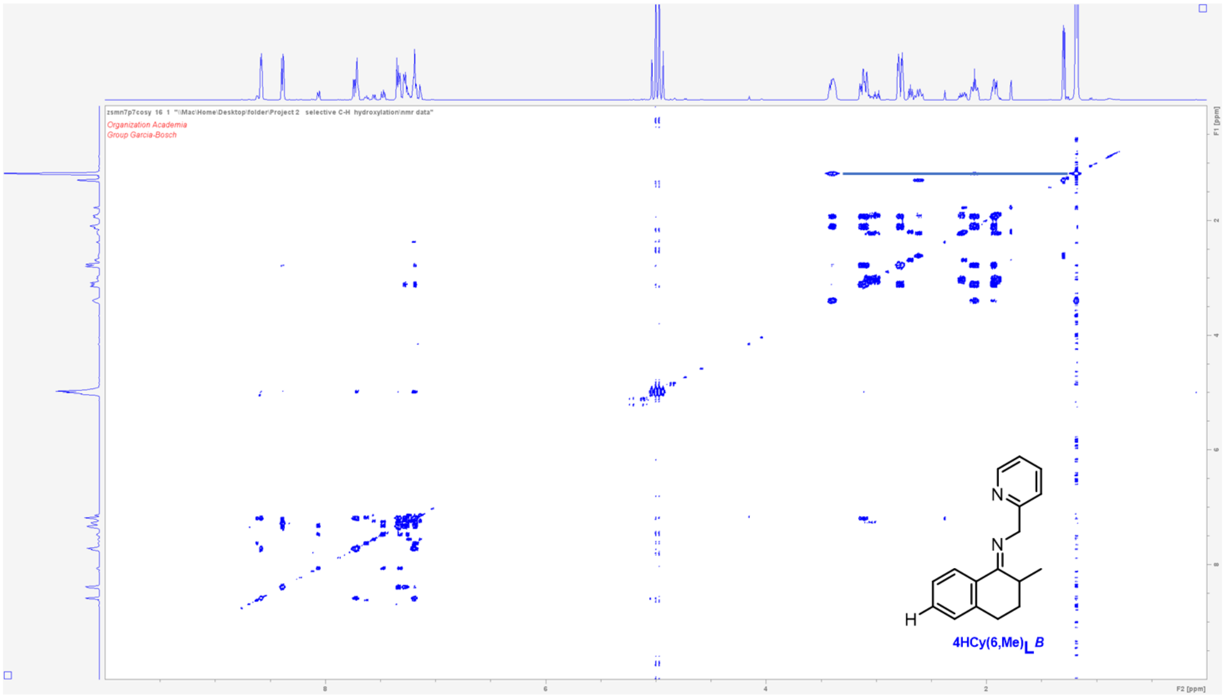

Figure S120. Homonuclear correlation spectroscopy (COSY) spectra of $^{4HCy(6,\ Me)}$L

3.43 $^{4HCy(6)}$S and 2-picolylamine

Synthesis of $^{4HCy(6)}$L

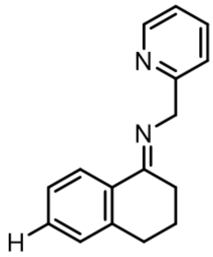

$^{4HCy(6)}$L*B*

In an oven dried flask, 2-picolylamine (2.2 equiv., 2.2 mL) was added to propiophenone (1.3 g, 9.85 mmol) and p- toluenesulfonic acid monohydrate (cat. 20 mg, 1.2 mol%) in toluene (50 mL). The reaction mixture was refluxed under argon with a Dean-Stark apparatus until imine formation was complete (12 hours). The reaction was cooled to room temperature and diluted with diethyl ether (30 mL). The organic layer was washed with saturated ammonia chloride (20 mL x 2), saturated aqueous sodium bicarbonate (20 mL), brine (20 mL), and dried with magnesium sulfate. The final product was isolated as a brown solid (91% yield, 2.12 g, 93% pure). ^{1}H-NMR (500 MHz, $CDCl_3$): δ 8.59 (d, 1H), 8.35 (d, 1H), 7.75 (m, 2H), 7.33 (t, 1H), 7.28 (t, 2H), 7.19 (t, 1H), 4.85 (s, 2H), 2.86 (t, 2H), 2.72 (t, 2H), 2.02 (m, 2H). 13C{1H} NMR (500 MHz, CD_3CN): δ 166.0, 161.1, 148.9, 140.8, 134.7, 129.9, 128.4, 126.4, 125.8, 122.0, 121.6, 56.6, 29.8, 28.6, 22.5. MS (ESI) m/z [M + H]+ calculated 236.3180, found 237.1347.

Hydroxylation of $^{4HCy(6)}$L

The reaction was carried out on 0.159 mmol scale using 41.0 mg of the imine according to the Standard Procedure. The reaction products were quantified using 0.159 mmol of 1,3,5-trimethoxybenzene (int. std.). (56% yield). The identity of the hydroxylation products was confirmed by ^{1}H-NMR.

^{1}H-NMR spectra of $^{4HCy(6)}$L

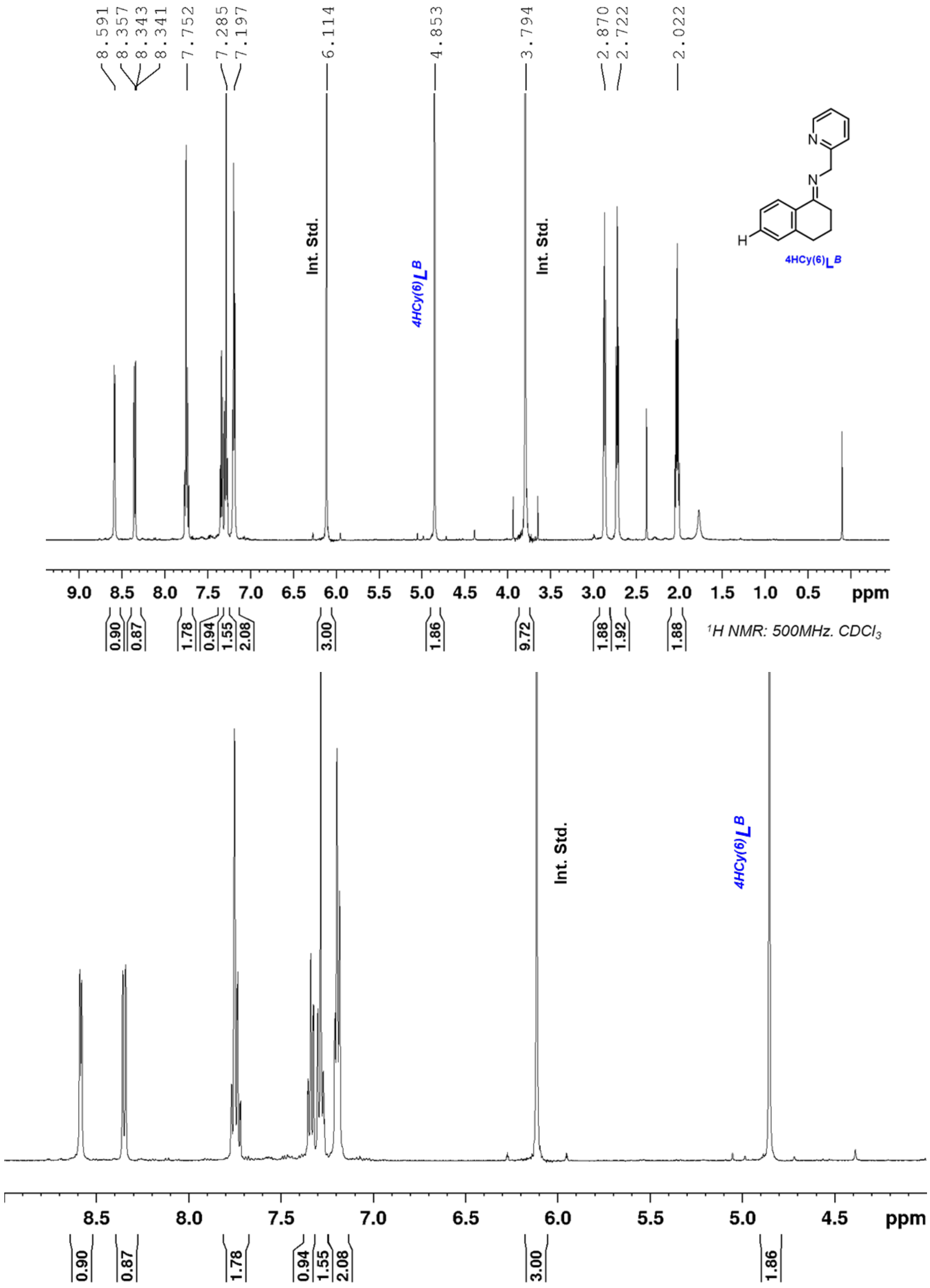

Figure S121. ^{1}H-NMR spectra of $^{4HCy(6)}$L. Note: Only one of the imine isomers is formed.

^{1}H-NMR spectra for the hydroxylation of $^{4HCy(6)}$L

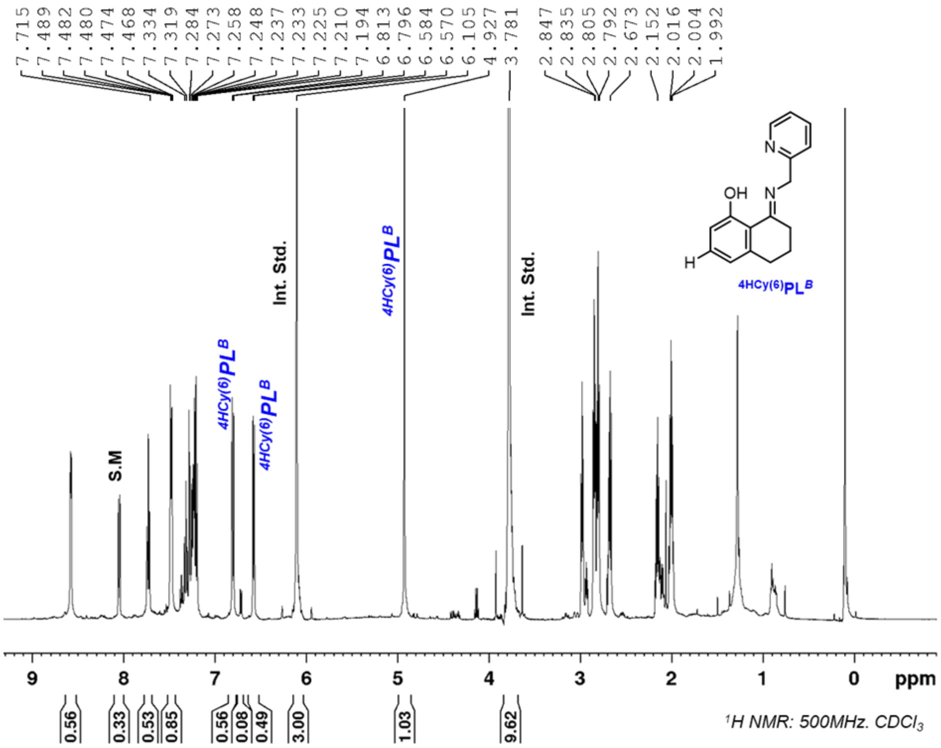

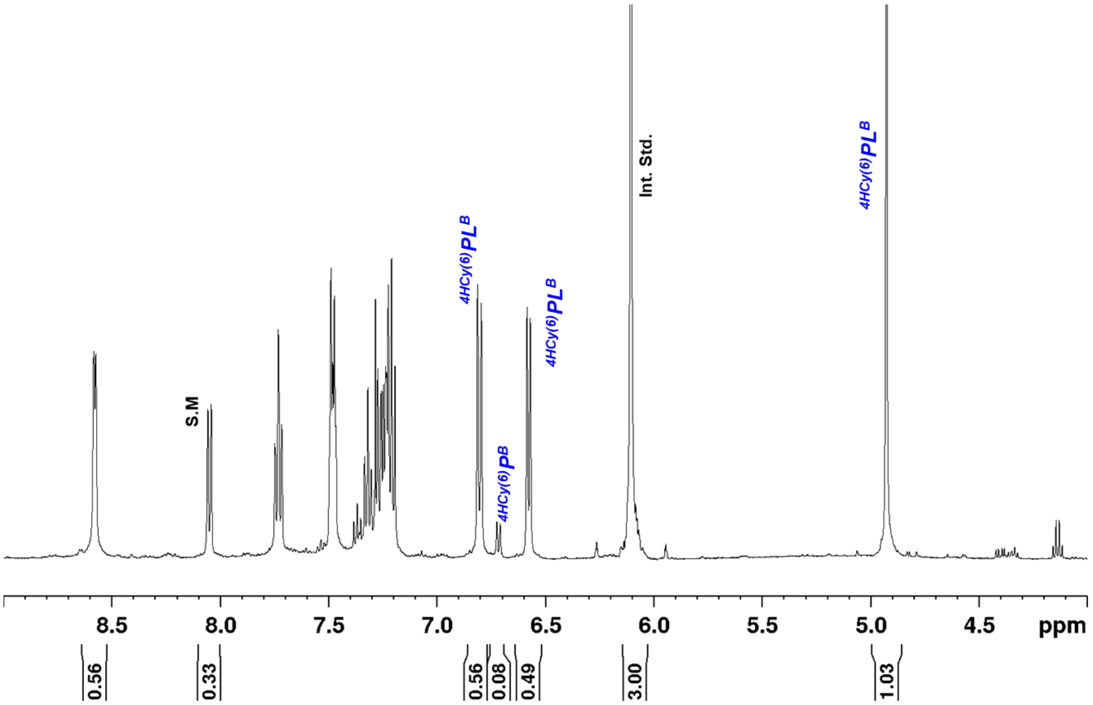

Figure S122. ^{1}H-NMR spectra for the hydroxylation of $^{4HCy(6)}$L.

3.44 $^{4MeOCy(6)}$S and 2-picolylamine

Synthesis of $^{4MeOCy(6)}$L

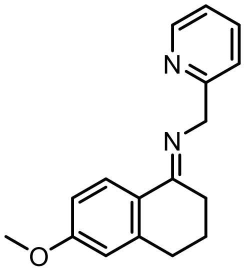

4MeOCy(6)L*B*

In an oven dried flask, 2-picolylamine (2.2 equiv., 2.2 mL) was added to 6-Methoxy-1-tetralone (1.75 g, 9.85 mmol) and p- toluenesulfonic acid monohydrate (cat. 20 mg, 1.2 mol%) in toluene (50 mL). The reaction mixture was refluxed under argon with a Dean-Stark apparatus until imine formation was complete (12 hours). The reaction was cooled to room temperature and diluted with diethyl ether (30 mL). The organic layer was washed with saturated ammonia chloride (20 mL x 2), saturated aqueous sodium bicarbonate (20 mL), brine (20 mL), and dried with magnesium sulfate. The final product was isolated as a brown solid (94% yield, 2.47 g, 96% pure). ^{1}H-NMR (500 MHz, $CDCl_3$): δ 8.59 (d, 1H), 8.30 (d, 1H), 7.72 (d, 2H), 7.16 (d, 1H), 6.81 (d, 1H), 6.67 (d, 1H), 4.82 (s, 2H), 2.80 (t, 2H) , 2.65 (t, 2H), 2.0 (t, 2H). 13C{1H} NMR (500 MHz, CD_3CN): δ 148.9, 136.5, 127.7, 121.9, 121.5, 112.9, 56.5, 55.2, 30.1, 28.3, 22.6. MS (ESI) m/z [M + H]+ calculated 266.3440, found 267.1653.

Hydroxylation of $^{4MeOCy(6)}$L

The reaction was carried out on 0.159 mmol scale using 44.0 mg of the imine according to the Standard Procedure. The reaction products were quantified using 0.159 mmol of 1,3,5-trimethoxybenzene (int. std.). (44% yield). The identity of the hydroxylation products was confirmed by ^{1}H-NMR.

Cleavage of $^{4MeOCy(6)}$PL

Dissolving $^{4MeO}PL_{4MeO\text{-}py}$ in round bottom flask with 50 mL EtOAc, then adding 100 mL 1M HCl. Reaction was going for 30 min. The resulting mixture was extracted with EtOAc (50 mL X 2). The organic phases were separated, combined, dried over $MgSO_4$, filtered, and dried under vacuum. The reaction products were dissolved in 1.4 mL of $CDCl_3$ solution containing 13.5 mg of 1,3,5-trimethoxybenzene (internal standard). The reaction products were quantified by ^{1}H-NMR using integration signals that correspond to the starting material and products with the integration signal of the internal standard.

^{1}H-NMR spectra of $^{4MeOCy(6)}$L

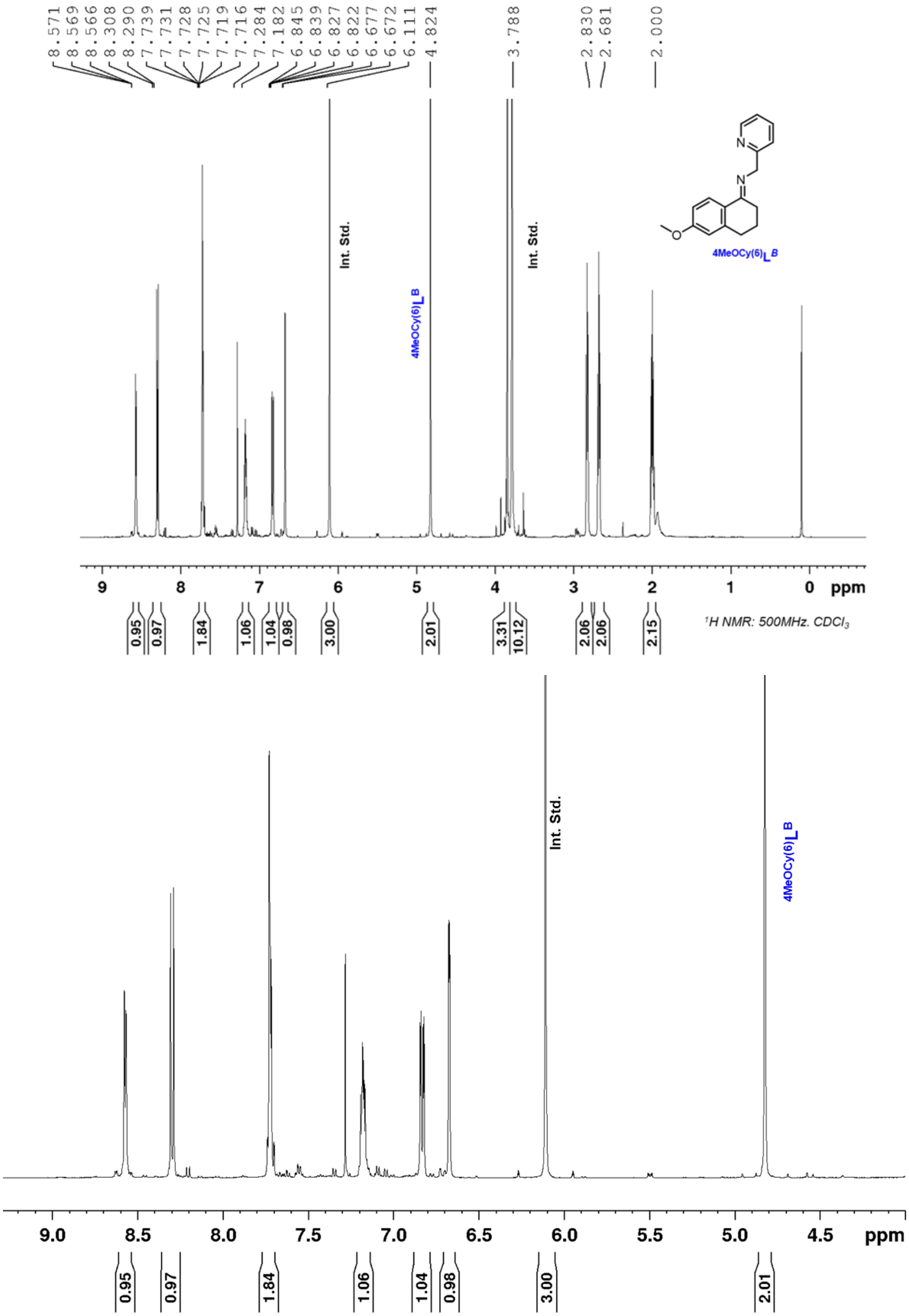

Figure S123. ^{1}H-NMR spectra of $^{4MeOCy(6)}$L. Note: Only one of the imine isomers is formed.

^{1}H-NMR spectra for the hydroxylation of $^{4MeOCy(6)}$L

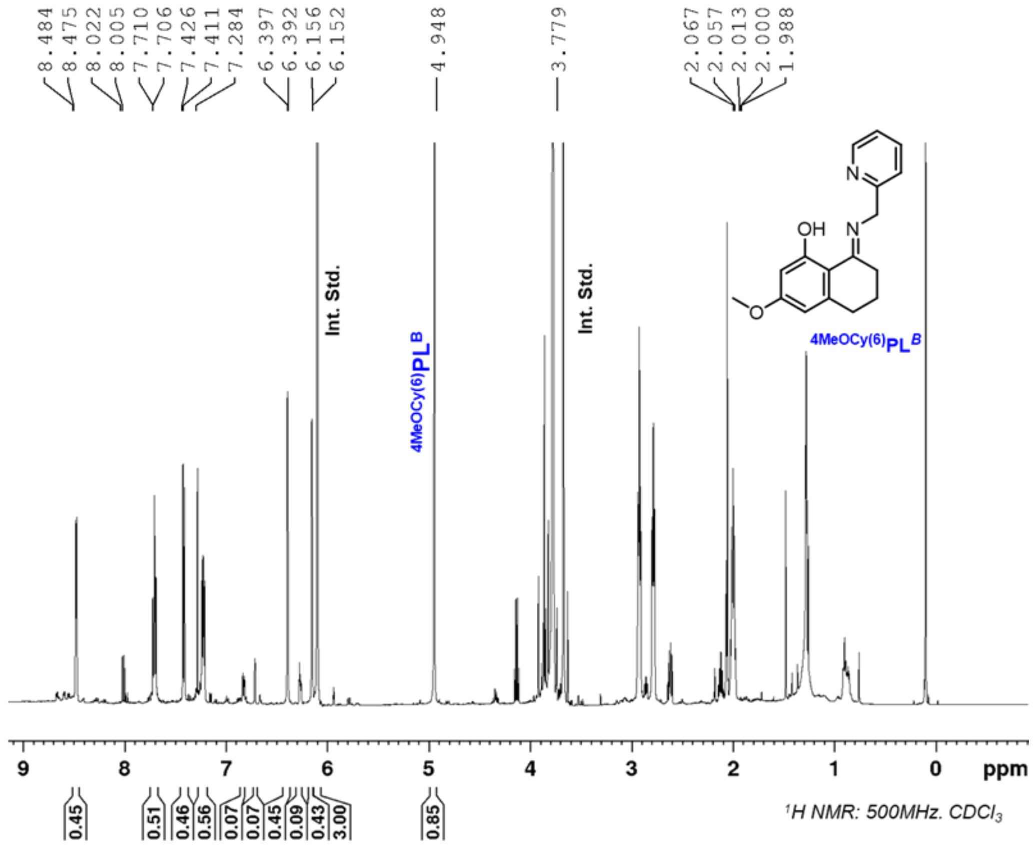

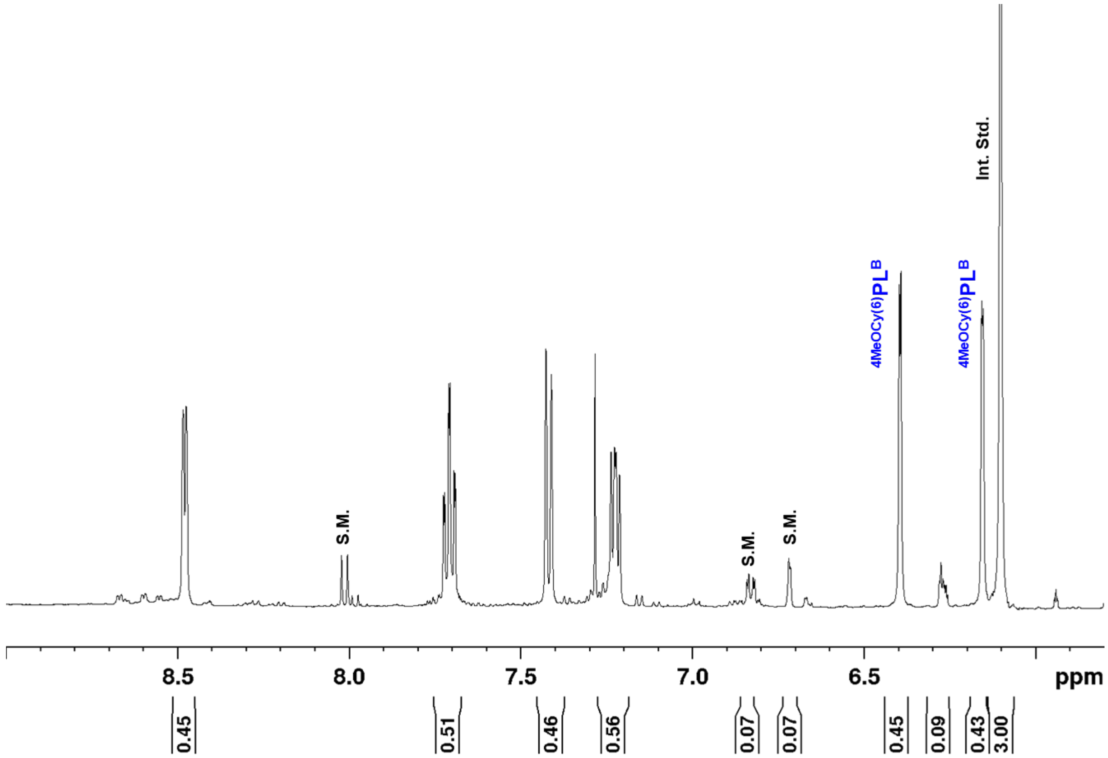

Figure S124. ^{1}H-NMR spectra for the hydroxylation of $^{4MeOCy(6)}$L.

^{1}H-NMR spectra for the cleavage of [4MeOCy(6)]PL

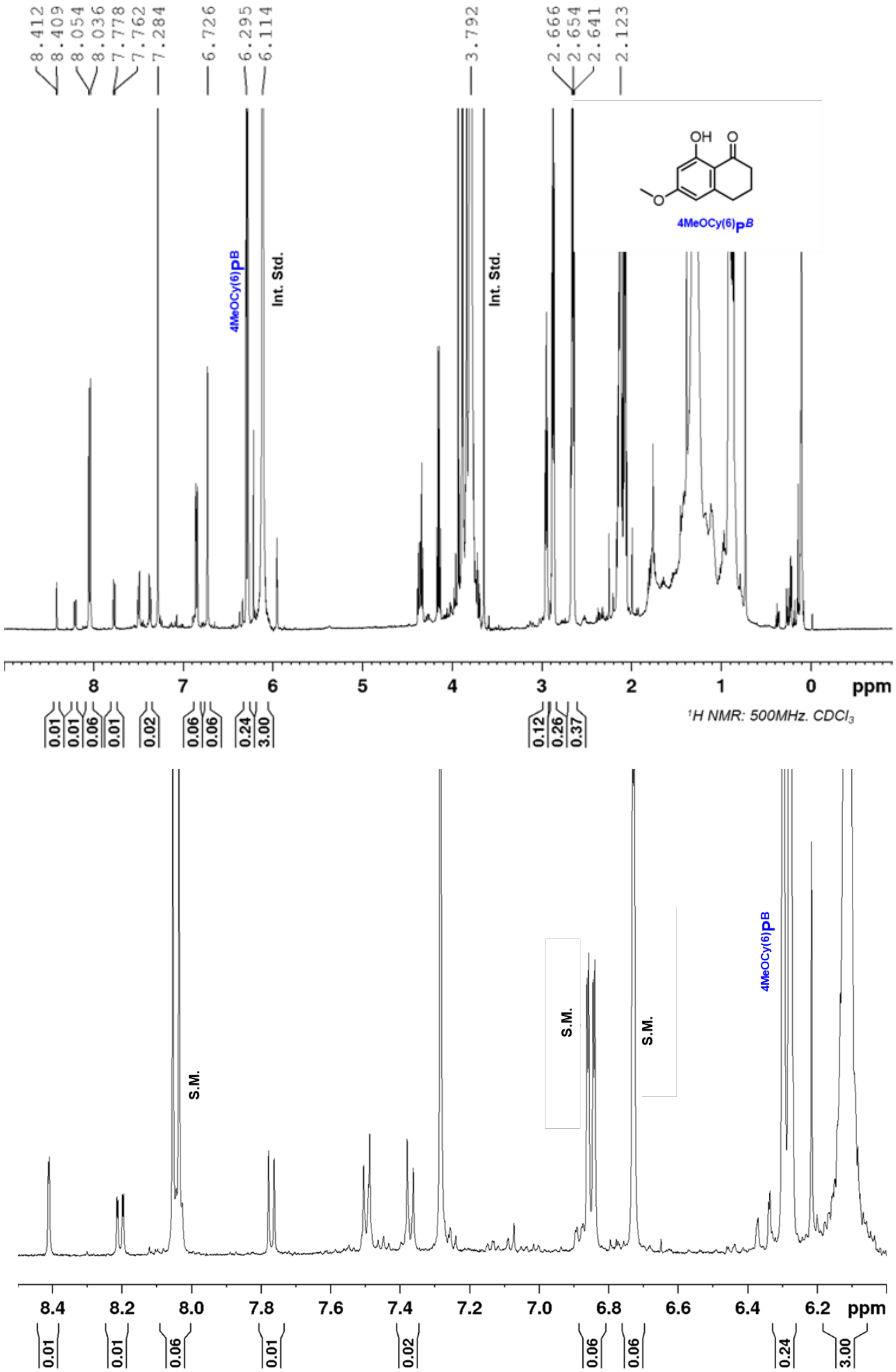

Figure S125. ^{1}H-NMR spectra for the hydroxylation of $^{4MeOCy(6)}$L.

3.45 ${}^{4FCy(6)}$S and 2-picolylamine

Synthesis of ${}^{4FCy(6)}$L

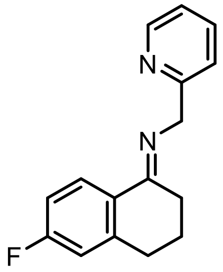

${}^{4FCy(6)}$L *B*

In an oven dried flask, 2-picolylamine (2.2 equiv., 2.2 mL) was added to 6-Fluoro-1-tetralone (1.6 g, 9.85 mmol) and p- toluenesulfonic acid monohydrate (cat. 20 mg, 1.2 mol%) in toluene (50 mL). The reaction mixture was refluxed under argon with a Dean-Stark apparatus until imine formation was complete (12 hours). The reaction was cooled to room temperature and diluted with diethyl ether (30 mL). The organic layer was washed with saturated ammonia chloride (20 mL x 2), saturated aqueous sodium bicarbonate (20 mL), brine (20 mL), and dried with magnesium sulfate. The final product was isolated as a brown solid (80% yield, 2.01 g, 92% pure). ^{1}H-NMR (500 MHz, $CDCl_3$): δ 8.57 (d, 1H), 8.35 (t, 1H), 7.72 (m, 2H), 7.28 (t, 1H), 6.96 (t, 1H), 6.86 (d, 1H), 4.82 (s, 2H), 2.84 (t, 2H), 2.70 (t, 2H), 2.01 (t, 2H). 13C{1H} NMR (500 MHz, CD_3CN): δ 164.9, 164.7, 162.91, 148.9, 143.0, 136.6, 128.4, 121.6, 113.6, 56.6, 29.8, 28.2, 22.4. MS (ESI) m/z [M + H]+ calculated 254.3084, found 255.1288.

Hydroxylation of ${}^{4FCy(6)}$L

The reaction was carried out on 0.159 mmol scale using 44.0 mg of the imine according to the Standard Procedure. The reaction products were quantified using 0.159 mmol of 1,3,5-trimethoxybenzene (int. std.). (53% yield). The identity of the hydroxylation products was confirmed by ^{1}H-NMR.

Cleavage of ${}^{4FCy(6)}$PL

Dissolving *${}^{4FCy(6)}$PL* in round bottom flask with 50 mL EtOAc, then adding 100 mL 1M HCl. Reaction was going for 30 min. The resulting mixture was extracted with EtOAc (50 mL X 2). The organic phases were separated, combined, dried over $MgSO_4$, filtered, and dried under vacuum. The reaction products were dissolved in 1.4 mL of $CDCl_3$ solution containing 13.5 mg of 1,3,5-trimethoxybenzene (internal standard). The reaction products were quantified by ^{1}H-NMR using integration signals that correspond to the starting material and products with the integration signal of the internal standard.

^{1}H-NMR spectra of $^{4FCy(6)}$L

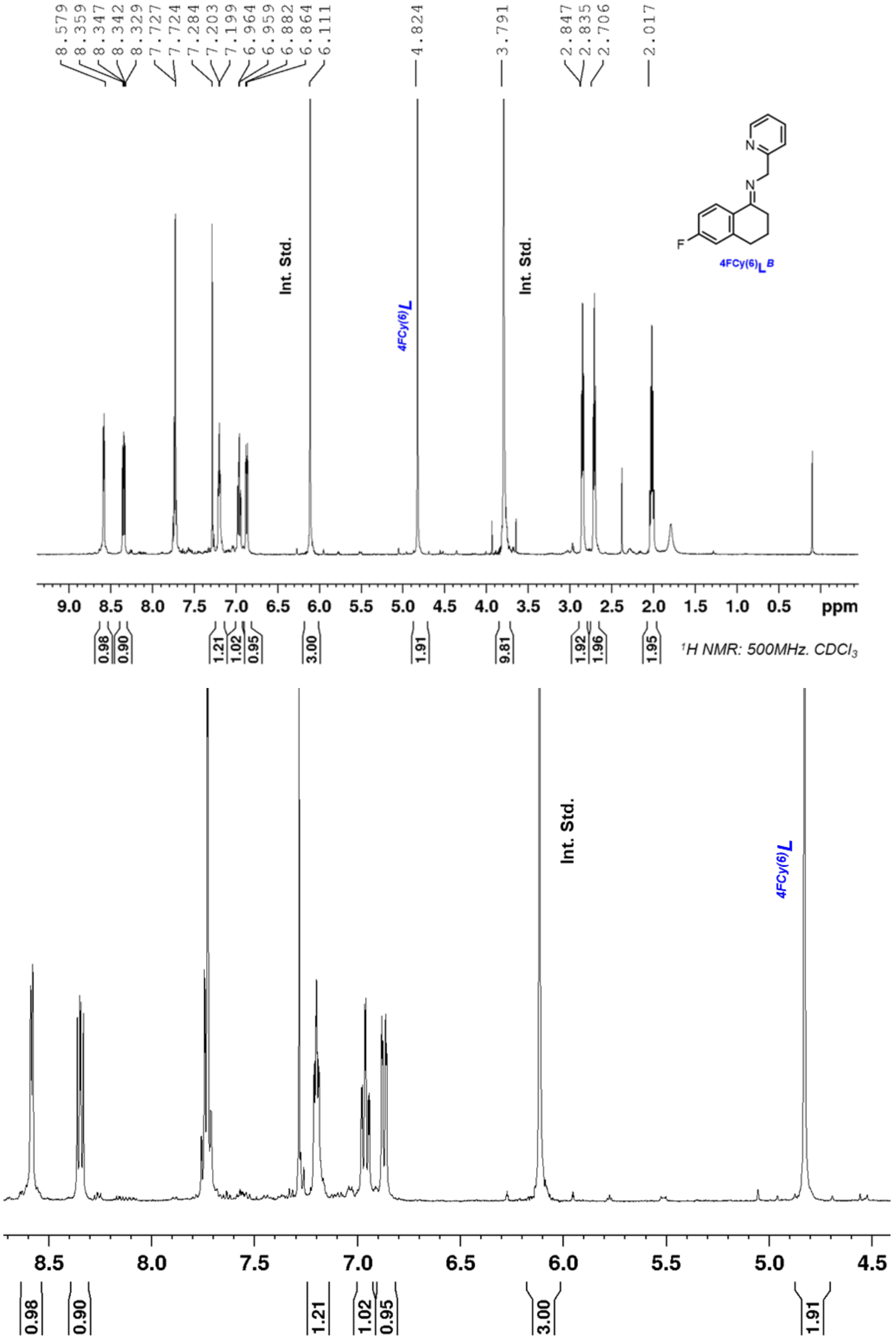

Figure S126. ^{1}H-NMR spectra of $^{4FCy(6)}$L. Note: Only one of the imine isomers is formed.

^{1}H-NMR spectra for the hydroxylation of $^{4FCy(6)}$L

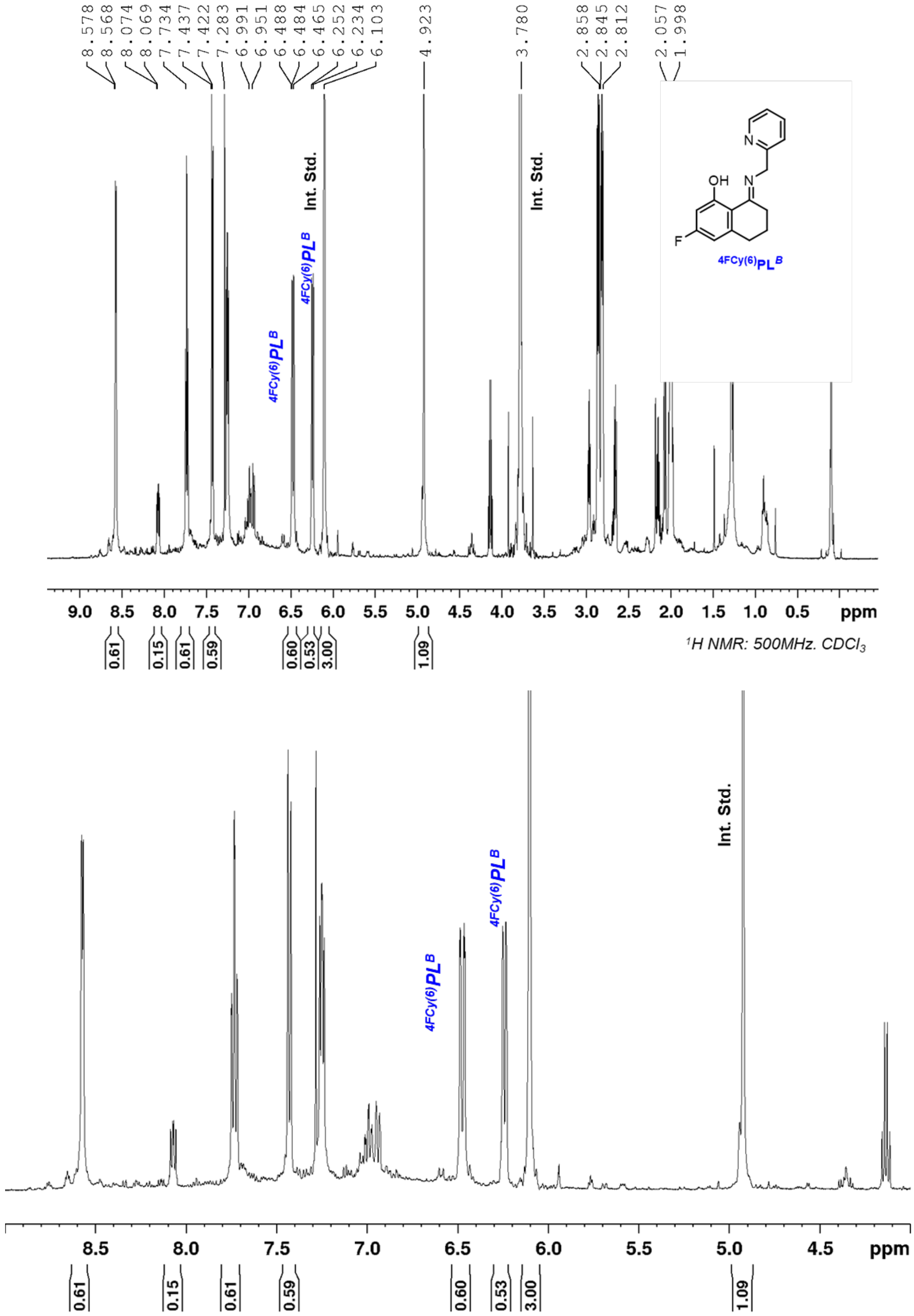

Figure S127. ^{1}H-NMR spectra for the hydroxylation of $^{4FCy(6)}$L.

^{1}H-NMR spectra for the cleavage of $^{4FCy(6)}$PL

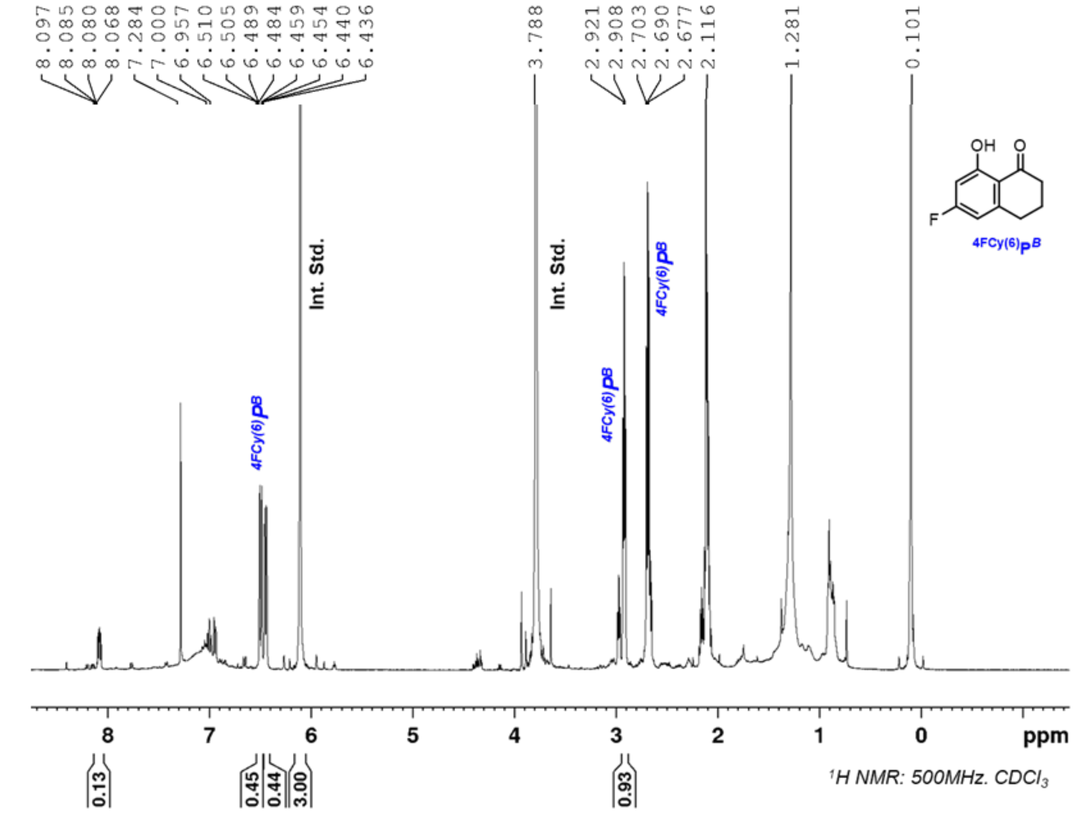

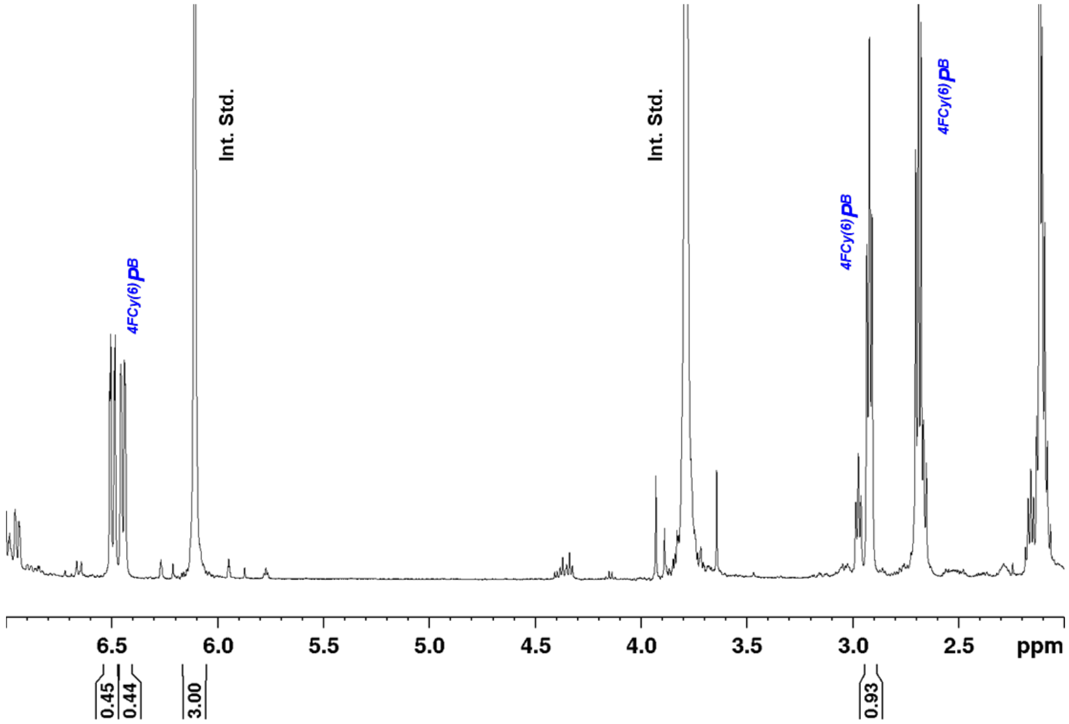

Figure S128. ^{1}H-NMR spectra for the cleavage of $^{4FCy(6)}$PL.

3.46 $^{4HCy(5)}$S and 2-picolylamine

Synthesis of $^{4HCy(5)}$L

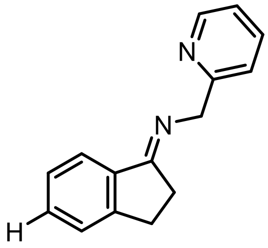

$^{4HCy(5)}$L *B*

In an oven dried flask, 2-picolylamine (2.2 equiv., 2.2mL) was added to 1-Indanone (1.3 g, 9.85 mmol) and p- toluenesulfonic acid monohydrate (cat. 20 mg, 1.2mol%) in toluene (50 mL). The reaction mixture was refluxed under argon with a Dean-Stark apparatus until imine formation was complete (7 hours). The reaction was cooled to room temperature and diluted with diethyl ether (30 mL). The organic layer was washed with saturated ammonia chloride (20 mL x 2), saturated aqueous sodium bicarbonate (20 mL), brine (20 mL), and dried with magnesium sulfate. The final product was isolated as a brown solid (70% yield, 1.54 g, 90% pure). ^{1}H-NMR (500 MHz, $CDCl_3$): δ 8.58 (d, 1H), 7.94 (d, 1H), 7.71 (t, 1H), 7.64 (d, 1H), 7.43 (t, 1H), 7.39 (t, 1H), 7.33 (t, 1H), 7.20 (t, 1H), 4.86 (s, 2H), 3.15 (t, 2H), 2.87 (t, 2H). 13C{1H} NMR (500 MHz, CD_3CN): δ 176.0, 160.4, 149.8, 139.7, 136.6, 131.3, 129.0, 128.2, 126.9, 122.5, 122.0, 121.7, 59.2, 28.7, 28.2. MS (ESI) m/z [M + H]+ calculated 222.2910, found 223.3191.

Hydroxylation of $^{4HCy(5)}$L

The reaction was carried out on 0.159 mmol scale using 40.0 mg of the imine according to the Standard Procedure. The reaction products were quantified using 0.159 mmol of 1,3,5-trimethoxybenzene (int. std.). (24% yield). The identity of the hydroxylation products was confirmed by ^{1}H-NMR.

^{1}H-NMR spectra of $^{4HCy(5)}$L

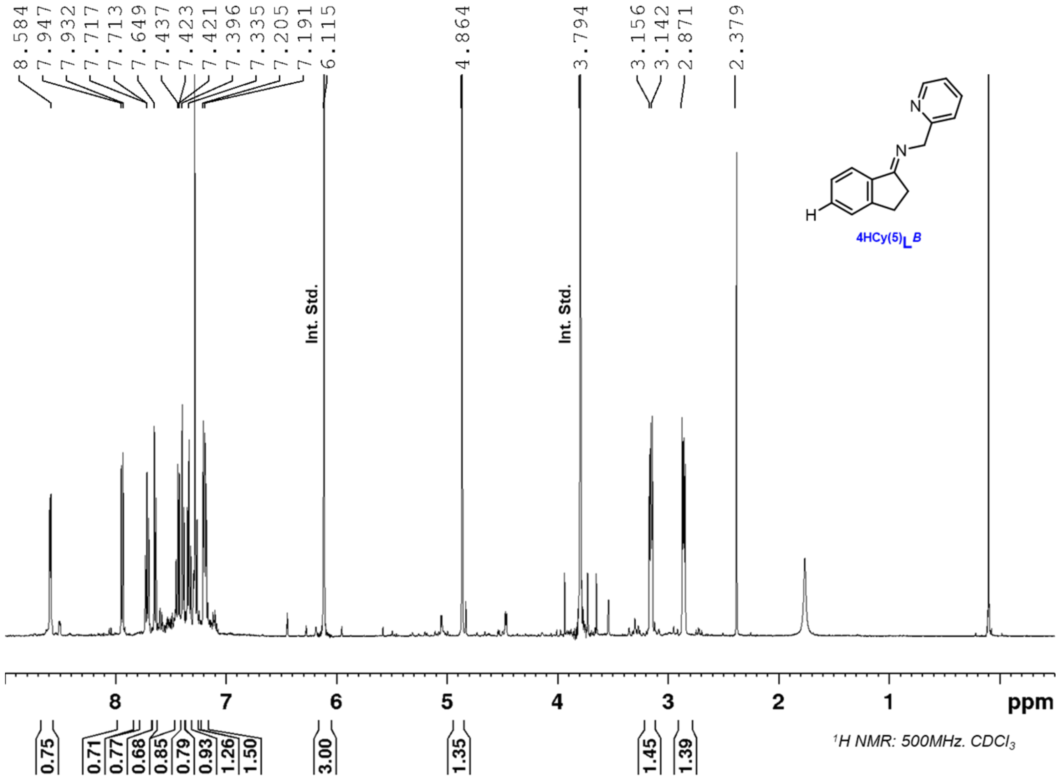

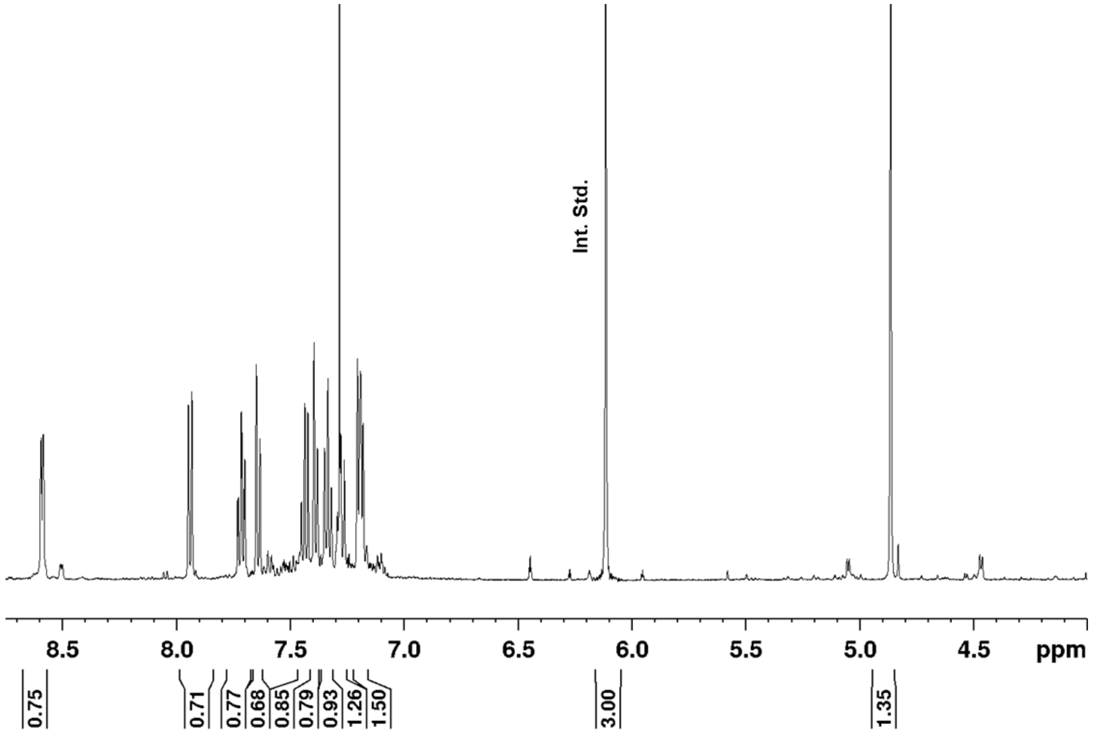

Figure S129. ^{1}H-NMR spectra of $^{4HCy(5)}$L. Note: Only one of the imine isomers is formed.

^{1}H-NMR spectra for the hydroxylation of $^{4HCy(5)}$L

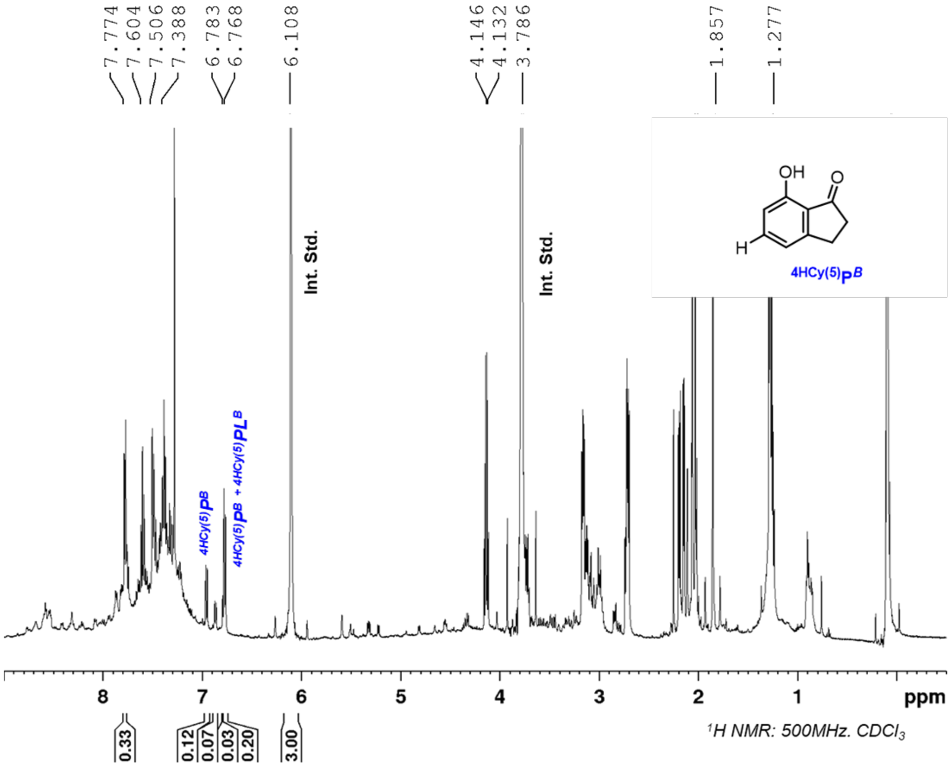

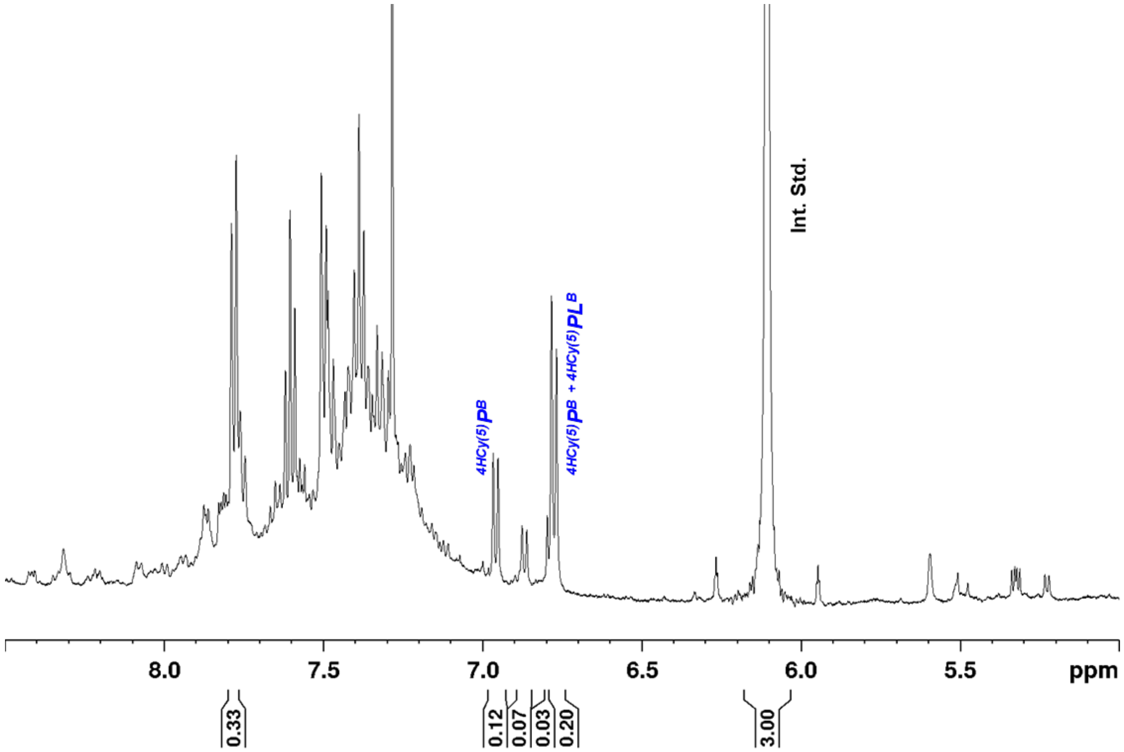

Figure S130. ^{1}H-NMR spectra for the hydroxylation of $^{4HCy(5)}$L.

3.47 $^{4HCy(7)}$S and 2-picolylamine

Synthesis of $^{4HCy(7)}$L

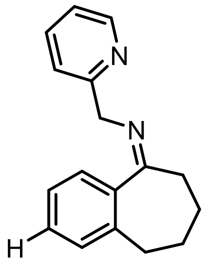

$^{4HCy(7)}$L*A*

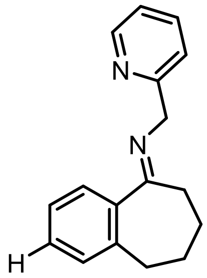

$^{4HCy(7)}$L*B*

In an oven dried flask, 2-picolylamine (2.2 equiv., 2.2 mL) was added to Benzosuberone (1.63 g, 9.85 mmol) and p- toluenesulfonic acid monohydrate (cat. 20 mg, 1.2 mol%) in toluene (50 mL). The reaction mixture was refluxed under argon with a Dean-Stark apparatus until imine formation was complete (12 hours). The reaction was cooled to room temperature and diluted with diethyl ether (30 mL). The organic layer was washed with saturated ammonia chloride (20 mL x 2), saturated aqueous sodium bicarbonate (20 mL), brine (20 mL), and dried with magnesium sulfate. The final product was isolated as a brown solid (75% yield, 1.85 g, 87% pure). ^{1}H-NMR (500 MHz, $CDCl_3$): δ 8.58 (d, 2H), 7.68 (m, 4H), 7.58 (d, 2H), 7.28 (m, 4H), 7.20 (m, 2H), 7.13 (m, 2H), 4.84 (s, 2H), 4.62 (s, 2H), 2.82 (t, 4H), 2.68 (t, 4H), 1.84 (t, 4H), 1.75(t, 4H). 13C{1H} NMR (500 MHz, CD_3CN): δ 175.1, 160.6, 148.9, 136.6, 129.2, 128.5, 127.8, 126.5, 122.1, 121.7, 59.1, 56.9, 40.7, 35.6, 32.0, 30.6, 30.1, 27.3, 25.8, 22.3. MS (ESI) m/z [M + H]+ calculated 250.3450, found 251.1539.

Hydroxylation of $^{4HCy(7)}$L

The reaction was carried out on 0.159 mmol scale using 45.7 mg of the imine according to the Standard Procedure. The reaction products were quantified using 0.159 mmol of 1,3,5-trimethoxybenzene (int. std.). (45% yield). The identity of the hydroxylation products was confirmed by ^{1}H-NMR.

Cleavage of $^{4HCy(7)}$PL

Dissolving *$^{4HCy(7)}$PL* in round bottom flask with 50 mL EtOAc, then adding 100 mL 1M HCl. Reaction was going for 30 min. The resulting mixture was extracted with EtOAc (50 mL X 2). The organic phases were separated, combined, dried over $MgSO_4$, filtered, and dried under vacuum. The reaction products were dissolved in 1.4 mL of $CDCl_3$ solution containing 13.5 mg of 1,3,5-trimethoxybenzene (internal standard). The reaction products were quantified by ^{1}H-NMR using integration signals that correspond to the starting material and products with the integration signal of the internal standard.

^{1}H NMR spectra of $^{4HCy(7)}$L

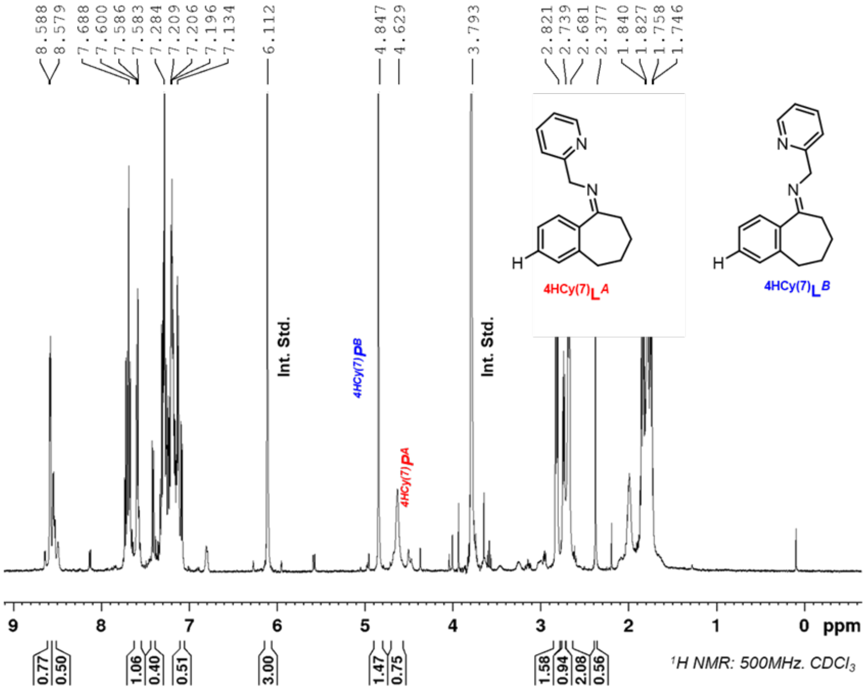

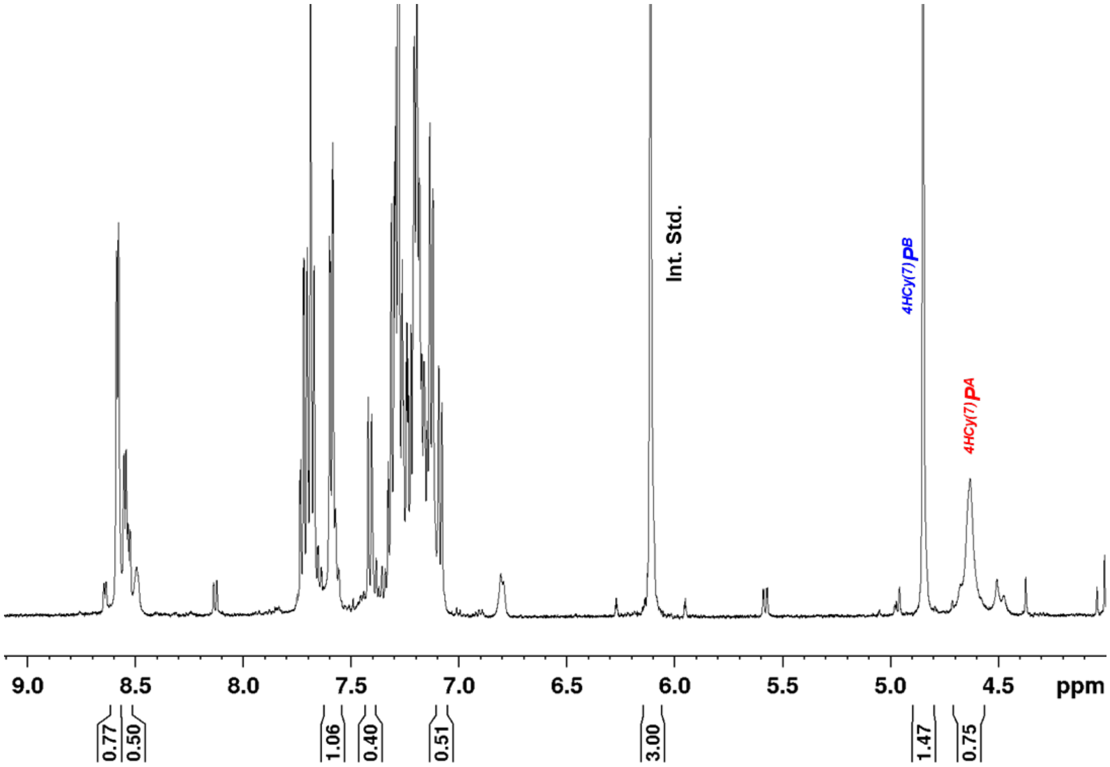

Figure S131. ^{1}H-NMR spectra of $^{4HCy(7)}$L. Note: Note: two imine isomers were formed. The ratio of $^{4HCy(7)}$L^{A} and $^{4HCy(7)}$L^{B} (34/66) is calculated using the average of the integration of CH_2 peaks and CH peaks.

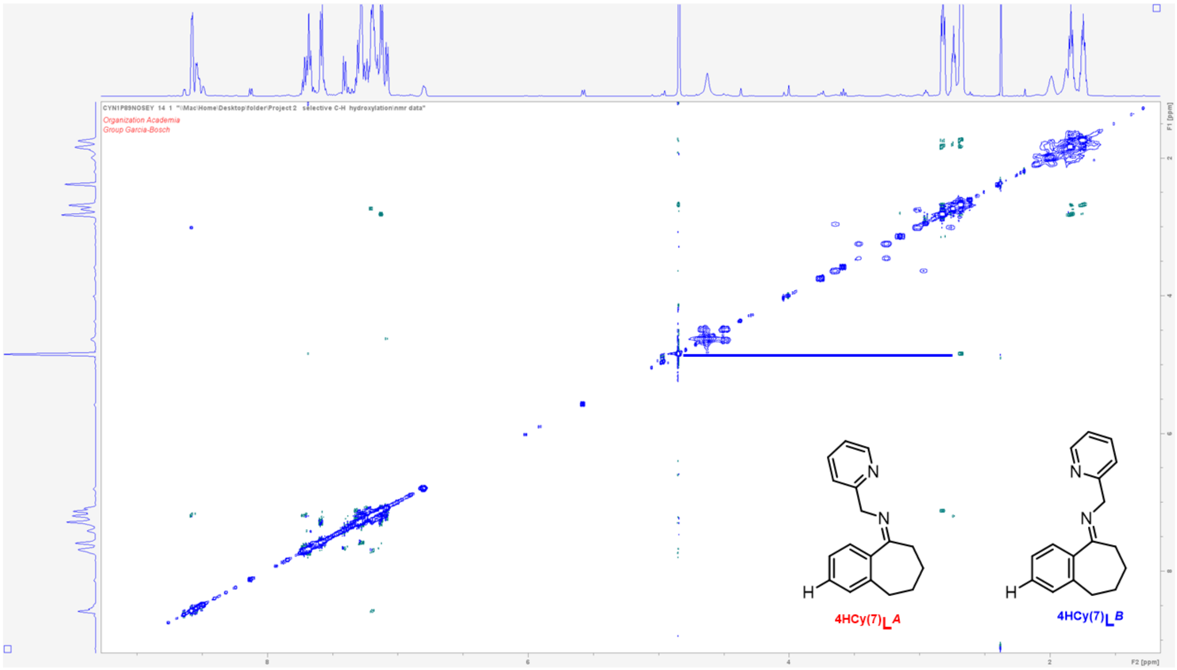

Figure 132. Nuclear Overhauser Effect Spectroscopy (NOESY) spectra for $^{4HCy(7)}L$

^{1}H NMR spectra for the Hydroxylation of $^{4HCy(7)}$L

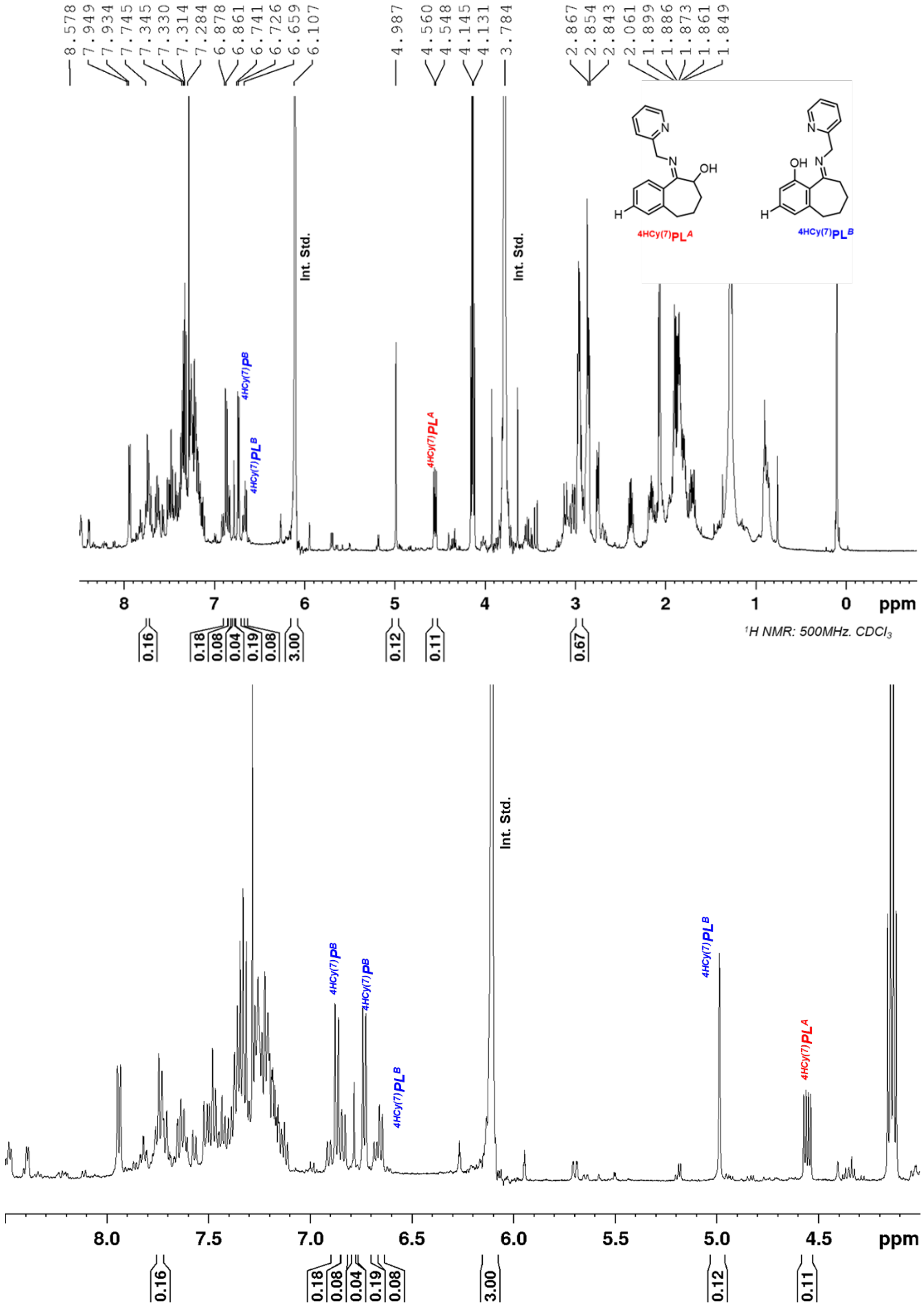

Figure S133. ^{1}H-NMR spectra for the hydroxylation of $^{4HCy(7)}$L. Note: The ratio of $^{4HCy(7)}$PL^{A} and $^{4HCy(7)}$PL^{B} (25/75) is calculated using the average of the integration of CH_2 peaks and CH peaks.

^{1}H NMR spectra for the cleavage of $^{4HCy(7)}$PL

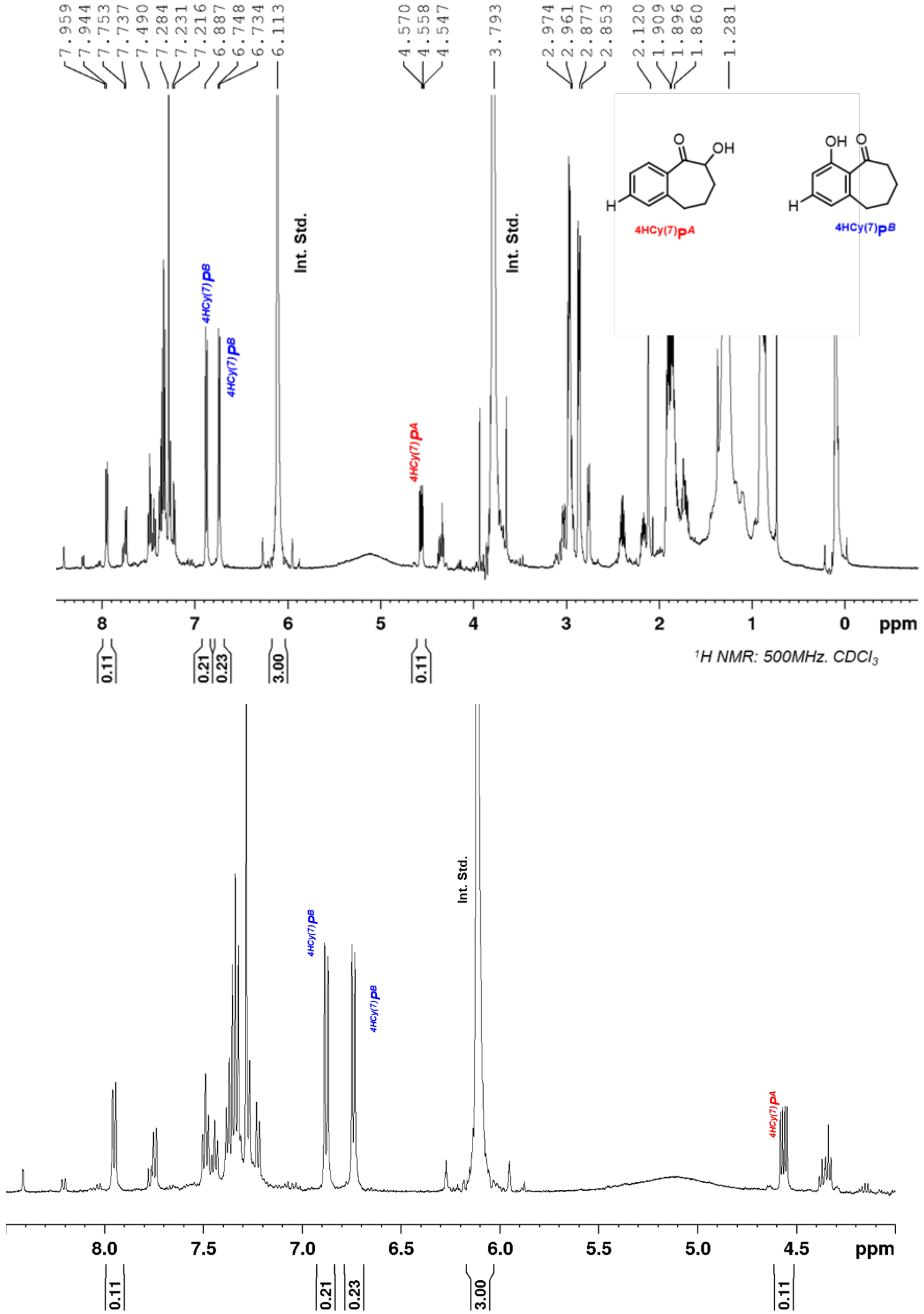

Figure S134. ^{1}H-NMR spectra for the cleavage of $^{4HCy(7)}$PL.

3.48 $^{4HCy(7)}$S and 2-(aminomethyl)-4-methoxypyridine

Synthesis of $^{4HCy(7)}L_{4MeO-Py}$

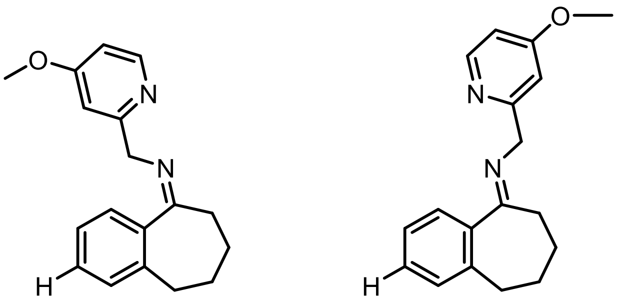

$^{4HCy(7)}L^{A}_{4MeO-Py}$ $^{4HCy(7)}L^{B}_{4MeO-Py}$

In an oven dried flask, 2-(aminomethyl)-4-methoxypyridine (2.2 equiv., 700 mg) was added to 1-Benzocycloheptanone (400 mg, 2.57 mmol) and p- toluenesulfonic acid monohydrate (cat. 10 mg, 2.2 mol%) in toluene (50 mL). The reaction mixture was refluxed under argon with a Dean-Stark apparatus until imine formation was complete (12 hours). The reaction was cooled to room temperature and diluted with diethyl ether (30 mL). The organic layer was washed with saturated ammonia chloride (20 mL x 2), saturated aqueous sodium bicarbonate (20 mL), brine (20 mL), and dried with magnesium sulfate. The final product was isolated as a brown solid (81% yield, 585 mg, 70% pure). ^{1}H-NMR (500 MHz, $CDCl_3$): δ 8.40 (d, 1H), 8.32 (d, 1H), 7.61 (d, 2H), 7.34 - 7.22 (m, 5H), 6.80-6.68 (m, 5H), 4.80 (s, 2H). 4.57 (s, 2H), 2.80 (t, 4H), 2.72 (t, 4H), 1.83(t, 4H), 1.73(t, 4H). 13C{1H} NMR (500 MHz, CD_3CN): δ 175.0, 166.6, 162.4, 150.3, 138.5, 129.1, 128.4, 127.7, 126.5, 108.3, 107.6, 58.9, 56.8, 55.1, 40.7, 32.1, 30.6, 30.1, 27.3, 25.8, 22.3, 21.41. MS (ESI) m/z [M + H]+ calculated 280.3710, found 281.1797.

Hydroxylation of $^{4HCy(7)}L_{4MeO-Py}$

The reaction was carried out on 0.159 mmol scale using 70.0 mg of the imine according to the Standard Procedure. The reaction products were quantified using 0.159 mmol of 1,3,5-trimethoxybenzene (int. std.). (50% yield). The identity of the hydroxylation products was confirmed by ^{1}H-NMR.

^{1}H-NMR spectra of $^{4HCy(7)}L_{4MeO\text{-}Py}$

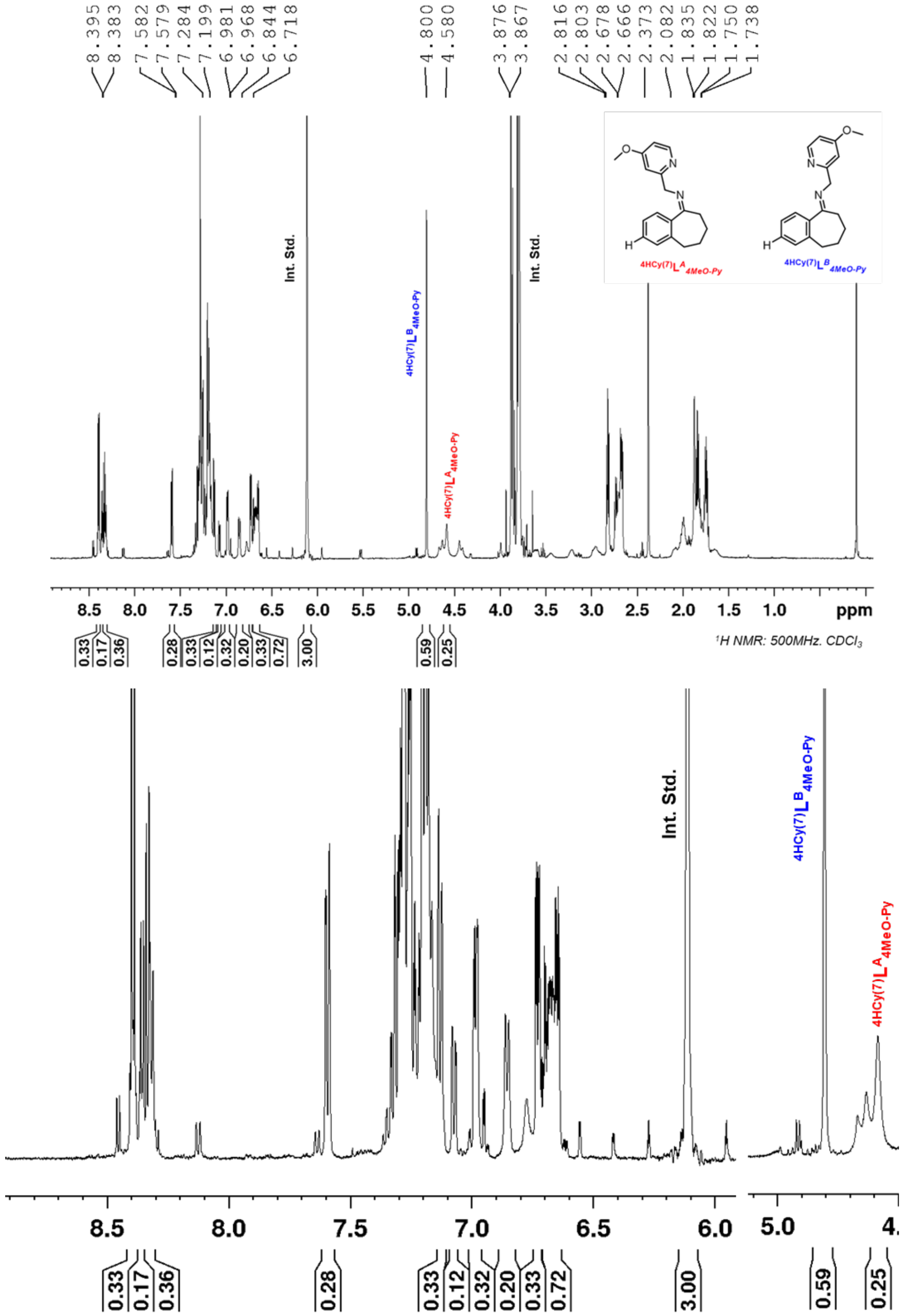

Figure S135. ^{1}H-NMR spectra of $^{4HCy(7)}L_{4MeO\text{-}Py}$. Note: Note: two imine isomers were formed. The ratio of $^{4HCy(7)}L^{A}_{4MeO\text{-}Py}$ and $^{4HCy(7)}L^{B}_{4MeO\text{-}Py}$ (33/67) is calculated using the average of the integration of CH_2 peaks and CH peaks.

^{1}H-NMR spectra for the hydroxylation of $^{4HCy(7)}L_{4MeO\text{-}Py}$

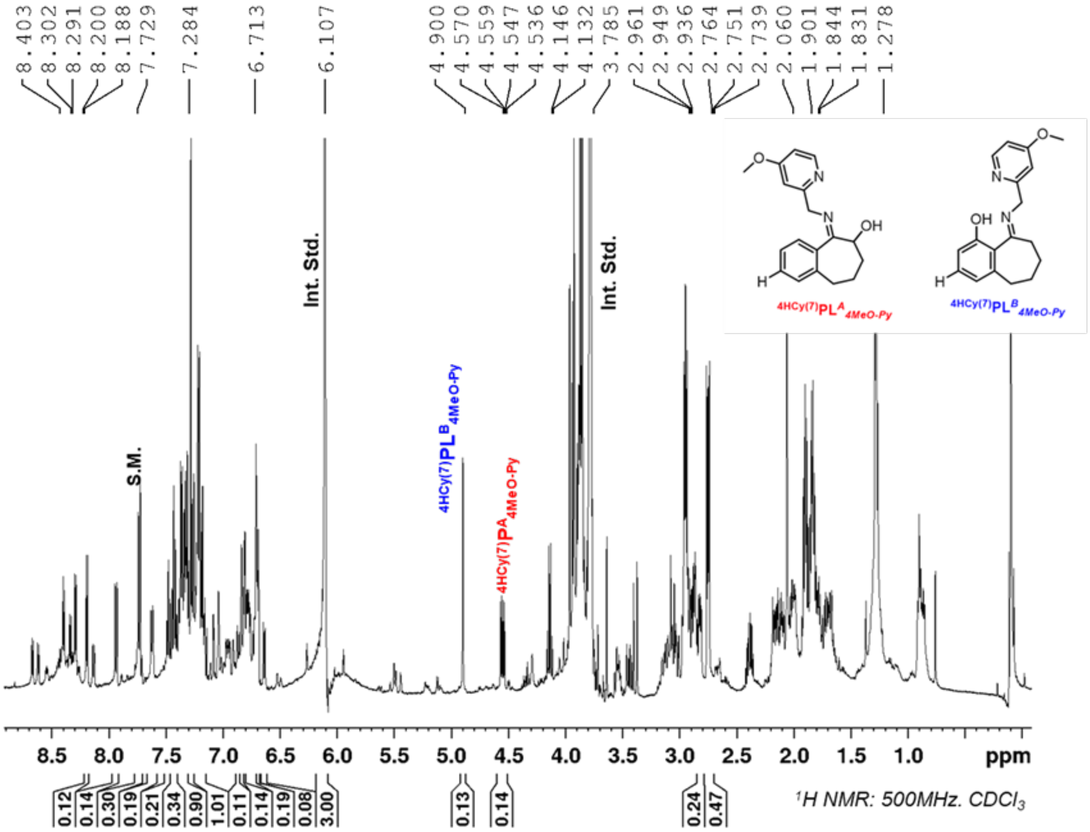

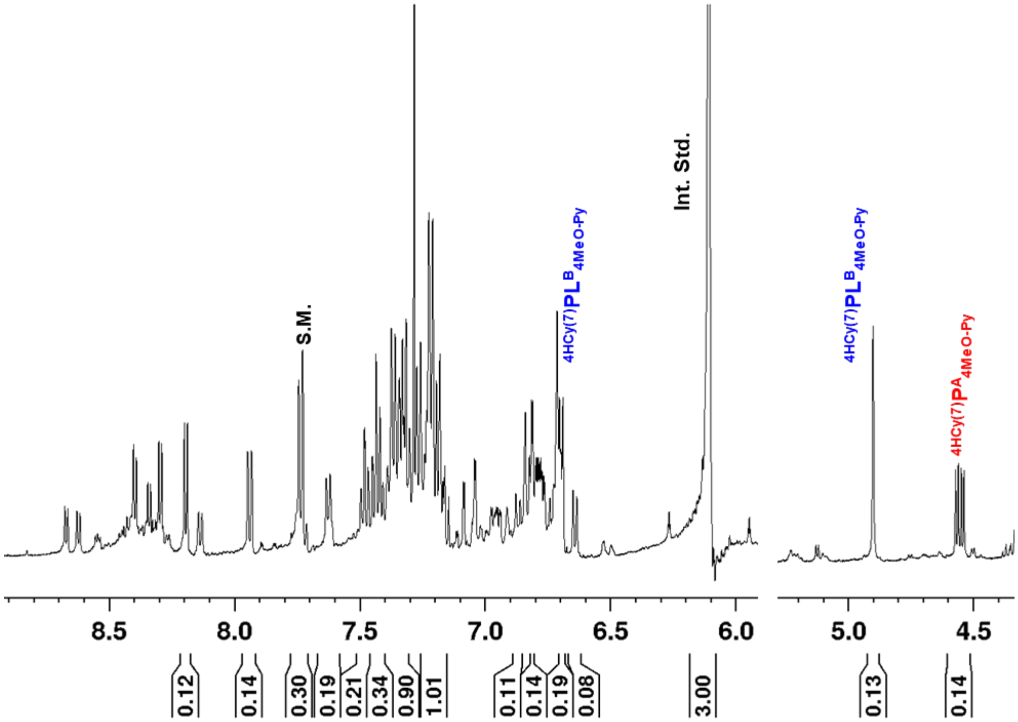

Figure S136. ^{1}H-NMR spectra for the hydroxylation of $^{4HCy(7)}L_{4MeO\text{-}Py}$. Note: The ratio of $^{4HCy(7)}PL^{A}_{4MeO\text{-}Py}$ and $^{4HCy(7)}PL^{B}_{4MeO\text{-}Py}$ (24/76) is calculated using the average of the integration of CH_2 peaks and CH peaks.

3.49 $^{4HCy(7)}$S and 2-(aminomethyl)-4-chloropyridine

Synthesis of $^{4HCy(7)}L_{4Cl\text{-}Py}$

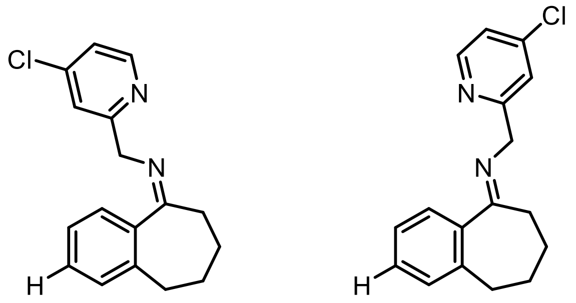

$^{4HCy(7)}L^{A}_{4Cl\text{-}Py}$ $^{4HCy(7)}L^{B}_{4Cl\text{-}Py}$

In an oven dried flask, 2-(aminomethyl)-4-chloropyridine (2.2 equiv., 700 mg) was added to Benzosuberone (400 mg, 2.57 mmol) and p- toluenesulfonic acid monohydrate (cat. 10 mg, 2.2 mol%) in toluene (50 mL). The reaction mixture was refluxed under argon with a Dean-Stark apparatus until imine formation was complete (12 hours). The reaction was cooled to room temperature and diluted with diethyl ether (30 mL). The organic layer was washed with saturated ammonia chloride (20 mL x 2), saturated aqueous sodium bicarbonate (20 mL), brine (20 mL), and dried with magnesium sulfate. The final product was isolated as a brown solid (70% yield, 513 mg, 85% pure). ^{1}H-NMR (500 MHz, $CDCl_3$): δ 8.45 (d, 2H), 7.77 (d, 2H), 7.43-7.10 (m, 10H), 4.81 (s, 2H), 4.60 (s, 2H), 2.94 (t, 2H), 2.82 (t, 2H), 2.76 (t, 2H), 2.67 (t, 2H), 1.91 (t, 2H), 1.84(m, 4H), 1.75 (t, 2H). 13C{1H} NMR (500 MHz, CD_3CN): δ 162.5, 131.2, 129.6, 128.8, 127.7, 126.6, 122.6, 122.0, 58.5, 40.9, 32.5, 30.8, 27.3, 25.2, 22.2, 21.4, 20.9. MS (ESI) m/z [M + H]+ calculated 284.7870, found 285.1112.

Hydroxylation of $^{4HCy(7)}L_{4Cl\text{-}Py}$

The reaction was carried out on 0.159 mmol scale using 53.0 mg of the imine according to the Standard Procedure. The reaction products were quantified using 0.159 mmol of 1,3,5-trimethoxybenzene (int. std.). (41% yield). The identity of the hydroxylation products was confirmed by ^{1}H-NMR.

^{1}H-NMR spectra of $^{4HCy(7)}L_{4Cl\text{-}Py}$

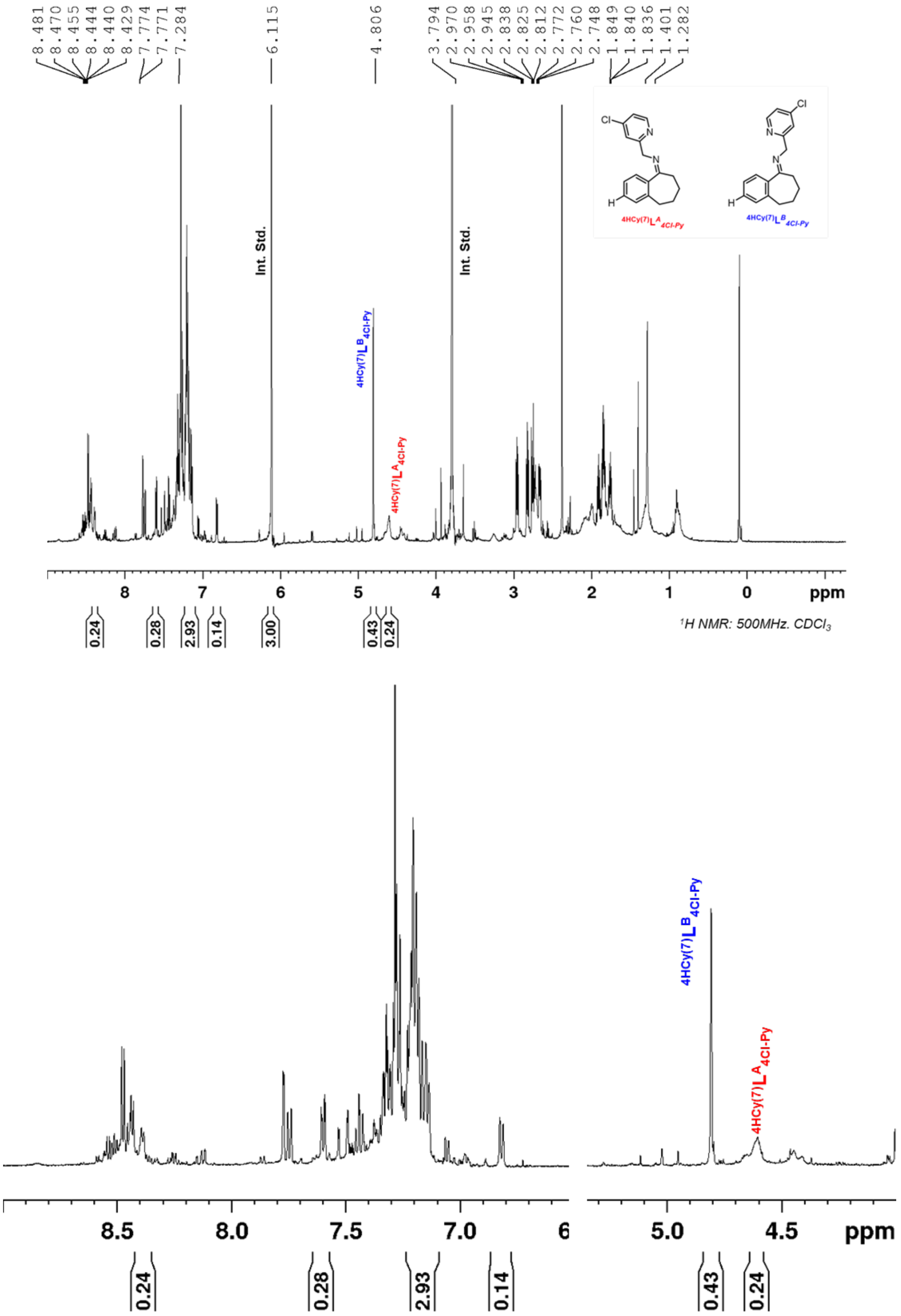

Figure S137. ^{1}H-NMR spectra of $^{4HCy(7)}L_{4Cl\text{-}Py}$. Note: There are two imine isomers. The ratio of $^{4HCy(7)}L^{A}_{4Cl\text{-}Py}$ and $^{4HCy(7)}L^{B}_{4Cl\text{-}Py}$ (35/65) is calculated using the average of the integration of CH_2 peaks.

^{1}H-NMR spectra for the hydroxylation of $^{4HCy(7)}L_{4Cl-Py}$

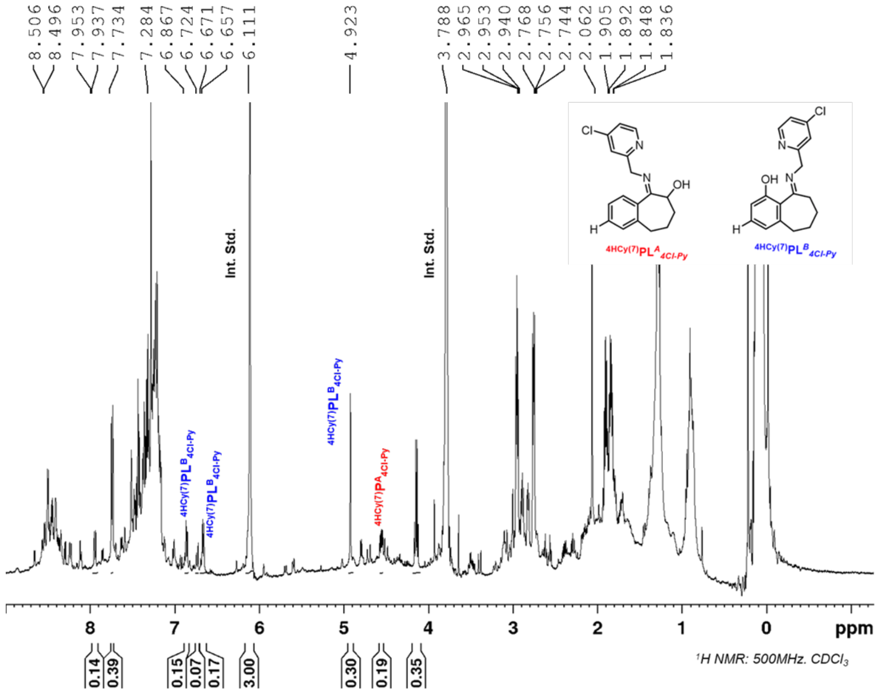

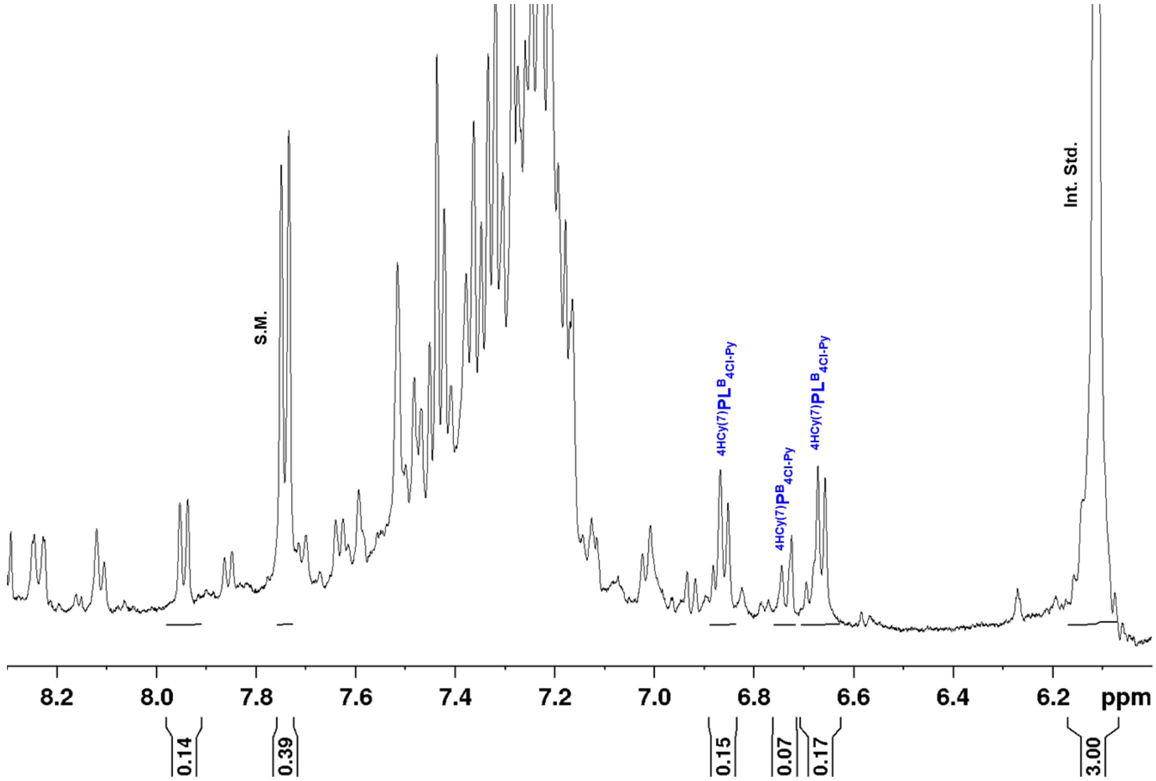

Figure S138. ^{1}H-NMR spectra for the hydroxylation of $^{4HCy(7)}PL^{A}_{4Cl-Py}$. Note: The ratio of $^{4HCy(7)}PL^{A}_{4Cl-Py}$ and $^{4HCy(7)}PL^{B}_{4Cl-Py}$ (46/54) is calculated using the average of the integration of CH_2 peaks and CH peaks.

4. ^{1}H-NMR experiments included in Figure 6

Analysis of the imine substrate-ligands: ^{1}H-NMR, ^{13}C-NMR, COSY and NOESY measurements were carried out in order to characterize the two isomers formed in the synthesis of the imine substrate-ligands. The ratio of the isomers is calculated using the average of the integration of CH_2 peaks (between 4.5 and 5.0 ppm) and CH peaks (between 7.0 and 7.5 ppm). No variations in the ratio of the isomers was found when the analysis was carried out in $CDCl_3$ or CD_3CN.

Analysis of the cuprous complexes: In the glovebox, 0.159 mmols of the imine substrate-ligand and 0.159 mmol of $[Cu^{I}(CH_3CN)_4](PF_6)$ were mixed in CD_3CN. The resulting solution was analyzed by ^{1}H-NMR. The ratio of the isomers is calculated using the average of the integration of CH_2 peaks (between 4.5 and 5.0 ppm) and CH peaks (between 7.0 and 7.5 ppm).

Analysis of the hydroxylation products derived from the oxidation with Cu^{I} and H_2O_2: In the glovebox, 4 mL of acetone were added to a 20-mL vial containing 0.159 mmols of the imine substrate-ligand equipped with a stir bar. To the solution, 0.159 mmol of $[Cu^{I}(CH_3CN)_4](PF_6)$ was added and allowed to react. The solution mixture was taken out of the glovebox and 5 equiv of 30% H_2O_2 were added. After 30 min, the reaction was quenched using Na_2EDTA (50 mL, pH = 4). The resulting mixture was extracted with EtOAc (50 mL X 3). The organic phases were separated, combined, dried over $MgSO_4$, filtered, and dried under vacuum. The reaction products were dissolved in 1.4 mL of $CDCl_3$ solution containing 27.1 mg of 1,3,5-trimethoxybenzene (internal standard). The reaction products were quantified by ^{1}H-NMR using integration signals that correspond to the starting material and products with the integration signal of the internal standard. Note: a similar protocol was followed for the analysis of the reactions carried out at different temperatures and using different solvents (CH_3CN, THF and CH_2Cl_2).

Analysis of the hydroxylation products derived from the oxidation with Cu^{II}, OH^- and H_2O_2: In the glovebox, 4 mL of acetone were added to a 20-mL vial containing 0.159 mmols of the imine substrate-ligand equipped with a stir bar. To the solution, 0.159 mmol of $Cu^{II}(CF_3SO_3)_2$ and 0.159 mmol of $NMe_4OH \cdot 5H_2O$ were added and allowed to react. The solution mixture was taken out of the glovebox and 5 equiv of 30% H_2O_2 were added. After 30 min, the reaction was quenched using Na_2EDTA (50 mL, pH = 4). The resulting mixture was extracted with EtOAc (50 mL X 3). The organic phases were separated, combined, dried over $MgSO_4$, filtered, and dried under vacuum. The reaction products were dissolved in 1.4 mL of $CDCl_3$ solution containing 27.1 mg of 1,3,5-trimethoxybenzene (internal standard).

Analysis of the hydroxylation products derived from the oxidation with Cu^{I} and O_2: In the glovebox, 4 mL of acetone were added to a 20-mL vial containing 0.159 mmols of the imine substrate-ligand equipped with a stir bar. To the solution, 0.159 mmol of $[Cu^{I}(CH_3CN)_4](PF_6)$ was added and allowed to react. The solution mixture was taken out of the glovebox and O_2 was bubbled though the solution for 30 seconds. The reaction was warmed up to 50 C and reacted overnight. After that, the reaction was quenched using Na_2EDTA (50 mL, pH = 4). The resulting mixture was extracted with EtOAc (50 mL X 3). The organic phases were

separated, combined, dried over $MgSO_4$, filtered, and dried under vacuum. The reaction products were dissolved in 1.4 mL of $CDCl_3$ solution containing 27.1 mg of 1,3,5-trimethoxybenzene (internal standard). The reaction products were quantified by ^{1}H-NMR using integration signals that correspond to the starting material and products with the integration signal of the internal standard.

Analysis of the imine substrate-ligands after addition and removal of copper: In the glovebox, 4 mL of acetone were added to a 20-mL vial containing 0.159 mmols of the imine substrate-ligand equipped with a stir bar. To the solution, 0.159 mmol of $[Cu^I(CH_3CN)_4](PF_6)$ (0.159 mmol of $Cu^{II}(CF_3SO_3)_2$ and 0.159 mmol of $NMe_4OH·5H_2O$) was added and allowed to react. The solution mixture was taken out of the glovebox. After 30 min, the reaction was quenched using Na_2EDTA (50 mL, pH = 4) and was extracted with EtOAc (50 mL X 3). The organic phases were separated, combined, dried over $MgSO_4$, filtered, and dried under vacuum. The reaction products were dissolved in 1.4 mL of $CDCl_3$ solution containing 27.1 mg of 1,3,5-trimethoxybenzene (internal standard). The reaction products were quantified by ^{1}H-NMR using integration signals that correspond to the starting material and products with the integration signal of the internal standard.

Table S1. Determination of the *E/Z* ratio of ligands, cuprous complexes and hydroxylation products under different reactions conditions.

Entry	System	Ligand *E/Z* ratio[a]	Ligand/Cu *E/Z* ratio[b]	Hydroxylation *E/Z* ratio[c]	Yield (%)	Mass balance (%)
1	^{4F}**L**	64/36	-	-	-	-
2	^{4F}**L** + Cu^I	-	57/43	-	-	-
3	^{4F}**L** + Cu^I + H_2O_2	-	-	33/67	60	67
4	^{4F}**L** + Cu^I + Na_2EDTA[d]	70/30	-	-	-	-
5	^{4F}**L** + Cu^{II} + NMe_4OH + Na_2EDTA[d]	72/28	-	-	-	-
6	^{4F}**L** + Cu^I + H_2O_2 (-90 ºC)	-	-	42/58	24	75
7	^{4F}**L** + Cu^I + H_2O_2 (-40 ºC)	-	-	38/62	25	67
8	^{4F}**L** + Cu^I + H_2O_2 (50 ºC)	-	-	34/66	40	63
9	^{4F}**L** + Cu^I + H_2O_2 (CH_3CN)	-	-	36/64	40	61
10	^{4F}**L** + Cu^I + H_2O_2 (THF)	-	-	33/67	15	57
11	^{4F}**L** + Cu^I + H_2O_2 (CH_2Cl_2)	-	-	33/67	10	60
12	^{4F}**L** + Cu^{II} + NMe_4OH + H_2O_2	-	-	38/62	56	71
13	^{4F}**L** + Cu^I + O_2 (50 ºC)	-	-	40/60	29	63
14	^{4MeO}**L**	67/33	-	-	-	-
15	^{4MeO}**L** + Cu^I	-	67/33	-	-	-
16	^{4MeO}**L** + Cu^I + H_2O_2	-	-	62/38	60	81
17	^{2MeO}**L**	0/100	-	-	-	-
18	^{2MeO}**L** + Cu^I	-	0/100	-	-	-
19	^{2MeO}**L** + Cu^I + H_2O_2	-	-	0/100	0/100	0/100

[a] The *E/Z* ratio of the ligands was determined in $CDCl_3$ at room temperature. [b] The *E/Z* ratio of the $[LCu^I]^{1+}$ complex formed upon addition of 1 equiv of $[Cu^I(CH_3CN)_4](PF_6)$ to the ligand in CD_3CN and at room temperature. [c]The *E/Z* ratio of the hydroxylation products, reaction yields and mass balance of the hydroxylation reaction was determined in $CDCl_3$ at room temperature using I.S. [d]Upon addition of Cu^I (or Cu^{II} + OH^-), the reaction was quenched with Na_2EDTA. The *E/Z* ratio of the ligand was determined in $CDCl_3$ at room temperature.

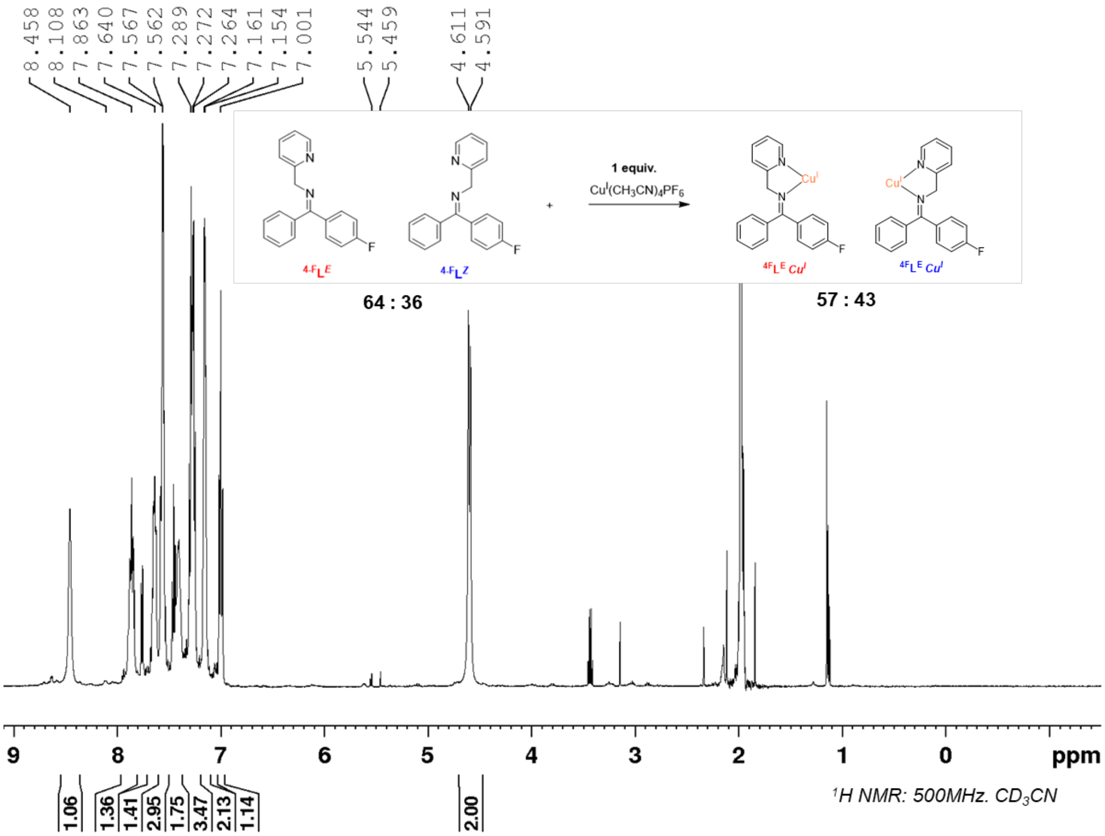

Figure S139. ^{1}H-NMR spectra of ^{4F}L after addition of Cu^{I} in CD_3CN.

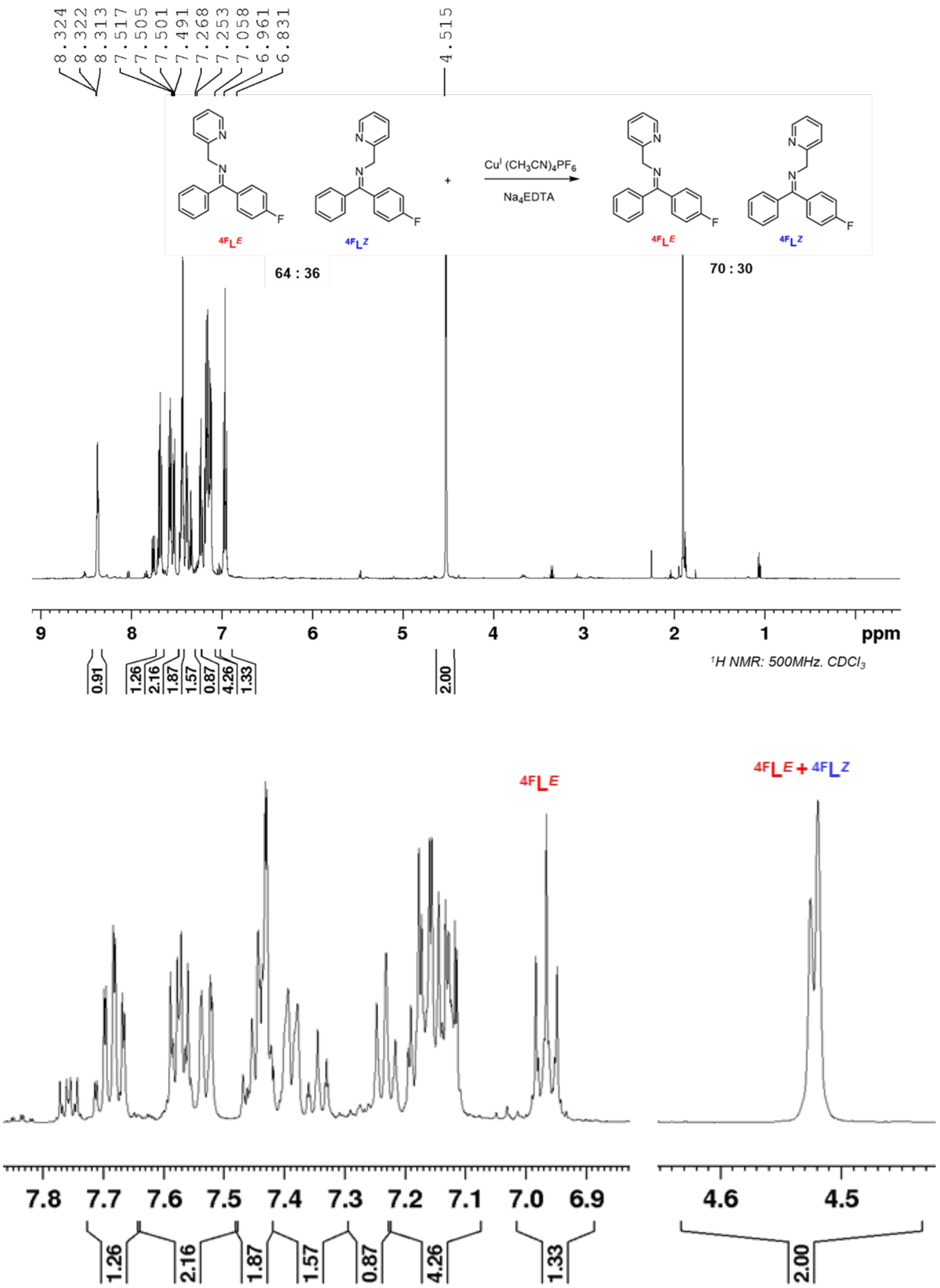

Figure S140. ^{1}H-NMR spectra of ^{4F}L after addition and removal of Cu^I.

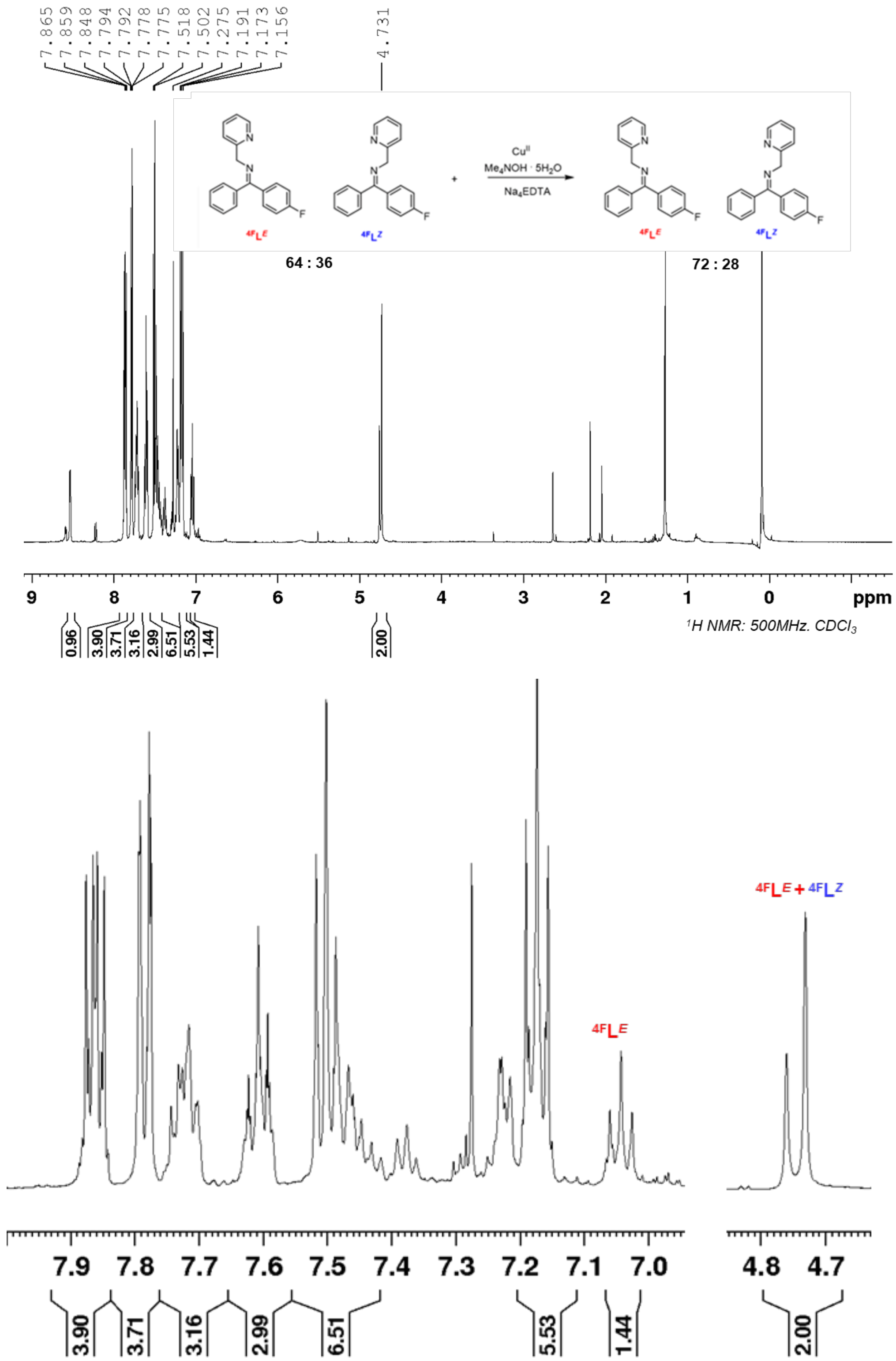

Figure S141. ^{1}H-NMR spectra of ^{4F}L after addition and removal of Cu^{II} and OH^{-}.

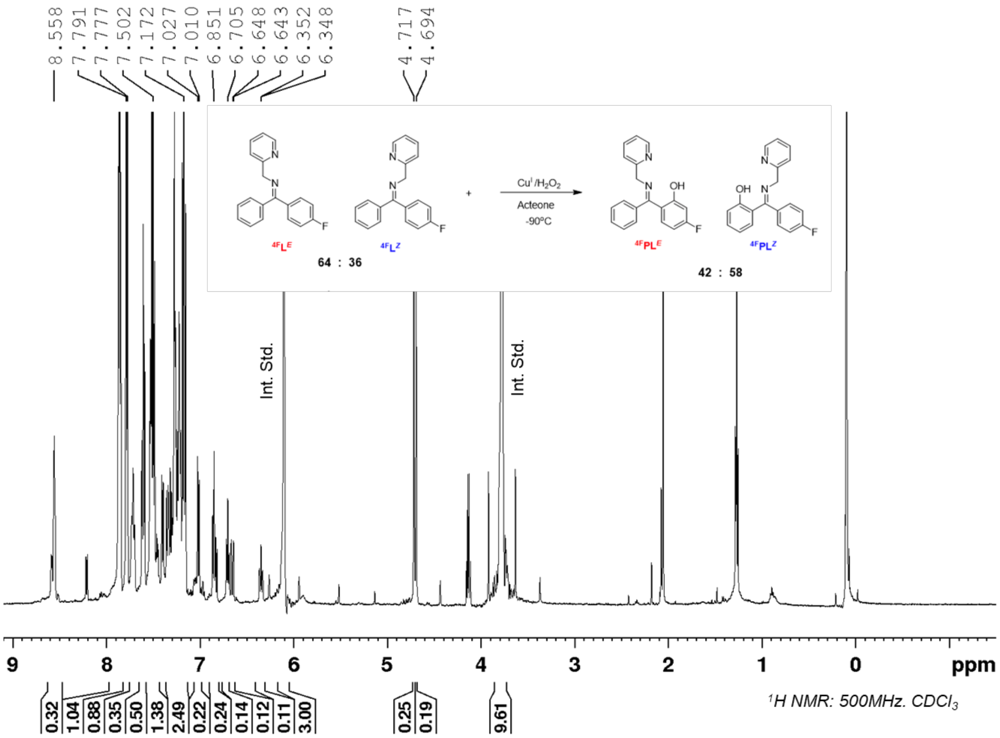

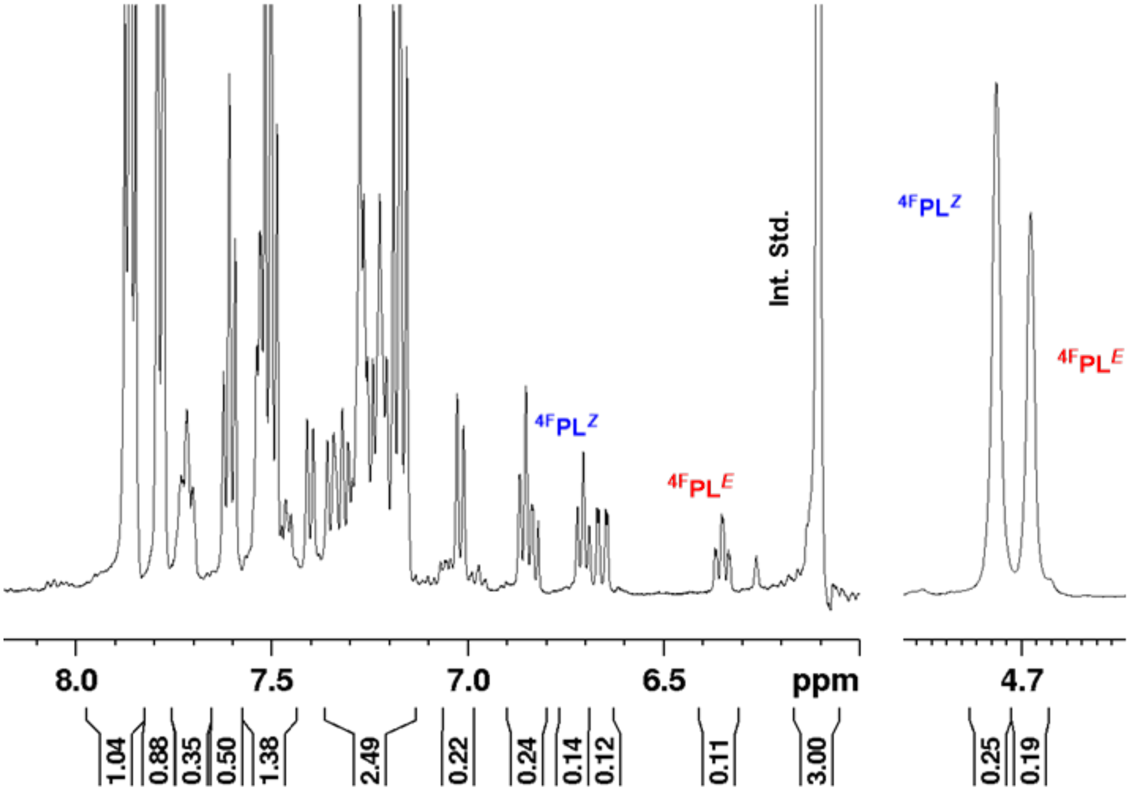

Figure S142. ^{1}H-NMR spectra for the hydroxylation of ^{4F}L with Cu^I and H_2O_2 at -90ºC.

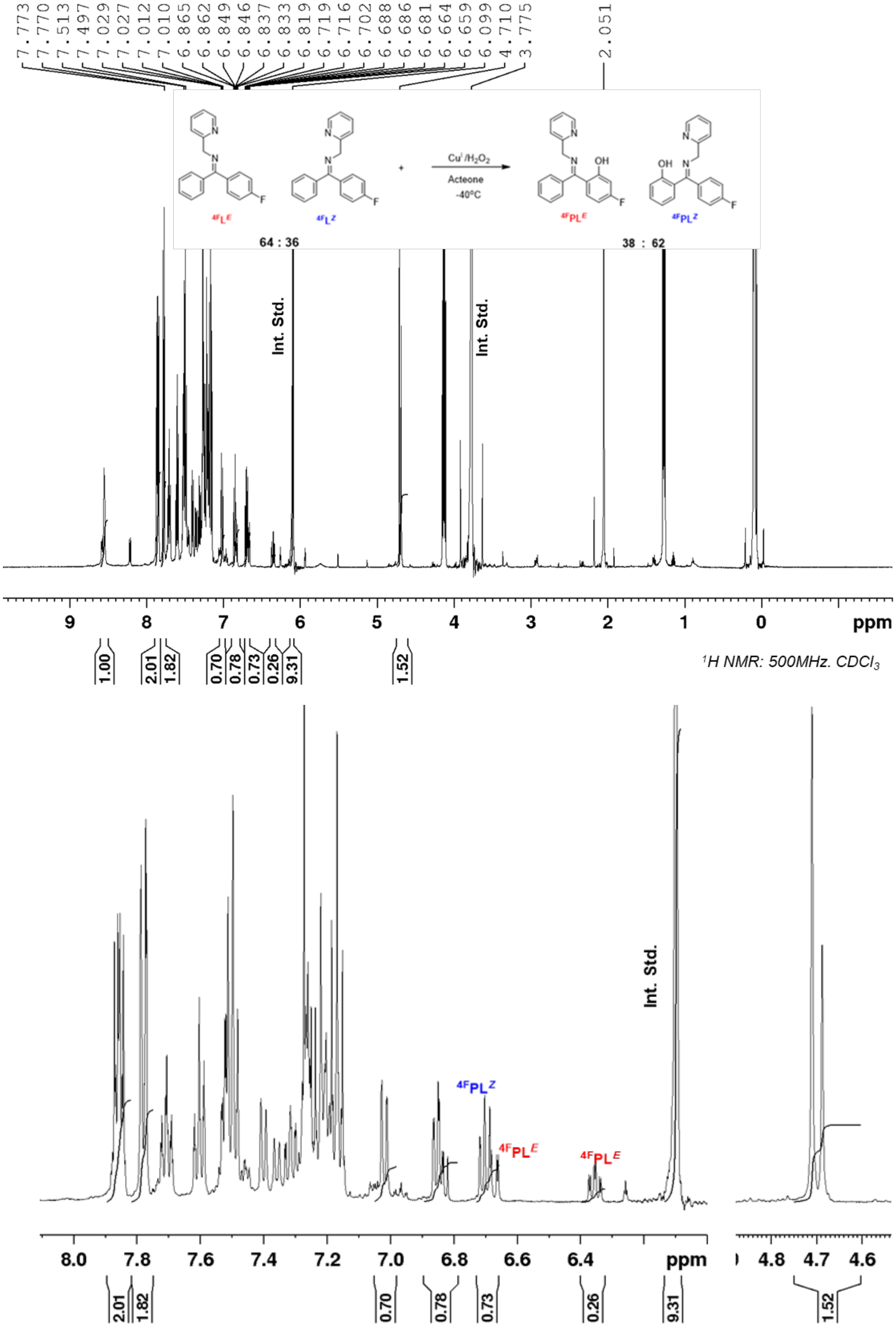

Figure S143. ^{1}H-NMR spectra for the hydroxylation of ^{4F}L with Cu^{I} and H_2O_2 at -40ºC.

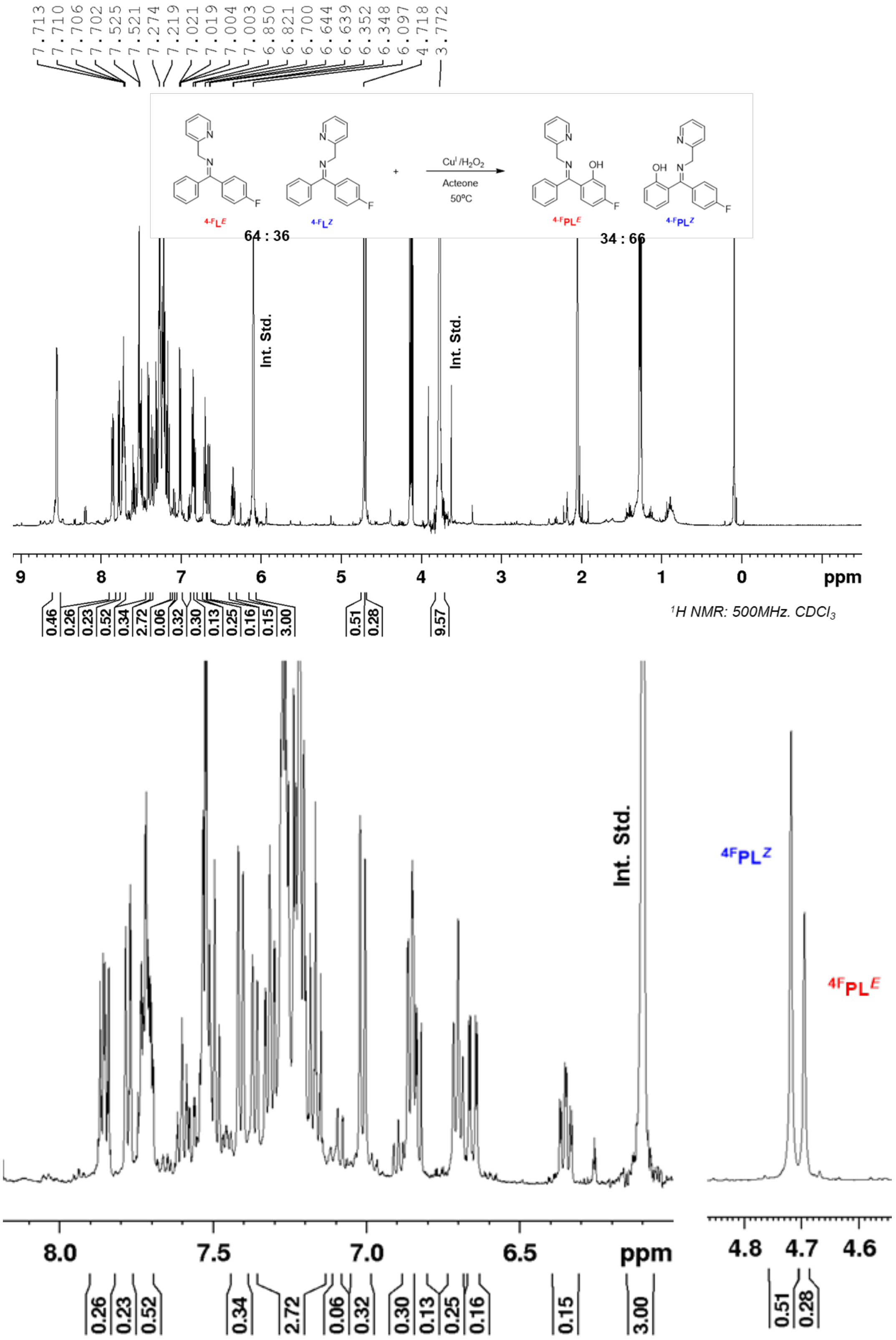

Figure S144. ^{1}H-NMR spectra for the hydroxylation of ^{4F}L with Cu^I and H_2O_2 at 50°C.

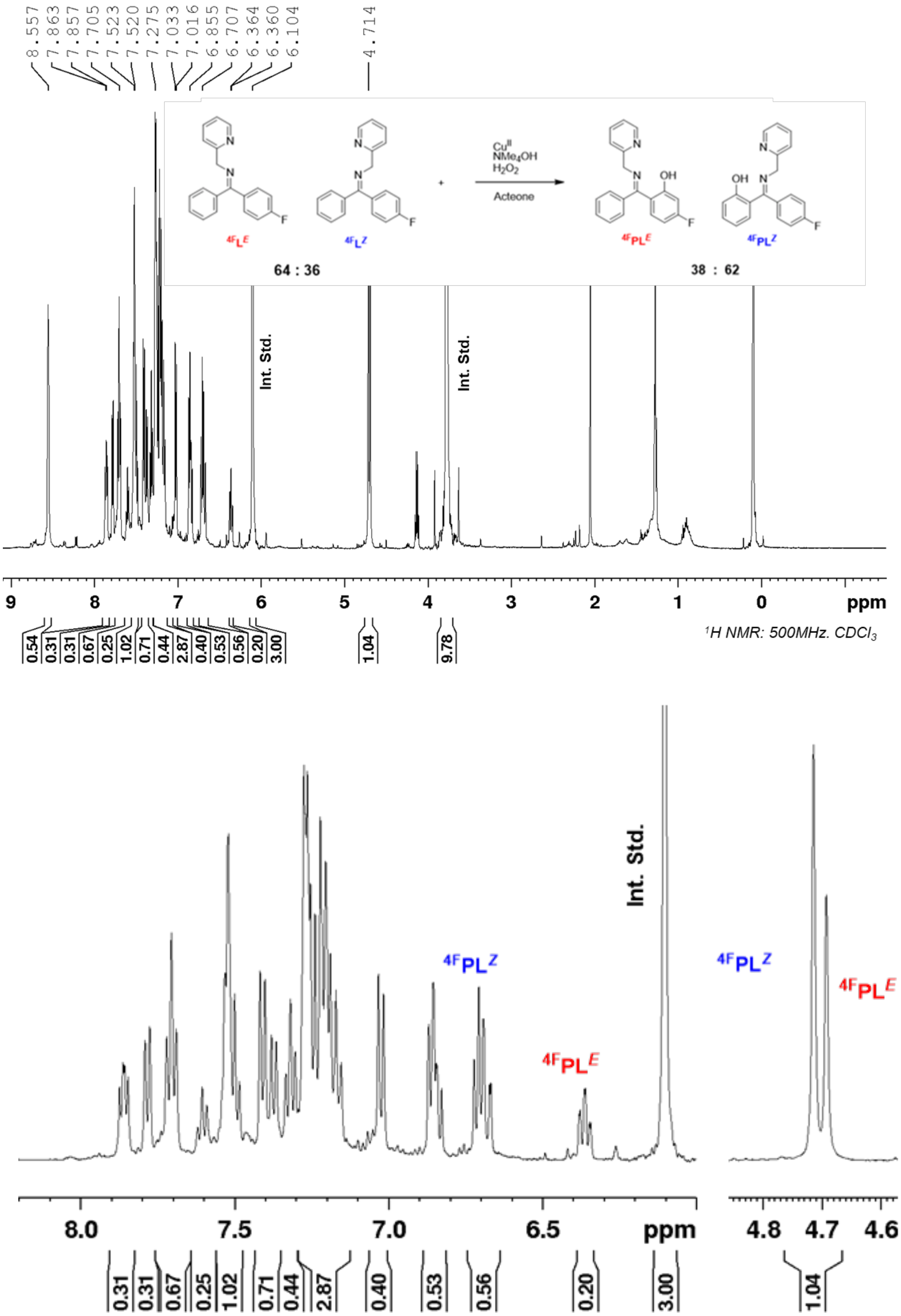

Figure S145. ^{1}H-NMR spectra for the hydroxylation of ^{4F}L with Cu^{II}, OH^- and H_2O_2.

^{1}H NMR for the reaction of 4F-ligand + Cu^{I} + O_2 (50°C)

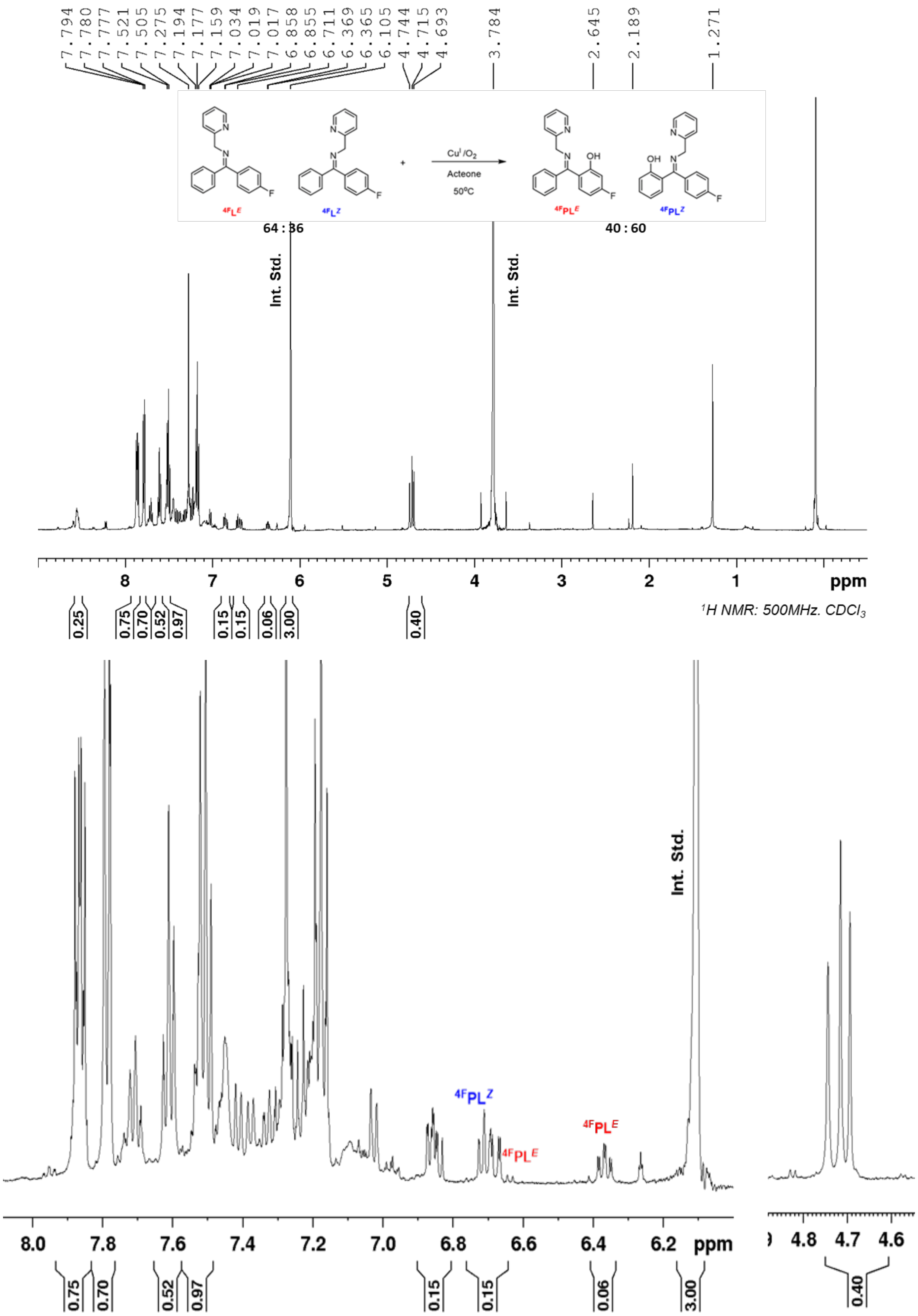

Figure S146. ^{1}H-NMR spectra for the hydroxylation of ^{4F}L with Cu^{I} and O_2 at 50°C.

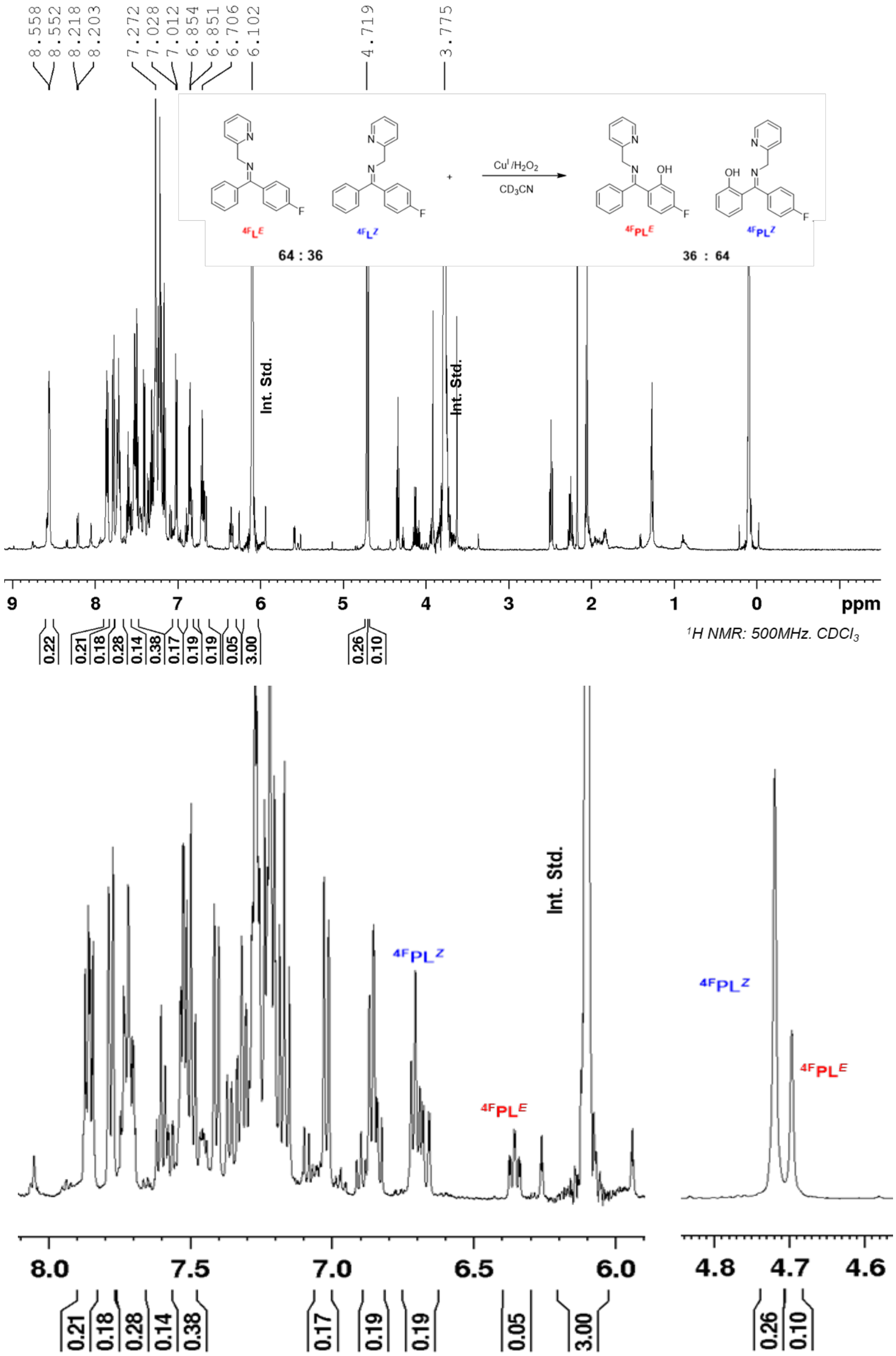

Figure S147. ^{1}H-NMR spectra for the hydroxylation of ^{4F}L with Cu^I and H_2O_2 in CH_3CN.

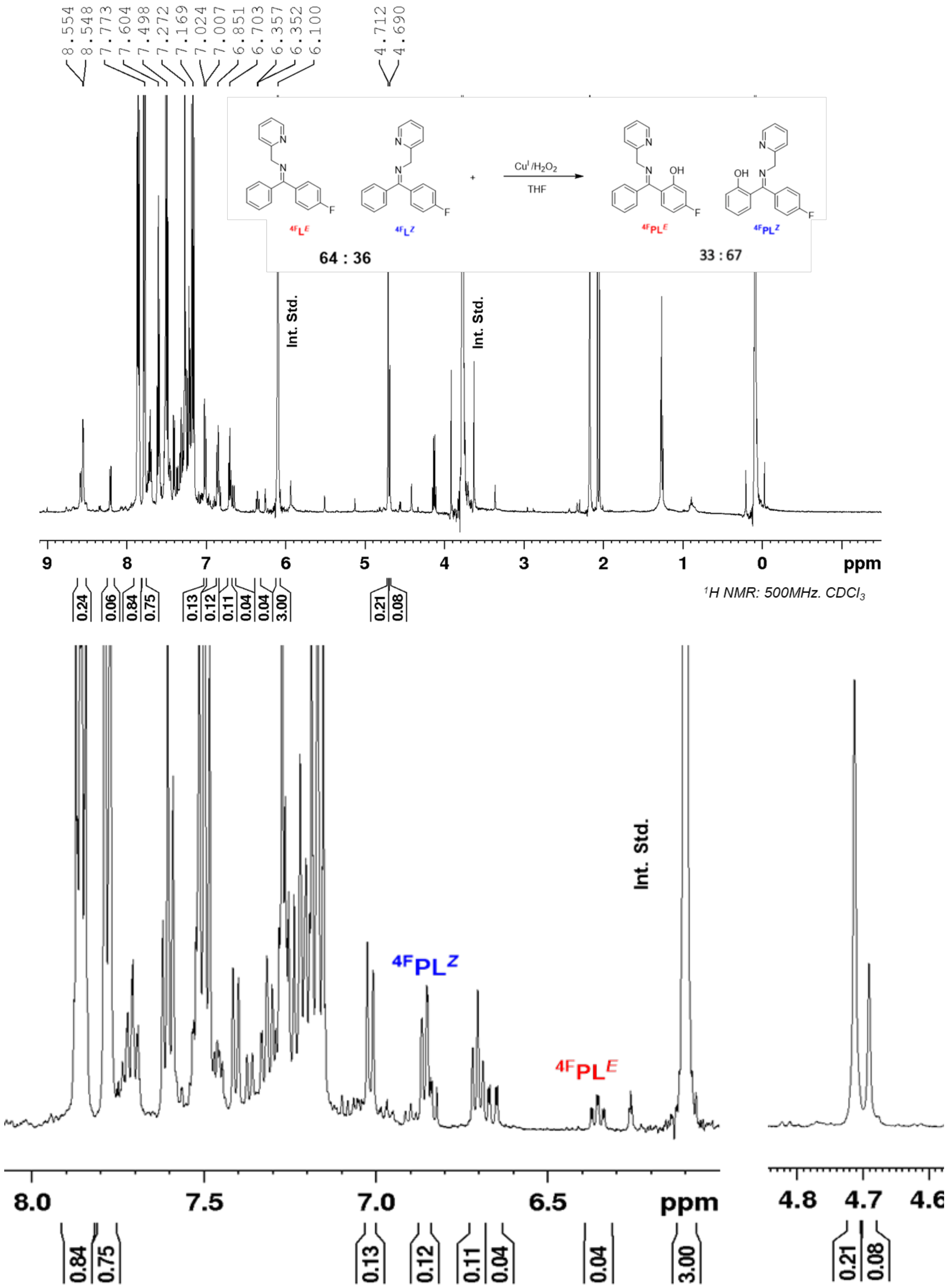

Figure S148. ^{1}H-NMR spectra for the hydroxylation of [4F]L with Cu[I] and H_2O_2 in THF.

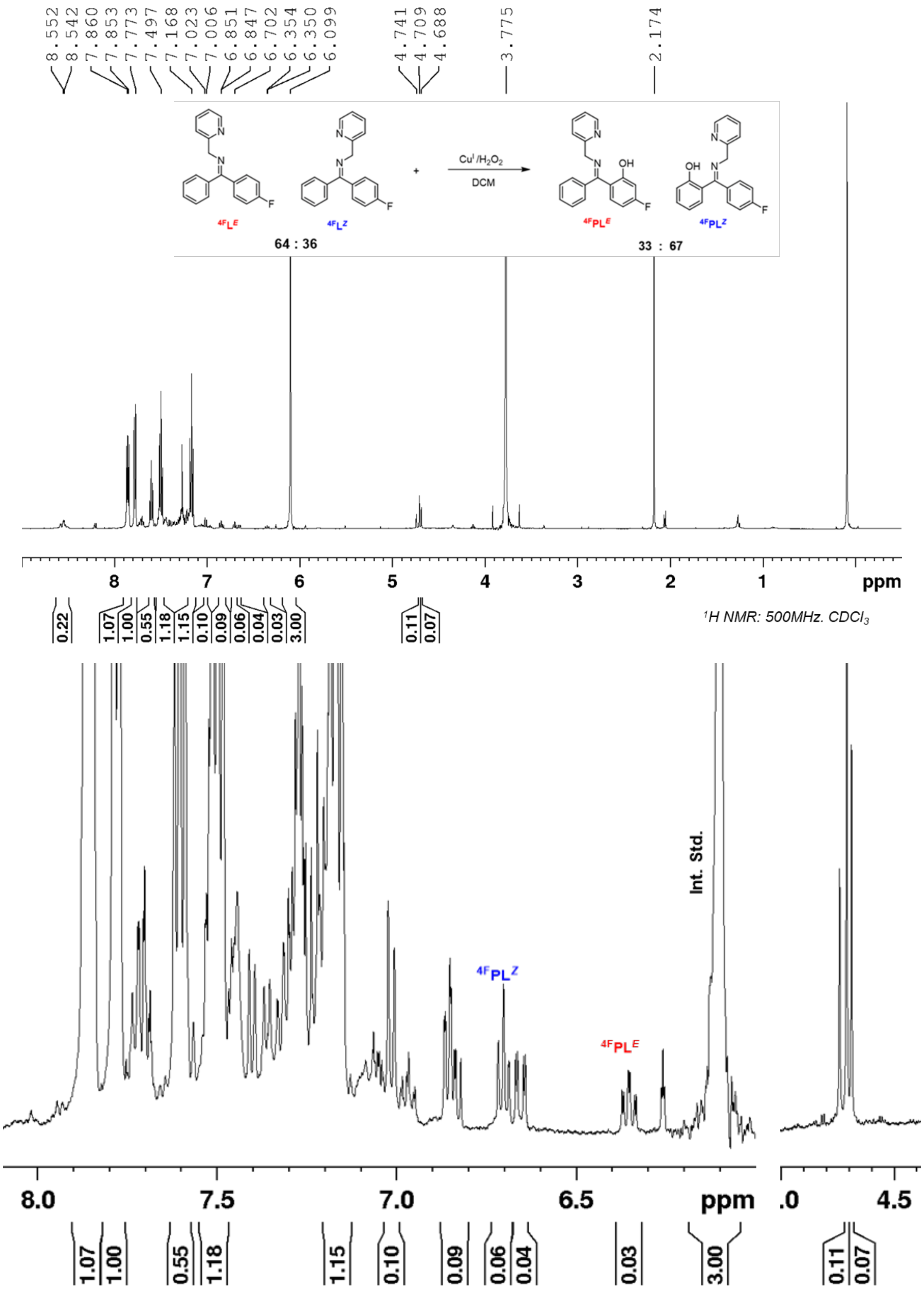

Figure S149. ^{1}H-NMR spectra for the hydroxylation of ^{4F}L with Cu^{I} and H_2O_2 in CH_2Cl_2.

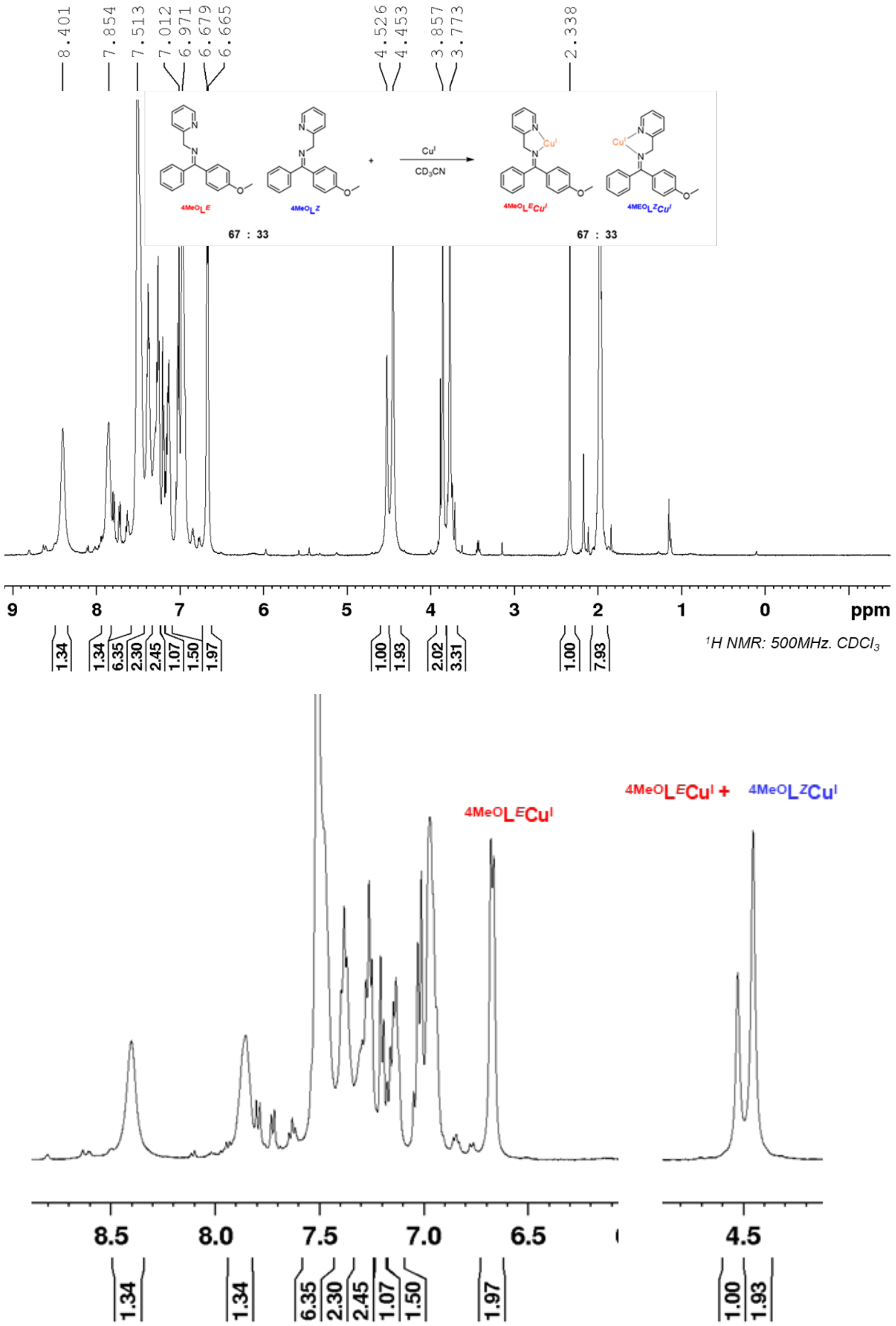

Figure S150. ^{1}H-NMR spectra of ^{4MeO}L upon addition of Cu^I in CD_3CN.

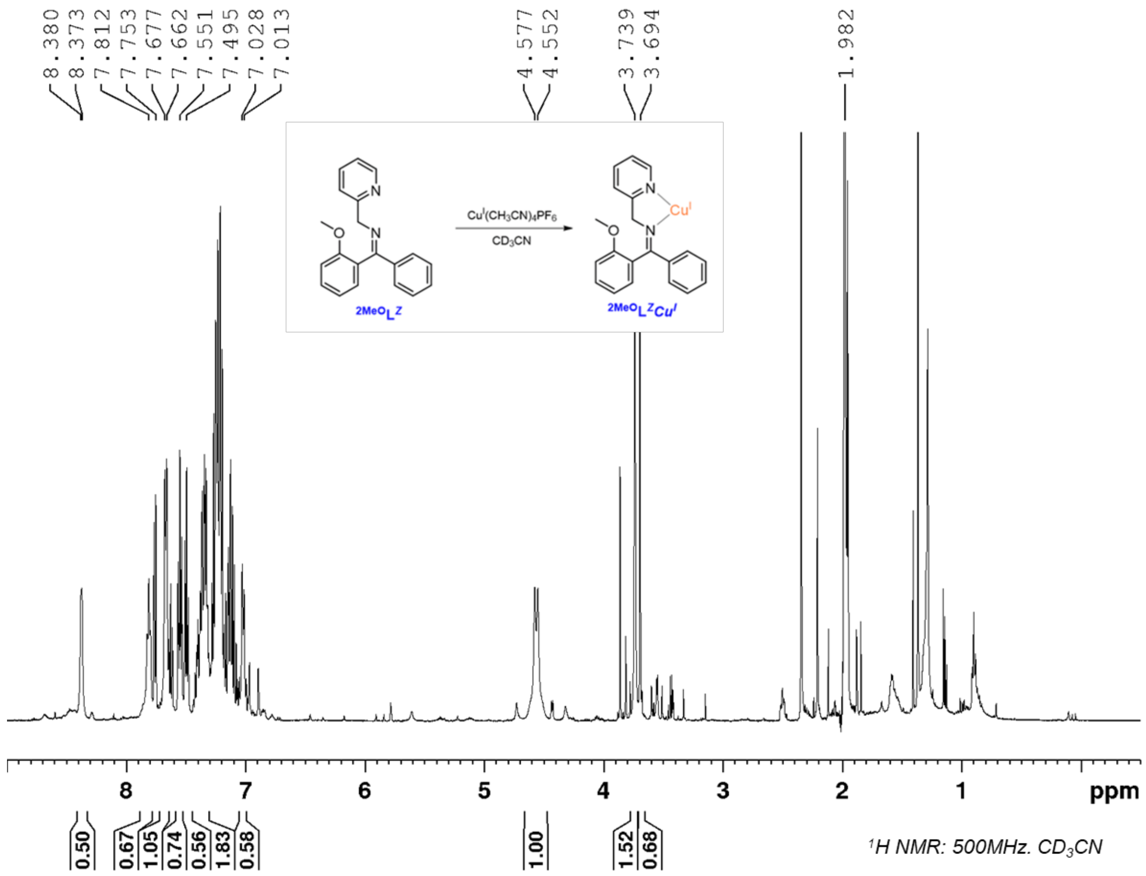

Figure S151. ^{1}H-NMR spectra of ^{2MeO}L upon addition of Cu^I in CD_3CN.

5. Mass balance experiments

In order to analyze the mass balance loss (15-30%), we independently synthesized **^{4MeO}PL^{E}** following our standard synthetic protocol (Dean-Stark) and we evaluated the mass loss upon:

i) Dissolving **^{4MeO}PL^{E}** in acetone (0.159 mmols of imine in 4 mL of acetone) and performing the workup of the reaction (i.e. Na_2EDTA + EtOAc);

ii) Dissolving **^{4MeO}PL^{E}** in acetone (0.159 mmols of imine in 4 mL of acetone), adding 1 equiv of $[Cu^{I}(CH_3CN)_4](PF_6)$ and performing the workup of the reaction (i.e. Na_2EDTA + EtOAc);

iii) Dissolving **^{4MeO}PL^{E}** in acetone (0.159 mmols of imine in 4 mL of acetone), adding 1 equiv of $Cu^{II}(NO_3)_2{\cdot}3H_2O$ and performing the workup of the reaction (i.e. Na_2EDTA + EtOAc);

iv) Dissolving **^{4MeO}PL^{E}** in acetone (0.159 mmols of imine in 4 mL of acetone), adding 1 equiv of $[Cu^{I}(CH_3CN)_4](PF_6)$ and 5 equiv of H_2O_2 and performing the workup of the reaction (i.e. Na_2EDTA + EtOAc);

The organic phases were separated, combined, dried over $MgSO_4$, filtered, and dried under vacuum. The reaction products were dissolved in 1.4 mL of $CDCl_3$ solution containing 27.1 mg of 1,3,5-trimethoxybenzene (internal standard). The reaction products were quantified by ^{1}H-NMR using integration signals that correspond to the starting material and products with the integration signal of the internal standard.

For all the experiments abovementioned, we recovered **^{4MeO}PL^{E}** with mass balances ranging from 62% to 80%, which is consistent with the mass balances observed in the hydroxylation of **^{4MeO}L** (81%).

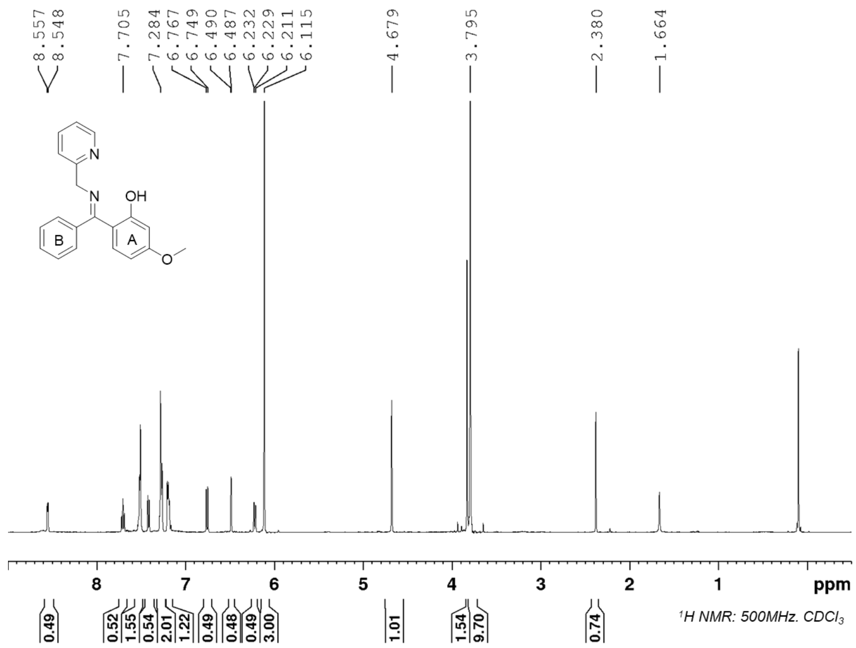

Figure S152. ^{1}H-NMR spectrum of **^{4MeO}PL^{E}** in $CDCl_3$.

6. Electrochemistry

Cyclic voltammetry: 3 mL of an CH_3CN solution containing 1 mM Cu complex (Note: the cuprous complexes were generated in situ via addition of 1 equiv of $[Cu^I(CH_3CN)_4](PF_6)$ to the corresponding imine substrate-ligand) and NBu_4PF_6 (100 mM) were prepared in the glovebox and were transferred to an electrochemical cell outside the glovebox, which has been purged with Ar for 10 minutes (note: a conventional three-electrode cell was used with a glassy carbon working electrode, an $Ag/AgNO_3$ (0.01 M in CH_3CN) and a platinum wire as the counter-electrode). The potentials were measured with respect to the $Ag/AgNO_3$ reference electrode and converted to $Fc^{0/+}$ ($Fc^{0/+}$ potential measured under the same experimental conditions). Cyclic voltammograms were obtained at a scan rate of 0.1 V/s. All electrochemical measurements were carried out under an Ar atmosphere.

Table S2. Redox potential measurements for the cuprous complexes bearing imine substrate-ligands derived from 4-substituted and 4,4'-disubstituted benzophenones.

Complex	$E_{1/2}$ (mV vs $Fc^{0/+)}$	Separation between anodic and cathodic peaks in mV
$[^{4MeO}LCu^I]^{1+}$	-116	110
$[^{4Me}LCu^I]^{1+}$	-88	106
$[^{4H}LCu^I]^{1+}$	-70	109
$[^{4F}LCu^I]^{1+}$	-42	125
$[^{4Cl}LCu^I]^{1+}$	0.0	150
$[^{4Br}LCu^I]^{1+}$	-17	160
$[^{4,4'\text{-}(MeO)2}LCu^I]^{1+}$	-160	110
$[^{4MeO4'Me}LCu^I]^{1+}$	-138	105
$[^{4MeO4'F}LCu^I]^{1+}$	-67	115
$[^{4MeO4'Cl}LCu^I]^{1+}$	-60	115
$[^{4MeO4'Br}LCu^I]^{1+}$	-53	110

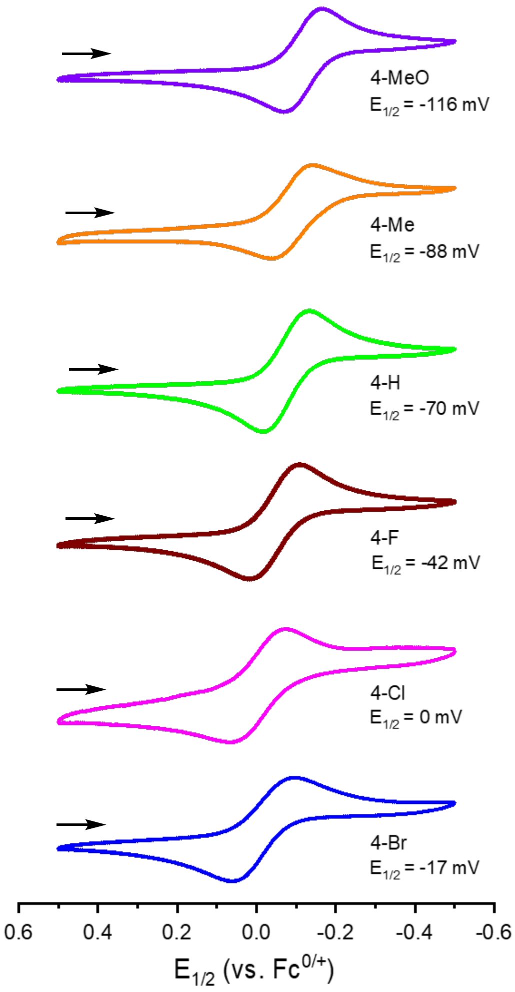

Figure S153. Cyclic voltammetry measurements the cuprous complexes bearing imine substrate-ligands derived from 4-substituted benzophenones. Note: Polarographic CV convention. Note: Working electrode: glassy carbon; counter electrode: platinum wire; and reference electrode: $Ag/AgNO_3$ (0.01 M in CH_3CN). Note: Measurements made in CH_3CN with 1 mM Cu complex and NBu_4PF_6 as electrolyte (100 mM) and at room temperature. Note: Starting point (0.5 V); direction of scan (from 0.5 to -0.5 V).

7. ^{13}C-NMR data for imine substrate-ligands

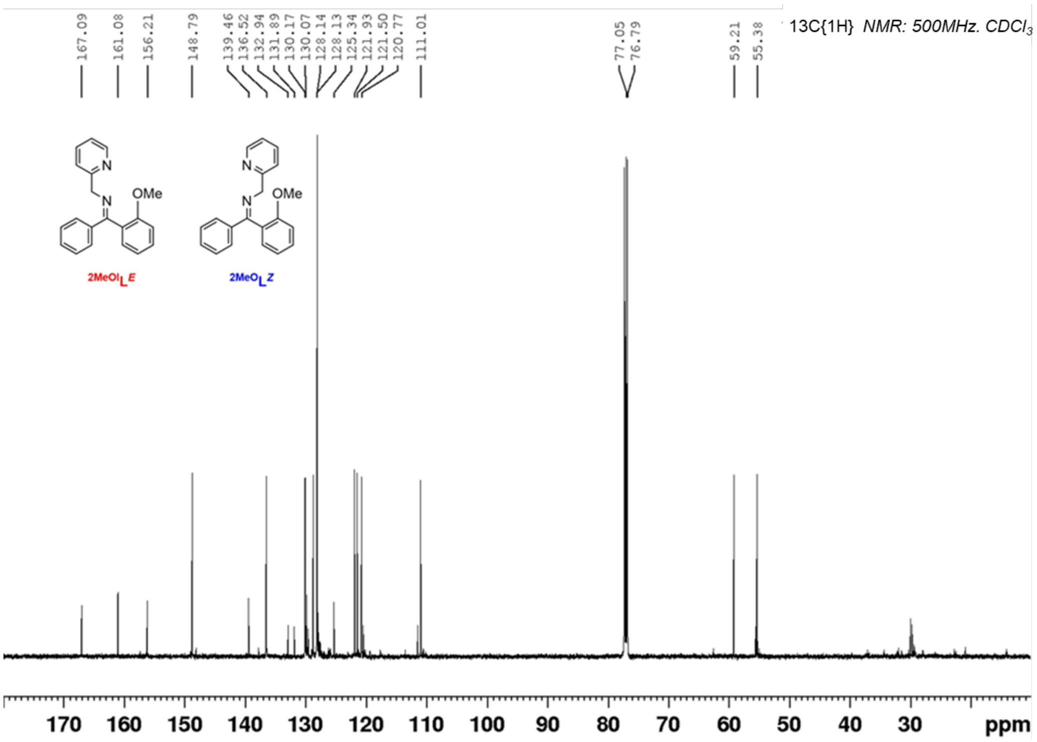

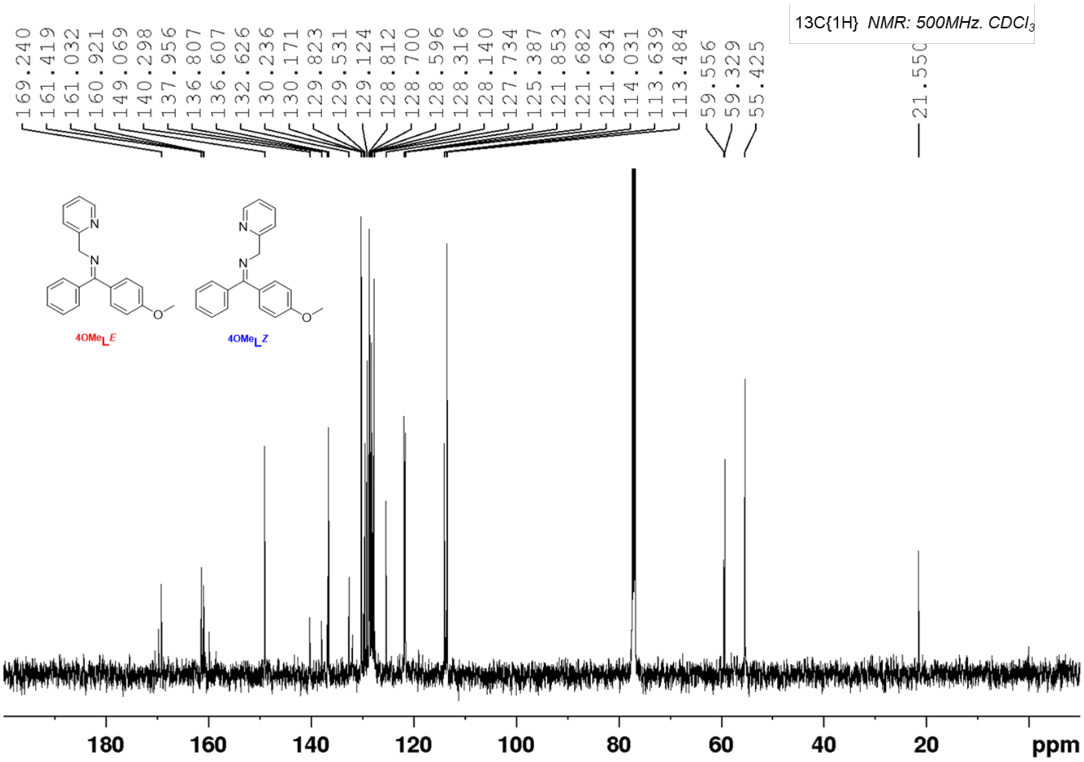

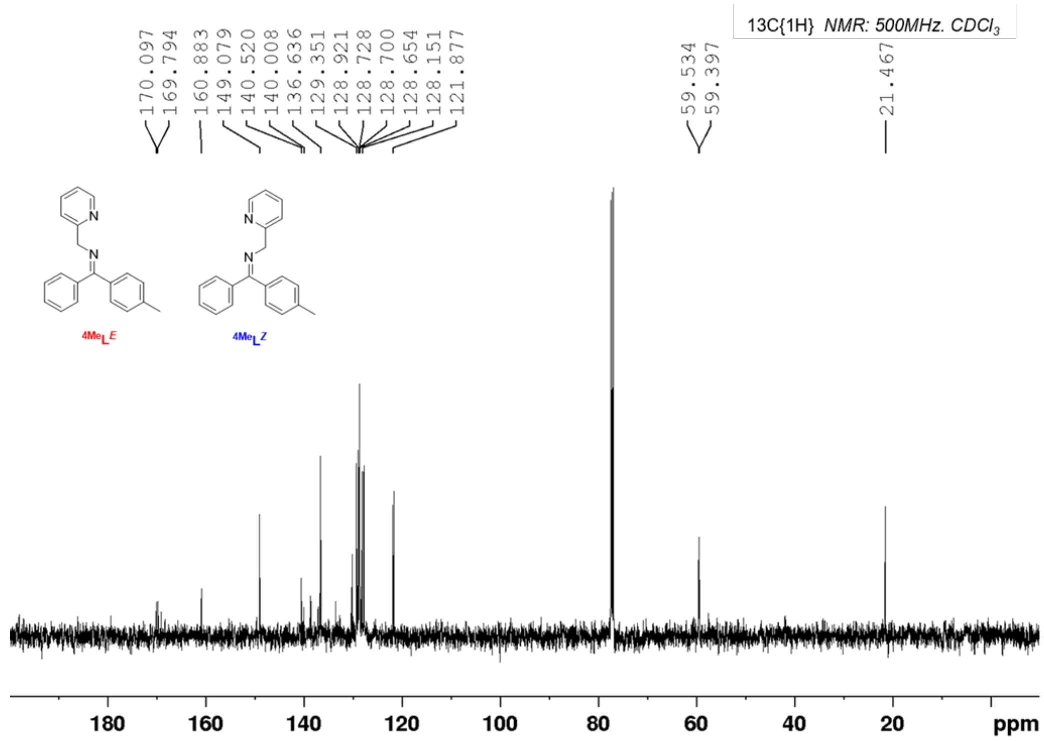
13C{1H} NMR: 500MHz. CDCl3
170.097
169.794
160.883
149.079
140.520
140.008
136.636
129.351
128.921
128.728
128.700
128.654
128.151
121.877
59.534
59.397
21.467
N
N
N
N
4MeL E
4MeL Z
180
160
140
120
100
80
60
40
20
ppm

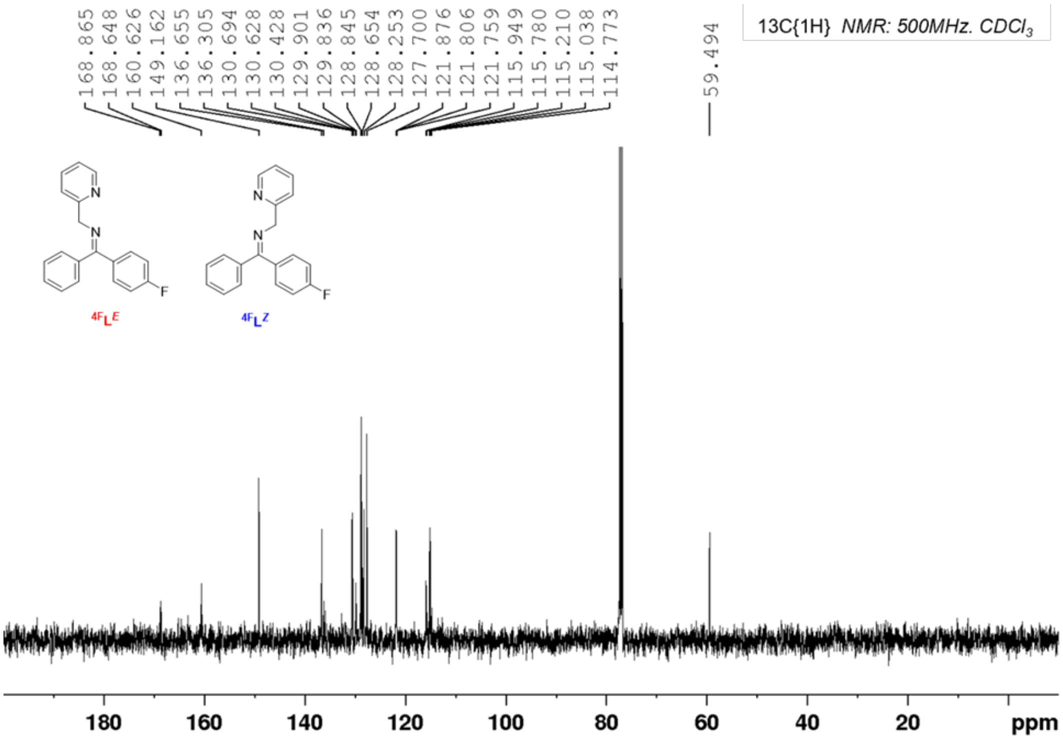
13C{1H} NMR: 500MHz. CDCl3
168.865
168.648
160.626
149.162
136.655
136.305
130.694
130.628
130.428
129.901
129.836
128.845
128.654
128.253
127.700
121.876
121.806
121.759
115.949
115.780
115.210
115.038
114.773
59.494
N
N
N
N
F
F
4FL E
4FL Z
180
160
140
120
100
80
60
40
20
ppm

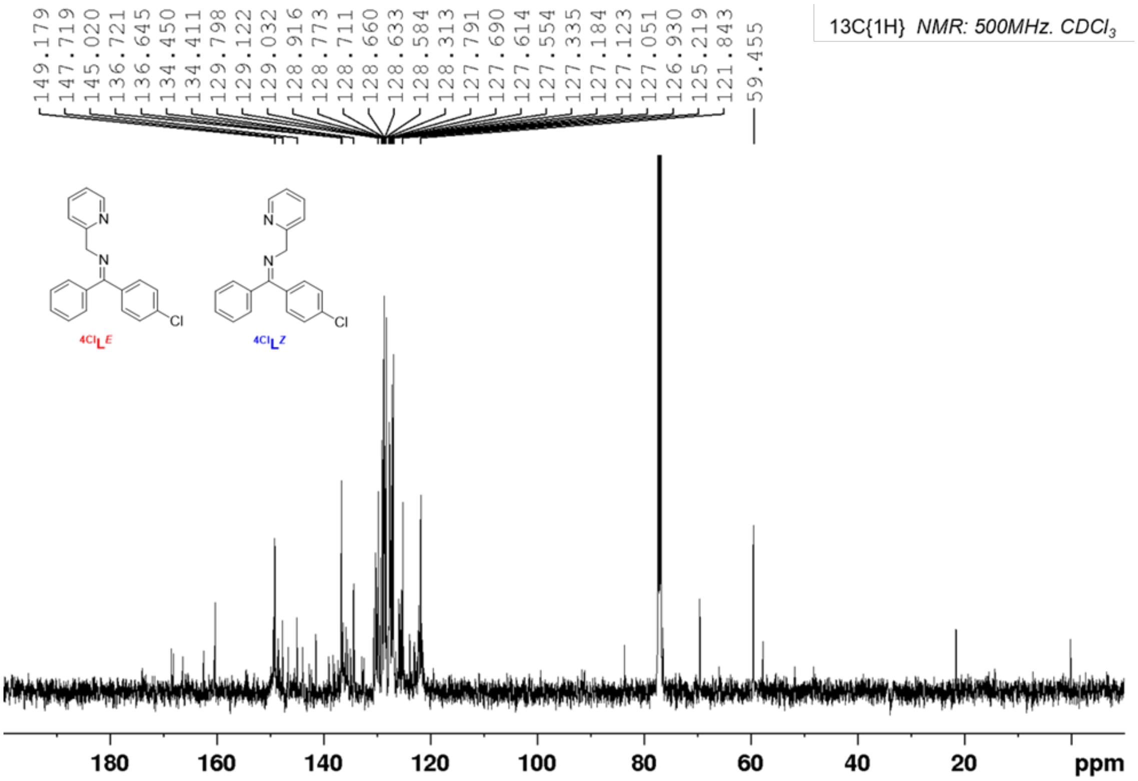
149.179
147.719
145.020
136.721
136.645
134.450
134.411
129.798
129.122
129.032
128.916
128.773
128.711
128.660
128.633
128.584
128.313
127.791
127.690
127.614
127.554
127.335
127.184
127.123
127.051
126.930
125.219
121.843
59.455
13C{1H} NMR: 500MHz. CDCl3
N
N
Cl
4ClL E
N
N
Cl
4ClL Z
180
160
140
120
100
80
60
40
20
ppm

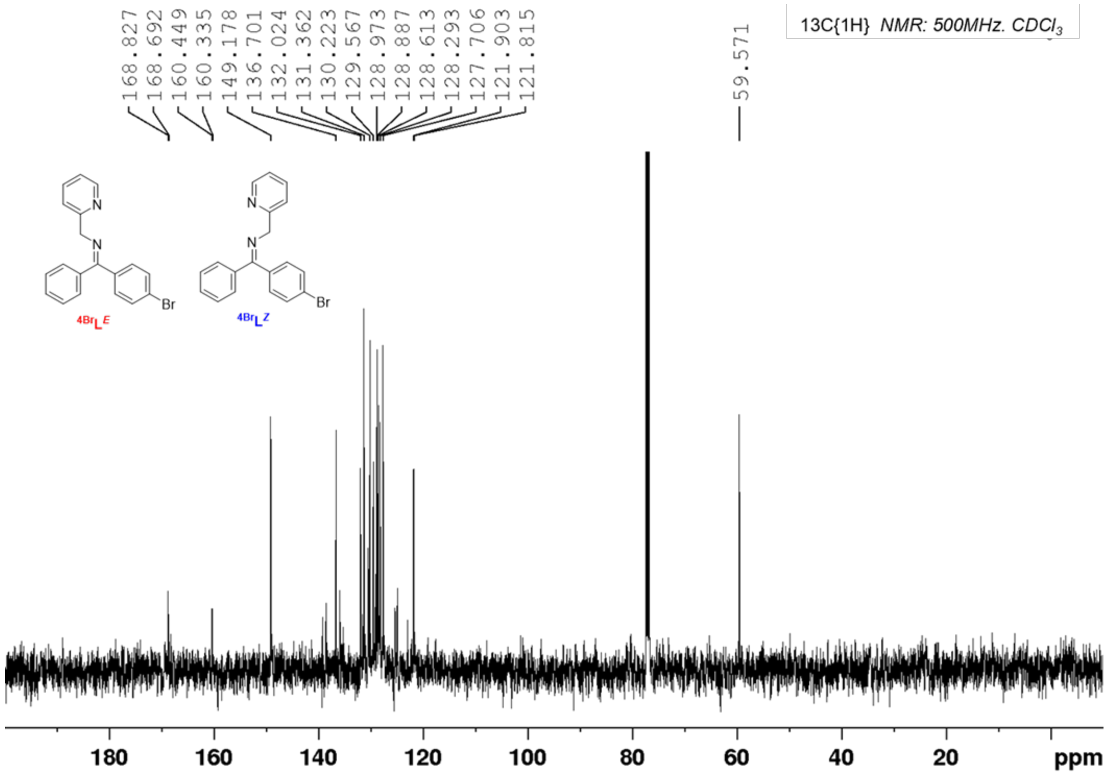
168.827
168.692
160.449
160.335
149.178
136.701
132.024
131.362
130.223
129.567
128.973
128.887
128.613
128.293
127.706
121.903
121.815
59.571
13C{1H} NMR: 500MHz. CDCl3
N
N
Br
4BrL E
N
N
Br
4BrL Z
180
160
140
120
100
80
60
40
20
ppm

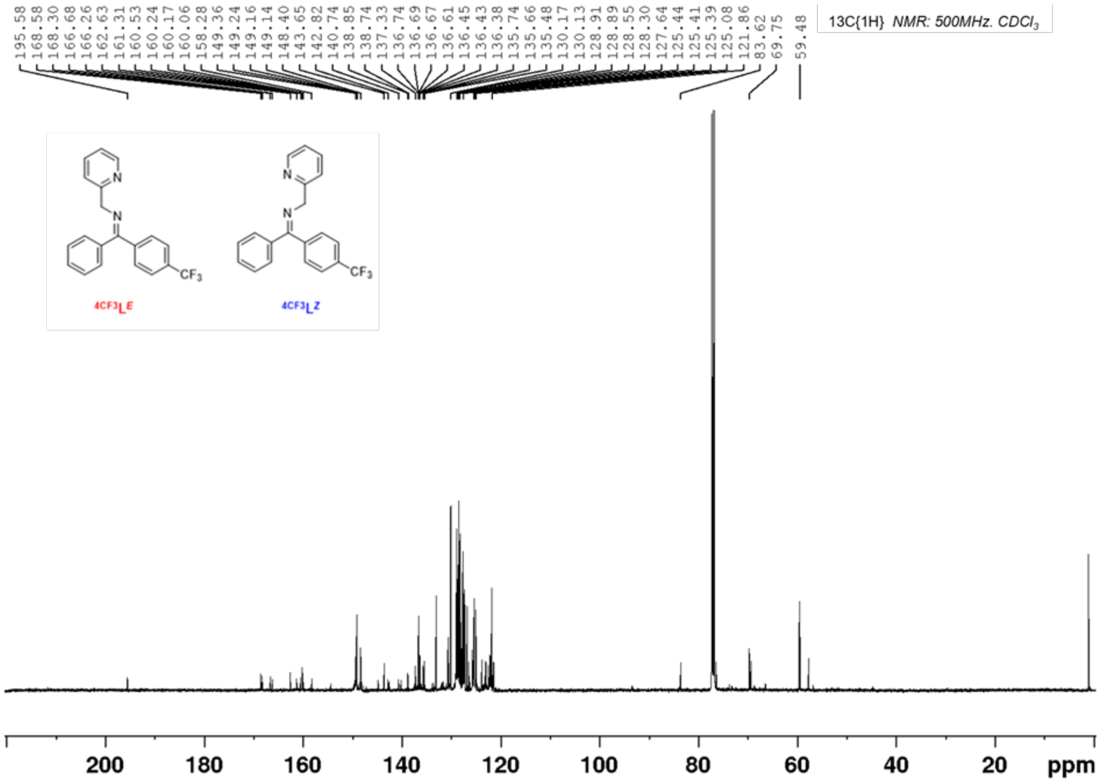
13C{1H} NMR: 500MHz. CDCl3
4CF3L E
4CF3L Z
ppm

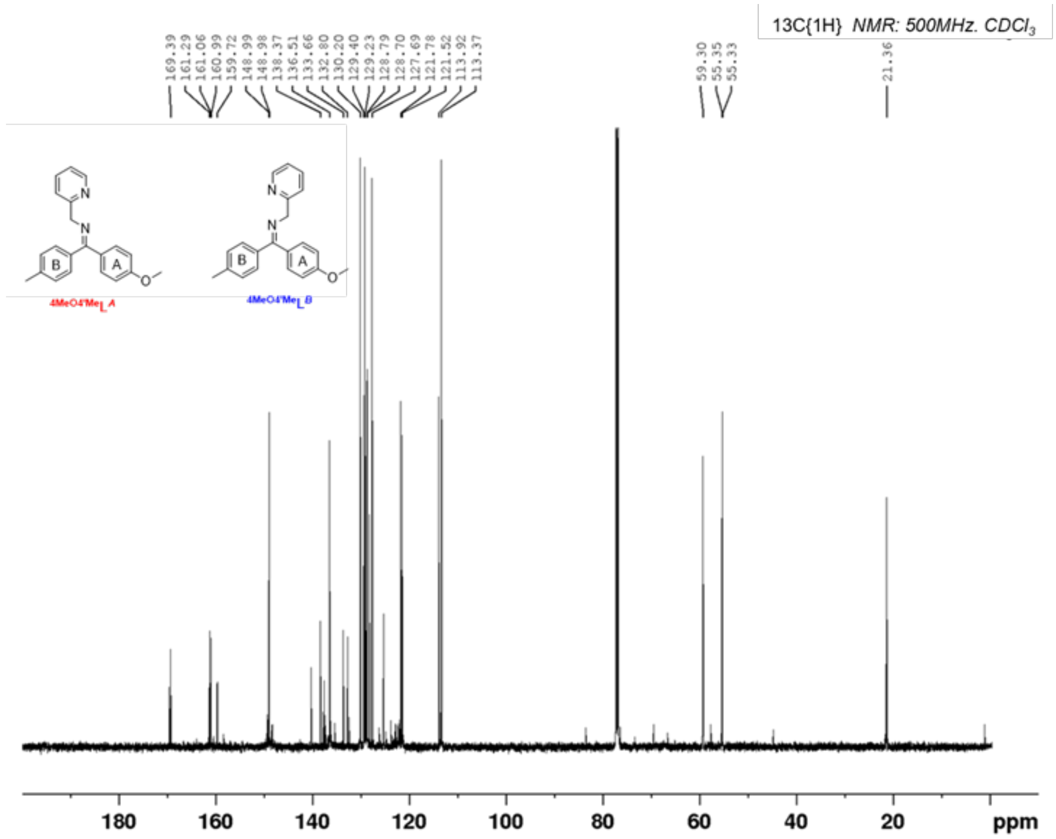
13C{1H} NMR: 500MHz. CDCl3
4MeO4'MeL A
4MeO4'MeL B
ppm

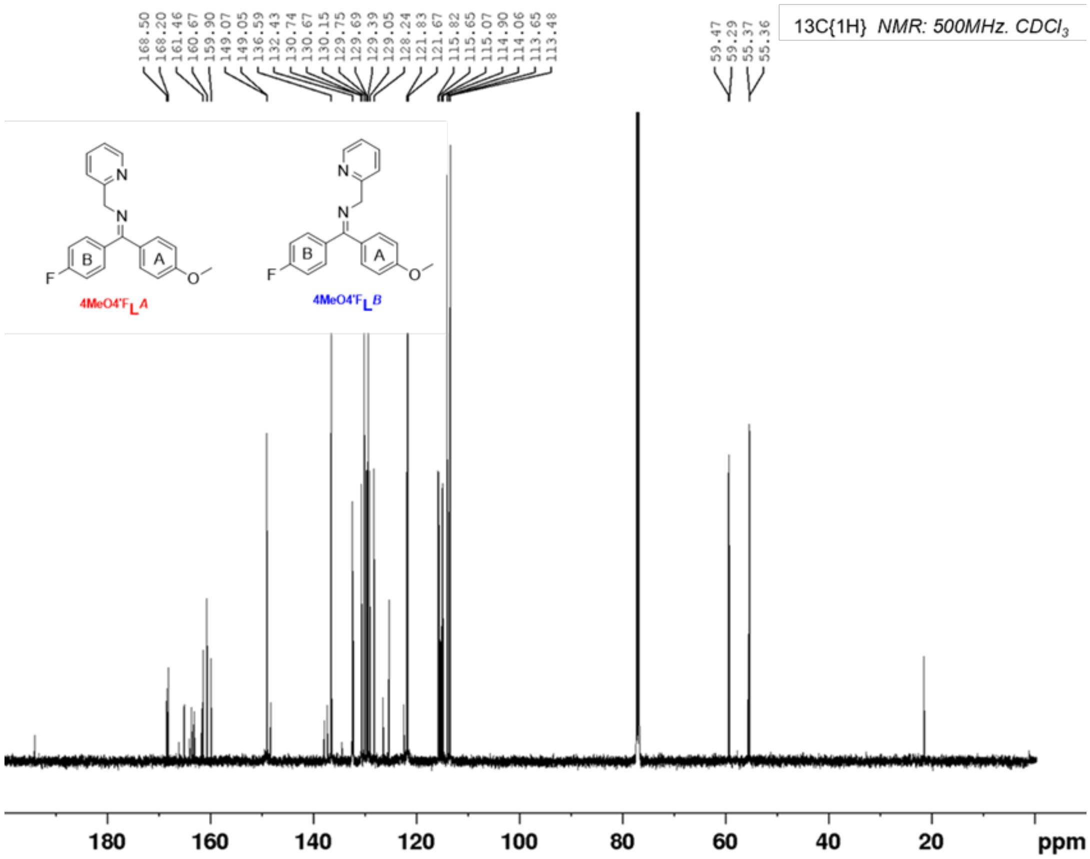
168.50
168.20
161.46
160.67
159.90
149.07
149.05
136.59
132.43
130.74
130.67
130.15
129.75
129.69
129.39
129.05
128.24
121.83
121.67
115.82
115.65
115.07
114.90
114.06
113.65
113.48
59.47
59.29
55.37
55.36
13C{1H} NMR: 500MHz. CDCl3
4MeO4'FL A
4MeO4'FL B
180
160
140
120
100
80
60
40
20
ppm

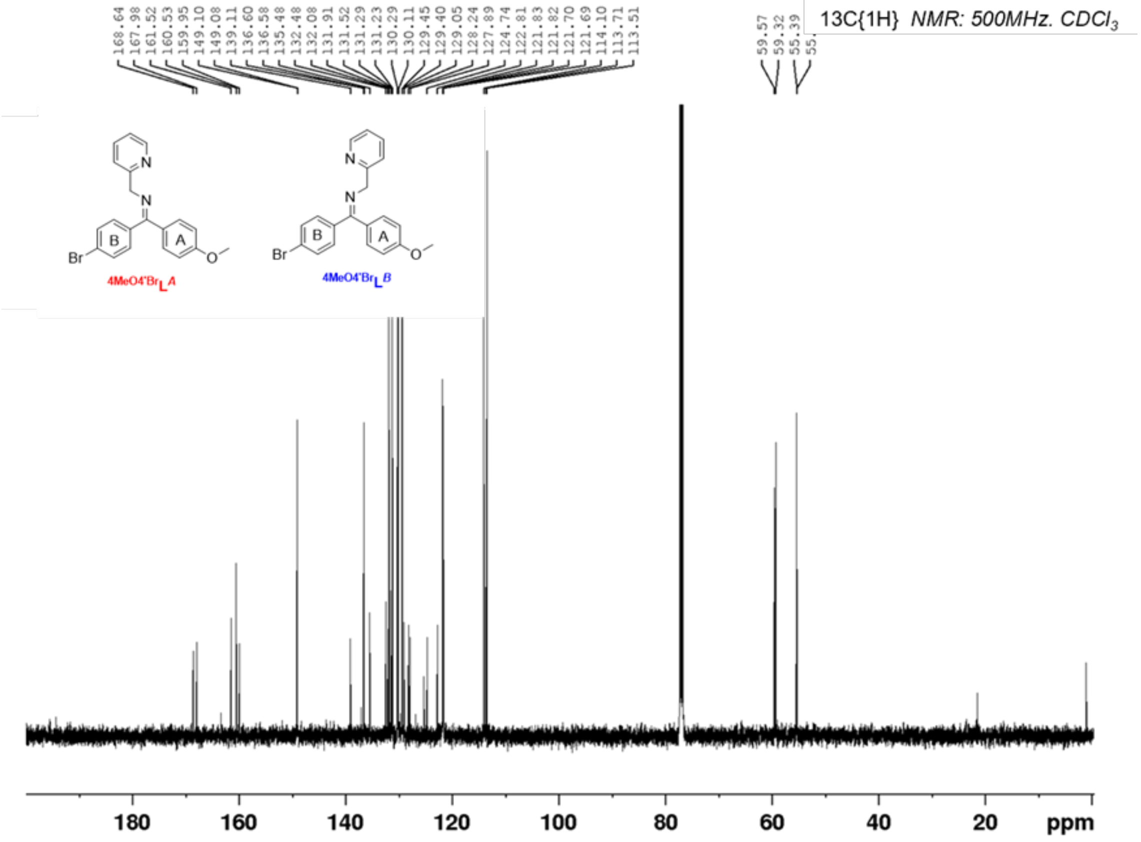
168.64
167.98
161.52
160.53
159.95
149.10
149.08
139.11
136.60
136.58
135.48
132.48
132.08
131.91
131.52
131.29
131.23
130.29
130.11
129.45
129.40
129.05
128.24
127.89
124.74
122.81
121.83
121.82
121.70
121.69
114.10
113.71
113.51
59.57
59.32
55.39
13C{1H} NMR: 500MHz. CDCl3
4MeO4'BrL A
4MeO4'BrL B
180
160
140
120
100
80
60
40
20
ppm

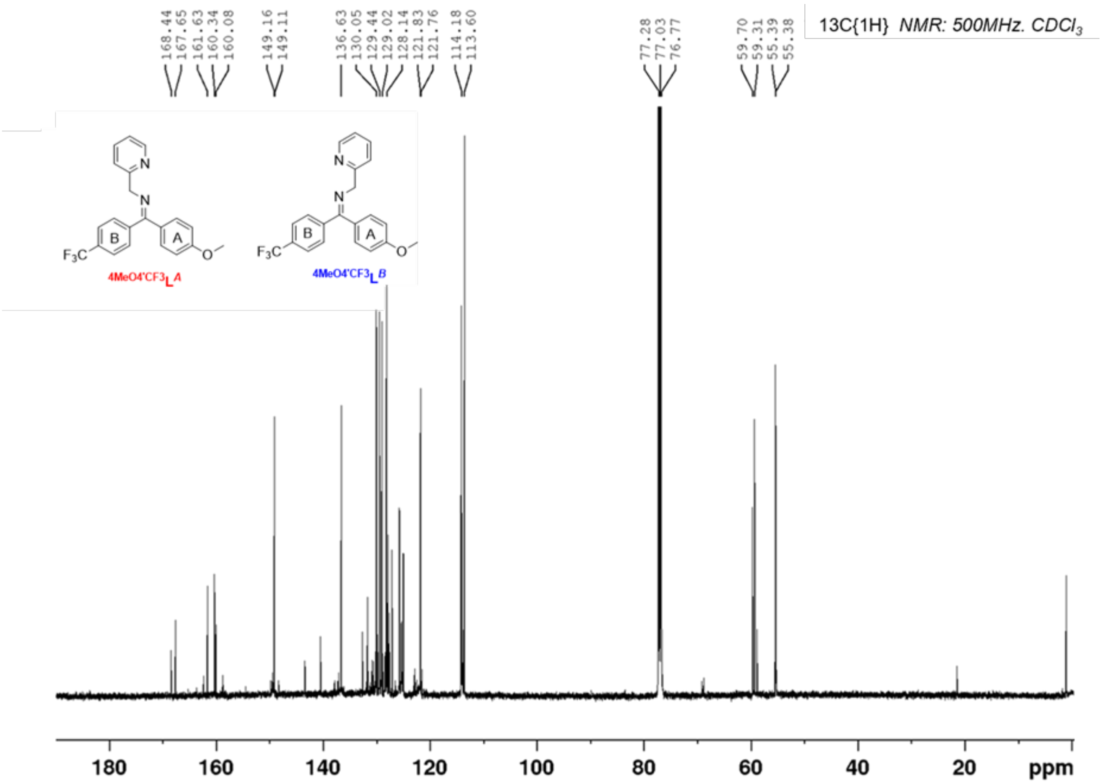
13C{1H} NMR: 500MHz. CDCl3
168.44
167.65
161.63
160.34
160.08
149.16
149.11
136.63
130.05
129.44
129.02
128.14
121.83
121.76
114.18
113.60
77.28
77.03
76.77
59.70
59.31
55.39
55.38
4MeO4'CF3LA
4MeO4'CF3LB
180
160
140
120
100
80
60
40
20
ppm

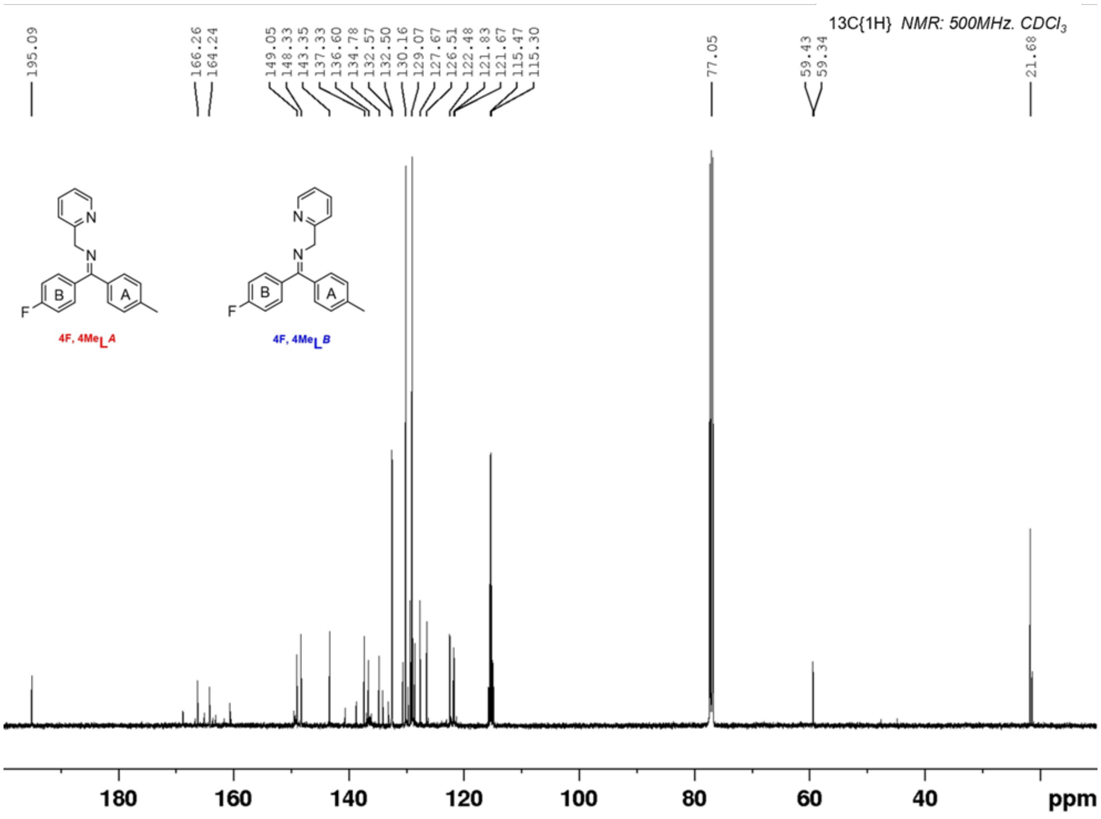
13C{1H} NMR: 500MHz. CDCl3
195.09
166.26
164.24
149.05
148.33
143.35
137.33
136.60
134.78
132.57
132.50
130.16
129.07
127.67
126.51
122.48
121.83
121.67
115.47
115.30
77.05
59.43
59.34
21.68
4F, 4MeLA
4F, 4MeLB
180
160
140
120
100
80
60
40
ppm

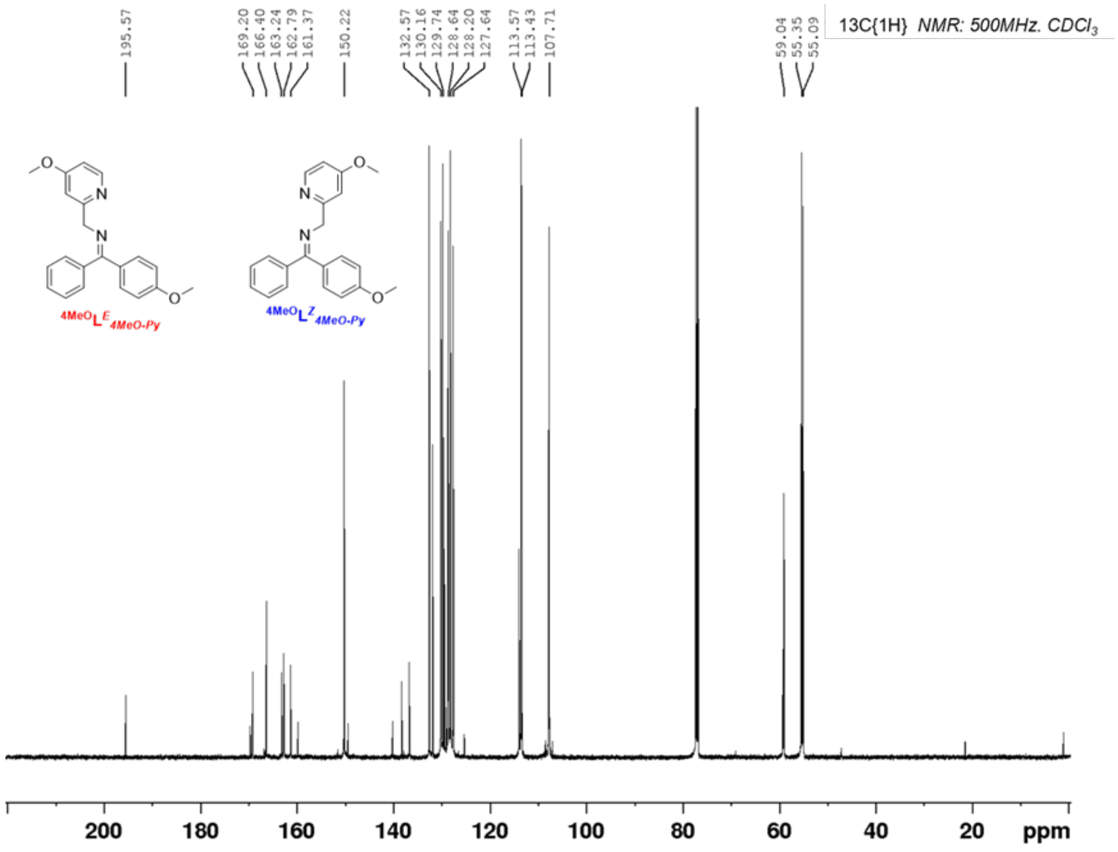

195.57
169.20
166.40
163.24
162.79
161.37
150.22
132.57
130.16
129.74
128.64
128.20
127.64
113.57
113.43
107.71
59.04
55.35
55.09
13C{1H} NMR: 500MHz. CDCl3
4MeOLE4MeO-Py
4MeOLZ4MeO-Py
200
180
160
140
120
100
80
60
40
20
ppm

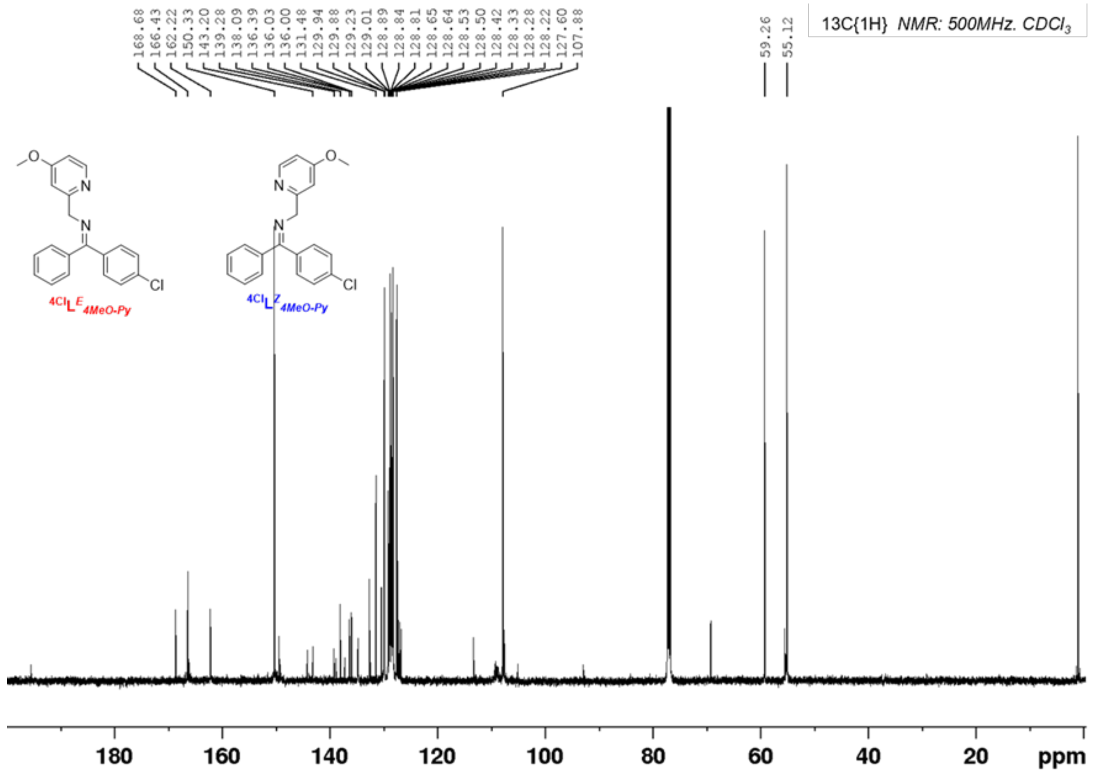

168.68
166.43
162.22
150.33
143.20
139.28
138.09
136.39
136.03
136.00
131.48
129.94
129.88
129.23
129.01
128.89
128.84
128.81
128.65
128.64
128.53
128.50
128.42
128.33
128.28
128.22
127.60
107.88
59.26
55.12
13C{1H} NMR: 500MHz. CDCl3
4ClLE4MeO-Py
4ClLZ4MeO-Py
180
160
140
120
100
80
60
40
20
ppm

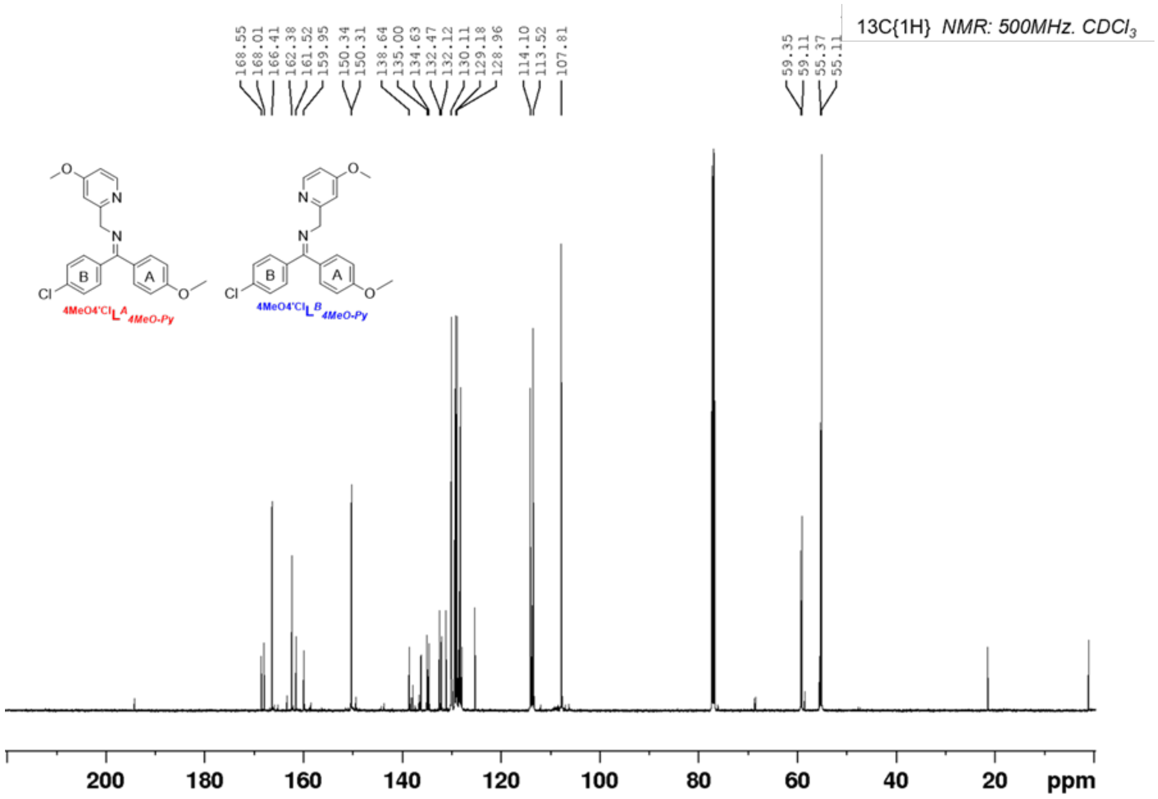
168.55
168.01
166.41
162.38
161.52
159.95
150.34
150.31
138.64
135.00
134.63
132.47
132.12
130.11
129.18
128.96
114.10
113.52
107.81
59.35
59.11
55.37
55.11
13C{1H} NMR: 500MHz. CDCl3
4MeO4'ClL^A 4MeO-Py
4MeO4'ClL^B 4MeO-Py
200
180
160
140
120
100
80
60
40
20
ppm

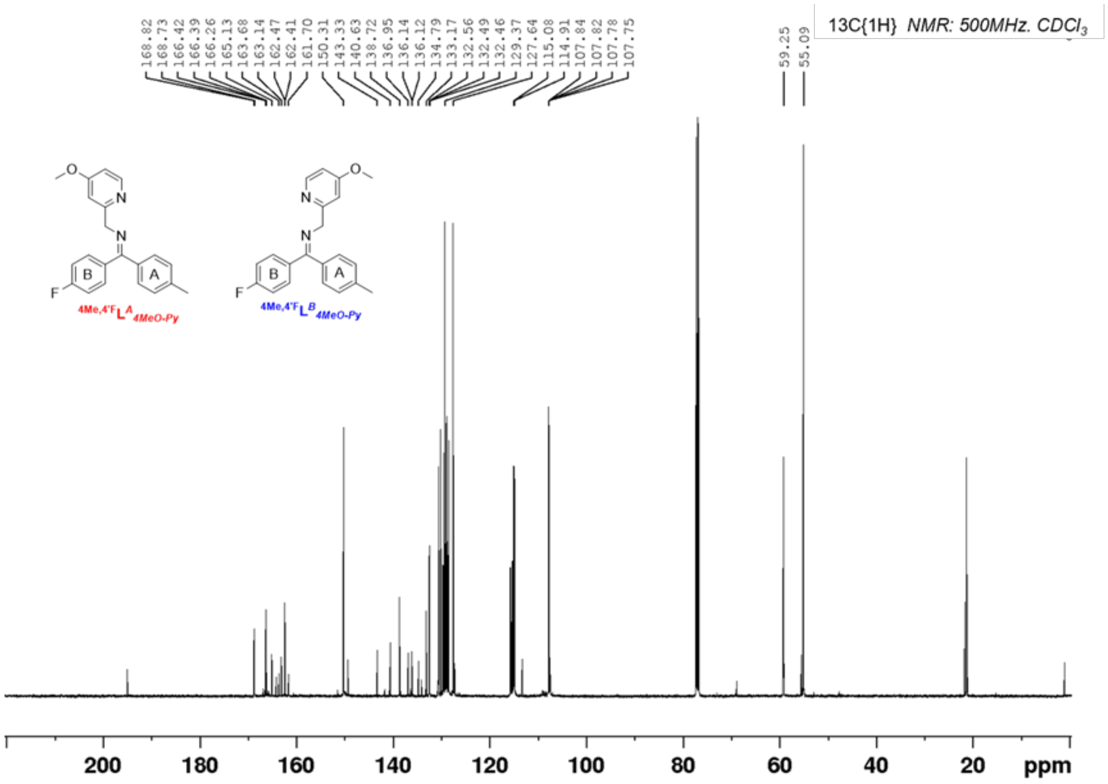
168.82
168.73
166.42
166.39
166.26
165.13
163.68
163.14
162.47
162.41
161.70
150.31
143.33
140.63
138.72
136.95
136.14
136.12
134.79
133.17
132.56
132.49
132.46
129.37
127.64
115.08
114.91
107.84
107.82
107.78
107.75
59.25
55.09
13C{1H} NMR: 500MHz. CDCl3
4Me,4'FL^A 4MeO-Py
4Me,4'FL^B 4MeO-Py
200
180
160
140
120
100
80
60
40
20
ppm

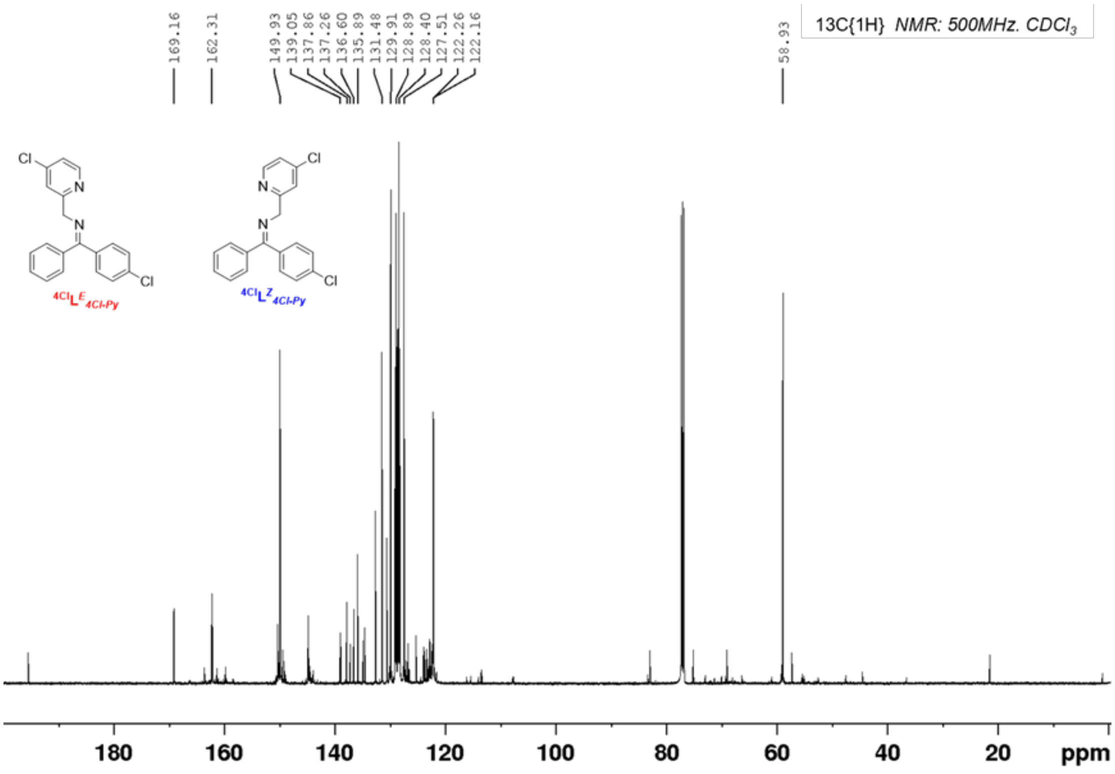
13C{1H} NMR: 500MHz. CDCl3
169.16
162.31
149.93
139.05
137.86
137.26
136.60
135.89
131.48
129.91
128.89
128.40
127.51
122.26
122.16
58.93
Cl
N
Cl
Cl
N
Cl
180
160
140
120
100
80
60
40
20
ppm

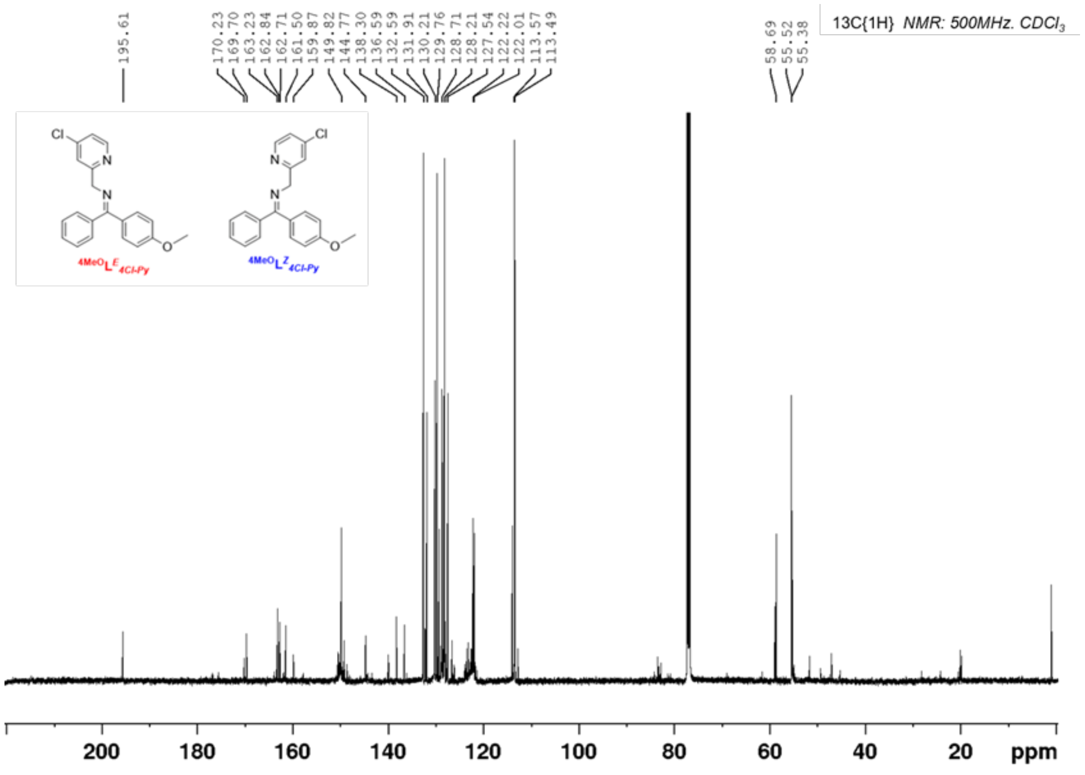
13C{1H} NMR: 500MHz. CDCl3
195.61
170.23
169.70
163.23
162.84
162.71
161.50
159.87
149.82
144.77
138.30
136.59
132.59
131.91
130.21
129.76
128.71
128.21
127.54
122.22
122.01
113.57
113.49
58.69
55.52
55.38
Cl
N
O
Cl
N
O
200
180
160
140
120
100
80
60
40
20
ppm

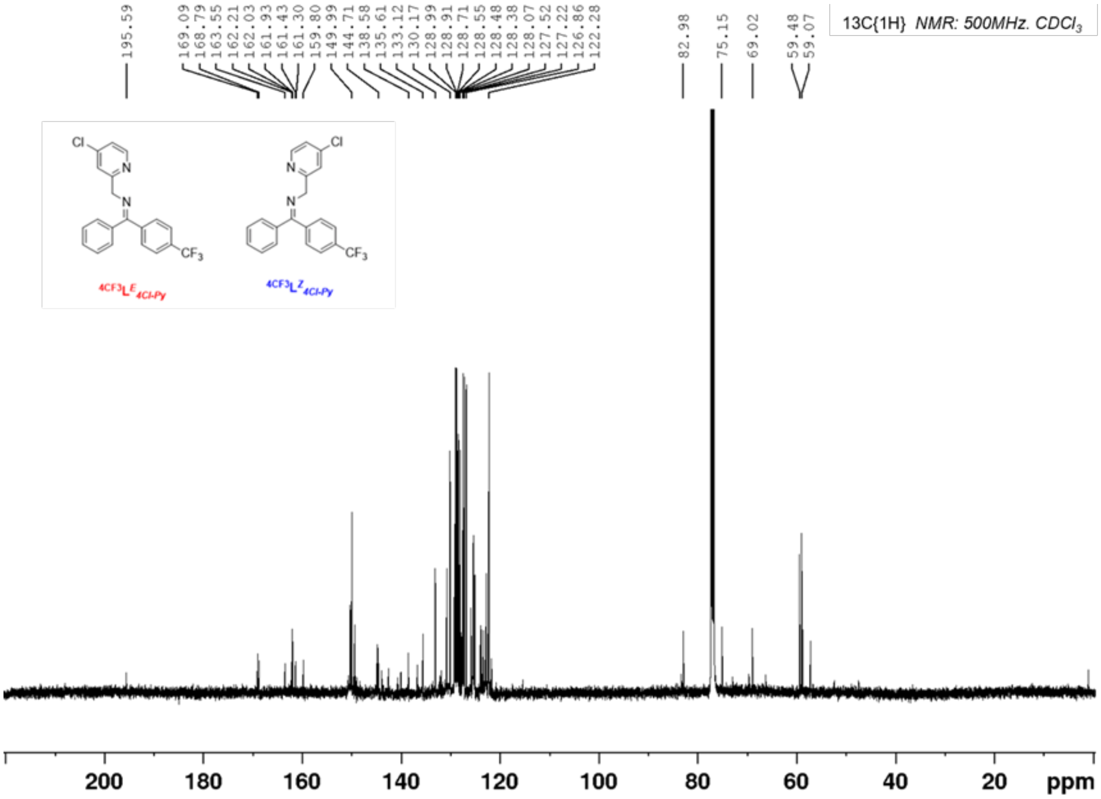
195.59
169.09
168.79
163.55
162.21
162.03
161.93
161.43
161.30
159.80
149.99
144.71
138.58
135.61
133.12
130.17
128.99
128.91
128.71
128.55
128.48
128.38
128.07
127.52
127.22
126.86
122.28
82.98
75.15
69.02
59.48
59.07
13C{1H} NMR: 500MHz. CDCl3
Cl
N
CF3
4CF3L E 4Cl-Py
4CF3L Z 4Cl-Py
200
180
160
140
120
100
80
60
40
20
ppm

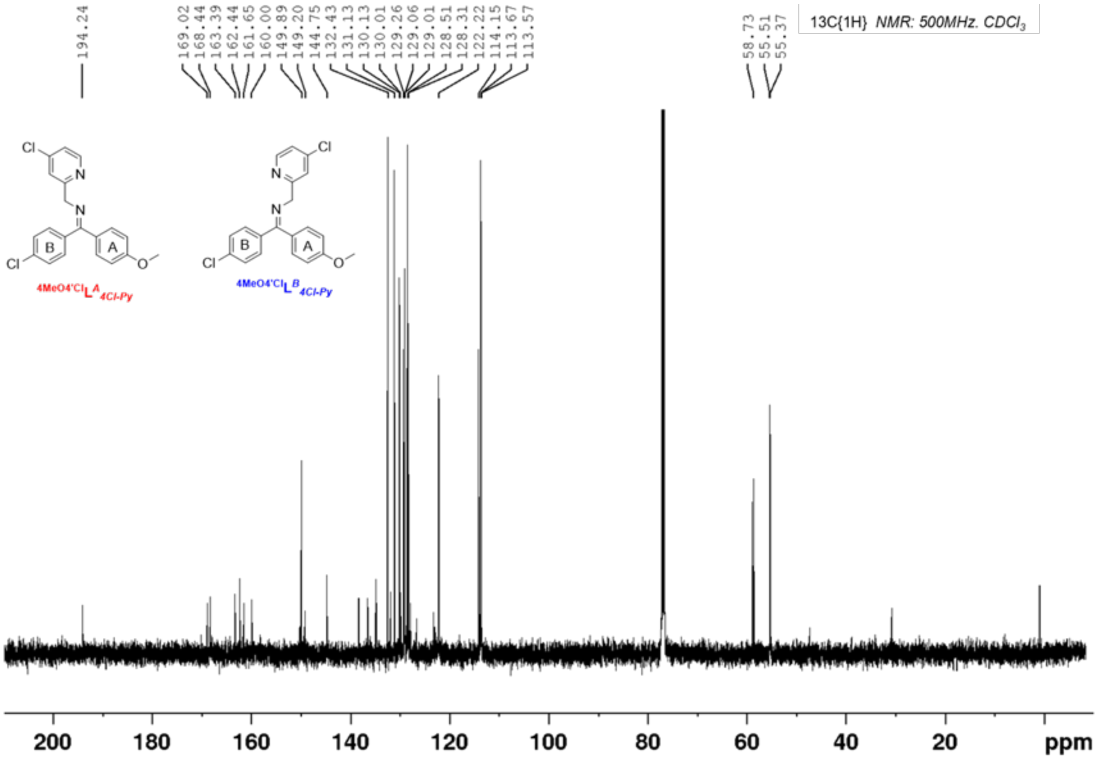
194.24
169.02
168.44
163.39
162.44
161.65
160.00
149.89
149.20
144.75
132.43
131.13
130.13
130.01
129.26
129.06
129.01
128.51
128.31
122.22
114.15
113.67
113.57
58.73
55.51
55.37
13C{1H} NMR: 500MHz. CDCl3
Cl
N
B
A
O
4MeO4'ClL A 4Cl-Py
4MeO4'ClL B 4Cl-Py
200
180
160
140
120
100
80
60
40
20
ppm

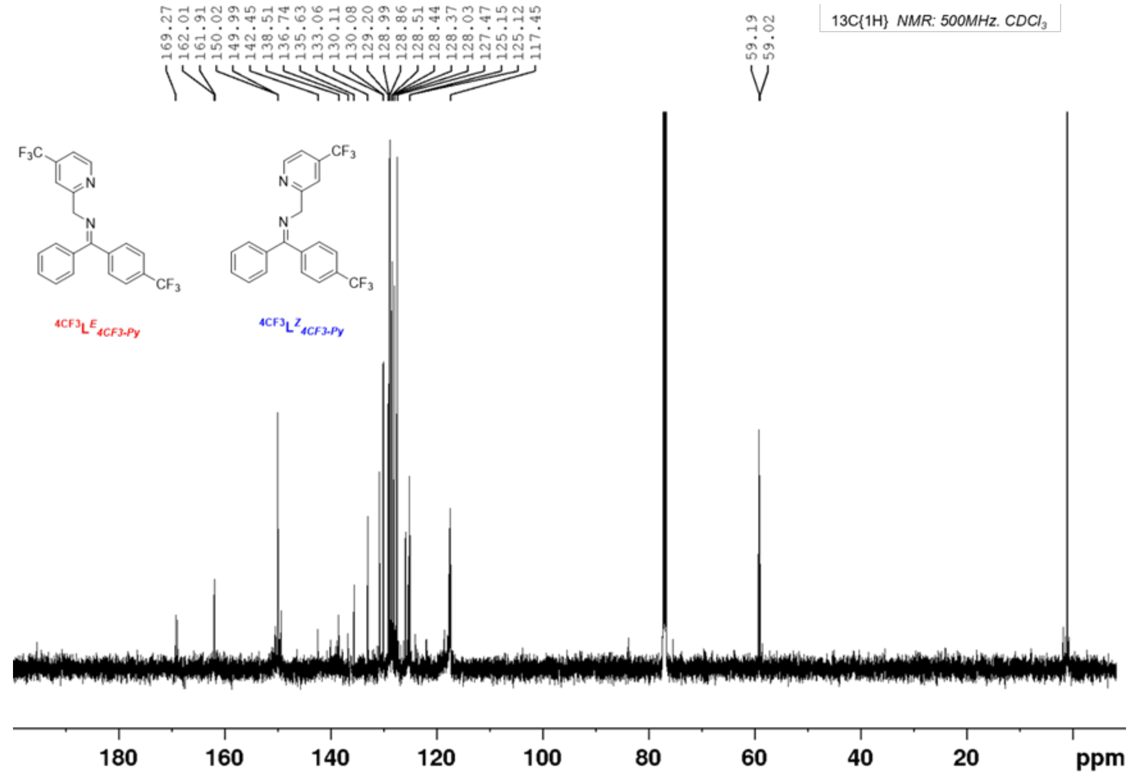

169.27
162.01
161.91
150.02
149.99
142.45
138.51
136.74
135.63
133.06
130.11
130.08
129.20
128.99
128.86
128.51
128.44
128.37
128.03
127.47
125.15
125.12
117.45
59.19
59.02
13C{1H} NMR: 500MHz. CDCl3
4CF3L E 4CF3-Py
4CF3L Z 4CF3-Py
180
160
140
120
100
80
60
40
20
ppm

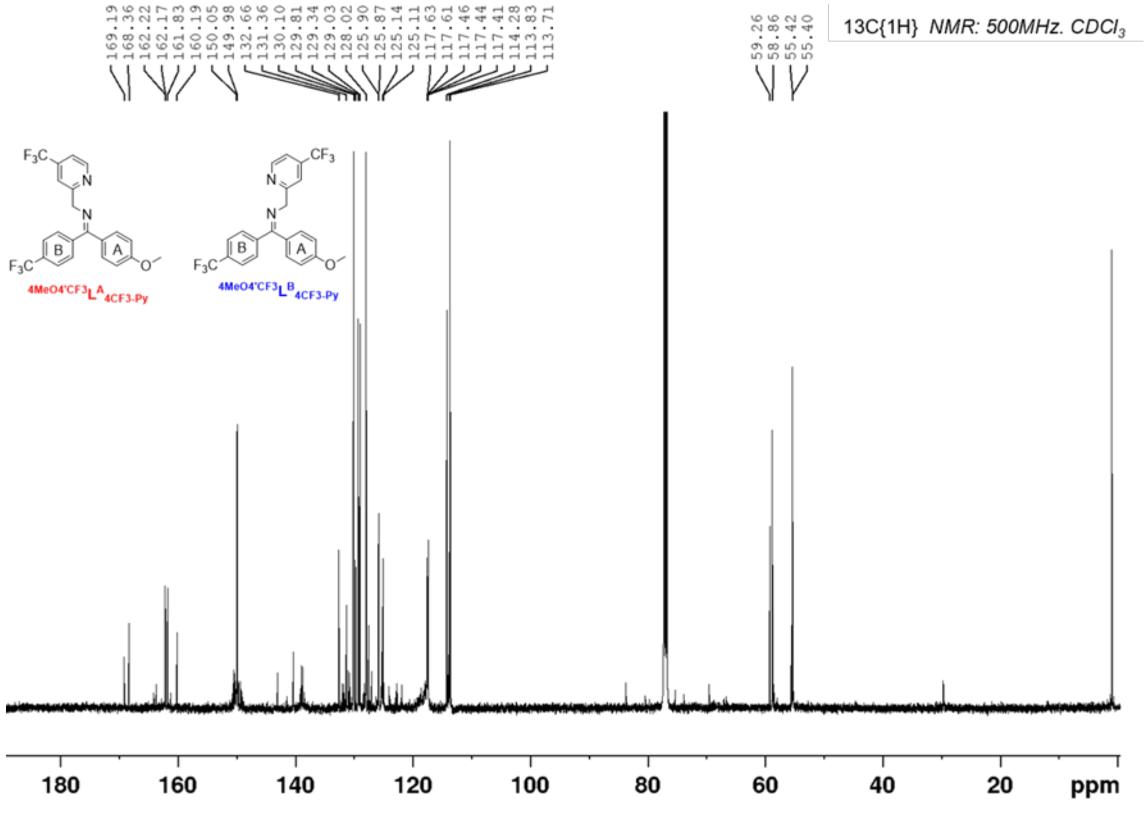

169.19
168.36
162.22
162.17
161.83
160.19
150.05
149.98
132.66
131.36
130.10
129.81
129.34
129.03
128.02
125.90
125.87
125.14
125.11
117.63
117.61
117.46
117.44
117.41
114.28
113.83
113.71
59.26
58.86
55.42
55.40
13C{1H} NMR: 500MHz. CDCl3
4MeO4'CF3L A 4CF3-Py
4MeO4'CF3L B 4CF3-Py
180
160
140
120
100
80
60
40
20
ppm

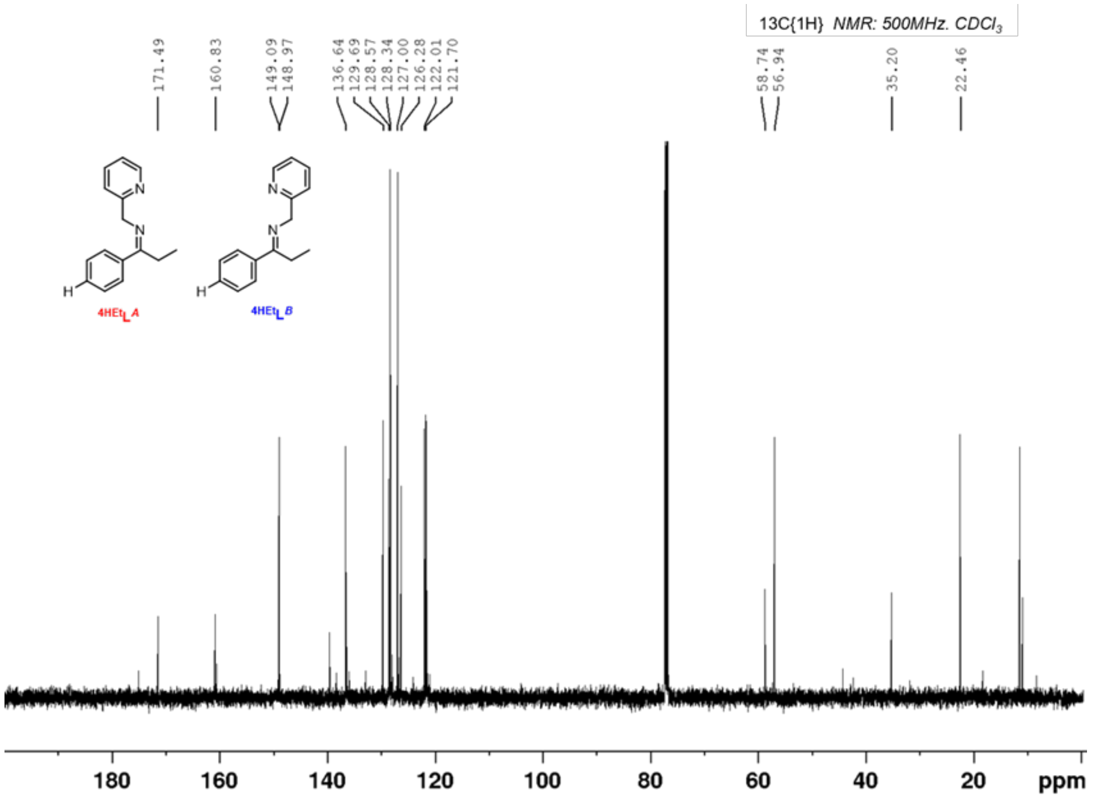
13C{1H} NMR: 500MHz. CDCl3
171.49
160.83
149.09
148.97
136.64
129.69
128.57
128.34
127.00
126.28
122.01
121.70
58.74
56.94
35.20
22.46
4HEtL A
4HEtL B
180
160
140
120
100
80
60
40
20
ppm

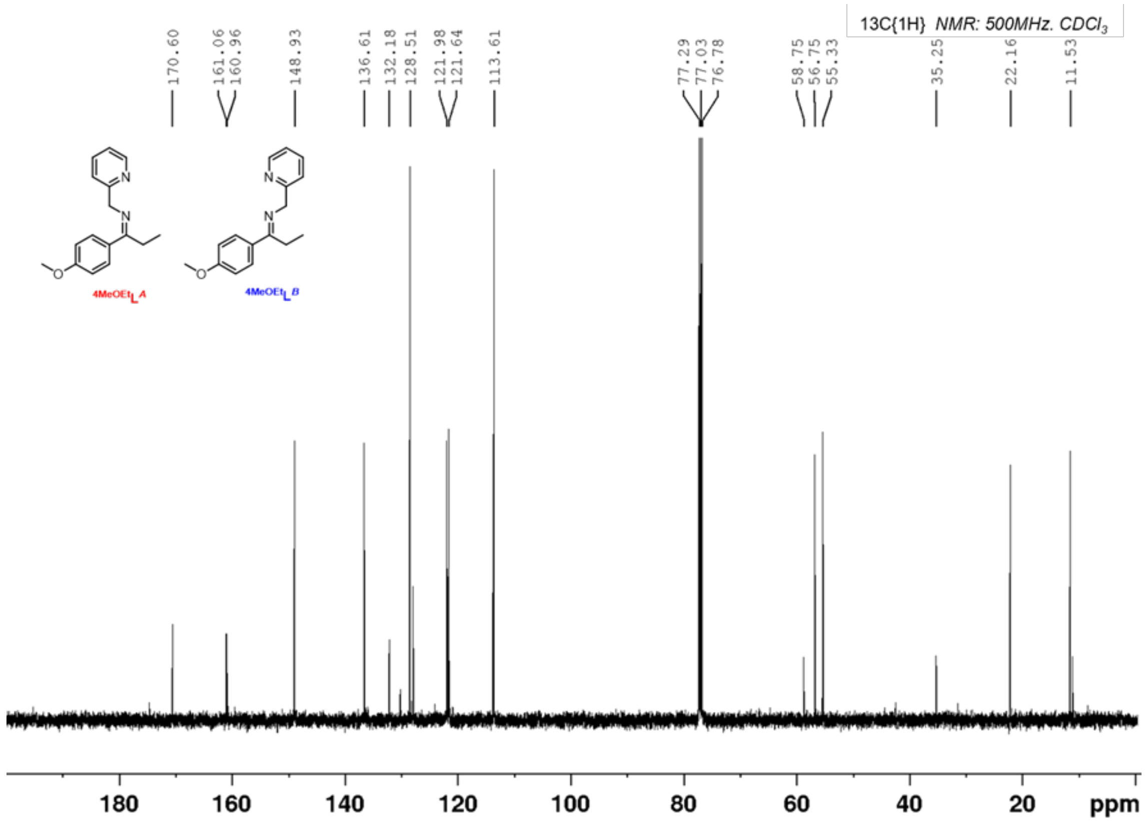
13C{1H} NMR: 500MHz. CDCl3
170.60
161.06
160.96
148.93
136.61
132.18
128.51
121.98
121.64
113.61
77.29
77.03
76.78
58.75
56.75
55.33
35.25
22.16
11.53
4MeOEtL A
4MeOEtL B
180
160
140
120
100
80
60
40
20
ppm

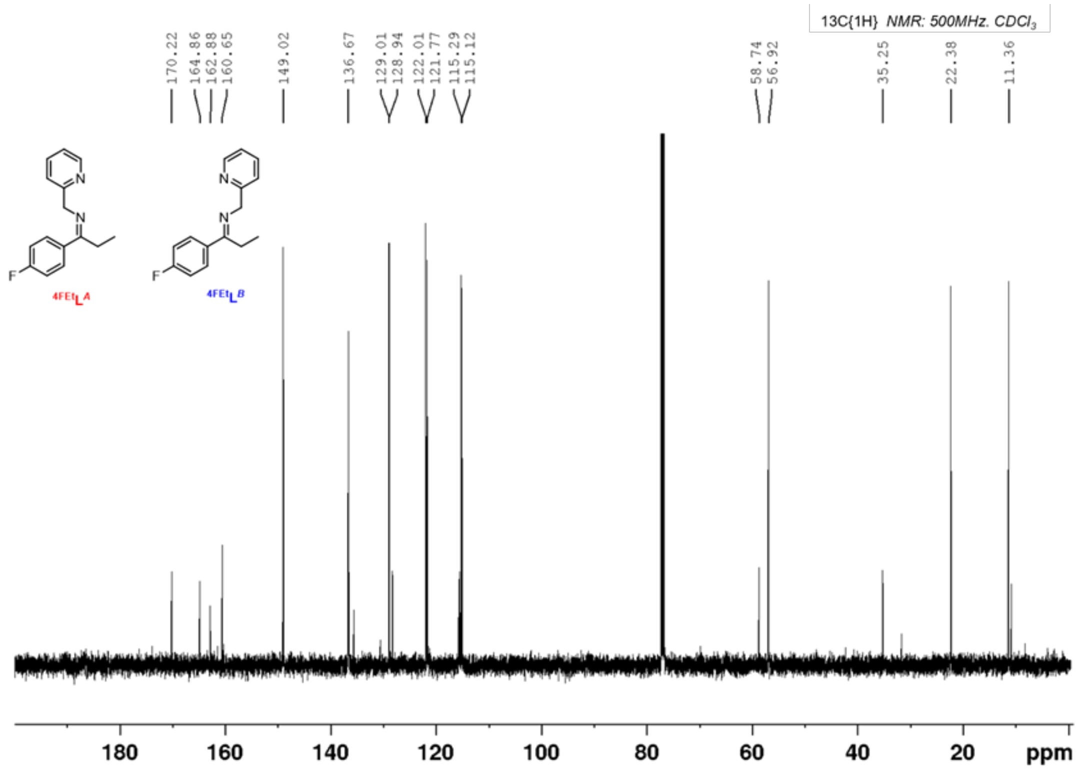
13C{1H} NMR: 500MHz. CDCl3
170.22
164.86
162.88
160.65
149.02
136.67
129.01
128.94
122.01
121.77
115.29
115.12
58.74
56.92
35.25
22.38
11.36
4FEtL A
4FEtL B
ppm

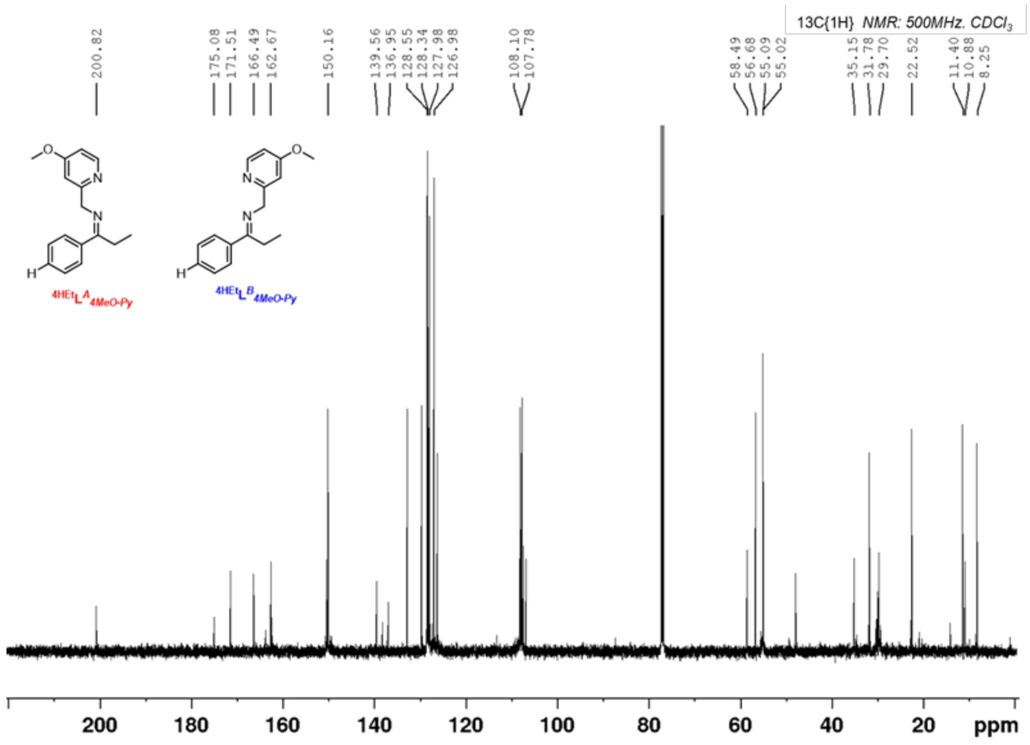
13C{1H} NMR: 500MHz. CDCl3
200.82
175.08
171.51
166.49
162.67
150.16
139.56
136.95
128.55
128.34
127.98
126.98
108.10
107.78
58.49
56.68
55.09
55.02
35.15
31.78
29.70
22.52
11.40
10.88
8.25
4HEtL A 4MeO-Py
4HEtL B 4MeO-Py
ppm

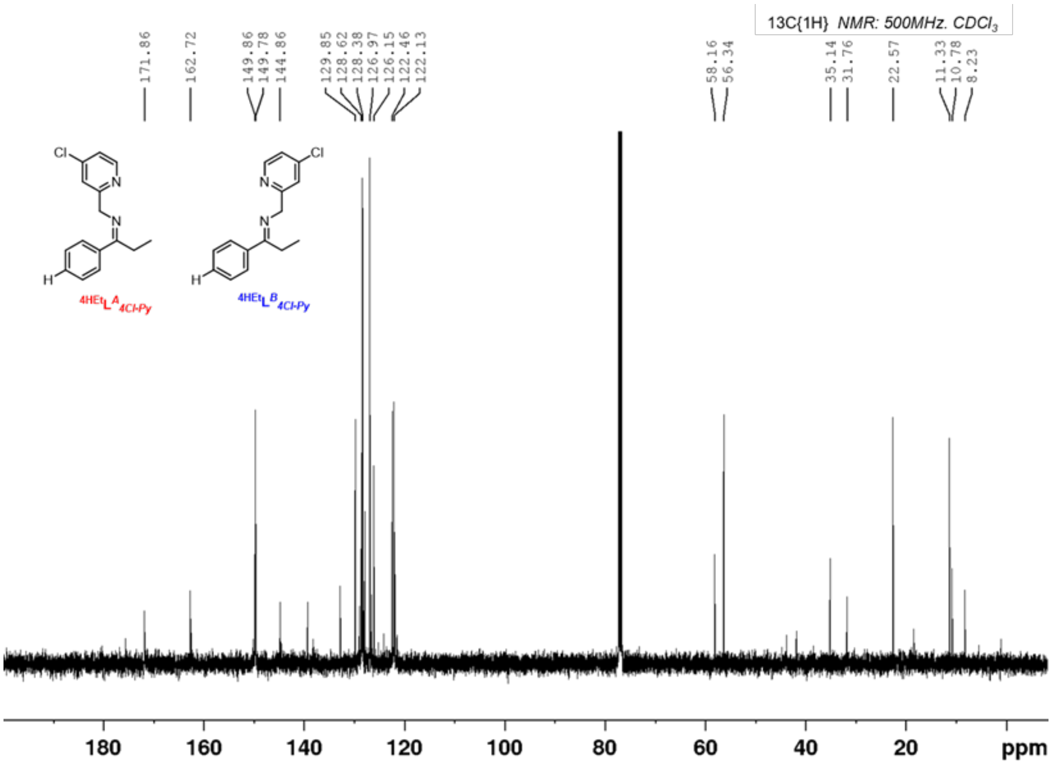
13C{1H} NMR: 500MHz. CDCl3
171.86
162.72
149.86
149.78
144.86
129.85
128.62
128.38
126.97
126.15
122.46
122.13
58.16
56.34
35.14
31.76
22.57
11.33
10.78
8.23
Cl
N
H
4HEtL A 4Cl-Py
4HEtL B 4Cl-Py
180
160
140
120
100
80
60
40
20
ppm

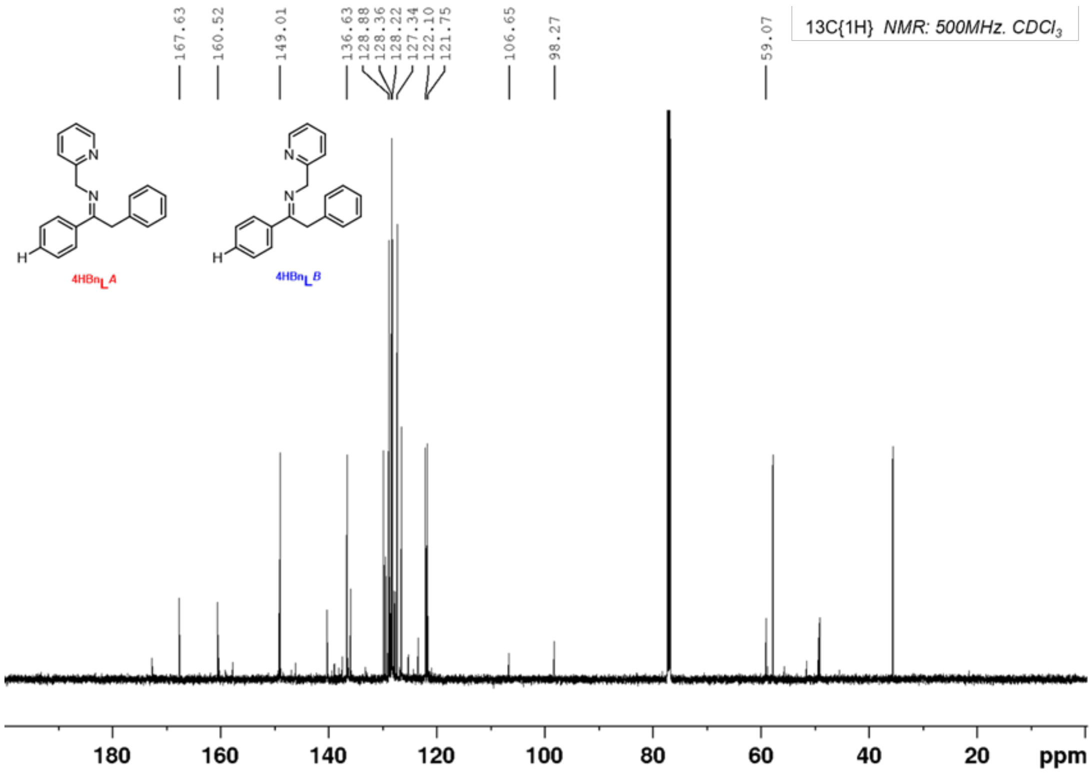
13C{1H} NMR: 500MHz. CDCl3
167.63
160.52
149.01
136.63
128.88
128.36
128.22
127.34
122.10
121.75
106.65
98.27
59.07
N
H
4HBnL A
4HBnL B
180
160
140
120
100
80
60
40
20
ppm

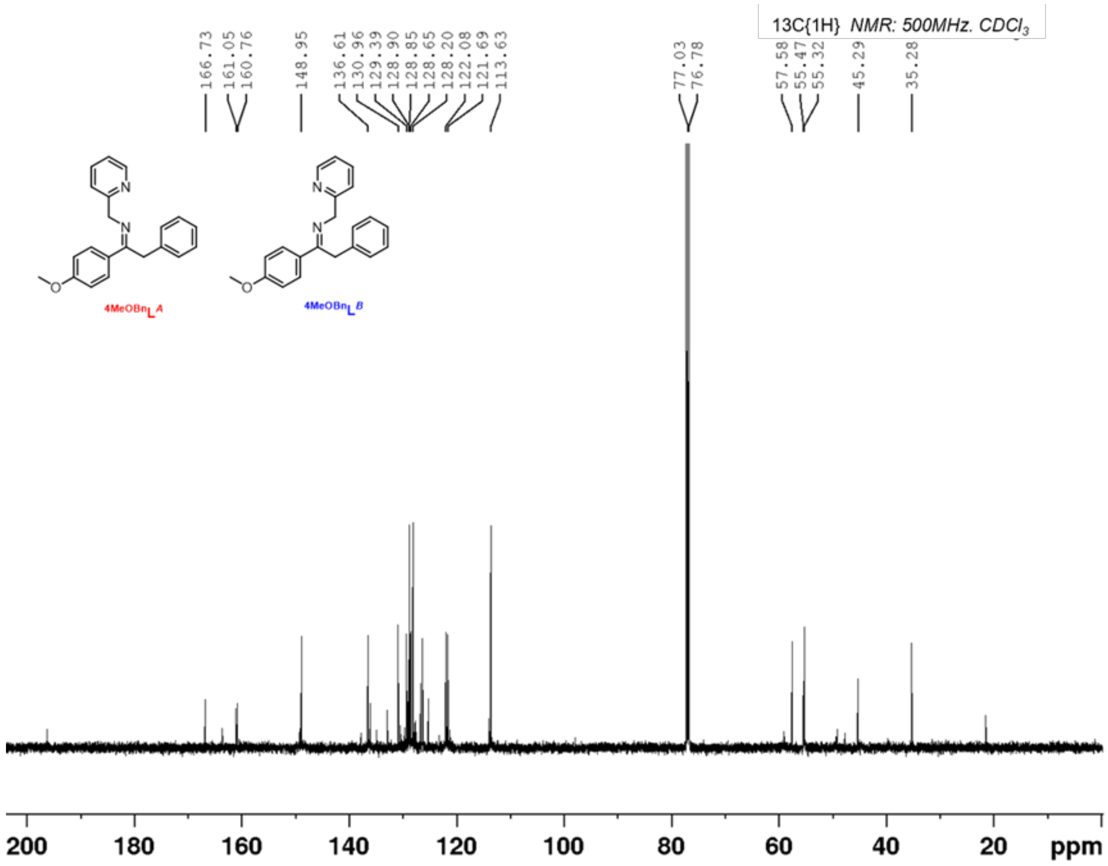
13C{1H} NMR: 500MHz. CDCl3
166.73
161.05
160.76
148.95
136.61
130.96
129.39
128.90
128.85
128.65
128.20
122.08
121.69
113.63
77.03
76.78
57.58
55.47
55.32
45.29
35.28
4MeOBnL A
4MeOBnL B
200
180
160
140
120
100
80
60
40
20
ppm

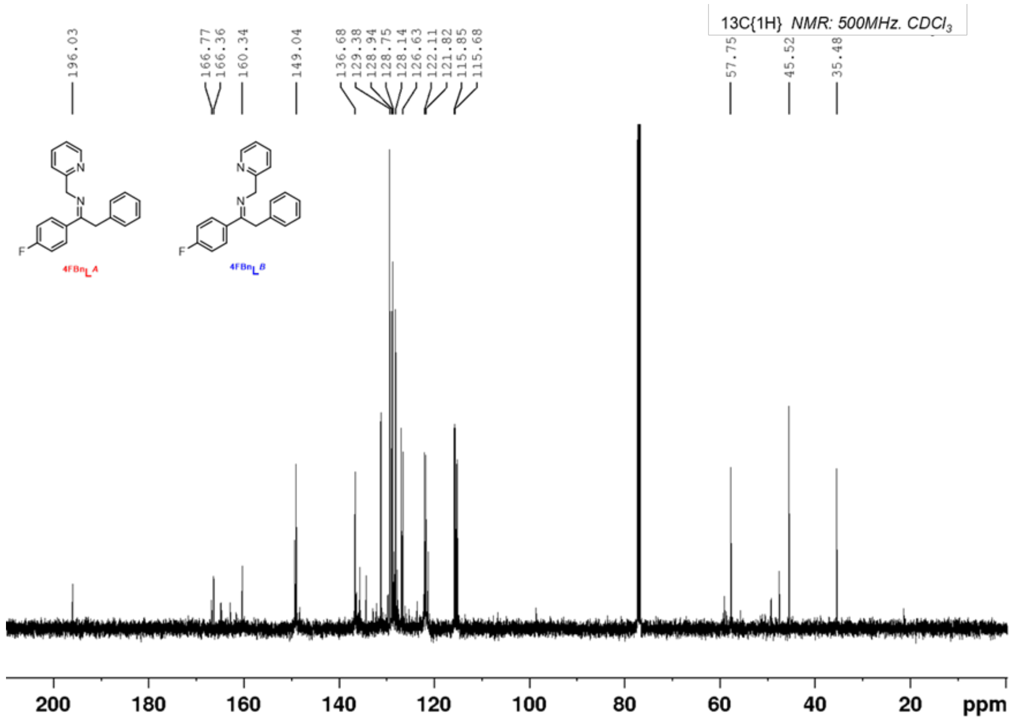
13C{1H} NMR: 500MHz. CDCl3
196.03
166.77
166.36
160.34
149.04
136.68
129.38
128.94
128.75
128.14
126.63
122.11
121.82
115.85
115.68
57.75
45.52
35.48
4FBnL A
4FBnL B
200
180
160
140
120
100
80
60
40
20
ppm

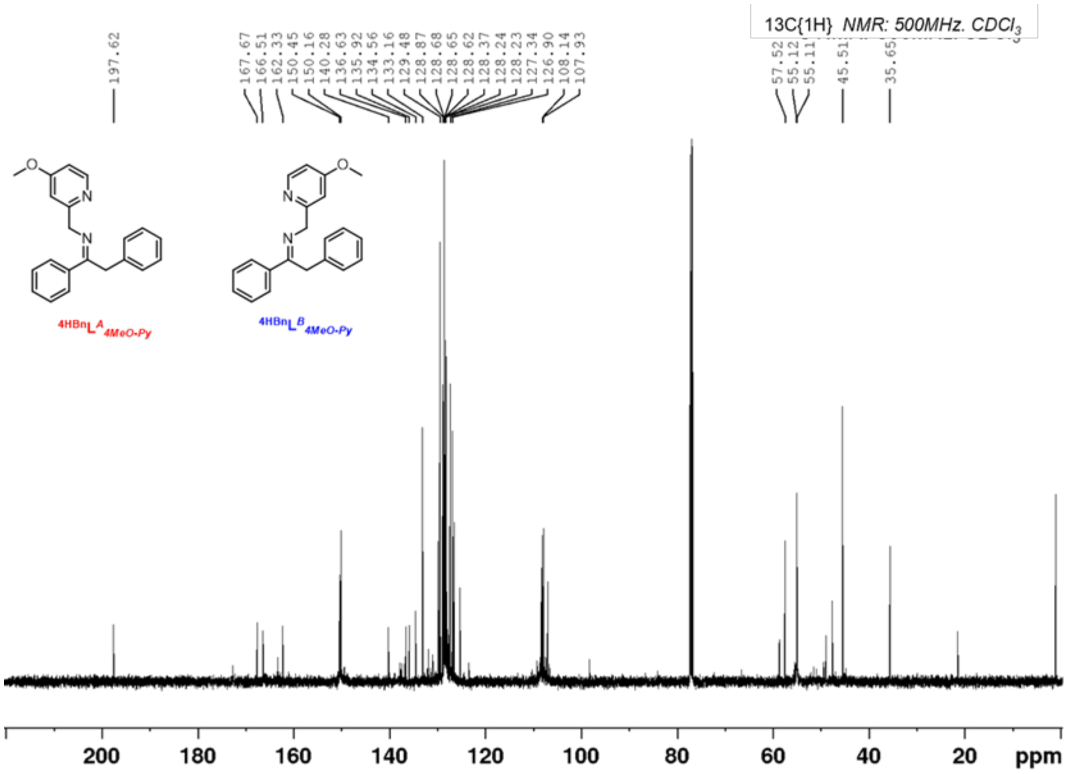
13C{1H} NMR: 500MHz. CDCl3
197.62
167.67
166.51
162.33
150.45
150.16
140.28
136.63
135.92
134.56
133.16
129.48
128.87
128.68
128.65
128.62
128.37
128.24
128.23
127.34
126.90
108.14
107.93
57.52
55.12
55.11
45.51
35.65
4HBnL A 4MeO-Py
4HBnL B 4MeO-Py
200
180
160
140
120
100
80
60
40
20
ppm

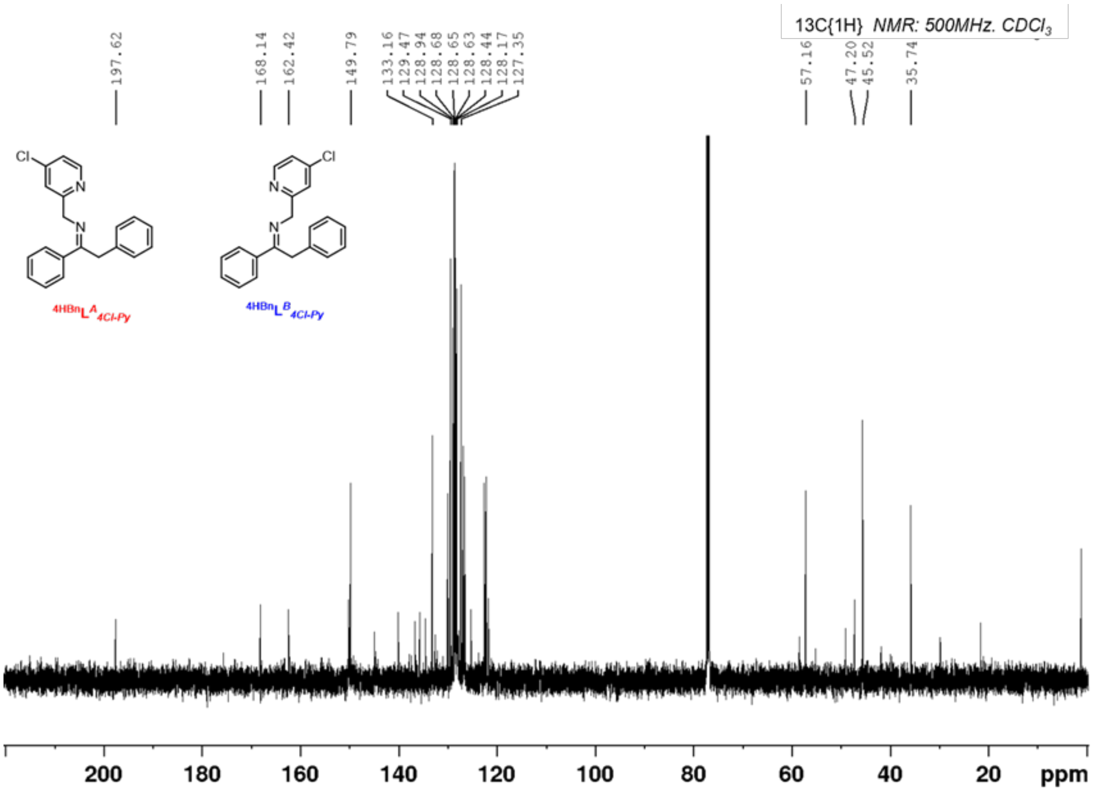
13C{1H} NMR: 500MHz. CDCl3
197.62
168.14
162.42
149.79
133.16
129.47
128.94
128.68
128.65
128.63
128.44
128.17
127.35
57.16
47.20
45.52
35.74
4HBnL A 4Cl-Py
4HBnL B 4Cl-Py
200
180
160
140
120
100
80
60
40
20
ppm

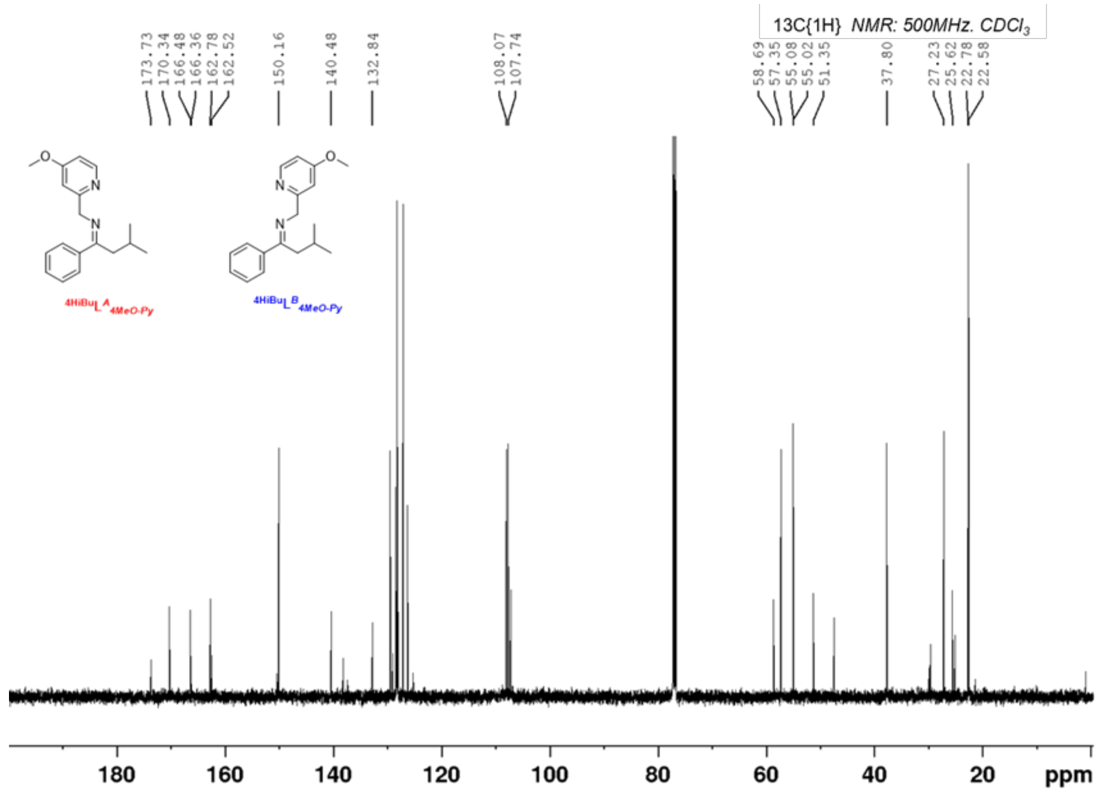
13C{1H} NMR: 500MHz. CDCl3
173.73
170.34
166.48
166.36
162.78
162.52
150.16
140.48
132.84
108.07
107.74
58.69
57.35
55.08
55.02
51.35
37.80
27.23
25.62
22.78
22.58
4HiBuL A 4MeO-Py
4HiBuL B 4MeO-Py
180
160
140
120
100
80
60
40
20
ppm

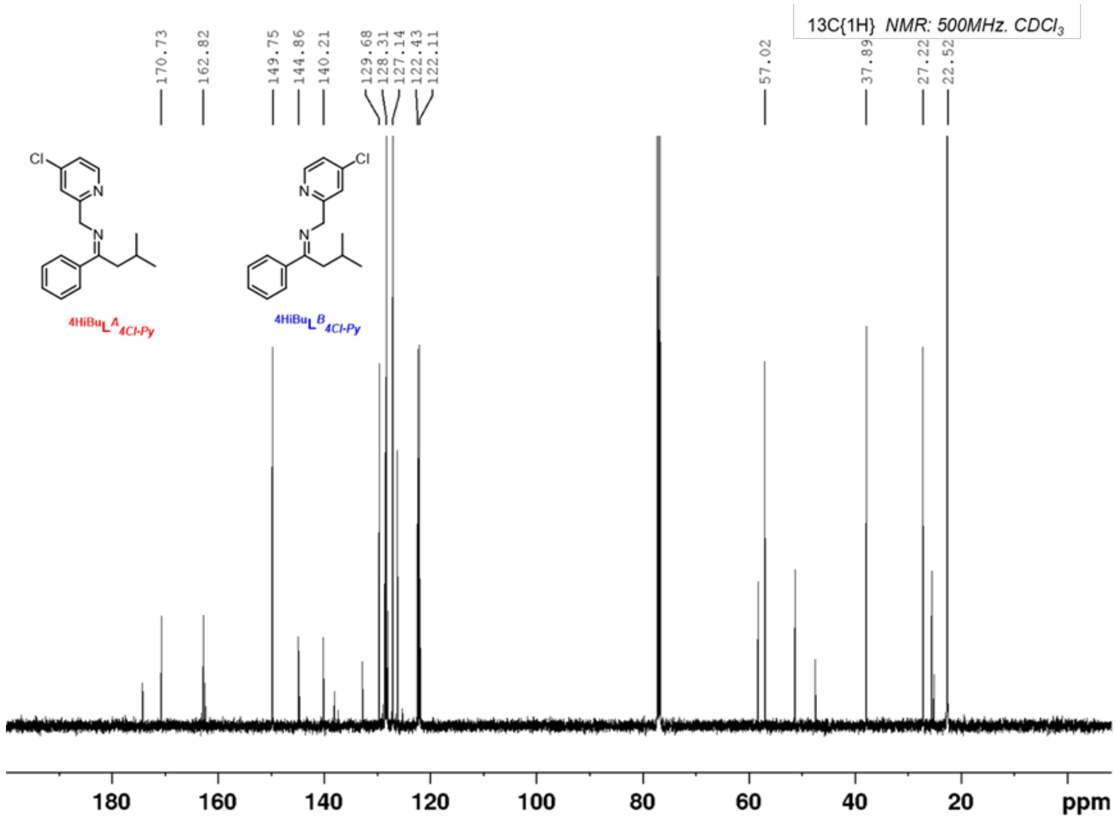
13C{1H} NMR: 500MHz. CDCl3
170.73
162.82
149.75
144.86
140.21
129.68
128.31
127.14
122.43
122.11
57.02
37.89
27.22
22.52
Cl
4HiBuL A 4Cl-Py
4HiBuL B 4Cl-Py
180
160
140
120
100
80
60
40
20
ppm

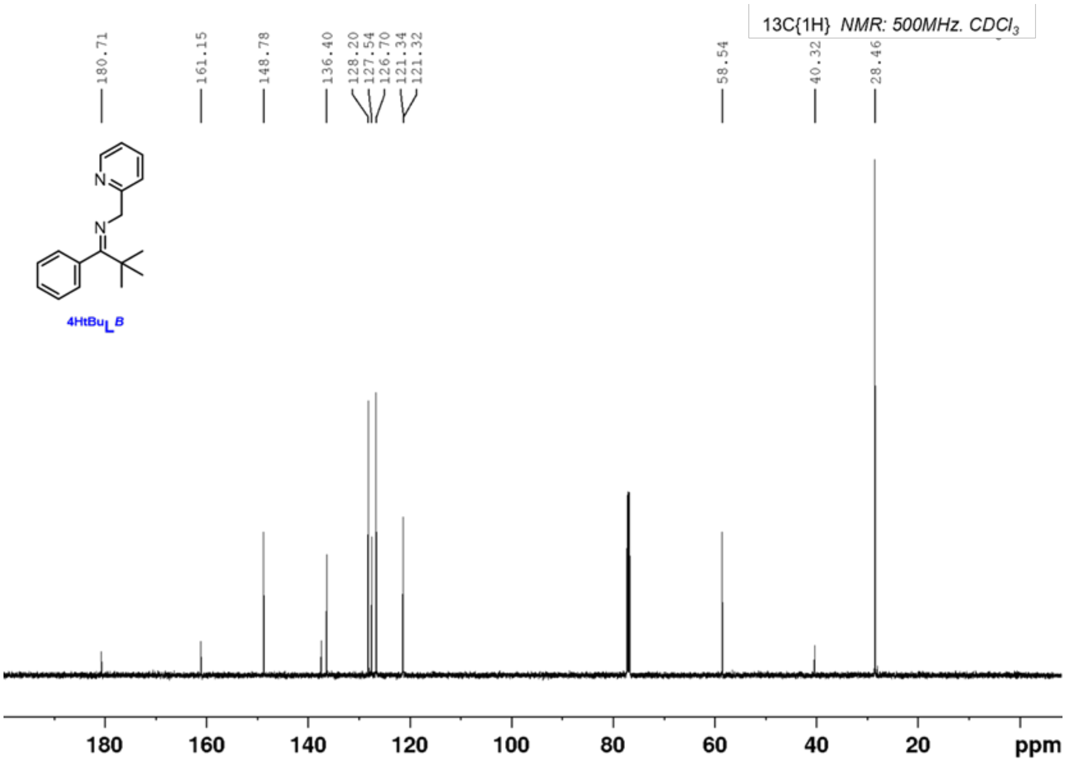
13C{1H} NMR: 500MHz. CDCl3
180.71
161.15
148.78
136.40
128.20
127.54
126.70
121.34
121.32
58.54
40.32
28.46
N
N
4HtBuL B
180
160
140
120
100
80
60
40
20
ppm

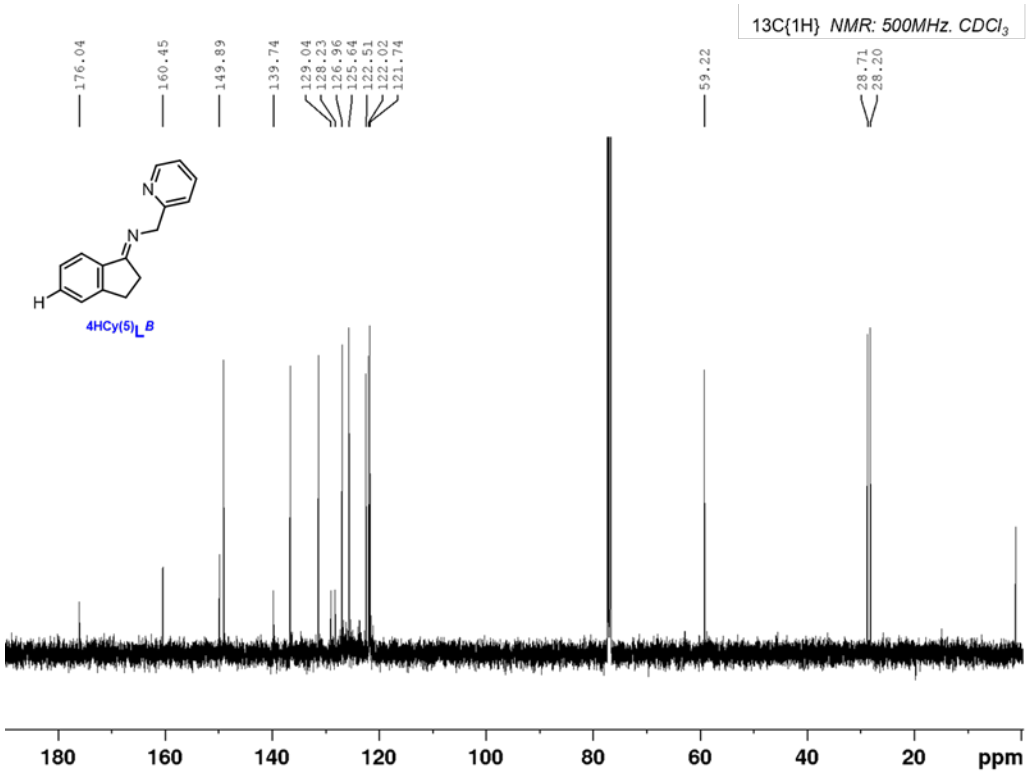
13C{1H} NMR: 500MHz. CDCl3
176.04
160.45
149.89
139.74
129.04
128.23
126.96
125.64
122.51
122.02
121.74
59.22
28.71
28.20
N
N
H
4HCy(5)L B
180
160
140
120
100
80
60
40
20
ppm

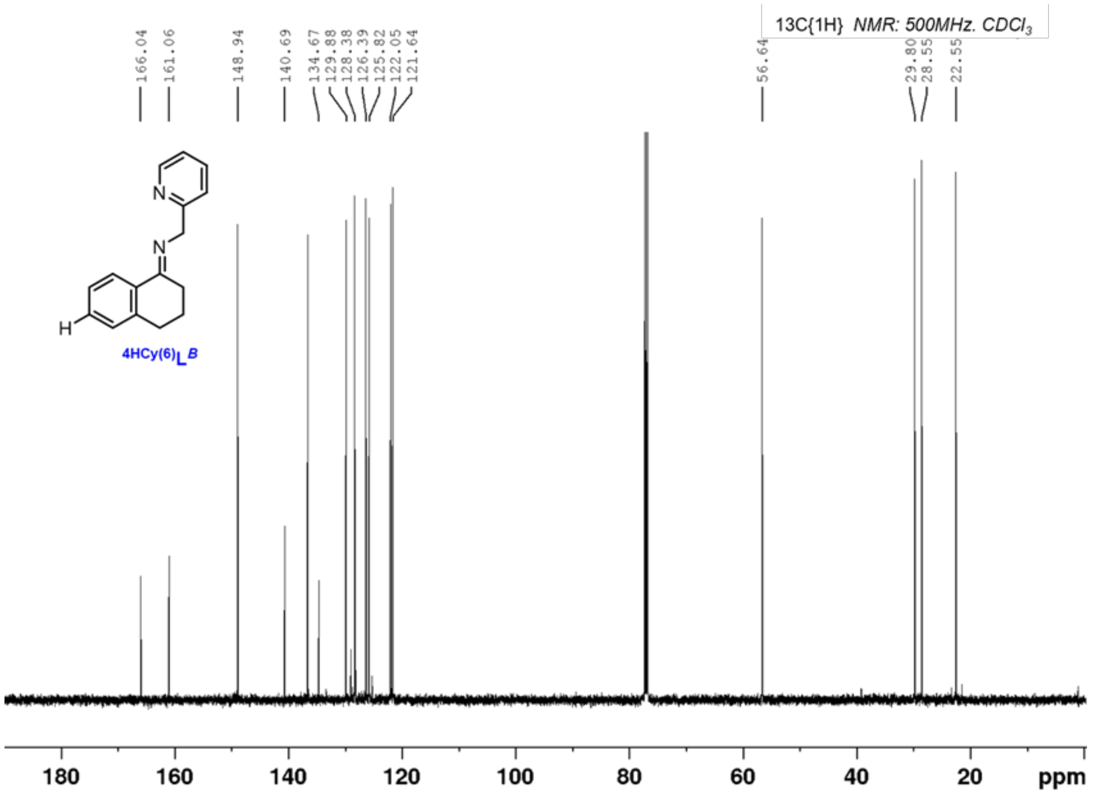
13C{1H} NMR: 500MHz. CDCl3
166.04
161.06
148.94
140.69
134.67
129.88
128.38
126.39
125.82
122.05
121.64
56.64
29.80
28.55
22.55
N
N
H
4HCy(6)LB
180
160
140
120
100
80
60
40
20
ppm

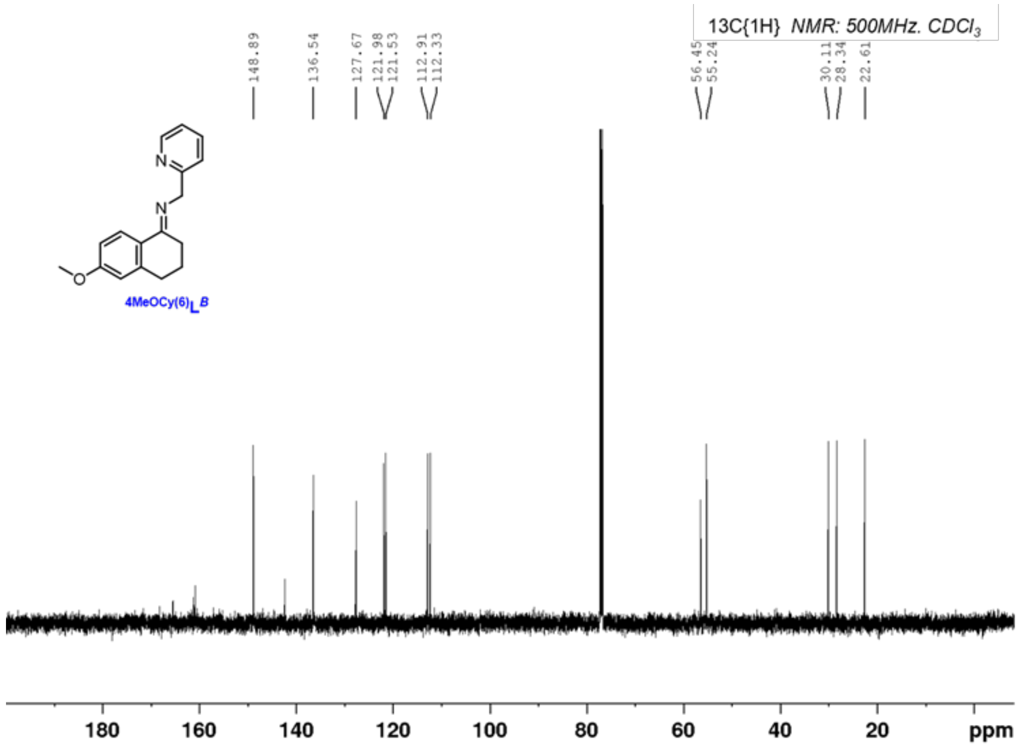
13C{1H} NMR: 500MHz. CDCl3
148.89
136.54
127.67
121.98
121.53
112.91
112.33
56.45
55.24
30.11
28.34
22.61
N
N
O
4MeOCy(6)LB
180
160
140
120
100
80
60
40
20
ppm

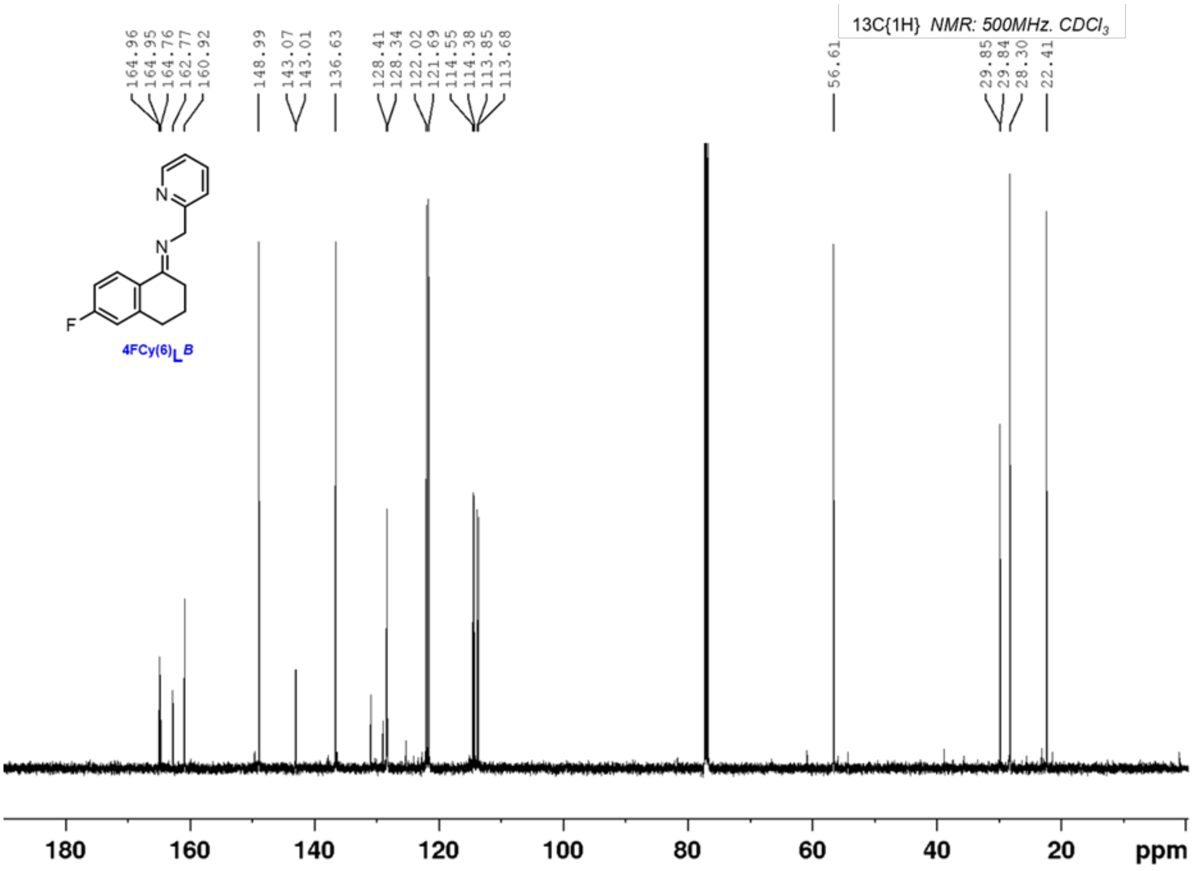
13C{1H} NMR: 500MHz. CDCl3
164.96
164.95
164.76
162.77
160.92
148.99
143.07
143.01
136.63
128.41
128.34
122.02
121.69
114.55
114.38
113.85
113.68
56.61
29.85
29.84
28.30
22.41
N
N
F
4FCy(6)L B
180
160
140
120
100
80
60
40
20
ppm

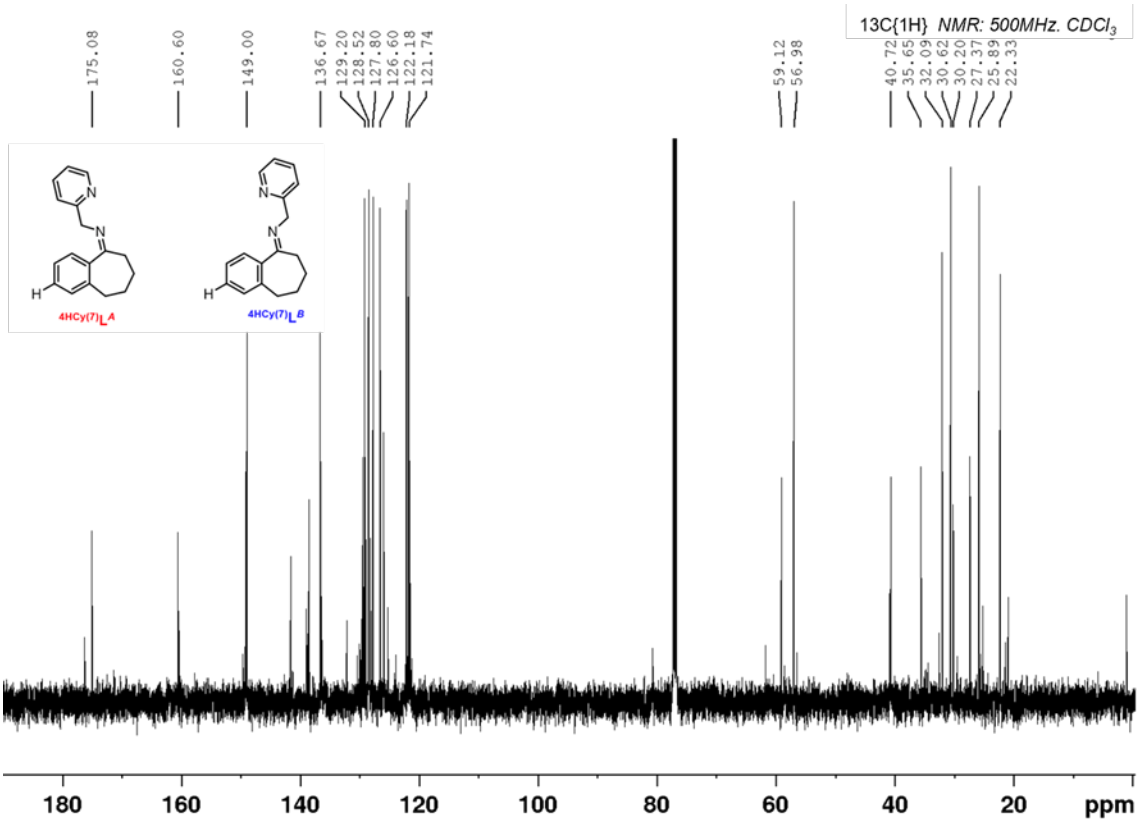
13C{1H} NMR: 500MHz. CDCl3
175.08
160.60
149.00
136.67
129.20
128.52
127.80
126.60
122.18
121.74
59.12
56.98
40.72
35.65
32.09
30.62
30.20
27.37
25.89
22.33
N
N
H
4HCy(7)L A
4HCy(7)L B
180
160
140
120
100
80
60
40
20
ppm

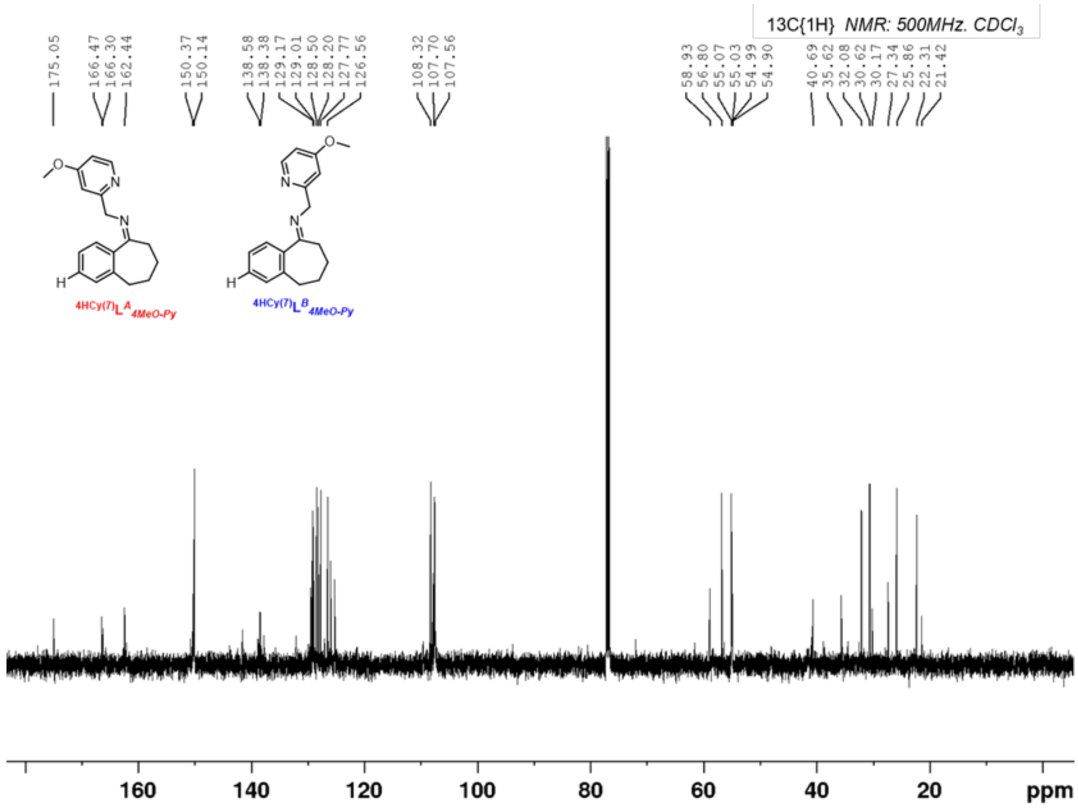
13C{1H} NMR: 500MHz. CDCl$_3$
175.05
166.47
166.30
162.44
150.37
150.14
138.58
138.38
129.17
129.01
128.50
128.20
127.77
126.56
108.32
107.70
107.56
58.93
56.80
55.07
55.03
54.99
54.90
40.69
35.62
32.08
30.62
30.17
27.34
25.86
22.31
21.42
4HCy(7)L$^A_{4MeO-Py}$
4HCy(7)L$^B_{4MeO-Py}$
160
140
120
100
80
60
40
20
ppm

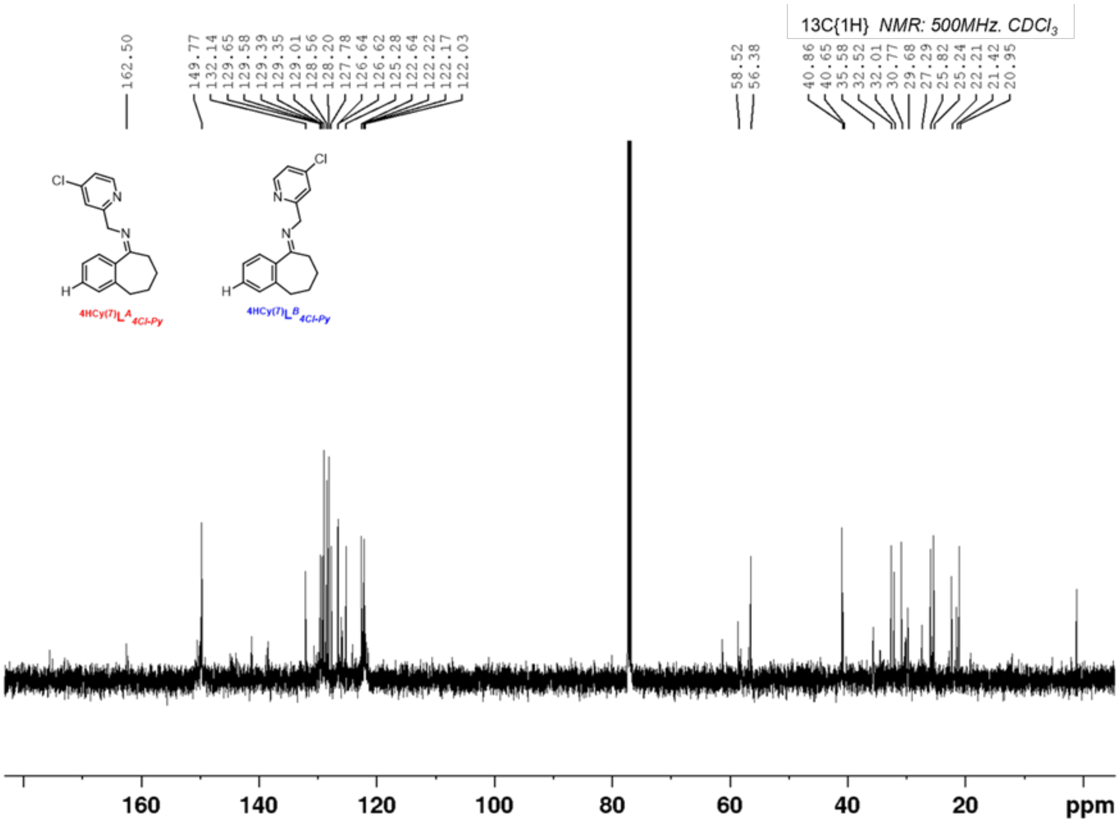
13C{1H} NMR: 500MHz. CDCl$_3$
162.50
149.77
132.14
129.65
129.58
129.39
129.35
129.01
128.56
128.20
127.78
126.64
126.62
125.28
122.64
122.22
122.17
122.03
58.52
56.38
40.86
40.65
35.58
32.52
32.01
30.77
29.68
27.29
25.82
25.24
22.21
21.42
20.95
Cl
4HCy(7)L$^A_{4Cl-Py}$
4HCy(7)L$^B_{4Cl-Py}$
160
140
120
100
80
60
40
20
ppm

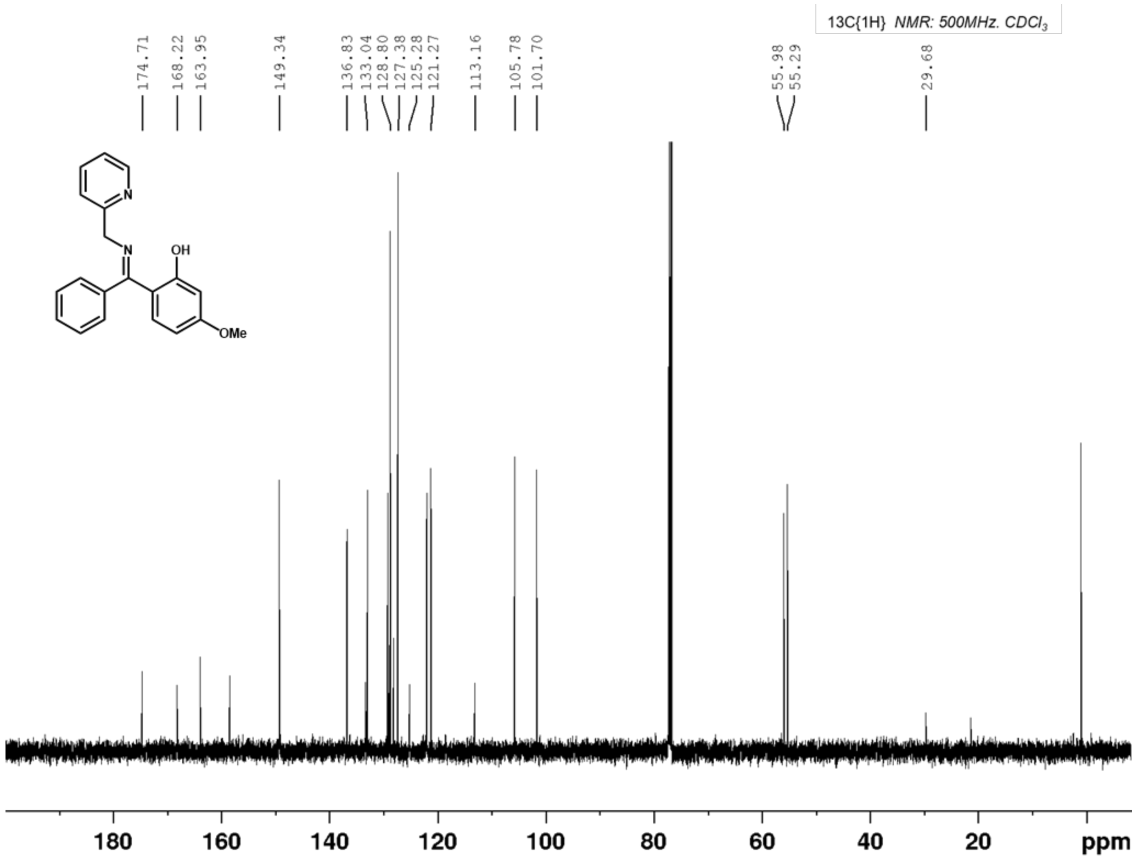
13C{1H} NMR: 500MHz. CDCl3
174.71
168.22
163.95
149.34
136.83
133.04
128.80
127.38
125.28
121.27
113.16
105.78
101.70
55.98
55.29
29.68
N
OH
OMe
180
160
140
120
100
80
60
40
20
ppm

8. References

[1] R. Trammell, L. D'Amore, A. Cordova, P. Polunin, N. Xie, M. A. Siegler, P. Belanzoni, M. Swart, I. Garcia-Bosch, *Inorg. Chem.* **2019**, *58*, 7584-7592.

[2] R. Trammell, A. Cordova, S. Zhang, S. Goswami, R. Murata, M. A. Siegler, I. Garcia-Bosch, *European Journal of Organic Chemistry* **2021**, *2021*, 4536-4540.
